# Draft genome of the Native American cold hardy grapevine *Vitis riparia* Michx. ‘Manitoba 37’

**DOI:** 10.1038/s41438-020-0316-2

**Published:** 2020-06-01

**Authors:** Sagar Patel, Michael Robben, Anne Fennell, Jason P. Londo, Dilmini Alahakoon, Roberto Villegas-Diaz, Padmapriya Swaminathan

**Affiliations:** 1 Agronomy, Horticulture and Plant Science Department and BioSNTR, South Dakota State University, Brookings, SD 57006 USA; 2 Grape Genetics Research Unit, USDA ARS, Geneva, NY 14456 USA

**Keywords:** Genome, Plant sciences

## Abstract

*Vitis riparia*, a critically important Native American grapevine species, is used globally in rootstock and scion breeding and contributed to the recovery of the French wine industry during the mid-19th century phylloxera epidemic. This species has abiotic and biotic stress tolerance and the largest natural geographic distribution of the North American grapevine species. Here we report an Illumina short-read 369X coverage, draft de novo heterozygous genome sequence of *V. riparia* Michx. ‘Manitoba 37’ with the size of ~495 Mb for 69,616 scaffolds and a N50 length of 518,740 bp. Using RNAseq data, 40,019 coding sequences were predicted and annotated. Benchmarking with Universal Single-Copy Orthologs (BUSCO) analysis of predicted gene models found 96% of the complete BUSCOs in this assembly. The assembly continuity and completeness were further validated using *V. riparia* ESTs, BACs, and three de novo transcriptome assemblies of three different *V. riparia* genotypes resulting in >98% of respective sequences/transcripts mapping with this assembly. Alignment of the *V. riparia* assembly and predicted CDS with the latest *V. vinifera* ‘PN40024’ CDS and genome assembly showed 99% CDS alignment and a high degree of synteny. An analysis of plant transcription factors indicates a high degree of homology with the *V. vinifera* transcription factors. QTL mapping to *V. riparia* ‘Manitoba 37’ and *V. vinifera* PN40024 has identified genetic relationships to phenotypic variation between species. This assembly provides reference sequences, gene models for marker development and understanding *V. riparia*’s genetic contributions in grape breeding and research.

## Introduction

Grapes (*Vitis* spp.), used for wine, juice, table grapes, raisins, and rootstocks are the most valuable fruit crop in the world. While the cultivated species *Vitis vinifera* is the predominant species used in the industry, other wild grape species are important contributors to commercial production. In particular, several North American species have been used by breeders to develop scion and rootstock cultivars that are disease, insect, and abiotic stress tolerant^1^. However, evidence from ongoing genome sequencing projects in grapevine demonstrates high variability between cultivars and that the wild grapevine species remain under-sampled for genomic data. With the advent of sequencing technologies for crop development, such as genotype by sequencing (GBS) and RNase H2-dependent amplicon sequencing (rhAmpSeq), many crop breeders are adopting larger genomic studies^2,3^. Thus, development of grape genomic resources is invaluable for ongoing crop improvement and ongoing gene annotation and gene function analyses.

Fossil evidence of *Vitis* seed from the Rocky Mountain region of the United States date back to the Plaocene era (65.5–55.8 Mya)^4–7^. Recent nuclear DNA analyses suggest that the most recent common *Vitis* ancestor for the existing global grape species originated in North America, diversifying from the rest of Vitaceae ~28 Mya (CI 41.2, 16.2 Mya), with *Vitis* and *Muscadinia* diverging ~18 Mya^8^. The *Vitis* genus, containing about 60 inter-fertile living species, is suggested to have diversified at 12–1.3 Mya^8^. The major cultivated species, *V. vinifera* is thought to have been domesticated from its wild ancestor *V. sylvestris* in the Mediterranean region of South and East Europe^9^. There are two major centers of wild grapevine species diversity, North America and East Asia with 28 and 30 species, respectively^1,8^. In the period following the retreat of the Wisconsin glaciation (~11–10,000 years ago), which radically altered the geography of central and eastern North America, it is likely that the receding glaciers and harsh conditions allowed the eastern North American grapevine species to expand their range north into more varied and marginal conditions^8^. In eastern North America, *Vitis riparia, V. labrusca, V. aestivalis*, and *V. cinerea* developed large overlapping distributions^1^. Hybridization of these species is common in the wild; however, distinct geographic, topographic, and climatic conditions have maintained the species diversification^10^. Many breeders have utilized the biotic and abiotic stress tolerance traits in these species to produce grapes that are sustainable for production in harsh climatic and biotic conditions, whereas *V. vinifera* would be killed outright or require extraordinary viticultural intervention to maintain production^11,12^.

One of the most commonly used species in abiotic and biotic stress tolerance breeding, *V. riparia*, has the largest continental distribution of the North American species. Its range stretches, from Texas in the south to the Riding Mountains (Manitoba, Canada) in the North and from the Rocky Mountains to the East Coast^1^. *V. riparia* genotypes have been utilized extensively in rootstock and scion breeding for its freezing tolerance, disease resistance (powdery mildew, downy mildew, and, *botrytis*), and phylloxera resistance^11–14^. *V. riparia* genotypes collected from the species’ northern range have been used to develop new cultivars. Presumably due to the capture of adaptive trait complexes that allow survival under harsh winter conditions. *V. riparia* based cultivars have contributed to the expansion of grapes in cold climate, resulting in over 2400 ha (hectares) of new production and 300 new wineries in the Northern United States and Southeastern Canada^11–13,15,16^. Mining the locally adaptive traits from *V. riparia* has resulted in new cultivars that incorporate the traits of early ripening, high sugar content and maximum freezing tolerance traits^11,12,17^. While breeding new cultivars using *V. riparia* has been successful, there are large gaps in our understanding of the genetic architecture of positive and negative traits that this species brings into the breeding programs.

To fill this gap, the genome of *V. riparia* ‘Manitoba 37’ a genotype from the Riding Mountains of Manitoba, Canada was sequenced for assembly, single nucleotide polymorphism mapping, pan-generic marker panel development and genetic analysis. *V. riparia* ‘Manitoba 37’ was obtained from the breeding program at the University of Minnesota, St. Paul, MN, USA and later placed in the USDA ARS Germplasm Repository at Geneva, New York under the identifier of PI588259. The strategy undertaken here was to use the highly accurate Illumina short-read sequencing in conjunction with mate pair libraries to produce an assembly with high fidelity that matches the greater heterozygosity of the *V. riparia* ‘Manitoba 37’ genome. This genotype has been used extensively in studies to develop improved cold hardiness, photoperiod responsiveness, identify the female allele of the *Vitis* sex locus, and break the linkage drag between high malic acid and soluble solids in segregating populations^2,3,17–19^. In addition to breeding, this genotype has also been used extensively for physiological, proteomic, transcriptomic, and metabolomic analyses and GBS mapping studies^2,3,20–22^.

## Results

We present here, a detailed analysis of stress tolerant *V. riparia* ‘Manitoba 37’ genome assembly, using short-read Illumina data. First the genetic relationship of *V. riparia* ‘Manitoba 37’ and USDA ARS Germplasm Repository *V. riparia* materials, originally collected throughout *V. riparia’s* native range, was examined using informative SNPs. Secondly, a de novo assembly was developed for this heterozygous species using paired-end short-read libraries totaling 369.7X coverage. Gene prediction was conducted using RNASeq data from multiple tissues and experiments. Finally, the final assembly and gene models were compared with the reference genome *V. vinifera* PN40024 12X.2 and V3 annotation and other recent *Vitis* cultivar assemblies and gene conservation was analyzed. In addition to validating the quality of the *V. riparia* ‘Manitoba 37’ short-read assembly, we conducted analysis of the *WRKY* domain (*WRKY*), myelobsastosis (*MYB*), and ethylene response factor (*ERF*) transcription factor (TF) gene families and evaluated transposon composition in *V. riparia* versus *V. vinifera* genomes. Sequenced SNP markers from a F2 mapping population, identified by aligning genotype sequence data to *V. vinifera*, were used to test the fidelity and utility of this *V. riparia* genome^2^. The SNP markers were aligned to the *V. riparia* ‘Manitoba 37’ and *V. vinifera* genomes and quantitative trait loci regions over genic locations were mapped to the genomes using *V. riparia* phenotypes.

### Genetic analysis of natively collected *V. riparia*

**Fig. 1:**
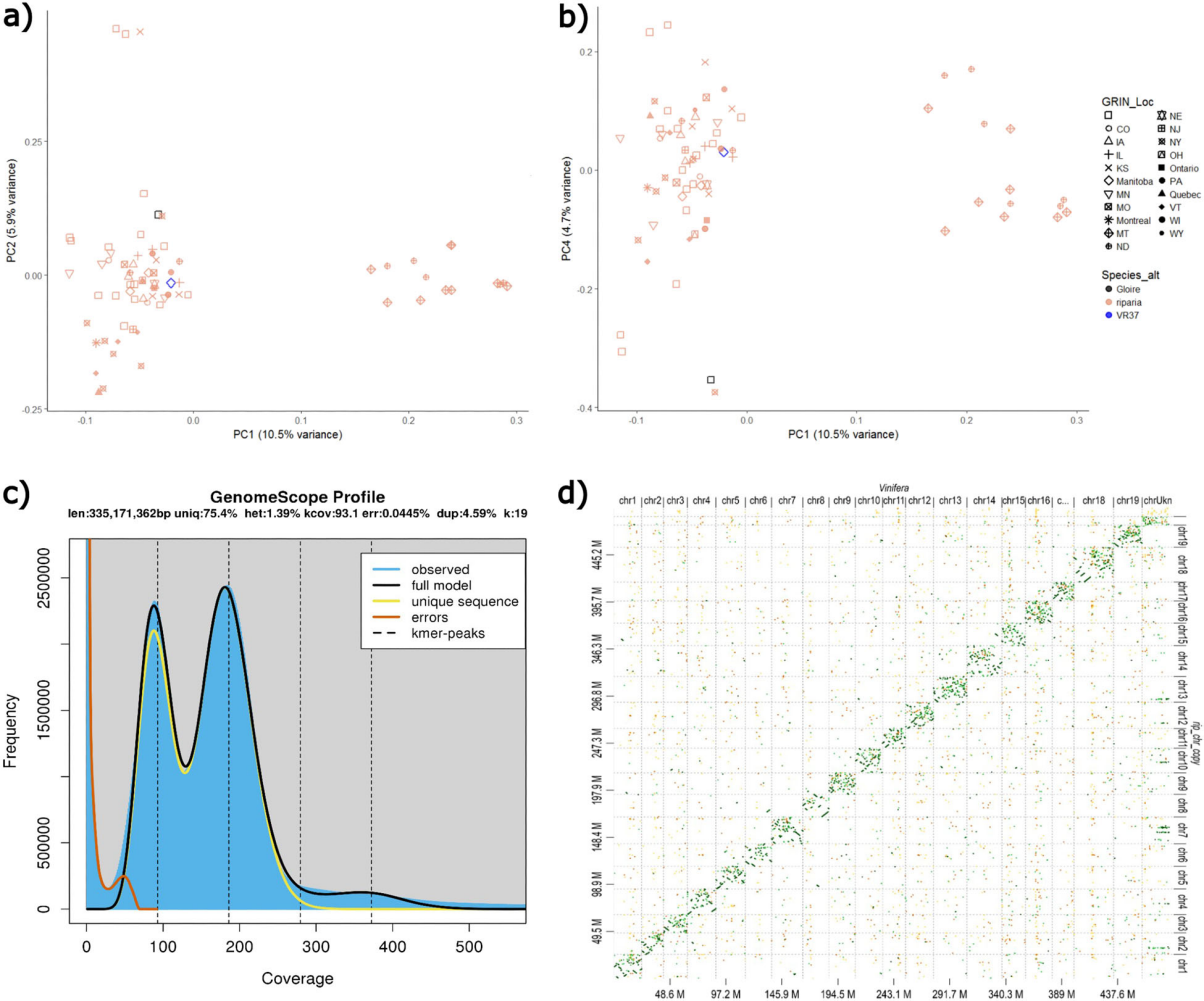
*V. riparia* genetic and assembly characterization. **a**, **b** Principal component analysis of informative SNPs in 68 *V. riparia* individuals in the USDA ARS Geneva New York germplasm repository. Symbols represent sample origin by state (United States) and Canadian province as noted in the USDA Germplasm Resource Information Network. Open squares indicate individuals with unknown geographic origin. *V. riparia* ‘Manitoba 37’ and *V. riparia*’Gloire de Montpellier’ (unknown geographic origin) are represented by blue diamond and black square, respectively. SNP data was derived from GBS data that was previously reported in Klein et al.^10^. **a** Principal components one and two, 10.6% and 5.9% of variation, respectively. **b** Presents principal components one and four, 10.6% and 4.5% of variation, respectively. **c** K-mer analysis of *V. riparia* ‘Manitoba 37’ genome. The 19 k-mer was carried out with 262.3X coverage by Jellyfish and heterozygosity obtained by GenomeScope. The first peak located at coverage 89X corresponds to the heterozygous and the second peak at 184X corresponds to the homozygous peak. The genome contains 1.39% heterozygosity (identified with 262.3X coverage). **d** Dot plot of global alignment between *V. vinifera* PN40024 on the *x*-axis and *V. riparia* ‘Manitoba 37’ on the *y*-axis

The genetic relationship of *V. riparia* ‘Manitoba 37’ relative to other *V. riparia* in the USDA ARS Germplasm Repository, Geneva, NY USA indicated that ‘Manitoba 37’ is representative of the *V. riparia* collected throughout its native range (Fig. 1a, b; Supplementary Fig. 1). Principal component analysis (PCA) of SNP data from 68 *V*. *riparia* samples demonstrated that *V. riparia* diversity is best described as two separate clusters. Both *V. riparia* ‘Manitoba 37’ and the important rootstock cultivar *V. riparia* ‘Gloire de Montpellier’ are in the primary cluster, and a secondary cluster is made up of species from the Northwestern edge of *V. riparia’s* range (Fig. 1a, b). This split is not observed in PCA2vsPCA3 (Supplementary Fig. 1). Further differentiation between ‘Manitoba 37’ and ‘Gloire de Montpellier’ and the secondary cluster can be observed in PCA1vsPCA4 (Fig. 1a, b).

### De novo heterozygous assembly and validation

Using Illumina HiSeq (Illumina, USA) short reads and three mate-pair libraries of varying insert sizes, we generated 2295.4 M raw reads for the *V. riparia* diploid genome draft assembly. A 1.39% heterozygosity was estimated from the unprocessed short-reads with the Jellyfish plot showing the heterozygous peak slightly lower than the homozygous peak (Fig. 1c). The initial assembly had a N50 of 512,151 bp and 13.63% scaffold %N. After gap closing, the final *V. riparia* assembly was 494.6 Mb in 69,616 scaffolds, with an N50 of 518,740 bp and a scaffold N of 3.57% (Table 1 and Supplementary Table 1). The *V. riparia* ‘Manitoba 37’ and *V. vinifera* PN40024 alignment are shown as a dot plot (Fig. 1d).

**Table 1:** *V. riparia* ‘Manitoba 37’ assembly and gene prediction statistics

Assembly statistics	Details	*V. riparia* assembly (≥500 bp)
	Number of scaffolds	69,616
	Total size of scaffolds	494,682,949
	Longest scaffold	5,123,774
	Number of scaffolds >1 K nt	31,418
	Number of scaffolds >10 K nt	1760
	Number of scaffolds >100 K nt	742
	Number of scaffolds >1 M nt	97
	Scaffold %*N*	3.57
	N50 scaffold length	518,740
	NG50 scaffold length	535,518
	N50 contig length	61,142
Gene prediction		
	Total CDS and protein	40,019
	Total CDS bp	39,395,553
	Mean CDS length	984.4
	Longest CDS length	16,443
	Total protein length	13,093,122
	Mean protein length	327.2
	Longest protein length	5480

In addition to the Assemblathon statistics, 96% of the filtered reads mapped back to the *V. riparia* ‘Manitoba 37’ genome assembly with zero mismatch. REAPR analysis of genome assembly accuracy using mate paired-end reads found evidence (low mate paired coverage) for potential mis-assembly in no more than 16% of the scaffolds (Supplementary Table 2a). Mapping of EST and BAC sequences indicated that 1935 of 1974 (98%) ESTs and 3811 of 4171 (91.3%) of the BACs mapped to the assembled genome (Supplementary Table 2b). Alignment of three different *V. riparia* de novo transcriptomes with the *V. riparia* genome assembly resulted in >99% mapping of the total transcripts for each transcriptome, with >93% transcripts mapping with >90% identity and >90% coverage (Supplementary Table 2b).

### Alignment of *V. riparia* ‘Manitoba 37’ assembly with *V. vinifera* genomes

The *V. riparia* ‘Manitoba 37’ de novo scaffolds aligned well with *V. vinifera* ‘PN40024’. A total of 76% of the *V. vinifera* PN40024 scaffolds were aligned with >90% identity and >1000 bp identity and 9496 scaffolds had >95% identity with > 1000 bp alignment (Supplementary Fig. 2, Supplementary Table 3). A total of 1,607,090 high quality SNPs were called with a rate of 1 every 504 bases (Supplementary Fig. 2).

Similar alignment statistics were observed for other recent *Vitis* genomes. *V. riparia* ‘Manitoba 37’ scaffolds aligned with >90% identity and >1000 bp identity to primary-contig scaffolds of *V. vinifera* cultivars ‘Cabernet Sauvignon’, ‘Chardonnay’, and ‘Carménère’ and *V. riparia ‘*Gloire de Montpellier’ at 75%, 74%, 76.5%, and 90.6%, respectively (Supplementary Table 3). A zero-mismatch filtered reads mapping found only 89% of the reads mapping to ‘Gloire de Montpellier’ assembly, in contrast to 96% mapping to the ‘Manitoba 37’ assembly.

### Repeat identification and de novo gene prediction

Repeat sequences were predicted to make up 46% of the *V. riparia* ‘Manitoba 37’ assembly (Supplementary Table 4a). These repeats are predominantly LTR regions (17.68%), long interspersed nuclear elements (LINE) (4.21%), DNA elements (2.06%) and Unclassified repeats (19.95%) (Supplementary Table 4a). The de novo gene prediction using *V. riparia* ‘Manitoba 37’ RNAseq data identified 40,019 putative coding sequences with the average size of predicted coding sequence (CDS) is 984.4 bp (Table 1). A total of 1548 (96%) Benchmarking Universal Single-copy Orthologs (BUSCO) were identified in the predicted genes (Supplementary Table 4b). Species distribution of BLASTX results showed most of the predicted *V. riparia* genes correspond with *V. vinifera* (Supplementary Table 4c). Over 89% of coding sequences were fully annotated using the *V. vinifera ‘*PN40024’ 41,733 CDS. A total of 41,189 (99%) *V. vinifera* ‘PN40024’ coding sequences aligned to the *V. riparia* ‘Manitoba 37’ assembly. Of these 33,370 (80%) *V. vinifera* CDS had >90% identity and >70% coverage (Supplementary Table 2b) with *V. riparia* predicted gene models. In total, 5,596 enzymes were identified from the six enzyme classes (Supplementary Table 4d). Gene ontology characterization of the *V. riparia* coding sequences of *V. riparia* functionally annotated the majority of the CDS (Biological Process (45,720), Cellular component (34,793), and Molecular Function (29,046)) (Supplementary Table 4e). All coding sequences were queried against the InterPro database and 35,643 sequences were annotated with 20,382 coding sequences containing GO identifiers. Analysis of KEGG annotations resulted in 133 different pathways covering 1,233 coding sequences (Supplementary Table 4f).

### Comparative genome analysis

**Fig. 2:**
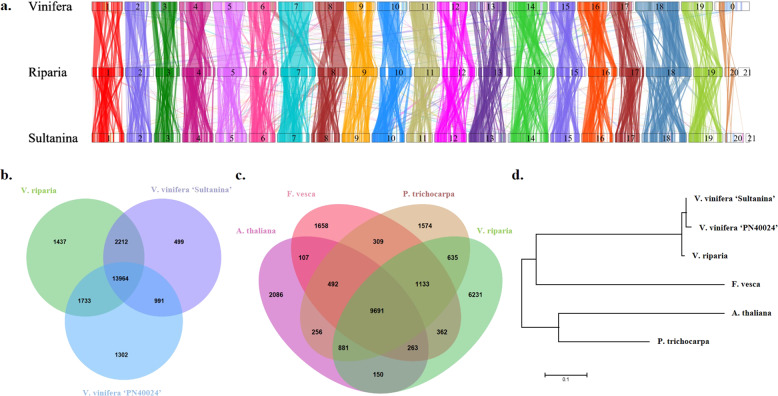
Comparative analysis of *V. riparia* ‘Manitoba 37’ with other plant species. **a** *V. riparia* ‘Manitoba 37’ synteny with *V. vinifera* ‘PN40024’ and *V. vinifera* ‘Sultanina’. **b** Core orthologous genes present in *V. vinifera* ‘PN40024’, *V. vinifera* ‘Sultanina’ and *V. riparia* ‘Manitoba 37’. **c** Orthologous genes in *V. riparia* ‘Manitoba 37’, *A. thaliana, F. vesca*, and *P. trichocarpa*. **d** Neighbor-joining phylogenetic tree of *V. riparia* ‘Manitoba 37’ with other species based on the orthologous genes

Synteny comparisons were made for *V. riparia* ‘Manitoba 37’, *V. vinifera* ‘PN40024’ and *V. vinifera* ‘Sultanina’ and genome assemblies in the Fabidae and Malvidae orders of the superrosids (*Medicago trunculata*, and *Fragaria vesca* and *Populus trichocarpa* and *Arabidopsis thaliana* respectively). The *V. riparia* scaffolds arranged in pseudo-molecules were aligned with each plant genome separately (Supplementary Table 5a, c, d). The greatest number of extended conserved syntenic blocks (>10 kb) was observed with the reference genome *V. vinifera* ‘PN40024’ and then *V. vinifera* ‘Sultanina’ (Fig. 2a; Supplementary Table 5c). *Medicago* had the highest number of syntenic blocks for the examined Rosid genomes, followed by *Populus* and *Fragaria*; the lowest synteny was observed with *Arabidopsis thaliana* (Supplementary Table 5b, c).

A comparison of predicted *V. riparia* genes to multiple species was used to further characterize gene coverage. There were 13,964 common orthologous protein sequences shared by *V. riparia*, *V. vinifera* 12X.2 and *V. vinifera* ‘Sultanina’, and 9661 found in common with Rosid species (*Arabidopsis thaliana, Fragaria vesca* and *Populus trichocarpa*) (Fig. 2b, c, Supplementary Table 5d).

### Plant transcription factor identification

**Fig. 3:**
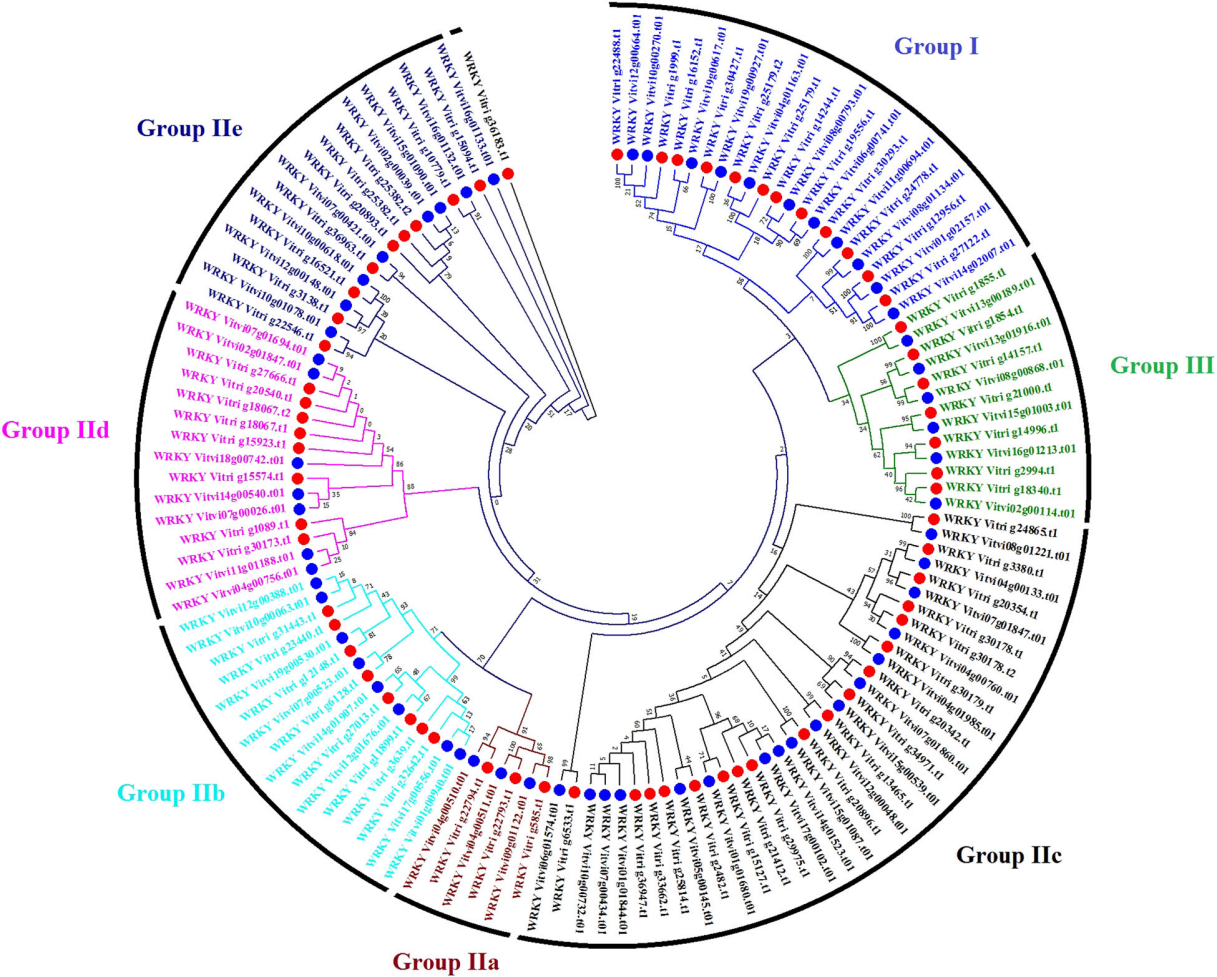
*V. riparia* ‘Manitoba 37’ and *V. vinifera* ‘PN40024’ *WRKY* transcription factors. Phylogenetic relationship of *WRKY* TFs between the two species annotated by conserved group. Red dots represent *V. riparia* ‘Manitoba 37’ genes and blue dots represent *V. vinifera* ‘PN40024’ genes. Bootstrap values displayed are at nodes. Gene id and group identity are found in Supplementary Table 7b

Plant transcription factor analysis indicated that the *V. riparia* ‘Manitoba 37’ assembly contained representatives of all the transcription factor gene families found in *V. vinifera* ‘PN40024’ (Supplementary Table 6). A total of 1,723 transcription factors from 58 families were identified in the *V. riparia* assembly. There were 67 *V. riparia WRKY* transcription factors annotated and compared phylogenetically to the *V. vinifera* WRKY and other species (Fig. 3; Supplementary Table 6a–c; 7a, b). A putative novel *WRKY* domain (WVDTDKR) was identified in the *V. riparia* gene Vitri g36183.t1; however, this putative *WRKY* domain was not shared with any *WRKY* domain in *V. vinifera* ‘PN40024’ (Fig. 3; Supplementary Table 7a–c). There was a greater number of potential *MYB* family transcription factors identified in *V. riparia* (13 *MYB* genes and 35 *MYB*-related genes) than found in *V. vinifera* ‘PN40024’ (Supplementary Table 7d). In the 25 *MYB* family subgroups, only subgroup 6 had potential gene duplications in comparison to *V. vinifera* ‘PN40024’ (Supplementary Fig. 3). Comparison of the *V. riparia* Ethylene Response Factor transcription factor (*ERF*) genes with the *V. vinifera* ‘PN40024’ *ERF* groupings indicate all groups were represented in *V. riparia;* however, there were fewer *ERF* identified in *V. riparia* ‘Manitoba 37’ than in *V. vinifera*. (Supplementary Fig. 4; Supplementary Table 7e).

### Analysis of protein families from *V. riparia* and *V. vinifera*

**Fig. 4:**
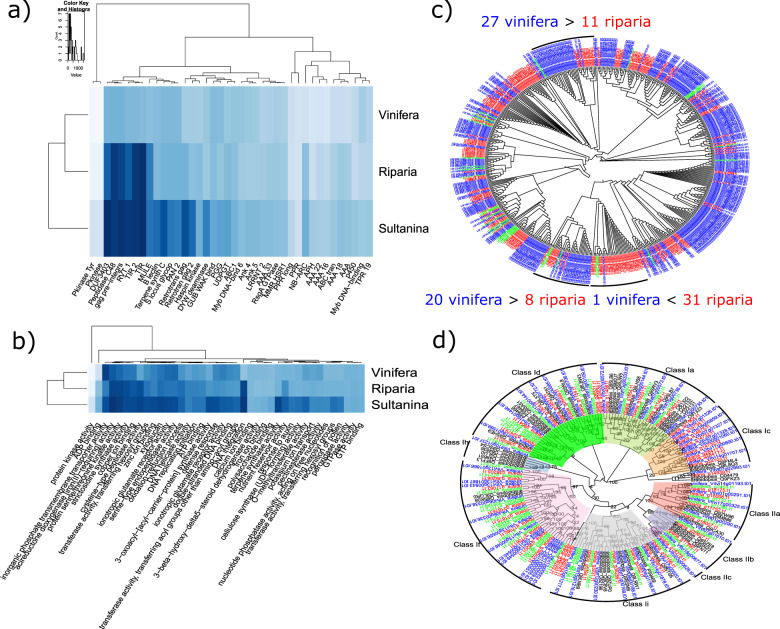
Analysis of protein families in *V. riparia* and *V. vinifera*. Heatmap displaying the differences in numbers of genes per **a** protein family or **b** Gene ontology (GO) term. **c** Protein alignment of members of the “retrotrans_gag 2” family (*LTR* retrotransposons). Monophyletic clades containing genes from *V. vinifera* ‘PN40024’ and *V. riparia* ‘Manitoba 37’ were annotated and the node containing the most recent common ancestor was indicated by black triangle. Gene id and group are found in Supplementary Table 7f. **d** Protein alignment of *LOB Domain* containing (*LBD*) family proteins, bootstrap values are at nodes. Gene id and group are found in Supplementary Table 7g

The predicted *V. vinifera* ‘PN40024’, *V. riparia ‘*Manitoba 37’, and *V. vinifera* ‘Sultanina’ genes were aligned to the protein family database (PFAM). The *V. riparia* and the seedless cultivar ‘Sultanina’ had a lower copy number of predicted genes related to transposases and transposons than *V. vinifera* (Fig. 4a). These results are supported by annotation to GO terms for each family (Fig. 4b). A closer look at the *RETROTRANS_GAG* 2 (*LTR*) transposon family shows paralogous duplication of genes in each clade that most likely occurred after the species diverged geographically (Fig. 4c, Supplementary Table 7f). A comparison of *LATERAL ORGAN BOUNDARY DOMAIN* containing proteins (*LBD*) in *V. vinifera* and *V. riparia* ‘Manitoba 37’ found high conservation similarities between members of each subfamily from both genomes (Fig. 4d, Supplementary Table 7g).

### Alignment of markers from an F2 mapping population to *V. riparia* and *V. vinifera* genome assemblies implies translocation events

SNP marker sequences for F2 mapping population derived from a single F1 (generated by crossing *V. riparia* ‘Manitoba 37’ (female; grandmother) and the cultivar ‘Seyval’ (male; grandfather)) previously identified from GBS SNP analysis against *V. vinifera* PN40024 12X.1 genome were used to evaluate the utility of the *V. riparia* genome as a reference. Alignment of the SNP marker sequence to *V. riparia* ‘Manitoba 37’ and *V. vinifera* ‘PN40024’ chromosomes indicated that 89.5% of the markers aligned to both species, while about 6.2% and 4.2% mapped uniquely to *V. riparia* or *V. vinifera*, respectively (Supplementary Fig. 5a, b, Supplementary Table 8). The number of markers that mapped was not proportional to genome or chromosome size between the two species indicating an even distribution (Supplementary Table 8). Putative rearrangements, needing further study to verify, were noted on sections of chromosomes 5, 6, and 8 appear between the two species, as well as between chromosomes 14 and 15 (Supplementary Fig. 6b).

**Fig. 5:**
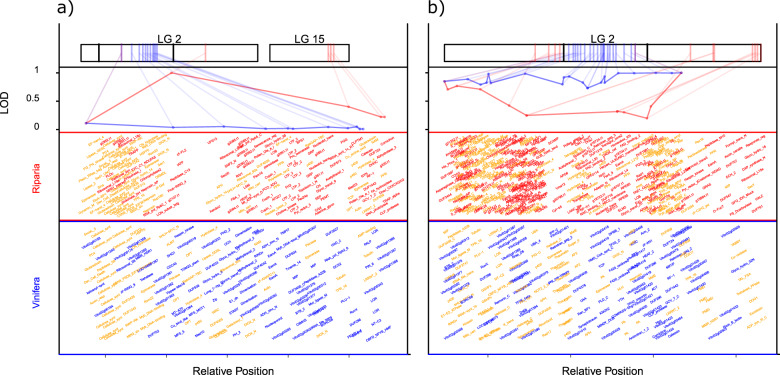
Genome browser view of genes contained within F2-derived QTL regions represented in *V. riparia* and *V. vinifera* ’PN40024’ genomes that are aligned by the GBS markers. The QTL’s represented are for **a** summer lateral critical photoperiod growth cessation and **b** flower type. In the top–middle row, markers found within a genomic region of each genome between the two flanking markers are plotted by their relative position along the *x* axis and the LOD score along the *y* axis. The LOD score at each marker is normalized and represented as values between 0 and 1 of the maximum LOD score. F2 markers present in *V. riparia* are colored red and those present in *V. vinifera* are blue. In the two bottom panels, genes are annotated by PFAM name, and genes within the QTL in both genomes are colored orange. In the top row, marker positions within the F2 genetic map are connected to the same marker position on each genome. Gene id and PFAM names are found in Supplementary Table 9

Using the aligned SNP markers and phenotype data for flower sex and summer lateral shoot cessation photoperiod response, we identified and aligned genes between flanking markers of the respective QTL (Fig. 5a, b) in the *V. riparia* pseudo-chromosomes and the *V. vinifera* ‘PN40024’ chromosomes. Genes in common between the species with similar position alignment and markers with similar LOD score as well as differential marker LOD and gene distribution differences are apparent.

## Discussion

The first published *Vitis* reference genome for cultivated grapevine was produced using a highly homozygous inbred line derived from *V. vinifera* ‘Pinot noir’^23^. While immensely useful in early genomic studies in grape, this inbred individual does not reflect the high heterozygosity found in other grapevine species and cultivars. The principal component analysis showed that *V. riparia* ‘Manitoba 37’ is likely a good representative reference for the *V. riparia* genome as it clusters with most of the collected diversity of this species. Both *V. riparia* ‘Manitoba 37’ and the other sequenced *V. riparia* genome ‘Gloire de Montpellier’^24^ are part of this core diversity. When examined at other principal component levels, ‘Manitoba 37’ appears to be more genetically related to Midwest and Eastern collections of *V. riparia* than the *V. riparia ‘*Gloire de Montpellier’. Unfortunately, geographic passport data for the ‘Gloire’ variety is unknown and its representation of *V. riparia* may not be verifiable. The analysis presented here using only *V. riparia* data also suggests, for the first time, that there may be two clades of *V. riparia*, with the North Dakota and Montana genotypes forming the second subclade of genotypes. This result was not observed when examining GBS based relationship data as presented by Klein et al^10^, presumably due to the inclusion of a much wider collection of SNP data across the *Vitis* genus in that study. Our results demonstrate the potential for cryptic speciation within *V. riparia* for this Northwestern subclade of genotypes, or loss of diversity due to population extinction or poor representation in the USDA germplasm collection for the full diversity of the species.

Recently, an improved reference genome *V. vinifera* ‘PN40024’ was published with improved gene prediction and annotation^25^. Genomes for other *V. vinifera* cultivars, as well as the rootstock variety ‘Riparia Gloire de Montpellier’ have recently been produced as well^24,26–28^. However, there remains a dearth of genome data available for wild grapevine species, and those being used in breeding programs in particular. Illumina short-read are very accurate and preserve the heterozygosity of the genome sequence; however, they provide a more fractured assembly than long-read sequences. For example, the *V. vinifera* ‘Sultanina’ genome, Illumina sequences were used to construct a haploidified assembly^29^ and subsequently an improved assembly with higher fidelity to the genotypes heterozygous nature was developed using the PLATANUS short-read assembler^30^. With interest in developing an assembly that could be used for the development of molecular markers for *V. riparia*^3^ Illumina short-read paired-end sequencing and mate paired long insert libraries were employed in the present study to assemble the heterozygous, diploid *V. riparia* ‘Manitoba 37’ genome.

This draft genome sequence, assembled using high quality Illumina reads (>369X coverage), provides a valuable resource for marker development and breeding efforts using wild germplasm. The assembly of 495 Mb in 69,616 scaffolds, has an N50 of 518 kb which is greater than the N50 value reported for the *V. vinifera* ‘Sultanina’ genome assembled using Illumina data^30^. The closest *Vitis* representative to our draft genome is *V. riparia* ‘Riparia Gloire de Montpellier’, a widely used rootstock variety. Interestingly, the long-read genome assembly of the ‘Gloire’ variety reports 33.9% repetitive sequences, far less than the 46% repeat sequences we detected in ‘Manitoba 37’ (which is similar *V. vinifera* ‘PN40024’ (41.4%))^24,25^. Several *Vitis* genome assemblies have been developed recently using Illumina HiSeq, PacBio RSII or 10X Chromium Genomics^24,26–28^. The alignment results of *V. riparia* ‘Manitoba 37’ with primary contigs of *V. vinifera* cultivars was greater than 74% and as expected, the greatest alignment occurred with *V. riparia* ‘Riparia Gloire de Montpellier’ PACBio assembly^24^. Further evidence of quality can be seen in the similarity of *V. riparia* and *V. vinifera* genes identified in a common orthologous core across multiple genus, which indicate not only gene preservation across genetic distance, but provide evidence of assembly quality. In addition, assembly quality was indicated by the strong relationship found between the predicted *V. riparia* and *V. vinifera* genes. Indeed, the strong similarity of *LBD* and *WRKY* transcription factor families, between *V. riparia* and *V. vinifera* provide further evidence of assembly quality^31,32^. The validation, completeness and accuracy of *V. riparia* ‘Manitoba 37’ genome features indicate that using only short-read Illumina sequences a high quality *V. riparia* genome assembly was developed.

*V. riparia* is typically used in breeding programs to incorporate abiotic stress tolerance traits into new hybrid cultivars. Thus, particular attention was paid to examining the *WRKY*, *MYB*, and *ERF* transcription factors that influence gene regulation and have a strong role in abiotic stress tolerance phenotypes^32–34^. The *V. riparia* predicted *WRKY* coding sequences were consistent with the published *V. vinifera* ‘PN40024’ 12X.1 *WRKY* groups^32^ and the V3 annotation of 12X.2^25^, showing a great level of conservation between the species. The *MYB* family of transcription factor genes was explored specifically because of their importance to color, flavor and chemistry in grapevine species. Phylogenetic reconstruction of *MYB* subgroups found that the subgroups (4, 13, and 24) that had potential paralogous duplications were involved in ABA response, flavanol synthesis, secondary growth and anther development^35,36^. The only homologues with deletions in *V. riparia* ‘Manitoba 37’ was *MYB*113 of subgroup 6 which has been shown to regulate the production of anthocyanins in a *BASIS HELIX-LOOP-HELEX* (*bHLH*) dependent manner^36^. We also noted an increased number of *bHLH* genes predicted in *V. riparia* ‘Manitoba 37’ genome assembly (by > 20 genes) which is known to produce high amounts of predominantly diglucoside derivatives as opposed to monoglucosides in *V. vinifera*^37,38^. This result adds genomic context to one of the key issues facing acceptance of hybrid derived grape products as the presence of diglucoside derivatives is considered an indication of low quality in hybrid wines^39^. Analysis of *ERF* genes using alignment and motif comparison between *V. riparia* and *V. vinifera* found functional similarities between members of each subfamily from both genomes. However, there were many instances of duplications present in *V. vinifera* ‘PN40024’ that were not present in our assembly, such as in subfamily IX where some duplications presented with different motifs.

Retrotransposon activity has long been associated with diversification of species clades. We observed a lower number of genes associated with transposases and retrotransposons in the *V. riparia* ‘Manitoba 37’ and *V. vinifera ‘*Sultanina’ than in the *V. vinifera* 12X.2. All three species seemed to share common ancestors for each LTR gene but experienced paralogous gene duplication at different rates in each clade. We posit that this change in retrotransposons could have played some impact on the divergent evolution of the species, as it has been found previously that Tvv1 transposon markers could accurately distinguish between North American species and *V. vinifera* cultivars^40^.

The SNP markers that were developed using GBS of the F2 population in comparison with *V. vinifera ‘*PN40024’ 12X.1 allowed further analysis of the *V. riparia* ‘Manitoba 37’ assembly. Aligning these SNP markers with the pseudochromosomes of the grandparent, *V. riparia* ‘Manitoba 37’ and the *V. vinifera* ‘PN40024’ 12X.2 chromosomes showed that the F2 population more closely modeled *V. riparia* ‘Manitoba 37’. This can be expected since informative SNPs were predicted using the F2 grandparents and the male parent ‘Seyval’ has a complex pedigree including *V. vinifera* and other species. The presence of markers that aligned to chromosome 20 of *V. vinifera*, un-assembled scaffolds, but to other chromosomes on *V. riparia* may give us a better indication of the actual genomic position of those scaffolds on *V. vinifera ‘*PN40024’ assembly. By using the markers that mapped to different chromosomes in female grandparent and *V. vinifera* as a representative portion of the male grandparent, we found evidence for potential large genomic alterations between these species that may have occurred during the evolution and geographic isolation 3.5–9.5 million years ago. When we look at areas of both genomes containing QTL’s we can see that large translocations between chromosomes shows missing genes found between flanking markers in *V. riparia* relative to *V. vinifera*, thus impacting the resulting observed phenotype. This shows the potential power of sequencing and assembling a genetic grandparent of a F2 population in identifying the genetic basis of QTL regions.

In conclusion, we present high coverage short-read draft genome sequence of the wild grapevine species *V. riparia*. This genome represents the second genome assembly of this critically important species and the first representative of a locally adapted stress tolerant genotype. The *V. riparia* ‘Manitoba 37’ genome assembly provides an important resource for comparative genomic and genetic marker studies. This *V. riparia* ‘Manitoba 37’ genome has already proven useful for the development of molecular markers in North American breeding programs and will serve as an important tool in the development of genomics-assisted selection for grapevine improvement, particularly for traits associated with abiotic and biotic stress resistance.

## Materials and methods

### *V. riparia* ‘Manitoba 37’ materials

*V. riparia* ‘Manitoba 37’ (identified as ‘PI588259’ in USDA Germplasm Repository, Geneva, NY, USA) was used for sequencing. The genetic relationship of *V. riparia* ‘Manitoba 37’ to other *V. riparia* genotypes was analyzed using a data set extracted from genotype data collected from multiple species housed at the Geneva USDA-ARS grape germplasm repository^10^. To identify highly specific SNPs, VCFtools filters were applied to 156,799 SNPs from 74 unique *V. riparia* genotypes, keeping those found in at least 50% of the individuals, resulting in 54,029 SNPs^41^. We then removed six *V. riparia* genotypes with missing data at greater than 30% of the total SNPs. A high stringency filter was applied to the remaining SNPs keeping all SNPs found in 95% or greater of the *V. riparia* providing 1485 highly specific SNPs. SNPRelate R package^42^ was used to calculate the principal components of the specific SNPs data set and plotted the 68 individuals using ggplot2^43^.

### DNA sequencing and pre-processing of reads

One centimeter diameter new leaves of greenhouse grown vines were used for DNA extraction and sequencing. A total of nine paired-end libraries were constructed with insert sizes of 346, 473, 478 by Illumina I and 250, 450, 600, 3–5 kb, 8–10 kb, 15–20 kb by Illumina Hiseq 2500 sequencer. In total, 2295.4 M raw reads were generated with 658.4X coverage and read length from 100 nt–260 nt (Supplementary Table 10a). Raw reads were then filtered and corrected using cutadapt, Trimmomatic, PEAR, FastUniq, Quake, and NGSTOOLKIT (Tool references are found in Supplementary Table 10b). The k-mer analysis was carried out with Jellyfish with 19 bp k-mers using only 262.3X coverage of filtered reads. The genome’s heterozygosity and other results were obtained with GenomeScope. All filtered reads used for de novo genome assembly were mapped back to our assembly using bowtie2. The SAM files of the bowtie2 mapping results were converted to BAM files using SAMtools, and then the alignment statistics were obtained using the flagstat option of SAMtools (Supplementary Table 10b).

### *V. riparia* ‘Manitoba 37’ de novo heterozygous genome assembly and assembly evaluation

A total of 1313.7 M filtered reads were used for de novo genome assembly. The first assembly was obtained with PLATANUS^44^ by changing parameters in each of the three steps ((assemble: -u 0.2 -d 0.3), (scaffold: -s 20 -v 20 -u 0.2) and (gap close: -s 20 -vo 20 -vd 20 -ed 0.1)).This assembly was processed with GapCloser and the result of this assembly was then subjected to a second round of GapCloser to produce the final assembly (https://openprairie.sdstate.edu/vitis_riparia_VR37_PI588259) (All tool references are found in Supplementary Table 10b). The assembly was tested for contamination using DeconSeq. The assembly statistics were evaluated using an Assemblathon script and processed with reference genome *V. vinifera* ‘PN40024’ assembly^25^ 486,205,130 bp. The quality of the assembly was further assessed by four independent methods. (1) The percentage filtered reads were mapped back to the *V. riparia* ‘Manitoba 37’ genome using a zero mismatch. (2) The quality of the assembly was further assessed by using REAPR program which measures the number of times that there is low mapped mate-paired-end read coverage of any specific site to predict potential errors in contig assembly. While we found few mistakes in assembly of reads (only 16% of contigs had errors), we did observe low incidence of error free bases (maximum 42.11%). The reason for this low rate is unknown, however, REAPR is a relatively new tool in plant genome development and may not be properly calibrated for the high heterozygosity of this genome. (3) The *V. riparia* ‘Manitoba 37’ assembly quality was further characterized by generating a dot plot of *V. vinifera* ‘PN40024’ and *V. riparia* assembly using the D-genie program which plotted a sorted and denoised global alignment of the two assemblies. 4) *V. riparia* ESTs and BAC sequences and three de novo *V. riparia* transcriptomes were aligned with the genome. GMAP with default parameters was used to map and EST sequences of *V. riparia* from NCBI to our assembly (SAMN00174930, SAMN00152554, SAMN00152555, SAMN00152556, and SAMN00150676). Three *V. riparia* de novo transcriptomes (PI588259, PI588271, and PI588587), were aligned with the *V. riparia* genome assembly using BLAT. We used the MUMmer package for alignment of *V. riparia* BAC sequences (BioProject PRJNA550997) and *V. vinifera ‘*PN40024’^25^. (1) BACs were aligned to each *V. riparia* scaffold using nucmer with -mum option. (2) The output results from nucmer were filtered using delta-filter with the -g option. (3) The filtered results were used in the show-coords program and the coordinates of the resulting alignments were obtained. (4) The alignments that represented the longest length (top-hit) for each BAC were summed (top-hits-length). The same steps were followed for mapping the de novo *V. riparia* ‘Manitoba 37’ assembly with the reference genome *V. vinifera* ‘PN40024’ (12X.2, V3), *V. vinifera* cv. Cabernet Sauvignon, *V. vinifera* ‘Chardonnay’, *V. vinifera* ‘Carménère’, and *V. riparia* ‘Riparia Gloire de Montpellier’.

### *V. riparia* SNP calling

All filtered reads were aligned to the reference genome *V. vinifera* ‘PN40024’^25^ with bowtie2. The SAM files were converted to BAM files then repeats were removed by rmdup, followed by sorting in bowtie2. We called the SNPs using the mpileup of SAMtools with default parameters. Then, SNPs were filtered by VCFtools using a window of 10, a minimum depth of 8, and a minimum quality 40. SNP effect was predicted by using the SnpEff program (Supplementary Table 10c).

### De novo gene prediction and functional annotation

The repeats were identified with RepeatModeler and then repeats were masked by RepeatMasker (All tool references used in gene prediction and annotation are shown in Supplementary Table 10d). The RNAseq data of *V. riparia* from our lab were mapped to the masked *V. riparia* genome assembly and all.bam files were used for de novo gene prediction with BRAKER-1. The assembly gene coverage was preliminarily assessed by BUSCO (version 4.0.5), after gene prediction, using the latest plant early release database (embryophyta_odb10) in genome mode. Coding sequences were further annotated using Blast2GO. BLASTX was performed using the nr database with parameters of: E value 1.0E−3; number of blast hits 1, word size 6, HSP length cutoff 33, and eukaryotes selected as taxonomy. The results from the BLASTX of the assembly was then searched for enzyme classification databases: InterPro, GO (gene ontology), and KEGG pathway analysis using Blast2GO.

### Whole genome synteny analysis of *V. riparia* and *V. vinifera*

The de novo *V. riparia* assembly was aligned to reference genome *V. vinifera* ‘PN40024’^25^. Masking of repeat sequences and gene predictions from this chromosome-level assembly were obtained as described above for the scaffold assembly. Syntenic blocks between the genomes of *V. riparia* and other *Vitis* genomes^24–28^ were computed by SyMAP (v4.2). *V. riparia* ‘Manitoba 37’ genome and gene models were aligned to all other genomes and gene models separately (*V. vinifera* ‘PN40024’^25^*, V. vinifera* ‘Sultanina’^30^*, Populus trichocarpa, Medicago truncatula*, and *Arabidopsis thaliana*^45^, and *Fragaria vesca*^46^, using the promer option of the MUMmer program (Supplementary Table 5b–d). Syntenic blocks between *V. riparia* and all other genomes were obtained from a script in SyMAP (v4.2). (All tool references are in Supplementary Table 10e).

### Analysis of orthologous genes

All the predicted protein sequences from *V. riparia*, *V. vinifera* ‘PN40024’*, V. vinifera* Sultanina*, Populus, Fragaria*, and *Arabidopsis* were analyzed using OrthoMCL with default settings (All tool references are in Supplementary Table 10e). OrthoMCL was performed step-by-step as described in the manual. (1) An all vs. all BLASTP was performed to identify best hit pairs between species (orthologs), as well as sets of genes more closely related within than between species (in-paralogs). (2) This best hit matrix was used for ortholog definition with the parameter (*I*) = 1.5. 3). The results from OrthoMCL were visualized by OrthoVenn. The single copy gene present in all six species was used to generate a phylogenetic tree in the program MEGA7. (1) All protein sequences were aligned with ClustalW using PAM weight matrix. (2) The multiple sequence alignments were then used for phylogenetic tree construction by Maximum Likelihood method using default parameters in MEGA7.

### Plant transcription factors prediction and phylogenetic tree of gene families

Using all predicted protein sequences from *V. riparia* ‘Manitoba 37’ assembly and *V. vinifera* ‘PN40024’ (12X.1, V2 and 12X.2, V3) annotation^25^ we predicted Plant transcription factors with PlantTFDB (4.0)^35^. The *V. vinifera* ‘PN40024’ 12X.1, V2^47^ annotation was also used so that comparisons could be made with earlier transcription factor characterization publications. Transcription factors were predicted together for all assemblies so that comparisons could be made directly in this study. The *WRKY* gene family results and protein sequences of *V. riparia* ‘Manitoba 37’ and *V. vinifera* ‘PN40024’. 12X.1, V1; 12X.2, V3 were retrieved from PlantTFDB (4.0)^35^ and classified into different groups based on a previous *WRKY* study^32^. We created a circular phylogenetic tree for *WRKY* in two steps, (1) *WRKY* protein sequences of *V. riparia* and *V. vinifera* ‘PN40024’ aligned together by ClustalW in MEGA7 (Supplementary Table 10b). The circular phylogenetic tree was then constructed by the neighbor-joining method using the complete deletion option and bootstrapping with 1000 replicates. Annotation of subgroups in *MYB* and *ERF* TFs was done through a BLAST alignment to classified *Arabidopsis* TFs in PlantTFDB^48,49^. The neighbor-joining tree for the *MYB* and *ERF* TFs was generated from a clustal alignment of all genes through R code ggtree and msa packages (Supplementary Table 10f).

### Alignment of F2 GBS markers to *V. riparia* ‘Manitoba 37’ and *V. vinifera* ‘PN40024’ 12X.2

The predicted *V. riparia* ‘Manitoba 37’ gene set and *V. vinifera* ‘PN40024’ annotation were aligned to the PFAM database using HMMer (All tool references for QTL alignment are in Supplementary Table 10g). The matching annotations were used to divide genes into families or domains. Then the differences in gene number for each gene family at each position of the corresponding gene on the *V. vinifera* chromosome were plotted with RCircos. The GBS genetic markers from a F2 mapping population, derived from a self of an individual F1 genotype from a cross of *V. riparia* ‘Manitoba 37’ and hybrid cultivar ‘Seyval’^2^, were then aligned to *V. vinifera* ‘PN40024’ 12X.2 and a pseudo-chromosomal assembly of *V. riparia* ‘Manitoba 37’. The *V. riparia* pseudo-chromosomal assembly was based on its genomic alignment to *V. vinifera*^35^, using the Bowtie aligner (Supplementary Table 10c). R programming was used to find the common marker set and plot the markers mapping to chromosomes using RCircos (Supplementary Table 10g). The gene containing regions of *V. vinifera* 12X.2 and *V. riparia* ‘Manitoba 37’ were extracted from between QTL markers for previously predicted QTL flanking markers for female sex and summer lateral cessation in response to decreasing photoperiod phenotype on chromosome 2 and scaled to markers shared between the species^2,18,19^. The LOD score of the species-specific markers were then plotted using scaled LOD values to present species protein domain distribution.

## Supplementary information

Supplementary Fig. 1

Supplementary Fig. 2

Supplementary Fig. 3

Supplementary Fig. 4

Supplementary Fig. 5

Supplementary Table 1

Supplementary Table 2

Supplementary Table 3

Supplementary Table 4

Supplementary Table 5

Supplementary Table 6

Supplementary Table 7

Supplementary Table 8

Supplementary Table 9

Supplementary Table 10

## Data Availability

The raw reads of Illumina data and BAC sequences are found in the NCBI BioProject PRJNA550997. EST sequences were downloaded from existing NCBI nucleotide resources. The genome assembly, gene annotation, proteins, and other data are publically available at: https://openprairie.sdstate.edu/vitis_riparia_VR37_PI588259.
