# Supplementary figures and images for: Draft genome of the Native American cold hardy grapevine Vitis riparia Michx. ‘Manitoba 37’
Source: Hortic Res. 2020 Jun 1;7:92. doi: 10.1038/s41438-020-0316-2 (PMC7261805; doi:10.1038/s41438-020-0316-2)

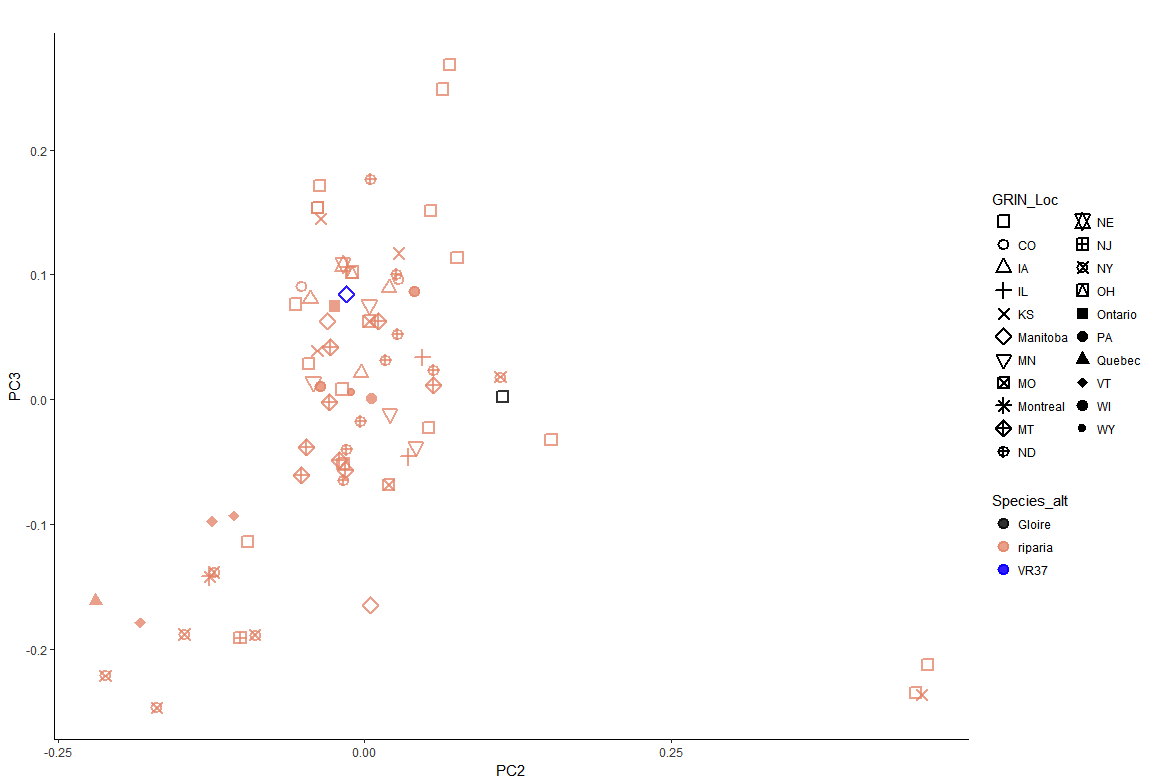

Supplement: Supplementary file 1 — Supplementary Fig. 1 [file 41438_2020_316_MOESM1_ESM.jpg]

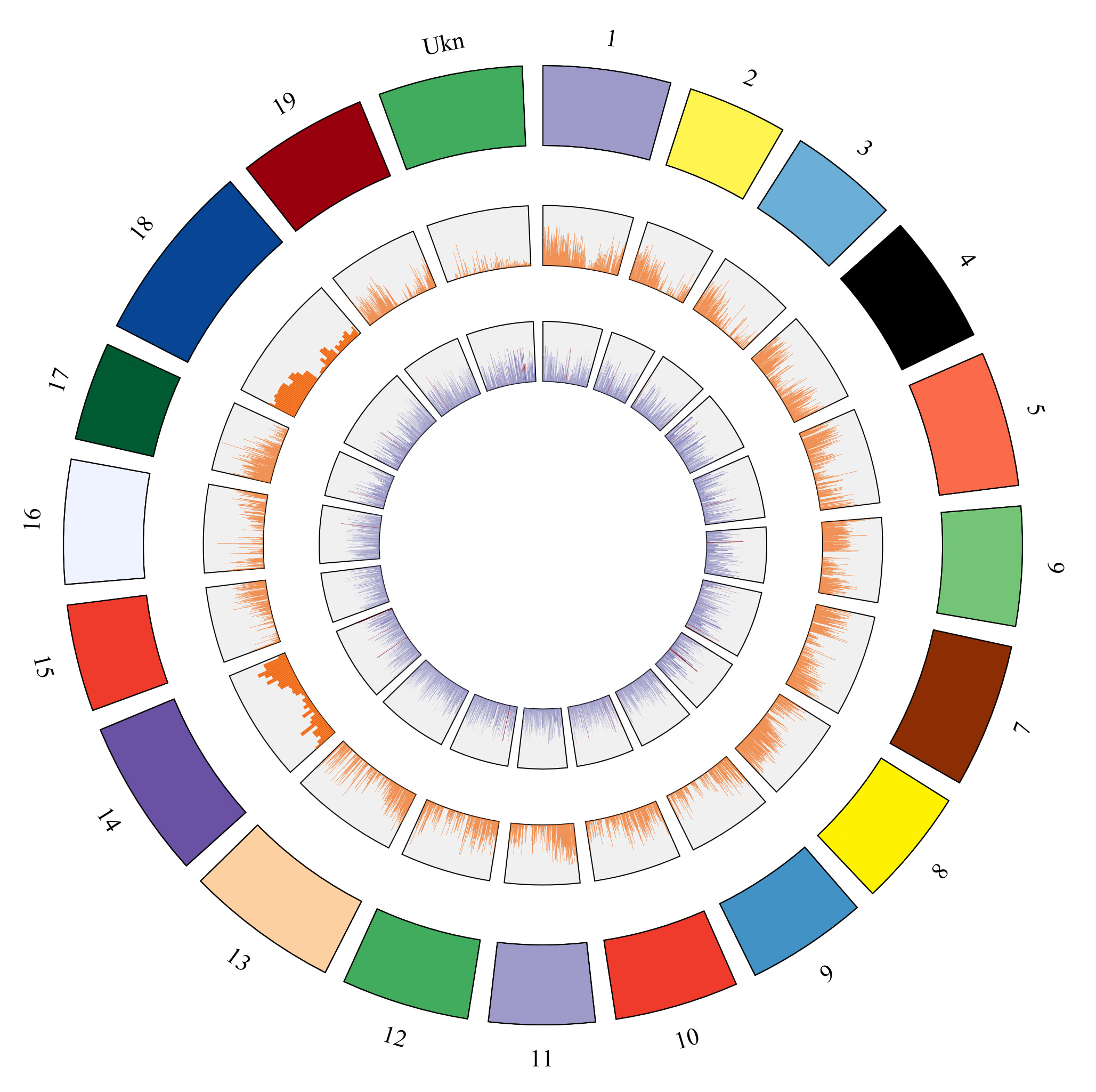

Supplement: Supplementary file 2 — Supplementary Fig. 2 [file 41438_2020_316_MOESM2_ESM.jpg]

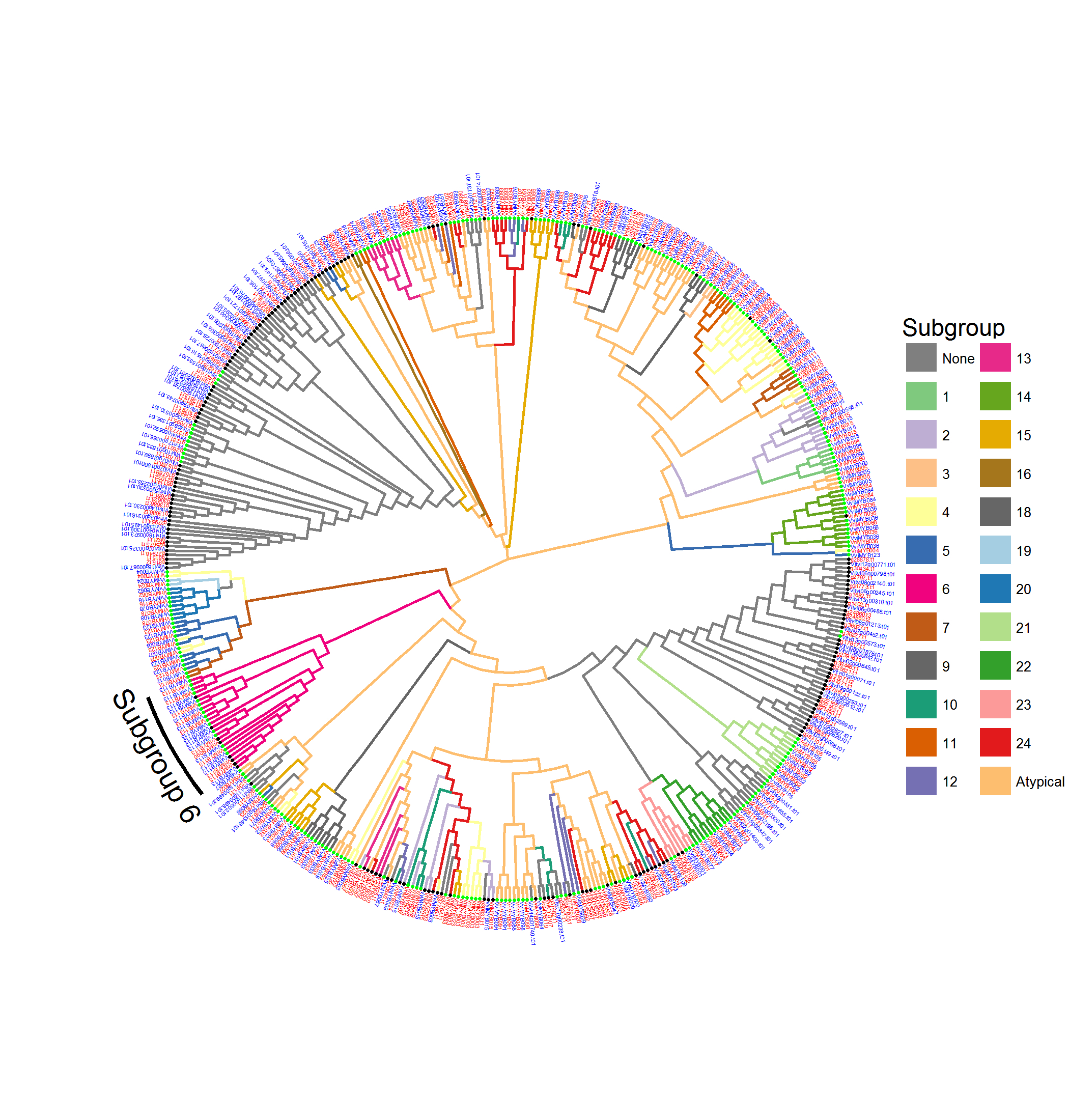

Supplement: Supplementary file 3 — Supplementary Fig. 3 [file 41438_2020_316_MOESM3_ESM.jpg]

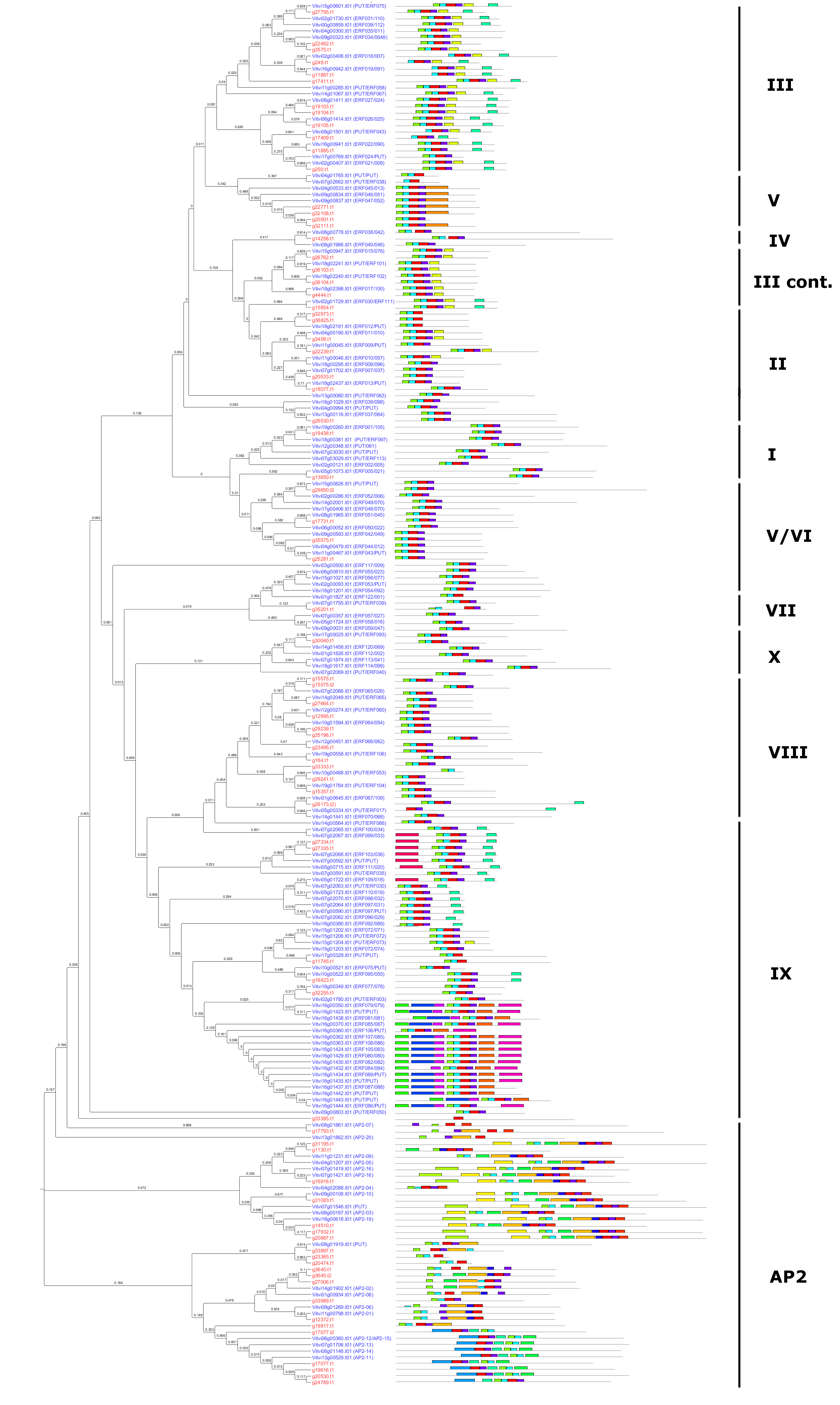

Supplement: Supplementary file 4 — Supplementary Fig. 4 [file 41438_2020_316_MOESM4_ESM.jpg]

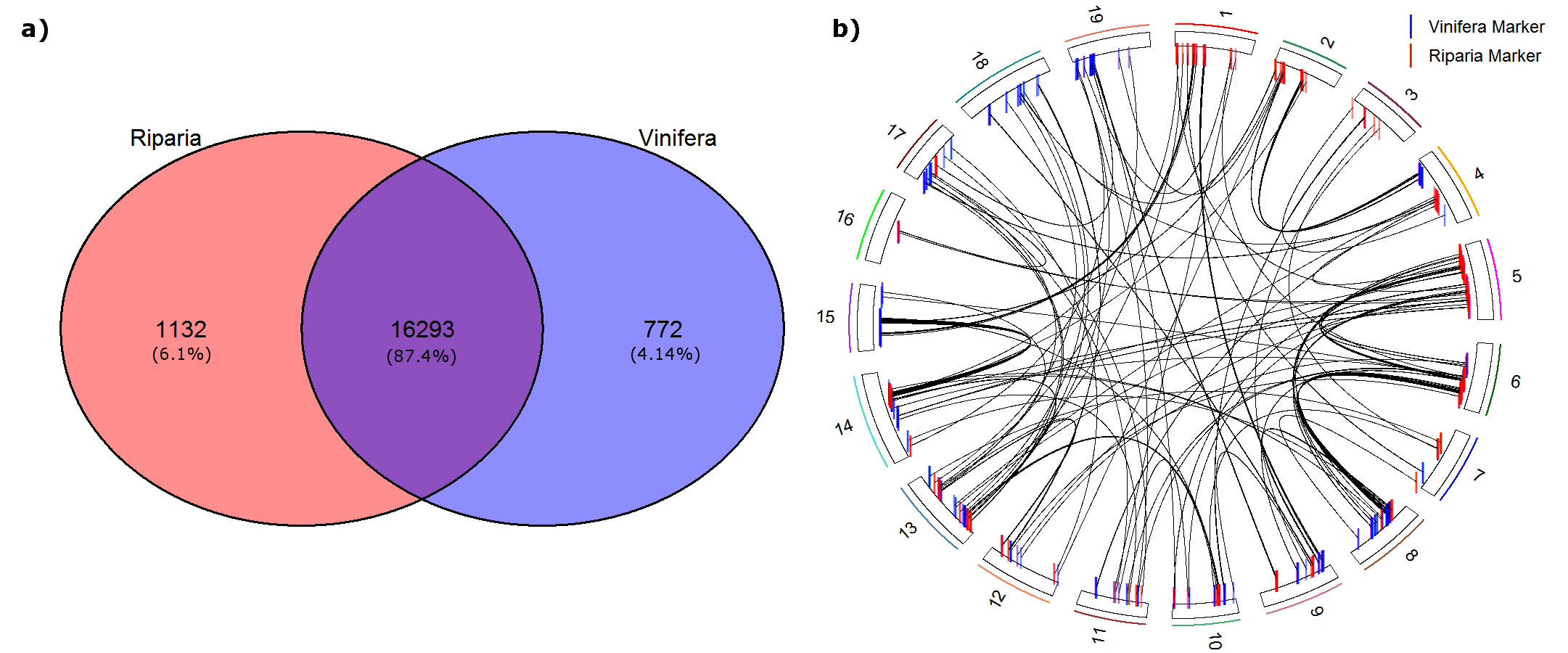

Supplement: Supplementary file 5 — Supplementary Fig. 5 [file 41438_2020_316_MOESM5_ESM.jpg]
