# Supplementary material for: Draft genome of the Native American cold hardy grapevine Vitis riparia Michx. ‘Manitoba 37’
Source: Hortic Res. 2020 Jun 1;7:92. doi: 10.1038/s41438-020-0316-2 (PMC7261805; doi:10.1038/s41438-020-0316-2)
Supplement: Supplementary file 6 — Supplementary Table 1 [file 41438_2020_316_MOESM6_ESM.docx]

| **Supplementary Table 1. *V. riparia VR37/PI588259 de novo* genome complete assembly statistics from Assemblathon.** | | | | | |
| --- | --- | --- | --- | --- | --- |
| **Details** |  |  |  |  |  |
| **Step 1** |  | **Step1: -u 0.2 -d 0.3** | **Assembly 1+ GapCloser using all filtered reads** | **Assembly 2+ GapCloser using all filtered reads** | **Final assembly after using DeconSeq** |
| **Step 2** |  | **Step2: -s 20 -v 20 -u 0.2** |  |  |  |
| **Step 3** |  | **Step 3: -s 20 -vo 20 -vd 20 -ed 0.1** |  |  |  |
| **Methods** |  | **Assembly- 1 (Platanus)** | **Assembly-2 (Platanus+GapCloser)** | **Assembly-3 (Platanus+Gapcloser+Gapcloser)** | **Final assembly** |
|  |  |  |  |  |  |
| Number of scaffolds |  | 69,629 | 69,618 | 69,618 | 69,616 |
| Total size of scaffolds |  | 490,253,995 | 494,164,024 | 494,684,997 | 494,682,949 |
| Total scaffold length as percentage of assumed genome size |  | 100.80% | 101.60% | 101.70% | 101.70% |
| Useful amount of scaffold sequences |  | 403,895,639 | 407,817,724 | 408,323,893 | 408,323,893 |
| (>= 25K nt) |  |  |  |  |  |
| % of estimated genome that is useful |  | 83.10% | 83.90% | 84.00% | 84.00% |
| Longest scaffold |  | 5,067,821 | 5,117,904 | 5,123,774 | 5,123,774 |
| Shortest scaffold |  | 500 | 500 | 500 | 500 |
| Number of scaffolds > 1K nt |  | 31,424 (45.1%) | 31,418 (45.1%) | 31,419 (45.1%) | 31,418 (45.1%) |
| Number of scaffolds > 10K nt |  | 1,758 (2.5%) | 1,759 (2.5%) | 1,760 (2.5%) | 1,760 (2.5%) |
| Number of scaffolds > 100K nt |  | 736 (1.1%) | 741 (1.1%) | 742 (1.1%) | 742 (1.1%) |
| Number of scaffolds > 1M nt |  | 94 (0.1%) | 97 (0.1%) | 97 (0.1%) | 97 (0.1%) |
| Number of scaffolds > 10M nt |  | 0 | 0 | 0 | 0 |
| Mean scaffold size |  | 7,041 | 7,098 | 7,106 | 7,106 |
| Median scaffold size |  | 929 | 929 | 929 | 929 |
| N50 scaffold length |  | 512,151 | 516,886 | 518,740 | 518,740 |
| L50 scaffold count |  | 213 | 213 | 212 | 212 |
| NG50 scaffold length |  | 515,528 | 533,810 | 535,518 | 535,518 |
| LG50 scaffold count |  | 209 | 205 | 204 | 204 |
| N50 scaffold - NG50 scaffold length difference |  | 3,377 | 16,924 | 16,778 | 16,778 |
| scaffold %A |  | 27.82 | 31.12 | 31.38 | 31.38 |
| scaffold %C |  | 15.43 | 16.77 | 16.89 | 16.89 |
| scaffold %G |  | 15.42 | 16.77 | 16.89 | 16.89 |
| scaffold %T |  | 27.7 | 31.01 | 31.27 | 31.27 |
| scaffold %N |  | 13.63 | 4.33 | 3.57 | 3.57 |
| scaffold %non-ACGTN |  | 0 | 0 | 0 | 0 |
| Number of scaffold non-ACGTN nt |  | 0 | 0 | 0 | 0 |
| Percentage of assembly in scaffolded contigs |  | 85.60% | 82.50% | 81.80% | 81.80% |
| Percentage of assembly in unscaffolded contigs |  | 14.40% | 17.50% | 18.20% | 18.20% |
| Average number of contigs per scaffold |  | 1.7 | 1.1 | 1.1 | 1.1 |
| Average length of break (>25 Ns) between contigs in scaffold |  | 1,359 | 2,091 | 2,167 | 2,167 |
| Number of contigs |  | 118,739 | 79,822 | 77,750 | 77,748 |
| Number of contigs in scaffolds |  | 56,288 | 12,029 | 9,792 | 9,792 |
| Number of contigs not in scaffolds |  | 62,451 | 67,793 | 67,958 | 67,956 |
| Total size of contigs |  | 423,466,205 | 472,819,441 | 477,057,753 | 477,055,705 |
| Longest contig |  | 149,686 | 548,923 | 549,140 | 549,140 |
| Shortest contig |  | 0 | 52 | 52 | 52 |
| Number of contigs > 1K nt |  | 70039 (59.0%) | 41,206 (51.6%) | 39231 (50.5%) | 39,230 (50.5%) |
| Number of contigs > 10K nt |  | 10734 (9.0%) | 8,224 (10.3%) | 7269 (9.3%) | 7,269 (9.3%) |
| Number of contigs > 100K nt |  | 12 (0.0%) | 811 (1.0%) | 974 (1.3%) | 974 (1.3%) |
| Number of contigs > 1M nt |  | 0 | 0 | 0 | 0 |
| Number of contigs > 10M nt |  | 0 | 0 | 0 | 0 |
| Mean contig size |  | 3,566 | 5,923 | 6,136 | 6,136 |
| Median contig size |  | 1,259 | 1,029 | 1,008 | 1,008 |
| N50 contig length |  | 9,805 | 48,653 | 61,142 | 61,142 |
| L50 contig count |  | 11,045 | 2,402 | 1,959 | 1,959 |
| NG50 contig length |  | 7,680 | 46,447 | 59,045 | 59,045 |
| LG50 contig count |  | 14,655 | 2,543 | 2,035 | 2,035 |
| N50 contig - NG50 contig length difference |  | 2,125 | 2,206 | 2,097 | 2,097 |
| contig %A |  | 32.2 | 32.53 | 32.54 | 32.54 |
| contig %C |  | 17.86 | 17.53 | 17.52 | 17.52 |
| contig %G |  | 17.85 | 17.53 | 17.51 | 17.51 |
| contig %T |  | 32.07 | 32.41 | 32.42 | 32.43 |
| contig %N |  | 0.01 | 0.01 | 0.01 | 0.01 |
| contig %non-ACGTN |  | 0 | 0 | 0 | 0 |
| Number of contig non-ACGTN nt |  | 0 | 0 | 0 | 0 |
