# Supplementary material for: Draft genome of the Native American cold hardy grapevine Vitis riparia Michx. ‘Manitoba 37’
Source: Hortic Res. 2020 Jun 1;7:92. doi: 10.1038/s41438-020-0316-2 (PMC7261805; doi:10.1038/s41438-020-0316-2)
Supplement: Supplementary file 7 — Supplementary Table 2 [file 41438_2020_316_MOESM7_ESM.docx]

**Supplemental Table 2a. Results from REAPR analysis using three mate-pair libraries to find errors in the *V. riparia* ‘Manitoba 37’ genome assembly.**

| Insert Library | Error Free Bases (bp) | Predicted Error |
| --- | --- | --- |
| 15-20 kb | 186,448,672 (37.69%) | 11,534 |
| 8-10 kb | 208,300,227 (42.11%) | 4,345 |
| 3-5 kb | 184,472,951 (37.29%) | 7,900 |

**Supplemental Table 2b. BAC, EST , three *de novo* transcriptome assemblies of *V. riparia* and CDS of *V. vinifera* PN40024, 12X.2, V3 alignment with *V. riparia*  ‘Manitoba 37’ assembly.**

| Details |  | Aligned to *V. riparia* assembly |
| --- | --- | --- |
| BAC sequences: 4,171 | Top hits* of BAC sequences | 3,811 |
| BAC bp: 2,671,662 | Top hits* BAC length (bp) | 2,400,641 |
|  | BAC sequences with >90% identity and  >90% coverage | 3,398 |
|  | BAC lengths with >90% identity and  >90% coverage (bp) | 2,205,459 |
|  |  |  |
| EST sequences: 1,974 | Top hits* of EST sequences | 1,935 |
| EST bp: 1,120,213 | Top hits* EST length (bp) | 1,085,372 |
|  | EST sequences with >90% identity and  >70% coverage | 1,880 |
|  | EST lengths with >90% identity and  >70% coverage (bp) | 1,066,031 |
|  |  |  |
| *De novo* transcriptome assemblies |  |  |
| (i) PI588259VVout.fasta | Top hits* of transcript sequences | 121,957 |
| Transcripts: 122,183 | Top hits* transcript length (bp) | 105,671,217 |
| Transcripts bp:   108,694,739 | Transcript sequence with >90% identity and  >90% coverage | 114,198 |
|  | Transcript lengths with >90% identity and  >90% coverage (bp) | 98,837,259 |
|  |  |  |
| (ii) PI588271VVout.fasta | Top hits* of transcript sequences | 59,758 |
| Transcripts: 59,972 | Top hits* transcript length (bp) | 51,762,958 |
| Transcripts bp:   53,308,863 | Transcript sequence with >90% identity and  >90% coverage | 55,956 |
|  | Transcript lengths with >90% identity and  >90% coverage (bp) | 48,691,733 |
|  |  |  |
| (iii) PI588587VVout.fasta | Top hits* of transcript sequences | 76,358 |
| Transcripts: 76,613 | Top hits* transcript length (bp) | 66,164,784 |
| Transcript bp:   68,230,349 | Transcript sequence with >90% identity and  >90% coverage | 71,184 |
|  | Transcript lengths with >90% identity and  >90% coverage (bp) | 61,939,988 |
|  |  |  |
| *V. vinifera* V3 CDS |  |  |
| CDS sequences: 41,733 | Top hits* of CDS sequences | 41,189 |
| CDS bp: 41,162,907 | Top hits* CDS length (bp) | 37,526,954 |
|  | CDS sequences with >90% identity and  >70% coverage | 33,370 |
|  | CDS lengths with >90% identity and  >70% coverage (bp) | 34,300,899 |

*Top hits represent the longest alignment for each BAC, EST, transcript of *V. riparia* and CDS of *V. vinifera* PN40024, 12X.2, V3 with *V. riparia* ‘Manitoba 37’ assembly and the total of those alignment lengths (Top hits lengths) was calculated.
