# Supplementary material for: Draft genome of the Native American cold hardy grapevine Vitis riparia Michx. ‘Manitoba 37’
Source: Hortic Res. 2020 Jun 1;7:92. doi: 10.1038/s41438-020-0316-2 (PMC7261805; doi:10.1038/s41438-020-0316-2)
Supplement: Supplementary file 8 — Supplementary Table 3 [file 41438_2020_316_MOESM8_ESM.docx]

**Supplementary File 3. Alignment of *V. riparia* ‘Manitoba 37’ assembly (494,682,449 bp, 69,616 scaffolds) to reference genome of *V. vinifera* PN40024, 12X.2 and recent *Vitis* PacBio assemblies.**

| *Vitis* genome | Top Hit* | > 90% Identity | > 95% Identity and > 1000 bp alignment** | > 99% Identity and  > 1000 bp alignment** |
| --- | --- | --- | --- | --- |
| *V. vinifera* (12X.2, V2) | 59,373 scaffolds  77,270,636 bp | 52,923 scaffolds  71,073,607 bp | 9,496 scaffolds  29,917,598 bp | 178  scaffolds 293,798 bp |
|  |  |  |  |  |
| *V. vinifera* cv. Cabernet Sauvignon  p-contigs: 718 sequences 591,420,921 bp | 58,582 scaffolds  78,379,487 bp | 52,211 scaffolds  72,079,677 bp | - | - |
| haplotigs: 2,037 sequences 367,781,855 bp | 50,319 scaffolds  65,139,815 bp | 43,519 scaffolds  58,152,473 bp | - | - |
|  |  |  |  |  |
| *V. vinifera* ‘Chardonnay’  p-contigs:  731 sequences 464,195,627 bp | 58,568 scaffolds 77,106,622 bp | 51,527 scaffolds 70,065,247 bp | - | - |
| haplotigs:  2,006 sequences 403,846,922 bp | 54,792 scaffolds 71,445,189 bp | 47,557 scaffolds 64,210,173 bp | - | - |
|  |  |  |  |  |
| *V. vinifera* ‘Carménère’  p-contigs:  1,411 sequences 622,795,289 bp | 59,601 scaffolds 80,447,407 bp | 53,284 scaffolds 74,280,227 bp | - | - |
| haplotigs:  7,969 sequences 420,345,460 bp | 48,262  scaffolds 62,151,745 bp | 41,732 scaffolds 55,555,956 bp | - | - |
|  |  |  |  |  |
| *V. riparia*  ‘Riparia Gloire  de Montpellier’ | 65,828 scaffolds 109,545,646 bp | 63,096 scaffolds 106,863,074 bp | - | - |

* Top Hit represents the longest alignment for each *V. riparia* ‘Manitoba 37’ scaffold with all *Vitis* species separately and the total of those alignment lengths (top-hit) was calculated.

** Indicates identification of red bars in Supplementary Fig. 2.
