# Supplementary material for: Draft genome of the Native American cold hardy grapevine Vitis riparia Michx. ‘Manitoba 37’
Source: Hortic Res. 2020 Jun 1;7:92. doi: 10.1038/s41438-020-0316-2 (PMC7261805; doi:10.1038/s41438-020-0316-2)
Supplement: Supplementary file 9 — Supplementary Table 4 [file 41438_2020_316_MOESM9_ESM.doc]

**Supplementary File 4 (a). Repetitive sequence information in our assembly by RepeatMasker.**

| **Number of elements*** | **Length occupied** | **Percentage of sequence** |
| --- | --- | --- |
| LINEs | 29,057 | 20,843,899 bp (4.21 %) |
| LTR elements | 113,141 | 87,435,832 bp (17.68 %) |
| DNA elements | 18,792 | 10,188,082 bp (2.06 %) |
| Unclassified | 308,816 | 98,701,664 bp (19.95 %) |
| Simple repeats | 153,890 | 7,656,807 bp (1.55 %) |
| Low complexity | 34,536 | 1,759,370 bp (0.36 %) |
| **Total** | **658,232** | **226,585,654 bp (45.81%)** |

* Most repeats fragmented by insertions or deletions have been counted as one element.

**Supplementary File 4 (b). BUSCO results.**

C:95.9%[S:94.4%,D:1.5%],F:1.8%,M:2.3%,n:1614

| 1,548 | Complete BUSCOs (C) |
| --- | --- |
| 1,523 | Complete and single-copy BUSCOs (S) |
| 25 | Complete and duplicated BUSCOs (D) |
| 29 | Fragmented BUSCOs (F) |
| 37 | Missing BUSCOs (M) |
| 1614 | Total BUSCO groups searched |

*BUSCO version 4.0.5 with embryophyta_odb10 dataset

**Supplementary File 4 (c). Blastx results of species distribution.**

| **Species** | **#BLAST Hits** |
| --- | --- |
| Vitis vinifera | 35,342 |
| Juglans regia | 97 |
| Theobroma cacao | 85 |
| Nelumbo nucifera | 61 |
| Citrus sinensis | 57 |
| Populus trichocarpa | 55 |
| Jatropha curcas | 47 |
| Manihot esculenta | 47 |
| Gossypium raimondii | 47 |
| Ricinus communis | 46 |
| Daucus carota subsp. sativus | 45 |
| Morus notabilis | 45 |
| Eucalyptus grandis | 41 |
| Gossypium arboreum | 39 |
| Ziziphus jujuba | 35 |
| Vitis pseudoreticulata | 34 |
| Glycine max | 33 |
| Cephalotus follicularis | 32 |
| Citrus clementina | 32 |
| Prunus mume | 30 |
| Lupinus angustifolius | 27 |
| Vitis hybrid cultivar | 27 |
| Gossypium hirsutum | 26 |
| Populus euphratica | 26 |
| Vitis riparia | 24 |
| Malus domestica | 23 |
| Cajanus cajan | 23 |
| Prunus persica | 22 |
| Nicotiana attenuata | 21 |
| Trifolium subterraneum | 21 |
| Nicotiana tabacum | 21 |

| Phoenix dactylifera | 20 |
| --- | --- |
| Medicago truncatula | 18 |
| Sesamum indicum | 17 |
| Vitis amurensis | 17 |
| Erythranthe guttata | 16 |
| Beta vulgaris subsp. vulgaris | 16 |
| Fragaria vesca subsp. vesca | 15 |
| Pyrus x bretschneideri | 15 |
| Capsicum annuum | 15 |
| Brassica napus | 14 |
| Nicotiana sylvestris | 14 |
| Vitis quinquangularis | 14 |
| Glycine soja | 13 |
| Cynara cardunculus var. scolymus | 13 |
| Ipomoea nil | 13 |
| Cucumis sativus | 12 |
| Coffea canephora | 12 |
| Vitis aestivalis | 12 |
| Elaeis guineensis | 11 |
| Vitis labrusca | 11 |
| Vigna angularis | 11 |
| Arachis duranensis | 10 |
| Zea mays | 10 |
| Oryza sativa Japonica Group | 10 |
| Amborella trichopoda | 10 |
| Noccaea caerulescens | 10 |
| Genlisea aurea | 10 |
| Musa acuminata subsp. malaccensis | 10 |
| Camelina sativa | 9 |
| Vitis vinifera subsp. caucasica | 9 |
| Dorcoceras hygrometricum | 9 |
| Vitis labrusca x Vitis vinifera | 8 |
| Nicotiana tomentosiformis | 8 |

| Tarenaya hassleriana | 8 |
| --- | --- |
| Vitis cinerea var. helleri x Vitis riparia | 8 |
| Phaseolus vulgaris | 8 |
| Dichanthelium oligosanthes | 7 |
| Vicia faba | 7 |
| Setaria italica | 7 |
| Lotus japonicus | 7 |
| Vitis rotundifolia | 7 |
| Arabidopsis thaliana | 7 |
| Vitis yeshanensis | 6 |
| Picea glauca | 6 |
| Solanum tuberosum | 6 |
| Spinacia oleracea | 6 |
| Vigna angularis var. angularis | 6 |
| Vitis cinerea var. helleri x Vitis rupestris | 6 |
| Cicer arietinum | 6 |
| Arabis alpina | 5 |
| Brassica rapa | 5 |
| Vigna radiata var**.** radiata | 5 |
| Ananas comosus | 5 |
| Cucumis melo | 5 |
| Vitis rupestris | 5 |
| Anthurium amnicola | 5 |
| Solanum lycopersicum | 5 |
| Raphanus sativus | 4 |
| Arachis ipaensis | 4 |
| Eutrema salsugineum | 4 |
| Solanum pennellii | 4 |
| Arabidopsis lyrata subsp. lyrata | 3 |
| Lingula anatina | 3 |
| Populus tremula | 3 |
| Beta vulgaris subsp. maritima | 3 |
| Populus tomentosa | 3 |

| Sorghum bicolor | 3 |
| --- | --- |
| Oryza sativa Indica Group | 3 |
| Brassica oleracea var. oleracea | 3 |
| Brachypodium distachyon | 3 |
| Zostera marina | 2 |
| Batis maritima | 2 |
| Capsella rubella | 2 |
| Triticum turgidum subsp. durum | 2 |
| Beta macrocarpa | 2 |
| Triticum aestivum | 2 |
| Rhinolophus sinicus | 2 |
| Hevea brasiliensis | 2 |
| Pinus taeda | 2 |
| Cucumis melo subsp. melo | 2 |
| Pisum sativum | 2 |
| Prunus dulcis | 2 |
| Nematostella vectensis | 2 |
| Arachis diogoi | 2 |
| Picea sitchensis | 2 |
| Catharanthus roseus | 2 |
| Cercis chinensis | 1 |
| Loa loa | 1 |
| Populus tremula x Populus alba | 1 |
| Triticum timopheevii subsp. armeniacum | 1 |
| Aralia elata | 1 |
| Eptesicus fuscus | 1 |
| Turnera krapovickasii | 1 |
| Rhizophagus irregularis DAOM 197198w | 1 |
| Capitella teleta | 1 |
| Populus trichocarpa x Populus deltoides | 1 |
| Closterium ehrenbergii | 1 |
| Petunia x hybrida | 1 |
| Biomphalaria glabrata | 1 |

| Tetrastigma trifoliolatum | 1 |
| --- | --- |
| Lolium perenne | 1 |
| Diospyros kaki | 1 |
| Toxocara canis | 1 |
| Vitis cinerea | 1 |
| Puccinia striiformis f. sp. tritici PST-78 | 1 |
| Austrofundulus limnaeus | 1 |
| Cryptocoryne ciliata | 1 |
| Vitis labrusca x Vitis riparia | 1 |
| Sinocyclocheilus rhinocerous | 1 |
| Prunus persica var. persica | 1 |
| Miconia staminea | 1 |
| Senecio integerrimus | 1 |
| Triticum dicoccoides | 1 |
| Micromonas pusilla CCMP1545 | 1 |
| Tupaia chinensis | 1 |
| Croton texensis | 1 |
| Xylia xylocarpa var. kerrii | 1 |
| Rosa multiflora | 1 |
| Nekemias grossedentata | 1 |
| Beauveria bassiana ARSEF 2860 | 1 |
| Stentor coeruleus | 1 |
| Brassica juncea var. tumida | 1 |
| Geissoloma marginatum | 1 |
| Sinopodophyllum hexandrum | 1 |
| Vitis shuttleworthii | 1 |
| Eleocharis cellulosa | 1 |
| Mus musculus | 1 |
| uncultured eukaryote | 1 |
| Sticholonche zanclea | 1 |
| Ammi majus | 1 |
| Cryptocarya concinna | 1 |
| Vitis vinifera=grape berries, Sultana, berries, Peptide, 664 a | 1 |

| Salmo salar | 1 |
| --- | --- |
| Spodoptera frugiperda | 1 |
| Salvia miltiorrhiza | 1 |
| Saccoglossus kowalevskii | 1 |
| Cinnamomum micranthum f. kanehirae | 1 |
| Cocos nucifera | 1 |
| Citrullus lanatus | 1 |
| Panax ginseng | 1 |
| Couroupita guianensis | 1 |
| Butomus umbellatus | 1 |
| Branchiostoma belcheri | 1 |
| Auricularia subglabra TFB-10046 SS5 | 1 |
| Vitis sp. cv. 'Norton' | 1 |
| Stachybotrys chartarum IBT 40293 | 1 |
| Viola cornuta | 1 |
| Gentiana macrophylla | 1 |
| Cedrela fissilis | 1 |
| Triticum dicoccon | 1 |
| Triticum urartu | 1 |
| Vitis arizonica x Vitis rupestris | 1 |
| Aquilegia coerulea | 1 |
| Camellia sinensis | 1 |
| Phillyrea latifolia | 1 |
| Sesbania rostrata | 1 |
| Nicotiana benthamiana | 1 |
| Litchi chinensis | 1 |
| Camellia oleifera | 1 |
| Helianthus petiolaris | 1 |
| Pinus radiata | 1 |
| Cryptocarya depauperata | 1 |
| Rhododendron macrophyllum | 1 |
| Beilschmiedia percoriacea | 1 |
| Trachymyrmex septentrionalis | 1 |

| Bixa orellana | 1 |
| --- | --- |
| Oryza brachyantha | 1 |
| Hordeum vulgare subsp. vulgare | 1 |
| Wolffia arrhiza | 1 |
| Illicium oligandrum | 1 |
| Vitis bellula | 1 |
| Beilschmiedia robusta | 1 |
| Ficedula albicollis | 1 |
| Fragaria chinensis | 1 |
| Cryptocarya metcalfiana | 1 |
| Phytophthora parasitica | 1 |
| Linum usitatissimum | 1 |
| Trichinella patagoniensis | 1 |
| Vitis cinerea var. helleri x Vitis vinifera | 1 |
| Vigna radiata | 1 |
| Medicago sativa | 1 |
| GM37 | 1 |
| Chimonanthus praecox | 1 |
| Vitis thunbergii | 1 |
| Symphoricarpos albus | 1 |
| **Total** | **37,245** |

**Supplementary File 4 (d). Enzyme classification.**

| **EC Classes** | **#Seqs** |
| --- | --- |
| Oxidoreductases | 855 |
| Transferases | 1,698 |
| Hydrolases | 2,399 |
| Lyases | 238 |
| Isomerases | 214 |
| Ligases | 192 |

**Supplementary File 4 (e). GO distribution by level 2.**

| **Distribution type** | **GO-id** | **GO-term** | **#Seqs** |
| --- | --- | --- | --- |
| **Biological Process** | GO:0008152 | metabolic process | 12125 |
|  | GO:0009987 | cellular process | 10305 |
|  | GO:0044699 | single-organism process | 6952 |
|  | GO:0065007 | biological regulation | 3101 |
|  | GO:0050789 | regulation of biological process | 2691 |
|  | GO:0051179 | localization | 2451 |
|  | GO:0050896 | response to stimulus | 2277 |
|  | GO:0071840 | cellular component organization or biogenesis | 1615 |
|  | GO:0023052 | signaling | 836 |
|  | GO:0032501 | multicellular organismal process | 712 |
|  | GO:0032502 | developmental process | 707 |
|  | GO:0022414 | reproductive process | 441 |
|  | GO:0000003 | reproduction | 441 |
|  | GO:0048518 | positive regulation of biological process | 325 |
|  | GO:0048519 | negative regulation of biological process | 250 |
|  | GO:0051704 | multi-organism process | 196 |
|  | GO:0098754 | detoxification | 110 |
|  | GO:0040007 | growth | 107 |
|  | GO:0002376 | immune system process | 41 |
|  | GO:0048511 | rhythmic process | 21 |
|  | GO:0022610 | biological adhesion | 9 |
|  | GO:0040011 | locomotion | 5 |
|  | GO:0001906 | cell killing | 2 |
| **Total** |  |  | **45720** |

|  |  |  |  |  |  |  | **Molecular Function** |  |
| --- | --- | --- | --- | --- | --- | --- | --- | --- |

**Total**

| **GO-id** | **GO-term** | **#Seqs** |
| --- | --- | --- |
| GO:0005488 | binding | 14244 |
| GO:0003824 | catalytic activity | 11033 |
| GO:0005215 | transporter activity | 1477 |
| GO:0001071 | nucleic acid binding transcription factor activit | 546 |
| GO:0005198 | structural molecule activity | 508 |
| GO:0098772 | molecular function regulator | 353 |
| GO:0009055 | electron carrier activity | 216 |
| GO:0004871 | signal transducer activity | 189 |
| GO:0016209 | antioxidant activity | 181 |
| GO:0060089 | molecular transducer activity | 126 |
| GO:0000988 | transcription factor activity, protein binding | 111 |
| GO:0045735 | nutrient reservoir activity | 52 |
| GO:0031386 | protein tag | 5 |
| GO:0016530 | metallochaperone activity | 4 |
| GO:0045182 | translation regulator activity | 1 |
|  |  | **29046** |

|  |  |  |  |  |  |  | **Cellular component** |
| --- | --- | --- | --- | --- | --- | --- | --- |

**Total**

| **GO-id** | **GO-term** | **#Seqs** |
| --- | --- | --- |
| GO:0005623 | cell | 7247 |
| GO:0044464 | cell part | 7183 |
| GO:0016020 | membrane | 5659 |
| GO:0043226 | organelle | 4878 |
| GO:0044425 | membrane part | 4570 |
| GO:0044422 | organelle part | 2115 |
| GO:0032991 | macromolecular complex | 1861 |
| GO:0031974 | membrane-enclosed lumen | 493 |
| GO:0005576 | extracellular region | 325 |
| GO:0030054 | cell junction | 148 |
| GO:0055044 | symplast | 148 |
| GO:0099080 | supramolecular complex | 108 |
| GO:0044421 | extracellular region part | 39 |
| GO:0009295 | nucleoid | 17 |
| GO:0019012 | virion | 1 |
| GO:0044423 | virion part | 1 |
|  |  | **34793** |

**Supplementary File 4 (f). KEGG pathway details.**

| **Pathway** | **Pathway ID** | **#Enzs in Pathway** |
| --- | --- | --- |
| Glyoxylate and dicarboxylate metabolism | map00630 | 19 |
| Tryptophan metabolism | map00380 | 8 |
| Pyruvate metabolism | map00620 | 26 |
| Caffeine metabolism | map00232 | 1 |
| Monobactam biosynthesis | map00261 | 5 |
| Lipopolysaccharide biosynthesis | map00540 | 5 |
| Valine, leucine and isoleucine biosynthesis | map00290 | 7 |
| Glycosphingolipid biosynthesis - ganglio series | map00604 | 2 |
| Styrene degradation | map00643 | 3 |
| Lipoic acid metabolism | map00785 | 1 |
| Valine, leucine and isoleucine degradation | map00280 | 11 |
| Amino sugar and nucleotide sugar metabolism | map00520 | 30 |
| Toluene degradation | map00623 | 1 |
| N-Glycan biosynthesis | map00510 | 7 |
| Flavonoid biosynthesis | map00941 | 7 |
| Metabolism of xenobiotics by cytochrome P450 | map00980 | 4 |
| Monoterpenoid biosynthesis | map00902 | 1 |
| mTOR signaling pathway | map04150 | 1 |
| Aminoacyl-tRNA biosynthesis | map00970 | 22 |
| Aminobenzoate degradation | map00627 | 5 |
| Other types of O-glycan biosynthesis | map00514 | 2 |
| Novobiocin biosynthesis | map00401 | 5 |
| Tropane, piperidine and pyridine alkaloid biosynthesis | map00960 | 5 |
| Oxidative phosphorylation | map00190 | 7 |
| Taurine and hypotaurine metabolism | map00430 | 3 |
| Phosphatidylinositol signaling system | map04070 | 13 |
| Inositol phosphate metabolism | map00562 | 17 |
| beta-Alanine metabolism | map00410 | 13 |
| Fatty acid degradation | map00071 | 8 |
| Linoleic acid metabolism | map00591 | 2 |
| Drug metabolism - other enzymes | map00983 | 11 |

| Th1 and Th2 cell differentiation | map04658 | 1 |
| --- | --- | --- |
| Fatty acid biosynthesis | map00061 | 8 |
| Porphyrin and chlorophyll metabolism | map00860 | 21 |
| Fructose and mannose metabolism | map00051 | 17 |
| Sesquiterpenoid and triterpenoid biosynthesis | map00909 | 3 |
| Arginine and proline metabolism | map00330 | 15 |
| D-Arginine and D-ornithine metabolism | map00472 | 1 |
| Lysine degradation | map00310 | 7 |
| Biotin metabolism | map00780 | 7 |
| Lysine biosynthesis | map00300 | 10 |
| Pantothenate and CoA biosynthesis | map00770 | 12 |
| Nicotinate and nicotinamide metabolism | map00760 | 10 |
| Purine metabolism | map00230 | 49 |
| Vitamin B6 metabolism | map00750 | 4 |
| Arginine biosynthesis | map00220 | 15 |
| Benzoate degradation | map00362 | 3 |
| Methane metabolism | map00680 | 15 |
| One carbon pool by folate | map00670 | 14 |
| Nitrogen metabolism | map00910 | 10 |
| C5-Branched dibasic acid metabolism | map00660 | 3 |
| Terpenoid backbone biosynthesis | map00900 | 19 |
| Ubiquinone and other terpenoid-quinone biosynthesis | map00130 | 7 |
| Butanoate metabolism | map00650 | 10 |
| Primary bile acid biosynthesis | map00120 | 1 |
| Chloroalkane and chloroalkene degradation | map00625 | 2 |
| Steroid biosynthesis | map00100 | 5 |
| Glycosaminoglycan degradation | map00531 | 2 |
| Geraniol degradation | map00281 | 3 |
| Streptomycin biosynthesis | map00521 | 5 |
| Pentose phosphate pathway | map00030 | 16 |
| Peptidoglycan biosynthesis | map00550 | 7 |
| Other glycan degradation | map00511 | 7 |
| Anthocyanin biosynthesis | map00942 | 1 |

| Limonene and pinene degradation | map00903 | 2 |
| --- | --- | --- |
| Insect hormone biosynthesis | map00981 | 2 |
| Citrate cycle (TCA cycle) | map00020 | 15 |
| Glycolysis / Gluconeogenesis | map00010 | 23 |
| Mannose type O-glycan biosynthesis | map00515 | 2 |
| Glutathione metabolism | map00480 | 17 |
| Glycosaminoglycan biosynthesis - heparan sulfate / heparin | map00534 | 1 |
| Cyanoamino acid metabolism | map00460 | 5 |
| Photosynthesis | map00195 | 2 |
| Biosynthesis of antibiotics | map01130 | 153 |
| Glycosylphosphatidylinositol (GPI)-anchor biosynthesis | map00563 | 1 |
| Neomycin, kanamycin and gentamicin biosynthesis | map00524 | 2 |
| Selenocompound metabolism | map00450 | 8 |
| Synthesis and degradation of ketone bodies | map00072 | 2 |
| alpha-Linolenic acid metabolism | map00592 | 7 |
| Steroid degradation | map00984 | 2 |
| Stilbenoid, diarylheptanoid and gingerol biosynthesis | map00945 | 3 |
| Carotenoid biosynthesis | map00906 | 3 |
| Fatty acid elongation | map00062 | 4 |
| Galactose metabolism | map00052 | 14 |
| Phenazine biosynthesis | map00405 | 1 |
| Biosynthesis of unsaturated fatty acids | map01040 | 4 |
| D-Alanine metabolism | map00473 | 1 |
| Phenylalanine metabolism | map00360 | 14 |
| Atrazine degradation | map00791 | 1 |
| Sphingolipid metabolism | map00600 | 7 |
| Tyrosine metabolism | map00350 | 13 |
| Histidine metabolism | map00340 | 10 |
| Cysteine and methionine metabolism | map00270 | 32 |
| Folate biosynthesis | map00790 | 11 |
| Biosynthesis of siderophore group nonribosomal peptides | map01053 | 1 |
| Glycine, serine and threonine metabolism | map00260 | 23 |
| Starch and sucrose metabolism | map00500 | 29 |

| Alanine, aspartate and glutamate metabolism | map00250 | 23 |
| --- | --- | --- |
| Glycosphingolipid biosynthesis - globo and isoglobo series | map00603 | 3 |
| T cell receptor signaling pathway | map04660 | 1 |
| Pyrimidine metabolism | map00240 | 30 |
| Aflatoxin biosynthesis | map00254 | 1 |
| Isoquinoline alkaloid biosynthesis | map00950 | 7 |
| Phenylpropanoid biosynthesis | map00940 | 6 |
| Indole alkaloid biosynthesis | map00901 | 1 |
| Caprolactam degradation | map00930 | 4 |
| Naphthalene degradation | map00626 | 1 |
| Various types of N-glycan biosynthesis | map00513 | 4 |
| Phenylalanine, tyrosine and tryptophan biosynthesis | map00400 | 23 |
| Sulfur metabolism | map00920 | 8 |
| Steroid hormone biosynthesis | map00140 | 2 |
| Glycosaminoglycan biosynthesis - chondroitin sulfate / dermatan sulfate | map00532 | 1 |
| Glycerolipid metabolism | map00561 | 13 |
| Arachidonic acid metabolism | map00590 | 4 |
| Diterpenoid biosynthesis | map00904 | 3 |
| Drug metabolism - cytochrome P450 | map00982 | 5 |
| Retinol metabolism | map00830 | 3 |
| Ether lipid metabolism | map00565 | 4 |
| Zeatin biosynthesis | map00908 | 3 |
| Pentose and glucuronate interconversions | map00040 | 10 |
| D-Glutamine and D-glutamate metabolism | map00471 | 5 |
| Glucosinolate biosynthesis | map00966 | 1 |
| Riboflavin metabolism | map00740 | 9 |
| Glycerophospholipid metabolism | map00564 | 16 |
| Thiamine metabolism | map00730 | 8 |
| Cutin, suberine and wax biosynthesis | map00073 | 2 |
| Carbon fixation pathways in prokaryotes | map00720 | 16 |
| Carbon fixation in photosynthetic organisms | map00710 | 17 |
| Ascorbate and aldarate metabolism | map00053 | 7 |
| Biosynthesis of ansamycins | map01051 | 1 |

| Carbapenem biosynthesis | map00332 | 2 |
| --- | --- | --- |
| Propanoate metabolism | map00640 | 11 |
| Glycosphingolipid biosynthesis - lacto and neolacto series | map00601 | 1 |
