# Supplementary material for: Draft genome of the Native American cold hardy grapevine Vitis riparia Michx. ‘Manitoba 37’
Source: Hortic Res. 2020 Jun 1;7:92. doi: 10.1038/s41438-020-0316-2 (PMC7261805; doi:10.1038/s41438-020-0316-2)
Supplement: Supplementary file 10 — Supplementary Table 5 [file 41438_2020_316_MOESM10_ESM.docx]

**Supplementary Table 5a. *V. riparia* ‘Manitoba 37’ scaffold assembly organized by chromosome-level pseudomolecules.**

| **Chromosome name*** | **Scaffolds in chromosome** | **Size of chromosome (bp)** |
| --- | --- | --- |
| Chr1 | 3,210 | 25,349,035 |
| Chr2 | 1,912 | 22,590,076 |
| Chr3 | 2,373 | 19,244,387 |
| Chr4 | 2,139 | 26,918,614 |
| Chr5 | 2,496 | 23,916,886 |
| Chr6 | 1,771 | 23,549,432 |
| Chr7 | 2,361 | 28,105,821 |
| Chr8 | 1,767 | 23,829,822 |
| Chr9 | 3,142 | 22,703,660 |
| Chr10 | 3,484 | 25,867,348 |
| Chr11 | 2,570 | 20,587,031 |
| Chr12 | 3,855 | 27,721,582 |
| Chr13 | 4,885 | 27,005,731 |
| Chr14 | 4,033 | 32,410,078 |
| Chr15 | 3,071 | 23,721,285 |
| Chr16 | 4,231 | 23,320,351 |
| Chr17 | 1,785 | 19,836,558 |
| Chr18 | 4,320 | 36,514,672 |
| Chr19 | 3,707 | 23,322,797 |
| Chr20 | 2,261 | 8,996,729 |
| Chr21 | 10,243 | 9,171,054 |
| **Total** | **69,616** | **494,682,949** |

*Chromosome-level assembly obtained by mapping *V. riparia* ‘Manitoba 37’ assembly to reference genome of *V. vinifera* PN40024, 12X.2 as described in methods of mapping of BAC sequence to *V. riparia* assembly by MUMmer. Then scaffolds mapped to *V. vinifera* chromosomes were considered based on the longest alignment between them. Duplicate scaffolds removed after this step. Those scaffolds not mapped to *V. vinifera* PN40024, 12X.2 were grouped into Chr21.

| **Supplementary Table 5b. Synteny anchor statistics** | | | | | | | | |
| --- | --- | --- | --- | --- | --- | --- | --- | --- |
| **Species** | **#Anchors  Total number of anchors loaded between the two projects** | **%InBlocks**  **Percentage of anchors in synteny blocks** | **%**  **Annotated** | **%Coverage Percent of total project sequence length covered by anchor alignment regions** | **<100 bp** | **100bp-1kb** | **1kb-10kb** | **>10 kb** |
|  |  |  |  |  | **Number of anchors having alignment lengths in  given ranges** | | | |
| *V. vinifera* PN40024 12X.2 | 41,607 | 75 | 78 | 54 | 171 | 4,925 | 27,852 | 8,659 |
| *V. vinifera* ‘Sultanina’ | 45,289 | 57 | 64 | 44 | 199 | 7,404 | 29,812 | 7,874 |
| *Populus tricocarpa* | 35,235 | 57 | 95 | 13 | 288 | 14,755 | 19,923 | 269 |
| *Fragaria vesca* | 23,830 | 53 | 95 | 15 | 241 | 10,492 | 12,914 | 183 |
| *Medicago trunculata* | 27,192 | 46 | 97 | 10 | 260 | 11,539 | 14,973 | 420 |
| *Arabidopsis thaliana* | 20,796 | 40 | 99 | 17 | 274 | 10,880 | 9,600 | 42 |

| **Supplementary Table 5c. Synteny block statistics** | | | | | | | | | |
| --- | --- | --- | --- | --- | --- | --- | --- | --- | --- |
| **Species** | **#Blocks Total number of synteny blocks** | **%Coverage Percent of total project sequence length covered by synteny blocks** | **%DoubleCov Percent of total project sequence length covered by two or more synteny blocks (i.e. mapping to a putative duplication in the other genome)** | **Inverted Number of blocks which are inverted (note, blocks may contain local regions of opposite orientation)** | **%**  **Gene Hit** | **<100 kb** | **100 kb- 1 Mb** | **1 Mb- 10 Mb** | **>10 Mb** |
|  |  |  |  |  |  | **Number of blocks whose total coverage region on the project is in the given ranges** | | | |
| *V. vinifera* PN40024 12X.2 | 550 | 93 | 52 | 280 | 34 | 32 | 317 | 193 | 8 |
| *V. vinifera* ‘Sultanina’ | 1,253 | 83 | 58 | 610 | 26 | 289 | 630 | 332 | 2 |
| *Populus tricocarpa* | 769 | 90 | 63 | 379 | 19 | 53 | 465 | 249 | 2 |
| *Fragaria vesca* | 441 | 91 | 66 | 210 | 15 | 41 | 276 | 123 | 1 |
| *Medicago trunculata* | 549 | 88 | 57 | 283 | 11 | 15 | 296 | 235 | 3 |
| *Arabidopsis thaliana* | 467 | 78 | 50 | 238 | 15 | 107 | 315 | 45 | 0 |

**Supplementary Table 5d. Orthologous genes**

| **Species** | **Proteins** | **Orthologus genes** | **Orthologous common with *V. riparia*** |
| --- | --- | --- | --- |
| *Fragaria vesca* | 34,809 | 14,015 | 11,449 |
| *Populus trichocarpa* | 45,778 | 14,915 | 12,340 |
| *Arabidopsis thaliana* | 48,321 | 13,926 | 10,985 |
| *V. riparia*  ‘Manitoba 37’ | 40,019 | 19,346 | -- |
| *V. vinifera* ‘Sultanina’ | 30,544 | 17,666 | 16,176 |
| *V. vinifera* PN40024 12X.2 | 41,733 | 17,990 | 15,697 |
