# Supplementary material for: Draft genome of the Native American cold hardy grapevine Vitis riparia Michx. ‘Manitoba 37’
Source: Hortic Res. 2020 Jun 1;7:92. doi: 10.1038/s41438-020-0316-2 (PMC7261805; doi:10.1038/s41438-020-0316-2)
Supplement: Supplementary file 11 — Supplementary Table 6 [file 41438_2020_316_MOESM11_ESM.docx]

| **Supplementary Table 6a. Results of PlantTFDB for *V. riparia* 'Manitoba 37' *and V. vinifera* PN40024, 12X.2, V3 and 12X.1, V1 predicted genes.** | | | | | | | |
| --- | --- | --- | --- | --- | --- | --- | --- |
| ***V. riparia* proteins TF** | **Total No. of TF** |  | ***V. vinifera* V3 proteins TF** | **Total No. of TF** |  | ***V. vinifera* V1 proteins TF** | **Total No. of TF** |
| AP2 | 21 |  | AP2 | 18 |  | AP2 | 18 |
| ARF | 22 |  | ARF | 19 |  | ARF | 18 |
| ARR-B | 16 |  | ARR-B | 16 |  | ARR-B | 11 |
| B3 | 61 |  | B3 | 47 |  | B3 | 30 |
| BBR-BPC | 5 |  | BBR-BPC | 5 |  | BBR-BPC | 5 |
| BES1 | 10 |  | BES1 | 9 |  | BES1 | 8 |
| bHLH | 157 |  | bHLH | 141 |  | bHLH | 124 |
| bZIP | 67 |  | bZIP | 67 |  | bZIP | 56 |
| C2H2 | 100 |  | C2H2 | 93 |  | C2H2 | 80 |
| C3H | 50 |  | C3H | 47 |  | C3H | 46 |
| CAMTA | 7 |  | CAMTA | 4 |  | CAMTA | 4 |
| CO-like | 12 |  | CO-like | 12 |  | CO-like | 10 |
| CPP | 8 |  | CPP | 6 |  | CPP | 6 |
| DBB | 6 |  | DBB | 7 |  | DBB | 7 |
| Dof | 24 |  | Dof | 25 |  | Dof | 25 |
| E2F/DP | 10 |  | E2F/DP | 7 |  | E2F/DP | 7 |
| EIL | 5 |  | EIL | 4 |  | EIL | 4 |
| ERF | 56 |  | ERF | 125 |  | ERF | 113 |
| FAR1 | 48 |  | FAR1 | 65 |  | FAR1 | 36 |
| G2-like | 65 |  | G2-like | 45 |  | G2-like | 41 |
| GATA | 28 |  | GATA | 23 |  | GATA | 20 |
| GeBP | 7 |  | GeBP | 4 |  | GeBP | 3 |
| GRAS | 65 |  | GRAS | 52 |  | GRAS | 48 |
| GRF | 11 |  | GRF | 10 |  | GRF | 9 |
| HB-other | 11 |  | HB-other | 11 |  | HB-other | 11 |
| HB-PHD | 2 |  | HB-PHD | 2 |  | HB-PHD | 2 |
| HD-ZIP | 39 |  | HD-ZIP | 35 |  | HD-ZIP | 34 |
| HRT-like | 1 |  | HRT-like | 1 |  | HRT-like | 1 |
| HSF | 21 |  | HSF | 19 |  | HSF | 19 |
| LBD | 48 |  | LBD | 50 |  | LBD | 47 |
| LFY | 1 |  | LFY | 1 |  | LFY | 1 |
| LSD | 3 |  | LSD | 3 |  | LSD | 3 |
| MIKC_MADS | 41 |  | MIKC_MADS | 41 |  | MIKC_MADS | 30 |
| M-type_MADS | 48 |  | M-type_MADS | 53 |  | M-type_MADS | 31 |
| MYB | 165 |  | MYB | 152 |  | MYB | 143 |
| MYB_related | 108 |  | MYB_related | 73 |  | MYB_related | 58 |
| NAC | 99 |  | NAC | 86 |  | NAC | 74 |
| NF-X1 | 2 |  | NF-X1 | 3 |  | NF-X1 | 3 |
| NF-YA | 8 |  | NF-YA | 7 |  | NF-YA | 7 |
| NF-YB | 21 |  | NF-YB | 18 |  | NF-YB | 18 |
| NF-YC | 10 |  | NF-YC | 10 |  | NF-YC | 10 |
| Nin-like | 10 |  | Nin-like | 9 |  | Nin-like | 8 |
| NZZ/SPL | 1 |  | NZZ/SPL | 2 |  | NZZ/SPL | 2 |
| RAV | 4 |  | RAV | 4 |  | RAV | 3 |
| S1Fa-like | 2 |  | S1Fa-like | 2 |  | S1Fa-like | 2 |
| SAP | 1 |  | SAP | 1 |  | SAP | 1 |
| SBP | 21 |  | SBP | 19 |  | SBP | 19 |
| SRS | 5 |  | SRS | 6 |  | SRS | 5 |
| STAT | 1 |  | STAT | 1 |  | STAT | 1 |
| TALE | 21 |  | TALE | 21 |  | TALE | 21 |
| TCP | 19 |  | TCP | 22 |  | TCP | 18 |
| Trihelix | 39 |  | Trihelix | 39 |  | Trihelix | 37 |
| VOZ | 2 |  | VOZ | 2 |  | VOZ | 2 |
| Whirly | 2 |  | Whirly | 2 |  | Whirly | 2 |
| WOX | 11 |  | WOX | 12 |  | WOX | 11 |
| WRKY | 67 |  | WRKY | 62 |  | WRKY | 59 |
| YABBY | 10 |  | YABBY | 7 |  | YABBY | 7 |
| ZF-HD | 18 |  | ZF-HD | 18 |  | ZF-HD | 15 |
| **Grand Total** | **1723** |  | **Grand Total** | **1645** |  | **Grand Total** | **1434** |

| **Supplementary Table 6b. Results of PlantTFDB for *V. riparia* 'Manitoba 37' and *V. vinifera* PN40024, 12X.2, V3 and V1 with protein sequence information.** | | | | | | | | |  |
| --- | --- | --- | --- | --- | --- | --- | --- | --- | --- |
| ***V. riparia* protein sequence** | | **TF Family** | ***V. vinifera* V3 protein sequence** | | **TF Family** | | ***V. vinifera* V1 protein sequence** | | **TF Family** |
| g12372.t1 | | AP2 | Vitvi01g00934.t01 | | AP2 | | chr1.gff3_PROT_VIT_01s0026g01690.t01 | | AP2 |
| g14510.t1 | | AP2 | Vitvi04g01207.t01 | | AP2 | | chr11.gff3_PROT_VIT_11s0037g00870.t01 | | AP2 |
| g16916.t1 | | AP2 | Vitvi06g00187.t01 | | AP2 | | chr11.gff3_PROT_VIT_11s0052g00840.t01 | | AP2 |
| g17077.t1 | | AP2 | Vitvi06g00360.t01 | | AP2 | | chr13.gff3_PROT_VIT_13s0019g03550.t01 | | AP2 |
| g17793.t1 | | AP2 | Vitvi07g01419.t01 | | AP2 | | chr13.gff3_PROT_VIT_13s0047g00340.t01 | | AP2 |
| g17932.t1 | | AP2 | Vitvi07g01421.t01 | | AP2 | | chr14.gff3_PROT_VIT_14s0108g00050.t01 | | AP2 |
| g19616.t1 | | AP2 | Vitvi07g01546.t01 | | AP2 | | chr18.gff3_PROT_VIT_18s0001g08610.t01 | | AP2 |
| g20530.t1 | | AP2 | Vitvi07g01706.t01 | | AP2 | | chr6.gff3_PROT_VIT_06s0004g01800.t01 | | AP2 |
| g20687.t1 | | AP2 | Vitvi08g01146.t01 | | AP2 | | chr6.gff3_PROT_VIT_06s0004g03590.t01 | | AP2 |
| g24789.t1 | | AP2 | Vitvi09g00108.t01 | | AP2 | | chr7.gff3_PROT_VIT_07s0031g00220.t01 | | AP2 |
| g27006.t1 | | AP2 | Vitvi09g01269.t01 | | AP2 | | chr7_random.gff3_PROT_VIT_07s0151g00440.t01 | | AP2 |
| g31195.t1 | | AP2 | Vitvi11g00798.t01 | | AP2 | | chr8.gff3_PROT_VIT_08s0040g03180.t01 | | AP2 |
| g3645.t1 | | AP2 | Vitvi11g01231.t01 | | AP2 | | chr9.gff3_PROT_VIT_09s0002g01370.t01 | | AP2 |
| g3645.t2 | | AP2 | Vitvi13g00529.t01 | | AP2 | | chr9.gff3_PROT_VIT_09s0018g01650.t01 | | AP2 |
| g31083.t1 | | AP2 | Vitvi13g01862.t01 | | AP2 | | chrUn.gff3_PROT_VIT_00s0772g00020.t01 | | AP2 |
| g33897.t1 | | AP2 | Vitvi14g01902.t01 | | AP2 | | chrUn.gff3_PROT_VIT_00s1291g00010.t01 | | AP2 |
| g23365.t1 | | AP2 | Vitvi18g00618.t01 | | AP2 | | chr4.gff3_PROT_VIT_04s0023g00950.t01 | | AP2 |
| g20474.t1 | | AP2 | Vitvi08g01919.t01 | | AP2 | | chr8.gff3_PROT_VIT_08s0007g08580.t01 | | AP2 |
| g19817.t1 | | AP2 | Vitvi01g01759.t01 | | ARF | | chr1.gff3_PROT_VIT_01s0244g00150.t01 | | ARF |
| g17077.t2 | | AP2 | Vitvi02g00163.t01 | | ARF | | chr10.gff3_PROT_VIT_10s0003g00420.t01 | | ARF |
| g1130.t1 | | AP2 | Vitvi04g00824.t01 | | ARF | | chr10.gff3_PROT_VIT_10s0003g04100.t01 | | ARF |
| g12025.t1 | | ARF | Vitvi06g00272.t01 | | ARF | | chr11.gff3_PROT_VIT_11s0016g00640.t01 | | ARF |
| g13413.t1 | | ARF | Vitvi06g00311.t01 | | ARF | | chr11.gff3_PROT_VIT_11s0065g00310.t01 | | ARF |
| g1515.t1 | | ARF | Vitvi07g00238.t01 | | ARF | | chr12.gff3_PROT_VIT_12s0028g01170.t01 | | ARF |
| g19385.t1 | | ARF | Vitvi08g01033.t01 | | ARF | | chr12.gff3_PROT_VIT_12s0035g01800.t01 | | ARF |
| g19385.t2 | | ARF | Vitvi10g00510.t01 | | ARF | | chr13.gff3_PROT_VIT_13s0019g04380.t01 | | ARF |
| g20160.t1 | | ARF | Vitvi10g00854.t01 | | ARF | | chr15.gff3_PROT_VIT_15s0046g00290.t01 | | ARF |
| g22234.t1 | | ARF | Vitvi11g00043.t01 | | ARF | | chr17.gff3_PROT_VIT_17s0000g00320.t01 | | ARF |
| g23277.t1 | | ARF | Vitvi11g00920.t01 | | ARF | | chr18.gff3_PROT_VIT_18s0001g13930.t01 | | ARF |
| g24564.t1 | | ARF | Vitvi12g00102.t01 | | ARF | | chr18.gff3_PROT_VIT_18s0089g00910.t01 | | ARF |
| g24564.t2 | | ARF | Vitvi12g02122.t01 | | ARF | | chr2.gff3_PROT_VIT_02s0025g01740.t01 | | ARF |
| g24564.t3 | | ARF | Vitvi13g02058.t01 | | ARF | | chr4.gff3_PROT_VIT_04s0079g00200.t01 | | ARF |
| g25099.t1 | | ARF | Vitvi15g00946.t01 | | ARF | | chr6.gff3_PROT_VIT_06s0004g02750.t01 | | ARF |
| g26759.t1 | | ARF | Vitvi17g00036.t01 | | ARF | | chr6.gff3_PROT_VIT_06s0004g03130.t01 | | ARF |
| g2777.t1 | | ARF | Vitvi18g00337.t01 | | ARF | | chr7.gff3_PROT_VIT_07s0104g01230.t01 | | ARF |
| g29213.t1 | | ARF | Vitvi18g01086.t01 | | ARF | | chr8.gff3_PROT_VIT_08s0040g01810.t01 | | ARF |
| g2947.t1 | | ARF | Vitvi18g02390.t01 | | ARF | | chr1.gff3_PROT_VIT_01s0010g02230.t01 | | ARR-B |
| g31805.t1 | | ARF | Vitvi01g00341.t01 | | ARR-B | | chr1.gff3_PROT_VIT_01s0011g05830.t01 | | ARR-B |
| g31849.t1 | | ARF | Vitvi01g00344.t01 | | ARR-B | | chr11.gff3_PROT_VIT_11s0052g01160.t01 | | ARR-B |
| g32848.t1 | | ARF | Vitvi01g01506.t01 | | ARR-B | | chr11.gff3_PROT_VIT_11s0206g00060.t01 | | ARR-B |
| g33442.t1 | | ARF | Vitvi01g01959.t01 | | ARR-B | | chr16.gff3_PROT_VIT_16s0100g00420.t01 | | ARR-B |
| g34055.t1 | | ARF | Vitvi01g01995.t01 | | ARR-B | | chr17.gff3_PROT_VIT_17s0000g10100.t01 | | ARR-B |
| g939.t1 | | ARF | Vitvi02g00636.t01 | | ARR-B | | chr17.gff3_PROT_VIT_17s0000g10110.t01 | | ARR-B |
| g11888.t1 | | ARR-B | Vitvi04g00525.t01 | | ARR-B | | chr2.gff3_PROT_VIT_02s0012g00570.t01 | | ARR-B |
| g21889.t1 | | ARR-B | Vitvi05g00075.t01 | | ARR-B | | chr4.gff3_PROT_VIT_04s0008g05900.t01 | | ARR-B |
| g24435.t1 | | ARR-B | Vitvi07g00374.t01 | | ARR-B | | chr5.gff3_PROT_VIT_05s0077g01480.t01 | | ARR-B |
| g27463.t1 | | ARR-B | Vitvi11g00651.t01 | | ARR-B | | chr7.gff3_PROT_VIT_07s0005g01010.t01 | | ARR-B |
| g27463.t2 | | ARR-B | Vitvi11g01262.t01 | | ARR-B | | chr10.gff3_PROT_VIT_10s0071g00270.t01 | | B3 |
| g27706.t1 | | ARR-B | Vitvi15g00751.t01 | | ARR-B | | chr12.gff3_PROT_VIT_12s0035g02120.t01 | | B3 |
| g28658.t1 | | ARR-B | Vitvi16g00946.t01 | | ARR-B | | chr14.gff3_PROT_VIT_14s0068g01290.t01 | | B3 |
| g28659.t1 | | ARR-B | Vitvi16g00948.t01 | | ARR-B | | chr15.gff3_PROT_VIT_15s0024g00930.t01 | | B3 |
| g30131.t1 | | ARR-B | Vitvi17g01017.t01 | | ARR-B | | chr15.gff3_PROT_VIT_15s0048g02370.t01 | | B3 |
| g30136.t1 | | ARR-B | Vitvi17g01018.t01 | | ARR-B | | chr17.gff3_PROT_VIT_17s0000g10080.t01 | | B3 |
| g30137.t1 | | ARR-B | Vitvi01g01743.t01 | | B3 | | chr18.gff3_PROT_VIT_18s0001g10710.t01 | | B3 |
| g34489.t1 | | ARR-B | Vitvi02g00275.t01 | | B3 | | chr18.gff3_PROT_VIT_18s0001g11360.t01 | | B3 |
| g34709.t1 | | ARR-B | Vitvi03g00415.t01 | | B3 | | chr19.gff3_PROT_VIT_19s0014g03560.t01 | | B3 |
| g35794.t1 | | ARR-B | Vitvi03g00416.t01 | | B3 | | chr2.gff3_PROT_VIT_02s0025g03000.t01 | | B3 |
| g36453.t1 | | ARR-B | Vitvi03g00417.t01 | | B3 | | chr3.gff3_PROT_VIT_03s0063g00620.t01 | | B3 |
| g37024.t1 | | ARR-B | Vitvi03g00419.t01 | | B3 | | chr3.gff3_PROT_VIT_03s0063g00630.t01 | | B3 |
| g10322.t1 | | B3 | Vitvi03g00421.t01 | | B3 | | chr3.gff3_PROT_VIT_03s0063g00640.t01 | | B3 |
| g13116.t1 | | B3 | Vitvi03g00470.t01 | | B3 | | chr3.gff3_PROT_VIT_03s0063g00910.t01 | | B3 |
| g14169.t1 | | B3 | Vitvi03g00487.t01 | | B3 | | chr3.gff3_PROT_VIT_03s0063g01420.t01 | | B3 |
| g14169.t2 | | B3 | Vitvi03g00488.t01 | | B3 | | chr3.gff3_PROT_VIT_03s0063g01440.t01 | | B3 |
| g14311.t1 | | B3 | Vitvi03g00489.t01 | | B3 | | chr3.gff3_PROT_VIT_03s0063g01450.t01 | | B3 |
| g14779.t1 | | B3 | Vitvi03g00907.t01 | | B3 | | chr3.gff3_PROT_VIT_03s0063g01460.t01 | | B3 |
| g14780.t1 | | B3 | Vitvi03g01537.t01 | | B3 | | chr3.gff3_PROT_VIT_03s0167g00010.t01 | | B3 |
| g14781.t1 | | B3 | Vitvi03g01552.t01 | | B3 | | chr4.gff3_PROT_VIT_04s0079g00160.t01 | | B3 |
| g14784.t1 | | B3 | Vitvi06g00802.t01 | | B3 | | chr6.gff3_PROT_VIT_06s0004g08100.t01 | | B3 |
| g14784.t2 | | B3 | Vitvi06g00822.t01 | | B3 | | chr6.gff3_PROT_VIT_06s0004g08270.t01 | | B3 |
| g15228.t1 | | B3 | Vitvi07g00781.t01 | | B3 | | chr7.gff3_PROT_VIT_07s0005g05400.t01 | | B3 |
| g15228.t2 | | B3 | Vitvi07g01289.t01 | | B3 | | chr7.gff3_PROT_VIT_07s0031g00780.t01 | | B3 |
| g15228.t3 | | B3 | Vitvi07g01290.t01 | | B3 | | chr7.gff3_PROT_VIT_07s0255g00070.t01 | | B3 |
| g15832.t1 | | B3 | Vitvi07g01291.t01 | | B3 | | chr7.gff3_PROT_VIT_07s0255g00080.t01 | | B3 |
| g17307.t1 | | B3 | Vitvi07g01292.t01 | | B3 | | chr8.gff3_PROT_VIT_08s0007g02810.t01 | | B3 |
| g18136.t1 | | B3 | Vitvi07g01293.t01 | | B3 | | chr8.gff3_PROT_VIT_08s0058g01300.t01 | | B3 |
| g18317.t1 | | B3 | Vitvi07g01762.t01 | | B3 | | chrUn.gff3_PROT_VIT_00s0181g00260.t01 | | B3 |
| g20455.t1 | | B3 | Vitvi07g02422.t01 | | B3 | | chrUn.gff3_PROT_VIT_00s1203g00010.t01 | | B3 |
| g21233.t1 | | B3 | Vitvi08g00859.t01 | | B3 | | chr1.gff3_PROT_VIT_01s0011g06380.t01 | | BBR-BPC |
| g21792.t1 | | B3 | Vitvi08g01412.t01 | | B3 | | chr17.gff3_PROT_VIT_17s0000g01860.t01 | | BBR-BPC |
| g24986.t1 | | B3 | Vitvi10g00384.t01 | | B3 | | chr18.gff3_PROT_VIT_18s0001g13710.t01 | | BBR-BPC |
| g26663.t1 | | B3 | Vitvi10g02053.t01 | | B3 | | chr18.gff3_PROT_VIT_18s0001g13730.t01 | | BBR-BPC |
| g27198.t1 | | B3 | Vitvi11g00842.t01 | | B3 | | chr3.gff3_PROT_VIT_03s0038g00840.t01 | | BBR-BPC |
| g28656.t1 | | B3 | Vitvi11g00854.t01 | | B3 | | chr10.gff3_PROT_VIT_10s0003g01790.t01 | | BES1 |
| g30016.t1 | | B3 | Vitvi11g00864.t01 | | B3 | | chr10.gff3_PROT_VIT_10s0003g04710.t01 | | BES1 |
| g30439.t1 | | B3 | Vitvi11g01537.t01 | | B3 | | chr15.gff3_PROT_VIT_15s0046g02640.t01 | | BES1 |
| g32712.t1 | | B3 | Vitvi11g01539.t01 | | B3 | | chr18.gff3_PROT_VIT_18s0001g12020.t01 | | BES1 |
| g32797.t1 | | B3 | Vitvi12g02160.t01 | | B3 | | chr19.gff3_PROT_VIT_19s0014g00870.t01 | | BES1 |
| g33365.t1 | | B3 | Vitvi14g01480.t01 | | B3 | | chr19.gff3_PROT_VIT_19s0014g00880.t01 | | BES1 |
| g33366.t1 | | B3 | Vitvi15g00863.t01 | | B3 | | chr2.gff3_PROT_VIT_02s0087g00430.t01 | | BES1 |
| g33368.t1 | | B3 | Vitvi15g00981.t01 | | B3 | | chr4.gff3_PROT_VIT_04s0023g01250.t01 | | BES1 |
| g33372.t1 | | B3 | Vitvi15g01244.t01 | | B3 | | chr1.gff3_PROT_VIT_01s0010g00540.t01 | | bHLH |
| g33376.t1 | | B3 | Vitvi15g01589.t01 | | B3 | | chr1.gff3_PROT_VIT_01s0010g00740.t01 | | bHLH |
| g33377.t1 | | B3 | Vitvi15g01592.t01 | | B3 | | chr1.gff3_PROT_VIT_01s0010g02070.t01 | | bHLH |
| g33378.t1 | | B3 | Vitvi15g01593.t01 | | B3 | | chr1.gff3_PROT_VIT_01s0011g02940.t01 | | bHLH |
| g34899.t1 | | B3 | Vitvi15g01767.t01 | | B3 | | chr1.gff3_PROT_VIT_01s0011g03720.t01 | | bHLH |
| g35337.t1 | | B3 | Vitvi17g01015.t01 | | B3 | | chr1.gff3_PROT_VIT_01s0026g01140.t01 | | bHLH |
| g35338.t1 | | B3 | Vitvi18g00807.t01 | | B3 | | chr1.gff3_PROT_VIT_01s0026g02030.t01 | | bHLH |
| g35339.t1 | | B3 | Vitvi18g00865.t01 | | B3 | | chr1.gff3_PROT_VIT_01s0127g00860.t01 | | bHLH |
| g35339.t2 | | B3 | Vitvi18g00866.t01 | | B3 | | chr1.gff3_PROT_VIT_01s0127g00910.t01 | | bHLH |
| g35364.t1 | | B3 | Vitvi19g00295.t01 | | B3 | | chr1.gff3_PROT_VIT_01s0244g00010.t01 | | bHLH |
| g35701.t1 | | B3 | Vitvi01g00547.t01 | | BBR-BPC | | chr1.gff3_PROT_VIT_01s0244g00130.t01 | | bHLH |
| g35703.t1 | | B3 | Vitvi03g00058.t01 | | BBR-BPC | | chr10.gff3_PROT_VIT_10s0003g01170.t01 | | bHLH |
| g35704.t1 | | B3 | Vitvi17g00156.t01 | | BBR-BPC | | chr10.gff3_PROT_VIT_10s0003g02940.t01 | | bHLH |
| g35705.t1 | | B3 | Vitvi18g01066.t01 | | BBR-BPC | | chr10.gff3_PROT_VIT_10s0092g00030.t01 | | bHLH |
| g35706.t1 | | B3 | Vitvi18g01067.t01 | | BBR-BPC | | chr11.gff3_PROT_VIT_11s0016g00380.t01 | | bHLH |
| g35708.t1 | | B3 | Vitvi02g01232.t01 | | BES1 | | chr11.gff3_PROT_VIT_11s0016g02070.t01 | | bHLH |
| g35708.t2 | | B3 | Vitvi04g01234.t01 | | BES1 | | chr11.gff3_PROT_VIT_11s0016g03560.t01 | | bHLH |
| g35708.t3 | | B3 | Vitvi08g00772.t01 | | BES1 | | chr11.gff3_PROT_VIT_11s0037g00040.t01 | | bHLH |
| g35708.t4 | | B3 | Vitvi10g00636.t01 | | BES1 | | chr11.gff3_PROT_VIT_11s0052g00100.t01 | | bHLH |
| g36312.t1 | | B3 | Vitvi10g01901.t01 | | BES1 | | chr12.gff3_PROT_VIT_12s0028g01110.t01 | | bHLH |
| g37920.t1 | | B3 | Vitvi15g01128.t01 | | BES1 | | chr12.gff3_PROT_VIT_12s0028g02350.t01 | | bHLH |
| g37921.t1 | | B3 | Vitvi18g00924.t01 | | BES1 | | chr12.gff3_PROT_VIT_12s0028g03550.t01 | | bHLH |
| g4003.t1 | | B3 | Vitvi19g00061.t01 | | BES1 | | chr12.gff3_PROT_VIT_12s0034g02150.t01 | | bHLH |
| g4590.t1 | | B3 | Vitvi19g00063.t01 | | BES1 | | chr12.gff3_PROT_VIT_12s0059g02650.t01 | | bHLH |
| g5537.t1 | | B3 | Vitvi00g00582.t01 | | bHLH | | chr13.gff3_PROT_VIT_13s0019g00540.t01 | | bHLH |
| g6452.t1 | | B3 | Vitvi00g00944.t01 | | bHLH | | chr13.gff3_PROT_VIT_13s0047g00450.t01 | | bHLH |
| g708.t1 | | B3 | Vitvi00g01001.t01 | | bHLH | | chr13.gff3_PROT_VIT_13s0064g01290.t01 | | bHLH |
| g708.t2 | | B3 | Vitvi01g00232.t01 | | bHLH | | chr13.gff3_PROT_VIT_13s0067g01350.t01 | | bHLH |
| g709.t1 | | B3 | Vitvi01g00727.t01 | | bHLH | | chr13.gff3_PROT_VIT_13s0073g00400.t01 | | bHLH |
| g8357.t1 | | B3 | Vitvi01g00746.t01 | | bHLH | | chr14.gff3_PROT_VIT_14s0006g01470.t01 | | bHLH |
| g23094.t1 | | BBR-BPC | Vitvi01g00752.t01 | | bHLH | | chr14.gff3_PROT_VIT_14s0006g02850.t01 | | bHLH |
| g24950.t1 | | BBR-BPC | Vitvi01g00762.t01 | | bHLH | | chr14.gff3_PROT_VIT_14s0030g02230.t01 | | bHLH |
| g26801.t1 | | BBR-BPC | Vitvi01g00876.t01 | | bHLH | | chr14.gff3_PROT_VIT_14s0060g00260.t01 | | bHLH |
| g921.t1 | | BBR-BPC | Vitvi01g00964.t01 | | bHLH | | chr14.gff3_PROT_VIT_14s0068g01200.t01 | | bHLH |
| g923.t1 | | BBR-BPC | Vitvi01g01287.t01 | | bHLH | | chr14.gff3_PROT_VIT_14s0068g01580.t01 | | bHLH |
| g14262.t1 | | BES1 | Vitvi01g01316.t01 | | bHLH | | chr14.gff3_PROT_VIT_14s0081g00720.t01 | | bHLH |
| g16543.t1 | | BES1 | Vitvi01g01482.t01 | | bHLH | | chr14.gff3_PROT_VIT_14s0083g00930.t01 | | bHLH |
| g19598.t1 | | BES1 | Vitvi01g01745.t01 | | bHLH | | chr14.gff3_PROT_VIT_14s0108g00420.t01 | | bHLH |
| g20856.t1 | | BES1 | Vitvi01g01757.t01 | | bHLH | | chr14.gff3_PROT_VIT_14s0128g00110.t01 | | bHLH |
| g22864.t1 | | BES1 | Vitvi01g01946.t01 | | bHLH | | chr15.gff3_PROT_VIT_15s0021g02690.t01 | | bHLH |
| g29598.t1 | | BES1 | Vitvi01g02070.t01 | | bHLH | | chr15.gff3_PROT_VIT_15s0046g00320.t01 | | bHLH |
| g31231.t1 | | BES1 | Vitvi01g02072.t01 | | bHLH | | chr15.gff3_PROT_VIT_15s0046g02560.t01 | | bHLH |
| g37571.t1 | | BES1 | Vitvi01g02073.t01 | | bHLH | | chr15.gff3_PROT_VIT_15s0048g02510.t01 | | bHLH |
| g5825.t1 | | BES1 | Vitvi01g02074.t01 | | bHLH | | chr15.gff3_PROT_VIT_15s0048g02820.t01 | | bHLH |
| g766.t1 | | BES1 | Vitvi01g02081.t01 | | bHLH | | chr15.gff3_PROT_VIT_15s0107g00380.t01 | | bHLH |
| g10089.t1 | | bHLH | Vitvi02g00231.t01 | | bHLH | | chr16.gff3_PROT_VIT_16s0022g02240.t01 | | bHLH |
| g10718.t1 | | bHLH | Vitvi02g00317.t01 | | bHLH | | chr16.gff3_PROT_VIT_16s0022g02250.t01 | | bHLH |
| g11483.t1 | | bHLH | Vitvi02g00439.t01 | | bHLH | | chr16.gff3_PROT_VIT_16s0022g02270.t01 | | bHLH |
| g11766.t1 | | bHLH | Vitvi02g00698.t01 | | bHLH | | chr16.gff3_PROT_VIT_16s0050g02500.t01 | | bHLH |
| g11769.t1 | | bHLH | Vitvi02g00709.t01 | | bHLH | | chr17.gff3_PROT_VIT_17s0000g00330.t01 | | bHLH |
| g11828.t1 | | bHLH | Vitvi03g00098.t01 | | bHLH | | chr17.gff3_PROT_VIT_17s0000g00430.t01 | | bHLH |
| g11828.t2 | | bHLH | Vitvi03g00157.t01 | | bHLH | | chr17.gff3_PROT_VIT_17s0000g03550.t01 | | bHLH |
| g11918.t1 | | bHLH | Vitvi03g00315.t01 | | bHLH | | chr17.gff3_PROT_VIT_17s0000g03580.t01 | | bHLH |
| g11920.t1 | | bHLH | Vitvi03g00520.t01 | | bHLH | | chr17.gff3_PROT_VIT_17s0000g04790.t01 | | bHLH |
| g11922.t1 | | bHLH | Vitvi03g00635.t01 | | bHLH | | chr17.gff3_PROT_VIT_17s0000g05370.t01 | | bHLH |
| g11923.t1 | | bHLH | Vitvi03g00791.t01 | | bHLH | | chr17.gff3_PROT_VIT_17s0000g05900.t01 | | bHLH |
| g12168.t1 | | bHLH | Vitvi04g01312.t01 | | bHLH | | chr17.gff3_PROT_VIT_17s0000g05950.t01 | | bHLH |
| g12168.t2 | | bHLH | Vitvi04g01361.t01 | | bHLH | | chr17.gff3_PROT_VIT_17s0000g06930.t01 | | bHLH |
| g13360.t1 | | bHLH | Vitvi04g01463.t01 | | bHLH | | chr17.gff3_PROT_VIT_17s0000g08150.t01 | | bHLH |
| g13418.t1 | | bHLH | Vitvi04g01662.t01 | | bHLH | | chr18.gff3_PROT_VIT_18s0001g06650.t01 | | bHLH |
| g13866.t1 | | bHLH | Vitvi05g00143.t01 | | bHLH | | chr18.gff3_PROT_VIT_18s0001g07410.t01 | | bHLH |
| g14518.t1 | | bHLH | Vitvi05g00427.t01 | | bHLH | | chr18.gff3_PROT_VIT_18s0001g08040.t01 | | bHLH |
| g14997.t1 | | bHLH | Vitvi05g00624.t01 | | bHLH | | chr18.gff3_PROT_VIT_18s0001g08600.t01 | | bHLH |
| g15546.t1 | | bHLH | Vitvi05g00640.t01 | | bHLH | | chr18.gff3_PROT_VIT_18s0001g09210.t01 | | bHLH |
| g15593.t1 | | bHLH | Vitvi05g00711.t01 | | bHLH | | chr18.gff3_PROT_VIT_18s0001g10270.t01 | | bHLH |
| g15719.t1 | | bHLH | Vitvi05g01063.t01 | | bHLH | | chr18.gff3_PROT_VIT_18s0001g10300.t01 | | bHLH |
| g15734.t1 | | bHLH | Vitvi05g01103.t01 | | bHLH | | chr18.gff3_PROT_VIT_18s0001g10400.t01 | | bHLH |
| g1596.t1 | | bHLH | Vitvi05g01393.t01 | | bHLH | | chr18.gff3_PROT_VIT_18s0001g12970.t01 | | bHLH |
| g15988.t1 | | bHLH | Vitvi05g01597.t01 | | bHLH | | chr18.gff3_PROT_VIT_18s0041g00690.t01 | | bHLH |
| g16065.t1 | | bHLH | Vitvi05g01857.t01 | | bHLH | | chr19.gff3_PROT_VIT_19s0014g04670.t01 | | bHLH |
| g1607.t1 | | bHLH | Vitvi06g00181.t01 | | bHLH | | chr19.gff3_PROT_VIT_19s0014g05100.t01 | | bHLH |
| g16173.t1 | | bHLH | Vitvi06g01345.t01 | | bHLH | | chr2.gff3_PROT_VIT_02s0012g01320.t01 | | bHLH |
| g16477.t1 | | bHLH | Vitvi07g00002.t01 | | bHLH | | chr2.gff3_PROT_VIT_02s0012g01450.t01 | | bHLH |
| g16637.t1 | | bHLH | Vitvi07g00046.t01 | | bHLH | | chr2.gff3_PROT_VIT_02s0025g02610.t01 | | bHLH |
| g16794.t1 | | bHLH | Vitvi07g00139.t01 | | bHLH | | chr2.gff3_PROT_VIT_02s0025g03450.t01 | | bHLH |
| g16795.t1 | | bHLH | Vitvi07g00151.t01 | | bHLH | | chr3.gff3_PROT_VIT_03s0038g01790.t01 | | bHLH |
| g16802.t1 | | bHLH | Vitvi07g00762.t01 | | bHLH | | chr3.gff3_PROT_VIT_03s0038g02540.t01 | | bHLH |
| g16802.t2 | | bHLH | Vitvi07g01475.t01 | | bHLH | | chr3.gff3_PROT_VIT_03s0038g04760.t01 | | bHLH |
| g16896.t1 | | bHLH | Vitvi07g01529.t01 | | bHLH | | chr3.gff3_PROT_VIT_03s0063g00170.t01 | | bHLH |
| g16933.t1 | | bHLH | Vitvi07g01530.t01 | | bHLH | | chr3.gff3_PROT_VIT_03s0088g01240.t01 | | bHLH |
| g16961.t1 | | bHLH | Vitvi07g01531.t01 | | bHLH | | chr3.gff3_PROT_VIT_03s0091g00730.t01 | | bHLH |
| g17162.t1 | | bHLH | Vitvi07g01532.t01 | | bHLH | | chr4.gff3_PROT_VIT_04s0023g01930.t01 | | bHLH |
| g17162.t2 | | bHLH | Vitvi07g01545.t01 | | bHLH | | chr4.gff3_PROT_VIT_04s0023g02410.t01 | | bHLH |
| g17163.t1 | | bHLH | Vitvi07g01729.t01 | | bHLH | | chr4.gff3_PROT_VIT_04s0023g03430.t01 | | bHLH |
| g17164.t1 | | bHLH | Vitvi07g01740.t01 | | bHLH | | chr4.gff3_PROT_VIT_04s0044g01040.t01 | | bHLH |
| g1752.t1 | | bHLH | Vitvi07g02251.t01 | | bHLH | | chr5.gff3_PROT_VIT_05s0020g02700.t01 | | bHLH |
| g17557.t1 | | bHLH | Vitvi07g02613.t01 | | bHLH | | chr5.gff3_PROT_VIT_05s0020g04620.t01 | | bHLH |
| g17783.t1 | | bHLH | Vitvi07g03072.t01 | | bHLH | | chr5.gff3_PROT_VIT_05s0020g04780.t01 | | bHLH |
| g17787.t1 | | bHLH | Vitvi07g03073.t01 | | bHLH | | chr5.gff3_PROT_VIT_05s0029g00050.t01 | | bHLH |
| g17877.t1 | | bHLH | Vitvi08g00719.t01 | | bHLH | | chr5.gff3_PROT_VIT_05s0029g00390.t01 | | bHLH |
| g17930.t1 | | bHLH | Vitvi08g00979.t01 | | bHLH | | chr5.gff3_PROT_VIT_05s0049g00460.t01 | | bHLH |
| g17930.t2 | | bHLH | Vitvi08g01649.t01 | | bHLH | | chr5.gff3_PROT_VIT_05s0077g00750.t01 | | bHLH |
| g17988.t1 | | bHLH | Vitvi08g01852.t01 | | bHLH | | chr5.gff3_PROT_VIT_05s0094g00480.t01 | | bHLH |
| g18087.t1 | | bHLH | Vitvi08g01856.t01 | | bHLH | | chr5.gff3_PROT_VIT_05s0124g00240.t01 | | bHLH |
| g18087.t2 | | bHLH | Vitvi09g00227.t01 | | bHLH | | chr6.gff3_PROT_VIT_06s0004g01740.t01 | | bHLH |
| g18088.t1 | | bHLH | Vitvi09g00339.t01 | | bHLH | | chr6.gff3_PROT_VIT_06s0061g00720.t01 | | bHLH |
| g18099.t1 | | bHLH | Vitvi10g00320.t01 | | bHLH | | chr7.gff3_PROT_VIT_07s0005g02510.t01 | | bHLH |
| g18165.t1 | | bHLH | Vitvi10g00323.t01 | | bHLH | | chr7.gff3_PROT_VIT_07s0005g05100.t01 | | bHLH |
| g18285.t1 | | bHLH | Vitvi10g00572.t01 | | bHLH | | chr7.gff3_PROT_VIT_07s0031g00450.t01 | | bHLH |
| g18436.t1 | | bHLH | Vitvi10g00744.t01 | | bHLH | | chr7.gff3_PROT_VIT_07s0031g00550.t01 | | bHLH |
| g18800.t1 | | bHLH | Vitvi10g01945.t01 | | bHLH | | chr7.gff3_PROT_VIT_07s0104g00090.t01 | | bHLH |
| g19024.t1 | | bHLH | Vitvi10g02097.t01 | | bHLH | | chr7.gff3_PROT_VIT_07s0104g00250.t01 | | bHLH |
| g1933.t1 | | bHLH | Vitvi10g02164.t01 | | bHLH | | chr7.gff3_PROT_VIT_07s0141g00220.t01 | | bHLH |
| g19602.t1 | | bHLH | Vitvi10g02218.t01 | | bHLH | | chr7.gff3_PROT_VIT_07s0141g01060.t01 | | bHLH |
| g19830.t1 | | bHLH | Vitvi10g02253.t01 | | bHLH | | chr7.gff3_PROT_VIT_07s0191g00240.t01 | | bHLH |
| g19858.t1 | | bHLH | Vitvi11g00021.t01 | | bHLH | | chr7.gff3_PROT_VIT_07s0205g00160.t01 | | bHLH |
| g20488.t1 | | bHLH | Vitvi11g00165.t01 | | bHLH | | chr7.gff3_PROT_VIT_07s0205g00180.t01 | | bHLH |
| g20499.t1 | | bHLH | Vitvi11g00303.t01 | | bHLH | | chr7.gff3_PROT_VIT_07s0205g00190.t01 | | bHLH |
| g20688.t1 | | bHLH | Vitvi11g00680.t01 | | bHLH | | chr7_random.gff3_PROT_VIT_07s0151g00450.t01 | | bHLH |
| g207.t1 | | bHLH | Vitvi11g01153.t01 | | bHLH | | chr8.gff3_PROT_VIT_08s0007g05300.t01 | | bHLH |
| g20722.t1 | | bHLH | Vitvi12g00096.t01 | | bHLH | | chr8.gff3_PROT_VIT_08s0007g07810.t01 | | bHLH |
| g20723.t1 | | bHLH | Vitvi12g00200.t01 | | bHLH | | chr8.gff3_PROT_VIT_08s0007g07870.t01 | | bHLH |
| g20724.t1 | | bHLH | Vitvi12g00556.t01 | | bHLH | | chr8.gff3_PROT_VIT_08s0040g01240.t01 | | bHLH |
| g20725.t1 | | bHLH | Vitvi12g00567.t01 | | bHLH | | chr8.gff3_PROT_VIT_08s0058g00110.t01 | | bHLH |
| g20726.t1 | | bHLH | Vitvi12g00860.t01 | | bHLH | | chr9.gff3_PROT_VIT_09s0002g02700.t01 | | bHLH |
| g20727.t1 | | bHLH | Vitvi12g01896.t01 | | bHLH | | chr9.gff3_PROT_VIT_09s0002g04120.t01 | | bHLH |
| g20741.t1 | | bHLH | Vitvi12g02307.t01 | | bHLH | | chrUn.gff3_PROT_VIT_00s0274g00070.t01 | | bHLH |
| g20860.t1 | | bHLH | Vitvi13g00075.t01 | | bHLH | | chrUn.gff3_PROT_VIT_00s0384g00020.t01 | | bHLH |
| g21046.t1 | | bHLH | Vitvi13g01037.t01 | | bHLH | | chrUn.gff3_PROT_VIT_00s0479g00020.t01 | | bHLH |
| g21190.t1 | | bHLH | Vitvi13g01164.t01 | | bHLH | | chrUn.gff3_PROT_VIT_00s0532g00050.t01 | | bHLH |
| g21272.t1 | | bHLH | Vitvi13g01764.t01 | | bHLH | | chrUn.gff3_PROT_VIT_00s0824g00020.t01 | | bHLH |
| g21386.t1 | | bHLH | Vitvi13g01958.t01 | | bHLH | | chrUn.gff3_PROT_VIT_00s0927g00010.t01 | | bHLH |
| g2164.t1 | | bHLH | Vitvi14g00020.t01 | | bHLH | | chrUn.gff3_PROT_VIT_00s1312g00010.t01 | | bHLH |
| g21968.t1 | | bHLH | Vitvi14g00277.t01 | | bHLH | | chrUn.gff3_PROT_VIT_00s1314g00010.t01 | | bHLH |
| g22149.t1 | | bHLH | Vitvi14g00477.t01 | | bHLH | | chr1.gff3_PROT_VIT_01s0010g00930.t01 | | bZIP |
| g22210.t1 | | bHLH | Vitvi14g00562.t01 | | bHLH | | chr1.gff3_PROT_VIT_01s0011g03230.t01 | | bZIP |
| g22210.t2 | | bHLH | Vitvi14g00958.t01 | | bHLH | | chr12.gff3_PROT_VIT_12s0028g02590.t01 | | bZIP |
| g22443.t1 | | bHLH | Vitvi14g01181.t01 | | bHLH | | chr12.gff3_PROT_VIT_12s0034g00110.t01 | | bZIP |
| g22703.t1 | | bHLH | Vitvi14g01326.t01 | | bHLH | | chr12.gff3_PROT_VIT_12s0035g00620.t01 | | bZIP |
| g22725.t1 | | bHLH | Vitvi14g01473.t01 | | bHLH | | chr12.gff3_PROT_VIT_12s0055g00420.t01 | | bZIP |
| g23265.t1 | | bHLH | Vitvi14g01508.t01 | | bHLH | | chr13.gff3_PROT_VIT_13s0067g02900.t01 | | bZIP |
| g23275.t1 | | bHLH | Vitvi14g01926.t01 | | bHLH | | chr13.gff3_PROT_VIT_13s0084g00660.t01 | | bZIP |
| g23607.t1 | | bHLH | Vitvi14g01930.t01 | | bHLH | | chr13.gff3_PROT_VIT_13s0158g00380.t01 | | bZIP |
| g23792.t1 | | bHLH | Vitvi15g00254.t01 | | bHLH | | chr13.gff3_PROT_VIT_13s0175g00120.t01 | | bZIP |
| g23867.t1 | | bHLH | Vitvi15g00642.t01 | | bHLH | | chr14.gff3_PROT_VIT_14s0030g02200.t01 | | bZIP |
| g23867.t2 | | bHLH | Vitvi15g00876.t01 | | bHLH | | chr14.gff3_PROT_VIT_14s0060g01210.t01 | | bZIP |
| g24211.t1 | | bHLH | Vitvi15g00906.t01 | | bHLH | | chr14.gff3_PROT_VIT_14s0083g00700.t01 | | bZIP |
| g24726.t1 | | bHLH | Vitvi15g00948.t01 | | bHLH | | chr15.gff3_PROT_VIT_15s0046g01440.t01 | | bZIP |
| g2479.t1 | | bHLH | Vitvi15g01124.t01 | | bHLH | | chr18.gff3_PROT_VIT_18s0001g04470.t01 | | bZIP |
| g25515.t1 | | bHLH | Vitvi16g00882.t01 | | bHLH | | chr18.gff3_PROT_VIT_18s0001g04500.t01 | | bZIP |
| g25621.t1 | | bHLH | Vitvi16g00883.t01 | | bHLH | | chr18.gff3_PROT_VIT_18s0001g08710.t01 | | bZIP |
| g25672.t1 | | bHLH | Vitvi16g00885.t01 | | bHLH | | chr18.gff3_PROT_VIT_18s0001g10450.t01 | | bZIP |
| g25739.t1 | | bHLH | Vitvi16g01212.t01 | | bHLH | | chr18.gff3_PROT_VIT_18s0001g12120.t01 | | bZIP |
| g26492.t1 | | bHLH | Vitvi17g00037.t01 | | bHLH | | chr18.gff3_PROT_VIT_18s0001g13040.t01 | | bZIP |
| g26681.t1 | | bHLH | Vitvi17g00046.t01 | | bHLH | | chr18.gff3_PROT_VIT_18s0001g13740.t01 | | bZIP |
| g26709.t1 | | bHLH | Vitvi17g00307.t01 | | bHLH | | chr18.gff3_PROT_VIT_18s0001g14890.t01 | | bZIP |
| g2675.t1 | | bHLH | Vitvi17g00311.t01 | | bHLH | | chr18.gff3_PROT_VIT_18s0072g00470.t01 | | bZIP |
| g26766.t1 | | bHLH | Vitvi17g00442.t01 | | bHLH | | chr18.gff3_PROT_VIT_18s0076g00330.t01 | | bZIP |
| g27041.t1 | | bHLH | Vitvi17g00507.t01 | | bHLH | | chr18.gff3_PROT_VIT_18s0122g00500.t01 | | bZIP |
| g27047.t1 | | bHLH | Vitvi17g00569.t01 | | bHLH | | chr18_random.gff3_PROT_VIT_18s0001g03010.t01 | | bZIP |
| g27051.t1 | | bHLH | Vitvi17g00573.t01 | | bHLH | | chr19.gff3_PROT_VIT_19s0014g01780.t01 | | bZIP |
| g27193.t1 | | bHLH | Vitvi17g00678.t01 | | bHLH | | chr19.gff3_PROT_VIT_19s0015g01020.t01 | | bZIP |
| g27303.t1 | | bHLH | Vitvi17g00795.t01 | | bHLH | | chr2.gff3_PROT_VIT_02s0012g02250.t01 | | bZIP |
| g27614.t1 | | bHLH | Vitvi18g00463.t01 | | bHLH | | chr2.gff3_PROT_VIT_02s0025g01020.t01 | | bZIP |
| g27703.t1 | | bHLH | Vitvi18g00513.t01 | | bHLH | | chr3.gff3_PROT_VIT_03s0038g00860.t01 | | bZIP |
| g278.t1 | | bHLH | Vitvi18g00567.t01 | | bHLH | | chr3.gff3_PROT_VIT_03s0038g02420.t01 | | bZIP |
| g27957.t1 | | bHLH | Vitvi18g00617.t01 | | bHLH | | chr3.gff3_PROT_VIT_03s0038g04450.t01 | | bZIP |
| g28186.t1 | | bHLH | Vitvi18g00673.t01 | | bHLH | | chr3.gff3_PROT_VIT_03s0063g00310.t01 | | bZIP |
| g28262.t1 | | bHLH | Vitvi18g00763.t01 | | bHLH | | chr4.gff3_PROT_VIT_04s0008g02750.t01 | | bZIP |
| g2828.t1 | | bHLH | Vitvi18g00766.t01 | | bHLH | | chr4.gff3_PROT_VIT_04s0008g05210.t01 | | bZIP |
| g28584.t1 | | bHLH | Vitvi18g00779.t01 | | bHLH | | chr4.gff3_PROT_VIT_04s0023g01360.t01 | | bZIP |
| g28752.t1 | | bHLH | Vitvi18g00999.t01 | | bHLH | | chr4.gff3_PROT_VIT_04s0023g02430.t01 | | bZIP |
| g28752.t2 | | bHLH | Vitvi18g02008.t01 | | bHLH | | chr4.gff3_PROT_VIT_04s0069g01150.t01 | | bZIP |
| g28753.t1 | | bHLH | Vitvi19g00367.t01 | | bHLH | | chr5.gff3_PROT_VIT_05s0020g01090.t01 | | bZIP |
| g29112.t1 | | bHLH | Vitvi19g00406.t01 | | bHLH | | chr5.gff3_PROT_VIT_05s0077g01140.t01 | | bZIP |
| g29990.t1 | | bHLH | Vitvi01g00260.t01 | | bZIP | | chr5.gff3_PROT_VIT_05s0102g01120.t01 | | bZIP |
| g30026.t1 | | bHLH | Vitvi01g01342.t01 | | bZIP | | chr6.gff3_PROT_VIT_06s0004g08070.t01 | | bZIP |
| g30150.t1 | | bHLH | Vitvi02g00089.t01 | | bZIP | | chr6.gff3_PROT_VIT_06s0009g01790.t01 | | bZIP |
| g30562.t1 | | bHLH | Vitvi02g00796.t01 | | bZIP | | chr6.gff3_PROT_VIT_06s0080g00340.t01 | | bZIP |
| g31285.t1 | | bHLH | Vitvi03g00059.t01 | | bZIP | | chr6.gff3_PROT_VIT_06s0080g00360.t01 | | bZIP |
| g31305.t1 | | bHLH | Vitvi03g00146.t01 | | bZIP | | chr7.gff3_PROT_VIT_07s0005g01450.t01 | | bZIP |
| g31700.t1 | | bHLH | Vitvi03g00292.t01 | | bZIP | | chr7.gff3_PROT_VIT_07s0031g01320.t01 | | bZIP |
| g3195.t1 | | bHLH | Vitvi03g01513.t01 | | bZIP | | chr7.gff3_PROT_VIT_07s0141g00170.t01 | | bZIP |
| g32177.t1 | | bHLH | Vitvi03g01574.t01 | | bZIP | | chr8.gff3_PROT_VIT_08s0007g03420.t01 | | bZIP |
| g32207.t1 | | bHLH | Vitvi04g00240.t01 | | bZIP | | chr8.gff3_PROT_VIT_08s0007g03640.t01 | | bZIP |
| g32365.t1 | | bHLH | Vitvi04g00464.t01 | | bZIP | | chr8.gff3_PROT_VIT_08s0007g05170.t01 | | bZIP |
| g32746.t1 | | bHLH | Vitvi04g01363.t01 | | bZIP | | chr8.gff3_PROT_VIT_08s0007g06160.t01 | | bZIP |
| g33153.t1 | | bHLH | Vitvi04g01505.t01 | | bZIP | | chr8.gff3_PROT_VIT_08s0040g00870.t01 | | bZIP |
| g33443.t1 | | bHLH | Vitvi04g01992.t01 | | bZIP | | chrUn.gff3_PROT_VIT_00s0144g00240.t01 | | bZIP |
| g33451.t1 | | bHLH | Vitvi04g02098.t01 | | bZIP | | chrUn.gff3_PROT_VIT_00s0541g00020.t01 | | bZIP |
| g34106.t1 | | bHLH | Vitvi05g00108.t01 | | bZIP | | chr1.gff3_PROT_VIT_01s0010g02090.t01 | | C2H2 |
| g34745.t1 | | bHLH | Vitvi05g00274.t01 | | bZIP | | chr1.gff3_PROT_VIT_01s0010g02100.t01 | | C2H2 |
| g35300.t1 | | bHLH | Vitvi05g01537.t01 | | bZIP | | chr1.gff3_PROT_VIT_01s0011g00710.t01 | | C2H2 |
| g35300.t2 | | bHLH | Vitvi06g00685.t01 | | bZIP | | chr1.gff3_PROT_VIT_01s0011g03100.t01 | | C2H2 |
| g36633.t1 | | bHLH | Vitvi06g00800.t01 | | bZIP | | chr1.gff3_PROT_VIT_01s0011g05570.t01 | | C2H2 |
| g37352.t1 | | bHLH | Vitvi06g01073.t01 | | bZIP | | chr1.gff3_PROT_VIT_01s0011g06200.t01 | | C2H2 |
| g37355.t1 | | bHLH | Vitvi06g01478.t01 | | bZIP | | chr1.gff3_PROT_VIT_01s0011g06400.t01 | | C2H2 |
| g37355.t2 | | bHLH | Vitvi06g01480.t01 | | bZIP | | chr1.gff3_PROT_VIT_01s0026g00850.t01 | | C2H2 |
| g3740.t1 | | bHLH | Vitvi06g01755.t01 | | bZIP | | chr11.gff3_PROT_VIT_11s0052g01120.t01 | | C2H2 |
| g38294.t1 | | bHLH | Vitvi06g01756.t01 | | bZIP | | chr12.gff3_PROT_VIT_12s0028g03030.t01 | | C2H2 |
| g38294.t2 | | bHLH | Vitvi06g01757.t01 | | bZIP | | chr12.gff3_PROT_VIT_12s0035g00660.t01 | | C2H2 |
| g4539.t1 | | bHLH | Vitvi07g00049.t01 | | bZIP | | chr13.gff3_PROT_VIT_13s0019g00480.t01 | | C2H2 |
| g4880.t1 | | bHLH | Vitvi07g00413.t01 | | bZIP | | chr13.gff3_PROT_VIT_13s0019g02120.t01 | | C2H2 |
| g542.t1 | | bHLH | Vitvi07g01552.t01 | | bZIP | | chr13.gff3_PROT_VIT_13s0019g04530.t01 | | C2H2 |
| g546.t1 | | bHLH | Vitvi07g01807.t01 | | bZIP | | chr13.gff3_PROT_VIT_13s0019g05170.t01 | | C2H2 |
| g6133.t1 | | bHLH | Vitvi08g00950.t01 | | bZIP | | chr13.gff3_PROT_VIT_13s0064g01060.t01 | | C2H2 |
| g6133.t2 | | bHLH | Vitvi08g01470.t01 | | bZIP | | chr14.gff3_PROT_VIT_14s0060g00320.t01 | | C2H2 |
| g636.t1 | | bHLH | Vitvi08g01638.t01 | | bZIP | | chr14.gff3_PROT_VIT_14s0060g02440.t01 | | C2H2 |
| g645.t1 | | bHLH | Vitvi08g01710.t01 | | bZIP | | chr14.gff3_PROT_VIT_14s0066g01860.t01 | | C2H2 |
| g7632.t1 | | bHLH | Vitvi08g02272.t01 | | bZIP | | chr14.gff3_PROT_VIT_14s0068g01930.t01 | | C2H2 |
| g7887.t1 | | bHLH | Vitvi12g00226.t01 | | bZIP | | chr14.gff3_PROT_VIT_14s0219g00070.t01 | | C2H2 |
| g857.t1 | | bHLH | Vitvi12g01667.t01 | | bZIP | | chr15.gff3_PROT_VIT_15s0021g02720.t01 | | C2H2 |
| g1020.t1 | | bZIP | Vitvi12g01996.t01 | | bZIP | | chr16.gff3_PROT_VIT_16s0050g00700.t01 | | C2H2 |
| g11688.t1 | | bZIP | Vitvi12g02596.t01 | | bZIP | | chr17.gff3_PROT_VIT_17s0000g01870.t01 | | C2H2 |
| g1182.t1 | | bZIP | Vitvi13g00171.t01 | | bZIP | | chr17.gff3_PROT_VIT_17s0000g06820.t01 | | C2H2 |
| g1183.t1 | | bZIP | Vitvi13g01083.t01 | | bZIP | | chr17.gff3_PROT_VIT_17s0000g06830.t01 | | C2H2 |
| g1184.t1 | | bZIP | Vitvi13g01350.t01 | | bZIP | | chr17.gff3_PROT_VIT_17s0000g09980.t01 | | C2H2 |
| g12754.t1 | | bZIP | Vitvi13g01609.t01 | | bZIP | | chr18.gff3_PROT_VIT_18s0001g03670.t01 | | C2H2 |
| g13490.t1 | | bZIP | Vitvi14g00094.t01 | | bZIP | | chr18.gff3_PROT_VIT_18s0001g05860.t01 | | C2H2 |
| g13609.t1 | | bZIP | Vitvi14g00473.t01 | | bZIP | | chr18.gff3_PROT_VIT_18s0001g09230.t01 | | C2H2 |
| g13627.t1 | | bZIP | Vitvi14g00860.t01 | | bZIP | | chr18.gff3_PROT_VIT_18s0001g10130.t01 | | C2H2 |
| g14064.t1 | | bZIP | Vitvi14g00866.t01 | | bZIP | | chr18.gff3_PROT_VIT_18s0001g12220.t01 | | C2H2 |
| g14343.t1 | | bZIP | Vitvi14g01302.t01 | | bZIP | | chr18.gff3_PROT_VIT_18s0001g14130.t01 | | C2H2 |
| g15403.t1 | | bZIP | Vitvi15g01027.t01 | | bZIP | | chr18.gff3_PROT_VIT_18s0072g00840.t01 | | C2H2 |
| g15493.t1 | | bZIP | Vitvi16g01750.t01 | | bZIP | | chr19.gff3_PROT_VIT_19s0014g05000.t01 | | C2H2 |
| g15543.t1 | | bZIP | Vitvi17g00434.t01 | | bZIP | | chr19.gff3_PROT_VIT_19s0015g01780.t01 | | C2H2 |
| g15543.t2 | | bZIP | Vitvi17g01153.t01 | | bZIP | | chr2.gff3_PROT_VIT_02s0012g01190.t01 | | C2H2 |
| g16040.t1 | | bZIP | Vitvi17g01720.t01 | | bZIP | | chr2.gff3_PROT_VIT_02s0241g00170.t01 | | C2H2 |
| g16309.t1 | | bZIP | Vitvi18g00036.t01 | | bZIP | | chr3.gff3_PROT_VIT_03s0017g01020.t01 | | C2H2 |
| g16311.t1 | | bZIP | Vitvi18g00346.t01 | | bZIP | | chr3.gff3_PROT_VIT_03s0017g02130.t01 | | C2H2 |
| g16599.t1 | | bZIP | Vitvi18g00347.t01 | | bZIP | | chr3.gff3_PROT_VIT_03s0017g02160.t01 | | C2H2 |
| g17209.t1 | | bZIP | Vitvi18g00628.t01 | | bZIP | | chr3.gff3_PROT_VIT_03s0091g00690.t01 | | C2H2 |
| g17369.t1 | | bZIP | Vitvi18g00784.t01 | | bZIP | | chr3.gff3_PROT_VIT_03s0132g00020.t01 | | C2H2 |
| g17395.t1 | | bZIP | Vitvi18g00930.t01 | | bZIP | | chr4.gff3_PROT_VIT_04s0008g01160.t01 | | C2H2 |
| g17395.t2 | | bZIP | Vitvi18g01005.t01 | | bZIP | | chr4.gff3_PROT_VIT_04s0008g02540.t01 | | C2H2 |
| g17549.t1 | | bZIP | Vitvi18g01068.t01 | | bZIP | | chr4.gff3_PROT_VIT_04s0008g05930.t01 | | C2H2 |
| g17629.t1 | | bZIP | Vitvi18g01165.t01 | | bZIP | | chr4.gff3_PROT_VIT_04s0023g01430.t01 | | C2H2 |
| g17941.t1 | | bZIP | Vitvi18g01305.t01 | | bZIP | | chr5.gff3_PROT_VIT_05s0049g00070.t01 | | C2H2 |
| g18104.t1 | | bZIP | Vitvi18g01928.t01 | | bZIP | | chr5.gff3_PROT_VIT_05s0049g00080.t01 | | C2H2 |
| g1876.t1 | | bZIP | Vitvi18g02978.t01 | | bZIP | | chr5.gff3_PROT_VIT_05s0077g01390.t01 | | C2H2 |
| g19394.t1 | | bZIP | Vitvi19g00147.t01 | | bZIP | | chr6.gff3_PROT_VIT_06s0004g01870.t01 | | C2H2 |
| g19395.t1 | | bZIP | Vitvi19g02076.t01 | | bZIP | | chr6.gff3_PROT_VIT_06s0004g03070.t01 | | C2H2 |
| g19397.t1 | | bZIP | Vitvi01g00063.t01 | | C2H2 | | chr6.gff3_PROT_VIT_06s0004g04180.t01 | | C2H2 |
| g19721.t1 | | bZIP | Vitvi01g00247.t01 | | C2H2 | | chr6.gff3_PROT_VIT_06s0004g05260.t01 | | C2H2 |
| g20404.t1 | | bZIP | Vitvi01g00474.t01 | | C2H2 | | chr6.gff3_PROT_VIT_06s0004g05270.t01 | | C2H2 |
| g20681.t1 | | bZIP | Vitvi01g00529.t01 | | C2H2 | | chr6.gff3_PROT_VIT_06s0004g06240.t01 | | C2H2 |
| g20972.t1 | | bZIP | Vitvi01g00552.t01 | | C2H2 | | chr6.gff3_PROT_VIT_06s0009g02460.t01 | | C2H2 |
| g24186.t1 | | bZIP | Vitvi01g00845.t01 | | C2H2 | | chr6.gff3_PROT_VIT_06s0009g02470.t01 | | C2H2 |
| g2438.t1 | | bZIP | Vitvi01g01492.t01 | | C2H2 | | chr6.gff3_PROT_VIT_06s0061g00760.t01 | | C2H2 |
| g24947.t1 | | bZIP | Vitvi01g01493.t01 | | C2H2 | | chr6.gff3_PROT_VIT_06s0061g00780.t01 | | C2H2 |
| g25302.t1 | | bZIP | Vitvi01g02054.t01 | | C2H2 | | chr6.gff3_PROT_VIT_06s0061g01240.t01 | | C2H2 |
| g25459.t1 | | bZIP | Vitvi02g00488.t01 | | C2H2 | | chr6.gff3_PROT_VIT_06s0080g00270.t01 | | C2H2 |
| g25618.t1 | | bZIP | Vitvi02g00560.t01 | | C2H2 | | chr7.gff3_PROT_VIT_07s0005g02980.t01 | | C2H2 |
| g2602.t1 | | bZIP | Vitvi02g00686.t01 | | C2H2 | | chr7.gff3_PROT_VIT_07s0104g01620.t01 | | C2H2 |
| g27612.t1 | | bZIP | Vitvi03g00630.t01 | | C2H2 | | chr7.gff3_PROT_VIT_07s0129g00240.t01 | | C2H2 |
| g27906.t1 | | bZIP | Vitvi03g00904.t01 | | C2H2 | | chr7_random.gff3_PROT_VIT_07s0141g00020.t01 | | C2H2 |
| g27906.t2 | | bZIP | Vitvi03g01277.t01 | | C2H2 | | chr8.gff3_PROT_VIT_08s0007g03940.t01 | | C2H2 |
| g27989.t1 | | bZIP | Vitvi03g01280.t01 | | C2H2 | | chr8.gff3_PROT_VIT_08s0007g04770.t01 | | C2H2 |
| g28197.t1 | | bZIP | Vitvi03g01287.t01 | | C2H2 | | chr8.gff3_PROT_VIT_08s0007g08070.t01 | | C2H2 |
| g28739.t1 | | bZIP | Vitvi04g00096.t01 | | C2H2 | | chr8.gff3_PROT_VIT_08s0007g08360.t01 | | C2H2 |
| g3021.t1 | | bZIP | Vitvi04g00219.t01 | | C2H2 | | chr8.gff3_PROT_VIT_08s0007g09030.t01 | | C2H2 |
| g3021.t2 | | bZIP | Vitvi04g00527.t01 | | C2H2 | | chr8.gff3_PROT_VIT_08s0040g00240.t01 | | C2H2 |
| g3021.t3 | | bZIP | Vitvi04g01252.t01 | | C2H2 | | chr8.gff3_PROT_VIT_08s0040g00750.t01 | | C2H2 |
| g30436.t1 | | bZIP | Vitvi04g02200.t01 | | C2H2 | | chr8.gff3_PROT_VIT_08s0040g02100.t01 | | C2H2 |
| g30960.t1 | | bZIP | Vitvi05g00082.t01 | | C2H2 | | chr9.gff3_PROT_VIT_09s0002g02300.t01 | | C2H2 |
| g31967.t1 | | bZIP | Vitvi05g01913.t01 | | C2H2 | | chr9.gff3_PROT_VIT_09s0002g09080.t01 | | C2H2 |
| g3227.t1 | | bZIP | Vitvi05g01914.t01 | | C2H2 | | chr9.gff3_PROT_VIT_09s0054g01820.t01 | | C2H2 |
| g34429.t1 | | bZIP | Vitvi05g01915.t01 | | C2H2 | | chrUn.gff3_PROT_VIT_00s0179g00170.t01 | | C2H2 |
| g34687.t1 | | bZIP | Vitvi06g00195.t01 | | C2H2 | | chrUn.gff3_PROT_VIT_00s0229g00050.t01 | | C2H2 |
| g34792.t1 | | bZIP | Vitvi06g00304.t01 | | C2H2 | | chrUn.gff3_PROT_VIT_00s2480g00010.t01 | | C2H2 |
| g3518.t1 | | bZIP | Vitvi06g00521.t01 | | C2H2 | | chr1.gff3_PROT_VIT_01s0010g01770.t01 | | C3H |
| g35228.t1 | | bZIP | Vitvi06g00607.t01 | | C2H2 | | chr1.gff3_PROT_VIT_01s0011g04260.t01 | | C3H |
| g35313.t1 | | bZIP | Vitvi06g01350.t01 | | C2H2 | | chr10.gff3_PROT_VIT_10s0003g00110.t01 | | C3H |
| g36698.t1 | | bZIP | Vitvi06g01353.t01 | | C2H2 | | chr11.gff3_PROT_VIT_11s0016g02020.t01 | | C3H |
| g36972.t1 | | bZIP | Vitvi06g01399.t01 | | C2H2 | | chr11.gff3_PROT_VIT_11s0016g02130.t01 | | C3H |
| g37545.t1 | | bZIP | Vitvi06g01471.t01 | | C2H2 | | chr11.gff3_PROT_VIT_11s0016g02790.t01 | | C3H |
| g773.t1 | | bZIP | Vitvi06g01682.t01 | | C2H2 | | chr11.gff3_PROT_VIT_11s0016g04280.t01 | | C3H |
| g865.t1 | | bZIP | Vitvi06g01710.t01 | | C2H2 | | chr11.gff3_PROT_VIT_11s0016g04940.t01 | | C3H |
| g924.t1 | | bZIP | Vitvi06g01861.t01 | | C2H2 | | chr11.gff3_PROT_VIT_11s0016g04980.t01 | | C3H |
| g11491.t1 | | C2H2 | Vitvi06g01862.t01 | | C2H2 | | chr12.gff3_PROT_VIT_12s0028g03300.t01 | | C3H |
| g11492.t1 | | C2H2 | Vitvi06g01864.t01 | | C2H2 | | chr12.gff3_PROT_VIT_12s0035g01790.t01 | | C3H |
| g11911.t1 | | C2H2 | Vitvi06g01865.t01 | | C2H2 | | chr12.gff3_PROT_VIT_12s0059g01450.t01 | | C3H |
| g12009.t1 | | C2H2 | Vitvi06g01866.t01 | | C2H2 | | chr12.gff3_PROT_VIT_12s0059g02450.t01 | | C3H |
| g12159.t1 | | C2H2 | Vitvi06g01867.t01 | | C2H2 | | chr13.gff3_PROT_VIT_13s0064g00920.t01 | | C3H |
| g1266.t1 | | C2H2 | Vitvi07g00077.t01 | | C2H2 | | chr13.gff3_PROT_VIT_13s0064g01090.t01 | | C3H |
| g13601.t1 | | C2H2 | Vitvi07g00271.t01 | | C2H2 | | chr13.gff3_PROT_VIT_13s0067g01070.t01 | | C3H |
| g13602.t1 | | C2H2 | Vitvi07g01366.t01 | | C2H2 | | chr13.gff3_PROT_VIT_13s0156g00320.t01 | | C3H |
| g1364.t1 | | C2H2 | Vitvi07g01602.t01 | | C2H2 | | chr14.gff3_PROT_VIT_14s0006g02450.t01 | | C3H |
| g1365.t1 | | C2H2 | Vitvi07g01724.t01 | | C2H2 | | chr14.gff3_PROT_VIT_14s0036g00380.t01 | | C3H |
| g13963.t1 | | C2H2 | Vitvi07g02261.t01 | | C2H2 | | chr14.gff3_PROT_VIT_14s0060g00610.t01 | | C3H |
| g13964.t1 | | C2H2 | Vitvi07g02262.t01 | | C2H2 | | chr14.gff3_PROT_VIT_14s0219g00170.t01 | | C3H |
| g13965.t1 | | C2H2 | Vitvi07g03165.t01 | | C2H2 | | chr15.gff3_PROT_VIT_15s0046g01030.t01 | | C3H |
| g13966.t1 | | C2H2 | Vitvi08g00893.t01 | | C2H2 | | chr16.gff3_PROT_VIT_16s0013g00870.t01 | | C3H |
| g13968.t1 | | C2H2 | Vitvi08g01050.t01 | | C2H2 | | chr16.gff3_PROT_VIT_16s0022g02520.t01 | | C3H |
| g13969.t1 | | C2H2 | Vitvi08g01518.t01 | | C2H2 | | chr16.gff3_PROT_VIT_16s0039g01570.t01 | | C3H |
| g14079.t1 | | C2H2 | Vitvi08g01600.t01 | | C2H2 | | chr16.gff3_PROT_VIT_16s0100g00320.t01 | | C3H |
| g14080.t1 | | C2H2 | Vitvi08g01869.t01 | | C2H2 | | chr17.gff3_PROT_VIT_17s0000g09990.t01 | | C3H |
| g14081.t1 | | C2H2 | Vitvi08g01895.t01 | | C2H2 | | chr18.gff3_PROT_VIT_18s0001g15570.t01 | | C3H |
| g14129.t1 | | C2H2 | Vitvi08g02113.t01 | | C2H2 | | chr19.gff3_PROT_VIT_19s0085g01180.t01 | | C3H |
| g14129.t2 | | C2H2 | Vitvi08g02203.t01 | | C2H2 | | chr2.gff3_PROT_VIT_02s0012g01550.t01 | | C3H |
| g14501.t1 | | C2H2 | Vitvi09g00191.t01 | | C2H2 | | chr4.gff3_PROT_VIT_04s0008g00610.t01 | | C3H |
| g1475.t1 | | C2H2 | Vitvi09g00827.t01 | | C2H2 | | chr4.gff3_PROT_VIT_04s0023g00370.t01 | | C3H |
| g15706.t1 | | C2H2 | Vitvi09g01479.t01 | | C2H2 | | chr4.gff3_PROT_VIT_04s0023g02950.t01 | | C3H |
| g15881.t1 | | C2H2 | Vitvi11g01259.t01 | | C2H2 | | chr5.gff3_PROT_VIT_05s0020g01650.t01 | | C3H |
| g16053.t1 | | C2H2 | Vitvi12g00252.t01 | | C2H2 | | chr6.gff3_PROT_VIT_06s0004g01290.t01 | | C3H |
| g16317.t1 | | C2H2 | Vitvi12g02001.t01 | | C2H2 | | chr6.gff3_PROT_VIT_06s0004g02160.t01 | | C3H |
| g1649.t1 | | C2H2 | Vitvi13g00262.t01 | | C2H2 | | chr6.gff3_PROT_VIT_06s0004g06710.t01 | | C3H |
| g16570.t1 | | C2H2 | Vitvi13g00340.t01 | | C2H2 | | chr7.gff3_PROT_VIT_07s0031g01870.t01 | | C3H |
| g16866.t1 | | C2H2 | Vitvi13g00630.t01 | | C2H2 | | chr7.gff3_PROT_VIT_07s0104g01460.t01 | | C3H |
| g17428.t1 | | C2H2 | Vitvi13g00694.t01 | | C2H2 | | chr8.gff3_PROT_VIT_08s0007g06400.t01 | | C3H |
| g17509.t1 | | C2H2 | Vitvi13g01403.t01 | | C2H2 | | chr8.gff3_PROT_VIT_08s0007g07440.t01 | | C3H |
| g1758.t1 | | C2H2 | Vitvi13g01748.t01 | | C2H2 | | chr8.gff3_PROT_VIT_08s0007g08210.t01 | | C3H |
| g17694.t1 | | C2H2 | Vitvi14g00028.t01 | | C2H2 | | chr9.gff3_PROT_VIT_09s0002g00800.t01 | | C3H |
| g17695.t2 | | C2H2 | Vitvi14g00197.t01 | | C2H2 | | chr9.gff3_PROT_VIT_09s0002g06130.t01 | | C3H |
| g17805.t1 | | C2H2 | Vitvi14g01533.t01 | | C2H2 | | chr9.gff3_PROT_VIT_09s0070g00470.t01 | | C3H |
| g17991.t1 | | C2H2 | Vitvi14g01617.t01 | | C2H2 | | chrUn.gff3_PROT_VIT_00s0279g00070.t01 | | C3H |
| g18076.t1 | | C2H2 | Vitvi14g01822.t01 | | C2H2 | | chr1.gff3_PROT_VIT_01s0010g03850.t01 | | CAMTA |
| g18280.t1 | | C2H2 | Vitvi14g02033.t01 | | C2H2 | | chr5.gff3_PROT_VIT_05s0020g00630.t01 | | CAMTA |
| g18417.t1 | | C2H2 | Vitvi14g02957.t01 | | C2H2 | | chr5.gff3_PROT_VIT_05s0077g01240.t01 | | CAMTA |
| g18487.t1 | | C2H2 | Vitvi15g01472.t01 | | C2H2 | | chr7_random.gff3_PROT_VIT_07s0141g00250.t01 | | CAMTA |
| g19029.t1 | | C2H2 | Vitvi16g00499.t01 | | C2H2 | | chr1.gff3_PROT_VIT_01s0011g03520.t01 | | CO-like |
| g19031.t1 | | C2H2 | Vitvi16g01067.t01 | | C2H2 | | chr1.gff3_PROT_VIT_01s0146g00360.t01 | | CO-like |
| g19086.t1 | | C2H2 | Vitvi17g00157.t01 | | C2H2 | | chr11.gff3_PROT_VIT_11s0052g01800.t01 | | CO-like |
| g19262.t1 | | C2H2 | Vitvi17g00670.t01 | | C2H2 | | chr12.gff3_PROT_VIT_12s0057g01350.t01 | | CO-like |
| g19352.t1 | | C2H2 | Vitvi17g01001.t01 | | C2H2 | | chr12.gff3_PROT_VIT_12s0059g02500.t01 | | CO-like |
| g19478.t1 | | C2H2 | Vitvi17g01488.t01 | | C2H2 | | chr14.gff3_PROT_VIT_14s0068g01380.t01 | | CO-like |
| g19865.t1 | | C2H2 | Vitvi18g00316.t01 | | C2H2 | | chr14.gff3_PROT_VIT_14s0083g00640.t01 | | CO-like |
| g19865.t2 | | C2H2 | Vitvi18g00417.t01 | | C2H2 | | chr4.gff3_PROT_VIT_04s0008g07340.t01 | | CO-like |
| g20005.t1 | | C2H2 | Vitvi18g00675.t01 | | C2H2 | | chr7.gff3_PROT_VIT_07s0104g01360.t01 | | CO-like |
| g20099.t1 | | C2H2 | Vitvi18g00755.t01 | | C2H2 | | chrUn.gff3_PROT_VIT_00s0194g00070.t01 | | CO-like |
| g20508.t1 | | C2H2 | Vitvi18g00935.t01 | | C2H2 | | chr11.gff3_PROT_VIT_11s0016g03680.t01 | | CPP |
| g21936.t1 | | C2H2 | Vitvi18g01109.t01 | | C2H2 | | chr14.gff3_PROT_VIT_14s0030g01800.t01 | | CPP |
| g23096.t1 | | C2H2 | Vitvi18g01662.t01 | | C2H2 | | chr4.gff3_PROT_VIT_04s0008g03670.t01 | | CPP |
| g23990.t1 | | C2H2 | Vitvi19g00397.t01 | | C2H2 | | chr4.gff3_PROT_VIT_04s0008g05130.t01 | | CPP |
| g23990.t2 | | C2H2 | Vitvi19g00912.t01 | | C2H2 | | chr5.gff3_PROT_VIT_05s0020g03880.t01 | | CPP |
| g24399.t1 | | C2H2 | Vitvi01g00347.t01 | | C3H | | chr9.gff3_PROT_VIT_09s0002g06020.t01 | | CPP |
| g24458.t1 | | C2H2 | Vitvi01g01447.t01 | | C3H | | chr18.gff3_PROT_VIT_18s0001g13520.t01 | | DBB |
| g24899.t1 | | C2H2 | Vitvi02g00715.t01 | | C3H | | chr18.gff3_PROT_VIT_18s0089g01280.t01 | | DBB |
| g25394.t1 | | C2H2 | Vitvi04g00047.t01 | | C3H | | chr19.gff3_PROT_VIT_19s0014g00350.t01 | | DBB |
| g25395.t1 | | C2H2 | Vitvi04g01157.t01 | | C3H | | chr3.gff3_PROT_VIT_03s0038g00340.t01 | | DBB |
| g25753.t1 | | C2H2 | Vitvi05g00333.t01 | | C3H | | chr3.gff3_PROT_VIT_03s0038g00690.t01 | | DBB |
| g25813.t1 | | C2H2 | Vitvi06g00123.t01 | | C3H | | chr4.gff3_PROT_VIT_04s0023g03030.t01 | | DBB |
| g26802.t1 | | C2H2 | Vitvi06g00218.t01 | | C3H | | chr5.gff3_PROT_VIT_05s0102g00750.t01 | | DBB |
| g26856.t1 | | C2H2 | Vitvi06g00504.t01 | | C3H | | chr1.gff3_PROT_VIT_01s0026g02580.t01 | | Dof |
| g27150.t1 | | C2H2 | Vitvi06g00653.t01 | | C3H | | chr10.gff3_PROT_VIT_10s0003g00030.t01 | | Dof |
| g2742.t1 | | C2H2 | Vitvi07g00262.t01 | | C3H | | chr10.gff3_PROT_VIT_10s0003g00040.t01 | | Dof |
| g27454.t1 | | C2H2 | Vitvi07g01864.t01 | | C3H | | chr10.gff3_PROT_VIT_10s0003g01260.t01 | | Dof |
| g28576.t1 | | C2H2 | Vitvi07g02693.t01 | | C3H | | chr13.gff3_PROT_VIT_13s0019g01410.t01 | | Dof |
| g28648.t1 | | C2H2 | Vitvi08g01732.t01 | | C3H | | chr14.gff3_PROT_VIT_14s0108g00980.t01 | | Dof |
| g30002.t1 | | C2H2 | Vitvi08g01816.t01 | | C3H | | chr15.gff3_PROT_VIT_15s0046g00150.t01 | | Dof |
| g30414.t1 | | C2H2 | Vitvi08g01880.t01 | | C3H | | chr16.gff3_PROT_VIT_16s0098g01420.t01 | | Dof |
| g30654.t1 | | C2H2 | Vitvi09g00059.t01 | | C3H | | chr17.gff3_PROT_VIT_17s0000g04850.t01 | | Dof |
| g31609.t1 | | C2H2 | Vitvi09g00531.t01 | | C3H | | chr17.gff3_PROT_VIT_17s0000g06310.t01 | | Dof |
| g31841.t1 | | C2H2 | Vitvi09g01011.t01 | | C3H | | chr17.gff3_PROT_VIT_17s0000g08290.t01 | | Dof |
| g3252.t1 | | C2H2 | Vitvi10g00486.t01 | | C3H | | chr18.gff3_PROT_VIT_18s0001g11310.t01 | | Dof |
| g33230.t1 | | C2H2 | Vitvi11g00170.t01 | | C3H | | chr18.gff3_PROT_VIT_18s0001g15730.t01 | | Dof |
| g3345.t1 | | C2H2 | Vitvi11g00229.t01 | | C3H | | chr2.gff3_PROT_VIT_02s0025g02250.t01 | | Dof |
| g33635.t1 | | C2H2 | Vitvi11g00372.t01 | | C3H | | chr3.gff3_PROT_VIT_03s0063g01350.t01 | | Dof |
| g33635.t2 | | C2H2 | Vitvi11g00436.t01 | | C3H | | chr6.gff3_PROT_VIT_06s0004g03420.t01 | | Dof |
| g33750.t1 | | C2H2 | Vitvi11g00440.t01 | | C3H | | chr6.gff3_PROT_VIT_06s0004g04520.t01 | | Dof |
| g340.t1 | | C2H2 | Vitvi11g01367.t01 | | C3H | | chr7.gff3_PROT_VIT_07s0255g00020.t01 | | Dof |
| g34094.t1 | | C2H2 | Vitvi12g00276.t01 | | C3H | | chr8.gff3_PROT_VIT_08s0007g00180.t01 | | Dof |
| g34116.t1 | | C2H2 | Vitvi12g00449.t01 | | C3H | | chr8.gff3_PROT_VIT_08s0056g01230.t01 | | Dof |
| g34501.t1 | | C2H2 | Vitvi12g00535.t01 | | C3H | | chr8.gff3_PROT_VIT_08s0105g00170.t01 | | Dof |
| g34502.t1 | | C2H2 | Vitvi12g02121.t01 | | C3H | | chr9.gff3_PROT_VIT_09s0002g02490.t01 | | Dof |
| g34681.t1 | | C2H2 | Vitvi13g00058.t01 | | C3H | | chrUn.gff3_PROT_VIT_00s0218g00040.t01 | | Dof |
| g34786.t1 | | C2H2 | Vitvi13g01732.t01 | | C3H | | chrUn.gff3_PROT_VIT_00s0253g00060.t01 | | Dof |
| g3487.t1 | | C2H2 | Vitvi13g01750.t01 | | C3H | | chrUn.gff3_PROT_VIT_00s0652g00010.t01 | | Dof |
| g35797.t1 | | C2H2 | Vitvi13g01819.t01 | | C3H | | chr14.gff3_PROT_VIT_14s0066g02370.t01 | | E2F/DP |
| g36508.t1 | | C2H2 | Vitvi14g00599.t01 | | C3H | | chr17.gff3_PROT_VIT_17s0000g07630.t01 | | E2F/DP |
| g36513.t1 | | C2H2 | Vitvi14g01114.t01 | | C3H | | chr18.gff3_PROT_VIT_18s0001g14110.t01 | | E2F/DP |
| g36517.t1 | | C2H2 | Vitvi14g01629.t01 | | C3H | | chr6.gff3_PROT_VIT_06s0080g01110.t01 | | E2F/DP |
| g36519.t1 | | C2H2 | Vitvi14g02452.t01 | | C3H | | chr8.gff3_PROT_VIT_08s0007g00170.t01 | | E2F/DP |
| g36703.t1 | | C2H2 | Vitvi15g00994.t01 | | C3H | | chr8.gff3_PROT_VIT_08s0007g02510.t01 | | E2F/DP |
| g3698.t1 | | C2H2 | Vitvi16g00084.t01 | | C3H | | chr8.gff3_PROT_VIT_08s0040g01330.t01 | | E2F/DP |
| g4968.t1 | | C2H2 | Vitvi16g00346.t01 | | C3H | | chr13.gff3_PROT_VIT_13s0047g00250.t01 | | EIL |
| g7755.t1 | | C2H2 | Vitvi16g00933.t01 | | C3H | | chr6.gff3_PROT_VIT_06s0004g01610.t01 | | EIL |
| g783.t1 | | C2H2 | Vitvi16g01601.t01 | | C3H | | chr6.gff3_PROT_VIT_06s0009g01380.t01 | | EIL |
| g960.t1 | | C2H2 | Vitvi16g01835.t01 | | C3H | | chrUn.gff3_PROT_VIT_00s0357g00120.t01 | | EIL |
| g11628.t1 | | C3H | Vitvi17g01002.t01 | | C3H | | chr1.gff3_PROT_VIT_01s0011g03470.t01 | | ERF |
| g11879.t1 | | C3H | Vitvi18g01240.t01 | | C3H | | chr1.gff3_PROT_VIT_01s0150g00120.t01 | | ERF |
| g1218.t1 | | C3H | Vitvi19g01773.t01 | | C3H | | chr10.gff3_PROT_VIT_10s0003g00130.t01 | | ERF |
| g12212.t1 | | C3H | Vitvi01g01671.t01 | | CAMTA | | chr10.gff3_PROT_VIT_10s0003g00140.t01 | | ERF |
| g12998.t1 | | C3H | Vitvi05g00096.t01 | | CAMTA | | chr10.gff3_PROT_VIT_10s0003g00580.t01 | | ERF |
| g136.t1 | | C3H | Vitvi05g00239.t01 | | CAMTA | | chr10.gff3_PROT_VIT_10s0003g00590.t01 | | ERF |
| g14468.t1 | | C3H | Vitvi14g00629.t01 | | CAMTA | | chr11.gff3_PROT_VIT_11s0016g00660.t01 | | ERF |
| g14572.t1 | | C3H | Vitvi01g00288.t01 | | CO-like | | chr11.gff3_PROT_VIT_11s0016g00670.t01 | | ERF |
| g14919.t1 | | C3H | Vitvi01g01729.t01 | | CO-like | | chr11.gff3_PROT_VIT_11s0016g02140.t01 | | ERF |
| g1492.t1 | | C3H | Vitvi04g00665.t01 | | CO-like | | chr11.gff3_PROT_VIT_11s0016g03350.t01 | | ERF |
| g15743.t1 | | C3H | Vitvi07g00252.t01 | | CO-like | | chr11.gff3_PROT_VIT_11s0016g05340.t01 | | ERF |
| g16327.t1 | | C3H | Vitvi10g00219.t01 | | CO-like | | chr12.gff3_PROT_VIT_12s0028g03270.t01 | | ERF |
| g17081.t1 | | C3H | Vitvi11g01309.t01 | | CO-like | | chr12.gff3_PROT_VIT_12s0059g00280.t01 | | ERF |
| g17653.t1 | | C3H | Vitvi12g00542.t01 | | CO-like | | chr12.gff3_PROT_VIT_12s0059g01460.t01 | | ERF |
| g17747.t1 | | C3H | Vitvi12g00757.t01 | | CO-like | | chr13.gff3_PROT_VIT_13s0067g01400.t01 | | ERF |
| g17821.t1 | | C3H | Vitvi14g01296.t01 | | CO-like | | chr13.gff3_PROT_VIT_13s0067g01960.t01 | | ERF |
| g18403.t1 | | C3H | Vitvi14g01487.t01 | | CO-like | | chr14.gff3_PROT_VIT_14s0006g02290.t01 | | ERF |
| g18420.t1 | | C3H | Vitvi17g00328.t01 | | CO-like | | chr14.gff3_PROT_VIT_14s0068g00860.t01 | | ERF |
| g19954.t1 | | C3H | Vitvi19g00408.t01 | | CO-like | | chr14.gff3_PROT_VIT_14s0068g01040.t01 | | ERF |
| g20338.t1 | | C3H | Vitvi04g00320.t01 | | CPP | | chr14.gff3_PROT_VIT_14s0081g00520.t01 | | ERF |
| g21012.t1 | | C3H | Vitvi04g00455.t01 | | CPP | | chr14.gff3_PROT_VIT_14s0081g00730.t01 | | ERF |
| g21494.t1 | | C3H | Vitvi05g00541.t01 | | CPP | | chr14.gff3_PROT_VIT_14s0108g01220.t01 | | ERF |
| g21735.t1 | | C3H | Vitvi09g00519.t01 | | CPP | | chr15.gff3_PROT_VIT_15s0021g01590.t01 | | ERF |
| g2242.t1 | | C3H | Vitvi11g00314.t01 | | CPP | | chr15.gff3_PROT_VIT_15s0021g01600.t01 | | ERF |
| g2312.t1 | | C3H | Vitvi14g00428.t01 | | CPP | | chr15.gff3_PROT_VIT_15s0021g01610.t01 | | ERF |
| g2316.t1 | | C3H | Vitvi03g00026.t01 | | DBB | | chr15.gff3_PROT_VIT_15s0021g01630.t01 | | ERF |
| g23341.t1 | | C3H | Vitvi03g00049.t01 | | DBB | | chr15.gff3_PROT_VIT_15s0021g02110.t01 | | ERF |
| g23494.t1 | | C3H | Vitvi04g01423.t01 | | DBB | | chr15.gff3_PROT_VIT_15s0046g00310.t01 | | ERF |
| g23594.t1 | | C3H | Vitvi05g01519.t01 | | DBB | | chr15.gff3_PROT_VIT_15s0046g01390.t01 | | ERF |
| g24563.t1 | | C3H | Vitvi18g01048.t01 | | DBB | | chr16.gff3_PROT_VIT_16s0013g00890.t01 | | ERF |
| g24679.t1 | | C3H | Vitvi18g02424.t01 | | DBB | | chr16.gff3_PROT_VIT_16s0013g00900.t01 | | ERF |
| g25171.t1 | | C3H | Vitvi19g00031.t01 | | DBB | | chr16.gff3_PROT_VIT_16s0013g00950.t01 | | ERF |
| g26472.t1 | | C3H | Vitvi01g01026.t01 | | Dof | | chr16.gff3_PROT_VIT_16s0013g00970.t01 | | ERF |
| g2657.t1 | | C3H | Vitvi02g00199.t01 | | Dof | | chr16.gff3_PROT_VIT_16s0013g00980.t01 | | ERF |
| g28378.t1 | | C3H | Vitvi03g00430.t01 | | Dof | | chr16.gff3_PROT_VIT_16s0013g00990.t01 | | ERF |
| g28560.t1 | | C3H | Vitvi06g00345.t01 | | Dof | | chr16.gff3_PROT_VIT_16s0013g01000.t01 | | ERF |
| g28649.t1 | | C3H | Vitvi06g00449.t01 | | Dof | | chr16.gff3_PROT_VIT_16s0013g01050.t01 | | ERF |
| g29243.t1 | | C3H | Vitvi07g01298.t01 | | Dof | | chr16.gff3_PROT_VIT_16s0013g01060.t01 | | ERF |
| g29869.t1 | | C3H | Vitvi07g01306.t01 | | Dof | | chr16.gff3_PROT_VIT_16s0013g01070.t01 | | ERF |
| g30129.t1 | | C3H | Vitvi07g01401.t01 | | Dof | | chr16.gff3_PROT_VIT_16s0013g01090.t01 | | ERF |
| g32251.t1 | | C3H | Vitvi08g00110.t01 | | Dof | | chr16.gff3_PROT_VIT_16s0013g01110.t01 | | ERF |
| g32454.t1 | | C3H | Vitvi08g00626.t01 | | Dof | | chr16.gff3_PROT_VIT_16s0013g01120.t01 | | ERF |
| g3286.t1 | | C3H | Vitvi08g01186.t01 | | Dof | | chr16.gff3_PROT_VIT_16s0050g02400.t01 | | ERF |
| g37338.t1 | | C3H | Vitvi09g00210.t01 | | Dof | | chr16.gff3_PROT_VIT_16s0100g00380.t01 | | ERF |
| g38221.t1 | | C3H | Vitvi10g00444.t01 | | Dof | | chr16.gff3_PROT_VIT_16s0100g00400.t01 | | ERF |
| g38286.t1 | | C3H | Vitvi10g00479.t01 | | Dof | | chr17.gff3_PROT_VIT_17s0000g00200.t01 | | ERF |
| g38298.t1 | | C3H | Vitvi10g00480.t01 | | Dof | | chr17.gff3_PROT_VIT_17s0000g04480.t01 | | ERF |
| g3906.t1 | | C3H | Vitvi10g00581.t01 | | Dof | | chr17.gff3_PROT_VIT_17s0000g07940.t01 | | ERF |
| g4166.t1 | | C3H | Vitvi13g00298.t01 | | Dof | | chr18.gff3_PROT_VIT_18s0001g03240.t01 | | ERF |
| g4911.t1 | | C3H | Vitvi14g01973.t01 | | Dof | | chr18.gff3_PROT_VIT_18s0001g05250.t01 | | ERF |
| g2430.t1 | | CAMTA | Vitvi15g00936.t01 | | Dof | | chr18.gff3_PROT_VIT_18s0001g10150.t01 | | ERF |
| g2557.t1 | | CAMTA | Vitvi16g01384.t01 | | Dof | | chr18.gff3_PROT_VIT_18s0001g13320.t01 | | ERF |
| g33239.t1 | | CAMTA | Vitvi17g00447.t01 | | Dof | | chr18.gff3_PROT_VIT_18s0072g00260.t01 | | ERF |
| g33239.t2 | | CAMTA | Vitvi17g00611.t01 | | Dof | | chr18.gff3_PROT_VIT_18s0089g01030.t01 | | ERF |
| g35671.t1 | | CAMTA | Vitvi17g00809.t01 | | Dof | | chr18_random.gff3_PROT_VIT_18s0001g05850.t01 | | ERF |
| g35671.t2 | | CAMTA | Vitvi18g00858.t01 | | Dof | | chr18_random.gff3_PROT_VIT_18s0001g05890.t01 | | ERF |
| g35671.t3 | | CAMTA | Vitvi18g01254.t01 | | Dof | | chr19.gff3_PROT_VIT_19s0014g02240.t01 | | ERF |
| g11750.t1 | | CO-like | Vitvi06g01552.t01 | | E2F/DP | | chr19.gff3_PROT_VIT_19s0014g03180.t01 | | ERF |
| g1503.t1 | | CO-like | Vitvi08g00987.t01 | | E2F/DP | | chr19.gff3_PROT_VIT_19s0090g01080.t01 | | ERF |
| g16008.t1 | | CO-like | Vitvi08g01185.t01 | | E2F/DP | | chr2.gff3_PROT_VIT_02s0025g01360.t01 | | ERF |
| g23250.t1 | | CO-like | Vitvi08g01388.t01 | | E2F/DP | | chr2.gff3_PROT_VIT_02s0025g03170.t01 | | ERF |
| g23599.t1 | | CO-like | Vitvi14g01870.t01 | | E2F/DP | | chr2.gff3_PROT_VIT_02s0025g04440.t01 | | ERF |
| g24176.t1 | | CO-like | Vitvi17g00736.t01 | | E2F/DP | | chr2.gff3_PROT_VIT_02s0025g04460.t01 | | ERF |
| g28586.t1 | | CO-like | Vitvi18g01106.t01 | | E2F/DP | | chr2.gff3_PROT_VIT_02s0234g00130.t01 | | ERF |
| g28863.t1 | | CO-like | Vitvi06g00166.t01 | | EIL | | chr3.gff3_PROT_VIT_03s0063g00460.t01 | | ERF |
| g30008.t1 | | CO-like | Vitvi06g01036.t01 | | EIL | | chr4.gff3_PROT_VIT_04s0008g02230.t01 | | ERF |
| g30943.t1 | | CO-like | Vitvi07g01353.t01 | | EIL | | chr4.gff3_PROT_VIT_04s0008g03400.t01 | | ERF |
| g31129.t1 | | CO-like | Vitvi13g01126.t01 | | EIL | | chr4.gff3_PROT_VIT_04s0008g05440.t01 | | ERF |
| g4680.t1 | | CO-like | Vitvi00g00859.t01 | | ERF | | chr4.gff3_PROT_VIT_04s0008g06000.t01 | | ERF |
| g13253.t1 | | CPP | Vitvi01g00645.t01 | | ERF | | chr4.gff3_PROT_VIT_04s0023g00970.t01 | | ERF |
| g16749.t1 | | CPP | Vitvi01g01826.t01 | | ERF | | chr4.gff3_PROT_VIT_04s0043g00510.t01 | | ERF |
| g2172.t1 | | CPP | Vitvi01g01827.t01 | | ERF | | chr5.gff3_PROT_VIT_05s0029g00140.t01 | | ERF |
| g2172.t2 | | CPP | Vitvi02g00093.t01 | | ERF | | chr5.gff3_PROT_VIT_05s0049g00490.t01 | | ERF |
| g25312.t1 | | CPP | Vitvi02g00121.t01 | | ERF | | chr5.gff3_PROT_VIT_05s0049g00500.t01 | | ERF |
| g30858.t1 | | CPP | Vitvi02g00286.t01 | | ERF | | chr5.gff3_PROT_VIT_05s0049g00510.t01 | | ERF |
| g30858.t2 | | CPP | Vitvi02g00406.t01 | | ERF | | chr5.gff3_PROT_VIT_05s0077g01860.t01 | | ERF |
| g3609.t1 | | CPP | Vitvi02g00407.t01 | | ERF | | chr6.gff3_PROT_VIT_06s0004g00490.t01 | | ERF |
| g15162.t1 | | DBB | Vitvi02g01729.t01 | | ERF | | chr6.gff3_PROT_VIT_06s0004g08190.t01 | | ERF |
| g16199.t1 | | DBB | Vitvi02g01730.t01 | | ERF | | chr6.gff3_PROT_VIT_06s0061g01390.t01 | | ERF |
| g24962.t1 | | DBB | Vitvi02g01780.t01 | | ERF | | chr6.gff3_PROT_VIT_06s0061g01400.t01 | | ERF |
| g25555.t1 | | DBB | Vitvi03g00500.t01 | | ERF | | chr7.gff3_PROT_VIT_07s0005g00820.t01 | | ERF |
| g26355.t1 | | DBB | Vitvi04g00190.t01 | | ERF | | chr7.gff3_PROT_VIT_07s0005g03190.t01 | | ERF |
| g905.t1 | | DBB | Vitvi04g00300.t01 | | ERF | | chr7.gff3_PROT_VIT_07s0005g03200.t01 | | ERF |
| g1447.t1 | | Dof | Vitvi04g00479.t01 | | ERF | | chr7.gff3_PROT_VIT_07s0005g03210.t01 | | ERF |
| g14756.t1 | | Dof | Vitvi04g00533.t01 | | ERF | | chr7.gff3_PROT_VIT_07s0005g03220.t01 | | ERF |
| g14774.t1 | | Dof | Vitvi04g00994.t01 | | ERF | | chr7.gff3_PROT_VIT_07s0005g03230.t01 | | ERF |
| g14939.t1 | | Dof | Vitvi04g01765.t01 | | ERF | | chr7.gff3_PROT_VIT_07s0005g03260.t01 | | ERF |
| g16486.t1 | | Dof | Vitvi04g02088.t01 | | ERF | | chr7.gff3_PROT_VIT_07s0005g03270.t01 | | ERF |
| g1691.t1 | | Dof | Vitvi05g00334.t01 | | ERF | | chr7.gff3_PROT_VIT_07s0005g03280.t01 | | ERF |
| g20246.t1 | | Dof | Vitvi05g00715.t01 | | ERF | | chr7.gff3_PROT_VIT_07s0031g00190.t01 | | ERF |
| g20776.t1 | | Dof | Vitvi05g01073.t01 | | ERF | | chr7.gff3_PROT_VIT_07s0031g00420.t01 | | ERF |
| g22619.t1 | | Dof | Vitvi05g01722.t01 | | ERF | | chr7.gff3_PROT_VIT_07s0031g00710.t01 | | ERF |
| g24832.t1 | | Dof | Vitvi05g01723.t01 | | ERF | | chr7.gff3_PROT_VIT_07s0031g00720.t01 | | ERF |
| g26745.t1 | | Dof | Vitvi05g01724.t01 | | ERF | | chr7.gff3_PROT_VIT_07s0031g01980.t01 | | ERF |
| g26745.t2 | | Dof | Vitvi06g00052.t01 | | ERF | | chr7.gff3_PROT_VIT_07s0141g00690.t01 | | ERF |
| g27088.t1 | | Dof | Vitvi06g00810.t01 | | ERF | | chr8.gff3_PROT_VIT_08s0007g03790.t01 | | ERF |
| g2900.t1 | | Dof | Vitvi06g01411.t01 | | ERF | | chr8.gff3_PROT_VIT_08s0007g03810.t01 | | ERF |
| g29101.t1 | | Dof | Vitvi06g01414.t01 | | ERF | | chr8.gff3_PROT_VIT_08s0007g07250.t01 | | ERF |
| g29250.t1 | | Dof | Vitvi07g00357.t01 | | ERF | | chr8.gff3_PROT_VIT_08s0007g08150.t01 | | ERF |
| g29251.t1 | | Dof | Vitvi07g00590.t01 | | ERF | | chr8.gff3_PROT_VIT_08s0058g00520.t01 | | ERF |
| g29390.t1 | | Dof | Vitvi07g00591.t01 | | ERF | | chr9.gff3_PROT_VIT_09s0002g00470.t01 | | ERF |
| g29731.t1 | | Dof | Vitvi07g00592.t01 | | ERF | | chr9.gff3_PROT_VIT_09s0002g03940.t01 | | ERF |
| g31883.t1 | | Dof | Vitvi07g01702.t01 | | ERF | | chr9.gff3_PROT_VIT_09s0002g06750.t01 | | ERF |
| g32379.t1 | | Dof | Vitvi07g01755.t01 | | ERF | | chr9.gff3_PROT_VIT_09s0002g08830.t01 | | ERF |
| g35692.t1 | | Dof | Vitvi07g01874.t01 | | ERF | | chr9.gff3_PROT_VIT_09s0002g09120.t01 | | ERF |
| g3721.t1 | | Dof | Vitvi07g02062.t01 | | ERF | | chr9.gff3_PROT_VIT_09s0002g09140.t01 | | ERF |
| g702.t1 | | Dof | Vitvi07g02063.t01 | | ERF | | chrUn.gff3_PROT_VIT_00s0184g00010.t01 | | ERF |
| g16240.t1 | | E2F/DP | Vitvi07g02064.t01 | | ERF | | chrUn.gff3_PROT_VIT_00s0291g00030.t01 | | ERF |
| g17276.t1 | | E2F/DP | Vitvi07g02065.t01 | | ERF | | chrUn.gff3_PROT_VIT_00s0341g00070.t01 | | ERF |
| g24831.t1 | | E2F/DP | Vitvi07g02066.t01 | | ERF | | chrUn.gff3_PROT_VIT_00s0341g00080.t01 | | ERF |
| g26968.t1 | | E2F/DP | Vitvi07g02067.t01 | | ERF | | chrUn.gff3_PROT_VIT_00s0632g00010.t01 | | ERF |
| g26968.t2 | | E2F/DP | Vitvi07g02068.t01 | | ERF | | chrUn.gff3_PROT_VIT_00s0662g00030.t01 | | ERF |
| g2822.t1 | | E2F/DP | Vitvi07g02069.t01 | | ERF | | chrUn.gff3_PROT_VIT_00s0662g00040.t01 | | ERF |
| g29163.t1 | | E2F/DP | Vitvi07g02070.t01 | | ERF | | chr1.gff3_PROT_VIT_01s0011g01470.t01 | | FAR1 |
| g29163.t2 | | E2F/DP | Vitvi07g02662.t01 | | ERF | | chr12.gff3_PROT_VIT_12s0035g01360.t01 | | FAR1 |
| g958.t1 | | E2F/DP | Vitvi07g03029.t01 | | ERF | | chr12.gff3_PROT_VIT_12s0057g00960.t01 | | FAR1 |
| g958.t2 | | E2F/DP | Vitvi07g03030.t01 | | ERF | | chr13.gff3_PROT_VIT_13s0047g00330.t01 | | FAR1 |
| g14530.t1 | | EIL | Vitvi08g00778.t01 | | ERF | | chr14.gff3_PROT_VIT_14s0068g00770.t01 | | FAR1 |
| g14530.t2 | | EIL | Vitvi08g01501.t01 | | ERF | | chr14.gff3_PROT_VIT_14s0108g01540.t01 | | FAR1 |
| g18867.t1 | | EIL | Vitvi08g01861.t01 | | ERF | | chr18.gff3_PROT_VIT_18s0117g00140.t01 | | FAR1 |
| g33271.t1 | | EIL | Vitvi08g01965.t01 | | ERF | | chr18.gff3_PROT_VIT_18s0117g00390.t01 | | FAR1 |
| g33975.t1 | | EIL | Vitvi08g01966.t01 | | ERF | | chr18.gff3_PROT_VIT_18s0122g00530.t01 | | FAR1 |
| g11745.t1 | | ERF | Vitvi09g00031.t01 | | ERF | | chr18_random.gff3_PROT_VIT_18s0001g06550.t01 | | FAR1 |
| g11885.t1 | | ERF | Vitvi09g00323.t01 | | ERF | | chr18_random.gff3_PROT_VIT_18s0001g06680.t01 | | FAR1 |
| g11887.t1 | | ERF | Vitvi09g00593.t01 | | ERF | | chr3.gff3_PROT_VIT_03s0017g01780.t01 | | FAR1 |
| g12995.t1 | | ERF | Vitvi09g00803.t01 | | ERF | | chr3.gff3_PROT_VIT_03s0017g01810.t01 | | FAR1 |
| g13650.t1 | | ERF | Vitvi09g00834.t01 | | ERF | | chr3.gff3_PROT_VIT_03s0017g01830.t01 | | FAR1 |
| g14258.t1 | | ERF | Vitvi09g00837.t01 | | ERF | | chr3.gff3_PROT_VIT_03s0038g04140.t01 | | FAR1 |
| g15357.t1 | | ERF | Vitvi10g00488.t01 | | ERF | | chr4.gff3_PROT_VIT_04s0044g01920.t01 | | FAR1 |
| g15575.t1 | | ERF | Vitvi10g00521.t01 | | ERF | | chr5.gff3_PROT_VIT_05s0020g02450.t01 | | FAR1 |
| g15575.t2 | | ERF | Vitvi10g00522.t01 | | ERF | | chr5.gff3_PROT_VIT_05s0077g01400.t01 | | FAR1 |
| g15854.t1 | | ERF | Vitvi10g01594.t01 | | ERF | | chr5.gff3_PROT_VIT_05s0102g00390.t01 | | FAR1 |
| g164.t1 | | ERF | Vitvi11g00045.t01 | | ERF | | chr5.gff3_PROT_VIT_05s0102g00400.t01 | | FAR1 |
| g16423.t1 | | ERF | Vitvi11g00046.t01 | | ERF | | chr5.gff3_PROT_VIT_05s0102g00410.t01 | | FAR1 |
| g17409.t1 | | ERF | Vitvi11g00285.t01 | | ERF | | chr5.gff3_PROT_VIT_05s0102g00440.t01 | | FAR1 |
| g17411.t1 | | ERF | Vitvi11g00467.t01 | | ERF | | chr5.gff3_PROT_VIT_05s0102g00470.t01 | | FAR1 |
| g17731.t1 | | ERF | Vitvi12g00274.t01 | | ERF | | chr5.gff3_PROT_VIT_05s0102g00690.t01 | | FAR1 |
| g18077.t1 | | ERF | Vitvi12g00348.t01 | | ERF | | chr7.gff3_PROT_VIT_07s0005g01120.t01 | | FAR1 |
| g19103.t1 | | ERF | Vitvi12g00451.t01 | | ERF | | chr8.gff3_PROT_VIT_08s0007g06410.t01 | | FAR1 |
| g19104.t1 | | ERF | Vitvi13g00080.t01 | | ERF | | chr8.gff3_PROT_VIT_08s0040g00200.t01 | | FAR1 |
| g19105.t1 | | ERF | Vitvi13g00116.t01 | | ERF | | chr9.gff3_PROT_VIT_09s0002g07400.t01 | | FAR1 |
| g19438.t1 | | ERF | Vitvi14g00564.t01 | | ERF | | chr9.gff3_PROT_VIT_09s0054g01830.t01 | | FAR1 |
| g20501.t1 | | ERF | Vitvi14g01067.t01 | | ERF | | chrUn.gff3_PROT_VIT_00s0174g00090.t01 | | FAR1 |
| g20533.t1 | | ERF | Vitvi14g01441.t01 | | ERF | | chrUn.gff3_PROT_VIT_00s0174g00120.t01 | | FAR1 |
| g22239.t1 | | ERF | Vitvi14g01458.t01 | | ERF | | chrUn.gff3_PROT_VIT_00s0174g00130.t01 | | FAR1 |
| g22462.t1 | | ERF | Vitvi14g02001.t01 | | ERF | | chrUn.gff3_PROT_VIT_00s0174g00140.t01 | | FAR1 |
| g22771.t1 | | ERF | Vitvi14g02049.t01 | | ERF | | chrUn.gff3_PROT_VIT_00s0194g00200.t01 | | FAR1 |
| g23495.t1 | | ERF | Vitvi15g00601.t01 | | ERF | | chrUn.gff3_PROT_VIT_00s0269g00010.t01 | | FAR1 |
| g249.t1 | | ERF | Vitvi15g00826.t01 | | ERF | | chrUn.gff3_PROT_VIT_00s0269g00040.t01 | | FAR1 |
| g250.t1 | | ERF | Vitvi15g00947.t01 | | ERF | | chr1.gff3_PROT_VIT_01s0011g03110.t01 | | G2-like |
| g25281.t1 | | ERF | Vitvi15g01021.t01 | | ERF | | chr1.gff3_PROT_VIT_01s0011g04220.t01 | | G2-like |
| g26173.t2 | | ERF | Vitvi15g01202.t01 | | ERF | | chr1.gff3_PROT_VIT_01s0026g02550.t01 | | G2-like |
| g26530.t1 | | ERF | Vitvi15g01203.t01 | | ERF | | chr1.gff3_PROT_VIT_01s0137g00050.t01 | | G2-like |
| g26762.t1 | | ERF | Vitvi15g01204.t01 | | ERF | | chr10.gff3_PROT_VIT_10s0003g01380.t01 | | G2-like |
| g27334.t1 | | ERF | Vitvi15g01206.t01 | | ERF | | chr10.gff3_PROT_VIT_10s0116g01590.t01 | | G2-like |
| g27335.t1 | | ERF | Vitvi16g00349.t01 | | ERF | | chr11.gff3_PROT_VIT_11s0016g04600.t01 | | G2-like |
| g27664.t1 | | ERF | Vitvi16g00350.t01 | | ERF | | chr12.gff3_PROT_VIT_12s0028g00980.t01 | | G2-like |
| g27795.t1 | | ERF | Vitvi16g00360.t01 | | ERF | | chr12.gff3_PROT_VIT_12s0028g02080.t01 | | G2-like |
| g29239.t1 | | ERF | Vitvi16g00362.t01 | | ERF | | chr12.gff3_PROT_VIT_12s0028g03100.t01 | | G2-like |
| g29241.t1 | | ERF | Vitvi16g00363.t01 | | ERF | | chr13.gff3_PROT_VIT_13s0067g01500.t01 | | G2-like |
| g29850.t2 | | ERF | Vitvi16g00370.t01 | | ERF | | chr13.gff3_PROT_VIT_13s0156g00370.t01 | | G2-like |
| g30040.t1 | | ERF | Vitvi16g00380.t01 | | ERF | | chr13.gff3_PROT_VIT_13s0156g00390.t01 | | G2-like |
| g32108.t1 | | ERF | Vitvi16g00941.t01 | | ERF | | chr14.gff3_PROT_VIT_14s0036g01380.t01 | | G2-like |
| g32111.t1 | | ERF | Vitvi16g00942.t01 | | ERF | | chr16.gff3_PROT_VIT_16s0039g01900.t01 | | G2-like |
| g32255.t1 | | ERF | Vitvi16g01201.t01 | | ERF | | chr16.gff3_PROT_VIT_16s0100g00430.t01 | | G2-like |
| g32973.t1 | | ERF | Vitvi16g01423.t01 | | ERF | | chr18.gff3_PROT_VIT_18s0001g11390.t01 | | G2-like |
| g33333.t1 | | ERF | Vitvi16g01424.t01 | | ERF | | chr19.gff3_PROT_VIT_19s0085g00890.t01 | | G2-like |
| g33385.t1 | | ERF | Vitvi16g01429.t01 | | ERF | | chr2.gff3_PROT_VIT_02s0012g01940.t01 | | G2-like |
| g33989.t1 | | ERF | Vitvi16g01430.t01 | | ERF | | chr2.gff3_PROT_VIT_02s0033g00300.t01 | | G2-like |
| g3458.t1 | | ERF | Vitvi16g01432.t01 | | ERF | | chr3.gff3_PROT_VIT_03s0017g02280.t01 | | G2-like |
| g35196.t1 | | ERF | Vitvi16g01434.t01 | | ERF | | chr3.gff3_PROT_VIT_03s0063g01060.t01 | | G2-like |
| g35201.t1 | | ERF | Vitvi16g01435.t01 | | ERF | | chr4.gff3_PROT_VIT_04s0043g00340.t01 | | G2-like |
| g35575.t1 | | ERF | Vitvi16g01437.t01 | | ERF | | chr5.gff3_PROT_VIT_05s0029g00060.t01 | | G2-like |
| g3575.t1 | | ERF | Vitvi16g01438.t01 | | ERF | | chr6.gff3_PROT_VIT_06s0004g00150.t01 | | G2-like |
| g36103.t1 | | ERF | Vitvi16g01442.t01 | | ERF | | chr6.gff3_PROT_VIT_06s0004g05120.t01 | | G2-like |
| g36104.t1 | | ERF | Vitvi16g01443.t01 | | ERF | | chr6.gff3_PROT_VIT_06s0004g07000.t01 | | G2-like |
| g36825.t1 | | ERF | Vitvi16g01444.t01 | | ERF | | chr7.gff3_PROT_VIT_07s0005g04120.t01 | | G2-like |
| g4444.t1 | | ERF | Vitvi17g00025.t01 | | ERF | | chr7.gff3_PROT_VIT_07s0197g00060.t01 | | G2-like |
| g11558.t1 | | FAR1 | Vitvi17g00329.t01 | | ERF | | chr8.gff3_PROT_VIT_08s0007g06180.t01 | | G2-like |
| g11560.t1 | | FAR1 | Vitvi17g00406.t01 | | ERF | | chr8.gff3_PROT_VIT_08s0007g06310.t01 | | G2-like |
| g14132.t1 | | FAR1 | Vitvi17g00769.t01 | | ERF | | chr8.gff3_PROT_VIT_08s0007g07580.t01 | | G2-like |
| g14330.t1 | | FAR1 | Vitvi18g00295.t01 | | ERF | | chr8.gff3_PROT_VIT_08s0040g00100.t01 | | G2-like |
| g14332.t1 | | FAR1 | Vitvi18g00381.t01 | | ERF | | chr8.gff3_PROT_VIT_08s0040g00900.t01 | | G2-like |
| g14333.t1 | | FAR1 | Vitvi18g01029.t01 | | ERF | | chr8.gff3_PROT_VIT_08s0058g00250.t01 | | G2-like |
| g14334.t1 | | FAR1 | Vitvi18g01617.t01 | | ERF | | chr8.gff3_PROT_VIT_08s0105g00370.t01 | | G2-like |
| g16619.t1 | | FAR1 | Vitvi18g02191.t01 | | ERF | | chr9.gff3_PROT_VIT_09s0054g01620.t01 | | G2-like |
| g16624.t1 | | FAR1 | Vitvi18g02240.t01 | | ERF | | chr9.gff3_PROT_VIT_09s0070g00410.t01 | | G2-like |
| g16626.t1 | | FAR1 | Vitvi18g02241.t01 | | ERF | | chrUn.gff3_PROT_VIT_00s0475g00030.t01 | | G2-like |
| g16626.t2 | | FAR1 | Vitvi18g02398.t01 | | ERF | | chrUn.gff3_PROT_VIT_00s0475g00040.t01 | | G2-like |
| g17654.t1 | | FAR1 | Vitvi18g02437.t01 | | ERF | | chrUn.gff3_PROT_VIT_00s1624g00010.t01 | | G2-like |
| g18683.t1 | | FAR1 | Vitvi19g00260.t01 | | ERF | | chr11.gff3_PROT_VIT_11s0016g02210.t01 | | GATA |
| g22069.t1 | | FAR1 | Vitvi19g00558.t01 | | ERF | | chr12.gff3_PROT_VIT_12s0121g00120.t01 | | GATA |
| g22094.t1 | | FAR1 | Vitvi19g01784.t01 | | ERF | | chr13.gff3_PROT_VIT_13s0019g04390.t01 | | GATA |
| g22517.t1 | | FAR1 | Vitvi00g02057.t01 | | FAR1 | | chr14.gff3_PROT_VIT_14s0060g01520.t01 | | GATA |
| g22801.t1 | | FAR1 | Vitvi01g00116.t01 | | FAR1 | | chr14.gff3_PROT_VIT_14s0066g00150.t01 | | GATA |
| g23073.t1 | | FAR1 | Vitvi02g01591.t01 | | FAR1 | | chr15.gff3_PROT_VIT_15s0021g02510.t01 | | GATA |
| g23165.t1 | | FAR1 | Vitvi03g01239.t01 | | FAR1 | | chr18.gff3_PROT_VIT_18s0001g07720.t01 | | GATA |
| g25893.t1 | | FAR1 | Vitvi03g01243.t01 | | FAR1 | | chr18.gff3_PROT_VIT_18s0001g07730.t01 | | GATA |
| g25893.t2 | | FAR1 | Vitvi03g01244.t01 | | FAR1 | | chr3.gff3_PROT_VIT_03s0038g00490.t01 | | GATA |
| g26322.t1 | | FAR1 | Vitvi03g01307.t01 | | FAR1 | | chr3_random.gff3_PROT_VIT_03s0038g00480.t01 | | GATA |
| g26325.t1 | | FAR1 | Vitvi03g01329.t01 | | FAR1 | | chr4.gff3_PROT_VIT_04s0008g01290.t01 | | GATA |
| g26326.t1 | | FAR1 | Vitvi03g01475.t01 | | FAR1 | | chr4.gff3_PROT_VIT_04s0008g03270.t01 | | GATA |
| g26362.t1 | | FAR1 | Vitvi03g01688.t01 | | FAR1 | | chr4.gff3_PROT_VIT_04s0023g01840.t01 | | GATA |
| g26373.t1 | | FAR1 | Vitvi03g01735.t01 | | FAR1 | | chr4.gff3_PROT_VIT_04s0023g02880.t01 | | GATA |
| g26382.t1 | | FAR1 | Vitvi03g01744.t01 | | FAR1 | | chr5.gff3_PROT_VIT_05s0051g00450.t01 | | GATA |
| g26391.t1 | | FAR1 | Vitvi04g00504.t01 | | FAR1 | | chr5.gff3_PROT_VIT_05s0077g01450.t01 | | GATA |
| g26393.t1 | | FAR1 | Vitvi04g01750.t01 | | FAR1 | | chr6.gff3_PROT_VIT_06s0004g02740.t01 | | GATA |
| g26395.t1 | | FAR1 | Vitvi05g00081.t01 | | FAR1 | | chr8.gff3_PROT_VIT_08s0007g07550.t01 | | GATA |
| g26396.t1 | | FAR1 | Vitvi05g01496.t01 | | FAR1 | | chr9.gff3_PROT_VIT_09s0002g03750.t01 | | GATA |
| g27147.t1 | | FAR1 | Vitvi05g01497.t01 | | FAR1 | | chr9.gff3_PROT_VIT_09s0054g00440.t01 | | GATA |
| g27455.t1 | | FAR1 | Vitvi05g01498.t01 | | FAR1 | | chr12.gff3_PROT_VIT_12s0059g02690.t01 | | GeBP |
| g27455.t2 | | FAR1 | Vitvi05g01499.t01 | | FAR1 | | chr19.gff3_PROT_VIT_19s0014g05030.t01 | | GeBP |
| g27746.t1 | | FAR1 | Vitvi05g01506.t01 | | FAR1 | | chrUn.gff3_PROT_VIT_00s0868g00010.t01 | | GeBP |
| g28017.t1 | | FAR1 | Vitvi05g01510.t01 | | FAR1 | | chr1.gff3_PROT_VIT_01s0010g02270.t01 | | GRAS |
| g29877.t1 | | FAR1 | Vitvi05g01512.t01 | | FAR1 | | chr1.gff3_PROT_VIT_01s0011g05260.t01 | | GRAS |
| g30877.t1 | | FAR1 | Vitvi05g01873.t01 | | FAR1 | | chr11.gff3_PROT_VIT_11s0016g04630.t01 | | GRAS |
| g32611.t1 | | FAR1 | Vitvi05g02179.t01 | | FAR1 | | chr12.gff3_PROT_VIT_12s0057g00560.t01 | | GRAS |
| g33988.t1 | | FAR1 | Vitvi05g02274.t01 | | FAR1 | | chr12.gff3_PROT_VIT_12s0059g02770.t01 | | GRAS |
| g34115.t1 | | FAR1 | Vitvi06g01048.t01 | | FAR1 | | chr13.gff3_PROT_VIT_13s0019g01700.t01 | | GRAS |
| g34115.t2 | | FAR1 | Vitvi06g01874.t01 | | FAR1 | | chr13.gff3_PROT_VIT_13s0019g01710.t01 | | GRAS |
| g34425.t1 | | FAR1 | Vitvi06g01900.t01 | | FAR1 | | chr13.gff3_PROT_VIT_13s0019g01780.t01 | | GRAS |
| g35204.t1 | | FAR1 | Vitvi07g00384.t01 | | FAR1 | | chr13.gff3_PROT_VIT_13s0019g01790.t01 | | GRAS |
| g36818.t1 | | FAR1 | Vitvi07g01172.t01 | | FAR1 | | chr13.gff3_PROT_VIT_13s0019g01810.t01 | | GRAS |
| g37011.t1 | | FAR1 | Vitvi07g01196.t01 | | FAR1 | | chr13_random.gff3_PROT_VIT_13s0019g01220.t01 | | GRAS |
| g4693.t1 | | FAR1 | Vitvi07g01198.t01 | | FAR1 | | chr14.gff3_PROT_VIT_14s0006g00640.t01 | | GRAS |
| g9324.t1 | | FAR1 | Vitvi07g01199.t01 | | FAR1 | | chr14.gff3_PROT_VIT_14s0068g01610.t01 | | GRAS |
| g1181.t1 | | G2-like | Vitvi07g02480.t01 | | FAR1 | | chr15.gff3_PROT_VIT_15s0048g00270.t01 | | GRAS |
| g11889.t1 | | G2-like | Vitvi08g00891.t01 | | FAR1 | | chr16.gff3_PROT_VIT_16s0050g00950.t01 | | GRAS |
| g11891.t1 | | G2-like | Vitvi08g01733.t01 | | FAR1 | | chr17.gff3_PROT_VIT_17s0000g10300.t01 | | GRAS |
| g12976.t1 | | G2-like | Vitvi08g02078.t01 | | FAR1 | | chr18.gff3_PROT_VIT_18s0001g03310.t01 | | GRAS |
| g13427.t1 | | G2-like | Vitvi09g00649.t01 | | FAR1 | | chr18.gff3_PROT_VIT_18s0001g15270.t01 | | GRAS |
| g13656.t1 | | G2-like | Vitvi09g00650.t01 | | FAR1 | | chr18.gff3_PROT_VIT_18s0076g00200.t01 | | GRAS |
| g13656.t2 | | G2-like | Vitvi09g01480.t01 | | FAR1 | | chr19.gff3_PROT_VIT_19s0014g04940.t01 | | GRAS |
| g1383.t1 | | G2-like | Vitvi09g01677.t01 | | FAR1 | | chr19.gff3_PROT_VIT_19s0015g01930.t01 | | GRAS |
| g14062.t1 | | G2-like | Vitvi10g00230.t01 | | FAR1 | | chr19.gff3_PROT_VIT_19s0085g00540.t01 | | GRAS |
| g14139.t1 | | G2-like | Vitvi11g01669.t01 | | FAR1 | | chr19.gff3_PROT_VIT_19s0090g01740.t01 | | GRAS |
| g14139.t2 | | G2-like | Vitvi11g01700.t01 | | FAR1 | | chr2.gff3_PROT_VIT_02s0025g04000.t01 | | GRAS |
| g14736.t1 | | G2-like | Vitvi12g00713.t01 | | FAR1 | | chr2.gff3_PROT_VIT_02s0033g00050.t01 | | GRAS |
| g14736.t2 | | G2-like | Vitvi12g02073.t01 | | FAR1 | | chr2.gff3_PROT_VIT_02s0154g00400.t01 | | GRAS |
| g16052.t1 | | G2-like | Vitvi12g02453.t01 | | FAR1 | | chr3.gff3_PROT_VIT_03s0017g01660.t01 | | GRAS |
| g16356.t1 | | G2-like | Vitvi13g01139.t01 | | FAR1 | | chr4.gff3_PROT_VIT_04s0023g01380.t01 | | GRAS |
| g16356.t2 | | G2-like | Vitvi13g02116.t01 | | FAR1 | | chr4.gff3_PROT_VIT_04s0023g01660.t01 | | GRAS |
| g16499.t1 | | G2-like | Vitvi13g02304.t01 | | FAR1 | | chr4.gff3_PROT_VIT_04s0044g00620.t01 | | GRAS |
| g17632.t1 | | G2-like | Vitvi13g02479.t01 | | FAR1 | | chr4.gff3_PROT_VIT_04s0044g01370.t01 | | GRAS |
| g17643.t1 | | G2-like | Vitvi14g00948.t01 | | FAR1 | | chr5.gff3_PROT_VIT_05s0077g01120.t01 | | GRAS |
| g17643.t2 | | G2-like | Vitvi14g01432.t01 | | FAR1 | | chr6.gff3_PROT_VIT_06s0004g04950.t01 | | GRAS |
| g17761.t1 | | G2-like | Vitvi14g01622.t01 | | FAR1 | | chr6.gff3_PROT_VIT_06s0004g04960.t01 | | GRAS |
| g18569.t1 | | G2-like | Vitvi14g02030.t01 | | FAR1 | | chr6.gff3_PROT_VIT_06s0004g04970.t01 | | GRAS |
| g18905.t1 | | G2-like | Vitvi14g02715.t01 | | FAR1 | | chr6.gff3_PROT_VIT_06s0004g04980.t01 | | GRAS |
| g1969.t1 | | G2-like | Vitvi14g02853.t01 | | FAR1 | | chr6.gff3_PROT_VIT_06s0004g04990.t01 | | GRAS |
| g1969.t2 | | G2-like | Vitvi15g01428.t01 | | FAR1 | | chr6.gff3_PROT_VIT_06s0009g02390.t01 | | GRAS |
| g1970.t1 | | G2-like | Vitvi17g01317.t01 | | FAR1 | | chr7.gff3_PROT_VIT_07s0005g01500.t01 | | GRAS |
| g1970.t2 | | G2-like | Vitvi18g00039.t01 | | FAR1 | | chr7.gff3_PROT_VIT_07s0005g03700.t01 | | GRAS |
| g20268.t1 | | G2-like | Vitvi18g01884.t01 | | FAR1 | | chr7.gff3_PROT_VIT_07s0129g00030.t01 | | GRAS |
| g20268.t2 | | G2-like | Vitvi18g02193.t01 | | FAR1 | | chr7.gff3_PROT_VIT_07s0129g00340.t01 | | GRAS |
| g21488.t1 | | G2-like | Vitvi18g02891.t01 | | FAR1 | | chr8.gff3_PROT_VIT_08s0007g00510.t01 | | GRAS |
| g21489.t1 | | G2-like | Vitvi18g03049.t01 | | FAR1 | | chr8.gff3_PROT_VIT_08s0056g00050.t01 | | GRAS |
| g22620.t1 | | G2-like | Vitvi18g03185.t01 | | FAR1 | | chr8.gff3_PROT_VIT_08s0058g00310.t01 | | GRAS |
| g2276.t1 | | G2-like | Vitvi01g00249.t01 | | G2-like | | chr9.gff3_PROT_VIT_09s0002g01190.t01 | | GRAS |
| g23123.t1 | | G2-like | Vitvi01g00578.t01 | | G2-like | | chrUn.gff3_PROT_VIT_00s0226g00070.t01 | | GRAS |
| g24216.t1 | | G2-like | Vitvi01g01024.t01 | | G2-like | | chrUn.gff3_PROT_VIT_00s0463g00020.t01 | | GRAS |
| g26502.t1 | | G2-like | Vitvi02g00757.t01 | | G2-like | | chr11.gff3_PROT_VIT_11s0016g01250.t01 | | GRF |
| g27277.t1 | | G2-like | Vitvi02g01004.t01 | | G2-like | | chr16.gff3_PROT_VIT_16s0039g01450.t01 | | GRF |
| g30384.t1 | | G2-like | Vitvi03g00454.t01 | | G2-like | | chr16.gff3_PROT_VIT_16s0098g01080.t01 | | GRF |
| g30384.t2 | | G2-like | Vitvi03g01303.t01 | | G2-like | | chr18.gff3_PROT_VIT_18s0001g08650.t01 | | GRF |
| g3097.t1 | | G2-like | Vitvi04g00965.t01 | | G2-like | | chr2.gff3_PROT_VIT_02s0025g02680.t01 | | GRF |
| g3097.t2 | | G2-like | Vitvi04g02230.t01 | | G2-like | | chr2.gff3_PROT_VIT_02s0025g04910.t01 | | GRF |
| g31.t1 | | G2-like | Vitvi05g01066.t01 | | G2-like | | chr8.gff3_PROT_VIT_08s0007g03760.t01 | | GRF |
| g31466.t1 | | G2-like | Vitvi06g00024.t01 | | G2-like | | chr9.gff3_PROT_VIT_09s0002g01350.t01 | | GRF |
| g3168.t1 | | G2-like | Vitvi06g00505.t01 | | G2-like | | chrUn.gff3_PROT_VIT_00s0494g00010.t01 | | GRF |
| g31712.t1 | | G2-like | Vitvi06g00686.t01 | | G2-like | | chr10.gff3_PROT_VIT_10s0003g00290.t01 | | HB-other |
| g32460.t1 | | G2-like | Vitvi07g00666.t01 | | G2-like | | chr12.gff3_PROT_VIT_12s0028g03080.t01 | | HB-other |
| g32944.t1 | | G2-like | Vitvi07g01325.t01 | | G2-like | | chr13.gff3_PROT_VIT_13s0067g01580.t01 | | HB-other |
| g33599.t1 | | G2-like | Vitvi08g00654.t01 | | G2-like | | chr14.gff3_PROT_VIT_14s0030g02130.t01 | | HB-other |
| g33645.t1 | | G2-like | Vitvi08g00736.t01 | | G2-like | | chr17.gff3_PROT_VIT_17s0000g08980.t01 | | HB-other |
| g33645.t2 | | G2-like | Vitvi08g00955.t01 | | G2-like | | chr19.gff3_PROT_VIT_19s0014g01880.t01 | | HB-other |
| g33645.t3 | | G2-like | Vitvi08g01712.t01 | | G2-like | | chr19.gff3_PROT_VIT_19s0085g00350.t01 | | HB-other |
| g34137.t1 | | G2-like | Vitvi08g01723.t01 | | G2-like | | chr4.gff3_PROT_VIT_04s0008g05190.t01 | | HB-other |
| g35376.t1 | | G2-like | Vitvi08g01834.t01 | | G2-like | | chr6.gff3_PROT_VIT_06s0009g03530.t01 | | HB-other |
| g35376.t2 | | G2-like | Vitvi08g02099.t01 | | G2-like | | chr7.gff3_PROT_VIT_07s0104g01580.t01 | | HB-other |
| g36137.t1 | | G2-like | Vitvi09g01008.t01 | | G2-like | | chr9.gff3_PROT_VIT_09s0018g02100.t01 | | HB-other |
| g5303.t1 | | G2-like | Vitvi09g01459.t01 | | G2-like | | chr3.gff3_PROT_VIT_03s0091g00290.t01 | | HB-PHD |
| g5754.t1 | | G2-like | Vitvi10g00301.t01 | | G2-like | | chr11.gff3_PROT_VIT_11s0103g00650.t01 | | HB-PHD |
| g5754.t2 | | G2-like | Vitvi10g00302.t01 | | G2-like | | chr14.gff3_PROT_VIT_14s0066g01440.t01 | | HD-ZIP |
| g6541.t1 | | G2-like | Vitvi10g01637.t01 | | G2-like | | chr1.gff3_PROT_VIT_01s0026g01950.t01 | | HD-ZIP |
| g6541.t2 | | G2-like | Vitvi10g01793.t01 | | G2-like | | chr18.gff3_PROT_VIT_18s0001g06430.t01 | | HD-ZIP |
| g6541.t3 | | G2-like | Vitvi10g02406.t01 | | G2-like | | chr7.gff3_PROT_VIT_07s0191g00180.t01 | | HD-ZIP |
| g712.t1 | | G2-like | Vitvi11g00405.t01 | | G2-like | | chr15.gff3_PROT_VIT_15s0021g01880.t01 | | HD-ZIP |
| g8276.t1 | | G2-like | Vitvi12g00177.t01 | | G2-like | | chr13.gff3_PROT_VIT_13s0156g00260.t01 | | HD-ZIP |
| g9613.t1 | | G2-like | Vitvi12g00260.t01 | | G2-like | | chr8.gff3_PROT_VIT_08s0007g06670.t01 | | HD-ZIP |
| g9614.t1 | | G2-like | Vitvi12g02204.t01 | | G2-like | | chr14.gff3_PROT_VIT_14s0108g00390.t01 | | HD-ZIP |
| g12024.t1 | | GATA | Vitvi12g02510.t01 | | G2-like | | chr17.gff3_PROT_VIT_17s0000g05630.t01 | | HD-ZIP |
| g12452.t1 | | GATA | Vitvi13g00088.t01 | | G2-like | | chrUn.gff3_PROT_VIT_00s0299g00100.t01 | | HD-ZIP |
| g14573.t1 | | GATA | Vitvi13g02566.t01 | | G2-like | | chr1.gff3_PROT_VIT_01s0026g01550.t01 | | HD-ZIP |
| g14574.t1 | | GATA | Vitvi13g02568.t01 | | G2-like | | chrUn.gff3_PROT_VIT_00s0732g00010.t01 | | HD-ZIP |
| g15171.t1 | | GATA | Vitvi14g00736.t01 | | G2-like | | chr15.gff3_PROT_VIT_15s0048g02870.t01 | | HD-ZIP |
| g15811.t1 | | GATA | Vitvi14g01333.t01 | | G2-like | | chr16.gff3_PROT_VIT_16s0098g01170.t01 | | HD-ZIP |
| g21682.t1 | | GATA | Vitvi14g02915.t01 | | G2-like | | chr18.gff3_PROT_VIT_18s0001g08410.t01 | | HD-ZIP |
| g22177.t1 | | GATA | Vitvi16g00121.t01 | | G2-like | | chr8.gff3_PROT_VIT_08s0007g04200.t01 | | HD-ZIP |
| g22178.t1 | | GATA | Vitvi16g00773.t01 | | G2-like | | chr1.gff3_PROT_VIT_01s0011g04870.t01 | | HD-ZIP |
| g22178.t2 | | GATA | Vitvi16g00947.t01 | | G2-like | | chr4.gff3_PROT_VIT_04s0023g01330.t01 | | HD-ZIP |
| g24151.t1 | | GATA | Vitvi18g00869.t01 | | G2-like | | chr19.gff3_PROT_VIT_19s0014g02110.t01 | | HD-ZIP |
| g25567.t1 | | GATA | Vitvi19g01742.t01 | | G2-like | | chr10.gff3_PROT_VIT_10s0003g00380.t01 | | HD-ZIP |
| g25682.t1 | | GATA | Vitvi03g00037.t01 | | GATA | | chr2.gff3_PROT_VIT_02s0025g02590.t01 | | HD-ZIP |
| g27459.t1 | | GATA | Vitvi03g01002.t01 | | GATA | | chr9.gff3_PROT_VIT_09s0002g04340.t01 | | HD-ZIP |
| g27579.t1 | | GATA | Vitvi03g01766.t01 | | GATA | | chr12.gff3_PROT_VIT_12s0059g02310.t01 | | HD-ZIP |
| g31803.t1 | | GATA | Vitvi04g00111.t01 | | GATA | | chr10.gff3_PROT_VIT_10s0116g00680.t01 | | HD-ZIP |
| g3360.t1 | | GATA | Vitvi04g00289.t01 | | GATA | | chr15.gff3_PROT_VIT_15s0048g02000.t01 | | HD-ZIP |
| g33737.t1 | | GATA | Vitvi04g01299.t01 | | GATA | | chr17.gff3_PROT_VIT_17s0053g00780.t01 | | HD-ZIP |
| g3563.t1 | | GATA | Vitvi04g01410.t01 | | GATA | | chr16.gff3_PROT_VIT_16s0100g00670.t01 | | HD-ZIP |
| g36033.t1 | | GATA | Vitvi05g00077.t01 | | GATA | | chr9.gff3_PROT_VIT_09s0002g03740.t01 | | HD-ZIP |
| g36309.t1 | | GATA | Vitvi05g00938.t01 | | GATA | | chr10.gff3_PROT_VIT_10s0003g04670.t01 | | HD-ZIP |
| g36412.t1 | | GATA | Vitvi06g00271.t01 | | GATA | | chr4.gff3_PROT_VIT_04s0008g03250.t01 | | HD-ZIP |
| g36412.t2 | | GATA | Vitvi06g01610.t01 | | GATA | | chr2.gff3_PROT_VIT_02s0012g02030.t01 | | HD-ZIP |
| g36416.t1 | | GATA | Vitvi07g02214.t01 | | GATA | | chr13.gff3_PROT_VIT_13s0019g04320.t01 | | HD-ZIP |
| g37016.t1 | | GATA | Vitvi08g01831.t01 | | GATA | | chr6.gff3_PROT_VIT_06s0004g02800.t01 | | HD-ZIP |
| g38279.t1 | | GATA | Vitvi09g00311.t01 | | GATA | | chr4.gff3_PROT_VIT_04s0079g00480.t01 | | HD-ZIP |
| g9001.t1 | | GATA | Vitvi09g01352.t01 | | GATA | | chr11.gff3_PROT_VIT_11s0016g02440.t01 | | HRT-like |
| g9476.t1 | | GATA | Vitvi11g00180.t01 | | GATA | | chr1.gff3_PROT_VIT_01s0011g05970.t01 | | HSF |
| g16178.t1 | | GeBP | Vitvi12g01002.t01 | | GATA | | chr10.gff3_PROT_VIT_10s0003g01770.t01 | | HSF |
| g1925.t1 | | GeBP | Vitvi13g00614.t01 | | GATA | | chr10.gff3_PROT_VIT_10s0597g00050.t01 | | HSF |
| g25697.t1 | | GeBP | Vitvi14g00123.t01 | | GATA | | chr11.gff3_PROT_VIT_11s0016g02010.t01 | | HSF |
| g28578.t1 | | GeBP | Vitvi14g02998.t01 | | GATA | | chr11.gff3_PROT_VIT_11s0016g03940.t01 | | HSF |
| g30874.t1 | | GeBP | Vitvi15g00636.t01 | | GATA | | chr12.gff3_PROT_VIT_12s0028g01410.t01 | | HSF |
| g30874.t2 | | GeBP | Vitvi18g00537.t01 | | GATA | | chr16.gff3_PROT_VIT_16s0039g01840.t01 | | HSF |
| g4794.t1 | | GeBP | Vitvi18g00538.t01 | | GATA | | chr16.gff3_PROT_VIT_16s0100g00720.t01 | | HSF |
| g1048.t1 | | GRAS | Vitvi10g02200.t01 | | GeBP | | chr18.gff3_PROT_VIT_18s0001g10380.t01 | | HSF |
| g11217.t1 | | GRAS | Vitvi12g00561.t01 | | GeBP | | chr2.gff3_PROT_VIT_02s0012g01810.t01 | | HSF |
| g12179.t1 | | GRAS | Vitvi14g00879.t01 | | GeBP | | chr2.gff3_PROT_VIT_02s0025g04170.t01 | | HSF |
| g1397.t1 | | GRAS | Vitvi19g01979.t01 | | GeBP | | chr4.gff3_PROT_VIT_04s0008g01110.t01 | | HSF |
| g1398.t1 | | GRAS | Vitvi01g00446.t01 | | GRAS | | chr5.gff3_PROT_VIT_05s0020g04090.t01 | | HSF |
| g1398.t2 | | GRAS | Vitvi01g01509.t01 | | GRAS | | chr5.gff3_PROT_VIT_05s0029g00340.t01 | | HSF |
| g1399.t1 | | GRAS | Vitvi02g00370.t01 | | GRAS | | chr6.gff3_PROT_VIT_06s0009g02730.t01 | | HSF |
| g1400.t1 | | GRAS | Vitvi02g00536.t01 | | GRAS | | chr7.gff3_PROT_VIT_07s0031g00670.t01 | | HSF |
| g1401.t1 | | GRAS | Vitvi02g00974.t01 | | GRAS | | chr8.gff3_PROT_VIT_08s0007g03900.t01 | | HSF |
| g15906.t1 | | GRAS | Vitvi03g01226.t01 | | GRAS | | chr8.gff3_PROT_VIT_08s0007g08750.t01 | | HSF |
| g16154.t1 | | GRAS | Vitvi04g01247.t01 | | GRAS | | chrUn.gff3_PROT_VIT_00s0179g00150.t01 | | HSF |
| g16154.t2 | | GRAS | Vitvi04g01281.t01 | | GRAS | | chr1.gff3_PROT_VIT_01s0011g03530.t01 | | LBD |
| g16169.t1 | | GRAS | Vitvi04g01622.t01 | | GRAS | | chr1.gff3_PROT_VIT_01s0011g03540.t01 | | LBD |
| g16381.t1 | | GRAS | Vitvi04g01696.t01 | | GRAS | | chr13.gff3_PROT_VIT_13s0019g02060.t01 | | LBD |
| g16381.t2 | | GRAS | Vitvi05g00110.t01 | | GRAS | | chr13.gff3_PROT_VIT_13s0019g03700.t01 | | LBD |
| g1676.t1 | | GRAS | Vitvi05g01554.t01 | | GRAS | | chr13.gff3_PROT_VIT_13s0019g03710.t01 | | LBD |
| g1677.t1 | | GRAS | Vitvi06g00489.t01 | | GRAS | | chr13.gff3_PROT_VIT_13s0019g03720.t01 | | LBD |
| g1678.t1 | | GRAS | Vitvi06g00490.t01 | | GRAS | | chr13.gff3_PROT_VIT_13s0019g03750.t01 | | LBD |
| g1679.t1 | | GRAS | Vitvi06g00491.t01 | | GRAS | | chr13.gff3_PROT_VIT_13s0019g03760.t01 | | LBD |
| g1680.t1 | | GRAS | Vitvi06g00492.t01 | | GRAS | | chr13.gff3_PROT_VIT_13s0019g03780.t01 | | LBD |
| g1681.t1 | | GRAS | Vitvi06g01133.t01 | | GRAS | | chr13.gff3_PROT_VIT_13s0019g03800.t01 | | LBD |
| g18559.t1 | | GRAS | Vitvi06g01569.t01 | | GRAS | | chr13.gff3_PROT_VIT_13s0019g03810.t01 | | LBD |
| g18635.t1 | | GRAS | Vitvi07g00418.t01 | | GRAS | | chr13.gff3_PROT_VIT_13s0019g03820.t01 | | LBD |
| g19332.t1 | | GRAS | Vitvi07g00627.t01 | | GRAS | | chr13.gff3_PROT_VIT_13s0019g03830.t01 | | LBD |
| g2000.t1 | | GRAS | Vitvi07g01612.t01 | | GRAS | | chr13.gff3_PROT_VIT_13s0019g03840.t01 | | LBD |
| g20201.t1 | | GRAS | Vitvi07g02073.t01 | | GRAS | | chr13.gff3_PROT_VIT_13s0067g01450.t01 | | LBD |
| g20626.t1 | | GRAS | Vitvi08g00007.t01 | | GRAS | | chr13.gff3_PROT_VIT_13s0067g01880.t01 | | LBD |
| g20653.t1 | | GRAS | Vitvi08g00746.t01 | | GRAS | | chr13.gff3_PROT_VIT_13s0067g02380.t01 | | LBD |
| g21329.t1 | | GRAS | Vitvi08g00751.t01 | | GRAS | | chr14.gff3_PROT_VIT_14s0006g02950.t01 | | LBD |
| g21331.t1 | | GRAS | Vitvi08g01214.t01 | | GRAS | | chr14.gff3_PROT_VIT_14s0066g00680.t01 | | LBD |
| g22489.t1 | | GRAS | Vitvi08g01969.t01 | | GRAS | | chr14.gff3_PROT_VIT_14s0066g02460.t01 | | LBD |
| g2280.t1 | | GRAS | Vitvi09g01487.t01 | | GRAS | | chr15.gff3_PROT_VIT_15s0046g00230.t01 | | LBD |
| g23640.t1 | | GRAS | Vitvi10g00271.t01 | | GRAS | | chr15.gff3_PROT_VIT_15s0046g00240.t01 | | LBD |
| g23848.t1 | | GRAS | Vitvi11g00409.t01 | | GRAS | | chr15.gff3_PROT_VIT_15s0048g00830.t01 | | LBD |
| g23848.t2 | | GRAS | Vitvi12g00571.t01 | | GRAS | | chr15.gff3_PROT_VIT_15s0048g00840.t01 | | LBD |
| g24229.t1 | | GRAS | Vitvi12g00665.t01 | | GRAS | | chr16.gff3_PROT_VIT_16s0022g02050.t01 | | LBD |
| g24486.t1 | | GRAS | Vitvi13g00311.t01 | | GRAS | | chr16.gff3_PROT_VIT_16s0100g00440.t01 | | LBD |
| g24862.t1 | GRAS | | Vitvi13g00312.t01 | GRAS | | chr17.gff3_PROT_VIT_17s0000g03720.t01 | | LBD | |
| g26562.t1 | GRAS | | Vitvi13g00313.t01 | GRAS | | chr17.gff3_PROT_VIT_17s0000g05490.t01 | | LBD | |
| g28677.t1 | GRAS | | Vitvi13g00314.t01 | GRAS | | chr17.gff3_PROT_VIT_17s0000g09010.t01 | | LBD | |
| g29988.t1 | GRAS | | Vitvi13g01556.t01 | GRAS | | chr18.gff3_PROT_VIT_18s0001g09250.t01 | | LBD | |
| g30321.t1 | GRAS | | Vitvi13g01865.t01 | GRAS | | chr19.gff3_PROT_VIT_19s0027g01120.t01 | | LBD | |
| g30433.t1 | GRAS | | Vitvi14g00841.t01 | GRAS | | chr3.gff3_PROT_VIT_03s0091g00670.t01 | | LBD | |
| g30555.t1 | GRAS | | Vitvi14g01348.t01 | GRAS | | chr6.gff3_PROT_VIT_06s0004g03330.t01 | | LBD | |
| g31098.t1 | GRAS | | Vitvi14g01510.t01 | GRAS | | chr6.gff3_PROT_VIT_06s0004g03350.t01 | | LBD | |
| g31594.t1 | GRAS | | Vitvi15g00680.t01 | GRAS | | chr6.gff3_PROT_VIT_06s0004g07200.t01 | | LBD | |
| g31719.t1 | GRAS | | Vitvi16g01086.t01 | GRAS | | chr6.gff3_PROT_VIT_06s0004g07790.t01 | | LBD | |
| g31722.t1 | GRAS | | Vitvi17g01040.t01 | GRAS | | chr7.gff3_PROT_VIT_07s0005g03020.t01 | | LBD | |
| g31723.t1 | GRAS | | Vitvi18g00300.t01 | GRAS | | chr7.gff3_PROT_VIT_07s0005g03030.t01 | | LBD | |
| g33577.t1 | GRAS | | Vitvi18g01210.t01 | GRAS | | chr7.gff3_PROT_VIT_07s0129g00330.t01 | | LBD | |
| g34486.t1 | GRAS | | Vitvi18g01322.t01 | GRAS | | chr7.gff3_PROT_VIT_07s0197g00020.t01 | | LBD | |
| g34656.t1 | GRAS | | Vitvi18g01651.t01 | GRAS | | chr7.gff3_PROT_VIT_07s0197g00030.t01 | | LBD | |
| g34685.t1 | GRAS | | Vitvi19g00392.t01 | GRAS | | chr7.gff3_PROT_VIT_07s0197g00040.t01 | | LBD | |
| g36505.t1 | GRAS | | Vitvi19g00619.t01 | GRAS | | chr8.gff3_PROT_VIT_08s0056g01650.t01 | | LBD | |
| g36966.t1 | GRAS | | Vitvi19g00932.t01 | GRAS | | chrUn.gff3_PROT_VIT_00s0125g00290.t01 | | LBD | |
| g37652.t1 | GRAS | | Vitvi19g01706.t01 | GRAS | | chrUn.gff3_PROT_VIT_00s0215g00010.t01 | | LBD | |
| g37878.t1 | GRAS | | Vitvi02g00239.t01 | GRF | | chrUn.gff3_PROT_VIT_00s0340g00090.t01 | | LBD | |
| g38315.t1 | GRAS | | Vitvi02g00449.t01 | GRF | | chrUn.gff3_PROT_VIT_00s1247g00010.t01 | | LBD | |
| g4582.t1 | GRAS | | Vitvi07g01551.t01 | GRF | | chr17.gff3_PROT_VIT_17s0000g00150.t01 | | LFY | |
| g5997.t1 | GRAS | | Vitvi08g01498.t01 | GRF | | chr10.gff3_PROT_VIT_10s0092g00230.t01 | | LSD | |
| g662.t1 | GRAS | | Vitvi09g00107.t01 | GRF | | chr2.gff3_PROT_VIT_02s0012g00990.t01 | | LSD | |
| g6980.t1 | GRAS | | Vitvi11g00092.t01 | GRF | | chrUn.gff3_PROT_VIT_00s0181g00230.t01 | | LSD | |
| g7067.t1 | GRAS | | Vitvi15g00815.t01 | GRF | | chr1.gff3_PROT_VIT_01s0010g03900.t01 | | MIKC_MADS | |
| g8811.t1 | GRAS | | Vitvi16g00073.t01 | GRF | | chr1.gff3_PROT_VIT_01s0011g00100.t01 | | MIKC_MADS | |
| g8814.t1 | GRAS | | Vitvi16g01354.t01 | GRF | | chr1.gff3_PROT_VIT_01s0011g00110.t01 | | MIKC_MADS | |
| g127.t1 | GRF | | Vitvi18g00623.t01 | GRF | | chr1.gff3_PROT_VIT_01s0011g01560.t01 | | MIKC_MADS | |
| g127.t2 | GRF | | Vitvi04g00463.t01 | HB-other | | chr10.gff3_PROT_VIT_10s0003g02070.t01 | | MIKC_MADS | |
| g17406.t1 | GRF | | Vitvi06g01244.t01 | HB-other | | chr10.gff3_PROT_VIT_10s0042g00820.t01 | | MIKC_MADS | |
| g17936.t1 | GRF | | Vitvi07g00267.t01 | HB-other | | chr12.gff3_PROT_VIT_12s0142g00360.t01 | | MIKC_MADS | |
| g20683.t1 | GRF | | Vitvi09g01924.t01 | HB-other | | chr13.gff3_PROT_VIT_13s0158g00100.t01 | | MIKC_MADS | |
| g21197.t1 | GRF | | Vitvi10g00497.t01 | HB-other | | chr14.gff3_PROT_VIT_14s0083g01030.t01 | | MIKC_MADS | |
| g22296.t1 | GRF | | Vitvi12g00258.t01 | HB-other | | chr14.gff3_PROT_VIT_14s0083g01050.t01 | | MIKC_MADS | |
| g29422.t1 | GRF | | Vitvi13g01895.t01 | HB-other | | chr15.gff3_PROT_VIT_15s0048g01270.t01 | | MIKC_MADS | |
| g296.t1 | GRF | | Vitvi14g00465.t01 | HB-other | | chr15.gff3_PROT_VIT_15s0107g00120.t01 | | MIKC_MADS | |
| g29838.t1 | GRF | | Vitvi17g00888.t01 | HB-other | | chr16.gff3_PROT_VIT_16s0022g02330.t01 | | MIKC_MADS | |
| g31085.t1 | GRF | | Vitvi19g00157.t01 | HB-other | | chr17.gff3_PROT_VIT_17s0000g01230.t01 | | MIKC_MADS | |
| g12975.t1 | HB-other | | Vitvi19g01688.t01 | HB-other | | chr17.gff3_PROT_VIT_17s0000g04990.t01 | | MIKC_MADS | |
| g1479.t1 | HB-other | | Vitvi03g01600.t01 | HB-PHD | | chr17.gff3_PROT_VIT_17s0000g05000.t01 | | MIKC_MADS | |
| g15390.t1 | HB-other | | Vitvi11g01140.t01 | HB-PHD | | chr18.gff3_PROT_VIT_18s0001g01760.t01 | | MIKC_MADS | |
| g16895.t1 | HB-other | | Vitvi14g01786.t01 | HD-ZIP | | chr18.gff3_PROT_VIT_18s0001g07460.t01 | | MIKC_MADS | |
| g25304.t1 | HB-other | | Vitvi01g00958.t01 | HD-ZIP | | chr18.gff3_PROT_VIT_18s0001g07900.t01 | | MIKC_MADS | |
| g26895.t1 | HB-other | | Vitvi18g00448.t01 | HD-ZIP | | chr18.gff3_PROT_VIT_18s0001g09540.t01 | | MIKC_MADS | |
| g28414.t1 | HB-other | | Vitvi07g01488.t01 | HD-ZIP | | chr18.gff3_PROT_VIT_18s0001g13460.t01 | | MIKC_MADS | |
| g28510.t1 | HB-other | | Vitvi15g00579.t01 | HD-ZIP | | chr18.gff3_PROT_VIT_18s0041g01880.t01 | | MIKC_MADS | |
| g28950.t1 | HB-other | | Vitvi13g01823.t01 | HD-ZIP | | chr18.gff3_PROT_VIT_18s0041g02140.t01 | | MIKC_MADS | |
| g29223.t1 | HB-other | | Vitvi08g01752.t01 | HD-ZIP | | chr2.gff3_PROT_VIT_02s0025g02350.t01 | | MIKC_MADS | |
| g36242.t1 | HB-other | | Vitvi14g01922.t01 | HD-ZIP | | chr2.gff3_PROT_VIT_02s0025g04650.t01 | | MIKC_MADS | |
| g18244.t1 | HB-PHD | | Vitvi17g00540.t01 | HD-ZIP | | chr4.gff3_PROT_VIT_04s0023g02820.t01 | | MIKC_MADS | |
| g21397.t1 | HB-PHD | | Vitvi02g01717.t01 | HD-ZIP | | chr7.gff3_PROT_VIT_07s0031g01140.t01 | | MIKC_MADS | |
| g24028.t1 | HD-ZIP | | Vitvi01g00918.t01 | HD-ZIP | | chr8.gff3_PROT_VIT_08s0007g08790.t01 | | MIKC_MADS | |
| g21497.t1 | HD-ZIP | | Vitvi07g03056.t01 | HD-ZIP | | chrUn.gff3_PROT_VIT_00s0211g00180.t01 | | MIKC_MADS | |
| g23076.t1 | HD-ZIP | | Vitvi15g00912.t01 | HD-ZIP | | chrUn.gff3_PROT_VIT_00s0313g00070.t01 | | MIKC_MADS | |
| g16970.t1 | HD-ZIP | | Vitvi16g01362.t01 | HD-ZIP | | chr1.gff3_PROT_VIT_01s0010g01500.t01 | | M-type_MADS | |
| g17678.t1 | HD-ZIP | | Vitvi18g00603.t01 | HD-ZIP | | chr1.gff3_PROT_VIT_01s0010g01530.t01 | | M-type_MADS | |
| g18850.t1 | HD-ZIP | | Vitvi08g01543.t01 | HD-ZIP | | chr10.gff3_PROT_VIT_10s0003g03970.t01 | | M-type_MADS | |
| g195.t1 | HD-ZIP | | Vitvi01g00412.t01 | HD-ZIP | | chr14.gff3_PROT_VIT_14s0060g00300.t01 | | M-type_MADS | |
| g27038.t1 | HD-ZIP | | Vitvi04g01244.t01 | HD-ZIP | | chr14.gff3_PROT_VIT_14s0068g01800.t01 | | M-type_MADS | |
| g15863.t1 | HD-ZIP | | Vitvi19g01855.t01 | HD-ZIP | | chr15.gff3_PROT_VIT_15s0021g00560.t01 | | M-type_MADS | |
| g3657.t1 | HD-ZIP | | Vitvi10g01773.t01 | HD-ZIP | | chr15.gff3_PROT_VIT_15s0021g02220.t01 | | M-type_MADS | |
| g32621.t1 | HD-ZIP | | Vitvi02g00228.t01 | HD-ZIP | | chr15.gff3_PROT_VIT_15s0021g02250.t01 | | M-type_MADS | |
| g20704.t2 | HD-ZIP | | Vitvi12g00253.t01 | HD-ZIP | | chr15.gff3_PROT_VIT_15s0024g01860.t01 | | M-type_MADS | |
| g26714.t1 | HD-ZIP | | Vitvi09g00363.t01 | HD-ZIP | | chr15.gff3_PROT_VIT_15s0024g02000.t01 | | M-type_MADS | |
| g17915.t1 | HD-ZIP | | Vitvi12g00522.t01 | HD-ZIP | | chr15.gff3_PROT_VIT_15s0048g01240.t01 | | M-type_MADS | |
| g17456.t2 | HD-ZIP | | Vitvi10g00033.t01 | HD-ZIP | | chr16.gff3_PROT_VIT_16s0022g02400.t01 | | M-type_MADS | |
| g17456.t1 | HD-ZIP | | Vitvi15g00839.t01 | HD-ZIP | | chr17.gff3_PROT_VIT_17s0000g06340.t01 | | M-type_MADS | |
| g20704.t1 | HD-ZIP | | Vitvi17g01232.t01 | HD-ZIP | | chr18.gff3_PROT_VIT_18s0001g04810.t01 | | M-type_MADS | |
| g24523.t1 | HD-ZIP | | Vitvi16g00974.t01 | HD-ZIP | | chr19.gff3_PROT_VIT_19s0014g00310.t01 | | M-type_MADS | |
| g29412.t1 | HD-ZIP | | Vitvi09g00310.t01 | HD-ZIP | | chr3.gff3_PROT_VIT_03s0017g00360.t01 | | M-type_MADS | |
| g15369.t1 | HD-ZIP | | Vitvi10g00913.t01 | HD-ZIP | | chr3.gff3_PROT_VIT_03s0017g00440.t01 | | M-type_MADS | |
| g21187.t1 | HD-ZIP | | Vitvi04g00287.t01 | HD-ZIP | | chr3.gff3_PROT_VIT_03s0017g00450.t01 | | M-type_MADS | |
| g29216.t1 | HD-ZIP | | Vitvi02g00772.t01 | HD-ZIP | | chr3.gff3_PROT_VIT_03s0088g00510.t01 | | M-type_MADS | |
| g34689.t1 | HD-ZIP | | Vitvi13g00609.t01 | HD-ZIP | | chr3.gff3_PROT_VIT_03s0088g00550.t01 | | M-type_MADS | |
| g22418.t1 | HD-ZIP | | Vitvi06g00276.t01 | HD-ZIP | | chr3.gff3_PROT_VIT_03s0088g00590.t01 | | M-type_MADS | |
| g23573.t3 | HD-ZIP | | Vitvi04g00854.t01 | HD-ZIP | | chr3.gff3_PROT_VIT_03s0088g00600.t01 | | M-type_MADS | |
| g23573.t1 | HD-ZIP | | Vitvi11g00200.t01 | HRT-like | | chr3.gff3_PROT_VIT_03s0088g00610.t01 | | M-type_MADS | |
| g23573.t2 | HD-ZIP | | Vitvi01g00511.t01 | HSF | | chr3.gff3_PROT_VIT_03s0097g00160.t01 | | M-type_MADS | |
| g31396.t1 | HD-ZIP | | Vitvi02g00387.t01 | HSF | | chr3.gff3_PROT_VIT_03s0167g00100.t01 | | M-type_MADS | |
| g26122.t1 | HD-ZIP | | Vitvi02g00739.t01 | HSF | | chr4.gff3_PROT_VIT_04s0008g01980.t01 | | M-type_MADS | |
| g26122.t2 | HD-ZIP | | Vitvi04g00092.t01 | HSF | | chr7.gff3_PROT_VIT_07s0005g06590.t01 | | M-type_MADS | |
| g28818.t1 | HD-ZIP | | Vitvi05g00561.t01 | HSF | | chr7.gff3_PROT_VIT_07s0129g00650.t01 | | M-type_MADS | |
| g25111.t1 | HD-ZIP | | Vitvi05g01097.t01 | HSF | | chrUn.gff3_PROT_VIT_00s0211g00150.t01 | | M-type_MADS | |
| g21681.t1 | HD-ZIP | | Vitvi06g01878.t01 | HSF | | chrUn.gff3_PROT_VIT_00s0386g00010.t01 | | M-type_MADS | |
| g22866.t1 | HD-ZIP | | Vitvi07g00078.t01 | HSF | | chrUn.gff3_PROT_VIT_00s0729g00010.t01 | | M-type_MADS | |
| g3561.t1 | HD-ZIP | | Vitvi07g01749.t01 | HSF | | chr1.gff3_PROT_VIT_01s0010g00410.t01 | | MYB | |
| g35244.t1 | HD-ZIP | | Vitvi08g01513.t01 | HSF | | chr1.gff3_PROT_VIT_01s0011g01190.t01 | | MYB | |
| g12030.t1 | HD-ZIP | | Vitvi08g01931.t01 | HSF | | chr1.gff3_PROT_VIT_01s0011g01200.t01 | | MYB | |
| g31811.t1 | HD-ZIP | | Vitvi10g00469.t01 | HSF | | chr1.gff3_PROT_VIT_01s0011g03730.t01 | | MYB | |
| g12246.t1 | HD-ZIP | | Vitvi10g00635.t01 | HSF | | chr1.gff3_PROT_VIT_01s0011g04760.t01 | | MYB | |
| g38257.t1 | HRT-like | | Vitvi11g00159.t01 | HSF | | chr1.gff3_PROT_VIT_01s0026g01910.t01 | | MYB | |
| g13999.t1 | HSF | | Vitvi11g00339.t01 | HSF | | chr1.gff3_PROT_VIT_01s0026g02600.t01 | | MYB | |
| g16403.t1 | HSF | | Vitvi12g00121.t01 | HSF | | chr1.gff3_PROT_VIT_01s0026g02770.t01 | | MYB | |
| g16542.t1 | HSF | | Vitvi16g00114.t01 | HSF | | chr1.gff3_PROT_VIT_01s0127g00730.t01 | | MYB | |
| g16767.t1 | HSF | | Vitvi16g00982.t01 | HSF | | chr10.gff3_PROT_VIT_10s0071g00660.t01 | | MYB | |
| g17422.t1 | HSF | | Vitvi18g00777.t01 | HSF | | chr11.gff3_PROT_VIT_11s0016g01300.t01 | | MYB | |
| g18097.t1 | HSF | | Vitvi00g01060.t01 | LBD | | chr11.gff3_PROT_VIT_11s0016g01310.t01 | | MYB | |
| g19599.t1 | HSF | | Vitvi01g00290.t01 | LBD | | chr11.gff3_PROT_VIT_11s0016g01320.t01 | | MYB | |
| g19599.t2 | HSF | | Vitvi01g00291.t01 | LBD | | chr11.gff3_PROT_VIT_11s0016g02780.t01 | | MYB | |
| g19805.t1 | HSF | | Vitvi03g00628.t01 | LBD | | chr11.gff3_PROT_VIT_11s0016g03750.t01 | | MYB | |
| g20475.t1 | HSF | | Vitvi04g01768.t01 | LBD | | chr11.gff3_PROT_VIT_11s0016g05660.t01 | | MYB | |
| g2199.t1 | HSF | | Vitvi06g00336.t01 | LBD | | chr11.gff3_PROT_VIT_11s0016g05690.t01 | | MYB | |
| g224.t1 | HSF | | Vitvi06g00338.t01 | LBD | | chr11.gff3_PROT_VIT_11s0052g01490.t01 | | MYB | |
| g24416.t1 | HSF | | Vitvi06g00706.t01 | LBD | | chr11.gff3_PROT_VIT_11s0078g00480.t01 | | MYB | |
| g28813.t1 | HSF | | Vitvi06g00772.t01 | LBD | | chr12.gff3_PROT_VIT_12s0059g00700.t01 | | MYB | |
| g29287.t1 | HSF | | Vitvi07g00572.t01 | LBD | | chr12.gff3_PROT_VIT_12s0134g00480.t01 | | MYB | |
| g3113.t1 | HSF | | Vitvi07g00573.t01 | LBD | | chr12.gff3_PROT_VIT_12s0134g00490.t01 | | MYB | |
| g3340.t1 | HSF | | Vitvi07g01326.t01 | LBD | | chr12.gff3_PROT_VIT_12s0134g00570.t01 | | MYB | |
| g36130.t1 | HSF | | Vitvi07g01327.t01 | LBD | | chr13.gff3_PROT_VIT_13s0019g03200.t01 | | MYB | |
| g36704.t1 | HSF | | Vitvi07g01328.t01 | LBD | | chr13.gff3_PROT_VIT_13s0019g03370.t01 | | MYB | |
| g38300.t1 | HSF | | Vitvi07g01610.t01 | LBD | | chr13.gff3_PROT_VIT_13s0019g04010.t01 | | MYB | |
| g5564.t1 | HSF | | Vitvi07g02993.t01 | LBD | | chr13.gff3_PROT_VIT_13s0064g00960.t01 | | MYB | |
| g11487.t1 | LBD | | Vitvi08g00144.t01 | LBD | | chr13.gff3_PROT_VIT_13s0067g01360.t01 | | MYB | |
| g11488.t1 | LBD | | Vitvi09g00188.t01 | LBD | | chr13.gff3_PROT_VIT_13s0084g00530.t01 | | MYB | |
| g1154.t1 | LBD | | Vitvi10g01237.t01 | LBD | | chr13_random.gff3_PROT_VIT_13s0067g01630.t01 | | MYB | |
| g11752.t1 | LBD | | Vitvi11g00169.t01 | LBD | | chr14.gff3_PROT_VIT_14s0006g00450.t01 | | MYB | |
| g14730.t2 | LBD | | Vitvi12g00230.t01 | LBD | | chr14.gff3_PROT_VIT_14s0006g01280.t01 | | MYB | |
| g14732.t1 | LBD | | Vitvi13g00085.t01 | LBD | | chr14.gff3_PROT_VIT_14s0006g01290.t01 | | MYB | |
| g14734.t1 | LBD | | Vitvi13g00109.t01 | LBD | | chr14.gff3_PROT_VIT_14s0006g01340.t01 | | MYB | |
| g14839.t1 | LBD | | Vitvi13g00144.t01 | LBD | | chr14.gff3_PROT_VIT_14s0006g01620.t01 | | MYB | |
| g14875.t1 | LBD | | Vitvi13g00333.t01 | LBD | | chr14.gff3_PROT_VIT_14s0060g00240.t01 | | MYB | |
| g16006.t1 | LBD | | Vitvi13g00543.t01 | LBD | | chr14.gff3_PROT_VIT_14s0066g01010.t01 | | MYB | |
| g16007.t1 | LBD | | Vitvi13g00545.t01 | LBD | | chr14.gff3_PROT_VIT_14s0066g01090.t01 | | MYB | |
| g1654.t1 | LBD | | Vitvi13g00546.t01 | LBD | | chr14.gff3_PROT_VIT_14s0066g01220.t01 | | MYB | |
| g17060.t1 | LBD | | Vitvi13g00549.t01 | LBD | | chr14.gff3_PROT_VIT_14s0066g02180.t01 | | MYB | |
| g17061.t1 | LBD | | Vitvi13g00551.t01 | LBD | | chr14.gff3_PROT_VIT_14s0083g00120.t01 | | MYB | |
| g17062.t1 | LBD | | Vitvi13g00552.t01 | LBD | | chr14.gff3_PROT_VIT_14s0083g01060.t01 | | MYB | |
| g17992.t1 | LBD | | Vitvi13g00555.t01 | LBD | | chr14.gff3_PROT_VIT_14s0108g00830.t01 | | MYB | |
| g18175.t1 | LBD | | Vitvi13g00556.t01 | LBD | | chr14.gff3_PROT_VIT_14s0108g01010.t01 | | MYB | |
| g18278.t1 | LBD | | Vitvi13g00559.t01 | LBD | | chr14.gff3_PROT_VIT_14s0108g01080.t01 | | MYB | |
| g1904.t1 | LBD | | Vitvi13g01866.t01 | LBD | | chr14.gff3_PROT_VIT_14s0219g00050.t01 | | MYB | |
| g19201.t1 | LBD | | Vitvi13g01867.t01 | LBD | | chr15.gff3_PROT_VIT_15s0021g02040.t01 | | MYB | |
| g19527.t1 | LBD | | Vitvi14g01193.t01 | LBD | | chr15.gff3_PROT_VIT_15s0046g00170.t01 | | MYB | |
| g20627.t1 | LBD | | Vitvi14g01707.t01 | LBD | | chr15.gff3_PROT_VIT_15s0046g03190.t01 | | MYB | |
| g21903.t1 | LBD | | Vitvi14g01878.t01 | LBD | | chr15.gff3_PROT_VIT_15s0048g02120.t01 | | MYB | |
| g21904.t1 | LBD | | Vitvi15g00735.t01 | LBD | | chr16.gff3_PROT_VIT_16s0013g01560.t01 | | MYB | |
| g24103.t1 | LBD | | Vitvi15g00736.t01 | LBD | | chr16.gff3_PROT_VIT_16s0013g01570.t01 | | MYB | |
| g26499.t1 | LBD | | Vitvi15g01216.t01 | LBD | | chr16.gff3_PROT_VIT_16s0022g01210.t01 | | MYB | |
| g26521.t1 | LBD | | Vitvi15g01217.t01 | LBD | | chr16.gff3_PROT_VIT_16s0039g01710.t01 | | MYB | |
| g26753.t1 | LBD | | Vitvi16g00859.t01 | LBD | | chr16.gff3_PROT_VIT_16s0039g01740.t01 | | MYB | |
| g26754.t1 | LBD | | Vitvi16g01446.t01 | LBD | | chr16.gff3_PROT_VIT_16s0039g01750.t01 | | MYB | |
| g26979.t1 | LBD | | Vitvi17g00325.t01 | LBD | | chr16.gff3_PROT_VIT_16s0050g00050.t01 | | MYB | |
| g28417.t1 | LBD | | Vitvi17g00520.t01 | LBD | | chr16.gff3_PROT_VIT_16s0050g00070.t01 | | MYB | |
| g28417.t2 | LBD | | Vitvi17g00890.t01 | LBD | | chr17.gff3_PROT_VIT_17s0000g01890.t01 | | MYB | |
| g31875.t1 | LBD | | Vitvi18g00677.t01 | LBD | | chr17.gff3_PROT_VIT_17s0000g02650.t01 | | MYB | |
| g31876.t1 | LBD | | Vitvi19g01589.t01 | LBD | | chr17.gff3_PROT_VIT_17s0000g02660.t01 | | MYB | |
| g32196.t1 | LBD | | Vitvi17g00021.t01 | LFY | | chr17.gff3_PROT_VIT_17s0000g02730.t01 | | MYB | |
| g3231.t1 | LBD | | Vitvi02g00666.t01 | LSD | | chr17.gff3_PROT_VIT_17s0000g03560.t01 | | MYB | |
| g32479.t1 | LBD | | Vitvi10g00381.t01 | LSD | | chr17.gff3_PROT_VIT_17s0000g04130.t01 | | MYB | |
| g3352.t1 | LBD | | Vitvi10g01122.t01 | LSD | | chr17.gff3_PROT_VIT_17s0000g05400.t01 | | MYB | |
| g35636.t1 | LBD | | Vitvi01g00008.t01 | MIKC_MADS | | chr17.gff3_PROT_VIT_17s0000g06190.t01 | | MYB | |
| g36020.t1 | LBD | | Vitvi01g00011.t01 | MIKC_MADS | | chr17.gff3_PROT_VIT_17s0000g06410.t01 | | MYB | |
| g36201.t1 | LBD | | Vitvi01g00126.t01 | MIKC_MADS | | chr17.gff3_PROT_VIT_17s0000g08480.t01 | | MYB | |
| g36202.t1 | LBD | | Vitvi01g01673.t01 | MIKC_MADS | | chr17.gff3_PROT_VIT_17s0000g08550.t01 | | MYB | |
| g36203.t1 | LBD | | Vitvi01g01677.t01 | MIKC_MADS | | chr17.gff3_PROT_VIT_17s0000g09080.t01 | | MYB | |
| g36205.t1 | LBD | | Vitvi01g01885.t01 | MIKC_MADS | | chr18.gff3_PROT_VIT_18s0001g05670.t01 | | MYB | |
| g36206.t1 | LBD | | Vitvi02g00206.t01 | MIKC_MADS | | chr18.gff3_PROT_VIT_18s0001g08470.t01 | | MYB | |
| g38289.t1 | LBD | | Vitvi02g00427.t01 | MIKC_MADS | | chr18.gff3_PROT_VIT_18s0001g09850.t01 | | MYB | |
| g5394.t1 | LBD | | Vitvi02g01567.t01 | MIKC_MADS | | chr18.gff3_PROT_VIT_18s0001g11170.t01 | | MYB | |
| g9041.t1 | LBD | | Vitvi03g00819.t01 | MIKC_MADS | | chr18.gff3_PROT_VIT_18s0001g15260.t01 | | MYB | |
| g33429.t1 | LFY | | Vitvi03g01059.t01 | MIKC_MADS | | chr18.gff3_PROT_VIT_18s0117g00200.t01 | | MYB | |
| g13119.t1 | LSD | | Vitvi03g01320.t01 | MIKC_MADS | | chr18.gff3_PROT_VIT_18s0117g00210.t01 | | MYB | |
| g15681.t1 | LSD | | Vitvi04g01404.t01 | MIKC_MADS | | chr18_random.gff3_PROT_VIT_18s0001g02210.t01 | | MYB | |
| g35456.t1 | LSD | | Vitvi07g01441.t01 | MIKC_MADS | | chr19.gff3_PROT_VIT_19s0014g03820.t01 | | MYB | |
| g11725.t1 | MIKC_MADS | | Vitvi07g01516.t01 | MIKC_MADS | | chr19.gff3_PROT_VIT_19s0015g01280.t01 | | MYB | |
| g14400.t1 | MIKC_MADS | | Vitvi07g01520.t01 | MIKC_MADS | | chr19.gff3_PROT_VIT_19s0085g00050.t01 | | MYB | |
| g15616.t1 | MIKC_MADS | | Vitvi07g01792.t01 | MIKC_MADS | | chr19.gff3_PROT_VIT_19s0085g00940.t01 | | MYB | |
| g16929.t1 | MIKC_MADS | | Vitvi08g01935.t01 | MIKC_MADS | | chr19.gff3_PROT_VIT_19s0090g00590.t01 | | MYB | |
| g17151.t1 | MIKC_MADS | | Vitvi10g00663.t01 | MIKC_MADS | | chr2.gff3_PROT_VIT_02s0012g01650.t01 | | MYB | |
| g17151.t2 | MIKC_MADS | | Vitvi10g01395.t01 | MIKC_MADS | | chr2.gff3_PROT_VIT_02s0025g00320.t01 | | MYB | |
| g17155.t1 | MIKC_MADS | | Vitvi12g00019.t01 | MIKC_MADS | | chr2.gff3_PROT_VIT_02s0025g04220.t01 | | MYB | |
| g18019.t1 | MIKC_MADS | | Vitvi13g01861.t01 | MIKC_MADS | | chr2.gff3_PROT_VIT_02s0033g00390.t01 | | MYB | |
| g19245.t1 | MIKC_MADS | | Vitvi14g01341.t01 | MIKC_MADS | | chr2.gff3_PROT_VIT_02s0033g00400.t01 | | MYB | |
| g19416.t1 | MIKC_MADS | | Vitvi14g01344.t01 | MIKC_MADS | | chr2.gff3_PROT_VIT_02s0033g00410.t01 | | MYB | |
| g19801.t1 | MIKC_MADS | | Vitvi14g01526.t01 | MIKC_MADS | | chr2.gff3_PROT_VIT_02s0033g00430.t01 | | MYB | |
| g19801.t2 | MIKC_MADS | | Vitvi15g00225.t01 | MIKC_MADS | | chr2.gff3_PROT_VIT_02s0033g00450.t01 | | MYB | |
| g20423.t1 | MIKC_MADS | | Vitvi15g00774.t01 | MIKC_MADS | | chr3.gff3_PROT_VIT_03s0038g02310.t01 | | MYB | |
| g20423.t2 | MIKC_MADS | | Vitvi15g00776.t01 | MIKC_MADS | | chr3.gff3_PROT_VIT_03s0180g00210.t01 | | MYB | |
| g20755.t1 | MIKC_MADS | | Vitvi16g00894.t01 | MIKC_MADS | | chr4.gff3_PROT_VIT_04s0008g00620.t01 | | MYB | |
| g21163.t1 | MIKC_MADS | | Vitvi16g00898.t01 | MIKC_MADS | | chr4.gff3_PROT_VIT_04s0008g01800.t01 | | MYB | |
| g21405.t1 | MIKC_MADS | | Vitvi17g00098.t01 | MIKC_MADS | | chr4.gff3_PROT_VIT_04s0008g01810.t01 | | MYB | |
| g21405.t2 | MIKC_MADS | | Vitvi17g00470.t01 | MIKC_MADS | | chr4.gff3_PROT_VIT_04s0008g01820.t01 | | MYB | |
| g21405.t3 | MIKC_MADS | | Vitvi17g00471.t01 | MIKC_MADS | | chr4.gff3_PROT_VIT_04s0008g01830.t01 | | MYB | |
| g22154.t1 | MIKC_MADS | | Vitvi18g00221.t01 | MIKC_MADS | | chr4.gff3_PROT_VIT_04s0008g01840.t01 | | MYB | |
| g24225.t1 | MIKC_MADS | | Vitvi18g00361.t01 | MIKC_MADS | | chr4.gff3_PROT_VIT_04s0008g01870.t01 | | MYB | |
| g24227.t1 | MIKC_MADS | | Vitvi18g00517.t01 | MIKC_MADS | | chr4.gff3_PROT_VIT_04s0008g03780.t01 | | MYB | |
| g24246.t1 | MIKC_MADS | | Vitvi18g00553.t01 | MIKC_MADS | | chr4.gff3_PROT_VIT_04s0008g03790.t01 | | MYB | |
| g24246.t2 | MIKC_MADS | | Vitvi18g00700.t01 | MIKC_MADS | | chr4.gff3_PROT_VIT_04s0023g03710.t01 | | MYB | |
| g25572.t1 | MIKC_MADS | | Vitvi18g01044.t01 | MIKC_MADS | | chr4.gff3_PROT_VIT_04s0044g01380.t01 | | MYB | |
| g25901.t1 | MIKC_MADS | | Vitvi18g02133.t01 | MIKC_MADS | | chr5.gff3_PROT_VIT_05s0020g01100.t01 | | MYB | |
| g2597.t1 | MIKC_MADS | | Vitvi18g02145.t01 | MIKC_MADS | | chr5.gff3_PROT_VIT_05s0049g01010.t01 | | MYB | |
| g268.t1 | MIKC_MADS | | Vitvi00g01439.t01 | M-type_MADS | | chr5.gff3_PROT_VIT_05s0049g01020.t01 | | MYB | |
| g28775.t1 | MIKC_MADS | | Vitvi01g01424.t01 | M-type_MADS | | chr5.gff3_PROT_VIT_05s0049g02260.t01 | | MYB | |
| g28776.t1 | MIKC_MADS | | Vitvi01g01831.t01 | M-type_MADS | | chr5.gff3_PROT_VIT_05s0077g00500.t01 | | MYB | |
| g29795.t1 | MIKC_MADS | | Vitvi02g01303.t01 | M-type_MADS | | chr5.gff3_PROT_VIT_05s0077g01360.t01 | | MYB | |
| g29797.t1 | MIKC_MADS | | Vitvi02g01306.t01 | M-type_MADS | | chr6.gff3_PROT_VIT_06s0004g00570.t01 | | MYB | |
| g29972.t1 | MIKC_MADS | | Vitvi03g00729.t01 | M-type_MADS | | chr6.gff3_PROT_VIT_06s0004g02110.t01 | | MYB | |
| g29972.t2 | MIKC_MADS | | Vitvi03g00730.t01 | M-type_MADS | | chr6.gff3_PROT_VIT_06s0004g04140.t01 | | MYB | |
| g33612.t1 | MIKC_MADS | | Vitvi03g00732.t01 | M-type_MADS | | chr6.gff3_PROT_VIT_06s0004g06100.t01 | | MYB | |
| g33930.t1 | MIKC_MADS | | Vitvi03g00733.t01 | M-type_MADS | | chr6.gff3_PROT_VIT_06s0004g06280.t01 | | MYB | |
| g35674.t1 | MIKC_MADS | | Vitvi03g00734.t01 | M-type_MADS | | chr6.gff3_PROT_VIT_06s0009g02480.t01 | | MYB | |
| g35675.t1 | MIKC_MADS | | Vitvi03g01067.t01 | M-type_MADS | | chr6.gff3_PROT_VIT_06s0061g00470.t01 | | MYB | |
| g36000.t1 | MIKC_MADS | | Vitvi03g01317.t01 | M-type_MADS | | chr6.gff3_PROT_VIT_06s0080g00790.t01 | | MYB | |
| g38107.t1 | MIKC_MADS | | Vitvi03g01318.t01 | M-type_MADS | | chr7.gff3_PROT_VIT_07s0005g01210.t01 | | MYB | |
| g902.t1 | MIKC_MADS | | Vitvi04g00171.t01 | M-type_MADS | | chr7.gff3_PROT_VIT_07s0005g01950.t01 | | MYB | |
| g10608.t1 | M-type_MADS | | Vitvi04g01016.t01 | M-type_MADS | | chr7.gff3_PROT_VIT_07s0005g02480.t01 | | MYB | |
| g16550.t1 | M-type_MADS | | Vitvi05g01725.t01 | M-type_MADS | | chr7.gff3_PROT_VIT_07s0005g03340.t01 | | MYB | |
| g17649.t1 | M-type_MADS | | Vitvi05g01726.t01 | M-type_MADS | | chr7.gff3_PROT_VIT_07s0129g01050.t01 | | MYB | |
| g18789.t1 | M-type_MADS | | Vitvi05g01727.t01 | M-type_MADS | | chr7.gff3_PROT_VIT_07s0130g00040.t01 | | MYB | |
| g18790.t1 | M-type_MADS | | Vitvi05g01728.t01 | M-type_MADS | | chr7_random.gff3_PROT_VIT_07s0141g00100.t01 | | MYB | |
| g18792.t1 | M-type_MADS | | Vitvi05g01729.t01 | M-type_MADS | | chr8.gff3_PROT_VIT_08s0007g00360.t01 | | MYB | |
| g18793.t1 | M-type_MADS | | Vitvi05g01730.t01 | M-type_MADS | | chr8.gff3_PROT_VIT_08s0007g00410.t01 | | MYB | |
| g19862.t1 | M-type_MADS | | Vitvi07g02071.t01 | M-type_MADS | | chr8.gff3_PROT_VIT_08s0007g01540.t01 | | MYB | |
| g19948.t1 | M-type_MADS | | Vitvi07g02072.t01 | M-type_MADS | | chr8.gff3_PROT_VIT_08s0007g01920.t01 | | MYB | |
| g20076.t1 | M-type_MADS | | Vitvi07g03065.t01 | M-type_MADS | | chr8.gff3_PROT_VIT_08s0007g04830.t01 | | MYB | |
| g20090.t1 | M-type_MADS | | Vitvi08g01967.t01 | M-type_MADS | | chr8.gff3_PROT_VIT_08s0007g05030.t01 | | MYB | |
| g20176.t1 | M-type_MADS | | Vitvi08g01968.t01 | M-type_MADS | | chr8.gff3_PROT_VIT_08s0007g07230.t01 | | MYB | |
| g20599.t1 | M-type_MADS | | Vitvi10g00842.t01 | M-type_MADS | | chr8.gff3_PROT_VIT_08s0056g00800.t01 | | MYB | |
| g21471.t1 | M-type_MADS | | Vitvi10g01588.t01 | M-type_MADS | | chr8.gff3_PROT_VIT_08s0056g01190.t01 | | MYB | |
| g21749.t1 | M-type_MADS | | Vitvi10g01589.t01 | M-type_MADS | | chr9.gff3_PROT_VIT_09s0002g01380.t01 | | MYB | |
| g21752.t1 | M-type_MADS | | Vitvi10g01590.t01 | M-type_MADS | | chr9.gff3_PROT_VIT_09s0002g01400.t01 | | MYB | |
| g22009.t1 | M-type_MADS | | Vitvi10g01591.t01 | M-type_MADS | | chr9.gff3_PROT_VIT_09s0002g01410.t01 | | MYB | |
| g22011.t1 | M-type_MADS | | Vitvi10g01592.t01 | M-type_MADS | | chr9.gff3_PROT_VIT_09s0002g01670.t01 | | MYB | |
| g23022.t1 | M-type_MADS | | Vitvi10g01593.t01 | M-type_MADS | | chrUn.gff3_PROT_VIT_00s0203g00070.t01 | | MYB | |
| g23655.t1 | M-type_MADS | | Vitvi12g00559.t01 | M-type_MADS | | chrUn.gff3_PROT_VIT_00s0203g00170.t01 | | MYB | |
| g25763.t1 | M-type_MADS | | Vitvi13g02085.t01 | M-type_MADS | | chrUn.gff3_PROT_VIT_00s0341g00050.t01 | | MYB | |
| g2595.t1 | M-type_MADS | | Vitvi14g00026.t01 | M-type_MADS | | chrUn.gff3_PROT_VIT_00s1241g00010.t01 | | MYB | |
| g2596.t1 | M-type_MADS | | Vitvi15g01207.t01 | M-type_MADS | | chrUn.gff3_PROT_VIT_00s1352g00010.t01 | | MYB | |
| g2598.t1 | M-type_MADS | | Vitvi15g01208.t01 | M-type_MADS | | chr1.gff3_PROT_VIT_01s0011g00790.t01 | | MYB_related | |
| g27804.t1 | M-type_MADS | | Vitvi15g01209.t01 | M-type_MADS | | chr1.gff3_PROT_VIT_01s0026g01050.t01 | | MYB_related | |
| g30374.t1 | M-type_MADS | | Vitvi15g01210.t01 | M-type_MADS | | chr10.gff3_PROT_VIT_10s0116g00500.t01 | | MYB_related | |
| g30679.t1 | M-type_MADS | | Vitvi15g01211.t01 | M-type_MADS | | chr10.gff3_PROT_VIT_10s0116g01760.t01 | | MYB_related | |
| g32510.t1 | M-type_MADS | | Vitvi15g01212.t01 | M-type_MADS | | chr11.gff3_PROT_VIT_11s0016g01890.t01 | | MYB_related | |
| g32511.t1 | M-type_MADS | | Vitvi15g01213.t01 | M-type_MADS | | chr11.gff3_PROT_VIT_11s0016g02410.t01 | | MYB_related | |
| g32512.t1 | M-type_MADS | | Vitvi15g01214.t01 | M-type_MADS | | chr11.gff3_PROT_VIT_11s0016g05650.t01 | | MYB_related | |
| g32513.t1 | M-type_MADS | | Vitvi15g01743.t01 | M-type_MADS | | chr12.gff3_PROT_VIT_12s0057g01490.t01 | | MYB_related | |
| g32514.t1 | M-type_MADS | | Vitvi17g00614.t01 | M-type_MADS | | chr12.gff3_PROT_VIT_12s0059g02360.t01 | | MYB_related | |
| g33676.t1 | M-type_MADS | | Vitvi17g01306.t01 | M-type_MADS | | chr13.gff3_PROT_VIT_13s0019g01670.t01 | | MYB_related | |
| g33767.t1 | M-type_MADS | | Vitvi17g01307.t01 | M-type_MADS | | chr13.gff3_PROT_VIT_13s0106g00370.t01 | | MYB_related | |
| g33772.t1 | M-type_MADS | | Vitvi17g01308.t01 | M-type_MADS | | chr13.gff3_PROT_VIT_13s0156g00340.t01 | | MYB_related | |
| g3437.t1 | M-type_MADS | | Vitvi19g00027.t01 | M-type_MADS | | chr14.gff3_PROT_VIT_14s0036g00460.t01 | | MYB_related | |
| g35443.t1 | M-type_MADS | | Vitvi19g01487.t01 | M-type_MADS | | chr14.gff3_PROT_VIT_14s0036g00500.t01 | | MYB_related | |
| g35741.t1 | M-type_MADS | | Vitvi19g01491.t01 | M-type_MADS | | chr14.gff3_PROT_VIT_14s0036g00510.t01 | | MYB_related | |
| g35999.t1 | M-type_MADS | | Vitvi19g02367.t01 | M-type_MADS | | chr14.gff3_PROT_VIT_14s0128g00860.t01 | | MYB_related | |
| g36185.t1 | M-type_MADS | | Vitvi01g00094.t01 | MYB | | chr15.gff3_PROT_VIT_15s0046g01130.t01 | | MYB_related | |
| g36665.t1 | M-type_MADS | | Vitvi01g00095.t01 | MYB | | chr15.gff3_PROT_VIT_15s0046g01790.t01 | | MYB_related | |
| g37305.t1 | M-type_MADS | | Vitvi01g00302.t01 | MYB | | chr15.gff3_PROT_VIT_15s0046g02260.t01 | | MYB_related | |
| g5931.t1 | M-type_MADS | | Vitvi01g00401.t01 | MYB | | chr15.gff3_PROT_VIT_15s0048g02410.t01 | | MYB_related | |
| g7114.t1 | M-type_MADS | | Vitvi01g00737.t01 | MYB | | chr16.gff3_PROT_VIT_16s0039g01920.t01 | | MYB_related | |
| g7714.t1 | M-type_MADS | | Vitvi01g00956.t01 | MYB | | chr16.gff3_PROT_VIT_16s0050g01180.t01 | | MYB_related | |
| g7715.t1 | M-type_MADS | | Vitvi01g01028.t01 | MYB | | chr16.gff3_PROT_VIT_16s0050g02530.t01 | | MYB_related | |
| g8177.t1 | M-type_MADS | | Vitvi01g01052.t01 | MYB | | chr17.gff3_PROT_VIT_17s0000g07510.t01 | | MYB_related | |
| g8818.t1 | M-type_MADS | | Vitvi01g01271.t01 | MYB | | chr18.gff3_PROT_VIT_18s0001g02160.t01 | | MYB_related | |
| g10287.t1 | MYB | | Vitvi02g00028.t01 | MYB | | chr18.gff3_PROT_VIT_18s0001g07620.t01 | | MYB_related | |
| g1047.t1 | MYB | | Vitvi02g00195.t01 | MYB | | chr18.gff3_PROT_VIT_18s0001g12530.t01 | | MYB_related | |
| g11583.t1 | MYB | | Vitvi02g00725.t01 | MYB | | chr18.gff3_PROT_VIT_18s0001g12610.t01 | | MYB_related | |
| g11584.t1 | MYB | | Vitvi02g01015.t01 | MYB | | chr18_random.gff3_PROT_VIT_18s0001g01170.t01 | | MYB_related | |
| g11768.t1 | MYB | | Vitvi02g01019.t01 | MYB | | chr19.gff3_PROT_VIT_19s0015g01170.t01 | | MYB_related | |
| g12303.t1 | MYB | | Vitvi02g01024.t01 | MYB | | chr2.gff3_PROT_VIT_02s0012g00630.t01 | | MYB_related | |
| g12350.t1 | MYB | | Vitvi02g01307.t01 | MYB | | chr2.gff3_PROT_VIT_02s0033g00370.t01 | | MYB_related | |
| g12485.t1 | MYB | | Vitvi02g01308.t01 | MYB | | chr2.gff3_PROT_VIT_02s0033g00440.t01 | | MYB_related | |
| g12605.t1 | MYB | | Vitvi02g01400.t01 | MYB | | chr2.gff3_PROT_VIT_02s0033g00460.t01 | | MYB_related | |
| g1281.t1 | MYB | | Vitvi02g01732.t01 | MYB | | chr3.gff3_PROT_VIT_03s0038g04460.t01 | | MYB_related | |
| g12826.t1 | MYB | | Vitvi02g01823.t01 | MYB | | chr3.gff3_PROT_VIT_03s0063g02620.t01 | | MYB_related | |
| g13148.t1 | MYB | | Vitvi03g00136.t01 | MYB | | chr3.gff3_PROT_VIT_03s0063g02670.t01 | | MYB_related | |
| g13157.t1 | MYB | | Vitvi03g00559.t01 | MYB | | chr3.gff3_PROT_VIT_03s0063g02680.t01 | | MYB_related | |
| g13376.t1 | MYB | | Vitvi04g00049.t01 | MYB | | chr3.gff3_PROT_VIT_03s0091g01030.t01 | | MYB_related | |
| g13376.t2 | MYB | | Vitvi04g00153.t01 | MYB | | chr4.gff3_PROT_VIT_04s0008g00900.t01 | | MYB_related | |
| g13581.t1 | MYB | | Vitvi04g00155.t01 | MYB | | chr4.gff3_PROT_VIT_04s0023g01910.t01 | | MYB_related | |
| g13736.t1 | MYB | | Vitvi04g00156.t01 | MYB | | chr4.gff3_PROT_VIT_04s0079g00410.t01 | | MYB_related | |
| g13773.t1 | MYB | | Vitvi04g00157.t01 | MYB | | chr5.gff3_PROT_VIT_05s0020g01610.t01 | | MYB_related | |
| g13970.t1 | MYB | | Vitvi04g00158.t01 | MYB | | chr6.gff3_PROT_VIT_06s0004g02520.t01 | | MYB_related | |
| g14472.t1 | MYB | | Vitvi04g00160.t01 | MYB | | chr6.gff3_PROT_VIT_06s0004g04940.t01 | | MYB_related | |
| g14651.t1 | MYB | | Vitvi04g00331.t01 | MYB | | chr6.gff3_PROT_VIT_06s0004g08050.t01 | | MYB_related | |
| g15207.t1 | MYB | | Vitvi04g01486.t01 | MYB | | chr7.gff3_PROT_VIT_07s0005g01910.t01 | | MYB_related | |
| g15752.t1 | MYB | | Vitvi04g01697.t01 | MYB | | chr7.gff3_PROT_VIT_07s0031g02280.t01 | | MYB_related | |
| g15851.t1 | MYB | | Vitvi04g01842.t01 | MYB | | chr7_random.gff3_PROT_VIT_07s0151g00480.t01 | | MYB_related | |
| g15957.t1 | MYB | | Vitvi04g01855.t01 | MYB | | chr8.gff3_PROT_VIT_08s0007g00500.t01 | | MYB_related | |
| g15987.t1 | MYB | | Vitvi05g00084.t01 | MYB | | chr8.gff3_PROT_VIT_08s0007g08160.t01 | | MYB_related | |
| g16097.t1 | MYB | | Vitvi05g00166.t01 | MYB | | chr8.gff3_PROT_VIT_08s0040g01640.t01 | | MYB_related | |
| g16275.t1 | MYB | | Vitvi05g00275.t01 | MYB | | chr8.gff3_PROT_VIT_08s0040g03220.t01 | | MYB_related | |
| g16350.t1 | MYB | | Vitvi05g00861.t01 | MYB | | chr9.gff3_PROT_VIT_09s0002g01480.t01 | | MYB_related | |
| g17011.t1 | MYB | | Vitvi05g01732.t01 | MYB | | chr9.gff3_PROT_VIT_09s0002g03540.t01 | | MYB_related | |
| g17095.t1 | MYB | | Vitvi05g01733.t01 | MYB | | chr9.gff3_PROT_VIT_09s0002g03610.t01 | | MYB_related | |
| g17113.t1 | MYB | | Vitvi06g00059.t01 | MYB | | chrUn.gff3_PROT_VIT_00s0299g00060.t01 | | MYB_related | |
| g17515.t1 | MYB | | Vitvi06g00214.t01 | MYB | | chrUn.gff3_PROT_VIT_00s1311g00020.t01 | | MYB_related | |
| g17534.t1 | MYB | | Vitvi06g00414.t01 | MYB | | chr1.gff3_PROT_VIT_01s0011g02990.t01 | | NAC | |
| g17729.t1 | MYB | | Vitvi06g00592.t01 | MYB | | chr1.gff3_PROT_VIT_01s0026g02360.t01 | | NAC | |
| g17920.t1 | MYB | | Vitvi06g00611.t01 | MYB | | chr1.gff3_PROT_VIT_01s0026g02710.t01 | | NAC | |
| g18051.t1 | MYB | | Vitvi06g01139.t01 | MYB | | chr1.gff3_PROT_VIT_01s0146g00280.t01 | | NAC | |
| g18211.t1 | MYB | | Vitvi06g01321.t01 | MYB | | chr10.gff3_PROT_VIT_10s0003g00350.t01 | | NAC | |
| g18408.t1 | MYB | | Vitvi06g01516.t01 | MYB | | chr11.gff3_PROT_VIT_11s0016g02880.t01 | | NAC | |
| g18636.t1 | MYB | | Vitvi07g00393.t01 | MYB | | chr12.gff3_PROT_VIT_12s0028g00860.t01 | | NAC | |
| g19001.t1 | MYB | | Vitvi07g00455.t01 | MYB | | chr12.gff3_PROT_VIT_12s0028g03050.t01 | | NAC | |
| g19466.t1 | MYB | | Vitvi07g00515.t01 | MYB | | chr12.gff3_PROT_VIT_12s0035g02020.t01 | | NAC | |
| g19683.t1 | MYB | | Vitvi07g00598.t01 | MYB | | chr12.gff3_PROT_VIT_12s0055g00510.t01 | | NAC | |
| g19855.t1 | MYB | | Vitvi07g01676.t01 | MYB | | chr13.gff3_PROT_VIT_13s0019g05230.t01 | | NAC | |
| g20290.t1 | MYB | | Vitvi07g02009.t01 | MYB | | chr13.gff3_PROT_VIT_13s0019g05240.t01 | | NAC | |
| g20557.t1 | MYB | | Vitvi07g02075.t01 | MYB | | chr14.gff3_PROT_VIT_14s0066g00360.t01 | | NAC | |
| g20700.t1 | MYB | | Vitvi07g03055.t01 | MYB | | chr14.gff3_PROT_VIT_14s0068g01490.t01 | | NAC | |
| g21037.t1 | MYB | | Vitvi08g00069.t01 | MYB | | chr14.gff3_PROT_VIT_14s0108g00860.t01 | | NAC | |
| g2113.t1 | MYB | | Vitvi08g00107.t01 | MYB | | chr14.gff3_PROT_VIT_14s0108g01070.t01 | | NAC | |
| g21692.t1 | MYB | | Vitvi08g01203.t01 | MYB | | chr15.gff3_PROT_VIT_15s0048g02270.t01 | | NAC | |
| g21693.t1 | MYB | | Vitvi08g01298.t01 | MYB | | chr15.gff3_PROT_VIT_15s0048g02280.t01 | | NAC | |
| g2177.t1 | MYB | | Vitvi08g01336.t01 | MYB | | chr15.gff3_PROT_VIT_15s0048g02290.t01 | | NAC | |
| g22067.t1 | MYB | | Vitvi08g01607.t01 | MYB | | chr15.gff3_PROT_VIT_15s0048g02300.t01 | | NAC | |
| g22085.t1 | MYB | | Vitvi08g01623.t01 | MYB | | chr15.gff3_PROT_VIT_15s0048g02310.t01 | | NAC | |
| g22086.t1 | MYB | | Vitvi08g01797.t01 | MYB | | chr15.gff3_PROT_VIT_15s0048g02320.t01 | | NAC | |
| g22302.t1 | MYB | | Vitvi08g02196.t01 | MYB | | chr15.gff3_PROT_VIT_15s0048g02340.t01 | | NAC | |
| g22303.t1 | MYB | | Vitvi09g00110.t01 | MYB | | chr15.gff3_PROT_VIT_15s0048g02660.t01 | | NAC | |
| g22304.t1 | MYB | | Vitvi09g00112.t01 | MYB | | chr16.gff3_PROT_VIT_16s0022g00690.t01 | | NAC | |
| g22617.t1 | MYB | | Vitvi09g00113.t01 | MYB | | chr16.gff3_PROT_VIT_16s0098g00760.t01 | | NAC | |
| g229.t1 | MYB | | Vitvi09g00142.t01 | MYB | | chr17.gff3_PROT_VIT_17s0000g00770.t01 | | NAC | |
| g23078.t1 | MYB | | Vitvi10g00329.t01 | MYB | | chr17.gff3_PROT_VIT_17s0000g03660.t01 | | NAC | |
| g23422.t1 | MYB | | Vitvi10g00345.t01 | MYB | | chr17.gff3_PROT_VIT_17s0000g06400.t01 | | NAC | |
| g2391.t1 | MYB | | Vitvi10g01533.t01 | MYB | | chr17.gff3_PROT_VIT_17s0000g06990.t01 | | NAC | |
| g2392.t1 | MYB | | Vitvi11g00097.t01 | MYB | | chr17.gff3_PROT_VIT_17s0053g00740.t01 | | NAC | |
| g24050.t1 | MYB | | Vitvi11g00098.t01 | MYB | | chr18.gff3_PROT_VIT_18s0001g02300.t01 | | NAC | |
| g24064.t1 | MYB | | Vitvi11g00099.t01 | MYB | | chr18.gff3_PROT_VIT_18s0001g10250.t01 | | NAC | |
| g24228.t1 | MYB | | Vitvi11g00228.t01 | MYB | | chr18.gff3_PROT_VIT_18s0001g11980.t01 | | NAC | |
| g24535.t1 | MYB | | Vitvi11g00320.t01 | MYB | | chr18.gff3_PROT_VIT_18s0041g00700.t01 | | NAC | |
| g24587.t1 | MYB | | Vitvi11g00499.t01 | MYB | | chr18.gff3_PROT_VIT_18s0072g01060.t01 | | NAC | |
| g24592.t1 | MYB | | Vitvi11g00502.t01 | MYB | | chr18.gff3_PROT_VIT_18s0089g01120.t01 | | NAC | |
| g24677.t1 | MYB | | Vitvi11g01283.t01 | MYB | | chr18.gff3_PROT_VIT_18s0122g00800.t01 | | NAC | |
| g24682.t1 | MYB | | Vitvi11g01323.t01 | MYB | | chr18_random.gff3_PROT_VIT_18s0001g01820.t01 | | NAC | |
| g24848.t1 | MYB | | Vitvi12g00376.t01 | MYB | | chr19.gff3_PROT_VIT_19s0014g02200.t01 | | NAC | |
| g24853.t1 | MYB | | Vitvi12g00625.t01 | MYB | | chr19.gff3_PROT_VIT_19s0014g03290.t01 | | NAC | |
| g2507.t1 | MYB | | Vitvi12g00626.t01 | MYB | | chr19.gff3_PROT_VIT_19s0014g03300.t01 | | NAC | |
| g25123.t1 | MYB | | Vitvi12g00632.t01 | MYB | | chr19.gff3_PROT_VIT_19s0027g00230.t01 | | NAC | |
| g25231.t1 | MYB | | Vitvi13g00076.t01 | MYB | | chr19.gff3_PROT_VIT_19s0027g00840.t01 | | NAC | |
| g25236.t1 | MYB | | Vitvi13g00494.t01 | MYB | | chr19.gff3_PROT_VIT_19s0027g00860.t01 | | NAC | |
| g25237.t1 | MYB | | Vitvi13g00510.t01 | MYB | | chr19.gff3_PROT_VIT_19s0027g00870.t01 | | NAC | |
| g25488.t1 | MYB | | Vitvi13g00573.t01 | MYB | | chr19.gff3_PROT_VIT_19s0027g00880.t01 | | NAC | |
| g25720.t1 | MYB | | Vitvi13g01266.t01 | MYB | | chr19.gff3_PROT_VIT_19s0027g00910.t01 | | NAC | |
| g25721.t1 | MYB | | Vitvi13g01359.t01 | MYB | | chr19.gff3_PROT_VIT_19s0085g00950.t01 | | NAC | |
| g25729.t1 | MYB | | Vitvi13g01737.t01 | MYB | | chr19.gff3_PROT_VIT_19s0085g01210.t01 | | NAC | |
| g25869.t1 | MYB | | Vitvi14g00018.t01 | MYB | | chr2.gff3_PROT_VIT_02s0012g01040.t01 | | NAC | |
| g25870.t1 | MYB | | Vitvi14g00612.t01 | MYB | | chr2.gff3_PROT_VIT_02s0025g02710.t01 | | NAC | |
| g26011.t1 | MYB | | Vitvi14g00618.t01 | MYB | | chr2.gff3_PROT_VIT_02s0025g03020.t01 | | NAC | |
| g2603.t1 | MYB | | Vitvi14g00925.t01 | MYB | | chr2.gff3_PROT_VIT_02s0154g00020.t01 | | NAC | |
| g26111.t1 | MYB | | Vitvi14g00930.t01 | MYB | | chr3.gff3_PROT_VIT_03s0038g03410.t01 | | NAC | |
| g26493.t1 | MYB | | Vitvi14g00940.t01 | MYB | | chr4.gff3_PROT_VIT_04s0008g02710.t01 | | NAC | |
| g26747.t1 | MYB | | Vitvi14g00974.t01 | MYB | | chr4.gff3_PROT_VIT_04s0008g06550.t01 | | NAC | |
| g26803.t1 | MYB | | Vitvi14g01247.t01 | MYB | | chr4.gff3_PROT_VIT_04s0023g03110.t01 | | NAC | |
| g27073.t1 | MYB | | Vitvi14g01346.t01 | MYB | | chr4.gff3_PROT_VIT_04s0044g01220.t01 | | NAC | |
| g27091.t1 | MYB | | Vitvi14g01615.t01 | MYB | | chr4.gff3_PROT_VIT_04s0044g01500.t01 | | NAC | |
| g27100.t1 | MYB | | Vitvi14g01740.t01 | MYB | | chr4.gff3_PROT_VIT_04s0079g00280.t01 | | NAC | |
| g27273.t1 | MYB | | Vitvi14g01750.t01 | MYB | | chr4_random.gff3_PROT_VIT_04s0008g00150.t01 | | NAC | |
| g27329.t1 | MYB | | Vitvi14g01845.t01 | MYB | | chr6.gff3_PROT_VIT_06s0004g00020.t01 | | NAC | |
| g27750.t1 | MYB | | Vitvi14g01960.t01 | MYB | | chr6.gff3_PROT_VIT_06s0004g02340.t01 | | NAC | |
| g27755.t1 | MYB | | Vitvi14g01976.t01 | MYB | | chr6.gff3_PROT_VIT_06s0004g03080.t01 | | NAC | |
| g27758.t1 | MYB | | Vitvi14g01987.t01 | MYB | | chr6.gff3_PROT_VIT_06s0080g00780.t01 | | NAC | |
| g28207.t1 | MYB | | Vitvi14g02430.t01 | MYB | | chr6.gff3_PROT_VIT_06s0080g00970.t01 | | NAC | |
| g28423.t1 | MYB | | Vitvi14g03020.t01 | MYB | | chr7.gff3_PROT_VIT_07s0005g03610.t01 | | NAC | |
| g2903.t1 | MYB | | Vitvi15g00594.t01 | MYB | | chr7.gff3_PROT_VIT_07s0031g02610.t01 | | NAC | |
| g29074.t1 | MYB | | Vitvi15g00847.t01 | MYB | | chr8.gff3_PROT_VIT_08s0007g02940.t01 | | NAC | |
| g29077.t1 | MYB | | Vitvi15g00938.t01 | MYB | | chr8.gff3_PROT_VIT_08s0007g07640.t01 | | NAC | |
| g29703.t1 | MYB | | Vitvi15g01161.t01 | MYB | | chr8.gff3_PROT_VIT_08s0007g07670.t01 | | NAC | |
| g29729.t1 | MYB | | Vitvi16g00098.t01 | MYB | | chr8.gff3_PROT_VIT_08s0040g02110.t01 | | NAC | |
| g29760.t1 | MYB | | Vitvi16g00103.t01 | MYB | | chrUn.gff3_PROT_VIT_00s0375g00040.t01 | | NAC | |
| g29881.t1 | MYB | | Vitvi16g00106.t01 | MYB | | chr13.gff3_PROT_VIT_13s0067g00920.t01 | | NF-X1 | |
| g30634.t1 | MYB | | Vitvi16g00125.t01 | MYB | | chr17.gff3_PROT_VIT_17s0000g01920.t01 | | NF-X1 | |
| g30635.t1 | MYB | | Vitvi16g00441.t01 | MYB | | chr19.gff3_PROT_VIT_19s0014g00390.t01 | | NF-X1 | |
| g30825.t1 | MYB | | Vitvi16g00442.t01 | MYB | | chr10.gff3_PROT_VIT_10s0003g04650.t01 | | NF-YA | |
| g30828.t1 | MYB | | Vitvi16g00775.t01 | MYB | | chr11.gff3_PROT_VIT_11s0016g01480.t01 | | NF-YA | |
| g30830.t1 | MYB | | Vitvi16g01015.t01 | MYB | | chr13.gff3_PROT_VIT_13s0064g00860.t01 | | NF-YA | |
| g30834.t1 | MYB | | Vitvi16g01017.t01 | MYB | | chr6.gff3_PROT_VIT_06s0004g02270.t01 | | NF-YA | |
| g30835.t1 | MYB | | Vitvi16g01448.t01 | MYB | | chr8.gff3_PROT_VIT_08s0007g08250.t01 | | NF-YA | |
| g30839.t1 | MYB | | Vitvi16g01522.t01 | MYB | | chr8.gff3_PROT_VIT_08s0032g01190.t01 | | NF-YA | |
| g30842.t1 | MYB | | Vitvi16g01523.t01 | MYB | | chr9.gff3_PROT_VIT_09s0002g01590.t01 | | NF-YA | |
| g31052.t1 | MYB | | Vitvi16g01689.t01 | MYB | | chr1.gff3_PROT_VIT_01s0010g01910.t01 | | NF-YB | |
| g31078.t1 | MYB | | Vitvi17g00159.t01 | MYB | | chr1.gff3_PROT_VIT_01s0010g01940.t01 | | NF-YB | |
| g31080.t1 | MYB | | Vitvi17g00231.t01 | MYB | | chr12.gff3_PROT_VIT_12s0057g01010.t01 | | NF-YB | |
| g31082.t1 | MYB | | Vitvi17g00232.t01 | MYB | | chr13.gff3_PROT_VIT_13s0019g03600.t01 | | NF-YB | |
| g31335.t1 | MYB | | Vitvi17g00238.t01 | MYB | | chr14.gff3_PROT_VIT_14s0060g02660.t01 | | NF-YB | |
| g32182.t1 | MYB | | Vitvi17g00309.t01 | MYB | | chr17.gff3_PROT_VIT_17s0000g04350.t01 | | NF-YB | |
| g32204.t1 | MYB | | Vitvi17g00366.t01 | MYB | | chr19.gff3_PROT_VIT_19s0015g00440.t01 | | NF-YB | |
| g32787.t1 | MYB | | Vitvi17g00510.t01 | MYB | | chr19.gff3_PROT_VIT_19s0015g00580.t01 | | NF-YB | |
| g3287.t1 | MYB | | Vitvi17g00598.t01 | MYB | | chr19.gff3_PROT_VIT_19s0015g00590.t01 | | NF-YB | |
| g32947.t1 | MYB | | Vitvi17g00623.t01 | MYB | | chr5.gff3_PROT_VIT_05s0020g01350.t01 | | NF-YB | |
| g33234.t1 | MYB | | Vitvi17g00822.t01 | MYB | | chr6.gff3_PROT_VIT_06s0004g03490.t01 | | NF-YB | |
| g33806.t1 | MYB | | Vitvi17g00832.t01 | MYB | | chr6.gff3_PROT_VIT_06s0080g00460.t01 | | NF-YB | |
| g3420.t1 | MYB | | Vitvi17g00895.t01 | MYB | | chr7.gff3_PROT_VIT_07s0031g01460.t01 | | NF-YB | |
| g3421.t1 | MYB | | Vitvi18g00406.t01 | MYB | | chr7.gff3_PROT_VIT_07s0104g01640.t01 | | NF-YB | |
| g3422.t1 | MYB | | Vitvi18g00605.t01 | MYB | | chr8.gff3_PROT_VIT_08s0040g03260.t01 | | NF-YB | |
| g3423.t1 | MYB | | Vitvi18g00725.t01 | MYB | | chrUn.gff3_PROT_VIT_00s0259g00180.t01 | | NF-YB | |
| g3424.t1 | MYB | | Vitvi18g00843.t01 | MYB | | chrUn.gff3_PROT_VIT_00s0956g00020.t01 | | NF-YB | |
| g35922.t1 | MYB | | Vitvi18g01209.t01 | MYB | | chrUn.gff3_PROT_VIT_00s1287g00010.t01 | | NF-YB | |
| g35935.t1 | MYB | | Vitvi18g01533.t01 | MYB | | chr1.gff3_PROT_VIT_01s0010g03550.t01 | | NF-YC | |
| g36077.t1 | MYB | | Vitvi18g01867.t01 | MYB | | chr13.gff3_PROT_VIT_13s0084g00040.t01 | | NF-YC | |
| g36079.t1 | MYB | | Vitvi18g01868.t01 | MYB | | chr14.gff3_PROT_VIT_14s0006g02320.t01 | | NF-YC | |
| g3619.t1 | MYB | | Vitvi19g00306.t01 | MYB | | chr14.gff3_PROT_VIT_14s0128g00250.t01 | | NF-YC | |
| g3620.t1 | MYB | | Vitvi19g00508.t01 | MYB | | chr17.gff3_PROT_VIT_17s0000g01130.t01 | | NF-YC | |
| g3621.t1 | MYB | | Vitvi19g00758.t01 | MYB | | chr19.gff3_PROT_VIT_19s0085g00520.t01 | | NF-YC | |
| g36227.t1 | MYB | | Vitvi19g01669.t01 | MYB | | chr2.gff3_PROT_VIT_02s0025g03770.t01 | | NF-YC | |
| g36686.t1 | MYB | | Vitvi19g01749.t01 | MYB | | chr5.gff3_PROT_VIT_05s0020g02790.t01 | | NF-YC | |
| g36924.t1 | MYB | | Vitvi01g00071.t01 | MYB_related | | chr6.gff3_PROT_VIT_06s0004g03030.t01 | | NF-YC | |
| g37000.t1 | MYB | | Vitvi01g00867.t01 | MYB_related | | chr6.gff3_PROT_VIT_06s0009g02120.t01 | | NF-YC | |
| g37762.t1 | MYB | | Vitvi02g00196.t01 | MYB_related | | chr12.gff3_PROT_VIT_12s0028g00370.t01 | | Nin-like | |
| g37764.t1 | MYB | | Vitvi02g00645.t01 | MYB_related | | chr16.gff3_PROT_VIT_16s0098g01730.t01 | | Nin-like | |
| g37765.t1 | MYB | | Vitvi02g01013.t01 | MYB_related | | chr17.gff3_PROT_VIT_17s0000g06250.t01 | | Nin-like | |
| g37767.t1 | MYB | | Vitvi02g01017.t01 | MYB_related | | chr18.gff3_PROT_VIT_18s0122g01290.t01 | | Nin-like | |
| g37769.t1 | MYB | | Vitvi02g01022.t01 | MYB_related | | chr2.gff3_PROT_VIT_02s0025g02070.t01 | | Nin-like | |
| g37769.t2 | MYB | | Vitvi02g01309.t01 | MYB_related | | chr3.gff3_PROT_VIT_03s0038g03710.t01 | | Nin-like | |
| g37779.t1 | MYB | | Vitvi02g01721.t01 | MYB_related | | chr4.gff3_PROT_VIT_04s0044g00060.t01 | | Nin-like | |
| g4257.t1 | MYB | | Vitvi03g00293.t01 | MYB_related | | chr5.gff3_PROT_VIT_05s0094g00020.t01 | | Nin-like | |
| g4276.t1 | MYB | | Vitvi03g00318.t01 | MYB_related | | chr19.gff3_PROT_VIT_19s0014g01700.t01 | | NZZ/SPL | |
| g439.t1 | MYB | | Vitvi03g00325.t01 | MYB_related | | chr19.gff3_PROT_VIT_19s0014g03940.t01 | | NZZ/SPL | |
| g5037.t1 | MYB | | Vitvi03g00668.t01 | MYB_related | | chr1.gff3_PROT_VIT_01s0011g03070.t01 | | RAV | |
| g5077.t1 | MYB | | Vitvi03g01247.t01 | MYB_related | | chr14.gff3_PROT_VIT_14s0083g00130.t01 | | RAV | |
| g5161.t1 | MYB | | Vitvi03g01495.t01 | MYB_related | | chr11.gff3_PROT_VIT_11s0037g00010.t01 | | RAV | |
| g5233.t1 | MYB | | Vitvi04g00076.t01 | MYB_related | | chr6.gff3_PROT_VIT_06s0004g03160.t01 | | S1Fa-like | |
| g569.t1 | MYB | | Vitvi04g00845.t01 | MYB_related | | chr8.gff3_PROT_VIT_08s0040g02220.t01 | | S1Fa-like | |
| g5764.t1 | MYB | | Vitvi04g01309.t01 | MYB_related | | chr12.gff3_PROT_VIT_12s0057g00910.t01 | | SAP | |
| g6138.t1 | MYB | | Vitvi05g00330.t01 | MYB_related | | chr1.gff3_PROT_VIT_01s0010g03710.t01 | | SBP | |
| g682.t1 | MYB | | Vitvi06g00245.t01 | MYB_related | | chr1.gff3_PROT_VIT_01s0010g03910.t01 | | SBP | |
| g682.t2 | MYB | | Vitvi06g00488.t01 | MYB_related | | chr1.gff3_PROT_VIT_01s0011g00130.t01 | | SBP | |
| g8673.t1 | MYB | | Vitvi06g00798.t01 | MYB_related | | chr1.gff3_PROT_VIT_01s0026g01820.t01 | | SBP | |
| g10390.t1 | MYB_related | | Vitvi07g00452.t01 | MYB_related | | chr10.gff3_PROT_VIT_10s0003g00050.t01 | | SBP | |
| g10791.t1 | MYB_related | | Vitvi07g01899.t01 | MYB_related | | chr11.gff3_PROT_VIT_11s0065g00170.t01 | | SBP | |
| g10813.t1 | MYB_related | | Vitvi07g01900.t01 | MYB_related | | chr12.gff3_PROT_VIT_12s0028g03350.t01 | | SBP | |
| g11202.t1 | MYB_related | | Vitvi07g02253.t01 | MYB_related | | chr14.gff3_PROT_VIT_14s0068g01780.t01 | | SBP | |
| g11203.t1 | MYB_related | | Vitvi07g02627.t01 | MYB_related | | chr15.gff3_PROT_VIT_15s0021g02290.t01 | | SBP | |
| g11585.t1 | MYB_related | | Vitvi07g02629.t01 | MYB_related | | chr15.gff3_PROT_VIT_15s0021g02300.t01 | | SBP | |
| g12251.t1 | MYB_related | | Vitvi08g01149.t01 | MYB_related | | chr17.gff3_PROT_VIT_17s0000g01260.t01 | | SBP | |
| g1263.t1 | MYB_related | | Vitvi08g01213.t01 | MYB_related | | chr17.gff3_PROT_VIT_17s0000g05020.t01 | | SBP | |
| g12680.t1 | MYB_related | | Vitvi08g01875.t01 | MYB_related | | chr18.gff3_PROT_VIT_18s0122g00380.t01 | | SBP | |
| g13320.t1 | MYB_related | | Vitvi08g02140.t01 | MYB_related | | chr19.gff3_PROT_VIT_19s0014g02350.t01 | | SBP | |
| g13612.t1 | MYB_related | | Vitvi09g00122.t01 | MYB_related | | chr4.gff3_PROT_VIT_04s0210g00170.t01 | | SBP | |
| g13613.t1 | MYB_related | | Vitvi09g00293.t01 | MYB_related | | chr5.gff3_PROT_VIT_05s0020g00700.t01 | | SBP | |
| g13614.t1 | MYB_related | | Vitvi09g00300.t01 | MYB_related | | chr5.gff3_PROT_VIT_05s0020g02160.t01 | | SBP | |
| g13686.t1 | MYB_related | | Vitvi10g01608.t01 | MYB_related | | chr7.gff3_PROT_VIT_07s0005g02260.t01 | | SBP | |
| g13686.t2 | MYB_related | | Vitvi10g01641.t01 | MYB_related | | chr8.gff3_PROT_VIT_08s0007g06270.t01 | | SBP | |
| g1402.t1 | MYB_related | | Vitvi11g00149.t01 | MYB_related | | chr13_random.gff3_PROT_VIT_13s0067g02860.t01 | | SRS | |
| g14994.t1 | MYB_related | | Vitvi11g00197.t01 | MYB_related | | chr18.gff3_PROT_VIT_18s0001g13420.t01 | | SRS | |
| g15788.t1 | MYB_related | | Vitvi11g00498.t01 | MYB_related | | chr3.gff3_PROT_VIT_03s0038g00310.t01 | | SRS | |
| g15789.t1 | MYB_related | | Vitvi12g00527.t01 | MYB_related | | chr4.gff3_PROT_VIT_04s0023g02780.t01 | | SRS | |
| g15860.t1 | MYB_related | | Vitvi12g00771.t01 | MYB_related | | chr6.gff3_PROT_VIT_06s0009g03450.t01 | | SRS | |
| g1682.t1 | MYB_related | | Vitvi13g00310.t01 | MYB_related | | chr19.gff3_PROT_VIT_19s0014g00140.t01 | | STAT | |
| g16887.t1 | MYB_related | | Vitvi13g00842.t01 | MYB_related | | chr3.gff3_PROT_VIT_03s0038g00050.t01 | | TALE | |
| g17817.t1 | MYB_related | | Vitvi13g02569.t01 | MYB_related | | chr8.gff3_PROT_VIT_08s0105g00230.t01 | | TALE | |
| g20658.t1 | MYB_related | | Vitvi14g00230.t01 | MYB_related | | chr6.gff3_PROT_VIT_06s0009g00410.t01 | | TALE | |
| g20887.t1 | MYB_related | | Vitvi15g00870.t01 | MYB_related | | chr2.gff3_PROT_VIT_02s0025g00200.t01 | | TALE | |
| g20938.t1 | MYB_related | | Vitvi15g01002.t01 | MYB_related | | chr8.gff3_PROT_VIT_08s0007g01290.t01 | | TALE | |
| g21001.t1 | MYB_related | | Vitvi15g01056.t01 | MYB_related | | chr6.gff3_PROT_VIT_06s0004g02580.t01 | | TALE | |
| g21032.t1 | MYB_related | | Vitvi15g01097.t01 | MYB_related | | chr18.gff3_PROT_VIT_18s0001g13150.t01 | | TALE | |
| g21486.t1 | MYB_related | | Vitvi16g00100.t01 | MYB_related | | chr4.gff3_PROT_VIT_04s0023g01020.t01 | | TALE | |
| g21493.t1 | MYB_related | | Vitvi16g00102.t01 | MYB_related | | chr18.gff3_PROT_VIT_18s0001g13130.t01 | | TALE | |
| g22086.t2 | MYB_related | | Vitvi16g00129.t01 | MYB_related | | chr13.gff3_PROT_VIT_13s0019g02450.t01 | | TALE | |
| g22165.t1 | MYB_related | | Vitvi16g00303.t01 | MYB_related | | chr18.gff3_PROT_VIT_18s0001g11800.t01 | | TALE | |
| g22165.t2 | MYB_related | | Vitvi16g00305.t01 | MYB_related | | chr4.gff3_PROT_VIT_04s0008g00880.t01 | | TALE | |
| g23579.t1 | MYB_related | | Vitvi16g01106.t01 | MYB_related | | chr10_random.gff3_PROT_VIT_10s0116g00190.t01 | | TALE | |
| g23916.t1 | MYB_related | | Vitvi16g01215.t01 | MYB_related | | chr18.gff3_PROT_VIT_18s0001g08380.t01 | | TALE | |
| g23963.t1 | MYB_related | | Vitvi16g01449.t01 | MYB_related | | chr12.gff3_PROT_VIT_12s0059g01190.t01 | | TALE | |
| g24072.t1 | MYB_related | | Vitvi16g01513.t01 | MYB_related | | chr14.gff3_PROT_VIT_14s0060g01180.t01 | | TALE | |
| g24681.t1 | MYB_related | | Vitvi16g01515.t01 | MYB_related | | chr17.gff3_PROT_VIT_17s0000g08710.t01 | | TALE | |
| g24792.t1 | MYB_related | | Vitvi16g01520.t01 | MYB_related | | chr4.gff3_PROT_VIT_04s0008g06130.t01 | | TALE | |
| g24860.t2 | MYB_related | | Vitvi16g01521.t01 | MYB_related | | chr11.gff3_PROT_VIT_11s0118g00800.t01 | | TALE | |
| g24860.t3 | MYB_related | | Vitvi16g01524.t01 | MYB_related | | chr1.gff3_PROT_VIT_01s0127g00210.t01 | | TALE | |
| g25373.t1 | MYB_related | | Vitvi16g01525.t01 | MYB_related | | chr2.gff3_PROT_VIT_02s0025g04630.t01 | | TALE | |
| g25675.t1 | MYB_related | | Vitvi17g00726.t01 | MYB_related | | chr1.gff3_PROT_VIT_01s0011g02920.t01 | | TCP | |
| g25821.t1 | MYB_related | | Vitvi18g00967.t01 | MYB_related | | chr1.gff3_PROT_VIT_01s0026g02200.t01 | | TCP | |
| g2651.t1 | MYB_related | | Vitvi18g00973.t01 | MYB_related | | chr10.gff3_PROT_VIT_10s0003g00870.t01 | | TCP | |
| g26667.t1 | MYB_related | | Vitvi18g02612.t01 | MYB_related | | chr10.gff3_PROT_VIT_10s0003g03910.t01 | | TCP | |
| g2792.t1 | MYB_related | | Vitvi18g02938.t01 | MYB_related | | chr10.gff3_PROT_VIT_10s0042g00170.t01 | | TCP | |
| g27948.t1 | MYB_related | | Vitvi18g02951.t01 | MYB_related | | chr12.gff3_PROT_VIT_12s0028g02520.t01 | | TCP | |
| g27954.t1 | MYB_related | | Vitvi19g00741.t01 | MYB_related | | chr12.gff3_PROT_VIT_12s0035g00690.t01 | | TCP | |
| g27987.t1 | MYB_related | | Vitvi19g00743.t01 | MYB_related | | chr14.gff3_PROT_VIT_14s0068g00330.t01 | | TCP | |
| g28878.t1 | MYB_related | | Vitvi19g02081.t01 | MYB_related | | chr14.gff3_PROT_VIT_14s0068g01690.t01 | | TCP | |
| g29015.t1 | MYB_related | | Vitvi01g00237.t01 | NAC | | chr14.gff3_PROT_VIT_14s0083g00150.t01 | | TCP | |
| g29018.t1 | MYB_related | | Vitvi01g00999.t01 | NAC | | chr15.gff3_PROT_VIT_15s0048g01150.t01 | | TCP | |
| g29051.t1 | MYB_related | | Vitvi01g01038.t01 | NAC | | chr16.gff3_PROT_VIT_16s0022g02480.t01 | | TCP | |
| g29177.t1 | MYB_related | | Vitvi01g01722.t01 | NAC | | chr17.gff3_PROT_VIT_17s0000g04180.t01 | | TCP | |
| g30434.t1 | MYB_related | | Vitvi02g00242.t01 | NAC | | chr17.gff3_PROT_VIT_17s0000g06020.t01 | | TCP | |
| g30623.t1 | MYB_related | | Vitvi02g00277.t01 | NAC | | chr18.gff3_PROT_VIT_18s0117g00340.t01 | | TCP | |
| g30826.t1 | MYB_related | | Vitvi02g00508.t01 | NAC | | chr19.gff3_PROT_VIT_19s0014g01680.t01 | | TCP | |
| g30827.t1 | MYB_related | | Vitvi02g00673.t01 | NAC | | chr2.gff3_PROT_VIT_02s0025g04590.t01 | | TCP | |
| g30829.t1 | MYB_related | | Vitvi02g00998.t01 | NAC | | chr8.gff3_PROT_VIT_08s0040g01600.t01 | | TCP | |
| g30831.t1 | MYB_related | | Vitvi03g00207.t01 | NAC | | chr1.gff3_PROT_VIT_01s0010g02420.t01 | | Trihelix | |
| g30833.t1 | MYB_related | | Vitvi04g00236.t01 | NAC | | chr1.gff3_PROT_VIT_01s0150g00050.t01 | | Trihelix | |
| g30837.t1 | MYB_related | | Vitvi04g00592.t01 | NAC | | chr10.gff3_PROT_VIT_10s0003g01300.t01 | | Trihelix | |
| g30838.t1 | MYB_related | | Vitvi04g00837.t01 | NAC | | chr10.gff3_PROT_VIT_10s0042g00940.t01 | | Trihelix | |
| g30840.t1 | MYB_related | | Vitvi04g01430.t01 | NAC | | chr11.gff3_PROT_VIT_11s0016g04130.t01 | | Trihelix | |
| g30843.t1 | MYB_related | | Vitvi04g01511.t01 | NAC | | chr12.gff3_PROT_VIT_12s0028g02150.t01 | | Trihelix | |
| g30879.t1 | MYB_related | | Vitvi04g01684.t01 | NAC | | chr13.gff3_PROT_VIT_13s0067g01440.t01 | | Trihelix | |
| g31071.t1 | MYB_related | | Vitvi04g01714.t01 | NAC | | chr13.gff3_PROT_VIT_13s0074g00240.t01 | | Trihelix | |
| g31071.t2 | MYB_related | | Vitvi06g00007.t01 | NAC | | chr13.gff3_PROT_VIT_13s0074g00400.t01 | | Trihelix | |
| g31385.t1 | MYB_related | | Vitvi06g00305.t01 | NAC | | chr13.gff3_PROT_VIT_13s0084g00800.t01 | | Trihelix | |
| g31482.t1 | MYB_related | | Vitvi06g01384.t01 | NAC | | chr14.gff3_PROT_VIT_14s0030g01860.t01 | | Trihelix | |
| g31564.t1 | MYB_related | | Vitvi06g01515.t01 | NAC | | chr14.gff3_PROT_VIT_14s0171g00450.t01 | | Trihelix | |
| g31618.t1 | MYB_related | | Vitvi06g01536.t01 | NAC | | chr15.gff3_PROT_VIT_15s0021g01220.t01 | | Trihelix | |
| g31773.t1 | MYB_related | | Vitvi06g01638.t01 | NAC | | chr16.gff3_PROT_VIT_16s0022g02290.t01 | | Trihelix | |
| g31985.t1 | MYB_related | | Vitvi06g01639.t01 | NAC | | chr17.gff3_PROT_VIT_17s0000g00270.t01 | | Trihelix | |
| g3316.t1 | MYB_related | | Vitvi06g01641.t01 | NAC | | chr17.gff3_PROT_VIT_17s0000g01640.t01 | | Trihelix | |
| g3427.t1 | MYB_related | | Vitvi06g01738.t01 | NAC | | chr17.gff3_PROT_VIT_17s0000g10420.t01 | | Trihelix | |
| g34640.t1 | MYB_related | | Vitvi07g00619.t01 | NAC | | chr18.gff3_PROT_VIT_18s0072g00670.t01 | | Trihelix | |
| g35128.t1 | MYB_related | | Vitvi07g01929.t01 | NAC | | chr18.gff3_PROT_VIT_18s0072g00690.t01 | | Trihelix | |
| g36017.t1 | MYB_related | | Vitvi07g02163.t01 | NAC | | chr2.gff3_PROT_VIT_02s0025g03220.t01 | | Trihelix | |
| g36145.t1 | MYB_related | | Vitvi08g01052.t01 | NAC | | chr2.gff3_PROT_VIT_02s0087g00140.t01 | | Trihelix | |
| g36446.t1 | MYB_related | | Vitvi08g01426.t01 | NAC | | chr4.gff3_PROT_VIT_04s0008g01850.t01 | | Trihelix | |
| g36610.t1 | MYB_related | | Vitvi08g01841.t01 | NAC | | chr4.gff3_PROT_VIT_04s0008g02870.t01 | | Trihelix | |
| g36927.t1 | MYB_related | | Vitvi08g01843.t01 | NAC | | chr4.gff3_PROT_VIT_04s0044g00510.t01 | | Trihelix | |
| g37395.t1 | MYB_related | | Vitvi08g02399.t01 | NAC | | chr6.gff3_PROT_VIT_06s0004g01900.t01 | | Trihelix | |
| g37482.t1 | MYB_related | | Vitvi09g01309.t01 | NAC | | chr7.gff3_PROT_VIT_07s0005g06670.t01 | | Trihelix | |
| g37555.t1 | MYB_related | | Vitvi10g00437.t01 | NAC | | chr7.gff3_PROT_VIT_07s0031g02800.t01 | | Trihelix | |
| g37763.t1 | MYB_related | | Vitvi10g00505.t01 | NAC | | chr8.gff3_PROT_VIT_08s0007g03820.t01 | | Trihelix | |
| g37766.t1 | MYB_related | | Vitvi10g00507.t01 | NAC | | chr8.gff3_PROT_VIT_08s0007g04160.t01 | | Trihelix | |
| g37843.t1 | MYB_related | | Vitvi11g00241.t01 | NAC | | chr8.gff3_PROT_VIT_08s0007g04180.t01 | | Trihelix | |
| g3810.t1 | MYB_related | | Vitvi12g00076.t01 | NAC | | chr8.gff3_PROT_VIT_08s0058g00200.t01 | | Trihelix | |
| g38222.t1 | MYB_related | | Vitvi12g00255.t01 | NAC | | chr8.gff3_PROT_VIT_08s0105g00400.t01 | | Trihelix | |
| g38260.t1 | MYB_related | | Vitvi12g01655.t01 | NAC | | chr9.gff3_PROT_VIT_09s0002g00720.t01 | | Trihelix | |
| g38310.t1 | MYB_related | | Vitvi12g02150.t01 | NAC | | chr9.gff3_PROT_VIT_09s0002g04700.t01 | | Trihelix | |
| g5062.t1 | MYB_related | | Vitvi13g00698.t01 | NAC | | chrUn.gff3_PROT_VIT_00s0204g00020.t01 | | Trihelix | |
| g5857.t1 | MYB_related | | Vitvi13g00699.t01 | NAC | | chrUn.gff3_PROT_VIT_00s0259g00210.t01 | | Trihelix | |
| g5966.t1 | MYB_related | | Vitvi13g01073.t01 | NAC | | chrUn.gff3_PROT_VIT_00s0956g00030.t01 | | Trihelix | |
| g6114.t1 | MYB_related | | Vitvi14g01499.t01 | NAC | | chr10.gff3_PROT_VIT_10s0003g00500.t01 | | VOZ | |
| g6196.t1 | MYB_related | | Vitvi14g01678.t01 | NAC | | chr12.gff3_PROT_VIT_12s0028g02670.t01 | | VOZ | |
| g7947.t1 | MYB_related | | Vitvi14g01963.t01 | NAC | | chr1.gff3_PROT_VIT_01s0026g01720.t01 | | Whirly | |
| g7954.t1 | MYB_related | | Vitvi14g01985.t01 | NAC | | chr18.gff3_PROT_VIT_18s0089g00900.t01 | | Whirly | |
| g815.t1 | MYB_related | | Vitvi15g00889.t01 | NAC | | chr6.gff3_PROT_VIT_06s0004g03780.t01 | | WOX | |
| g815.t2 | MYB_related | | Vitvi15g01539.t01 | NAC | | chr18.gff3_PROT_VIT_18s0001g10160.t01 | | WOX | |
| g821.t1 | MYB_related | | Vitvi15g01540.t01 | NAC | | chr17.gff3_PROT_VIT_17s0000g02460.t01 | | WOX | |
| g8536.t1 | MYB_related | | Vitvi15g01541.t01 | NAC | | chr13.gff3_PROT_VIT_13s0019g03460.t01 | | WOX | |
| g8695.t1 | MYB_related | | Vitvi15g01542.t01 | NAC | | chr4.gff3_PROT_VIT_04s0023g03310.t01 | | WOX | |
| g8701.t1 | MYB_related | | Vitvi15g01543.t01 | NAC | | chr1.gff3_PROT_VIT_01s0011g05020.t01 | | WOX | |
| g9018.t1 | MYB_related | | Vitvi15g01544.t01 | NAC | | chr10.gff3_PROT_VIT_10s0003g00560.t01 | | WOX | |
| g10568.t1 | NAC | | Vitvi15g01545.t01 | NAC | | chr8.gff3_PROT_VIT_08s0056g01200.t01 | | WOX | |
| g10719.t1 | NAC | | Vitvi15g01546.t01 | NAC | | chr18.gff3_PROT_VIT_18s0122g01140.t01 | | WOX | |
| g10878.t1 | NAC | | Vitvi16g00716.t01 | NAC | | chr4.gff3_PROT_VIT_04s0044g00150.t01 | | WOX | |
| g11758.t1 | NAC | | Vitvi16g01327.t01 | NAC | | chr4.gff3_PROT_VIT_04s0044g00160.t01 | | WOX | |
| g12584.t1 | NAC | | Vitvi17g00066.t01 | NAC | | chr1.gff3_PROT_VIT_01s0010g03930.t01 | | WRKY | |
| g1285.t1 | NAC | | Vitvi17g00316.t01 | NAC | | chr1.gff3_PROT_VIT_01s0011g00720.t01 | | WRKY | |
| g13434.t1 | NAC | | Vitvi17g00622.t01 | NAC | | chr1.gff3_PROT_VIT_01s0026g01730.t01 | | WRKY | |
| g14440.t1 | NAC | | Vitvi17g00685.t01 | NAC | | chr10.gff3_PROT_VIT_10s0003g01600.t01 | | WRKY | |
| g14443.t1 | NAC | | Vitvi17g01227.t01 | NAC | | chr10.gff3_PROT_VIT_10s0003g02810.t01 | | WRKY | |
| g14444.t1 | NAC | | Vitvi18g00052.t01 | NAC | | chr10.gff3_PROT_VIT_10s0003g05740.t01 | | WRKY | |
| g14445.t1 | NAC | | Vitvi18g00250.t01 | NAC | | chr10.gff3_PROT_VIT_10s0116g01200.t01 | | WRKY | |
| g14446.t1 | NAC | | Vitvi18g00761.t01 | NAC | | chr11.gff3_PROT_VIT_11s0037g00150.t01 | | WRKY | |
| g14449.t1 | NAC | | Vitvi18g01520.t01 | NAC | | chr11.gff3_PROT_VIT_11s0052g00450.t01 | | WRKY | |
| g14620.t1 | NAC | | Vitvi18g01684.t01 | NAC | | chr12.gff3_PROT_VIT_12s0028g00270.t01 | | WRKY | |
| g14708.t1 | NAC | | Vitvi18g02011.t01 | NAC | | chr12.gff3_PROT_VIT_12s0055g00340.t01 | | WRKY | |
| g1500.t1 | NAC | | Vitvi18g02404.t01 | NAC | | chr12.gff3_PROT_VIT_12s0057g00550.t01 | | WRKY | |
| g15249.t1 | NAC | | Vitvi18g02732.t01 | NAC | | chr12.gff3_PROT_VIT_12s0059g00880.t01 | | WRKY | |
| g15250.t1 | NAC | | Vitvi19g00188.t01 | NAC | | chr13.gff3_PROT_VIT_13s0067g03130.t01 | | WRKY | |
| g15362.t1 | NAC | | Vitvi19g00270.t01 | NAC | | chr13.gff3_PROT_VIT_13s0067g03140.t01 | | WRKY | |
| g15650.t1 | NAC | | Vitvi19g00271.t01 | NAC | | chr14.gff3_PROT_VIT_14s0068g01770.t01 | | WRKY | |
| g15690.t1 | NAC | | Vitvi19g01484.t01 | NAC | | chr14.gff3_PROT_VIT_14s0081g00560.t01 | | WRKY | |
| g15945.t1 | NAC | | Vitvi19g01561.t01 | NAC | | chr14.gff3_PROT_VIT_14s0108g00120.t01 | | WRKY | |
| g16061.t1 | NAC | | Vitvi19g01564.t01 | NAC | | chr14.gff3_PROT_VIT_14s0108g01280.t01 | | WRKY | |
| g16255.t1 | NAC | | Vitvi19g01566.t01 | NAC | | chr15.gff3_PROT_VIT_15s0021g01310.t01 | | WRKY | |
| g16276.t1 | NAC | | Vitvi19g01647.t01 | NAC | | chr15.gff3_PROT_VIT_15s0046g01140.t01 | | WRKY | |
| g16322.t1 | NAC | | Vitvi19g01751.t01 | NAC | | chr15.gff3_PROT_VIT_15s0046g02150.t01 | | WRKY | |
| g16322.t2 | NAC | | Vitvi19g01778.t01 | NAC | | chr15.gff3_PROT_VIT_15s0046g02190.t01 | | WRKY | |
| g16349.t1 | NAC | | Vitvi19g02272.t01 | NAC | | chr16.gff3_PROT_VIT_16s0050g01480.t01 | | WRKY | |
| g16397.t1 | NAC | | Vitvi19g02273.t01 | NAC | | chr16.gff3_PROT_VIT_16s0050g02510.t01 | | WRKY | |
| g17320.t1 | NAC | | Vitvi13g00049.t01 | NF-X1 | | chr17.gff3_PROT_VIT_17s0000g01280.t01 | | WRKY | |
| g17767.t1 | NAC | | Vitvi17g00161.t01 | NF-X1 | | chr17.gff3_PROT_VIT_17s0000g05810.t01 | | WRKY | |
| g17769.t1 | NAC | | Vitvi19g00036.t01 | NF-X1 | | chr18.gff3_PROT_VIT_18s0001g10030.t01 | | WRKY | |
| g17845.t1 | NAC | | Vitvi06g00224.t01 | NF-YA | | chr19.gff3_PROT_VIT_19s0015g01870.t01 | | WRKY | |
| g18085.t1 | NAC | | Vitvi08g00292.t01 | NF-YA | | chr19.gff3_PROT_VIT_19s0090g00840.t01 | | WRKY | |
| g18618.t1 | NAC | | Vitvi08g01883.t01 | NF-YA | | chr19.gff3_PROT_VIT_19s0090g01720.t01 | | WRKY | |
| g18646.t1 | NAC | | Vitvi09g00133.t01 | NF-YA | | chr1_random.gff3_PROT_VIT_01s0011g00220.t01 | | WRKY | |
| g19064.t1 | NAC | | Vitvi10g00911.t01 | NF-YA | | chr2.gff3_PROT_VIT_02s0025g00420.t01 | | WRKY | |
| g19065.t1 | NAC | | Vitvi11g00109.t01 | NF-YA | | chr2.gff3_PROT_VIT_02s0025g01280.t01 | | WRKY | |
| g19281.t1 | NAC | | Vitvi13g01729.t01 | NF-YA | | chr2.gff3_PROT_VIT_02s0154g00210.t01 | | WRKY | |
| g19913.t1 | NAC | | Vitvi01g01464.t01 | NF-YB | | chr4.gff3_PROT_VIT_04s0008g01470.t01 | | WRKY | |
| g19992.t1 | NAC | | Vitvi01g01468.t01 | NF-YB | | chr4.gff3_PROT_VIT_04s0008g05750.t01 | | WRKY | |
| g20023.t1 | NAC | | Vitvi05g00299.t01 | NF-YB | | chr4.gff3_PROT_VIT_04s0008g05760.t01 | | WRKY | |
| g20094.t1 | NAC | | Vitvi06g00351.t01 | NF-YB | | chr4.gff3_PROT_VIT_04s0008g06600.t01 | | WRKY | |
| g20095.t1 | NAC | | Vitvi06g01492.t01 | NF-YB | | chr4.gff3_PROT_VIT_04s0023g00470.t01 | | WRKY | |
| g20192.t1 | NAC | | Vitvi07g00273.t01 | NF-YB | | chr4.gff3_PROT_VIT_04s0069g00920.t01 | | WRKY | |
| g21200.t1 | NAC | | Vitvi07g01823.t01 | NF-YB | | chr4.gff3_PROT_VIT_04s0069g00970.t01 | | WRKY | |
| g21236.t1 | NAC | | Vitvi08g01154.t01 | NF-YB | | chr4.gff3_PROT_VIT_04s0069g00980.t01 | | WRKY | |
| g21613.t1 | NAC | | Vitvi10g00251.t01 | NF-YB | | chr5.gff3_PROT_VIT_05s0077g00730.t01 | | WRKY | |
| g22605.t1 | NAC | | Vitvi10g02273.t01 | NF-YB | | chr6.gff3_PROT_VIT_06s0004g00230.t01 | | WRKY | |
| g23242.t1 | NAC | | Vitvi10g02311.t01 | NF-YB | | chr6.gff3_PROT_VIT_06s0004g07500.t01 | | WRKY | |
| g24131.t1 | NAC | | Vitvi12g00719.t01 | NF-YB | | chr7.gff3_PROT_VIT_07s0005g01520.t01 | | WRKY | |
| g24727.t1 | NAC | | Vitvi13g00535.t01 | NF-YB | | chr7.gff3_PROT_VIT_07s0005g01710.t01 | | WRKY | |
| g25115.t1 | NAC | | Vitvi14g00217.t01 | NF-YB | | chr7.gff3_PROT_VIT_07s0005g02570.t01 | | WRKY | |
| g25401.t1 | NAC | | Vitvi17g01421.t01 | NF-YB | | chr7.gff3_PROT_VIT_07s0031g00080.t01 | | WRKY | |
| g25548.t1 | NAC | | Vitvi19g00673.t01 | NF-YB | | chr7.gff3_PROT_VIT_07s0031g01710.t01 | | WRKY | |
| g26653.t1 | NAC | | Vitvi19g00686.t01 | NF-YB | | chr7.gff3_PROT_VIT_07s0031g01840.t01 | | WRKY | |
| g26654.t1 | NAC | | Vitvi19g02067.t01 | NF-YB | | chr7.gff3_PROT_VIT_07s0141g00680.t01 | | WRKY | |
| g26655.t1 | NAC | | Vitvi01g01642.t01 | NF-YC | | chr8.gff3_PROT_VIT_08s0007g00570.t01 | | WRKY | |
| g26656.t1 | NAC | | Vitvi02g00347.t01 | NF-YC | | chr8.gff3_PROT_VIT_08s0040g03070.t01 | | WRKY | |
| g26656.t2 | NAC | | Vitvi05g00435.t01 | NF-YC | | chr8.gff3_PROT_VIT_08s0058g00690.t01 | | WRKY | |
| g26657.t1 | NAC | | Vitvi06g01109.t01 | NF-YC | | chr8.gff3_PROT_VIT_08s0058g01390.t01 | | WRKY | |
| g26659.t1 | NAC | | Vitvi06g01659.t01 | NF-YC | | chr9.gff3_PROT_VIT_09s0018g00240.t01 | | WRKY | |
| g26660.t1 | NAC | | Vitvi13g01402.t01 | NF-YC | | chrUn.gff3_PROT_VIT_00s0463g00010.t01 | | WRKY | |
| g26695.t1 | NAC | | Vitvi14g00264.t01 | NF-YC | | chr1.gff3_PROT_VIT_01s0011g00140.t01 | | YABBY | |
| g26843.t1 | NAC | | Vitvi14g02861.t01 | NF-YC | | chr1.gff3_PROT_VIT_01s0127g00330.t01 | | YABBY | |
| g27076.t1 | NAC | | Vitvi17g00088.t01 | NF-YC | | chr11.gff3_PROT_VIT_11s0016g05590.t01 | | YABBY | |
| g27099.t1 | NAC | | Vitvi19g01705.t01 | NF-YC | | chr15.gff3_PROT_VIT_15s0048g00550.t01 | | YABBY | |
| g2741.t1 | NAC | | Vitvi02g00180.t01 | Nin-like | | chr2.gff3_PROT_VIT_02s0154g00070.t01 | | YABBY | |
| g28110.t1 | NAC | | Vitvi03g00231.t01 | Nin-like | | chr6.gff3_PROT_VIT_06s0009g00880.t01 | | YABBY | |
| g29052.t1 | NAC | | Vitvi04g00143.t01 | Nin-like | | chr8.gff3_PROT_VIT_08s0032g01110.t01 | | YABBY | |
| g29218.t1 | NAC | | Vitvi04g01569.t01 | Nin-like | | chr1.gff3_PROT_VIT_01s0011g06360.t01 | | ZF-HD | |
| g29331.t1 | NAC | | Vitvi05g01560.t01 | Nin-like | | chr1.gff3_PROT_VIT_01s0026g02450.t01 | | ZF-HD | |
| g29461.t1 | NAC | | Vitvi12g00055.t01 | Nin-like | | chr1.gff3_PROT_VIT_01s0026g02460.t01 | | ZF-HD | |
| g29998.t1 | NAC | | Vitvi16g01402.t01 | Nin-like | | chr12.gff3_PROT_VIT_12s0035g01880.t01 | | ZF-HD | |
| g30383.t1 | NAC | | Vitvi17g00605.t01 | Nin-like | | chr14.gff3_PROT_VIT_14s0108g00750.t01 | | ZF-HD | |
| g31134.t1 | NAC | | Vitvi18g02488.t01 | Nin-like | | chr14.gff3_PROT_VIT_14s0108g00760.t01 | | ZF-HD | |
| g31842.t1 | NAC | | Vitvi19g00320.t01 | NZZ/SPL | | chr14.gff3_PROT_VIT_14s0108g00810.t01 | | ZF-HD | |
| g3256.t1 | NAC | | Vitvi19g01844.t01 | NZZ/SPL | | chr17.gff3_PROT_VIT_17s0000g06200.t01 | | ZF-HD | |
| g32932.t1 | NAC | | Vitvi01g00244.t01 | RAV | | chr17_random.gff3_PROT_VIT_17s0000g00810.t01 | | ZF-HD | |
| g32936.t1 | NAC | | Vitvi14g01248.t01 | RAV | | chr18.gff3_PROT_VIT_18s0001g07140.t01 | | ZF-HD | |
| g34404.t1 | NAC | | Vitvi02g01775.t01 | RAV | | chr18.gff3_PROT_VIT_18s0001g12580.t01 | | ZF-HD | |
| g3513.t1 | NAC | | Vitvi11g00678.t01 | RAV | | chr18.gff3_PROT_VIT_18s0117g00270.t01 | | ZF-HD | |
| g35180.t1 | NAC | | Vitvi06g01662.t01 | S1Fa-like | | chr4.gff3_PROT_VIT_04s0023g01880.t01 | | ZF-HD | |
| g35509.t1 | NAC | | Vitvi08g02161.t01 | S1Fa-like | | chr8.gff3_PROT_VIT_08s0056g01130.t01 | | ZF-HD | |
| g35514.t1 | NAC | | Vitvi12g00707.t01 | SAP | | chrUn.gff3_PROT_VIT_00s0199g00250.t01 | | ZF-HD | |
| g35515.t1 | NAC | | Vitvi01g01660.t01 | SBP | |  | |  | |
| g35516.t1 | NAC | | Vitvi01g01678.t01 | SBP | |  | |  | |
| g36187.t1 | NAC | | Vitvi01g01837.t01 | SBP | |  | |  | |
| g36314.t1 | NAC | | Vitvi01g02117.t01 | SBP | |  | |  | |
| g37143.t1 | NAC | | Vitvi04g01556.t01 | SBP | |  | |  | |
| g38214.t1 | NAC | | Vitvi05g00245.t01 | SBP | |  | |  | |
| g4719.t1 | NAC | | Vitvi05g00376.t01 | SBP | |  | |  | |
| g6554.t1 | NAC | | Vitvi07g00491.t01 | SBP | |  | |  | |
| g7268.t1 | NAC | | Vitvi08g01720.t01 | SBP | |  | |  | |
| g763.t1 | NAC | | Vitvi10g00481.t01 | SBP | |  | |  | |
| g8089.t1 | NAC | | Vitvi11g00909.t01 | SBP | |  | |  | |
| g9136.t1 | NAC | | Vitvi12g00280.t01 | SBP | |  | |  | |
| g9210.t1 | NAC | | Vitvi14g01524.t01 | SBP | |  | |  | |
| g9597.t1 | NAC | | Vitvi15g00619.t01 | SBP | |  | |  | |
| g26805.t1 | NF-X1 | | Vitvi15g00620.t01 | SBP | |  | |  | |
| g29633.t1 | NF-X1 | | Vitvi17g00100.t01 | SBP | |  | |  | |
| g14458.t1 | NF-YA | | Vitvi17g00473.t01 | SBP | |  | |  | |
| g17823.t1 | NF-YA | | Vitvi18g00027.t01 | SBP | |  | |  | |
| g18396.t1 | NF-YA | | Vitvi19g00200.t01 | SBP | |  | |  | |
| g22319.t1 | NF-YA | | Vitvi02g00197.t01 | SRS | |  | |  | |
| g22319.t2 | NF-YA | | Vitvi03g00024.t01 | SRS | |  | |  | |
| g22867.t1 | NF-YA | | Vitvi04g01399.t01 | SRS | |  | |  | |
| g31060.t1 | NF-YA | | Vitvi06g01239.t01 | SRS | |  | |  | |
| g32010.t1 | NF-YA | | Vitvi13g01413.t01 | SRS | |  | |  | |
| g1472.t1 | NF-YB | | Vitvi18g01041.t01 | SRS | |  | |  | |
| g16301.t1 | NF-YB | | Vitvi19g00013.t01 | STAT | |  | |  | |
| g17072.t1 | NF-YB | | Vitvi03g00004.t01 | TALE | |  | |  | |
| g2029.t1 | NF-YB | | Vitvi08g00633.t01 | TALE | |  | |  | |
| g2032.t1 | NF-YB | | Vitvi06g00918.t01 | TALE | |  | |  | |
| g20386.t1 | NF-YB | | Vitvi02g00016.t01 | TALE | |  | |  | |
| g22524.t1 | NF-YB | | Vitvi08g01274.t01 | TALE | |  | |  | |
| g24796.t1 | NF-YB | | Vitvi06g00248.t01 | TALE | |  | |  | |
| g24796.t2 | NF-YB | | Vitvi18g00902.t01 | TALE | |  | |  | |
| g24796.t3 | NF-YB | | Vitvi18g01016.t01 | TALE | |  | |  | |
| g26037.t1 | NF-YB | | Vitvi04g01210.t01 | TALE | |  | |  | |
| g2622.t1 | NF-YB | | Vitvi18g02768.t01 | TALE | |  | |  | |
| g3078.t1 | NF-YB | | Vitvi13g00366.t01 | TALE | |  | |  | |
| g31668.t1 | NF-YB | | Vitvi04g00072.t01 | TALE | |  | |  | |
| g31681.t1 | NF-YB | | Vitvi10g00124.t01 | TALE | |  | |  | |
| g31682.t1 | NF-YB | | Vitvi18g00602.t01 | TALE | |  | |  | |
| g31889.t1 | NF-YB | | Vitvi12g00424.t01 | TALE | |  | |  | |
| g33192.t1 | NF-YB | | Vitvi14g00093.t01 | TALE | |  | |  | |
| g34518.t1 | NF-YB | | Vitvi17g00850.t01 | TALE | |  | |  | |
| g34520.t1 | NF-YB | | Vitvi04g00546.t01 | TALE | |  | |  | |
| g37268.t1 | NF-YB | | Vitvi11g00601.t01 | TALE | |  | |  | |
| g11852.t1 | NF-YC | | Vitvi01g00694.t01 | TALE | |  | |  | |
| g16382.t1 | NF-YC | | Vitvi02g00425.t01 | TALE | |  | |  | |
| g16643.t1 | NF-YC | | Vitvi01g00228.t01 | TCP | |  | |  | |
| g21310.t1 | NF-YC | | Vitvi01g00980.t01 | TCP | |  | |  | |
| g21937.t1 | NF-YC | | Vitvi02g00421.t01 | TCP | |  | |  | |
| g2888.t1 | NF-YC | | Vitvi06g01646.t01 | TCP | |  | |  | |
| g30575.t1 | NF-YC | | Vitvi08g01011.t01 | TCP | |  | |  | |
| g31770.t1 | NF-YC | | Vitvi10g00540.t01 | TCP | |  | |  | |
| g31838.t1 | NF-YC | | Vitvi10g00838.t01 | TCP | |  | |  | |
| g35640.t1 | NF-YC | | Vitvi10g01466.t01 | TCP | |  | |  | |
| g13459.t1 | Nin-like | | Vitvi12g00219.t01 | TCP | |  | |  | |
| g18495.t1 | Nin-like | | Vitvi12g02006.t01 | TCP | |  | |  | |
| g18495.t2 | Nin-like | | Vitvi13g02208.t01 | TCP | |  | |  | |
| g23837.t1 | Nin-like | | Vitvi14g01253.t01 | TCP | |  | |  | |
| g28060.t1 | Nin-like | | Vitvi14g01398.t01 | TCP | |  | |  | |
| g2917.t1 | Nin-like | | Vitvi14g01519.t01 | TCP | |  | |  | |
| g29370.t1 | Nin-like | | Vitvi15g00765.t01 | TCP | |  | |  | |
| g3409.t1 | Nin-like | | Vitvi16g01833.t01 | TCP | |  | |  | |
| g34366.t1 | Nin-like | | Vitvi16g01846.t01 | TCP | |  | |  | |
| g576.t1 | Nin-like | | Vitvi17g00374.t01 | TCP | |  | |  | |
| g28365.t1 | NZZ/SPL | | Vitvi17g00495.t01 | TCP | |  | |  | |
| g16056.t1 | RAV | | Vitvi17g00581.t01 | TCP | |  | |  | |
| g18212.t1 | RAV | | Vitvi18g01875.t01 | TCP | |  | |  | |
| g7473.t1 | RAV | | Vitvi19g00140.t01 | TCP | |  | |  | |
| g11825.t1 | RAV | | Vitvi01g01477.t01 | Trihelix | |  | |  | |
| g2730.t1 | S1Fa-like | | Vitvi01g01533.t01 | Trihelix | |  | |  | |
| g31856.t1 | S1Fa-like | | Vitvi01g02298.t01 | Trihelix | |  | |  | |
| g22513.t1 | SAP | | Vitvi02g00291.t01 | Trihelix | |  | |  | |
| g10762.t1 | SBP | | Vitvi02g01203.t01 | Trihelix | |  | |  | |
| g10837.t1 | SBP | | Vitvi02g01753.t01 | Trihelix | |  | |  | |
| g13001.t1 | SBP | | Vitvi04g00253.t01 | Trihelix | |  | |  | |
| g15125.t1 | SBP | | Vitvi04g01604.t01 | Trihelix | |  | |  | |
| g15348.t1 | SBP | | Vitvi04g01808.t01 | Trihelix | |  | |  | |
| g17639.t1 | SBP | | Vitvi06g00200.t01 | Trihelix | |  | |  | |
| g18478.t1 | SBP | | Vitvi07g00941.t01 | Trihelix | |  | |  | |
| g21409.t1 | SBP | | Vitvi07g01957.t01 | Trihelix | |  | |  | |
| g23138.t1 | SBP | | Vitvi08g00658.t01 | Trihelix | |  | |  | |
| g25089.t1 | SBP | | Vitvi08g00730.t01 | Trihelix | |  | |  | |
| g2562.t1 | SBP | | Vitvi08g01505.t01 | Trihelix | |  | |  | |
| g25766.t1 | SBP | | Vitvi08g01538.t01 | Trihelix | |  | |  | |
| g28778.t1 | SBP | | Vitvi08g01540.t01 | Trihelix | |  | |  | |
| g29249.t1 | SBP | | Vitvi09g00393.t01 | Trihelix | |  | |  | |
| g29974.t1 | SBP | | Vitvi09g01306.t01 | Trihelix | |  | |  | |
| g33761.t1 | SBP | | Vitvi09g01496.t01 | Trihelix | |  | |  | |
| g33762.t1 | SBP | | Vitvi10g00254.t01 | Trihelix | |  | |  | |
| g33762.t2 | SBP | | Vitvi10g00584.t01 | Trihelix | |  | |  | |
| g34437.t1 | SBP | | Vitvi10g01388.t01 | Trihelix | |  | |  | |
| g36888.t1 | SBP | | Vitvi10g02271.t01 | Trihelix | |  | |  | |
| g5654.t1 | SBP | | Vitvi11g00354.t01 | Trihelix | |  | |  | |
| g13506.t1 | SRS | | Vitvi12g00182.t01 | Trihelix | |  | |  | |
| g15160.t1 | SRS | | Vitvi13g00084.t01 | Trihelix | |  | |  | |
| g25578.t1 | SRS | | Vitvi13g00728.t01 | Trihelix | |  | |  | |
| g28946.t1 | SRS | | Vitvi13g00740.t01 | Trihelix | |  | |  | |
| g899.t1 | SRS | | Vitvi13g01340.t01 | Trihelix | |  | |  | |
| g33924.t1 | STAT | | Vitvi14g00433.t01 | Trihelix | |  | |  | |
| g15144.t1 | TALE | | Vitvi14g01602.t01 | Trihelix | |  | |  | |
| g20251.t1 | TALE | | Vitvi15g00532.t01 | Trihelix | |  | |  | |
| g33945.t1 | TALE | | Vitvi16g00887.t01 | Trihelix | |  | |  | |
| g27216.t1 | TALE | | Vitvi17g00029.t01 | Trihelix | |  | |  | |
| g13710.t1 | TALE | | Vitvi17g00132.t01 | Trihelix | |  | |  | |
| g31785.t1 | TALE | | Vitvi17g01050.t01 | Trihelix | |  | |  | |
| g750.t1 | TALE | | Vitvi18g01649.t01 | Trihelix | |  | |  | |
| g876.t1 | TALE | | Vitvi18g01652.t01 | Trihelix | |  | |  | |
| g875.t1 | TALE | | Vitvi10g00515.t01 | VOZ | |  | |  | |
| g21114.t1 | TALE | | Vitvi12g00235.t01 | VOZ | |  | |  | |
| g3314.t1 | TALE | | Vitvi01g00938.t01 | Whirly | |  | |  | |
| g31510.t1 | TALE | | Vitvi18g02389.t01 | Whirly | |  | |  | |
| g17914.t1 | TALE | | Vitvi06g00380.t01 | WOX | |  | |  | |
| g23471.t1 | TALE | | Vitvi18g00757.t01 | WOX | |  | |  | |
| g11685.t1 | TALE | | Vitvi17g00216.t01 | WOX | |  | |  | |
| g28385.t1 | TALE | | Vitvi06g00445.t01 | WOX | |  | |  | |
| g22760.t2 | TALE | | Vitvi04g01449.t01 | WOX | |  | |  | |
| g22760.t1 | TALE | | Vitvi13g02039.t01 | WOX | |  | |  | |
| g12524.t1 | TALE | | Vitvi01g00427.t01 | WOX | |  | |  | |
| g26129.t1 | TALE | | Vitvi10g00519.t01 | WOX | |  | |  | |
| g266.t1 | TALE | | Vitvi08g00108.t01 | WOX | |  | |  | |
| g10503.t1 | TCP | | Vitvi18g00084.t01 | WOX | |  | |  | |
| g14431.t1 | TCP | | Vitvi04g01578.t01 | WOX | |  | |  | |
| g16067.t1 | TCP | | Vitvi04g01579.t01 | WOX | |  | |  | |
| g16449.t1 | TCP | | Vitvi01g00940.t01 | WRKY | |  | |  | |
| g18213.t1 | TCP | | Vitvi01g01680.t01 | WRKY | |  | |  | |
| g22074.t1 | TCP | | Vitvi01g01844.t01 | WRKY | |  | |  | |
| g23724.t1 | TCP | | Vitvi01g02157.t01 | WRKY | |  | |  | |
| g26018.t1 | TCP | | Vitvi02g00039.t01 | WRKY | |  | |  | |
| g262.t1 | TCP | | Vitvi02g00114.t01 | WRKY | |  | |  | |
| g2795.t2 | TCP | | Vitvi02g01435.t01 | WRKY | |  | |  | |
| g29980.t1 | TCP | | Vitvi02g01847.t01 | WRKY | |  | |  | |
| g30379.t1 | TCP | | Vitvi04g00133.t01 | WRKY | |  | |  | |
| g3218.t1 | TCP | | Vitvi04g00510.t01 | WRKY | |  | |  | |
| g32221.t1 | TCP | | Vitvi04g00511.t01 | WRKY | |  | |  | |
| g32985.t1 | TCP | | Vitvi04g00596.t01 | WRKY | |  | |  | |
| g34644.t1 | TCP | | Vitvi04g00756.t01 | WRKY | |  | |  | |
| g34724.t1 | TCP | | Vitvi04g00760.t01 | WRKY | |  | |  | |
| g34783.t1 | TCP | | Vitvi04g01163.t01 | WRKY | |  | |  | |
| g553.t1 | TCP | | Vitvi04g01985.t01 | WRKY | |  | |  | |
| g14497.t1 | Trihelix | | Vitvi05g00145.t01 | WRKY | |  | |  | |
| g14689.t1 | Trihelix | | Vitvi06g00741.t01 | WRKY | |  | |  | |
| g15135.t1 | Trihelix | | Vitvi06g01574.t01 | WRKY | |  | |  | |
| g15135.t2 | Trihelix | | Vitvi07g00026.t01 | WRKY | |  | |  | |
| g15189.t1 | Trihelix | | Vitvi07g00421.t01 | WRKY | |  | |  | |
| g15501.t1 | Trihelix | | Vitvi07g00434.t01 | WRKY | |  | |  | |
| g16489.t1 | Trihelix | | Vitvi07g00523.t01 | WRKY | |  | |  | |
| g17159.t1 | Trihelix | | Vitvi07g01694.t01 | WRKY | |  | |  | |
| g17220.t1 | Trihelix | | Vitvi07g01847.t01 | WRKY | |  | |  | |
| g17222.t1 | Trihelix | | Vitvi07g01860.t01 | WRKY | |  | |  | |
| g17413.t1 | Trihelix | | Vitvi08g00793.t01 | WRKY | |  | |  | |
| g17450.t1 | Trihelix | | Vitvi08g00868.t01 | WRKY | |  | |  | |
| g17453.t1 | Trihelix | | Vitvi08g01134.t01 | WRKY | |  | |  | |
| g18549.t1 | Trihelix | | Vitvi08g01221.t01 | WRKY | |  | |  | |
| g20271.t1 | Trihelix | | Vitvi09g01122.t01 | WRKY | |  | |  | |
| g2030.t1 | Trihelix | | Vitvi10g00063.t01 | WRKY | |  | |  | |
| g21249.t1 | Trihelix | | Vitvi10g00270.t01 | WRKY | |  | |  | |
| g21249.t2 | Trihelix | | Vitvi10g00618.t01 | WRKY | |  | |  | |
| g21447.t1 | Trihelix | | Vitvi10g00732.t01 | WRKY | |  | |  | |
| g21474.t1 | Trihelix | | Vitvi10g01078.t01 | WRKY | |  | |  | |
| g21806.t1 | Trihelix | | Vitvi11g00694.t01 | WRKY | |  | |  | |
| g2222.t1 | Trihelix | | Vitvi11g01188.t01 | WRKY | |  | |  | |
| g22383.t1 | Trihelix | | Vitvi12g00048.t01 | WRKY | |  | |  | |
| g23283.t1 | Trihelix | | Vitvi12g00148.t01 | WRKY | |  | |  | |
| g26498.t1 | Trihelix | | Vitvi12g00388.t01 | WRKY | |  | |  | |
| g29198.t1 | Trihelix | | Vitvi12g00664.t01 | WRKY | |  | |  | |
| g29564.t1 | Trihelix | | Vitvi12g01676.t01 | WRKY | |  | |  | |
| g29895.t1 | Trihelix | | Vitvi13g00189.t01 | WRKY | |  | |  | |
| g30851.t1 | Trihelix | | Vitvi13g01916.t01 | WRKY | |  | |  | |
| g31708.t1 | Trihelix | | Vitvi14g00540.t01 | WRKY | |  | |  | |
| g3173.t1 | Trihelix | | Vitvi14g01523.t01 | WRKY | |  | |  | |
| g33437.t1 | Trihelix | | Vitvi14g01907.t01 | WRKY | |  | |  | |
| g3425.t1 | Trihelix | | Vitvi14g02007.t01 | WRKY | |  | |  | |
| g34468.t1 | Trihelix | | Vitvi15g00539.t01 | WRKY | |  | |  | |
| g34468.t2 | Trihelix | | Vitvi15g01003.t01 | WRKY | |  | |  | |
| g34506.t1 | Trihelix | | Vitvi15g01087.t01 | WRKY | |  | |  | |
| g3529.t1 | Trihelix | | Vitvi15g01090.t01 | WRKY | |  | |  | |
| g353.t1 | Trihelix | | Vitvi16g01132.t01 | WRKY | |  | |  | |
| g36476.t1 | Trihelix | | Vitvi16g01133.t01 | WRKY | |  | |  | |
| g29208.t1 | VOZ | | Vitvi16g01213.t01 | WRKY | |  | |  | |
| g3237.t1 | VOZ | | Vitvi17g00102.t01 | WRKY | |  | |  | |
| g34054.t1 | Whirly | | Vitvi17g00556.t01 | WRKY | |  | |  | |
| g3640.t1 | Whirly | | Vitvi18g00742.t01 | WRKY | |  | |  | |
| g18078.t1 | WOX | | Vitvi19g00530.t01 | WRKY | |  | |  | |
| g25528.t1 | WOX | | Vitvi19g00617.t01 | WRKY | |  | |  | |
| g25254.t1 | WOX | | Vitvi19g00927.t01 | WRKY | |  | |  | |
| g24508.t1 | WOX | | Vitvi01g00013.t01 | YABBY | |  | |  | |
| g16421.t1 | WOX | | Vitvi01g00703.t01 | YABBY | |  | |  | |
| g29730.t1 | WOX | | Vitvi02g00510.t01 | YABBY | |  | |  | |
| g34377.t1 | WOX | | Vitvi06g00972.t01 | YABBY | |  | |  | |
| g18508.t1 | WOX | | Vitvi08g00274.t01 | YABBY | |  | |  | |
| g18509.t1 | WOX | | Vitvi11g00492.t01 | YABBY | |  | |  | |
| g19639.t1 | WOX | | Vitvi15g00708.t01 | YABBY | |  | |  | |
| g17085.t1 | WOX | | Vitvi01g00545.t01 | ZF-HD | |  | |  | |
| g10779.t1 | WRKY | | Vitvi01g01012.t01 | ZF-HD | |  | |  | |
| g1089.t1 | WRKY | | Vitvi01g01013.t01 | ZF-HD | |  | |  | |
| g11899.t1 | WRKY | | Vitvi04g01304.t01 | ZF-HD | |  | |  | |
| g12148.t1 | WRKY | | Vitvi07g01465.t01 | ZF-HD | |  | |  | |
| g12956.t1 | WRKY | | Vitvi07g02032.t01 | ZF-HD | |  | |  | |
| g13465.t1 | WRKY | | Vitvi08g00100.t01 | ZF-HD | |  | |  | |
| g14157.t1 | WRKY | | Vitvi12g01999.t01 | ZF-HD | |  | |  | |
| g14244.t1 | WRKY | | Vitvi12g02000.t01 | ZF-HD | |  | |  | |
| g14996.t1 | WRKY | | Vitvi12g02135.t01 | ZF-HD | |  | |  | |
| g15094.t1 | WRKY | | Vitvi14g01955.t01 | ZF-HD | |  | |  | |
| g15127.t1 | WRKY | | Vitvi14g01956.t01 | ZF-HD | |  | |  | |
| g15574.t1 | WRKY | | Vitvi14g01959.t01 | ZF-HD | |  | |  | |
| g15654.t1 | WRKY | | Vitvi17g00600.t01 | ZF-HD | |  | |  | |
| g15923.t1 | WRKY | | Vitvi17g01082.t01 | ZF-HD | |  | |  | |
| g16152.t1 | WRKY | | Vitvi18g00493.t01 | ZF-HD | |  | |  | |
| g16521.t1 | WRKY | | Vitvi18g00972.t01 | ZF-HD | |  | |  | |
| g18067.t1 | WRKY | | Vitvi18g01872.t01 | ZF-HD | |  | |  | |
| g18067.t2 | WRKY | |  |  | |  | |  | |
| g18340.t1 | WRKY | |  |  | |  | |  | |
| g1854.t1 | WRKY | |  |  | |  | |  | |
| g1855.t1 | WRKY | |  |  | |  | |  | |
| g19556.t1 | WRKY | |  |  | |  | |  | |
| g1999.t1 | WRKY | |  |  | |  | |  | |
| g20342.t1 | WRKY | |  |  | |  | |  | |
| g20354.t1 | WRKY | |  |  | |  | |  | |
| g20540.t1 | WRKY | |  |  | |  | |  | |
| g20893.t1 | WRKY | |  |  | |  | |  | |
| g20896.t1 | WRKY | |  |  | |  | |  | |
| g21000.t1 | WRKY | |  |  | |  | |  | |
| g21412.t1 | WRKY | |  |  | |  | |  | |
| g22488.t1 | WRKY | |  |  | |  | |  | |
| g22546.t1 | WRKY | |  |  | |  | |  | |
| g22793.t1 | WRKY | |  |  | |  | |  | |
| g22794.t1 | WRKY | |  |  | |  | |  | |
| g23440.t1 | WRKY | |  |  | |  | |  | |
| g24778.t1 | WRKY | |  |  | |  | |  | |
| g2482.t1 | WRKY | |  |  | |  | |  | |
| g24865.t1 | WRKY | |  |  | |  | |  | |
| g25179.t1 | WRKY | |  |  | |  | |  | |
| g25179.t2 | WRKY | |  |  | |  | |  | |
| g25382.t1 | WRKY | |  |  | |  | |  | |
| g25382.t2 | WRKY | |  |  | |  | |  | |
| g25814.t1 | WRKY | |  |  | |  | |  | |
| g27013.t1 | WRKY | |  |  | |  | |  | |
| g27122.t1 | WRKY | |  |  | |  | |  | |
| g27666.t1 | WRKY | |  |  | |  | |  | |
| g2994.t1 | WRKY | |  |  | |  | |  | |
| g29975.t1 | WRKY | |  |  | |  | |  | |
| g30173.t1 | WRKY | |  |  | |  | |  | |
| g30178.t1 | WRKY | |  |  | |  | |  | |
| g30178.t2 | WRKY | |  |  | |  | |  | |
| g30179.t1 | WRKY | |  |  | |  | |  | |
| g30293.t1 | WRKY | |  |  | |  | |  | |
| g30427.t1 | WRKY | |  |  | |  | |  | |
| g3138.t1 | WRKY | |  |  | |  | |  | |
| g31443.t1 | WRKY | |  |  | |  | |  | |
| g32642.t1 | WRKY | |  |  | |  | |  | |
| g33662.t1 | WRKY | |  |  | |  | |  | |
| g3380.t1 | WRKY | |  |  | |  | |  | |
| g34971.t1 | WRKY | |  |  | |  | |  | |
| g36183.t1 | WRKY | |  |  | |  | |  | |
| g3639.t1 | WRKY | |  |  | |  | |  | |
| g36947.t1 | WRKY | |  |  | |  | |  | |
| g36963.t1 | WRKY | |  |  | |  | |  | |
| g585.t1 | WRKY | |  |  | |  | |  | |
| g6128.t1 | WRKY | |  |  | |  | |  | |
| g6533.t1 | WRKY | |  |  | |  | |  | |
| g15940.t1 | YABBY | |  |  | |  | |  | |
| g21063.t1 | YABBY | |  |  | |  | |  | |
| g22017.t1 | YABBY | |  |  | |  | |  | |
| g2383.t1 | YABBY | |  |  | |  | |  | |
| g25767.t1 | YABBY | |  |  | |  | |  | |
| g30714.t1 | YABBY | |  |  | |  | |  | |
| g30715.t1 | YABBY | |  |  | |  | |  | |
| g32018.t1 | YABBY | |  |  | |  | |  | |
| g32018.t2 | YABBY | |  |  | |  | |  | |
| g32018.t3 | YABBY | |  |  | |  | |  | |
| g16949.t1 | ZF-HD | |  |  | |  | |  | |
| g22046.t1 | ZF-HD | |  |  | |  | |  | |
| g22077.t1 | ZF-HD | |  |  | |  | |  | |
| g22120.t1 | ZF-HD | |  |  | |  | |  | |
| g22631.t1 | ZF-HD | |  |  | |  | |  | |
| g22632.t1 | ZF-HD | |  |  | |  | |  | |
| g23092.t1 | ZF-HD | |  |  | |  | |  | |
| g25678.t1 | ZF-HD | |  |  | |  | |  | |
| g26831.t1 | ZF-HD | |  |  | |  | |  | |
| g27070.t1 | ZF-HD | |  |  | |  | |  | |
| g27071.t1 | ZF-HD | |  |  | |  | |  | |
| g27072.t1 | ZF-HD | |  |  | |  | |  | |
| g29723.t1 | ZF-HD | |  |  | |  | |  | |
| g34788.t1 | ZF-HD | |  |  | |  | |  | |
| g34789.t1 | ZF-HD | |  |  | |  | |  | |
| g34813.t1 | ZF-HD | |  |  | |  | |  | |
| g570.t1 | ZF-HD | |  |  | |  | |  | |
| g819.t2 | ZF-HD | |  |  | |  | |  | |

| **Supplementary Table 6b. Results of PlantTFDB for *V. riparia* 'Manitoba 37' and V. vinifera PN40024, 12X.2, V3 and V1 with protein sequence information.** | | | | | |
| --- | --- | --- | --- | --- | --- |
| **V. riparia protein sequence** | **TF Family** | **V. vinifera V3 protein sequence** | **TF Family** | **V. vinifera V1 protein sequence** | **TF Family** |
| g12372.t1 | AP2 | Vitvi01g00934.t01 | AP2 | chr1.gff3_PROT_VIT_01s0026g01690.t01 | AP2 |
| g14510.t1 | AP2 | Vitvi04g01207.t01 | AP2 | chr11.gff3_PROT_VIT_11s0037g00870.t01 | AP2 |
| g16916.t1 | AP2 | Vitvi06g00187.t01 | AP2 | chr11.gff3_PROT_VIT_11s0052g00840.t01 | AP2 |
| g17077.t1 | AP2 | Vitvi06g00360.t01 | AP2 | chr13.gff3_PROT_VIT_13s0019g03550.t01 | AP2 |
| g17793.t1 | AP2 | Vitvi07g01419.t01 | AP2 | chr13.gff3_PROT_VIT_13s0047g00340.t01 | AP2 |
| g17932.t1 | AP2 | Vitvi07g01421.t01 | AP2 | chr14.gff3_PROT_VIT_14s0108g00050.t01 | AP2 |
| g19616.t1 | AP2 | Vitvi07g01546.t01 | AP2 | chr18.gff3_PROT_VIT_18s0001g08610.t01 | AP2 |
| g20530.t1 | AP2 | Vitvi07g01706.t01 | AP2 | chr6.gff3_PROT_VIT_06s0004g01800.t01 | AP2 |
| g20687.t1 | AP2 | Vitvi08g01146.t01 | AP2 | chr6.gff3_PROT_VIT_06s0004g03590.t01 | AP2 |
| g24789.t1 | AP2 | Vitvi09g00108.t01 | AP2 | chr7.gff3_PROT_VIT_07s0031g00220.t01 | AP2 |
| g27006.t1 | AP2 | Vitvi09g01269.t01 | AP2 | chr7_random.gff3_PROT_VIT_07s0151g00440.t01 | AP2 |
| g31195.t1 | AP2 | Vitvi11g00798.t01 | AP2 | chr8.gff3_PROT_VIT_08s0040g03180.t01 | AP2 |
| g3645.t1 | AP2 | Vitvi11g01231.t01 | AP2 | chr9.gff3_PROT_VIT_09s0002g01370.t01 | AP2 |
| g3645.t2 | AP2 | Vitvi13g00529.t01 | AP2 | chr9.gff3_PROT_VIT_09s0018g01650.t01 | AP2 |
| g31083.t1 | AP2 | Vitvi13g01862.t01 | AP2 | chrUn.gff3_PROT_VIT_00s0772g00020.t01 | AP2 |
| g33897.t1 | AP2 | Vitvi14g01902.t01 | AP2 | chrUn.gff3_PROT_VIT_00s1291g00010.t01 | AP2 |
| g23365.t1 | AP2 | Vitvi18g00618.t01 | AP2 | chr4.gff3_PROT_VIT_04s0023g00950.t01 | AP2 |
| g20474.t1 | AP2 | Vitvi08g01919.t01 | AP2 | chr8.gff3_PROT_VIT_08s0007g08580.t01 | AP2 |
| g19817.t1 | AP2 | Vitvi01g01759.t01 | ARF | chr1.gff3_PROT_VIT_01s0244g00150.t01 | ARF |
| g17077.t2 | AP2 | Vitvi02g00163.t01 | ARF | chr10.gff3_PROT_VIT_10s0003g00420.t01 | ARF |
| g1130.t1 | AP2 | Vitvi04g00824.t01 | ARF | chr10.gff3_PROT_VIT_10s0003g04100.t01 | ARF |
| g12025.t1 | ARF | Vitvi06g00272.t01 | ARF | chr11.gff3_PROT_VIT_11s0016g00640.t01 | ARF |
| g13413.t1 | ARF | Vitvi06g00311.t01 | ARF | chr11.gff3_PROT_VIT_11s0065g00310.t01 | ARF |
| g1515.t1 | ARF | Vitvi07g00238.t01 | ARF | chr12.gff3_PROT_VIT_12s0028g01170.t01 | ARF |
| g19385.t1 | ARF | Vitvi08g01033.t01 | ARF | chr12.gff3_PROT_VIT_12s0035g01800.t01 | ARF |
| g19385.t2 | ARF | Vitvi10g00510.t01 | ARF | chr13.gff3_PROT_VIT_13s0019g04380.t01 | ARF |
| g20160.t1 | ARF | Vitvi10g00854.t01 | ARF | chr15.gff3_PROT_VIT_15s0046g00290.t01 | ARF |
| g22234.t1 | ARF | Vitvi11g00043.t01 | ARF | chr17.gff3_PROT_VIT_17s0000g00320.t01 | ARF |
| g23277.t1 | ARF | Vitvi11g00920.t01 | ARF | chr18.gff3_PROT_VIT_18s0001g13930.t01 | ARF |
| g24564.t1 | ARF | Vitvi12g00102.t01 | ARF | chr18.gff3_PROT_VIT_18s0089g00910.t01 | ARF |
| g24564.t2 | ARF | Vitvi12g02122.t01 | ARF | chr2.gff3_PROT_VIT_02s0025g01740.t01 | ARF |
| g24564.t3 | ARF | Vitvi13g02058.t01 | ARF | chr4.gff3_PROT_VIT_04s0079g00200.t01 | ARF |
| g25099.t1 | ARF | Vitvi15g00946.t01 | ARF | chr6.gff3_PROT_VIT_06s0004g02750.t01 | ARF |
| g26759.t1 | ARF | Vitvi17g00036.t01 | ARF | chr6.gff3_PROT_VIT_06s0004g03130.t01 | ARF |
| g2777.t1 | ARF | Vitvi18g00337.t01 | ARF | chr7.gff3_PROT_VIT_07s0104g01230.t01 | ARF |
| g29213.t1 | ARF | Vitvi18g01086.t01 | ARF | chr8.gff3_PROT_VIT_08s0040g01810.t01 | ARF |
| g2947.t1 | ARF | Vitvi18g02390.t01 | ARF | chr1.gff3_PROT_VIT_01s0010g02230.t01 | ARR-B |
| g31805.t1 | ARF | Vitvi01g00341.t01 | ARR-B | chr1.gff3_PROT_VIT_01s0011g05830.t01 | ARR-B |
| g31849.t1 | ARF | Vitvi01g00344.t01 | ARR-B | chr11.gff3_PROT_VIT_11s0052g01160.t01 | ARR-B |
| g32848.t1 | ARF | Vitvi01g01506.t01 | ARR-B | chr11.gff3_PROT_VIT_11s0206g00060.t01 | ARR-B |
| g33442.t1 | ARF | Vitvi01g01959.t01 | ARR-B | chr16.gff3_PROT_VIT_16s0100g00420.t01 | ARR-B |
| g34055.t1 | ARF | Vitvi01g01995.t01 | ARR-B | chr17.gff3_PROT_VIT_17s0000g10100.t01 | ARR-B |
| g939.t1 | ARF | Vitvi02g00636.t01 | ARR-B | chr17.gff3_PROT_VIT_17s0000g10110.t01 | ARR-B |
| g11888.t1 | ARR-B | Vitvi04g00525.t01 | ARR-B | chr2.gff3_PROT_VIT_02s0012g00570.t01 | ARR-B |
| g21889.t1 | ARR-B | Vitvi05g00075.t01 | ARR-B | chr4.gff3_PROT_VIT_04s0008g05900.t01 | ARR-B |
| g24435.t1 | ARR-B | Vitvi07g00374.t01 | ARR-B | chr5.gff3_PROT_VIT_05s0077g01480.t01 | ARR-B |
| g27463.t1 | ARR-B | Vitvi11g00651.t01 | ARR-B | chr7.gff3_PROT_VIT_07s0005g01010.t01 | ARR-B |
| g27463.t2 | ARR-B | Vitvi11g01262.t01 | ARR-B | chr10.gff3_PROT_VIT_10s0071g00270.t01 | B3 |
| g27706.t1 | ARR-B | Vitvi15g00751.t01 | ARR-B | chr12.gff3_PROT_VIT_12s0035g02120.t01 | B3 |
| g28658.t1 | ARR-B | Vitvi16g00946.t01 | ARR-B | chr14.gff3_PROT_VIT_14s0068g01290.t01 | B3 |
| g28659.t1 | ARR-B | Vitvi16g00948.t01 | ARR-B | chr15.gff3_PROT_VIT_15s0024g00930.t01 | B3 |
| g30131.t1 | ARR-B | Vitvi17g01017.t01 | ARR-B | chr15.gff3_PROT_VIT_15s0048g02370.t01 | B3 |
| g30136.t1 | ARR-B | Vitvi17g01018.t01 | ARR-B | chr17.gff3_PROT_VIT_17s0000g10080.t01 | B3 |
| g30137.t1 | ARR-B | Vitvi01g01743.t01 | B3 | chr18.gff3_PROT_VIT_18s0001g10710.t01 | B3 |
| g34489.t1 | ARR-B | Vitvi02g00275.t01 | B3 | chr18.gff3_PROT_VIT_18s0001g11360.t01 | B3 |
| g34709.t1 | ARR-B | Vitvi03g00415.t01 | B3 | chr19.gff3_PROT_VIT_19s0014g03560.t01 | B3 |
| g35794.t1 | ARR-B | Vitvi03g00416.t01 | B3 | chr2.gff3_PROT_VIT_02s0025g03000.t01 | B3 |
| g36453.t1 | ARR-B | Vitvi03g00417.t01 | B3 | chr3.gff3_PROT_VIT_03s0063g00620.t01 | B3 |
| g37024.t1 | ARR-B | Vitvi03g00419.t01 | B3 | chr3.gff3_PROT_VIT_03s0063g00630.t01 | B3 |
| g10322.t1 | B3 | Vitvi03g00421.t01 | B3 | chr3.gff3_PROT_VIT_03s0063g00640.t01 | B3 |
| g13116.t1 | B3 | Vitvi03g00470.t01 | B3 | chr3.gff3_PROT_VIT_03s0063g00910.t01 | B3 |
| g14169.t1 | B3 | Vitvi03g00487.t01 | B3 | chr3.gff3_PROT_VIT_03s0063g01420.t01 | B3 |
| g14169.t2 | B3 | Vitvi03g00488.t01 | B3 | chr3.gff3_PROT_VIT_03s0063g01440.t01 | B3 |
| g14311.t1 | B3 | Vitvi03g00489.t01 | B3 | chr3.gff3_PROT_VIT_03s0063g01450.t01 | B3 |
| g14779.t1 | B3 | Vitvi03g00907.t01 | B3 | chr3.gff3_PROT_VIT_03s0063g01460.t01 | B3 |
| g14780.t1 | B3 | Vitvi03g01537.t01 | B3 | chr3.gff3_PROT_VIT_03s0167g00010.t01 | B3 |
| g14781.t1 | B3 | Vitvi03g01552.t01 | B3 | chr4.gff3_PROT_VIT_04s0079g00160.t01 | B3 |
| g14784.t1 | B3 | Vitvi06g00802.t01 | B3 | chr6.gff3_PROT_VIT_06s0004g08100.t01 | B3 |
| g14784.t2 | B3 | Vitvi06g00822.t01 | B3 | chr6.gff3_PROT_VIT_06s0004g08270.t01 | B3 |
| g15228.t1 | B3 | Vitvi07g00781.t01 | B3 | chr7.gff3_PROT_VIT_07s0005g05400.t01 | B3 |
| g15228.t2 | B3 | Vitvi07g01289.t01 | B3 | chr7.gff3_PROT_VIT_07s0031g00780.t01 | B3 |
| g15228.t3 | B3 | Vitvi07g01290.t01 | B3 | chr7.gff3_PROT_VIT_07s0255g00070.t01 | B3 |
| g15832.t1 | B3 | Vitvi07g01291.t01 | B3 | chr7.gff3_PROT_VIT_07s0255g00080.t01 | B3 |
| g17307.t1 | B3 | Vitvi07g01292.t01 | B3 | chr8.gff3_PROT_VIT_08s0007g02810.t01 | B3 |
| g18136.t1 | B3 | Vitvi07g01293.t01 | B3 | chr8.gff3_PROT_VIT_08s0058g01300.t01 | B3 |
| g18317.t1 | B3 | Vitvi07g01762.t01 | B3 | chrUn.gff3_PROT_VIT_00s0181g00260.t01 | B3 |
| g20455.t1 | B3 | Vitvi07g02422.t01 | B3 | chrUn.gff3_PROT_VIT_00s1203g00010.t01 | B3 |
| g21233.t1 | B3 | Vitvi08g00859.t01 | B3 | chr1.gff3_PROT_VIT_01s0011g06380.t01 | BBR-BPC |
| g21792.t1 | B3 | Vitvi08g01412.t01 | B3 | chr17.gff3_PROT_VIT_17s0000g01860.t01 | BBR-BPC |
| g24986.t1 | B3 | Vitvi10g00384.t01 | B3 | chr18.gff3_PROT_VIT_18s0001g13710.t01 | BBR-BPC |
| g26663.t1 | B3 | Vitvi10g02053.t01 | B3 | chr18.gff3_PROT_VIT_18s0001g13730.t01 | BBR-BPC |
| g27198.t1 | B3 | Vitvi11g00842.t01 | B3 | chr3.gff3_PROT_VIT_03s0038g00840.t01 | BBR-BPC |
| g28656.t1 | B3 | Vitvi11g00854.t01 | B3 | chr10.gff3_PROT_VIT_10s0003g01790.t01 | BES1 |
| g30016.t1 | B3 | Vitvi11g00864.t01 | B3 | chr10.gff3_PROT_VIT_10s0003g04710.t01 | BES1 |
| g30439.t1 | B3 | Vitvi11g01537.t01 | B3 | chr15.gff3_PROT_VIT_15s0046g02640.t01 | BES1 |
| g32712.t1 | B3 | Vitvi11g01539.t01 | B3 | chr18.gff3_PROT_VIT_18s0001g12020.t01 | BES1 |
| g32797.t1 | B3 | Vitvi12g02160.t01 | B3 | chr19.gff3_PROT_VIT_19s0014g00870.t01 | BES1 |
| g33365.t1 | B3 | Vitvi14g01480.t01 | B3 | chr19.gff3_PROT_VIT_19s0014g00880.t01 | BES1 |
| g33366.t1 | B3 | Vitvi15g00863.t01 | B3 | chr2.gff3_PROT_VIT_02s0087g00430.t01 | BES1 |
| g33368.t1 | B3 | Vitvi15g00981.t01 | B3 | chr4.gff3_PROT_VIT_04s0023g01250.t01 | BES1 |
| g33372.t1 | B3 | Vitvi15g01244.t01 | B3 | chr1.gff3_PROT_VIT_01s0010g00540.t01 | bHLH |
| g33376.t1 | B3 | Vitvi15g01589.t01 | B3 | chr1.gff3_PROT_VIT_01s0010g00740.t01 | bHLH |
| g33377.t1 | B3 | Vitvi15g01592.t01 | B3 | chr1.gff3_PROT_VIT_01s0010g02070.t01 | bHLH |
| g33378.t1 | B3 | Vitvi15g01593.t01 | B3 | chr1.gff3_PROT_VIT_01s0011g02940.t01 | bHLH |
| g34899.t1 | B3 | Vitvi15g01767.t01 | B3 | chr1.gff3_PROT_VIT_01s0011g03720.t01 | bHLH |
| g35337.t1 | B3 | Vitvi17g01015.t01 | B3 | chr1.gff3_PROT_VIT_01s0026g01140.t01 | bHLH |
| g35338.t1 | B3 | Vitvi18g00807.t01 | B3 | chr1.gff3_PROT_VIT_01s0026g02030.t01 | bHLH |
| g35339.t1 | B3 | Vitvi18g00865.t01 | B3 | chr1.gff3_PROT_VIT_01s0127g00860.t01 | bHLH |
| g35339.t2 | B3 | Vitvi18g00866.t01 | B3 | chr1.gff3_PROT_VIT_01s0127g00910.t01 | bHLH |
| g35364.t1 | B3 | Vitvi19g00295.t01 | B3 | chr1.gff3_PROT_VIT_01s0244g00010.t01 | bHLH |
| g35701.t1 | B3 | Vitvi01g00547.t01 | BBR-BPC | chr1.gff3_PROT_VIT_01s0244g00130.t01 | bHLH |
| g35703.t1 | B3 | Vitvi03g00058.t01 | BBR-BPC | chr10.gff3_PROT_VIT_10s0003g01170.t01 | bHLH |
| g35704.t1 | B3 | Vitvi17g00156.t01 | BBR-BPC | chr10.gff3_PROT_VIT_10s0003g02940.t01 | bHLH |
| g35705.t1 | B3 | Vitvi18g01066.t01 | BBR-BPC | chr10.gff3_PROT_VIT_10s0092g00030.t01 | bHLH |
| g35706.t1 | B3 | Vitvi18g01067.t01 | BBR-BPC | chr11.gff3_PROT_VIT_11s0016g00380.t01 | bHLH |
| g35708.t1 | B3 | Vitvi02g01232.t01 | BES1 | chr11.gff3_PROT_VIT_11s0016g02070.t01 | bHLH |
| g35708.t2 | B3 | Vitvi04g01234.t01 | BES1 | chr11.gff3_PROT_VIT_11s0016g03560.t01 | bHLH |
| g35708.t3 | B3 | Vitvi08g00772.t01 | BES1 | chr11.gff3_PROT_VIT_11s0037g00040.t01 | bHLH |
| g35708.t4 | B3 | Vitvi10g00636.t01 | BES1 | chr11.gff3_PROT_VIT_11s0052g00100.t01 | bHLH |
| g36312.t1 | B3 | Vitvi10g01901.t01 | BES1 | chr12.gff3_PROT_VIT_12s0028g01110.t01 | bHLH |
| g37920.t1 | B3 | Vitvi15g01128.t01 | BES1 | chr12.gff3_PROT_VIT_12s0028g02350.t01 | bHLH |
| g37921.t1 | B3 | Vitvi18g00924.t01 | BES1 | chr12.gff3_PROT_VIT_12s0028g03550.t01 | bHLH |
| g4003.t1 | B3 | Vitvi19g00061.t01 | BES1 | chr12.gff3_PROT_VIT_12s0034g02150.t01 | bHLH |
| g4590.t1 | B3 | Vitvi19g00063.t01 | BES1 | chr12.gff3_PROT_VIT_12s0059g02650.t01 | bHLH |
| g5537.t1 | B3 | Vitvi00g00582.t01 | bHLH | chr13.gff3_PROT_VIT_13s0019g00540.t01 | bHLH |
| g6452.t1 | B3 | Vitvi00g00944.t01 | bHLH | chr13.gff3_PROT_VIT_13s0047g00450.t01 | bHLH |
| g708.t1 | B3 | Vitvi00g01001.t01 | bHLH | chr13.gff3_PROT_VIT_13s0064g01290.t01 | bHLH |
| g708.t2 | B3 | Vitvi01g00232.t01 | bHLH | chr13.gff3_PROT_VIT_13s0067g01350.t01 | bHLH |
| g709.t1 | B3 | Vitvi01g00727.t01 | bHLH | chr13.gff3_PROT_VIT_13s0073g00400.t01 | bHLH |
| g8357.t1 | B3 | Vitvi01g00746.t01 | bHLH | chr14.gff3_PROT_VIT_14s0006g01470.t01 | bHLH |
| g23094.t1 | BBR-BPC | Vitvi01g00752.t01 | bHLH | chr14.gff3_PROT_VIT_14s0006g02850.t01 | bHLH |
| g24950.t1 | BBR-BPC | Vitvi01g00762.t01 | bHLH | chr14.gff3_PROT_VIT_14s0030g02230.t01 | bHLH |
| g26801.t1 | BBR-BPC | Vitvi01g00876.t01 | bHLH | chr14.gff3_PROT_VIT_14s0060g00260.t01 | bHLH |
| g921.t1 | BBR-BPC | Vitvi01g00964.t01 | bHLH | chr14.gff3_PROT_VIT_14s0068g01200.t01 | bHLH |
| g923.t1 | BBR-BPC | Vitvi01g01287.t01 | bHLH | chr14.gff3_PROT_VIT_14s0068g01580.t01 | bHLH |
| g14262.t1 | BES1 | Vitvi01g01316.t01 | bHLH | chr14.gff3_PROT_VIT_14s0081g00720.t01 | bHLH |
| g16543.t1 | BES1 | Vitvi01g01482.t01 | bHLH | chr14.gff3_PROT_VIT_14s0083g00930.t01 | bHLH |
| g19598.t1 | BES1 | Vitvi01g01745.t01 | bHLH | chr14.gff3_PROT_VIT_14s0108g00420.t01 | bHLH |
| g20856.t1 | BES1 | Vitvi01g01757.t01 | bHLH | chr14.gff3_PROT_VIT_14s0128g00110.t01 | bHLH |
| g22864.t1 | BES1 | Vitvi01g01946.t01 | bHLH | chr15.gff3_PROT_VIT_15s0021g02690.t01 | bHLH |
| g29598.t1 | BES1 | Vitvi01g02070.t01 | bHLH | chr15.gff3_PROT_VIT_15s0046g00320.t01 | bHLH |
| g31231.t1 | BES1 | Vitvi01g02072.t01 | bHLH | chr15.gff3_PROT_VIT_15s0046g02560.t01 | bHLH |
| g37571.t1 | BES1 | Vitvi01g02073.t01 | bHLH | chr15.gff3_PROT_VIT_15s0048g02510.t01 | bHLH |
| g5825.t1 | BES1 | Vitvi01g02074.t01 | bHLH | chr15.gff3_PROT_VIT_15s0048g02820.t01 | bHLH |
| g766.t1 | BES1 | Vitvi01g02081.t01 | bHLH | chr15.gff3_PROT_VIT_15s0107g00380.t01 | bHLH |
| g10089.t1 | bHLH | Vitvi02g00231.t01 | bHLH | chr16.gff3_PROT_VIT_16s0022g02240.t01 | bHLH |
| g10718.t1 | bHLH | Vitvi02g00317.t01 | bHLH | chr16.gff3_PROT_VIT_16s0022g02250.t01 | bHLH |
| g11483.t1 | bHLH | Vitvi02g00439.t01 | bHLH | chr16.gff3_PROT_VIT_16s0022g02270.t01 | bHLH |
| g11766.t1 | bHLH | Vitvi02g00698.t01 | bHLH | chr16.gff3_PROT_VIT_16s0050g02500.t01 | bHLH |
| g11769.t1 | bHLH | Vitvi02g00709.t01 | bHLH | chr17.gff3_PROT_VIT_17s0000g00330.t01 | bHLH |
| g11828.t1 | bHLH | Vitvi03g00098.t01 | bHLH | chr17.gff3_PROT_VIT_17s0000g00430.t01 | bHLH |
| g11828.t2 | bHLH | Vitvi03g00157.t01 | bHLH | chr17.gff3_PROT_VIT_17s0000g03550.t01 | bHLH |
| g11918.t1 | bHLH | Vitvi03g00315.t01 | bHLH | chr17.gff3_PROT_VIT_17s0000g03580.t01 | bHLH |
| g11920.t1 | bHLH | Vitvi03g00520.t01 | bHLH | chr17.gff3_PROT_VIT_17s0000g04790.t01 | bHLH |
| g11922.t1 | bHLH | Vitvi03g00635.t01 | bHLH | chr17.gff3_PROT_VIT_17s0000g05370.t01 | bHLH |
| g11923.t1 | bHLH | Vitvi03g00791.t01 | bHLH | chr17.gff3_PROT_VIT_17s0000g05900.t01 | bHLH |
| g12168.t1 | bHLH | Vitvi04g01312.t01 | bHLH | chr17.gff3_PROT_VIT_17s0000g05950.t01 | bHLH |
| g12168.t2 | bHLH | Vitvi04g01361.t01 | bHLH | chr17.gff3_PROT_VIT_17s0000g06930.t01 | bHLH |
| g13360.t1 | bHLH | Vitvi04g01463.t01 | bHLH | chr17.gff3_PROT_VIT_17s0000g08150.t01 | bHLH |
| g13418.t1 | bHLH | Vitvi04g01662.t01 | bHLH | chr18.gff3_PROT_VIT_18s0001g06650.t01 | bHLH |
| g13866.t1 | bHLH | Vitvi05g00143.t01 | bHLH | chr18.gff3_PROT_VIT_18s0001g07410.t01 | bHLH |
| g14518.t1 | bHLH | Vitvi05g00427.t01 | bHLH | chr18.gff3_PROT_VIT_18s0001g08040.t01 | bHLH |
| g14997.t1 | bHLH | Vitvi05g00624.t01 | bHLH | chr18.gff3_PROT_VIT_18s0001g08600.t01 | bHLH |
| g15546.t1 | bHLH | Vitvi05g00640.t01 | bHLH | chr18.gff3_PROT_VIT_18s0001g09210.t01 | bHLH |
| g15593.t1 | bHLH | Vitvi05g00711.t01 | bHLH | chr18.gff3_PROT_VIT_18s0001g10270.t01 | bHLH |
| g15719.t1 | bHLH | Vitvi05g01063.t01 | bHLH | chr18.gff3_PROT_VIT_18s0001g10300.t01 | bHLH |
| g15734.t1 | bHLH | Vitvi05g01103.t01 | bHLH | chr18.gff3_PROT_VIT_18s0001g10400.t01 | bHLH |
| g1596.t1 | bHLH | Vitvi05g01393.t01 | bHLH | chr18.gff3_PROT_VIT_18s0001g12970.t01 | bHLH |
| g15988.t1 | bHLH | Vitvi05g01597.t01 | bHLH | chr18.gff3_PROT_VIT_18s0041g00690.t01 | bHLH |
| g16065.t1 | bHLH | Vitvi05g01857.t01 | bHLH | chr19.gff3_PROT_VIT_19s0014g04670.t01 | bHLH |
| g1607.t1 | bHLH | Vitvi06g00181.t01 | bHLH | chr19.gff3_PROT_VIT_19s0014g05100.t01 | bHLH |
| g16173.t1 | bHLH | Vitvi06g01345.t01 | bHLH | chr2.gff3_PROT_VIT_02s0012g01320.t01 | bHLH |
| g16477.t1 | bHLH | Vitvi07g00002.t01 | bHLH | chr2.gff3_PROT_VIT_02s0012g01450.t01 | bHLH |
| g16637.t1 | bHLH | Vitvi07g00046.t01 | bHLH | chr2.gff3_PROT_VIT_02s0025g02610.t01 | bHLH |
| g16794.t1 | bHLH | Vitvi07g00139.t01 | bHLH | chr2.gff3_PROT_VIT_02s0025g03450.t01 | bHLH |
| g16795.t1 | bHLH | Vitvi07g00151.t01 | bHLH | chr3.gff3_PROT_VIT_03s0038g01790.t01 | bHLH |
| g16802.t1 | bHLH | Vitvi07g00762.t01 | bHLH | chr3.gff3_PROT_VIT_03s0038g02540.t01 | bHLH |
| g16802.t2 | bHLH | Vitvi07g01475.t01 | bHLH | chr3.gff3_PROT_VIT_03s0038g04760.t01 | bHLH |
| g16896.t1 | bHLH | Vitvi07g01529.t01 | bHLH | chr3.gff3_PROT_VIT_03s0063g00170.t01 | bHLH |
| g16933.t1 | bHLH | Vitvi07g01530.t01 | bHLH | chr3.gff3_PROT_VIT_03s0088g01240.t01 | bHLH |
| g16961.t1 | bHLH | Vitvi07g01531.t01 | bHLH | chr3.gff3_PROT_VIT_03s0091g00730.t01 | bHLH |
| g17162.t1 | bHLH | Vitvi07g01532.t01 | bHLH | chr4.gff3_PROT_VIT_04s0023g01930.t01 | bHLH |
| g17162.t2 | bHLH | Vitvi07g01545.t01 | bHLH | chr4.gff3_PROT_VIT_04s0023g02410.t01 | bHLH |
| g17163.t1 | bHLH | Vitvi07g01729.t01 | bHLH | chr4.gff3_PROT_VIT_04s0023g03430.t01 | bHLH |
| g17164.t1 | bHLH | Vitvi07g01740.t01 | bHLH | chr4.gff3_PROT_VIT_04s0044g01040.t01 | bHLH |
| g1752.t1 | bHLH | Vitvi07g02251.t01 | bHLH | chr5.gff3_PROT_VIT_05s0020g02700.t01 | bHLH |
| g17557.t1 | bHLH | Vitvi07g02613.t01 | bHLH | chr5.gff3_PROT_VIT_05s0020g04620.t01 | bHLH |
| g17783.t1 | bHLH | Vitvi07g03072.t01 | bHLH | chr5.gff3_PROT_VIT_05s0020g04780.t01 | bHLH |
| g17787.t1 | bHLH | Vitvi07g03073.t01 | bHLH | chr5.gff3_PROT_VIT_05s0029g00050.t01 | bHLH |
| g17877.t1 | bHLH | Vitvi08g00719.t01 | bHLH | chr5.gff3_PROT_VIT_05s0029g00390.t01 | bHLH |
| g17930.t1 | bHLH | Vitvi08g00979.t01 | bHLH | chr5.gff3_PROT_VIT_05s0049g00460.t01 | bHLH |
| g17930.t2 | bHLH | Vitvi08g01649.t01 | bHLH | chr5.gff3_PROT_VIT_05s0077g00750.t01 | bHLH |
| g17988.t1 | bHLH | Vitvi08g01852.t01 | bHLH | chr5.gff3_PROT_VIT_05s0094g00480.t01 | bHLH |
| g18087.t1 | bHLH | Vitvi08g01856.t01 | bHLH | chr5.gff3_PROT_VIT_05s0124g00240.t01 | bHLH |
| g18087.t2 | bHLH | Vitvi09g00227.t01 | bHLH | chr6.gff3_PROT_VIT_06s0004g01740.t01 | bHLH |
| g18088.t1 | bHLH | Vitvi09g00339.t01 | bHLH | chr6.gff3_PROT_VIT_06s0061g00720.t01 | bHLH |
| g18099.t1 | bHLH | Vitvi10g00320.t01 | bHLH | chr7.gff3_PROT_VIT_07s0005g02510.t01 | bHLH |
| g18165.t1 | bHLH | Vitvi10g00323.t01 | bHLH | chr7.gff3_PROT_VIT_07s0005g05100.t01 | bHLH |
| g18285.t1 | bHLH | Vitvi10g00572.t01 | bHLH | chr7.gff3_PROT_VIT_07s0031g00450.t01 | bHLH |
| g18436.t1 | bHLH | Vitvi10g00744.t01 | bHLH | chr7.gff3_PROT_VIT_07s0031g00550.t01 | bHLH |
| g18800.t1 | bHLH | Vitvi10g01945.t01 | bHLH | chr7.gff3_PROT_VIT_07s0104g00090.t01 | bHLH |
| g19024.t1 | bHLH | Vitvi10g02097.t01 | bHLH | chr7.gff3_PROT_VIT_07s0104g00250.t01 | bHLH |
| g1933.t1 | bHLH | Vitvi10g02164.t01 | bHLH | chr7.gff3_PROT_VIT_07s0141g00220.t01 | bHLH |
| g19602.t1 | bHLH | Vitvi10g02218.t01 | bHLH | chr7.gff3_PROT_VIT_07s0141g01060.t01 | bHLH |
| g19830.t1 | bHLH | Vitvi10g02253.t01 | bHLH | chr7.gff3_PROT_VIT_07s0191g00240.t01 | bHLH |
| g19858.t1 | bHLH | Vitvi11g00021.t01 | bHLH | chr7.gff3_PROT_VIT_07s0205g00160.t01 | bHLH |
| g20488.t1 | bHLH | Vitvi11g00165.t01 | bHLH | chr7.gff3_PROT_VIT_07s0205g00180.t01 | bHLH |
| g20499.t1 | bHLH | Vitvi11g00303.t01 | bHLH | chr7.gff3_PROT_VIT_07s0205g00190.t01 | bHLH |
| g20688.t1 | bHLH | Vitvi11g00680.t01 | bHLH | chr7_random.gff3_PROT_VIT_07s0151g00450.t01 | bHLH |
| g207.t1 | bHLH | Vitvi11g01153.t01 | bHLH | chr8.gff3_PROT_VIT_08s0007g05300.t01 | bHLH |
| g20722.t1 | bHLH | Vitvi12g00096.t01 | bHLH | chr8.gff3_PROT_VIT_08s0007g07810.t01 | bHLH |
| g20723.t1 | bHLH | Vitvi12g00200.t01 | bHLH | chr8.gff3_PROT_VIT_08s0007g07870.t01 | bHLH |
| g20724.t1 | bHLH | Vitvi12g00556.t01 | bHLH | chr8.gff3_PROT_VIT_08s0040g01240.t01 | bHLH |
| g20725.t1 | bHLH | Vitvi12g00567.t01 | bHLH | chr8.gff3_PROT_VIT_08s0058g00110.t01 | bHLH |
| g20726.t1 | bHLH | Vitvi12g00860.t01 | bHLH | chr9.gff3_PROT_VIT_09s0002g02700.t01 | bHLH |
| g20727.t1 | bHLH | Vitvi12g01896.t01 | bHLH | chr9.gff3_PROT_VIT_09s0002g04120.t01 | bHLH |
| g20741.t1 | bHLH | Vitvi12g02307.t01 | bHLH | chrUn.gff3_PROT_VIT_00s0274g00070.t01 | bHLH |
| g20860.t1 | bHLH | Vitvi13g00075.t01 | bHLH | chrUn.gff3_PROT_VIT_00s0384g00020.t01 | bHLH |
| g21046.t1 | bHLH | Vitvi13g01037.t01 | bHLH | chrUn.gff3_PROT_VIT_00s0479g00020.t01 | bHLH |
| g21190.t1 | bHLH | Vitvi13g01164.t01 | bHLH | chrUn.gff3_PROT_VIT_00s0532g00050.t01 | bHLH |
| g21272.t1 | bHLH | Vitvi13g01764.t01 | bHLH | chrUn.gff3_PROT_VIT_00s0824g00020.t01 | bHLH |
| g21386.t1 | bHLH | Vitvi13g01958.t01 | bHLH | chrUn.gff3_PROT_VIT_00s0927g00010.t01 | bHLH |
| g2164.t1 | bHLH | Vitvi14g00020.t01 | bHLH | chrUn.gff3_PROT_VIT_00s1312g00010.t01 | bHLH |
| g21968.t1 | bHLH | Vitvi14g00277.t01 | bHLH | chrUn.gff3_PROT_VIT_00s1314g00010.t01 | bHLH |
| g22149.t1 | bHLH | Vitvi14g00477.t01 | bHLH | chr1.gff3_PROT_VIT_01s0010g00930.t01 | bZIP |
| g22210.t1 | bHLH | Vitvi14g00562.t01 | bHLH | chr1.gff3_PROT_VIT_01s0011g03230.t01 | bZIP |
| g22210.t2 | bHLH | Vitvi14g00958.t01 | bHLH | chr12.gff3_PROT_VIT_12s0028g02590.t01 | bZIP |
| g22443.t1 | bHLH | Vitvi14g01181.t01 | bHLH | chr12.gff3_PROT_VIT_12s0034g00110.t01 | bZIP |
| g22703.t1 | bHLH | Vitvi14g01326.t01 | bHLH | chr12.gff3_PROT_VIT_12s0035g00620.t01 | bZIP |
| g22725.t1 | bHLH | Vitvi14g01473.t01 | bHLH | chr12.gff3_PROT_VIT_12s0055g00420.t01 | bZIP |
| g23265.t1 | bHLH | Vitvi14g01508.t01 | bHLH | chr13.gff3_PROT_VIT_13s0067g02900.t01 | bZIP |
| g23275.t1 | bHLH | Vitvi14g01926.t01 | bHLH | chr13.gff3_PROT_VIT_13s0084g00660.t01 | bZIP |
| g23607.t1 | bHLH | Vitvi14g01930.t01 | bHLH | chr13.gff3_PROT_VIT_13s0158g00380.t01 | bZIP |
| g23792.t1 | bHLH | Vitvi15g00254.t01 | bHLH | chr13.gff3_PROT_VIT_13s0175g00120.t01 | bZIP |
| g23867.t1 | bHLH | Vitvi15g00642.t01 | bHLH | chr14.gff3_PROT_VIT_14s0030g02200.t01 | bZIP |
| g23867.t2 | bHLH | Vitvi15g00876.t01 | bHLH | chr14.gff3_PROT_VIT_14s0060g01210.t01 | bZIP |
| g24211.t1 | bHLH | Vitvi15g00906.t01 | bHLH | chr14.gff3_PROT_VIT_14s0083g00700.t01 | bZIP |
| g24726.t1 | bHLH | Vitvi15g00948.t01 | bHLH | chr15.gff3_PROT_VIT_15s0046g01440.t01 | bZIP |
| g2479.t1 | bHLH | Vitvi15g01124.t01 | bHLH | chr18.gff3_PROT_VIT_18s0001g04470.t01 | bZIP |
| g25515.t1 | bHLH | Vitvi16g00882.t01 | bHLH | chr18.gff3_PROT_VIT_18s0001g04500.t01 | bZIP |
| g25621.t1 | bHLH | Vitvi16g00883.t01 | bHLH | chr18.gff3_PROT_VIT_18s0001g08710.t01 | bZIP |
| g25672.t1 | bHLH | Vitvi16g00885.t01 | bHLH | chr18.gff3_PROT_VIT_18s0001g10450.t01 | bZIP |
| g25739.t1 | bHLH | Vitvi16g01212.t01 | bHLH | chr18.gff3_PROT_VIT_18s0001g12120.t01 | bZIP |
| g26492.t1 | bHLH | Vitvi17g00037.t01 | bHLH | chr18.gff3_PROT_VIT_18s0001g13040.t01 | bZIP |
| g26681.t1 | bHLH | Vitvi17g00046.t01 | bHLH | chr18.gff3_PROT_VIT_18s0001g13740.t01 | bZIP |
| g26709.t1 | bHLH | Vitvi17g00307.t01 | bHLH | chr18.gff3_PROT_VIT_18s0001g14890.t01 | bZIP |
| g2675.t1 | bHLH | Vitvi17g00311.t01 | bHLH | chr18.gff3_PROT_VIT_18s0072g00470.t01 | bZIP |
| g26766.t1 | bHLH | Vitvi17g00442.t01 | bHLH | chr18.gff3_PROT_VIT_18s0076g00330.t01 | bZIP |
| g27041.t1 | bHLH | Vitvi17g00507.t01 | bHLH | chr18.gff3_PROT_VIT_18s0122g00500.t01 | bZIP |
| g27047.t1 | bHLH | Vitvi17g00569.t01 | bHLH | chr18_random.gff3_PROT_VIT_18s0001g03010.t01 | bZIP |
| g27051.t1 | bHLH | Vitvi17g00573.t01 | bHLH | chr19.gff3_PROT_VIT_19s0014g01780.t01 | bZIP |
| g27193.t1 | bHLH | Vitvi17g00678.t01 | bHLH | chr19.gff3_PROT_VIT_19s0015g01020.t01 | bZIP |
| g27303.t1 | bHLH | Vitvi17g00795.t01 | bHLH | chr2.gff3_PROT_VIT_02s0012g02250.t01 | bZIP |
| g27614.t1 | bHLH | Vitvi18g00463.t01 | bHLH | chr2.gff3_PROT_VIT_02s0025g01020.t01 | bZIP |
| g27703.t1 | bHLH | Vitvi18g00513.t01 | bHLH | chr3.gff3_PROT_VIT_03s0038g00860.t01 | bZIP |
| g278.t1 | bHLH | Vitvi18g00567.t01 | bHLH | chr3.gff3_PROT_VIT_03s0038g02420.t01 | bZIP |
| g27957.t1 | bHLH | Vitvi18g00617.t01 | bHLH | chr3.gff3_PROT_VIT_03s0038g04450.t01 | bZIP |
| g28186.t1 | bHLH | Vitvi18g00673.t01 | bHLH | chr3.gff3_PROT_VIT_03s0063g00310.t01 | bZIP |
| g28262.t1 | bHLH | Vitvi18g00763.t01 | bHLH | chr4.gff3_PROT_VIT_04s0008g02750.t01 | bZIP |
| g2828.t1 | bHLH | Vitvi18g00766.t01 | bHLH | chr4.gff3_PROT_VIT_04s0008g05210.t01 | bZIP |
| g28584.t1 | bHLH | Vitvi18g00779.t01 | bHLH | chr4.gff3_PROT_VIT_04s0023g01360.t01 | bZIP |
| g28752.t1 | bHLH | Vitvi18g00999.t01 | bHLH | chr4.gff3_PROT_VIT_04s0023g02430.t01 | bZIP |
| g28752.t2 | bHLH | Vitvi18g02008.t01 | bHLH | chr4.gff3_PROT_VIT_04s0069g01150.t01 | bZIP |
| g28753.t1 | bHLH | Vitvi19g00367.t01 | bHLH | chr5.gff3_PROT_VIT_05s0020g01090.t01 | bZIP |
| g29112.t1 | bHLH | Vitvi19g00406.t01 | bHLH | chr5.gff3_PROT_VIT_05s0077g01140.t01 | bZIP |
| g29990.t1 | bHLH | Vitvi01g00260.t01 | bZIP | chr5.gff3_PROT_VIT_05s0102g01120.t01 | bZIP |
| g30026.t1 | bHLH | Vitvi01g01342.t01 | bZIP | chr6.gff3_PROT_VIT_06s0004g08070.t01 | bZIP |
| g30150.t1 | bHLH | Vitvi02g00089.t01 | bZIP | chr6.gff3_PROT_VIT_06s0009g01790.t01 | bZIP |
| g30562.t1 | bHLH | Vitvi02g00796.t01 | bZIP | chr6.gff3_PROT_VIT_06s0080g00340.t01 | bZIP |
| g31285.t1 | bHLH | Vitvi03g00059.t01 | bZIP | chr6.gff3_PROT_VIT_06s0080g00360.t01 | bZIP |
| g31305.t1 | bHLH | Vitvi03g00146.t01 | bZIP | chr7.gff3_PROT_VIT_07s0005g01450.t01 | bZIP |
| g31700.t1 | bHLH | Vitvi03g00292.t01 | bZIP | chr7.gff3_PROT_VIT_07s0031g01320.t01 | bZIP |
| g3195.t1 | bHLH | Vitvi03g01513.t01 | bZIP | chr7.gff3_PROT_VIT_07s0141g00170.t01 | bZIP |
| g32177.t1 | bHLH | Vitvi03g01574.t01 | bZIP | chr8.gff3_PROT_VIT_08s0007g03420.t01 | bZIP |
| g32207.t1 | bHLH | Vitvi04g00240.t01 | bZIP | chr8.gff3_PROT_VIT_08s0007g03640.t01 | bZIP |
| g32365.t1 | bHLH | Vitvi04g00464.t01 | bZIP | chr8.gff3_PROT_VIT_08s0007g05170.t01 | bZIP |
| g32746.t1 | bHLH | Vitvi04g01363.t01 | bZIP | chr8.gff3_PROT_VIT_08s0007g06160.t01 | bZIP |
| g33153.t1 | bHLH | Vitvi04g01505.t01 | bZIP | chr8.gff3_PROT_VIT_08s0040g00870.t01 | bZIP |
| g33443.t1 | bHLH | Vitvi04g01992.t01 | bZIP | chrUn.gff3_PROT_VIT_00s0144g00240.t01 | bZIP |
| g33451.t1 | bHLH | Vitvi04g02098.t01 | bZIP | chrUn.gff3_PROT_VIT_00s0541g00020.t01 | bZIP |
| g34106.t1 | bHLH | Vitvi05g00108.t01 | bZIP | chr1.gff3_PROT_VIT_01s0010g02090.t01 | C2H2 |
| g34745.t1 | bHLH | Vitvi05g00274.t01 | bZIP | chr1.gff3_PROT_VIT_01s0010g02100.t01 | C2H2 |
| g35300.t1 | bHLH | Vitvi05g01537.t01 | bZIP | chr1.gff3_PROT_VIT_01s0011g00710.t01 | C2H2 |
| g35300.t2 | bHLH | Vitvi06g00685.t01 | bZIP | chr1.gff3_PROT_VIT_01s0011g03100.t01 | C2H2 |
| g36633.t1 | bHLH | Vitvi06g00800.t01 | bZIP | chr1.gff3_PROT_VIT_01s0011g05570.t01 | C2H2 |
| g37352.t1 | bHLH | Vitvi06g01073.t01 | bZIP | chr1.gff3_PROT_VIT_01s0011g06200.t01 | C2H2 |
| g37355.t1 | bHLH | Vitvi06g01478.t01 | bZIP | chr1.gff3_PROT_VIT_01s0011g06400.t01 | C2H2 |
| g37355.t2 | bHLH | Vitvi06g01480.t01 | bZIP | chr1.gff3_PROT_VIT_01s0026g00850.t01 | C2H2 |
| g3740.t1 | bHLH | Vitvi06g01755.t01 | bZIP | chr11.gff3_PROT_VIT_11s0052g01120.t01 | C2H2 |
| g38294.t1 | bHLH | Vitvi06g01756.t01 | bZIP | chr12.gff3_PROT_VIT_12s0028g03030.t01 | C2H2 |
| g38294.t2 | bHLH | Vitvi06g01757.t01 | bZIP | chr12.gff3_PROT_VIT_12s0035g00660.t01 | C2H2 |
| g4539.t1 | bHLH | Vitvi07g00049.t01 | bZIP | chr13.gff3_PROT_VIT_13s0019g00480.t01 | C2H2 |
| g4880.t1 | bHLH | Vitvi07g00413.t01 | bZIP | chr13.gff3_PROT_VIT_13s0019g02120.t01 | C2H2 |
| g542.t1 | bHLH | Vitvi07g01552.t01 | bZIP | chr13.gff3_PROT_VIT_13s0019g04530.t01 | C2H2 |
| g546.t1 | bHLH | Vitvi07g01807.t01 | bZIP | chr13.gff3_PROT_VIT_13s0019g05170.t01 | C2H2 |
| g6133.t1 | bHLH | Vitvi08g00950.t01 | bZIP | chr13.gff3_PROT_VIT_13s0064g01060.t01 | C2H2 |
| g6133.t2 | bHLH | Vitvi08g01470.t01 | bZIP | chr14.gff3_PROT_VIT_14s0060g00320.t01 | C2H2 |
| g636.t1 | bHLH | Vitvi08g01638.t01 | bZIP | chr14.gff3_PROT_VIT_14s0060g02440.t01 | C2H2 |
| g645.t1 | bHLH | Vitvi08g01710.t01 | bZIP | chr14.gff3_PROT_VIT_14s0066g01860.t01 | C2H2 |
| g7632.t1 | bHLH | Vitvi08g02272.t01 | bZIP | chr14.gff3_PROT_VIT_14s0068g01930.t01 | C2H2 |
| g7887.t1 | bHLH | Vitvi12g00226.t01 | bZIP | chr14.gff3_PROT_VIT_14s0219g00070.t01 | C2H2 |
| g857.t1 | bHLH | Vitvi12g01667.t01 | bZIP | chr15.gff3_PROT_VIT_15s0021g02720.t01 | C2H2 |
| g1020.t1 | bZIP | Vitvi12g01996.t01 | bZIP | chr16.gff3_PROT_VIT_16s0050g00700.t01 | C2H2 |
| g11688.t1 | bZIP | Vitvi12g02596.t01 | bZIP | chr17.gff3_PROT_VIT_17s0000g01870.t01 | C2H2 |
| g1182.t1 | bZIP | Vitvi13g00171.t01 | bZIP | chr17.gff3_PROT_VIT_17s0000g06820.t01 | C2H2 |
| g1183.t1 | bZIP | Vitvi13g01083.t01 | bZIP | chr17.gff3_PROT_VIT_17s0000g06830.t01 | C2H2 |
| g1184.t1 | bZIP | Vitvi13g01350.t01 | bZIP | chr17.gff3_PROT_VIT_17s0000g09980.t01 | C2H2 |
| g12754.t1 | bZIP | Vitvi13g01609.t01 | bZIP | chr18.gff3_PROT_VIT_18s0001g03670.t01 | C2H2 |
| g13490.t1 | bZIP | Vitvi14g00094.t01 | bZIP | chr18.gff3_PROT_VIT_18s0001g05860.t01 | C2H2 |
| g13609.t1 | bZIP | Vitvi14g00473.t01 | bZIP | chr18.gff3_PROT_VIT_18s0001g09230.t01 | C2H2 |
| g13627.t1 | bZIP | Vitvi14g00860.t01 | bZIP | chr18.gff3_PROT_VIT_18s0001g10130.t01 | C2H2 |
| g14064.t1 | bZIP | Vitvi14g00866.t01 | bZIP | chr18.gff3_PROT_VIT_18s0001g12220.t01 | C2H2 |
| g14343.t1 | bZIP | Vitvi14g01302.t01 | bZIP | chr18.gff3_PROT_VIT_18s0001g14130.t01 | C2H2 |
| g15403.t1 | bZIP | Vitvi15g01027.t01 | bZIP | chr18.gff3_PROT_VIT_18s0072g00840.t01 | C2H2 |
| g15493.t1 | bZIP | Vitvi16g01750.t01 | bZIP | chr19.gff3_PROT_VIT_19s0014g05000.t01 | C2H2 |
| g15543.t1 | bZIP | Vitvi17g00434.t01 | bZIP | chr19.gff3_PROT_VIT_19s0015g01780.t01 | C2H2 |
| g15543.t2 | bZIP | Vitvi17g01153.t01 | bZIP | chr2.gff3_PROT_VIT_02s0012g01190.t01 | C2H2 |
| g16040.t1 | bZIP | Vitvi17g01720.t01 | bZIP | chr2.gff3_PROT_VIT_02s0241g00170.t01 | C2H2 |
| g16309.t1 | bZIP | Vitvi18g00036.t01 | bZIP | chr3.gff3_PROT_VIT_03s0017g01020.t01 | C2H2 |
| g16311.t1 | bZIP | Vitvi18g00346.t01 | bZIP | chr3.gff3_PROT_VIT_03s0017g02130.t01 | C2H2 |
| g16599.t1 | bZIP | Vitvi18g00347.t01 | bZIP | chr3.gff3_PROT_VIT_03s0017g02160.t01 | C2H2 |
| g17209.t1 | bZIP | Vitvi18g00628.t01 | bZIP | chr3.gff3_PROT_VIT_03s0091g00690.t01 | C2H2 |
| g17369.t1 | bZIP | Vitvi18g00784.t01 | bZIP | chr3.gff3_PROT_VIT_03s0132g00020.t01 | C2H2 |
| g17395.t1 | bZIP | Vitvi18g00930.t01 | bZIP | chr4.gff3_PROT_VIT_04s0008g01160.t01 | C2H2 |
| g17395.t2 | bZIP | Vitvi18g01005.t01 | bZIP | chr4.gff3_PROT_VIT_04s0008g02540.t01 | C2H2 |
| g17549.t1 | bZIP | Vitvi18g01068.t01 | bZIP | chr4.gff3_PROT_VIT_04s0008g05930.t01 | C2H2 |
| g17629.t1 | bZIP | Vitvi18g01165.t01 | bZIP | chr4.gff3_PROT_VIT_04s0023g01430.t01 | C2H2 |
| g17941.t1 | bZIP | Vitvi18g01305.t01 | bZIP | chr5.gff3_PROT_VIT_05s0049g00070.t01 | C2H2 |
| g18104.t1 | bZIP | Vitvi18g01928.t01 | bZIP | chr5.gff3_PROT_VIT_05s0049g00080.t01 | C2H2 |
| g1876.t1 | bZIP | Vitvi18g02978.t01 | bZIP | chr5.gff3_PROT_VIT_05s0077g01390.t01 | C2H2 |
| g19394.t1 | bZIP | Vitvi19g00147.t01 | bZIP | chr6.gff3_PROT_VIT_06s0004g01870.t01 | C2H2 |
| g19395.t1 | bZIP | Vitvi19g02076.t01 | bZIP | chr6.gff3_PROT_VIT_06s0004g03070.t01 | C2H2 |
| g19397.t1 | bZIP | Vitvi01g00063.t01 | C2H2 | chr6.gff3_PROT_VIT_06s0004g04180.t01 | C2H2 |
| g19721.t1 | bZIP | Vitvi01g00247.t01 | C2H2 | chr6.gff3_PROT_VIT_06s0004g05260.t01 | C2H2 |
| g20404.t1 | bZIP | Vitvi01g00474.t01 | C2H2 | chr6.gff3_PROT_VIT_06s0004g05270.t01 | C2H2 |
| g20681.t1 | bZIP | Vitvi01g00529.t01 | C2H2 | chr6.gff3_PROT_VIT_06s0004g06240.t01 | C2H2 |
| g20972.t1 | bZIP | Vitvi01g00552.t01 | C2H2 | chr6.gff3_PROT_VIT_06s0009g02460.t01 | C2H2 |
| g24186.t1 | bZIP | Vitvi01g00845.t01 | C2H2 | chr6.gff3_PROT_VIT_06s0009g02470.t01 | C2H2 |
| g2438.t1 | bZIP | Vitvi01g01492.t01 | C2H2 | chr6.gff3_PROT_VIT_06s0061g00760.t01 | C2H2 |
| g24947.t1 | bZIP | Vitvi01g01493.t01 | C2H2 | chr6.gff3_PROT_VIT_06s0061g00780.t01 | C2H2 |
| g25302.t1 | bZIP | Vitvi01g02054.t01 | C2H2 | chr6.gff3_PROT_VIT_06s0061g01240.t01 | C2H2 |
| g25459.t1 | bZIP | Vitvi02g00488.t01 | C2H2 | chr6.gff3_PROT_VIT_06s0080g00270.t01 | C2H2 |
| g25618.t1 | bZIP | Vitvi02g00560.t01 | C2H2 | chr7.gff3_PROT_VIT_07s0005g02980.t01 | C2H2 |
| g2602.t1 | bZIP | Vitvi02g00686.t01 | C2H2 | chr7.gff3_PROT_VIT_07s0104g01620.t01 | C2H2 |
| g27612.t1 | bZIP | Vitvi03g00630.t01 | C2H2 | chr7.gff3_PROT_VIT_07s0129g00240.t01 | C2H2 |
| g27906.t1 | bZIP | Vitvi03g00904.t01 | C2H2 | chr7_random.gff3_PROT_VIT_07s0141g00020.t01 | C2H2 |
| g27906.t2 | bZIP | Vitvi03g01277.t01 | C2H2 | chr8.gff3_PROT_VIT_08s0007g03940.t01 | C2H2 |
| g27989.t1 | bZIP | Vitvi03g01280.t01 | C2H2 | chr8.gff3_PROT_VIT_08s0007g04770.t01 | C2H2 |
| g28197.t1 | bZIP | Vitvi03g01287.t01 | C2H2 | chr8.gff3_PROT_VIT_08s0007g08070.t01 | C2H2 |
| g28739.t1 | bZIP | Vitvi04g00096.t01 | C2H2 | chr8.gff3_PROT_VIT_08s0007g08360.t01 | C2H2 |
| g3021.t1 | bZIP | Vitvi04g00219.t01 | C2H2 | chr8.gff3_PROT_VIT_08s0007g09030.t01 | C2H2 |
| g3021.t2 | bZIP | Vitvi04g00527.t01 | C2H2 | chr8.gff3_PROT_VIT_08s0040g00240.t01 | C2H2 |
| g3021.t3 | bZIP | Vitvi04g01252.t01 | C2H2 | chr8.gff3_PROT_VIT_08s0040g00750.t01 | C2H2 |
| g30436.t1 | bZIP | Vitvi04g02200.t01 | C2H2 | chr8.gff3_PROT_VIT_08s0040g02100.t01 | C2H2 |
| g30960.t1 | bZIP | Vitvi05g00082.t01 | C2H2 | chr9.gff3_PROT_VIT_09s0002g02300.t01 | C2H2 |
| g31967.t1 | bZIP | Vitvi05g01913.t01 | C2H2 | chr9.gff3_PROT_VIT_09s0002g09080.t01 | C2H2 |
| g3227.t1 | bZIP | Vitvi05g01914.t01 | C2H2 | chr9.gff3_PROT_VIT_09s0054g01820.t01 | C2H2 |
| g34429.t1 | bZIP | Vitvi05g01915.t01 | C2H2 | chrUn.gff3_PROT_VIT_00s0179g00170.t01 | C2H2 |
| g34687.t1 | bZIP | Vitvi06g00195.t01 | C2H2 | chrUn.gff3_PROT_VIT_00s0229g00050.t01 | C2H2 |
| g34792.t1 | bZIP | Vitvi06g00304.t01 | C2H2 | chrUn.gff3_PROT_VIT_00s2480g00010.t01 | C2H2 |
| g3518.t1 | bZIP | Vitvi06g00521.t01 | C2H2 | chr1.gff3_PROT_VIT_01s0010g01770.t01 | C3H |
| g35228.t1 | bZIP | Vitvi06g00607.t01 | C2H2 | chr1.gff3_PROT_VIT_01s0011g04260.t01 | C3H |
| g35313.t1 | bZIP | Vitvi06g01350.t01 | C2H2 | chr10.gff3_PROT_VIT_10s0003g00110.t01 | C3H |
| g36698.t1 | bZIP | Vitvi06g01353.t01 | C2H2 | chr11.gff3_PROT_VIT_11s0016g02020.t01 | C3H |
| g36972.t1 | bZIP | Vitvi06g01399.t01 | C2H2 | chr11.gff3_PROT_VIT_11s0016g02130.t01 | C3H |
| g37545.t1 | bZIP | Vitvi06g01471.t01 | C2H2 | chr11.gff3_PROT_VIT_11s0016g02790.t01 | C3H |
| g773.t1 | bZIP | Vitvi06g01682.t01 | C2H2 | chr11.gff3_PROT_VIT_11s0016g04280.t01 | C3H |
| g865.t1 | bZIP | Vitvi06g01710.t01 | C2H2 | chr11.gff3_PROT_VIT_11s0016g04940.t01 | C3H |
| g924.t1 | bZIP | Vitvi06g01861.t01 | C2H2 | chr11.gff3_PROT_VIT_11s0016g04980.t01 | C3H |
| g11491.t1 | C2H2 | Vitvi06g01862.t01 | C2H2 | chr12.gff3_PROT_VIT_12s0028g03300.t01 | C3H |
| g11492.t1 | C2H2 | Vitvi06g01864.t01 | C2H2 | chr12.gff3_PROT_VIT_12s0035g01790.t01 | C3H |
| g11911.t1 | C2H2 | Vitvi06g01865.t01 | C2H2 | chr12.gff3_PROT_VIT_12s0059g01450.t01 | C3H |
| g12009.t1 | C2H2 | Vitvi06g01866.t01 | C2H2 | chr12.gff3_PROT_VIT_12s0059g02450.t01 | C3H |
| g12159.t1 | C2H2 | Vitvi06g01867.t01 | C2H2 | chr13.gff3_PROT_VIT_13s0064g00920.t01 | C3H |
| g1266.t1 | C2H2 | Vitvi07g00077.t01 | C2H2 | chr13.gff3_PROT_VIT_13s0064g01090.t01 | C3H |
| g13601.t1 | C2H2 | Vitvi07g00271.t01 | C2H2 | chr13.gff3_PROT_VIT_13s0067g01070.t01 | C3H |
| g13602.t1 | C2H2 | Vitvi07g01366.t01 | C2H2 | chr13.gff3_PROT_VIT_13s0156g00320.t01 | C3H |
| g1364.t1 | C2H2 | Vitvi07g01602.t01 | C2H2 | chr14.gff3_PROT_VIT_14s0006g02450.t01 | C3H |
| g1365.t1 | C2H2 | Vitvi07g01724.t01 | C2H2 | chr14.gff3_PROT_VIT_14s0036g00380.t01 | C3H |
| g13963.t1 | C2H2 | Vitvi07g02261.t01 | C2H2 | chr14.gff3_PROT_VIT_14s0060g00610.t01 | C3H |
| g13964.t1 | C2H2 | Vitvi07g02262.t01 | C2H2 | chr14.gff3_PROT_VIT_14s0219g00170.t01 | C3H |
| g13965.t1 | C2H2 | Vitvi07g03165.t01 | C2H2 | chr15.gff3_PROT_VIT_15s0046g01030.t01 | C3H |
| g13966.t1 | C2H2 | Vitvi08g00893.t01 | C2H2 | chr16.gff3_PROT_VIT_16s0013g00870.t01 | C3H |
| g13968.t1 | C2H2 | Vitvi08g01050.t01 | C2H2 | chr16.gff3_PROT_VIT_16s0022g02520.t01 | C3H |
| g13969.t1 | C2H2 | Vitvi08g01518.t01 | C2H2 | chr16.gff3_PROT_VIT_16s0039g01570.t01 | C3H |
| g14079.t1 | C2H2 | Vitvi08g01600.t01 | C2H2 | chr16.gff3_PROT_VIT_16s0100g00320.t01 | C3H |
| g14080.t1 | C2H2 | Vitvi08g01869.t01 | C2H2 | chr17.gff3_PROT_VIT_17s0000g09990.t01 | C3H |
| g14081.t1 | C2H2 | Vitvi08g01895.t01 | C2H2 | chr18.gff3_PROT_VIT_18s0001g15570.t01 | C3H |
| g14129.t1 | C2H2 | Vitvi08g02113.t01 | C2H2 | chr19.gff3_PROT_VIT_19s0085g01180.t01 | C3H |
| g14129.t2 | C2H2 | Vitvi08g02203.t01 | C2H2 | chr2.gff3_PROT_VIT_02s0012g01550.t01 | C3H |
| g14501.t1 | C2H2 | Vitvi09g00191.t01 | C2H2 | chr4.gff3_PROT_VIT_04s0008g00610.t01 | C3H |
| g1475.t1 | C2H2 | Vitvi09g00827.t01 | C2H2 | chr4.gff3_PROT_VIT_04s0023g00370.t01 | C3H |
| g15706.t1 | C2H2 | Vitvi09g01479.t01 | C2H2 | chr4.gff3_PROT_VIT_04s0023g02950.t01 | C3H |
| g15881.t1 | C2H2 | Vitvi11g01259.t01 | C2H2 | chr5.gff3_PROT_VIT_05s0020g01650.t01 | C3H |
| g16053.t1 | C2H2 | Vitvi12g00252.t01 | C2H2 | chr6.gff3_PROT_VIT_06s0004g01290.t01 | C3H |
| g16317.t1 | C2H2 | Vitvi12g02001.t01 | C2H2 | chr6.gff3_PROT_VIT_06s0004g02160.t01 | C3H |
| g1649.t1 | C2H2 | Vitvi13g00262.t01 | C2H2 | chr6.gff3_PROT_VIT_06s0004g06710.t01 | C3H |
| g16570.t1 | C2H2 | Vitvi13g00340.t01 | C2H2 | chr7.gff3_PROT_VIT_07s0031g01870.t01 | C3H |
| g16866.t1 | C2H2 | Vitvi13g00630.t01 | C2H2 | chr7.gff3_PROT_VIT_07s0104g01460.t01 | C3H |
| g17428.t1 | C2H2 | Vitvi13g00694.t01 | C2H2 | chr8.gff3_PROT_VIT_08s0007g06400.t01 | C3H |
| g17509.t1 | C2H2 | Vitvi13g01403.t01 | C2H2 | chr8.gff3_PROT_VIT_08s0007g07440.t01 | C3H |
| g1758.t1 | C2H2 | Vitvi13g01748.t01 | C2H2 | chr8.gff3_PROT_VIT_08s0007g08210.t01 | C3H |
| g17694.t1 | C2H2 | Vitvi14g00028.t01 | C2H2 | chr9.gff3_PROT_VIT_09s0002g00800.t01 | C3H |
| g17695.t2 | C2H2 | Vitvi14g00197.t01 | C2H2 | chr9.gff3_PROT_VIT_09s0002g06130.t01 | C3H |
| g17805.t1 | C2H2 | Vitvi14g01533.t01 | C2H2 | chr9.gff3_PROT_VIT_09s0070g00470.t01 | C3H |
| g17991.t1 | C2H2 | Vitvi14g01617.t01 | C2H2 | chrUn.gff3_PROT_VIT_00s0279g00070.t01 | C3H |
| g18076.t1 | C2H2 | Vitvi14g01822.t01 | C2H2 | chr1.gff3_PROT_VIT_01s0010g03850.t01 | CAMTA |
| g18280.t1 | C2H2 | Vitvi14g02033.t01 | C2H2 | chr5.gff3_PROT_VIT_05s0020g00630.t01 | CAMTA |
| g18417.t1 | C2H2 | Vitvi14g02957.t01 | C2H2 | chr5.gff3_PROT_VIT_05s0077g01240.t01 | CAMTA |
| g18487.t1 | C2H2 | Vitvi15g01472.t01 | C2H2 | chr7_random.gff3_PROT_VIT_07s0141g00250.t01 | CAMTA |
| g19029.t1 | C2H2 | Vitvi16g00499.t01 | C2H2 | chr1.gff3_PROT_VIT_01s0011g03520.t01 | CO-like |
| g19031.t1 | C2H2 | Vitvi16g01067.t01 | C2H2 | chr1.gff3_PROT_VIT_01s0146g00360.t01 | CO-like |
| g19086.t1 | C2H2 | Vitvi17g00157.t01 | C2H2 | chr11.gff3_PROT_VIT_11s0052g01800.t01 | CO-like |
| g19262.t1 | C2H2 | Vitvi17g00670.t01 | C2H2 | chr12.gff3_PROT_VIT_12s0057g01350.t01 | CO-like |
| g19352.t1 | C2H2 | Vitvi17g01001.t01 | C2H2 | chr12.gff3_PROT_VIT_12s0059g02500.t01 | CO-like |
| g19478.t1 | C2H2 | Vitvi17g01488.t01 | C2H2 | chr14.gff3_PROT_VIT_14s0068g01380.t01 | CO-like |
| g19865.t1 | C2H2 | Vitvi18g00316.t01 | C2H2 | chr14.gff3_PROT_VIT_14s0083g00640.t01 | CO-like |
| g19865.t2 | C2H2 | Vitvi18g00417.t01 | C2H2 | chr4.gff3_PROT_VIT_04s0008g07340.t01 | CO-like |
| g20005.t1 | C2H2 | Vitvi18g00675.t01 | C2H2 | chr7.gff3_PROT_VIT_07s0104g01360.t01 | CO-like |
| g20099.t1 | C2H2 | Vitvi18g00755.t01 | C2H2 | chrUn.gff3_PROT_VIT_00s0194g00070.t01 | CO-like |
| g20508.t1 | C2H2 | Vitvi18g00935.t01 | C2H2 | chr11.gff3_PROT_VIT_11s0016g03680.t01 | CPP |
| g21936.t1 | C2H2 | Vitvi18g01109.t01 | C2H2 | chr14.gff3_PROT_VIT_14s0030g01800.t01 | CPP |
| g23096.t1 | C2H2 | Vitvi18g01662.t01 | C2H2 | chr4.gff3_PROT_VIT_04s0008g03670.t01 | CPP |
| g23990.t1 | C2H2 | Vitvi19g00397.t01 | C2H2 | chr4.gff3_PROT_VIT_04s0008g05130.t01 | CPP |
| g23990.t2 | C2H2 | Vitvi19g00912.t01 | C2H2 | chr5.gff3_PROT_VIT_05s0020g03880.t01 | CPP |
| g24399.t1 | C2H2 | Vitvi01g00347.t01 | C3H | chr9.gff3_PROT_VIT_09s0002g06020.t01 | CPP |
| g24458.t1 | C2H2 | Vitvi01g01447.t01 | C3H | chr18.gff3_PROT_VIT_18s0001g13520.t01 | DBB |
| g24899.t1 | C2H2 | Vitvi02g00715.t01 | C3H | chr18.gff3_PROT_VIT_18s0089g01280.t01 | DBB |
| g25394.t1 | C2H2 | Vitvi04g00047.t01 | C3H | chr19.gff3_PROT_VIT_19s0014g00350.t01 | DBB |
| g25395.t1 | C2H2 | Vitvi04g01157.t01 | C3H | chr3.gff3_PROT_VIT_03s0038g00340.t01 | DBB |
| g25753.t1 | C2H2 | Vitvi05g00333.t01 | C3H | chr3.gff3_PROT_VIT_03s0038g00690.t01 | DBB |
| g25813.t1 | C2H2 | Vitvi06g00123.t01 | C3H | chr4.gff3_PROT_VIT_04s0023g03030.t01 | DBB |
| g26802.t1 | C2H2 | Vitvi06g00218.t01 | C3H | chr5.gff3_PROT_VIT_05s0102g00750.t01 | DBB |
| g26856.t1 | C2H2 | Vitvi06g00504.t01 | C3H | chr1.gff3_PROT_VIT_01s0026g02580.t01 | Dof |
| g27150.t1 | C2H2 | Vitvi06g00653.t01 | C3H | chr10.gff3_PROT_VIT_10s0003g00030.t01 | Dof |
| g2742.t1 | C2H2 | Vitvi07g00262.t01 | C3H | chr10.gff3_PROT_VIT_10s0003g00040.t01 | Dof |
| g27454.t1 | C2H2 | Vitvi07g01864.t01 | C3H | chr10.gff3_PROT_VIT_10s0003g01260.t01 | Dof |
| g28576.t1 | C2H2 | Vitvi07g02693.t01 | C3H | chr13.gff3_PROT_VIT_13s0019g01410.t01 | Dof |
| g28648.t1 | C2H2 | Vitvi08g01732.t01 | C3H | chr14.gff3_PROT_VIT_14s0108g00980.t01 | Dof |
| g30002.t1 | C2H2 | Vitvi08g01816.t01 | C3H | chr15.gff3_PROT_VIT_15s0046g00150.t01 | Dof |
| g30414.t1 | C2H2 | Vitvi08g01880.t01 | C3H | chr16.gff3_PROT_VIT_16s0098g01420.t01 | Dof |
| g30654.t1 | C2H2 | Vitvi09g00059.t01 | C3H | chr17.gff3_PROT_VIT_17s0000g04850.t01 | Dof |
| g31609.t1 | C2H2 | Vitvi09g00531.t01 | C3H | chr17.gff3_PROT_VIT_17s0000g06310.t01 | Dof |
| g31841.t1 | C2H2 | Vitvi09g01011.t01 | C3H | chr17.gff3_PROT_VIT_17s0000g08290.t01 | Dof |
| g3252.t1 | C2H2 | Vitvi10g00486.t01 | C3H | chr18.gff3_PROT_VIT_18s0001g11310.t01 | Dof |
| g33230.t1 | C2H2 | Vitvi11g00170.t01 | C3H | chr18.gff3_PROT_VIT_18s0001g15730.t01 | Dof |
| g3345.t1 | C2H2 | Vitvi11g00229.t01 | C3H | chr2.gff3_PROT_VIT_02s0025g02250.t01 | Dof |
| g33635.t1 | C2H2 | Vitvi11g00372.t01 | C3H | chr3.gff3_PROT_VIT_03s0063g01350.t01 | Dof |
| g33635.t2 | C2H2 | Vitvi11g00436.t01 | C3H | chr6.gff3_PROT_VIT_06s0004g03420.t01 | Dof |
| g33750.t1 | C2H2 | Vitvi11g00440.t01 | C3H | chr6.gff3_PROT_VIT_06s0004g04520.t01 | Dof |
| g340.t1 | C2H2 | Vitvi11g01367.t01 | C3H | chr7.gff3_PROT_VIT_07s0255g00020.t01 | Dof |
| g34094.t1 | C2H2 | Vitvi12g00276.t01 | C3H | chr8.gff3_PROT_VIT_08s0007g00180.t01 | Dof |
| g34116.t1 | C2H2 | Vitvi12g00449.t01 | C3H | chr8.gff3_PROT_VIT_08s0056g01230.t01 | Dof |
| g34501.t1 | C2H2 | Vitvi12g00535.t01 | C3H | chr8.gff3_PROT_VIT_08s0105g00170.t01 | Dof |
| g34502.t1 | C2H2 | Vitvi12g02121.t01 | C3H | chr9.gff3_PROT_VIT_09s0002g02490.t01 | Dof |
| g34681.t1 | C2H2 | Vitvi13g00058.t01 | C3H | chrUn.gff3_PROT_VIT_00s0218g00040.t01 | Dof |
| g34786.t1 | C2H2 | Vitvi13g01732.t01 | C3H | chrUn.gff3_PROT_VIT_00s0253g00060.t01 | Dof |
| g3487.t1 | C2H2 | Vitvi13g01750.t01 | C3H | chrUn.gff3_PROT_VIT_00s0652g00010.t01 | Dof |
| g35797.t1 | C2H2 | Vitvi13g01819.t01 | C3H | chr14.gff3_PROT_VIT_14s0066g02370.t01 | E2F/DP |
| g36508.t1 | C2H2 | Vitvi14g00599.t01 | C3H | chr17.gff3_PROT_VIT_17s0000g07630.t01 | E2F/DP |
| g36513.t1 | C2H2 | Vitvi14g01114.t01 | C3H | chr18.gff3_PROT_VIT_18s0001g14110.t01 | E2F/DP |
| g36517.t1 | C2H2 | Vitvi14g01629.t01 | C3H | chr6.gff3_PROT_VIT_06s0080g01110.t01 | E2F/DP |
| g36519.t1 | C2H2 | Vitvi14g02452.t01 | C3H | chr8.gff3_PROT_VIT_08s0007g00170.t01 | E2F/DP |
| g36703.t1 | C2H2 | Vitvi15g00994.t01 | C3H | chr8.gff3_PROT_VIT_08s0007g02510.t01 | E2F/DP |
| g3698.t1 | C2H2 | Vitvi16g00084.t01 | C3H | chr8.gff3_PROT_VIT_08s0040g01330.t01 | E2F/DP |
| g4968.t1 | C2H2 | Vitvi16g00346.t01 | C3H | chr13.gff3_PROT_VIT_13s0047g00250.t01 | EIL |
| g7755.t1 | C2H2 | Vitvi16g00933.t01 | C3H | chr6.gff3_PROT_VIT_06s0004g01610.t01 | EIL |
| g783.t1 | C2H2 | Vitvi16g01601.t01 | C3H | chr6.gff3_PROT_VIT_06s0009g01380.t01 | EIL |
| g960.t1 | C2H2 | Vitvi16g01835.t01 | C3H | chrUn.gff3_PROT_VIT_00s0357g00120.t01 | EIL |
| g11628.t1 | C3H | Vitvi17g01002.t01 | C3H | chr1.gff3_PROT_VIT_01s0011g03470.t01 | ERF |
| g11879.t1 | C3H | Vitvi18g01240.t01 | C3H | chr1.gff3_PROT_VIT_01s0150g00120.t01 | ERF |
| g1218.t1 | C3H | Vitvi19g01773.t01 | C3H | chr10.gff3_PROT_VIT_10s0003g00130.t01 | ERF |
| g12212.t1 | C3H | Vitvi01g01671.t01 | CAMTA | chr10.gff3_PROT_VIT_10s0003g00140.t01 | ERF |
| g12998.t1 | C3H | Vitvi05g00096.t01 | CAMTA | chr10.gff3_PROT_VIT_10s0003g00580.t01 | ERF |
| g136.t1 | C3H | Vitvi05g00239.t01 | CAMTA | chr10.gff3_PROT_VIT_10s0003g00590.t01 | ERF |
| g14468.t1 | C3H | Vitvi14g00629.t01 | CAMTA | chr11.gff3_PROT_VIT_11s0016g00660.t01 | ERF |
| g14572.t1 | C3H | Vitvi01g00288.t01 | CO-like | chr11.gff3_PROT_VIT_11s0016g00670.t01 | ERF |
| g14919.t1 | C3H | Vitvi01g01729.t01 | CO-like | chr11.gff3_PROT_VIT_11s0016g02140.t01 | ERF |
| g1492.t1 | C3H | Vitvi04g00665.t01 | CO-like | chr11.gff3_PROT_VIT_11s0016g03350.t01 | ERF |
| g15743.t1 | C3H | Vitvi07g00252.t01 | CO-like | chr11.gff3_PROT_VIT_11s0016g05340.t01 | ERF |
| g16327.t1 | C3H | Vitvi10g00219.t01 | CO-like | chr12.gff3_PROT_VIT_12s0028g03270.t01 | ERF |
| g17081.t1 | C3H | Vitvi11g01309.t01 | CO-like | chr12.gff3_PROT_VIT_12s0059g00280.t01 | ERF |
| g17653.t1 | C3H | Vitvi12g00542.t01 | CO-like | chr12.gff3_PROT_VIT_12s0059g01460.t01 | ERF |
| g17747.t1 | C3H | Vitvi12g00757.t01 | CO-like | chr13.gff3_PROT_VIT_13s0067g01400.t01 | ERF |
| g17821.t1 | C3H | Vitvi14g01296.t01 | CO-like | chr13.gff3_PROT_VIT_13s0067g01960.t01 | ERF |
| g18403.t1 | C3H | Vitvi14g01487.t01 | CO-like | chr14.gff3_PROT_VIT_14s0006g02290.t01 | ERF |
| g18420.t1 | C3H | Vitvi17g00328.t01 | CO-like | chr14.gff3_PROT_VIT_14s0068g00860.t01 | ERF |
| g19954.t1 | C3H | Vitvi19g00408.t01 | CO-like | chr14.gff3_PROT_VIT_14s0068g01040.t01 | ERF |
| g20338.t1 | C3H | Vitvi04g00320.t01 | CPP | chr14.gff3_PROT_VIT_14s0081g00520.t01 | ERF |
| g21012.t1 | C3H | Vitvi04g00455.t01 | CPP | chr14.gff3_PROT_VIT_14s0081g00730.t01 | ERF |
| g21494.t1 | C3H | Vitvi05g00541.t01 | CPP | chr14.gff3_PROT_VIT_14s0108g01220.t01 | ERF |
| g21735.t1 | C3H | Vitvi09g00519.t01 | CPP | chr15.gff3_PROT_VIT_15s0021g01590.t01 | ERF |
| g2242.t1 | C3H | Vitvi11g00314.t01 | CPP | chr15.gff3_PROT_VIT_15s0021g01600.t01 | ERF |
| g2312.t1 | C3H | Vitvi14g00428.t01 | CPP | chr15.gff3_PROT_VIT_15s0021g01610.t01 | ERF |
| g2316.t1 | C3H | Vitvi03g00026.t01 | DBB | chr15.gff3_PROT_VIT_15s0021g01630.t01 | ERF |
| g23341.t1 | C3H | Vitvi03g00049.t01 | DBB | chr15.gff3_PROT_VIT_15s0021g02110.t01 | ERF |
| g23494.t1 | C3H | Vitvi04g01423.t01 | DBB | chr15.gff3_PROT_VIT_15s0046g00310.t01 | ERF |
| g23594.t1 | C3H | Vitvi05g01519.t01 | DBB | chr15.gff3_PROT_VIT_15s0046g01390.t01 | ERF |
| g24563.t1 | C3H | Vitvi18g01048.t01 | DBB | chr16.gff3_PROT_VIT_16s0013g00890.t01 | ERF |
| g24679.t1 | C3H | Vitvi18g02424.t01 | DBB | chr16.gff3_PROT_VIT_16s0013g00900.t01 | ERF |
| g25171.t1 | C3H | Vitvi19g00031.t01 | DBB | chr16.gff3_PROT_VIT_16s0013g00950.t01 | ERF |
| g26472.t1 | C3H | Vitvi01g01026.t01 | Dof | chr16.gff3_PROT_VIT_16s0013g00970.t01 | ERF |
| g2657.t1 | C3H | Vitvi02g00199.t01 | Dof | chr16.gff3_PROT_VIT_16s0013g00980.t01 | ERF |
| g28378.t1 | C3H | Vitvi03g00430.t01 | Dof | chr16.gff3_PROT_VIT_16s0013g00990.t01 | ERF |
| g28560.t1 | C3H | Vitvi06g00345.t01 | Dof | chr16.gff3_PROT_VIT_16s0013g01000.t01 | ERF |
| g28649.t1 | C3H | Vitvi06g00449.t01 | Dof | chr16.gff3_PROT_VIT_16s0013g01050.t01 | ERF |
| g29243.t1 | C3H | Vitvi07g01298.t01 | Dof | chr16.gff3_PROT_VIT_16s0013g01060.t01 | ERF |
| g29869.t1 | C3H | Vitvi07g01306.t01 | Dof | chr16.gff3_PROT_VIT_16s0013g01070.t01 | ERF |
| g30129.t1 | C3H | Vitvi07g01401.t01 | Dof | chr16.gff3_PROT_VIT_16s0013g01090.t01 | ERF |
| g32251.t1 | C3H | Vitvi08g00110.t01 | Dof | chr16.gff3_PROT_VIT_16s0013g01110.t01 | ERF |
| g32454.t1 | C3H | Vitvi08g00626.t01 | Dof | chr16.gff3_PROT_VIT_16s0013g01120.t01 | ERF |
| g3286.t1 | C3H | Vitvi08g01186.t01 | Dof | chr16.gff3_PROT_VIT_16s0050g02400.t01 | ERF |
| g37338.t1 | C3H | Vitvi09g00210.t01 | Dof | chr16.gff3_PROT_VIT_16s0100g00380.t01 | ERF |
| g38221.t1 | C3H | Vitvi10g00444.t01 | Dof | chr16.gff3_PROT_VIT_16s0100g00400.t01 | ERF |
| g38286.t1 | C3H | Vitvi10g00479.t01 | Dof | chr17.gff3_PROT_VIT_17s0000g00200.t01 | ERF |
| g38298.t1 | C3H | Vitvi10g00480.t01 | Dof | chr17.gff3_PROT_VIT_17s0000g04480.t01 | ERF |
| g3906.t1 | C3H | Vitvi10g00581.t01 | Dof | chr17.gff3_PROT_VIT_17s0000g07940.t01 | ERF |
| g4166.t1 | C3H | Vitvi13g00298.t01 | Dof | chr18.gff3_PROT_VIT_18s0001g03240.t01 | ERF |
| g4911.t1 | C3H | Vitvi14g01973.t01 | Dof | chr18.gff3_PROT_VIT_18s0001g05250.t01 | ERF |
| g2430.t1 | CAMTA | Vitvi15g00936.t01 | Dof | chr18.gff3_PROT_VIT_18s0001g10150.t01 | ERF |
| g2557.t1 | CAMTA | Vitvi16g01384.t01 | Dof | chr18.gff3_PROT_VIT_18s0001g13320.t01 | ERF |
| g33239.t1 | CAMTA | Vitvi17g00447.t01 | Dof | chr18.gff3_PROT_VIT_18s0072g00260.t01 | ERF |
| g33239.t2 | CAMTA | Vitvi17g00611.t01 | Dof | chr18.gff3_PROT_VIT_18s0089g01030.t01 | ERF |
| g35671.t1 | CAMTA | Vitvi17g00809.t01 | Dof | chr18_random.gff3_PROT_VIT_18s0001g05850.t01 | ERF |
| g35671.t2 | CAMTA | Vitvi18g00858.t01 | Dof | chr18_random.gff3_PROT_VIT_18s0001g05890.t01 | ERF |
| g35671.t3 | CAMTA | Vitvi18g01254.t01 | Dof | chr19.gff3_PROT_VIT_19s0014g02240.t01 | ERF |
| g11750.t1 | CO-like | Vitvi06g01552.t01 | E2F/DP | chr19.gff3_PROT_VIT_19s0014g03180.t01 | ERF |
| g1503.t1 | CO-like | Vitvi08g00987.t01 | E2F/DP | chr19.gff3_PROT_VIT_19s0090g01080.t01 | ERF |
| g16008.t1 | CO-like | Vitvi08g01185.t01 | E2F/DP | chr2.gff3_PROT_VIT_02s0025g01360.t01 | ERF |
| g23250.t1 | CO-like | Vitvi08g01388.t01 | E2F/DP | chr2.gff3_PROT_VIT_02s0025g03170.t01 | ERF |
| g23599.t1 | CO-like | Vitvi14g01870.t01 | E2F/DP | chr2.gff3_PROT_VIT_02s0025g04440.t01 | ERF |
| g24176.t1 | CO-like | Vitvi17g00736.t01 | E2F/DP | chr2.gff3_PROT_VIT_02s0025g04460.t01 | ERF |
| g28586.t1 | CO-like | Vitvi18g01106.t01 | E2F/DP | chr2.gff3_PROT_VIT_02s0234g00130.t01 | ERF |
| g28863.t1 | CO-like | Vitvi06g00166.t01 | EIL | chr3.gff3_PROT_VIT_03s0063g00460.t01 | ERF |
| g30008.t1 | CO-like | Vitvi06g01036.t01 | EIL | chr4.gff3_PROT_VIT_04s0008g02230.t01 | ERF |
| g30943.t1 | CO-like | Vitvi07g01353.t01 | EIL | chr4.gff3_PROT_VIT_04s0008g03400.t01 | ERF |
| g31129.t1 | CO-like | Vitvi13g01126.t01 | EIL | chr4.gff3_PROT_VIT_04s0008g05440.t01 | ERF |
| g4680.t1 | CO-like | Vitvi00g00859.t01 | ERF | chr4.gff3_PROT_VIT_04s0008g06000.t01 | ERF |
| g13253.t1 | CPP | Vitvi01g00645.t01 | ERF | chr4.gff3_PROT_VIT_04s0023g00970.t01 | ERF |
| g16749.t1 | CPP | Vitvi01g01826.t01 | ERF | chr4.gff3_PROT_VIT_04s0043g00510.t01 | ERF |
| g2172.t1 | CPP | Vitvi01g01827.t01 | ERF | chr5.gff3_PROT_VIT_05s0029g00140.t01 | ERF |
| g2172.t2 | CPP | Vitvi02g00093.t01 | ERF | chr5.gff3_PROT_VIT_05s0049g00490.t01 | ERF |
| g25312.t1 | CPP | Vitvi02g00121.t01 | ERF | chr5.gff3_PROT_VIT_05s0049g00500.t01 | ERF |
| g30858.t1 | CPP | Vitvi02g00286.t01 | ERF | chr5.gff3_PROT_VIT_05s0049g00510.t01 | ERF |
| g30858.t2 | CPP | Vitvi02g00406.t01 | ERF | chr5.gff3_PROT_VIT_05s0077g01860.t01 | ERF |
| g3609.t1 | CPP | Vitvi02g00407.t01 | ERF | chr6.gff3_PROT_VIT_06s0004g00490.t01 | ERF |
| g15162.t1 | DBB | Vitvi02g01729.t01 | ERF | chr6.gff3_PROT_VIT_06s0004g08190.t01 | ERF |
| g16199.t1 | DBB | Vitvi02g01730.t01 | ERF | chr6.gff3_PROT_VIT_06s0061g01390.t01 | ERF |
| g24962.t1 | DBB | Vitvi02g01780.t01 | ERF | chr6.gff3_PROT_VIT_06s0061g01400.t01 | ERF |
| g25555.t1 | DBB | Vitvi03g00500.t01 | ERF | chr7.gff3_PROT_VIT_07s0005g00820.t01 | ERF |
| g26355.t1 | DBB | Vitvi04g00190.t01 | ERF | chr7.gff3_PROT_VIT_07s0005g03190.t01 | ERF |
| g905.t1 | DBB | Vitvi04g00300.t01 | ERF | chr7.gff3_PROT_VIT_07s0005g03200.t01 | ERF |
| g1447.t1 | Dof | Vitvi04g00479.t01 | ERF | chr7.gff3_PROT_VIT_07s0005g03210.t01 | ERF |
| g14756.t1 | Dof | Vitvi04g00533.t01 | ERF | chr7.gff3_PROT_VIT_07s0005g03220.t01 | ERF |
| g14774.t1 | Dof | Vitvi04g00994.t01 | ERF | chr7.gff3_PROT_VIT_07s0005g03230.t01 | ERF |
| g14939.t1 | Dof | Vitvi04g01765.t01 | ERF | chr7.gff3_PROT_VIT_07s0005g03260.t01 | ERF |
| g16486.t1 | Dof | Vitvi04g02088.t01 | ERF | chr7.gff3_PROT_VIT_07s0005g03270.t01 | ERF |
| g1691.t1 | Dof | Vitvi05g00334.t01 | ERF | chr7.gff3_PROT_VIT_07s0005g03280.t01 | ERF |
| g20246.t1 | Dof | Vitvi05g00715.t01 | ERF | chr7.gff3_PROT_VIT_07s0031g00190.t01 | ERF |
| g20776.t1 | Dof | Vitvi05g01073.t01 | ERF | chr7.gff3_PROT_VIT_07s0031g00420.t01 | ERF |
| g22619.t1 | Dof | Vitvi05g01722.t01 | ERF | chr7.gff3_PROT_VIT_07s0031g00710.t01 | ERF |
| g24832.t1 | Dof | Vitvi05g01723.t01 | ERF | chr7.gff3_PROT_VIT_07s0031g00720.t01 | ERF |
| g26745.t1 | Dof | Vitvi05g01724.t01 | ERF | chr7.gff3_PROT_VIT_07s0031g01980.t01 | ERF |
| g26745.t2 | Dof | Vitvi06g00052.t01 | ERF | chr7.gff3_PROT_VIT_07s0141g00690.t01 | ERF |
| g27088.t1 | Dof | Vitvi06g00810.t01 | ERF | chr8.gff3_PROT_VIT_08s0007g03790.t01 | ERF |
| g2900.t1 | Dof | Vitvi06g01411.t01 | ERF | chr8.gff3_PROT_VIT_08s0007g03810.t01 | ERF |
| g29101.t1 | Dof | Vitvi06g01414.t01 | ERF | chr8.gff3_PROT_VIT_08s0007g07250.t01 | ERF |
| g29250.t1 | Dof | Vitvi07g00357.t01 | ERF | chr8.gff3_PROT_VIT_08s0007g08150.t01 | ERF |
| g29251.t1 | Dof | Vitvi07g00590.t01 | ERF | chr8.gff3_PROT_VIT_08s0058g00520.t01 | ERF |
| g29390.t1 | Dof | Vitvi07g00591.t01 | ERF | chr9.gff3_PROT_VIT_09s0002g00470.t01 | ERF |
| g29731.t1 | Dof | Vitvi07g00592.t01 | ERF | chr9.gff3_PROT_VIT_09s0002g03940.t01 | ERF |
| g31883.t1 | Dof | Vitvi07g01702.t01 | ERF | chr9.gff3_PROT_VIT_09s0002g06750.t01 | ERF |
| g32379.t1 | Dof | Vitvi07g01755.t01 | ERF | chr9.gff3_PROT_VIT_09s0002g08830.t01 | ERF |
| g35692.t1 | Dof | Vitvi07g01874.t01 | ERF | chr9.gff3_PROT_VIT_09s0002g09120.t01 | ERF |
| g3721.t1 | Dof | Vitvi07g02062.t01 | ERF | chr9.gff3_PROT_VIT_09s0002g09140.t01 | ERF |
| g702.t1 | Dof | Vitvi07g02063.t01 | ERF | chrUn.gff3_PROT_VIT_00s0184g00010.t01 | ERF |
| g16240.t1 | E2F/DP | Vitvi07g02064.t01 | ERF | chrUn.gff3_PROT_VIT_00s0291g00030.t01 | ERF |
| g17276.t1 | E2F/DP | Vitvi07g02065.t01 | ERF | chrUn.gff3_PROT_VIT_00s0341g00070.t01 | ERF |
| g24831.t1 | E2F/DP | Vitvi07g02066.t01 | ERF | chrUn.gff3_PROT_VIT_00s0341g00080.t01 | ERF |
| g26968.t1 | E2F/DP | Vitvi07g02067.t01 | ERF | chrUn.gff3_PROT_VIT_00s0632g00010.t01 | ERF |
| g26968.t2 | E2F/DP | Vitvi07g02068.t01 | ERF | chrUn.gff3_PROT_VIT_00s0662g00030.t01 | ERF |
| g2822.t1 | E2F/DP | Vitvi07g02069.t01 | ERF | chrUn.gff3_PROT_VIT_00s0662g00040.t01 | ERF |
| g29163.t1 | E2F/DP | Vitvi07g02070.t01 | ERF | chr1.gff3_PROT_VIT_01s0011g01470.t01 | FAR1 |
| g29163.t2 | E2F/DP | Vitvi07g02662.t01 | ERF | chr12.gff3_PROT_VIT_12s0035g01360.t01 | FAR1 |
| g958.t1 | E2F/DP | Vitvi07g03029.t01 | ERF | chr12.gff3_PROT_VIT_12s0057g00960.t01 | FAR1 |
| g958.t2 | E2F/DP | Vitvi07g03030.t01 | ERF | chr13.gff3_PROT_VIT_13s0047g00330.t01 | FAR1 |
| g14530.t1 | EIL | Vitvi08g00778.t01 | ERF | chr14.gff3_PROT_VIT_14s0068g00770.t01 | FAR1 |
| g14530.t2 | EIL | Vitvi08g01501.t01 | ERF | chr14.gff3_PROT_VIT_14s0108g01540.t01 | FAR1 |
| g18867.t1 | EIL | Vitvi08g01861.t01 | ERF | chr18.gff3_PROT_VIT_18s0117g00140.t01 | FAR1 |
| g33271.t1 | EIL | Vitvi08g01965.t01 | ERF | chr18.gff3_PROT_VIT_18s0117g00390.t01 | FAR1 |
| g33975.t1 | EIL | Vitvi08g01966.t01 | ERF | chr18.gff3_PROT_VIT_18s0122g00530.t01 | FAR1 |
| g11745.t1 | ERF | Vitvi09g00031.t01 | ERF | chr18_random.gff3_PROT_VIT_18s0001g06550.t01 | FAR1 |
| g11885.t1 | ERF | Vitvi09g00323.t01 | ERF | chr18_random.gff3_PROT_VIT_18s0001g06680.t01 | FAR1 |
| g11887.t1 | ERF | Vitvi09g00593.t01 | ERF | chr3.gff3_PROT_VIT_03s0017g01780.t01 | FAR1 |
| g12995.t1 | ERF | Vitvi09g00803.t01 | ERF | chr3.gff3_PROT_VIT_03s0017g01810.t01 | FAR1 |
| g13650.t1 | ERF | Vitvi09g00834.t01 | ERF | chr3.gff3_PROT_VIT_03s0017g01830.t01 | FAR1 |
| g14258.t1 | ERF | Vitvi09g00837.t01 | ERF | chr3.gff3_PROT_VIT_03s0038g04140.t01 | FAR1 |
| g15357.t1 | ERF | Vitvi10g00488.t01 | ERF | chr4.gff3_PROT_VIT_04s0044g01920.t01 | FAR1 |
| g15575.t1 | ERF | Vitvi10g00521.t01 | ERF | chr5.gff3_PROT_VIT_05s0020g02450.t01 | FAR1 |
| g15575.t2 | ERF | Vitvi10g00522.t01 | ERF | chr5.gff3_PROT_VIT_05s0077g01400.t01 | FAR1 |
| g15854.t1 | ERF | Vitvi10g01594.t01 | ERF | chr5.gff3_PROT_VIT_05s0102g00390.t01 | FAR1 |
| g164.t1 | ERF | Vitvi11g00045.t01 | ERF | chr5.gff3_PROT_VIT_05s0102g00400.t01 | FAR1 |
| g16423.t1 | ERF | Vitvi11g00046.t01 | ERF | chr5.gff3_PROT_VIT_05s0102g00410.t01 | FAR1 |
| g17409.t1 | ERF | Vitvi11g00285.t01 | ERF | chr5.gff3_PROT_VIT_05s0102g00440.t01 | FAR1 |
| g17411.t1 | ERF | Vitvi11g00467.t01 | ERF | chr5.gff3_PROT_VIT_05s0102g00470.t01 | FAR1 |
| g17731.t1 | ERF | Vitvi12g00274.t01 | ERF | chr5.gff3_PROT_VIT_05s0102g00690.t01 | FAR1 |
| g18077.t1 | ERF | Vitvi12g00348.t01 | ERF | chr7.gff3_PROT_VIT_07s0005g01120.t01 | FAR1 |
| g19103.t1 | ERF | Vitvi12g00451.t01 | ERF | chr8.gff3_PROT_VIT_08s0007g06410.t01 | FAR1 |
| g19104.t1 | ERF | Vitvi13g00080.t01 | ERF | chr8.gff3_PROT_VIT_08s0040g00200.t01 | FAR1 |
| g19105.t1 | ERF | Vitvi13g00116.t01 | ERF | chr9.gff3_PROT_VIT_09s0002g07400.t01 | FAR1 |
| g19438.t1 | ERF | Vitvi14g00564.t01 | ERF | chr9.gff3_PROT_VIT_09s0054g01830.t01 | FAR1 |
| g20501.t1 | ERF | Vitvi14g01067.t01 | ERF | chrUn.gff3_PROT_VIT_00s0174g00090.t01 | FAR1 |
| g20533.t1 | ERF | Vitvi14g01441.t01 | ERF | chrUn.gff3_PROT_VIT_00s0174g00120.t01 | FAR1 |
| g22239.t1 | ERF | Vitvi14g01458.t01 | ERF | chrUn.gff3_PROT_VIT_00s0174g00130.t01 | FAR1 |
| g22462.t1 | ERF | Vitvi14g02001.t01 | ERF | chrUn.gff3_PROT_VIT_00s0174g00140.t01 | FAR1 |
| g22771.t1 | ERF | Vitvi14g02049.t01 | ERF | chrUn.gff3_PROT_VIT_00s0194g00200.t01 | FAR1 |
| g23495.t1 | ERF | Vitvi15g00601.t01 | ERF | chrUn.gff3_PROT_VIT_00s0269g00010.t01 | FAR1 |
| g249.t1 | ERF | Vitvi15g00826.t01 | ERF | chrUn.gff3_PROT_VIT_00s0269g00040.t01 | FAR1 |
| g250.t1 | ERF | Vitvi15g00947.t01 | ERF | chr1.gff3_PROT_VIT_01s0011g03110.t01 | G2-like |
| g25281.t1 | ERF | Vitvi15g01021.t01 | ERF | chr1.gff3_PROT_VIT_01s0011g04220.t01 | G2-like |
| g26173.t2 | ERF | Vitvi15g01202.t01 | ERF | chr1.gff3_PROT_VIT_01s0026g02550.t01 | G2-like |
| g26530.t1 | ERF | Vitvi15g01203.t01 | ERF | chr1.gff3_PROT_VIT_01s0137g00050.t01 | G2-like |
| g26762.t1 | ERF | Vitvi15g01204.t01 | ERF | chr10.gff3_PROT_VIT_10s0003g01380.t01 | G2-like |
| g27334.t1 | ERF | Vitvi15g01206.t01 | ERF | chr10.gff3_PROT_VIT_10s0116g01590.t01 | G2-like |
| g27335.t1 | ERF | Vitvi16g00349.t01 | ERF | chr11.gff3_PROT_VIT_11s0016g04600.t01 | G2-like |
| g27664.t1 | ERF | Vitvi16g00350.t01 | ERF | chr12.gff3_PROT_VIT_12s0028g00980.t01 | G2-like |
| g27795.t1 | ERF | Vitvi16g00360.t01 | ERF | chr12.gff3_PROT_VIT_12s0028g02080.t01 | G2-like |
| g29239.t1 | ERF | Vitvi16g00362.t01 | ERF | chr12.gff3_PROT_VIT_12s0028g03100.t01 | G2-like |
| g29241.t1 | ERF | Vitvi16g00363.t01 | ERF | chr13.gff3_PROT_VIT_13s0067g01500.t01 | G2-like |
| g29850.t2 | ERF | Vitvi16g00370.t01 | ERF | chr13.gff3_PROT_VIT_13s0156g00370.t01 | G2-like |
| g30040.t1 | ERF | Vitvi16g00380.t01 | ERF | chr13.gff3_PROT_VIT_13s0156g00390.t01 | G2-like |
| g32108.t1 | ERF | Vitvi16g00941.t01 | ERF | chr14.gff3_PROT_VIT_14s0036g01380.t01 | G2-like |
| g32111.t1 | ERF | Vitvi16g00942.t01 | ERF | chr16.gff3_PROT_VIT_16s0039g01900.t01 | G2-like |
| g32255.t1 | ERF | Vitvi16g01201.t01 | ERF | chr16.gff3_PROT_VIT_16s0100g00430.t01 | G2-like |
| g32973.t1 | ERF | Vitvi16g01423.t01 | ERF | chr18.gff3_PROT_VIT_18s0001g11390.t01 | G2-like |
| g33333.t1 | ERF | Vitvi16g01424.t01 | ERF | chr19.gff3_PROT_VIT_19s0085g00890.t01 | G2-like |
| g33385.t1 | ERF | Vitvi16g01429.t01 | ERF | chr2.gff3_PROT_VIT_02s0012g01940.t01 | G2-like |
| g33989.t1 | ERF | Vitvi16g01430.t01 | ERF | chr2.gff3_PROT_VIT_02s0033g00300.t01 | G2-like |
| g3458.t1 | ERF | Vitvi16g01432.t01 | ERF | chr3.gff3_PROT_VIT_03s0017g02280.t01 | G2-like |
| g35196.t1 | ERF | Vitvi16g01434.t01 | ERF | chr3.gff3_PROT_VIT_03s0063g01060.t01 | G2-like |
| g35201.t1 | ERF | Vitvi16g01435.t01 | ERF | chr4.gff3_PROT_VIT_04s0043g00340.t01 | G2-like |
| g35575.t1 | ERF | Vitvi16g01437.t01 | ERF | chr5.gff3_PROT_VIT_05s0029g00060.t01 | G2-like |
| g3575.t1 | ERF | Vitvi16g01438.t01 | ERF | chr6.gff3_PROT_VIT_06s0004g00150.t01 | G2-like |
| g36103.t1 | ERF | Vitvi16g01442.t01 | ERF | chr6.gff3_PROT_VIT_06s0004g05120.t01 | G2-like |
| g36104.t1 | ERF | Vitvi16g01443.t01 | ERF | chr6.gff3_PROT_VIT_06s0004g07000.t01 | G2-like |
| g36825.t1 | ERF | Vitvi16g01444.t01 | ERF | chr7.gff3_PROT_VIT_07s0005g04120.t01 | G2-like |
| g4444.t1 | ERF | Vitvi17g00025.t01 | ERF | chr7.gff3_PROT_VIT_07s0197g00060.t01 | G2-like |
| g11558.t1 | FAR1 | Vitvi17g00329.t01 | ERF | chr8.gff3_PROT_VIT_08s0007g06180.t01 | G2-like |
| g11560.t1 | FAR1 | Vitvi17g00406.t01 | ERF | chr8.gff3_PROT_VIT_08s0007g06310.t01 | G2-like |
| g14132.t1 | FAR1 | Vitvi17g00769.t01 | ERF | chr8.gff3_PROT_VIT_08s0007g07580.t01 | G2-like |
| g14330.t1 | FAR1 | Vitvi18g00295.t01 | ERF | chr8.gff3_PROT_VIT_08s0040g00100.t01 | G2-like |
| g14332.t1 | FAR1 | Vitvi18g00381.t01 | ERF | chr8.gff3_PROT_VIT_08s0040g00900.t01 | G2-like |
| g14333.t1 | FAR1 | Vitvi18g01029.t01 | ERF | chr8.gff3_PROT_VIT_08s0058g00250.t01 | G2-like |
| g14334.t1 | FAR1 | Vitvi18g01617.t01 | ERF | chr8.gff3_PROT_VIT_08s0105g00370.t01 | G2-like |
| g16619.t1 | FAR1 | Vitvi18g02191.t01 | ERF | chr9.gff3_PROT_VIT_09s0054g01620.t01 | G2-like |
| g16624.t1 | FAR1 | Vitvi18g02240.t01 | ERF | chr9.gff3_PROT_VIT_09s0070g00410.t01 | G2-like |
| g16626.t1 | FAR1 | Vitvi18g02241.t01 | ERF | chrUn.gff3_PROT_VIT_00s0475g00030.t01 | G2-like |
| g16626.t2 | FAR1 | Vitvi18g02398.t01 | ERF | chrUn.gff3_PROT_VIT_00s0475g00040.t01 | G2-like |
| g17654.t1 | FAR1 | Vitvi18g02437.t01 | ERF | chrUn.gff3_PROT_VIT_00s1624g00010.t01 | G2-like |
| g18683.t1 | FAR1 | Vitvi19g00260.t01 | ERF | chr11.gff3_PROT_VIT_11s0016g02210.t01 | GATA |
| g22069.t1 | FAR1 | Vitvi19g00558.t01 | ERF | chr12.gff3_PROT_VIT_12s0121g00120.t01 | GATA |
| g22094.t1 | FAR1 | Vitvi19g01784.t01 | ERF | chr13.gff3_PROT_VIT_13s0019g04390.t01 | GATA |
| g22517.t1 | FAR1 | Vitvi00g02057.t01 | FAR1 | chr14.gff3_PROT_VIT_14s0060g01520.t01 | GATA |
| g22801.t1 | FAR1 | Vitvi01g00116.t01 | FAR1 | chr14.gff3_PROT_VIT_14s0066g00150.t01 | GATA |
| g23073.t1 | FAR1 | Vitvi02g01591.t01 | FAR1 | chr15.gff3_PROT_VIT_15s0021g02510.t01 | GATA |
| g23165.t1 | FAR1 | Vitvi03g01239.t01 | FAR1 | chr18.gff3_PROT_VIT_18s0001g07720.t01 | GATA |
| g25893.t1 | FAR1 | Vitvi03g01243.t01 | FAR1 | chr18.gff3_PROT_VIT_18s0001g07730.t01 | GATA |
| g25893.t2 | FAR1 | Vitvi03g01244.t01 | FAR1 | chr3.gff3_PROT_VIT_03s0038g00490.t01 | GATA |
| g26322.t1 | FAR1 | Vitvi03g01307.t01 | FAR1 | chr3_random.gff3_PROT_VIT_03s0038g00480.t01 | GATA |
| g26325.t1 | FAR1 | Vitvi03g01329.t01 | FAR1 | chr4.gff3_PROT_VIT_04s0008g01290.t01 | GATA |
| g26326.t1 | FAR1 | Vitvi03g01475.t01 | FAR1 | chr4.gff3_PROT_VIT_04s0008g03270.t01 | GATA |
| g26362.t1 | FAR1 | Vitvi03g01688.t01 | FAR1 | chr4.gff3_PROT_VIT_04s0023g01840.t01 | GATA |
| g26373.t1 | FAR1 | Vitvi03g01735.t01 | FAR1 | chr4.gff3_PROT_VIT_04s0023g02880.t01 | GATA |
| g26382.t1 | FAR1 | Vitvi03g01744.t01 | FAR1 | chr5.gff3_PROT_VIT_05s0051g00450.t01 | GATA |
| g26391.t1 | FAR1 | Vitvi04g00504.t01 | FAR1 | chr5.gff3_PROT_VIT_05s0077g01450.t01 | GATA |
| g26393.t1 | FAR1 | Vitvi04g01750.t01 | FAR1 | chr6.gff3_PROT_VIT_06s0004g02740.t01 | GATA |
| g26395.t1 | FAR1 | Vitvi05g00081.t01 | FAR1 | chr8.gff3_PROT_VIT_08s0007g07550.t01 | GATA |
| g26396.t1 | FAR1 | Vitvi05g01496.t01 | FAR1 | chr9.gff3_PROT_VIT_09s0002g03750.t01 | GATA |
| g27147.t1 | FAR1 | Vitvi05g01497.t01 | FAR1 | chr9.gff3_PROT_VIT_09s0054g00440.t01 | GATA |
| g27455.t1 | FAR1 | Vitvi05g01498.t01 | FAR1 | chr12.gff3_PROT_VIT_12s0059g02690.t01 | GeBP |
| g27455.t2 | FAR1 | Vitvi05g01499.t01 | FAR1 | chr19.gff3_PROT_VIT_19s0014g05030.t01 | GeBP |
| g27746.t1 | FAR1 | Vitvi05g01506.t01 | FAR1 | chrUn.gff3_PROT_VIT_00s0868g00010.t01 | GeBP |
| g28017.t1 | FAR1 | Vitvi05g01510.t01 | FAR1 | chr1.gff3_PROT_VIT_01s0010g02270.t01 | GRAS |
| g29877.t1 | FAR1 | Vitvi05g01512.t01 | FAR1 | chr1.gff3_PROT_VIT_01s0011g05260.t01 | GRAS |
| g30877.t1 | FAR1 | Vitvi05g01873.t01 | FAR1 | chr11.gff3_PROT_VIT_11s0016g04630.t01 | GRAS |
| g32611.t1 | FAR1 | Vitvi05g02179.t01 | FAR1 | chr12.gff3_PROT_VIT_12s0057g00560.t01 | GRAS |
| g33988.t1 | FAR1 | Vitvi05g02274.t01 | FAR1 | chr12.gff3_PROT_VIT_12s0059g02770.t01 | GRAS |
| g34115.t1 | FAR1 | Vitvi06g01048.t01 | FAR1 | chr13.gff3_PROT_VIT_13s0019g01700.t01 | GRAS |
| g34115.t2 | FAR1 | Vitvi06g01874.t01 | FAR1 | chr13.gff3_PROT_VIT_13s0019g01710.t01 | GRAS |
| g34425.t1 | FAR1 | Vitvi06g01900.t01 | FAR1 | chr13.gff3_PROT_VIT_13s0019g01780.t01 | GRAS |
| g35204.t1 | FAR1 | Vitvi07g00384.t01 | FAR1 | chr13.gff3_PROT_VIT_13s0019g01790.t01 | GRAS |
| g36818.t1 | FAR1 | Vitvi07g01172.t01 | FAR1 | chr13.gff3_PROT_VIT_13s0019g01810.t01 | GRAS |
| g37011.t1 | FAR1 | Vitvi07g01196.t01 | FAR1 | chr13_random.gff3_PROT_VIT_13s0019g01220.t01 | GRAS |
| g4693.t1 | FAR1 | Vitvi07g01198.t01 | FAR1 | chr14.gff3_PROT_VIT_14s0006g00640.t01 | GRAS |
| g9324.t1 | FAR1 | Vitvi07g01199.t01 | FAR1 | chr14.gff3_PROT_VIT_14s0068g01610.t01 | GRAS |
| g1181.t1 | G2-like | Vitvi07g02480.t01 | FAR1 | chr15.gff3_PROT_VIT_15s0048g00270.t01 | GRAS |
| g11889.t1 | G2-like | Vitvi08g00891.t01 | FAR1 | chr16.gff3_PROT_VIT_16s0050g00950.t01 | GRAS |
| g11891.t1 | G2-like | Vitvi08g01733.t01 | FAR1 | chr17.gff3_PROT_VIT_17s0000g10300.t01 | GRAS |
| g12976.t1 | G2-like | Vitvi08g02078.t01 | FAR1 | chr18.gff3_PROT_VIT_18s0001g03310.t01 | GRAS |
| g13427.t1 | G2-like | Vitvi09g00649.t01 | FAR1 | chr18.gff3_PROT_VIT_18s0001g15270.t01 | GRAS |
| g13656.t1 | G2-like | Vitvi09g00650.t01 | FAR1 | chr18.gff3_PROT_VIT_18s0076g00200.t01 | GRAS |
| g13656.t2 | G2-like | Vitvi09g01480.t01 | FAR1 | chr19.gff3_PROT_VIT_19s0014g04940.t01 | GRAS |
| g1383.t1 | G2-like | Vitvi09g01677.t01 | FAR1 | chr19.gff3_PROT_VIT_19s0015g01930.t01 | GRAS |
| g14062.t1 | G2-like | Vitvi10g00230.t01 | FAR1 | chr19.gff3_PROT_VIT_19s0085g00540.t01 | GRAS |
| g14139.t1 | G2-like | Vitvi11g01669.t01 | FAR1 | chr19.gff3_PROT_VIT_19s0090g01740.t01 | GRAS |
| g14139.t2 | G2-like | Vitvi11g01700.t01 | FAR1 | chr2.gff3_PROT_VIT_02s0025g04000.t01 | GRAS |
| g14736.t1 | G2-like | Vitvi12g00713.t01 | FAR1 | chr2.gff3_PROT_VIT_02s0033g00050.t01 | GRAS |
| g14736.t2 | G2-like | Vitvi12g02073.t01 | FAR1 | chr2.gff3_PROT_VIT_02s0154g00400.t01 | GRAS |
| g16052.t1 | G2-like | Vitvi12g02453.t01 | FAR1 | chr3.gff3_PROT_VIT_03s0017g01660.t01 | GRAS |
| g16356.t1 | G2-like | Vitvi13g01139.t01 | FAR1 | chr4.gff3_PROT_VIT_04s0023g01380.t01 | GRAS |
| g16356.t2 | G2-like | Vitvi13g02116.t01 | FAR1 | chr4.gff3_PROT_VIT_04s0023g01660.t01 | GRAS |
| g16499.t1 | G2-like | Vitvi13g02304.t01 | FAR1 | chr4.gff3_PROT_VIT_04s0044g00620.t01 | GRAS |
| g17632.t1 | G2-like | Vitvi13g02479.t01 | FAR1 | chr4.gff3_PROT_VIT_04s0044g01370.t01 | GRAS |
| g17643.t1 | G2-like | Vitvi14g00948.t01 | FAR1 | chr5.gff3_PROT_VIT_05s0077g01120.t01 | GRAS |
| g17643.t2 | G2-like | Vitvi14g01432.t01 | FAR1 | chr6.gff3_PROT_VIT_06s0004g04950.t01 | GRAS |
| g17761.t1 | G2-like | Vitvi14g01622.t01 | FAR1 | chr6.gff3_PROT_VIT_06s0004g04960.t01 | GRAS |
| g18569.t1 | G2-like | Vitvi14g02030.t01 | FAR1 | chr6.gff3_PROT_VIT_06s0004g04970.t01 | GRAS |
| g18905.t1 | G2-like | Vitvi14g02715.t01 | FAR1 | chr6.gff3_PROT_VIT_06s0004g04980.t01 | GRAS |
| g1969.t1 | G2-like | Vitvi14g02853.t01 | FAR1 | chr6.gff3_PROT_VIT_06s0004g04990.t01 | GRAS |
| g1969.t2 | G2-like | Vitvi15g01428.t01 | FAR1 | chr6.gff3_PROT_VIT_06s0009g02390.t01 | GRAS |
| g1970.t1 | G2-like | Vitvi17g01317.t01 | FAR1 | chr7.gff3_PROT_VIT_07s0005g01500.t01 | GRAS |
| g1970.t2 | G2-like | Vitvi18g00039.t01 | FAR1 | chr7.gff3_PROT_VIT_07s0005g03700.t01 | GRAS |
| g20268.t1 | G2-like | Vitvi18g01884.t01 | FAR1 | chr7.gff3_PROT_VIT_07s0129g00030.t01 | GRAS |
| g20268.t2 | G2-like | Vitvi18g02193.t01 | FAR1 | chr7.gff3_PROT_VIT_07s0129g00340.t01 | GRAS |
| g21488.t1 | G2-like | Vitvi18g02891.t01 | FAR1 | chr8.gff3_PROT_VIT_08s0007g00510.t01 | GRAS |
| g21489.t1 | G2-like | Vitvi18g03049.t01 | FAR1 | chr8.gff3_PROT_VIT_08s0056g00050.t01 | GRAS |
| g22620.t1 | G2-like | Vitvi18g03185.t01 | FAR1 | chr8.gff3_PROT_VIT_08s0058g00310.t01 | GRAS |
| g2276.t1 | G2-like | Vitvi01g00249.t01 | G2-like | chr9.gff3_PROT_VIT_09s0002g01190.t01 | GRAS |
| g23123.t1 | G2-like | Vitvi01g00578.t01 | G2-like | chrUn.gff3_PROT_VIT_00s0226g00070.t01 | GRAS |
| g24216.t1 | G2-like | Vitvi01g01024.t01 | G2-like | chrUn.gff3_PROT_VIT_00s0463g00020.t01 | GRAS |
| g26502.t1 | G2-like | Vitvi02g00757.t01 | G2-like | chr11.gff3_PROT_VIT_11s0016g01250.t01 | GRF |
| g27277.t1 | G2-like | Vitvi02g01004.t01 | G2-like | chr16.gff3_PROT_VIT_16s0039g01450.t01 | GRF |
| g30384.t1 | G2-like | Vitvi03g00454.t01 | G2-like | chr16.gff3_PROT_VIT_16s0098g01080.t01 | GRF |
| g30384.t2 | G2-like | Vitvi03g01303.t01 | G2-like | chr18.gff3_PROT_VIT_18s0001g08650.t01 | GRF |
| g3097.t1 | G2-like | Vitvi04g00965.t01 | G2-like | chr2.gff3_PROT_VIT_02s0025g02680.t01 | GRF |
| g3097.t2 | G2-like | Vitvi04g02230.t01 | G2-like | chr2.gff3_PROT_VIT_02s0025g04910.t01 | GRF |
| g31.t1 | G2-like | Vitvi05g01066.t01 | G2-like | chr8.gff3_PROT_VIT_08s0007g03760.t01 | GRF |
| g31466.t1 | G2-like | Vitvi06g00024.t01 | G2-like | chr9.gff3_PROT_VIT_09s0002g01350.t01 | GRF |
| g3168.t1 | G2-like | Vitvi06g00505.t01 | G2-like | chrUn.gff3_PROT_VIT_00s0494g00010.t01 | GRF |
| g31712.t1 | G2-like | Vitvi06g00686.t01 | G2-like | chr10.gff3_PROT_VIT_10s0003g00290.t01 | HB-other |
| g32460.t1 | G2-like | Vitvi07g00666.t01 | G2-like | chr12.gff3_PROT_VIT_12s0028g03080.t01 | HB-other |
| g32944.t1 | G2-like | Vitvi07g01325.t01 | G2-like | chr13.gff3_PROT_VIT_13s0067g01580.t01 | HB-other |
| g33599.t1 | G2-like | Vitvi08g00654.t01 | G2-like | chr14.gff3_PROT_VIT_14s0030g02130.t01 | HB-other |
| g33645.t1 | G2-like | Vitvi08g00736.t01 | G2-like | chr17.gff3_PROT_VIT_17s0000g08980.t01 | HB-other |
| g33645.t2 | G2-like | Vitvi08g00955.t01 | G2-like | chr19.gff3_PROT_VIT_19s0014g01880.t01 | HB-other |
| g33645.t3 | G2-like | Vitvi08g01712.t01 | G2-like | chr19.gff3_PROT_VIT_19s0085g00350.t01 | HB-other |
| g34137.t1 | G2-like | Vitvi08g01723.t01 | G2-like | chr4.gff3_PROT_VIT_04s0008g05190.t01 | HB-other |
| g35376.t1 | G2-like | Vitvi08g01834.t01 | G2-like | chr6.gff3_PROT_VIT_06s0009g03530.t01 | HB-other |
| g35376.t2 | G2-like | Vitvi08g02099.t01 | G2-like | chr7.gff3_PROT_VIT_07s0104g01580.t01 | HB-other |
| g36137.t1 | G2-like | Vitvi09g01008.t01 | G2-like | chr9.gff3_PROT_VIT_09s0018g02100.t01 | HB-other |
| g5303.t1 | G2-like | Vitvi09g01459.t01 | G2-like | chr3.gff3_PROT_VIT_03s0091g00290.t01 | HB-PHD |
| g5754.t1 | G2-like | Vitvi10g00301.t01 | G2-like | chr11.gff3_PROT_VIT_11s0103g00650.t01 | HB-PHD |
| g5754.t2 | G2-like | Vitvi10g00302.t01 | G2-like | chr14.gff3_PROT_VIT_14s0066g01440.t01 | HD-ZIP |
| g6541.t1 | G2-like | Vitvi10g01637.t01 | G2-like | chr1.gff3_PROT_VIT_01s0026g01950.t01 | HD-ZIP |
| g6541.t2 | G2-like | Vitvi10g01793.t01 | G2-like | chr18.gff3_PROT_VIT_18s0001g06430.t01 | HD-ZIP |
| g6541.t3 | G2-like | Vitvi10g02406.t01 | G2-like | chr7.gff3_PROT_VIT_07s0191g00180.t01 | HD-ZIP |
| g712.t1 | G2-like | Vitvi11g00405.t01 | G2-like | chr15.gff3_PROT_VIT_15s0021g01880.t01 | HD-ZIP |
| g8276.t1 | G2-like | Vitvi12g00177.t01 | G2-like | chr13.gff3_PROT_VIT_13s0156g00260.t01 | HD-ZIP |
| g9613.t1 | G2-like | Vitvi12g00260.t01 | G2-like | chr8.gff3_PROT_VIT_08s0007g06670.t01 | HD-ZIP |
| g9614.t1 | G2-like | Vitvi12g02204.t01 | G2-like | chr14.gff3_PROT_VIT_14s0108g00390.t01 | HD-ZIP |
| g12024.t1 | GATA | Vitvi12g02510.t01 | G2-like | chr17.gff3_PROT_VIT_17s0000g05630.t01 | HD-ZIP |
| g12452.t1 | GATA | Vitvi13g00088.t01 | G2-like | chrUn.gff3_PROT_VIT_00s0299g00100.t01 | HD-ZIP |
| g14573.t1 | GATA | Vitvi13g02566.t01 | G2-like | chr1.gff3_PROT_VIT_01s0026g01550.t01 | HD-ZIP |
| g14574.t1 | GATA | Vitvi13g02568.t01 | G2-like | chrUn.gff3_PROT_VIT_00s0732g00010.t01 | HD-ZIP |
| g15171.t1 | GATA | Vitvi14g00736.t01 | G2-like | chr15.gff3_PROT_VIT_15s0048g02870.t01 | HD-ZIP |
| g15811.t1 | GATA | Vitvi14g01333.t01 | G2-like | chr16.gff3_PROT_VIT_16s0098g01170.t01 | HD-ZIP |
| g21682.t1 | GATA | Vitvi14g02915.t01 | G2-like | chr18.gff3_PROT_VIT_18s0001g08410.t01 | HD-ZIP |
| g22177.t1 | GATA | Vitvi16g00121.t01 | G2-like | chr8.gff3_PROT_VIT_08s0007g04200.t01 | HD-ZIP |
| g22178.t1 | GATA | Vitvi16g00773.t01 | G2-like | chr1.gff3_PROT_VIT_01s0011g04870.t01 | HD-ZIP |
| g22178.t2 | GATA | Vitvi16g00947.t01 | G2-like | chr4.gff3_PROT_VIT_04s0023g01330.t01 | HD-ZIP |
| g24151.t1 | GATA | Vitvi18g00869.t01 | G2-like | chr19.gff3_PROT_VIT_19s0014g02110.t01 | HD-ZIP |
| g25567.t1 | GATA | Vitvi19g01742.t01 | G2-like | chr10.gff3_PROT_VIT_10s0003g00380.t01 | HD-ZIP |
| g25682.t1 | GATA | Vitvi03g00037.t01 | GATA | chr2.gff3_PROT_VIT_02s0025g02590.t01 | HD-ZIP |
| g27459.t1 | GATA | Vitvi03g01002.t01 | GATA | chr9.gff3_PROT_VIT_09s0002g04340.t01 | HD-ZIP |
| g27579.t1 | GATA | Vitvi03g01766.t01 | GATA | chr12.gff3_PROT_VIT_12s0059g02310.t01 | HD-ZIP |
| g31803.t1 | GATA | Vitvi04g00111.t01 | GATA | chr10.gff3_PROT_VIT_10s0116g00680.t01 | HD-ZIP |
| g3360.t1 | GATA | Vitvi04g00289.t01 | GATA | chr15.gff3_PROT_VIT_15s0048g02000.t01 | HD-ZIP |
| g33737.t1 | GATA | Vitvi04g01299.t01 | GATA | chr17.gff3_PROT_VIT_17s0053g00780.t01 | HD-ZIP |
| g3563.t1 | GATA | Vitvi04g01410.t01 | GATA | chr16.gff3_PROT_VIT_16s0100g00670.t01 | HD-ZIP |
| g36033.t1 | GATA | Vitvi05g00077.t01 | GATA | chr9.gff3_PROT_VIT_09s0002g03740.t01 | HD-ZIP |
| g36309.t1 | GATA | Vitvi05g00938.t01 | GATA | chr10.gff3_PROT_VIT_10s0003g04670.t01 | HD-ZIP |
| g36412.t1 | GATA | Vitvi06g00271.t01 | GATA | chr4.gff3_PROT_VIT_04s0008g03250.t01 | HD-ZIP |
| g36412.t2 | GATA | Vitvi06g01610.t01 | GATA | chr2.gff3_PROT_VIT_02s0012g02030.t01 | HD-ZIP |
| g36416.t1 | GATA | Vitvi07g02214.t01 | GATA | chr13.gff3_PROT_VIT_13s0019g04320.t01 | HD-ZIP |
| g37016.t1 | GATA | Vitvi08g01831.t01 | GATA | chr6.gff3_PROT_VIT_06s0004g02800.t01 | HD-ZIP |
| g38279.t1 | GATA | Vitvi09g00311.t01 | GATA | chr4.gff3_PROT_VIT_04s0079g00480.t01 | HD-ZIP |
| g9001.t1 | GATA | Vitvi09g01352.t01 | GATA | chr11.gff3_PROT_VIT_11s0016g02440.t01 | HRT-like |
| g9476.t1 | GATA | Vitvi11g00180.t01 | GATA | chr1.gff3_PROT_VIT_01s0011g05970.t01 | HSF |
| g16178.t1 | GeBP | Vitvi12g01002.t01 | GATA | chr10.gff3_PROT_VIT_10s0003g01770.t01 | HSF |
| g1925.t1 | GeBP | Vitvi13g00614.t01 | GATA | chr10.gff3_PROT_VIT_10s0597g00050.t01 | HSF |
| g25697.t1 | GeBP | Vitvi14g00123.t01 | GATA | chr11.gff3_PROT_VIT_11s0016g02010.t01 | HSF |
| g28578.t1 | GeBP | Vitvi14g02998.t01 | GATA | chr11.gff3_PROT_VIT_11s0016g03940.t01 | HSF |
| g30874.t1 | GeBP | Vitvi15g00636.t01 | GATA | chr12.gff3_PROT_VIT_12s0028g01410.t01 | HSF |
| g30874.t2 | GeBP | Vitvi18g00537.t01 | GATA | chr16.gff3_PROT_VIT_16s0039g01840.t01 | HSF |
| g4794.t1 | GeBP | Vitvi18g00538.t01 | GATA | chr16.gff3_PROT_VIT_16s0100g00720.t01 | HSF |
| g1048.t1 | GRAS | Vitvi10g02200.t01 | GeBP | chr18.gff3_PROT_VIT_18s0001g10380.t01 | HSF |
| g11217.t1 | GRAS | Vitvi12g00561.t01 | GeBP | chr2.gff3_PROT_VIT_02s0012g01810.t01 | HSF |
| g12179.t1 | GRAS | Vitvi14g00879.t01 | GeBP | chr2.gff3_PROT_VIT_02s0025g04170.t01 | HSF |
| g1397.t1 | GRAS | Vitvi19g01979.t01 | GeBP | chr4.gff3_PROT_VIT_04s0008g01110.t01 | HSF |
| g1398.t1 | GRAS | Vitvi01g00446.t01 | GRAS | chr5.gff3_PROT_VIT_05s0020g04090.t01 | HSF |
| g1398.t2 | GRAS | Vitvi01g01509.t01 | GRAS | chr5.gff3_PROT_VIT_05s0029g00340.t01 | HSF |
| g1399.t1 | GRAS | Vitvi02g00370.t01 | GRAS | chr6.gff3_PROT_VIT_06s0009g02730.t01 | HSF |
| g1400.t1 | GRAS | Vitvi02g00536.t01 | GRAS | chr7.gff3_PROT_VIT_07s0031g00670.t01 | HSF |
| g1401.t1 | GRAS | Vitvi02g00974.t01 | GRAS | chr8.gff3_PROT_VIT_08s0007g03900.t01 | HSF |
| g15906.t1 | GRAS | Vitvi03g01226.t01 | GRAS | chr8.gff3_PROT_VIT_08s0007g08750.t01 | HSF |
| g16154.t1 | GRAS | Vitvi04g01247.t01 | GRAS | chrUn.gff3_PROT_VIT_00s0179g00150.t01 | HSF |
| g16154.t2 | GRAS | Vitvi04g01281.t01 | GRAS | chr1.gff3_PROT_VIT_01s0011g03530.t01 | LBD |
| g16169.t1 | GRAS | Vitvi04g01622.t01 | GRAS | chr1.gff3_PROT_VIT_01s0011g03540.t01 | LBD |
| g16381.t1 | GRAS | Vitvi04g01696.t01 | GRAS | chr13.gff3_PROT_VIT_13s0019g02060.t01 | LBD |
| g16381.t2 | GRAS | Vitvi05g00110.t01 | GRAS | chr13.gff3_PROT_VIT_13s0019g03700.t01 | LBD |
| g1676.t1 | GRAS | Vitvi05g01554.t01 | GRAS | chr13.gff3_PROT_VIT_13s0019g03710.t01 | LBD |
| g1677.t1 | GRAS | Vitvi06g00489.t01 | GRAS | chr13.gff3_PROT_VIT_13s0019g03720.t01 | LBD |
| g1678.t1 | GRAS | Vitvi06g00490.t01 | GRAS | chr13.gff3_PROT_VIT_13s0019g03750.t01 | LBD |
| g1679.t1 | GRAS | Vitvi06g00491.t01 | GRAS | chr13.gff3_PROT_VIT_13s0019g03760.t01 | LBD |
| g1680.t1 | GRAS | Vitvi06g00492.t01 | GRAS | chr13.gff3_PROT_VIT_13s0019g03780.t01 | LBD |
| g1681.t1 | GRAS | Vitvi06g01133.t01 | GRAS | chr13.gff3_PROT_VIT_13s0019g03800.t01 | LBD |
| g18559.t1 | GRAS | Vitvi06g01569.t01 | GRAS | chr13.gff3_PROT_VIT_13s0019g03810.t01 | LBD |
| g18635.t1 | GRAS | Vitvi07g00418.t01 | GRAS | chr13.gff3_PROT_VIT_13s0019g03820.t01 | LBD |
| g19332.t1 | GRAS | Vitvi07g00627.t01 | GRAS | chr13.gff3_PROT_VIT_13s0019g03830.t01 | LBD |
| g2000.t1 | GRAS | Vitvi07g01612.t01 | GRAS | chr13.gff3_PROT_VIT_13s0019g03840.t01 | LBD |
| g20201.t1 | GRAS | Vitvi07g02073.t01 | GRAS | chr13.gff3_PROT_VIT_13s0067g01450.t01 | LBD |
| g20626.t1 | GRAS | Vitvi08g00007.t01 | GRAS | chr13.gff3_PROT_VIT_13s0067g01880.t01 | LBD |
| g20653.t1 | GRAS | Vitvi08g00746.t01 | GRAS | chr13.gff3_PROT_VIT_13s0067g02380.t01 | LBD |
| g21329.t1 | GRAS | Vitvi08g00751.t01 | GRAS | chr14.gff3_PROT_VIT_14s0006g02950.t01 | LBD |
| g21331.t1 | GRAS | Vitvi08g01214.t01 | GRAS | chr14.gff3_PROT_VIT_14s0066g00680.t01 | LBD |
| g22489.t1 | GRAS | Vitvi08g01969.t01 | GRAS | chr14.gff3_PROT_VIT_14s0066g02460.t01 | LBD |
| g2280.t1 | GRAS | Vitvi09g01487.t01 | GRAS | chr15.gff3_PROT_VIT_15s0046g00230.t01 | LBD |
| g23640.t1 | GRAS | Vitvi10g00271.t01 | GRAS | chr15.gff3_PROT_VIT_15s0046g00240.t01 | LBD |
| g23848.t1 | GRAS | Vitvi11g00409.t01 | GRAS | chr15.gff3_PROT_VIT_15s0048g00830.t01 | LBD |
| g23848.t2 | GRAS | Vitvi12g00571.t01 | GRAS | chr15.gff3_PROT_VIT_15s0048g00840.t01 | LBD |
| g24229.t1 | GRAS | Vitvi12g00665.t01 | GRAS | chr16.gff3_PROT_VIT_16s0022g02050.t01 | LBD |
| g24486.t1 | GRAS | Vitvi13g00311.t01 | GRAS | chr16.gff3_PROT_VIT_16s0100g00440.t01 | LBD |
| g24862.t1 | GRAS | Vitvi13g00312.t01 | GRAS | chr17.gff3_PROT_VIT_17s0000g03720.t01 | LBD |
| g26562.t1 | GRAS | Vitvi13g00313.t01 | GRAS | chr17.gff3_PROT_VIT_17s0000g05490.t01 | LBD |
| g28677.t1 | GRAS | Vitvi13g00314.t01 | GRAS | chr17.gff3_PROT_VIT_17s0000g09010.t01 | LBD |
| g29988.t1 | GRAS | Vitvi13g01556.t01 | GRAS | chr18.gff3_PROT_VIT_18s0001g09250.t01 | LBD |
| g30321.t1 | GRAS | Vitvi13g01865.t01 | GRAS | chr19.gff3_PROT_VIT_19s0027g01120.t01 | LBD |
| g30433.t1 | GRAS | Vitvi14g00841.t01 | GRAS | chr3.gff3_PROT_VIT_03s0091g00670.t01 | LBD |
| g30555.t1 | GRAS | Vitvi14g01348.t01 | GRAS | chr6.gff3_PROT_VIT_06s0004g03330.t01 | LBD |
| g31098.t1 | GRAS | Vitvi14g01510.t01 | GRAS | chr6.gff3_PROT_VIT_06s0004g03350.t01 | LBD |
| g31594.t1 | GRAS | Vitvi15g00680.t01 | GRAS | chr6.gff3_PROT_VIT_06s0004g07200.t01 | LBD |
| g31719.t1 | GRAS | Vitvi16g01086.t01 | GRAS | chr6.gff3_PROT_VIT_06s0004g07790.t01 | LBD |
| g31722.t1 | GRAS | Vitvi17g01040.t01 | GRAS | chr7.gff3_PROT_VIT_07s0005g03020.t01 | LBD |
| g31723.t1 | GRAS | Vitvi18g00300.t01 | GRAS | chr7.gff3_PROT_VIT_07s0005g03030.t01 | LBD |
| g33577.t1 | GRAS | Vitvi18g01210.t01 | GRAS | chr7.gff3_PROT_VIT_07s0129g00330.t01 | LBD |
| g34486.t1 | GRAS | Vitvi18g01322.t01 | GRAS | chr7.gff3_PROT_VIT_07s0197g00020.t01 | LBD |
| g34656.t1 | GRAS | Vitvi18g01651.t01 | GRAS | chr7.gff3_PROT_VIT_07s0197g00030.t01 | LBD |
| g34685.t1 | GRAS | Vitvi19g00392.t01 | GRAS | chr7.gff3_PROT_VIT_07s0197g00040.t01 | LBD |
| g36505.t1 | GRAS | Vitvi19g00619.t01 | GRAS | chr8.gff3_PROT_VIT_08s0056g01650.t01 | LBD |
| g36966.t1 | GRAS | Vitvi19g00932.t01 | GRAS | chrUn.gff3_PROT_VIT_00s0125g00290.t01 | LBD |
| g37652.t1 | GRAS | Vitvi19g01706.t01 | GRAS | chrUn.gff3_PROT_VIT_00s0215g00010.t01 | LBD |
| g37878.t1 | GRAS | Vitvi02g00239.t01 | GRF | chrUn.gff3_PROT_VIT_00s0340g00090.t01 | LBD |
| g38315.t1 | GRAS | Vitvi02g00449.t01 | GRF | chrUn.gff3_PROT_VIT_00s1247g00010.t01 | LBD |
| g4582.t1 | GRAS | Vitvi07g01551.t01 | GRF | chr17.gff3_PROT_VIT_17s0000g00150.t01 | LFY |
| g5997.t1 | GRAS | Vitvi08g01498.t01 | GRF | chr10.gff3_PROT_VIT_10s0092g00230.t01 | LSD |
| g662.t1 | GRAS | Vitvi09g00107.t01 | GRF | chr2.gff3_PROT_VIT_02s0012g00990.t01 | LSD |
| g6980.t1 | GRAS | Vitvi11g00092.t01 | GRF | chrUn.gff3_PROT_VIT_00s0181g00230.t01 | LSD |
| g7067.t1 | GRAS | Vitvi15g00815.t01 | GRF | chr1.gff3_PROT_VIT_01s0010g03900.t01 | MIKC_MADS |
| g8811.t1 | GRAS | Vitvi16g00073.t01 | GRF | chr1.gff3_PROT_VIT_01s0011g00100.t01 | MIKC_MADS |
| g8814.t1 | GRAS | Vitvi16g01354.t01 | GRF | chr1.gff3_PROT_VIT_01s0011g00110.t01 | MIKC_MADS |
| g127.t1 | GRF | Vitvi18g00623.t01 | GRF | chr1.gff3_PROT_VIT_01s0011g01560.t01 | MIKC_MADS |
| g127.t2 | GRF | Vitvi04g00463.t01 | HB-other | chr10.gff3_PROT_VIT_10s0003g02070.t01 | MIKC_MADS |
| g17406.t1 | GRF | Vitvi06g01244.t01 | HB-other | chr10.gff3_PROT_VIT_10s0042g00820.t01 | MIKC_MADS |
| g17936.t1 | GRF | Vitvi07g00267.t01 | HB-other | chr12.gff3_PROT_VIT_12s0142g00360.t01 | MIKC_MADS |
| g20683.t1 | GRF | Vitvi09g01924.t01 | HB-other | chr13.gff3_PROT_VIT_13s0158g00100.t01 | MIKC_MADS |
| g21197.t1 | GRF | Vitvi10g00497.t01 | HB-other | chr14.gff3_PROT_VIT_14s0083g01030.t01 | MIKC_MADS |
| g22296.t1 | GRF | Vitvi12g00258.t01 | HB-other | chr14.gff3_PROT_VIT_14s0083g01050.t01 | MIKC_MADS |
| g29422.t1 | GRF | Vitvi13g01895.t01 | HB-other | chr15.gff3_PROT_VIT_15s0048g01270.t01 | MIKC_MADS |
| g296.t1 | GRF | Vitvi14g00465.t01 | HB-other | chr15.gff3_PROT_VIT_15s0107g00120.t01 | MIKC_MADS |
| g29838.t1 | GRF | Vitvi17g00888.t01 | HB-other | chr16.gff3_PROT_VIT_16s0022g02330.t01 | MIKC_MADS |
| g31085.t1 | GRF | Vitvi19g00157.t01 | HB-other | chr17.gff3_PROT_VIT_17s0000g01230.t01 | MIKC_MADS |
| g12975.t1 | HB-other | Vitvi19g01688.t01 | HB-other | chr17.gff3_PROT_VIT_17s0000g04990.t01 | MIKC_MADS |
| g1479.t1 | HB-other | Vitvi03g01600.t01 | HB-PHD | chr17.gff3_PROT_VIT_17s0000g05000.t01 | MIKC_MADS |
| g15390.t1 | HB-other | Vitvi11g01140.t01 | HB-PHD | chr18.gff3_PROT_VIT_18s0001g01760.t01 | MIKC_MADS |
| g16895.t1 | HB-other | Vitvi14g01786.t01 | HD-ZIP | chr18.gff3_PROT_VIT_18s0001g07460.t01 | MIKC_MADS |
| g25304.t1 | HB-other | Vitvi01g00958.t01 | HD-ZIP | chr18.gff3_PROT_VIT_18s0001g07900.t01 | MIKC_MADS |
| g26895.t1 | HB-other | Vitvi18g00448.t01 | HD-ZIP | chr18.gff3_PROT_VIT_18s0001g09540.t01 | MIKC_MADS |
| g28414.t1 | HB-other | Vitvi07g01488.t01 | HD-ZIP | chr18.gff3_PROT_VIT_18s0001g13460.t01 | MIKC_MADS |
| g28510.t1 | HB-other | Vitvi15g00579.t01 | HD-ZIP | chr18.gff3_PROT_VIT_18s0041g01880.t01 | MIKC_MADS |
| g28950.t1 | HB-other | Vitvi13g01823.t01 | HD-ZIP | chr18.gff3_PROT_VIT_18s0041g02140.t01 | MIKC_MADS |
| g29223.t1 | HB-other | Vitvi08g01752.t01 | HD-ZIP | chr2.gff3_PROT_VIT_02s0025g02350.t01 | MIKC_MADS |
| g36242.t1 | HB-other | Vitvi14g01922.t01 | HD-ZIP | chr2.gff3_PROT_VIT_02s0025g04650.t01 | MIKC_MADS |
| g18244.t1 | HB-PHD | Vitvi17g00540.t01 | HD-ZIP | chr4.gff3_PROT_VIT_04s0023g02820.t01 | MIKC_MADS |
| g21397.t1 | HB-PHD | Vitvi02g01717.t01 | HD-ZIP | chr7.gff3_PROT_VIT_07s0031g01140.t01 | MIKC_MADS |
| g24028.t1 | HD-ZIP | Vitvi01g00918.t01 | HD-ZIP | chr8.gff3_PROT_VIT_08s0007g08790.t01 | MIKC_MADS |
| g21497.t1 | HD-ZIP | Vitvi07g03056.t01 | HD-ZIP | chrUn.gff3_PROT_VIT_00s0211g00180.t01 | MIKC_MADS |
| g23076.t1 | HD-ZIP | Vitvi15g00912.t01 | HD-ZIP | chrUn.gff3_PROT_VIT_00s0313g00070.t01 | MIKC_MADS |
| g16970.t1 | HD-ZIP | Vitvi16g01362.t01 | HD-ZIP | chr1.gff3_PROT_VIT_01s0010g01500.t01 | M-type_MADS |
| g17678.t1 | HD-ZIP | Vitvi18g00603.t01 | HD-ZIP | chr1.gff3_PROT_VIT_01s0010g01530.t01 | M-type_MADS |
| g18850.t1 | HD-ZIP | Vitvi08g01543.t01 | HD-ZIP | chr10.gff3_PROT_VIT_10s0003g03970.t01 | M-type_MADS |
| g195.t1 | HD-ZIP | Vitvi01g00412.t01 | HD-ZIP | chr14.gff3_PROT_VIT_14s0060g00300.t01 | M-type_MADS |
| g27038.t1 | HD-ZIP | Vitvi04g01244.t01 | HD-ZIP | chr14.gff3_PROT_VIT_14s0068g01800.t01 | M-type_MADS |
| g15863.t1 | HD-ZIP | Vitvi19g01855.t01 | HD-ZIP | chr15.gff3_PROT_VIT_15s0021g00560.t01 | M-type_MADS |
| g3657.t1 | HD-ZIP | Vitvi10g01773.t01 | HD-ZIP | chr15.gff3_PROT_VIT_15s0021g02220.t01 | M-type_MADS |
| g32621.t1 | HD-ZIP | Vitvi02g00228.t01 | HD-ZIP | chr15.gff3_PROT_VIT_15s0021g02250.t01 | M-type_MADS |
| g20704.t2 | HD-ZIP | Vitvi12g00253.t01 | HD-ZIP | chr15.gff3_PROT_VIT_15s0024g01860.t01 | M-type_MADS |
| g26714.t1 | HD-ZIP | Vitvi09g00363.t01 | HD-ZIP | chr15.gff3_PROT_VIT_15s0024g02000.t01 | M-type_MADS |
| g17915.t1 | HD-ZIP | Vitvi12g00522.t01 | HD-ZIP | chr15.gff3_PROT_VIT_15s0048g01240.t01 | M-type_MADS |
| g17456.t2 | HD-ZIP | Vitvi10g00033.t01 | HD-ZIP | chr16.gff3_PROT_VIT_16s0022g02400.t01 | M-type_MADS |
| g17456.t1 | HD-ZIP | Vitvi15g00839.t01 | HD-ZIP | chr17.gff3_PROT_VIT_17s0000g06340.t01 | M-type_MADS |
| g20704.t1 | HD-ZIP | Vitvi17g01232.t01 | HD-ZIP | chr18.gff3_PROT_VIT_18s0001g04810.t01 | M-type_MADS |
| g24523.t1 | HD-ZIP | Vitvi16g00974.t01 | HD-ZIP | chr19.gff3_PROT_VIT_19s0014g00310.t01 | M-type_MADS |
| g29412.t1 | HD-ZIP | Vitvi09g00310.t01 | HD-ZIP | chr3.gff3_PROT_VIT_03s0017g00360.t01 | M-type_MADS |
| g15369.t1 | HD-ZIP | Vitvi10g00913.t01 | HD-ZIP | chr3.gff3_PROT_VIT_03s0017g00440.t01 | M-type_MADS |
| g21187.t1 | HD-ZIP | Vitvi04g00287.t01 | HD-ZIP | chr3.gff3_PROT_VIT_03s0017g00450.t01 | M-type_MADS |
| g29216.t1 | HD-ZIP | Vitvi02g00772.t01 | HD-ZIP | chr3.gff3_PROT_VIT_03s0088g00510.t01 | M-type_MADS |
| g34689.t1 | HD-ZIP | Vitvi13g00609.t01 | HD-ZIP | chr3.gff3_PROT_VIT_03s0088g00550.t01 | M-type_MADS |
| g22418.t1 | HD-ZIP | Vitvi06g00276.t01 | HD-ZIP | chr3.gff3_PROT_VIT_03s0088g00590.t01 | M-type_MADS |
| g23573.t3 | HD-ZIP | Vitvi04g00854.t01 | HD-ZIP | chr3.gff3_PROT_VIT_03s0088g00600.t01 | M-type_MADS |
| g23573.t1 | HD-ZIP | Vitvi11g00200.t01 | HRT-like | chr3.gff3_PROT_VIT_03s0088g00610.t01 | M-type_MADS |
| g23573.t2 | HD-ZIP | Vitvi01g00511.t01 | HSF | chr3.gff3_PROT_VIT_03s0097g00160.t01 | M-type_MADS |
| g31396.t1 | HD-ZIP | Vitvi02g00387.t01 | HSF | chr3.gff3_PROT_VIT_03s0167g00100.t01 | M-type_MADS |
| g26122.t1 | HD-ZIP | Vitvi02g00739.t01 | HSF | chr4.gff3_PROT_VIT_04s0008g01980.t01 | M-type_MADS |
| g26122.t2 | HD-ZIP | Vitvi04g00092.t01 | HSF | chr7.gff3_PROT_VIT_07s0005g06590.t01 | M-type_MADS |
| g28818.t1 | HD-ZIP | Vitvi05g00561.t01 | HSF | chr7.gff3_PROT_VIT_07s0129g00650.t01 | M-type_MADS |
| g25111.t1 | HD-ZIP | Vitvi05g01097.t01 | HSF | chrUn.gff3_PROT_VIT_00s0211g00150.t01 | M-type_MADS |
| g21681.t1 | HD-ZIP | Vitvi06g01878.t01 | HSF | chrUn.gff3_PROT_VIT_00s0386g00010.t01 | M-type_MADS |
| g22866.t1 | HD-ZIP | Vitvi07g00078.t01 | HSF | chrUn.gff3_PROT_VIT_00s0729g00010.t01 | M-type_MADS |
| g3561.t1 | HD-ZIP | Vitvi07g01749.t01 | HSF | chr1.gff3_PROT_VIT_01s0010g00410.t01 | MYB |
| g35244.t1 | HD-ZIP | Vitvi08g01513.t01 | HSF | chr1.gff3_PROT_VIT_01s0011g01190.t01 | MYB |
| g12030.t1 | HD-ZIP | Vitvi08g01931.t01 | HSF | chr1.gff3_PROT_VIT_01s0011g01200.t01 | MYB |
| g31811.t1 | HD-ZIP | Vitvi10g00469.t01 | HSF | chr1.gff3_PROT_VIT_01s0011g03730.t01 | MYB |
| g12246.t1 | HD-ZIP | Vitvi10g00635.t01 | HSF | chr1.gff3_PROT_VIT_01s0011g04760.t01 | MYB |
| g38257.t1 | HRT-like | Vitvi11g00159.t01 | HSF | chr1.gff3_PROT_VIT_01s0026g01910.t01 | MYB |
| g13999.t1 | HSF | Vitvi11g00339.t01 | HSF | chr1.gff3_PROT_VIT_01s0026g02600.t01 | MYB |
| g16403.t1 | HSF | Vitvi12g00121.t01 | HSF | chr1.gff3_PROT_VIT_01s0026g02770.t01 | MYB |
| g16542.t1 | HSF | Vitvi16g00114.t01 | HSF | chr1.gff3_PROT_VIT_01s0127g00730.t01 | MYB |
| g16767.t1 | HSF | Vitvi16g00982.t01 | HSF | chr10.gff3_PROT_VIT_10s0071g00660.t01 | MYB |
| g17422.t1 | HSF | Vitvi18g00777.t01 | HSF | chr11.gff3_PROT_VIT_11s0016g01300.t01 | MYB |
| g18097.t1 | HSF | Vitvi00g01060.t01 | LBD | chr11.gff3_PROT_VIT_11s0016g01310.t01 | MYB |
| g19599.t1 | HSF | Vitvi01g00290.t01 | LBD | chr11.gff3_PROT_VIT_11s0016g01320.t01 | MYB |
| g19599.t2 | HSF | Vitvi01g00291.t01 | LBD | chr11.gff3_PROT_VIT_11s0016g02780.t01 | MYB |
| g19805.t1 | HSF | Vitvi03g00628.t01 | LBD | chr11.gff3_PROT_VIT_11s0016g03750.t01 | MYB |
| g20475.t1 | HSF | Vitvi04g01768.t01 | LBD | chr11.gff3_PROT_VIT_11s0016g05660.t01 | MYB |
| g2199.t1 | HSF | Vitvi06g00336.t01 | LBD | chr11.gff3_PROT_VIT_11s0016g05690.t01 | MYB |
| g224.t1 | HSF | Vitvi06g00338.t01 | LBD | chr11.gff3_PROT_VIT_11s0052g01490.t01 | MYB |
| g24416.t1 | HSF | Vitvi06g00706.t01 | LBD | chr11.gff3_PROT_VIT_11s0078g00480.t01 | MYB |
| g28813.t1 | HSF | Vitvi06g00772.t01 | LBD | chr12.gff3_PROT_VIT_12s0059g00700.t01 | MYB |
| g29287.t1 | HSF | Vitvi07g00572.t01 | LBD | chr12.gff3_PROT_VIT_12s0134g00480.t01 | MYB |
| g3113.t1 | HSF | Vitvi07g00573.t01 | LBD | chr12.gff3_PROT_VIT_12s0134g00490.t01 | MYB |
| g3340.t1 | HSF | Vitvi07g01326.t01 | LBD | chr12.gff3_PROT_VIT_12s0134g00570.t01 | MYB |
| g36130.t1 | HSF | Vitvi07g01327.t01 | LBD | chr13.gff3_PROT_VIT_13s0019g03200.t01 | MYB |
| g36704.t1 | HSF | Vitvi07g01328.t01 | LBD | chr13.gff3_PROT_VIT_13s0019g03370.t01 | MYB |
| g38300.t1 | HSF | Vitvi07g01610.t01 | LBD | chr13.gff3_PROT_VIT_13s0019g04010.t01 | MYB |
| g5564.t1 | HSF | Vitvi07g02993.t01 | LBD | chr13.gff3_PROT_VIT_13s0064g00960.t01 | MYB |
| g11487.t1 | LBD | Vitvi08g00144.t01 | LBD | chr13.gff3_PROT_VIT_13s0067g01360.t01 | MYB |
| g11488.t1 | LBD | Vitvi09g00188.t01 | LBD | chr13.gff3_PROT_VIT_13s0084g00530.t01 | MYB |
| g1154.t1 | LBD | Vitvi10g01237.t01 | LBD | chr13_random.gff3_PROT_VIT_13s0067g01630.t01 | MYB |
| g11752.t1 | LBD | Vitvi11g00169.t01 | LBD | chr14.gff3_PROT_VIT_14s0006g00450.t01 | MYB |
| g14730.t2 | LBD | Vitvi12g00230.t01 | LBD | chr14.gff3_PROT_VIT_14s0006g01280.t01 | MYB |
| g14732.t1 | LBD | Vitvi13g00085.t01 | LBD | chr14.gff3_PROT_VIT_14s0006g01290.t01 | MYB |
| g14734.t1 | LBD | Vitvi13g00109.t01 | LBD | chr14.gff3_PROT_VIT_14s0006g01340.t01 | MYB |
| g14839.t1 | LBD | Vitvi13g00144.t01 | LBD | chr14.gff3_PROT_VIT_14s0006g01620.t01 | MYB |
| g14875.t1 | LBD | Vitvi13g00333.t01 | LBD | chr14.gff3_PROT_VIT_14s0060g00240.t01 | MYB |
| g16006.t1 | LBD | Vitvi13g00543.t01 | LBD | chr14.gff3_PROT_VIT_14s0066g01010.t01 | MYB |
| g16007.t1 | LBD | Vitvi13g00545.t01 | LBD | chr14.gff3_PROT_VIT_14s0066g01090.t01 | MYB |
| g1654.t1 | LBD | Vitvi13g00546.t01 | LBD | chr14.gff3_PROT_VIT_14s0066g01220.t01 | MYB |
| g17060.t1 | LBD | Vitvi13g00549.t01 | LBD | chr14.gff3_PROT_VIT_14s0066g02180.t01 | MYB |
| g17061.t1 | LBD | Vitvi13g00551.t01 | LBD | chr14.gff3_PROT_VIT_14s0083g00120.t01 | MYB |
| g17062.t1 | LBD | Vitvi13g00552.t01 | LBD | chr14.gff3_PROT_VIT_14s0083g01060.t01 | MYB |
| g17992.t1 | LBD | Vitvi13g00555.t01 | LBD | chr14.gff3_PROT_VIT_14s0108g00830.t01 | MYB |
| g18175.t1 | LBD | Vitvi13g00556.t01 | LBD | chr14.gff3_PROT_VIT_14s0108g01010.t01 | MYB |
| g18278.t1 | LBD | Vitvi13g00559.t01 | LBD | chr14.gff3_PROT_VIT_14s0108g01080.t01 | MYB |
| g1904.t1 | LBD | Vitvi13g01866.t01 | LBD | chr14.gff3_PROT_VIT_14s0219g00050.t01 | MYB |
| g19201.t1 | LBD | Vitvi13g01867.t01 | LBD | chr15.gff3_PROT_VIT_15s0021g02040.t01 | MYB |
| g19527.t1 | LBD | Vitvi14g01193.t01 | LBD | chr15.gff3_PROT_VIT_15s0046g00170.t01 | MYB |
| g20627.t1 | LBD | Vitvi14g01707.t01 | LBD | chr15.gff3_PROT_VIT_15s0046g03190.t01 | MYB |
| g21903.t1 | LBD | Vitvi14g01878.t01 | LBD | chr15.gff3_PROT_VIT_15s0048g02120.t01 | MYB |
| g21904.t1 | LBD | Vitvi15g00735.t01 | LBD | chr16.gff3_PROT_VIT_16s0013g01560.t01 | MYB |
| g24103.t1 | LBD | Vitvi15g00736.t01 | LBD | chr16.gff3_PROT_VIT_16s0013g01570.t01 | MYB |
| g26499.t1 | LBD | Vitvi15g01216.t01 | LBD | chr16.gff3_PROT_VIT_16s0022g01210.t01 | MYB |
| g26521.t1 | LBD | Vitvi15g01217.t01 | LBD | chr16.gff3_PROT_VIT_16s0039g01710.t01 | MYB |
| g26753.t1 | LBD | Vitvi16g00859.t01 | LBD | chr16.gff3_PROT_VIT_16s0039g01740.t01 | MYB |
| g26754.t1 | LBD | Vitvi16g01446.t01 | LBD | chr16.gff3_PROT_VIT_16s0039g01750.t01 | MYB |
| g26979.t1 | LBD | Vitvi17g00325.t01 | LBD | chr16.gff3_PROT_VIT_16s0050g00050.t01 | MYB |
| g28417.t1 | LBD | Vitvi17g00520.t01 | LBD | chr16.gff3_PROT_VIT_16s0050g00070.t01 | MYB |
| g28417.t2 | LBD | Vitvi17g00890.t01 | LBD | chr17.gff3_PROT_VIT_17s0000g01890.t01 | MYB |
| g31875.t1 | LBD | Vitvi18g00677.t01 | LBD | chr17.gff3_PROT_VIT_17s0000g02650.t01 | MYB |
| g31876.t1 | LBD | Vitvi19g01589.t01 | LBD | chr17.gff3_PROT_VIT_17s0000g02660.t01 | MYB |
| g32196.t1 | LBD | Vitvi17g00021.t01 | LFY | chr17.gff3_PROT_VIT_17s0000g02730.t01 | MYB |
| g3231.t1 | LBD | Vitvi02g00666.t01 | LSD | chr17.gff3_PROT_VIT_17s0000g03560.t01 | MYB |
| g32479.t1 | LBD | Vitvi10g00381.t01 | LSD | chr17.gff3_PROT_VIT_17s0000g04130.t01 | MYB |
| g3352.t1 | LBD | Vitvi10g01122.t01 | LSD | chr17.gff3_PROT_VIT_17s0000g05400.t01 | MYB |
| g35636.t1 | LBD | Vitvi01g00008.t01 | MIKC_MADS | chr17.gff3_PROT_VIT_17s0000g06190.t01 | MYB |
| g36020.t1 | LBD | Vitvi01g00011.t01 | MIKC_MADS | chr17.gff3_PROT_VIT_17s0000g06410.t01 | MYB |
| g36201.t1 | LBD | Vitvi01g00126.t01 | MIKC_MADS | chr17.gff3_PROT_VIT_17s0000g08480.t01 | MYB |
| g36202.t1 | LBD | Vitvi01g01673.t01 | MIKC_MADS | chr17.gff3_PROT_VIT_17s0000g08550.t01 | MYB |
| g36203.t1 | LBD | Vitvi01g01677.t01 | MIKC_MADS | chr17.gff3_PROT_VIT_17s0000g09080.t01 | MYB |
| g36205.t1 | LBD | Vitvi01g01885.t01 | MIKC_MADS | chr18.gff3_PROT_VIT_18s0001g05670.t01 | MYB |
| g36206.t1 | LBD | Vitvi02g00206.t01 | MIKC_MADS | chr18.gff3_PROT_VIT_18s0001g08470.t01 | MYB |
| g38289.t1 | LBD | Vitvi02g00427.t01 | MIKC_MADS | chr18.gff3_PROT_VIT_18s0001g09850.t01 | MYB |
| g5394.t1 | LBD | Vitvi02g01567.t01 | MIKC_MADS | chr18.gff3_PROT_VIT_18s0001g11170.t01 | MYB |
| g9041.t1 | LBD | Vitvi03g00819.t01 | MIKC_MADS | chr18.gff3_PROT_VIT_18s0001g15260.t01 | MYB |
| g33429.t1 | LFY | Vitvi03g01059.t01 | MIKC_MADS | chr18.gff3_PROT_VIT_18s0117g00200.t01 | MYB |
| g13119.t1 | LSD | Vitvi03g01320.t01 | MIKC_MADS | chr18.gff3_PROT_VIT_18s0117g00210.t01 | MYB |
| g15681.t1 | LSD | Vitvi04g01404.t01 | MIKC_MADS | chr18_random.gff3_PROT_VIT_18s0001g02210.t01 | MYB |
| g35456.t1 | LSD | Vitvi07g01441.t01 | MIKC_MADS | chr19.gff3_PROT_VIT_19s0014g03820.t01 | MYB |
| g11725.t1 | MIKC_MADS | Vitvi07g01516.t01 | MIKC_MADS | chr19.gff3_PROT_VIT_19s0015g01280.t01 | MYB |
| g14400.t1 | MIKC_MADS | Vitvi07g01520.t01 | MIKC_MADS | chr19.gff3_PROT_VIT_19s0085g00050.t01 | MYB |
| g15616.t1 | MIKC_MADS | Vitvi07g01792.t01 | MIKC_MADS | chr19.gff3_PROT_VIT_19s0085g00940.t01 | MYB |
| g16929.t1 | MIKC_MADS | Vitvi08g01935.t01 | MIKC_MADS | chr19.gff3_PROT_VIT_19s0090g00590.t01 | MYB |
| g17151.t1 | MIKC_MADS | Vitvi10g00663.t01 | MIKC_MADS | chr2.gff3_PROT_VIT_02s0012g01650.t01 | MYB |
| g17151.t2 | MIKC_MADS | Vitvi10g01395.t01 | MIKC_MADS | chr2.gff3_PROT_VIT_02s0025g00320.t01 | MYB |
| g17155.t1 | MIKC_MADS | Vitvi12g00019.t01 | MIKC_MADS | chr2.gff3_PROT_VIT_02s0025g04220.t01 | MYB |
| g18019.t1 | MIKC_MADS | Vitvi13g01861.t01 | MIKC_MADS | chr2.gff3_PROT_VIT_02s0033g00390.t01 | MYB |
| g19245.t1 | MIKC_MADS | Vitvi14g01341.t01 | MIKC_MADS | chr2.gff3_PROT_VIT_02s0033g00400.t01 | MYB |
| g19416.t1 | MIKC_MADS | Vitvi14g01344.t01 | MIKC_MADS | chr2.gff3_PROT_VIT_02s0033g00410.t01 | MYB |
| g19801.t1 | MIKC_MADS | Vitvi14g01526.t01 | MIKC_MADS | chr2.gff3_PROT_VIT_02s0033g00430.t01 | MYB |
| g19801.t2 | MIKC_MADS | Vitvi15g00225.t01 | MIKC_MADS | chr2.gff3_PROT_VIT_02s0033g00450.t01 | MYB |
| g20423.t1 | MIKC_MADS | Vitvi15g00774.t01 | MIKC_MADS | chr3.gff3_PROT_VIT_03s0038g02310.t01 | MYB |
| g20423.t2 | MIKC_MADS | Vitvi15g00776.t01 | MIKC_MADS | chr3.gff3_PROT_VIT_03s0180g00210.t01 | MYB |
| g20755.t1 | MIKC_MADS | Vitvi16g00894.t01 | MIKC_MADS | chr4.gff3_PROT_VIT_04s0008g00620.t01 | MYB |
| g21163.t1 | MIKC_MADS | Vitvi16g00898.t01 | MIKC_MADS | chr4.gff3_PROT_VIT_04s0008g01800.t01 | MYB |
| g21405.t1 | MIKC_MADS | Vitvi17g00098.t01 | MIKC_MADS | chr4.gff3_PROT_VIT_04s0008g01810.t01 | MYB |
| g21405.t2 | MIKC_MADS | Vitvi17g00470.t01 | MIKC_MADS | chr4.gff3_PROT_VIT_04s0008g01820.t01 | MYB |
| g21405.t3 | MIKC_MADS | Vitvi17g00471.t01 | MIKC_MADS | chr4.gff3_PROT_VIT_04s0008g01830.t01 | MYB |
| g22154.t1 | MIKC_MADS | Vitvi18g00221.t01 | MIKC_MADS | chr4.gff3_PROT_VIT_04s0008g01840.t01 | MYB |
| g24225.t1 | MIKC_MADS | Vitvi18g00361.t01 | MIKC_MADS | chr4.gff3_PROT_VIT_04s0008g01870.t01 | MYB |
| g24227.t1 | MIKC_MADS | Vitvi18g00517.t01 | MIKC_MADS | chr4.gff3_PROT_VIT_04s0008g03780.t01 | MYB |
| g24246.t1 | MIKC_MADS | Vitvi18g00553.t01 | MIKC_MADS | chr4.gff3_PROT_VIT_04s0008g03790.t01 | MYB |
| g24246.t2 | MIKC_MADS | Vitvi18g00700.t01 | MIKC_MADS | chr4.gff3_PROT_VIT_04s0023g03710.t01 | MYB |
| g25572.t1 | MIKC_MADS | Vitvi18g01044.t01 | MIKC_MADS | chr4.gff3_PROT_VIT_04s0044g01380.t01 | MYB |
| g25901.t1 | MIKC_MADS | Vitvi18g02133.t01 | MIKC_MADS | chr5.gff3_PROT_VIT_05s0020g01100.t01 | MYB |
| g2597.t1 | MIKC_MADS | Vitvi18g02145.t01 | MIKC_MADS | chr5.gff3_PROT_VIT_05s0049g01010.t01 | MYB |
| g268.t1 | MIKC_MADS | Vitvi00g01439.t01 | M-type_MADS | chr5.gff3_PROT_VIT_05s0049g01020.t01 | MYB |
| g28775.t1 | MIKC_MADS | Vitvi01g01424.t01 | M-type_MADS | chr5.gff3_PROT_VIT_05s0049g02260.t01 | MYB |
| g28776.t1 | MIKC_MADS | Vitvi01g01831.t01 | M-type_MADS | chr5.gff3_PROT_VIT_05s0077g00500.t01 | MYB |
| g29795.t1 | MIKC_MADS | Vitvi02g01303.t01 | M-type_MADS | chr5.gff3_PROT_VIT_05s0077g01360.t01 | MYB |
| g29797.t1 | MIKC_MADS | Vitvi02g01306.t01 | M-type_MADS | chr6.gff3_PROT_VIT_06s0004g00570.t01 | MYB |
| g29972.t1 | MIKC_MADS | Vitvi03g00729.t01 | M-type_MADS | chr6.gff3_PROT_VIT_06s0004g02110.t01 | MYB |
| g29972.t2 | MIKC_MADS | Vitvi03g00730.t01 | M-type_MADS | chr6.gff3_PROT_VIT_06s0004g04140.t01 | MYB |
| g33612.t1 | MIKC_MADS | Vitvi03g00732.t01 | M-type_MADS | chr6.gff3_PROT_VIT_06s0004g06100.t01 | MYB |
| g33930.t1 | MIKC_MADS | Vitvi03g00733.t01 | M-type_MADS | chr6.gff3_PROT_VIT_06s0004g06280.t01 | MYB |
| g35674.t1 | MIKC_MADS | Vitvi03g00734.t01 | M-type_MADS | chr6.gff3_PROT_VIT_06s0009g02480.t01 | MYB |
| g35675.t1 | MIKC_MADS | Vitvi03g01067.t01 | M-type_MADS | chr6.gff3_PROT_VIT_06s0061g00470.t01 | MYB |
| g36000.t1 | MIKC_MADS | Vitvi03g01317.t01 | M-type_MADS | chr6.gff3_PROT_VIT_06s0080g00790.t01 | MYB |
| g38107.t1 | MIKC_MADS | Vitvi03g01318.t01 | M-type_MADS | chr7.gff3_PROT_VIT_07s0005g01210.t01 | MYB |
| g902.t1 | MIKC_MADS | Vitvi04g00171.t01 | M-type_MADS | chr7.gff3_PROT_VIT_07s0005g01950.t01 | MYB |
| g10608.t1 | M-type_MADS | Vitvi04g01016.t01 | M-type_MADS | chr7.gff3_PROT_VIT_07s0005g02480.t01 | MYB |
| g16550.t1 | M-type_MADS | Vitvi05g01725.t01 | M-type_MADS | chr7.gff3_PROT_VIT_07s0005g03340.t01 | MYB |
| g17649.t1 | M-type_MADS | Vitvi05g01726.t01 | M-type_MADS | chr7.gff3_PROT_VIT_07s0129g01050.t01 | MYB |
| g18789.t1 | M-type_MADS | Vitvi05g01727.t01 | M-type_MADS | chr7.gff3_PROT_VIT_07s0130g00040.t01 | MYB |
| g18790.t1 | M-type_MADS | Vitvi05g01728.t01 | M-type_MADS | chr7_random.gff3_PROT_VIT_07s0141g00100.t01 | MYB |
| g18792.t1 | M-type_MADS | Vitvi05g01729.t01 | M-type_MADS | chr8.gff3_PROT_VIT_08s0007g00360.t01 | MYB |
| g18793.t1 | M-type_MADS | Vitvi05g01730.t01 | M-type_MADS | chr8.gff3_PROT_VIT_08s0007g00410.t01 | MYB |
| g19862.t1 | M-type_MADS | Vitvi07g02071.t01 | M-type_MADS | chr8.gff3_PROT_VIT_08s0007g01540.t01 | MYB |
| g19948.t1 | M-type_MADS | Vitvi07g02072.t01 | M-type_MADS | chr8.gff3_PROT_VIT_08s0007g01920.t01 | MYB |
| g20076.t1 | M-type_MADS | Vitvi07g03065.t01 | M-type_MADS | chr8.gff3_PROT_VIT_08s0007g04830.t01 | MYB |
| g20090.t1 | M-type_MADS | Vitvi08g01967.t01 | M-type_MADS | chr8.gff3_PROT_VIT_08s0007g05030.t01 | MYB |
| g20176.t1 | M-type_MADS | Vitvi08g01968.t01 | M-type_MADS | chr8.gff3_PROT_VIT_08s0007g07230.t01 | MYB |
| g20599.t1 | M-type_MADS | Vitvi10g00842.t01 | M-type_MADS | chr8.gff3_PROT_VIT_08s0056g00800.t01 | MYB |
| g21471.t1 | M-type_MADS | Vitvi10g01588.t01 | M-type_MADS | chr8.gff3_PROT_VIT_08s0056g01190.t01 | MYB |
| g21749.t1 | M-type_MADS | Vitvi10g01589.t01 | M-type_MADS | chr9.gff3_PROT_VIT_09s0002g01380.t01 | MYB |
| g21752.t1 | M-type_MADS | Vitvi10g01590.t01 | M-type_MADS | chr9.gff3_PROT_VIT_09s0002g01400.t01 | MYB |
| g22009.t1 | M-type_MADS | Vitvi10g01591.t01 | M-type_MADS | chr9.gff3_PROT_VIT_09s0002g01410.t01 | MYB |
| g22011.t1 | M-type_MADS | Vitvi10g01592.t01 | M-type_MADS | chr9.gff3_PROT_VIT_09s0002g01670.t01 | MYB |
| g23022.t1 | M-type_MADS | Vitvi10g01593.t01 | M-type_MADS | chrUn.gff3_PROT_VIT_00s0203g00070.t01 | MYB |
| g23655.t1 | M-type_MADS | Vitvi12g00559.t01 | M-type_MADS | chrUn.gff3_PROT_VIT_00s0203g00170.t01 | MYB |
| g25763.t1 | M-type_MADS | Vitvi13g02085.t01 | M-type_MADS | chrUn.gff3_PROT_VIT_00s0341g00050.t01 | MYB |
| g2595.t1 | M-type_MADS | Vitvi14g00026.t01 | M-type_MADS | chrUn.gff3_PROT_VIT_00s1241g00010.t01 | MYB |
| g2596.t1 | M-type_MADS | Vitvi15g01207.t01 | M-type_MADS | chrUn.gff3_PROT_VIT_00s1352g00010.t01 | MYB |
| g2598.t1 | M-type_MADS | Vitvi15g01208.t01 | M-type_MADS | chr1.gff3_PROT_VIT_01s0011g00790.t01 | MYB_related |
| g27804.t1 | M-type_MADS | Vitvi15g01209.t01 | M-type_MADS | chr1.gff3_PROT_VIT_01s0026g01050.t01 | MYB_related |
| g30374.t1 | M-type_MADS | Vitvi15g01210.t01 | M-type_MADS | chr10.gff3_PROT_VIT_10s0116g00500.t01 | MYB_related |
| g30679.t1 | M-type_MADS | Vitvi15g01211.t01 | M-type_MADS | chr10.gff3_PROT_VIT_10s0116g01760.t01 | MYB_related |
| g32510.t1 | M-type_MADS | Vitvi15g01212.t01 | M-type_MADS | chr11.gff3_PROT_VIT_11s0016g01890.t01 | MYB_related |
| g32511.t1 | M-type_MADS | Vitvi15g01213.t01 | M-type_MADS | chr11.gff3_PROT_VIT_11s0016g02410.t01 | MYB_related |
| g32512.t1 | M-type_MADS | Vitvi15g01214.t01 | M-type_MADS | chr11.gff3_PROT_VIT_11s0016g05650.t01 | MYB_related |
| g32513.t1 | M-type_MADS | Vitvi15g01743.t01 | M-type_MADS | chr12.gff3_PROT_VIT_12s0057g01490.t01 | MYB_related |
| g32514.t1 | M-type_MADS | Vitvi17g00614.t01 | M-type_MADS | chr12.gff3_PROT_VIT_12s0059g02360.t01 | MYB_related |
| g33676.t1 | M-type_MADS | Vitvi17g01306.t01 | M-type_MADS | chr13.gff3_PROT_VIT_13s0019g01670.t01 | MYB_related |
| g33767.t1 | M-type_MADS | Vitvi17g01307.t01 | M-type_MADS | chr13.gff3_PROT_VIT_13s0106g00370.t01 | MYB_related |
| g33772.t1 | M-type_MADS | Vitvi17g01308.t01 | M-type_MADS | chr13.gff3_PROT_VIT_13s0156g00340.t01 | MYB_related |
| g3437.t1 | M-type_MADS | Vitvi19g00027.t01 | M-type_MADS | chr14.gff3_PROT_VIT_14s0036g00460.t01 | MYB_related |
| g35443.t1 | M-type_MADS | Vitvi19g01487.t01 | M-type_MADS | chr14.gff3_PROT_VIT_14s0036g00500.t01 | MYB_related |
| g35741.t1 | M-type_MADS | Vitvi19g01491.t01 | M-type_MADS | chr14.gff3_PROT_VIT_14s0036g00510.t01 | MYB_related |
| g35999.t1 | M-type_MADS | Vitvi19g02367.t01 | M-type_MADS | chr14.gff3_PROT_VIT_14s0128g00860.t01 | MYB_related |
| g36185.t1 | M-type_MADS | Vitvi01g00094.t01 | MYB | chr15.gff3_PROT_VIT_15s0046g01130.t01 | MYB_related |
| g36665.t1 | M-type_MADS | Vitvi01g00095.t01 | MYB | chr15.gff3_PROT_VIT_15s0046g01790.t01 | MYB_related |
| g37305.t1 | M-type_MADS | Vitvi01g00302.t01 | MYB | chr15.gff3_PROT_VIT_15s0046g02260.t01 | MYB_related |
| g5931.t1 | M-type_MADS | Vitvi01g00401.t01 | MYB | chr15.gff3_PROT_VIT_15s0048g02410.t01 | MYB_related |
| g7114.t1 | M-type_MADS | Vitvi01g00737.t01 | MYB | chr16.gff3_PROT_VIT_16s0039g01920.t01 | MYB_related |
| g7714.t1 | M-type_MADS | Vitvi01g00956.t01 | MYB | chr16.gff3_PROT_VIT_16s0050g01180.t01 | MYB_related |
| g7715.t1 | M-type_MADS | Vitvi01g01028.t01 | MYB | chr16.gff3_PROT_VIT_16s0050g02530.t01 | MYB_related |
| g8177.t1 | M-type_MADS | Vitvi01g01052.t01 | MYB | chr17.gff3_PROT_VIT_17s0000g07510.t01 | MYB_related |
| g8818.t1 | M-type_MADS | Vitvi01g01271.t01 | MYB | chr18.gff3_PROT_VIT_18s0001g02160.t01 | MYB_related |
| g10287.t1 | MYB | Vitvi02g00028.t01 | MYB | chr18.gff3_PROT_VIT_18s0001g07620.t01 | MYB_related |
| g1047.t1 | MYB | Vitvi02g00195.t01 | MYB | chr18.gff3_PROT_VIT_18s0001g12530.t01 | MYB_related |
| g11583.t1 | MYB | Vitvi02g00725.t01 | MYB | chr18.gff3_PROT_VIT_18s0001g12610.t01 | MYB_related |
| g11584.t1 | MYB | Vitvi02g01015.t01 | MYB | chr18_random.gff3_PROT_VIT_18s0001g01170.t01 | MYB_related |
| g11768.t1 | MYB | Vitvi02g01019.t01 | MYB | chr19.gff3_PROT_VIT_19s0015g01170.t01 | MYB_related |
| g12303.t1 | MYB | Vitvi02g01024.t01 | MYB | chr2.gff3_PROT_VIT_02s0012g00630.t01 | MYB_related |
| g12350.t1 | MYB | Vitvi02g01307.t01 | MYB | chr2.gff3_PROT_VIT_02s0033g00370.t01 | MYB_related |
| g12485.t1 | MYB | Vitvi02g01308.t01 | MYB | chr2.gff3_PROT_VIT_02s0033g00440.t01 | MYB_related |
| g12605.t1 | MYB | Vitvi02g01400.t01 | MYB | chr2.gff3_PROT_VIT_02s0033g00460.t01 | MYB_related |
| g1281.t1 | MYB | Vitvi02g01732.t01 | MYB | chr3.gff3_PROT_VIT_03s0038g04460.t01 | MYB_related |
| g12826.t1 | MYB | Vitvi02g01823.t01 | MYB | chr3.gff3_PROT_VIT_03s0063g02620.t01 | MYB_related |
| g13148.t1 | MYB | Vitvi03g00136.t01 | MYB | chr3.gff3_PROT_VIT_03s0063g02670.t01 | MYB_related |
| g13157.t1 | MYB | Vitvi03g00559.t01 | MYB | chr3.gff3_PROT_VIT_03s0063g02680.t01 | MYB_related |
| g13376.t1 | MYB | Vitvi04g00049.t01 | MYB | chr3.gff3_PROT_VIT_03s0091g01030.t01 | MYB_related |
| g13376.t2 | MYB | Vitvi04g00153.t01 | MYB | chr4.gff3_PROT_VIT_04s0008g00900.t01 | MYB_related |
| g13581.t1 | MYB | Vitvi04g00155.t01 | MYB | chr4.gff3_PROT_VIT_04s0023g01910.t01 | MYB_related |
| g13736.t1 | MYB | Vitvi04g00156.t01 | MYB | chr4.gff3_PROT_VIT_04s0079g00410.t01 | MYB_related |
| g13773.t1 | MYB | Vitvi04g00157.t01 | MYB | chr5.gff3_PROT_VIT_05s0020g01610.t01 | MYB_related |
| g13970.t1 | MYB | Vitvi04g00158.t01 | MYB | chr6.gff3_PROT_VIT_06s0004g02520.t01 | MYB_related |
| g14472.t1 | MYB | Vitvi04g00160.t01 | MYB | chr6.gff3_PROT_VIT_06s0004g04940.t01 | MYB_related |
| g14651.t1 | MYB | Vitvi04g00331.t01 | MYB | chr6.gff3_PROT_VIT_06s0004g08050.t01 | MYB_related |
| g15207.t1 | MYB | Vitvi04g01486.t01 | MYB | chr7.gff3_PROT_VIT_07s0005g01910.t01 | MYB_related |
| g15752.t1 | MYB | Vitvi04g01697.t01 | MYB | chr7.gff3_PROT_VIT_07s0031g02280.t01 | MYB_related |
| g15851.t1 | MYB | Vitvi04g01842.t01 | MYB | chr7_random.gff3_PROT_VIT_07s0151g00480.t01 | MYB_related |
| g15957.t1 | MYB | Vitvi04g01855.t01 | MYB | chr8.gff3_PROT_VIT_08s0007g00500.t01 | MYB_related |
| g15987.t1 | MYB | Vitvi05g00084.t01 | MYB | chr8.gff3_PROT_VIT_08s0007g08160.t01 | MYB_related |
| g16097.t1 | MYB | Vitvi05g00166.t01 | MYB | chr8.gff3_PROT_VIT_08s0040g01640.t01 | MYB_related |
| g16275.t1 | MYB | Vitvi05g00275.t01 | MYB | chr8.gff3_PROT_VIT_08s0040g03220.t01 | MYB_related |
| g16350.t1 | MYB | Vitvi05g00861.t01 | MYB | chr9.gff3_PROT_VIT_09s0002g01480.t01 | MYB_related |
| g17011.t1 | MYB | Vitvi05g01732.t01 | MYB | chr9.gff3_PROT_VIT_09s0002g03540.t01 | MYB_related |
| g17095.t1 | MYB | Vitvi05g01733.t01 | MYB | chr9.gff3_PROT_VIT_09s0002g03610.t01 | MYB_related |
| g17113.t1 | MYB | Vitvi06g00059.t01 | MYB | chrUn.gff3_PROT_VIT_00s0299g00060.t01 | MYB_related |
| g17515.t1 | MYB | Vitvi06g00214.t01 | MYB | chrUn.gff3_PROT_VIT_00s1311g00020.t01 | MYB_related |
| g17534.t1 | MYB | Vitvi06g00414.t01 | MYB | chr1.gff3_PROT_VIT_01s0011g02990.t01 | NAC |
| g17729.t1 | MYB | Vitvi06g00592.t01 | MYB | chr1.gff3_PROT_VIT_01s0026g02360.t01 | NAC |
| g17920.t1 | MYB | Vitvi06g00611.t01 | MYB | chr1.gff3_PROT_VIT_01s0026g02710.t01 | NAC |
| g18051.t1 | MYB | Vitvi06g01139.t01 | MYB | chr1.gff3_PROT_VIT_01s0146g00280.t01 | NAC |
| g18211.t1 | MYB | Vitvi06g01321.t01 | MYB | chr10.gff3_PROT_VIT_10s0003g00350.t01 | NAC |
| g18408.t1 | MYB | Vitvi06g01516.t01 | MYB | chr11.gff3_PROT_VIT_11s0016g02880.t01 | NAC |
| g18636.t1 | MYB | Vitvi07g00393.t01 | MYB | chr12.gff3_PROT_VIT_12s0028g00860.t01 | NAC |
| g19001.t1 | MYB | Vitvi07g00455.t01 | MYB | chr12.gff3_PROT_VIT_12s0028g03050.t01 | NAC |
| g19466.t1 | MYB | Vitvi07g00515.t01 | MYB | chr12.gff3_PROT_VIT_12s0035g02020.t01 | NAC |
| g19683.t1 | MYB | Vitvi07g00598.t01 | MYB | chr12.gff3_PROT_VIT_12s0055g00510.t01 | NAC |
| g19855.t1 | MYB | Vitvi07g01676.t01 | MYB | chr13.gff3_PROT_VIT_13s0019g05230.t01 | NAC |
| g20290.t1 | MYB | Vitvi07g02009.t01 | MYB | chr13.gff3_PROT_VIT_13s0019g05240.t01 | NAC |
| g20557.t1 | MYB | Vitvi07g02075.t01 | MYB | chr14.gff3_PROT_VIT_14s0066g00360.t01 | NAC |
| g20700.t1 | MYB | Vitvi07g03055.t01 | MYB | chr14.gff3_PROT_VIT_14s0068g01490.t01 | NAC |
| g21037.t1 | MYB | Vitvi08g00069.t01 | MYB | chr14.gff3_PROT_VIT_14s0108g00860.t01 | NAC |
| g2113.t1 | MYB | Vitvi08g00107.t01 | MYB | chr14.gff3_PROT_VIT_14s0108g01070.t01 | NAC |
| g21692.t1 | MYB | Vitvi08g01203.t01 | MYB | chr15.gff3_PROT_VIT_15s0048g02270.t01 | NAC |
| g21693.t1 | MYB | Vitvi08g01298.t01 | MYB | chr15.gff3_PROT_VIT_15s0048g02280.t01 | NAC |
| g2177.t1 | MYB | Vitvi08g01336.t01 | MYB | chr15.gff3_PROT_VIT_15s0048g02290.t01 | NAC |
| g22067.t1 | MYB | Vitvi08g01607.t01 | MYB | chr15.gff3_PROT_VIT_15s0048g02300.t01 | NAC |
| g22085.t1 | MYB | Vitvi08g01623.t01 | MYB | chr15.gff3_PROT_VIT_15s0048g02310.t01 | NAC |
| g22086.t1 | MYB | Vitvi08g01797.t01 | MYB | chr15.gff3_PROT_VIT_15s0048g02320.t01 | NAC |
| g22302.t1 | MYB | Vitvi08g02196.t01 | MYB | chr15.gff3_PROT_VIT_15s0048g02340.t01 | NAC |
| g22303.t1 | MYB | Vitvi09g00110.t01 | MYB | chr15.gff3_PROT_VIT_15s0048g02660.t01 | NAC |
| g22304.t1 | MYB | Vitvi09g00112.t01 | MYB | chr16.gff3_PROT_VIT_16s0022g00690.t01 | NAC |
| g22617.t1 | MYB | Vitvi09g00113.t01 | MYB | chr16.gff3_PROT_VIT_16s0098g00760.t01 | NAC |
| g229.t1 | MYB | Vitvi09g00142.t01 | MYB | chr17.gff3_PROT_VIT_17s0000g00770.t01 | NAC |
| g23078.t1 | MYB | Vitvi10g00329.t01 | MYB | chr17.gff3_PROT_VIT_17s0000g03660.t01 | NAC |
| g23422.t1 | MYB | Vitvi10g00345.t01 | MYB | chr17.gff3_PROT_VIT_17s0000g06400.t01 | NAC |
| g2391.t1 | MYB | Vitvi10g01533.t01 | MYB | chr17.gff3_PROT_VIT_17s0000g06990.t01 | NAC |
| g2392.t1 | MYB | Vitvi11g00097.t01 | MYB | chr17.gff3_PROT_VIT_17s0053g00740.t01 | NAC |
| g24050.t1 | MYB | Vitvi11g00098.t01 | MYB | chr18.gff3_PROT_VIT_18s0001g02300.t01 | NAC |
| g24064.t1 | MYB | Vitvi11g00099.t01 | MYB | chr18.gff3_PROT_VIT_18s0001g10250.t01 | NAC |
| g24228.t1 | MYB | Vitvi11g00228.t01 | MYB | chr18.gff3_PROT_VIT_18s0001g11980.t01 | NAC |
| g24535.t1 | MYB | Vitvi11g00320.t01 | MYB | chr18.gff3_PROT_VIT_18s0041g00700.t01 | NAC |
| g24587.t1 | MYB | Vitvi11g00499.t01 | MYB | chr18.gff3_PROT_VIT_18s0072g01060.t01 | NAC |
| g24592.t1 | MYB | Vitvi11g00502.t01 | MYB | chr18.gff3_PROT_VIT_18s0089g01120.t01 | NAC |
| g24677.t1 | MYB | Vitvi11g01283.t01 | MYB | chr18.gff3_PROT_VIT_18s0122g00800.t01 | NAC |
| g24682.t1 | MYB | Vitvi11g01323.t01 | MYB | chr18_random.gff3_PROT_VIT_18s0001g01820.t01 | NAC |
| g24848.t1 | MYB | Vitvi12g00376.t01 | MYB | chr19.gff3_PROT_VIT_19s0014g02200.t01 | NAC |
| g24853.t1 | MYB | Vitvi12g00625.t01 | MYB | chr19.gff3_PROT_VIT_19s0014g03290.t01 | NAC |
| g2507.t1 | MYB | Vitvi12g00626.t01 | MYB | chr19.gff3_PROT_VIT_19s0014g03300.t01 | NAC |
| g25123.t1 | MYB | Vitvi12g00632.t01 | MYB | chr19.gff3_PROT_VIT_19s0027g00230.t01 | NAC |
| g25231.t1 | MYB | Vitvi13g00076.t01 | MYB | chr19.gff3_PROT_VIT_19s0027g00840.t01 | NAC |
| g25236.t1 | MYB | Vitvi13g00494.t01 | MYB | chr19.gff3_PROT_VIT_19s0027g00860.t01 | NAC |
| g25237.t1 | MYB | Vitvi13g00510.t01 | MYB | chr19.gff3_PROT_VIT_19s0027g00870.t01 | NAC |
| g25488.t1 | MYB | Vitvi13g00573.t01 | MYB | chr19.gff3_PROT_VIT_19s0027g00880.t01 | NAC |
| g25720.t1 | MYB | Vitvi13g01266.t01 | MYB | chr19.gff3_PROT_VIT_19s0027g00910.t01 | NAC |
| g25721.t1 | MYB | Vitvi13g01359.t01 | MYB | chr19.gff3_PROT_VIT_19s0085g00950.t01 | NAC |
| g25729.t1 | MYB | Vitvi13g01737.t01 | MYB | chr19.gff3_PROT_VIT_19s0085g01210.t01 | NAC |
| g25869.t1 | MYB | Vitvi14g00018.t01 | MYB | chr2.gff3_PROT_VIT_02s0012g01040.t01 | NAC |
| g25870.t1 | MYB | Vitvi14g00612.t01 | MYB | chr2.gff3_PROT_VIT_02s0025g02710.t01 | NAC |
| g26011.t1 | MYB | Vitvi14g00618.t01 | MYB | chr2.gff3_PROT_VIT_02s0025g03020.t01 | NAC |
| g2603.t1 | MYB | Vitvi14g00925.t01 | MYB | chr2.gff3_PROT_VIT_02s0154g00020.t01 | NAC |
| g26111.t1 | MYB | Vitvi14g00930.t01 | MYB | chr3.gff3_PROT_VIT_03s0038g03410.t01 | NAC |
| g26493.t1 | MYB | Vitvi14g00940.t01 | MYB | chr4.gff3_PROT_VIT_04s0008g02710.t01 | NAC |
| g26747.t1 | MYB | Vitvi14g00974.t01 | MYB | chr4.gff3_PROT_VIT_04s0008g06550.t01 | NAC |
| g26803.t1 | MYB | Vitvi14g01247.t01 | MYB | chr4.gff3_PROT_VIT_04s0023g03110.t01 | NAC |
| g27073.t1 | MYB | Vitvi14g01346.t01 | MYB | chr4.gff3_PROT_VIT_04s0044g01220.t01 | NAC |
| g27091.t1 | MYB | Vitvi14g01615.t01 | MYB | chr4.gff3_PROT_VIT_04s0044g01500.t01 | NAC |
| g27100.t1 | MYB | Vitvi14g01740.t01 | MYB | chr4.gff3_PROT_VIT_04s0079g00280.t01 | NAC |
| g27273.t1 | MYB | Vitvi14g01750.t01 | MYB | chr4_random.gff3_PROT_VIT_04s0008g00150.t01 | NAC |
| g27329.t1 | MYB | Vitvi14g01845.t01 | MYB | chr6.gff3_PROT_VIT_06s0004g00020.t01 | NAC |
| g27750.t1 | MYB | Vitvi14g01960.t01 | MYB | chr6.gff3_PROT_VIT_06s0004g02340.t01 | NAC |
| g27755.t1 | MYB | Vitvi14g01976.t01 | MYB | chr6.gff3_PROT_VIT_06s0004g03080.t01 | NAC |
| g27758.t1 | MYB | Vitvi14g01987.t01 | MYB | chr6.gff3_PROT_VIT_06s0080g00780.t01 | NAC |
| g28207.t1 | MYB | Vitvi14g02430.t01 | MYB | chr6.gff3_PROT_VIT_06s0080g00970.t01 | NAC |
| g28423.t1 | MYB | Vitvi14g03020.t01 | MYB | chr7.gff3_PROT_VIT_07s0005g03610.t01 | NAC |
| g2903.t1 | MYB | Vitvi15g00594.t01 | MYB | chr7.gff3_PROT_VIT_07s0031g02610.t01 | NAC |
| g29074.t1 | MYB | Vitvi15g00847.t01 | MYB | chr8.gff3_PROT_VIT_08s0007g02940.t01 | NAC |
| g29077.t1 | MYB | Vitvi15g00938.t01 | MYB | chr8.gff3_PROT_VIT_08s0007g07640.t01 | NAC |
| g29703.t1 | MYB | Vitvi15g01161.t01 | MYB | chr8.gff3_PROT_VIT_08s0007g07670.t01 | NAC |
| g29729.t1 | MYB | Vitvi16g00098.t01 | MYB | chr8.gff3_PROT_VIT_08s0040g02110.t01 | NAC |
| g29760.t1 | MYB | Vitvi16g00103.t01 | MYB | chrUn.gff3_PROT_VIT_00s0375g00040.t01 | NAC |
| g29881.t1 | MYB | Vitvi16g00106.t01 | MYB | chr13.gff3_PROT_VIT_13s0067g00920.t01 | NF-X1 |
| g30634.t1 | MYB | Vitvi16g00125.t01 | MYB | chr17.gff3_PROT_VIT_17s0000g01920.t01 | NF-X1 |
| g30635.t1 | MYB | Vitvi16g00441.t01 | MYB | chr19.gff3_PROT_VIT_19s0014g00390.t01 | NF-X1 |
| g30825.t1 | MYB | Vitvi16g00442.t01 | MYB | chr10.gff3_PROT_VIT_10s0003g04650.t01 | NF-YA |
| g30828.t1 | MYB | Vitvi16g00775.t01 | MYB | chr11.gff3_PROT_VIT_11s0016g01480.t01 | NF-YA |
| g30830.t1 | MYB | Vitvi16g01015.t01 | MYB | chr13.gff3_PROT_VIT_13s0064g00860.t01 | NF-YA |
| g30834.t1 | MYB | Vitvi16g01017.t01 | MYB | chr6.gff3_PROT_VIT_06s0004g02270.t01 | NF-YA |
| g30835.t1 | MYB | Vitvi16g01448.t01 | MYB | chr8.gff3_PROT_VIT_08s0007g08250.t01 | NF-YA |
| g30839.t1 | MYB | Vitvi16g01522.t01 | MYB | chr8.gff3_PROT_VIT_08s0032g01190.t01 | NF-YA |
| g30842.t1 | MYB | Vitvi16g01523.t01 | MYB | chr9.gff3_PROT_VIT_09s0002g01590.t01 | NF-YA |
| g31052.t1 | MYB | Vitvi16g01689.t01 | MYB | chr1.gff3_PROT_VIT_01s0010g01910.t01 | NF-YB |
| g31078.t1 | MYB | Vitvi17g00159.t01 | MYB | chr1.gff3_PROT_VIT_01s0010g01940.t01 | NF-YB |
| g31080.t1 | MYB | Vitvi17g00231.t01 | MYB | chr12.gff3_PROT_VIT_12s0057g01010.t01 | NF-YB |
| g31082.t1 | MYB | Vitvi17g00232.t01 | MYB | chr13.gff3_PROT_VIT_13s0019g03600.t01 | NF-YB |
| g31335.t1 | MYB | Vitvi17g00238.t01 | MYB | chr14.gff3_PROT_VIT_14s0060g02660.t01 | NF-YB |
| g32182.t1 | MYB | Vitvi17g00309.t01 | MYB | chr17.gff3_PROT_VIT_17s0000g04350.t01 | NF-YB |
| g32204.t1 | MYB | Vitvi17g00366.t01 | MYB | chr19.gff3_PROT_VIT_19s0015g00440.t01 | NF-YB |
| g32787.t1 | MYB | Vitvi17g00510.t01 | MYB | chr19.gff3_PROT_VIT_19s0015g00580.t01 | NF-YB |
| g3287.t1 | MYB | Vitvi17g00598.t01 | MYB | chr19.gff3_PROT_VIT_19s0015g00590.t01 | NF-YB |
| g32947.t1 | MYB | Vitvi17g00623.t01 | MYB | chr5.gff3_PROT_VIT_05s0020g01350.t01 | NF-YB |
| g33234.t1 | MYB | Vitvi17g00822.t01 | MYB | chr6.gff3_PROT_VIT_06s0004g03490.t01 | NF-YB |
| g33806.t1 | MYB | Vitvi17g00832.t01 | MYB | chr6.gff3_PROT_VIT_06s0080g00460.t01 | NF-YB |
| g3420.t1 | MYB | Vitvi17g00895.t01 | MYB | chr7.gff3_PROT_VIT_07s0031g01460.t01 | NF-YB |
| g3421.t1 | MYB | Vitvi18g00406.t01 | MYB | chr7.gff3_PROT_VIT_07s0104g01640.t01 | NF-YB |
| g3422.t1 | MYB | Vitvi18g00605.t01 | MYB | chr8.gff3_PROT_VIT_08s0040g03260.t01 | NF-YB |
| g3423.t1 | MYB | Vitvi18g00725.t01 | MYB | chrUn.gff3_PROT_VIT_00s0259g00180.t01 | NF-YB |
| g3424.t1 | MYB | Vitvi18g00843.t01 | MYB | chrUn.gff3_PROT_VIT_00s0956g00020.t01 | NF-YB |
| g35922.t1 | MYB | Vitvi18g01209.t01 | MYB | chrUn.gff3_PROT_VIT_00s1287g00010.t01 | NF-YB |
| g35935.t1 | MYB | Vitvi18g01533.t01 | MYB | chr1.gff3_PROT_VIT_01s0010g03550.t01 | NF-YC |
| g36077.t1 | MYB | Vitvi18g01867.t01 | MYB | chr13.gff3_PROT_VIT_13s0084g00040.t01 | NF-YC |
| g36079.t1 | MYB | Vitvi18g01868.t01 | MYB | chr14.gff3_PROT_VIT_14s0006g02320.t01 | NF-YC |
| g3619.t1 | MYB | Vitvi19g00306.t01 | MYB | chr14.gff3_PROT_VIT_14s0128g00250.t01 | NF-YC |
| g3620.t1 | MYB | Vitvi19g00508.t01 | MYB | chr17.gff3_PROT_VIT_17s0000g01130.t01 | NF-YC |
| g3621.t1 | MYB | Vitvi19g00758.t01 | MYB | chr19.gff3_PROT_VIT_19s0085g00520.t01 | NF-YC |
| g36227.t1 | MYB | Vitvi19g01669.t01 | MYB | chr2.gff3_PROT_VIT_02s0025g03770.t01 | NF-YC |
| g36686.t1 | MYB | Vitvi19g01749.t01 | MYB | chr5.gff3_PROT_VIT_05s0020g02790.t01 | NF-YC |
| g36924.t1 | MYB | Vitvi01g00071.t01 | MYB_related | chr6.gff3_PROT_VIT_06s0004g03030.t01 | NF-YC |
| g37000.t1 | MYB | Vitvi01g00867.t01 | MYB_related | chr6.gff3_PROT_VIT_06s0009g02120.t01 | NF-YC |
| g37762.t1 | MYB | Vitvi02g00196.t01 | MYB_related | chr12.gff3_PROT_VIT_12s0028g00370.t01 | Nin-like |
| g37764.t1 | MYB | Vitvi02g00645.t01 | MYB_related | chr16.gff3_PROT_VIT_16s0098g01730.t01 | Nin-like |
| g37765.t1 | MYB | Vitvi02g01013.t01 | MYB_related | chr17.gff3_PROT_VIT_17s0000g06250.t01 | Nin-like |
| g37767.t1 | MYB | Vitvi02g01017.t01 | MYB_related | chr18.gff3_PROT_VIT_18s0122g01290.t01 | Nin-like |
| g37769.t1 | MYB | Vitvi02g01022.t01 | MYB_related | chr2.gff3_PROT_VIT_02s0025g02070.t01 | Nin-like |
| g37769.t2 | MYB | Vitvi02g01309.t01 | MYB_related | chr3.gff3_PROT_VIT_03s0038g03710.t01 | Nin-like |
| g37779.t1 | MYB | Vitvi02g01721.t01 | MYB_related | chr4.gff3_PROT_VIT_04s0044g00060.t01 | Nin-like |
| g4257.t1 | MYB | Vitvi03g00293.t01 | MYB_related | chr5.gff3_PROT_VIT_05s0094g00020.t01 | Nin-like |
| g4276.t1 | MYB | Vitvi03g00318.t01 | MYB_related | chr19.gff3_PROT_VIT_19s0014g01700.t01 | NZZ/SPL |
| g439.t1 | MYB | Vitvi03g00325.t01 | MYB_related | chr19.gff3_PROT_VIT_19s0014g03940.t01 | NZZ/SPL |
| g5037.t1 | MYB | Vitvi03g00668.t01 | MYB_related | chr1.gff3_PROT_VIT_01s0011g03070.t01 | RAV |
| g5077.t1 | MYB | Vitvi03g01247.t01 | MYB_related | chr14.gff3_PROT_VIT_14s0083g00130.t01 | RAV |
| g5161.t1 | MYB | Vitvi03g01495.t01 | MYB_related | chr11.gff3_PROT_VIT_11s0037g00010.t01 | RAV |
| g5233.t1 | MYB | Vitvi04g00076.t01 | MYB_related | chr6.gff3_PROT_VIT_06s0004g03160.t01 | S1Fa-like |
| g569.t1 | MYB | Vitvi04g00845.t01 | MYB_related | chr8.gff3_PROT_VIT_08s0040g02220.t01 | S1Fa-like |
| g5764.t1 | MYB | Vitvi04g01309.t01 | MYB_related | chr12.gff3_PROT_VIT_12s0057g00910.t01 | SAP |
| g6138.t1 | MYB | Vitvi05g00330.t01 | MYB_related | chr1.gff3_PROT_VIT_01s0010g03710.t01 | SBP |
| g682.t1 | MYB | Vitvi06g00245.t01 | MYB_related | chr1.gff3_PROT_VIT_01s0010g03910.t01 | SBP |
| g682.t2 | MYB | Vitvi06g00488.t01 | MYB_related | chr1.gff3_PROT_VIT_01s0011g00130.t01 | SBP |
| g8673.t1 | MYB | Vitvi06g00798.t01 | MYB_related | chr1.gff3_PROT_VIT_01s0026g01820.t01 | SBP |
| g10390.t1 | MYB_related | Vitvi07g00452.t01 | MYB_related | chr10.gff3_PROT_VIT_10s0003g00050.t01 | SBP |
| g10791.t1 | MYB_related | Vitvi07g01899.t01 | MYB_related | chr11.gff3_PROT_VIT_11s0065g00170.t01 | SBP |
| g10813.t1 | MYB_related | Vitvi07g01900.t01 | MYB_related | chr12.gff3_PROT_VIT_12s0028g03350.t01 | SBP |
| g11202.t1 | MYB_related | Vitvi07g02253.t01 | MYB_related | chr14.gff3_PROT_VIT_14s0068g01780.t01 | SBP |
| g11203.t1 | MYB_related | Vitvi07g02627.t01 | MYB_related | chr15.gff3_PROT_VIT_15s0021g02290.t01 | SBP |
| g11585.t1 | MYB_related | Vitvi07g02629.t01 | MYB_related | chr15.gff3_PROT_VIT_15s0021g02300.t01 | SBP |
| g12251.t1 | MYB_related | Vitvi08g01149.t01 | MYB_related | chr17.gff3_PROT_VIT_17s0000g01260.t01 | SBP |
| g1263.t1 | MYB_related | Vitvi08g01213.t01 | MYB_related | chr17.gff3_PROT_VIT_17s0000g05020.t01 | SBP |
| g12680.t1 | MYB_related | Vitvi08g01875.t01 | MYB_related | chr18.gff3_PROT_VIT_18s0122g00380.t01 | SBP |
| g13320.t1 | MYB_related | Vitvi08g02140.t01 | MYB_related | chr19.gff3_PROT_VIT_19s0014g02350.t01 | SBP |
| g13612.t1 | MYB_related | Vitvi09g00122.t01 | MYB_related | chr4.gff3_PROT_VIT_04s0210g00170.t01 | SBP |
| g13613.t1 | MYB_related | Vitvi09g00293.t01 | MYB_related | chr5.gff3_PROT_VIT_05s0020g00700.t01 | SBP |
| g13614.t1 | MYB_related | Vitvi09g00300.t01 | MYB_related | chr5.gff3_PROT_VIT_05s0020g02160.t01 | SBP |
| g13686.t1 | MYB_related | Vitvi10g01608.t01 | MYB_related | chr7.gff3_PROT_VIT_07s0005g02260.t01 | SBP |
| g13686.t2 | MYB_related | Vitvi10g01641.t01 | MYB_related | chr8.gff3_PROT_VIT_08s0007g06270.t01 | SBP |
| g1402.t1 | MYB_related | Vitvi11g00149.t01 | MYB_related | chr13_random.gff3_PROT_VIT_13s0067g02860.t01 | SRS |
| g14994.t1 | MYB_related | Vitvi11g00197.t01 | MYB_related | chr18.gff3_PROT_VIT_18s0001g13420.t01 | SRS |
| g15788.t1 | MYB_related | Vitvi11g00498.t01 | MYB_related | chr3.gff3_PROT_VIT_03s0038g00310.t01 | SRS |
| g15789.t1 | MYB_related | Vitvi12g00527.t01 | MYB_related | chr4.gff3_PROT_VIT_04s0023g02780.t01 | SRS |
| g15860.t1 | MYB_related | Vitvi12g00771.t01 | MYB_related | chr6.gff3_PROT_VIT_06s0009g03450.t01 | SRS |
| g1682.t1 | MYB_related | Vitvi13g00310.t01 | MYB_related | chr19.gff3_PROT_VIT_19s0014g00140.t01 | STAT |
| g16887.t1 | MYB_related | Vitvi13g00842.t01 | MYB_related | chr3.gff3_PROT_VIT_03s0038g00050.t01 | TALE |
| g17817.t1 | MYB_related | Vitvi13g02569.t01 | MYB_related | chr8.gff3_PROT_VIT_08s0105g00230.t01 | TALE |
| g20658.t1 | MYB_related | Vitvi14g00230.t01 | MYB_related | chr6.gff3_PROT_VIT_06s0009g00410.t01 | TALE |
| g20887.t1 | MYB_related | Vitvi15g00870.t01 | MYB_related | chr2.gff3_PROT_VIT_02s0025g00200.t01 | TALE |
| g20938.t1 | MYB_related | Vitvi15g01002.t01 | MYB_related | chr8.gff3_PROT_VIT_08s0007g01290.t01 | TALE |
| g21001.t1 | MYB_related | Vitvi15g01056.t01 | MYB_related | chr6.gff3_PROT_VIT_06s0004g02580.t01 | TALE |
| g21032.t1 | MYB_related | Vitvi15g01097.t01 | MYB_related | chr18.gff3_PROT_VIT_18s0001g13150.t01 | TALE |
| g21486.t1 | MYB_related | Vitvi16g00100.t01 | MYB_related | chr4.gff3_PROT_VIT_04s0023g01020.t01 | TALE |
| g21493.t1 | MYB_related | Vitvi16g00102.t01 | MYB_related | chr18.gff3_PROT_VIT_18s0001g13130.t01 | TALE |
| g22086.t2 | MYB_related | Vitvi16g00129.t01 | MYB_related | chr13.gff3_PROT_VIT_13s0019g02450.t01 | TALE |
| g22165.t1 | MYB_related | Vitvi16g00303.t01 | MYB_related | chr18.gff3_PROT_VIT_18s0001g11800.t01 | TALE |
| g22165.t2 | MYB_related | Vitvi16g00305.t01 | MYB_related | chr4.gff3_PROT_VIT_04s0008g00880.t01 | TALE |
| g23579.t1 | MYB_related | Vitvi16g01106.t01 | MYB_related | chr10_random.gff3_PROT_VIT_10s0116g00190.t01 | TALE |
| g23916.t1 | MYB_related | Vitvi16g01215.t01 | MYB_related | chr18.gff3_PROT_VIT_18s0001g08380.t01 | TALE |
| g23963.t1 | MYB_related | Vitvi16g01449.t01 | MYB_related | chr12.gff3_PROT_VIT_12s0059g01190.t01 | TALE |
| g24072.t1 | MYB_related | Vitvi16g01513.t01 | MYB_related | chr14.gff3_PROT_VIT_14s0060g01180.t01 | TALE |
| g24681.t1 | MYB_related | Vitvi16g01515.t01 | MYB_related | chr17.gff3_PROT_VIT_17s0000g08710.t01 | TALE |
| g24792.t1 | MYB_related | Vitvi16g01520.t01 | MYB_related | chr4.gff3_PROT_VIT_04s0008g06130.t01 | TALE |
| g24860.t2 | MYB_related | Vitvi16g01521.t01 | MYB_related | chr11.gff3_PROT_VIT_11s0118g00800.t01 | TALE |
| g24860.t3 | MYB_related | Vitvi16g01524.t01 | MYB_related | chr1.gff3_PROT_VIT_01s0127g00210.t01 | TALE |
| g25373.t1 | MYB_related | Vitvi16g01525.t01 | MYB_related | chr2.gff3_PROT_VIT_02s0025g04630.t01 | TALE |
| g25675.t1 | MYB_related | Vitvi17g00726.t01 | MYB_related | chr1.gff3_PROT_VIT_01s0011g02920.t01 | TCP |
| g25821.t1 | MYB_related | Vitvi18g00967.t01 | MYB_related | chr1.gff3_PROT_VIT_01s0026g02200.t01 | TCP |
| g2651.t1 | MYB_related | Vitvi18g00973.t01 | MYB_related | chr10.gff3_PROT_VIT_10s0003g00870.t01 | TCP |
| g26667.t1 | MYB_related | Vitvi18g02612.t01 | MYB_related | chr10.gff3_PROT_VIT_10s0003g03910.t01 | TCP |
| g2792.t1 | MYB_related | Vitvi18g02938.t01 | MYB_related | chr10.gff3_PROT_VIT_10s0042g00170.t01 | TCP |
| g27948.t1 | MYB_related | Vitvi18g02951.t01 | MYB_related | chr12.gff3_PROT_VIT_12s0028g02520.t01 | TCP |
| g27954.t1 | MYB_related | Vitvi19g00741.t01 | MYB_related | chr12.gff3_PROT_VIT_12s0035g00690.t01 | TCP |
| g27987.t1 | MYB_related | Vitvi19g00743.t01 | MYB_related | chr14.gff3_PROT_VIT_14s0068g00330.t01 | TCP |
| g28878.t1 | MYB_related | Vitvi19g02081.t01 | MYB_related | chr14.gff3_PROT_VIT_14s0068g01690.t01 | TCP |
| g29015.t1 | MYB_related | Vitvi01g00237.t01 | NAC | chr14.gff3_PROT_VIT_14s0083g00150.t01 | TCP |
| g29018.t1 | MYB_related | Vitvi01g00999.t01 | NAC | chr15.gff3_PROT_VIT_15s0048g01150.t01 | TCP |
| g29051.t1 | MYB_related | Vitvi01g01038.t01 | NAC | chr16.gff3_PROT_VIT_16s0022g02480.t01 | TCP |
| g29177.t1 | MYB_related | Vitvi01g01722.t01 | NAC | chr17.gff3_PROT_VIT_17s0000g04180.t01 | TCP |
| g30434.t1 | MYB_related | Vitvi02g00242.t01 | NAC | chr17.gff3_PROT_VIT_17s0000g06020.t01 | TCP |
| g30623.t1 | MYB_related | Vitvi02g00277.t01 | NAC | chr18.gff3_PROT_VIT_18s0117g00340.t01 | TCP |
| g30826.t1 | MYB_related | Vitvi02g00508.t01 | NAC | chr19.gff3_PROT_VIT_19s0014g01680.t01 | TCP |
| g30827.t1 | MYB_related | Vitvi02g00673.t01 | NAC | chr2.gff3_PROT_VIT_02s0025g04590.t01 | TCP |
| g30829.t1 | MYB_related | Vitvi02g00998.t01 | NAC | chr8.gff3_PROT_VIT_08s0040g01600.t01 | TCP |
| g30831.t1 | MYB_related | Vitvi03g00207.t01 | NAC | chr1.gff3_PROT_VIT_01s0010g02420.t01 | Trihelix |
| g30833.t1 | MYB_related | Vitvi04g00236.t01 | NAC | chr1.gff3_PROT_VIT_01s0150g00050.t01 | Trihelix |
| g30837.t1 | MYB_related | Vitvi04g00592.t01 | NAC | chr10.gff3_PROT_VIT_10s0003g01300.t01 | Trihelix |
| g30838.t1 | MYB_related | Vitvi04g00837.t01 | NAC | chr10.gff3_PROT_VIT_10s0042g00940.t01 | Trihelix |
| g30840.t1 | MYB_related | Vitvi04g01430.t01 | NAC | chr11.gff3_PROT_VIT_11s0016g04130.t01 | Trihelix |
| g30843.t1 | MYB_related | Vitvi04g01511.t01 | NAC | chr12.gff3_PROT_VIT_12s0028g02150.t01 | Trihelix |
| g30879.t1 | MYB_related | Vitvi04g01684.t01 | NAC | chr13.gff3_PROT_VIT_13s0067g01440.t01 | Trihelix |
| g31071.t1 | MYB_related | Vitvi04g01714.t01 | NAC | chr13.gff3_PROT_VIT_13s0074g00240.t01 | Trihelix |
| g31071.t2 | MYB_related | Vitvi06g00007.t01 | NAC | chr13.gff3_PROT_VIT_13s0074g00400.t01 | Trihelix |
| g31385.t1 | MYB_related | Vitvi06g00305.t01 | NAC | chr13.gff3_PROT_VIT_13s0084g00800.t01 | Trihelix |
| g31482.t1 | MYB_related | Vitvi06g01384.t01 | NAC | chr14.gff3_PROT_VIT_14s0030g01860.t01 | Trihelix |
| g31564.t1 | MYB_related | Vitvi06g01515.t01 | NAC | chr14.gff3_PROT_VIT_14s0171g00450.t01 | Trihelix |
| g31618.t1 | MYB_related | Vitvi06g01536.t01 | NAC | chr15.gff3_PROT_VIT_15s0021g01220.t01 | Trihelix |
| g31773.t1 | MYB_related | Vitvi06g01638.t01 | NAC | chr16.gff3_PROT_VIT_16s0022g02290.t01 | Trihelix |
| g31985.t1 | MYB_related | Vitvi06g01639.t01 | NAC | chr17.gff3_PROT_VIT_17s0000g00270.t01 | Trihelix |
| g3316.t1 | MYB_related | Vitvi06g01641.t01 | NAC | chr17.gff3_PROT_VIT_17s0000g01640.t01 | Trihelix |
| g3427.t1 | MYB_related | Vitvi06g01738.t01 | NAC | chr17.gff3_PROT_VIT_17s0000g10420.t01 | Trihelix |
| g34640.t1 | MYB_related | Vitvi07g00619.t01 | NAC | chr18.gff3_PROT_VIT_18s0072g00670.t01 | Trihelix |
| g35128.t1 | MYB_related | Vitvi07g01929.t01 | NAC | chr18.gff3_PROT_VIT_18s0072g00690.t01 | Trihelix |
| g36017.t1 | MYB_related | Vitvi07g02163.t01 | NAC | chr2.gff3_PROT_VIT_02s0025g03220.t01 | Trihelix |
| g36145.t1 | MYB_related | Vitvi08g01052.t01 | NAC | chr2.gff3_PROT_VIT_02s0087g00140.t01 | Trihelix |
| g36446.t1 | MYB_related | Vitvi08g01426.t01 | NAC | chr4.gff3_PROT_VIT_04s0008g01850.t01 | Trihelix |
| g36610.t1 | MYB_related | Vitvi08g01841.t01 | NAC | chr4.gff3_PROT_VIT_04s0008g02870.t01 | Trihelix |
| g36927.t1 | MYB_related | Vitvi08g01843.t01 | NAC | chr4.gff3_PROT_VIT_04s0044g00510.t01 | Trihelix |
| g37395.t1 | MYB_related | Vitvi08g02399.t01 | NAC | chr6.gff3_PROT_VIT_06s0004g01900.t01 | Trihelix |
| g37482.t1 | MYB_related | Vitvi09g01309.t01 | NAC | chr7.gff3_PROT_VIT_07s0005g06670.t01 | Trihelix |
| g37555.t1 | MYB_related | Vitvi10g00437.t01 | NAC | chr7.gff3_PROT_VIT_07s0031g02800.t01 | Trihelix |
| g37763.t1 | MYB_related | Vitvi10g00505.t01 | NAC | chr8.gff3_PROT_VIT_08s0007g03820.t01 | Trihelix |
| g37766.t1 | MYB_related | Vitvi10g00507.t01 | NAC | chr8.gff3_PROT_VIT_08s0007g04160.t01 | Trihelix |
| g37843.t1 | MYB_related | Vitvi11g00241.t01 | NAC | chr8.gff3_PROT_VIT_08s0007g04180.t01 | Trihelix |
| g3810.t1 | MYB_related | Vitvi12g00076.t01 | NAC | chr8.gff3_PROT_VIT_08s0058g00200.t01 | Trihelix |
| g38222.t1 | MYB_related | Vitvi12g00255.t01 | NAC | chr8.gff3_PROT_VIT_08s0105g00400.t01 | Trihelix |
| g38260.t1 | MYB_related | Vitvi12g01655.t01 | NAC | chr9.gff3_PROT_VIT_09s0002g00720.t01 | Trihelix |
| g38310.t1 | MYB_related | Vitvi12g02150.t01 | NAC | chr9.gff3_PROT_VIT_09s0002g04700.t01 | Trihelix |
| g5062.t1 | MYB_related | Vitvi13g00698.t01 | NAC | chrUn.gff3_PROT_VIT_00s0204g00020.t01 | Trihelix |
| g5857.t1 | MYB_related | Vitvi13g00699.t01 | NAC | chrUn.gff3_PROT_VIT_00s0259g00210.t01 | Trihelix |
| g5966.t1 | MYB_related | Vitvi13g01073.t01 | NAC | chrUn.gff3_PROT_VIT_00s0956g00030.t01 | Trihelix |
| g6114.t1 | MYB_related | Vitvi14g01499.t01 | NAC | chr10.gff3_PROT_VIT_10s0003g00500.t01 | VOZ |
| g6196.t1 | MYB_related | Vitvi14g01678.t01 | NAC | chr12.gff3_PROT_VIT_12s0028g02670.t01 | VOZ |
| g7947.t1 | MYB_related | Vitvi14g01963.t01 | NAC | chr1.gff3_PROT_VIT_01s0026g01720.t01 | Whirly |
| g7954.t1 | MYB_related | Vitvi14g01985.t01 | NAC | chr18.gff3_PROT_VIT_18s0089g00900.t01 | Whirly |
| g815.t1 | MYB_related | Vitvi15g00889.t01 | NAC | chr6.gff3_PROT_VIT_06s0004g03780.t01 | WOX |
| g815.t2 | MYB_related | Vitvi15g01539.t01 | NAC | chr18.gff3_PROT_VIT_18s0001g10160.t01 | WOX |
| g821.t1 | MYB_related | Vitvi15g01540.t01 | NAC | chr17.gff3_PROT_VIT_17s0000g02460.t01 | WOX |
| g8536.t1 | MYB_related | Vitvi15g01541.t01 | NAC | chr13.gff3_PROT_VIT_13s0019g03460.t01 | WOX |
| g8695.t1 | MYB_related | Vitvi15g01542.t01 | NAC | chr4.gff3_PROT_VIT_04s0023g03310.t01 | WOX |
| g8701.t1 | MYB_related | Vitvi15g01543.t01 | NAC | chr1.gff3_PROT_VIT_01s0011g05020.t01 | WOX |
| g9018.t1 | MYB_related | Vitvi15g01544.t01 | NAC | chr10.gff3_PROT_VIT_10s0003g00560.t01 | WOX |
| g10568.t1 | NAC | Vitvi15g01545.t01 | NAC | chr8.gff3_PROT_VIT_08s0056g01200.t01 | WOX |
| g10719.t1 | NAC | Vitvi15g01546.t01 | NAC | chr18.gff3_PROT_VIT_18s0122g01140.t01 | WOX |
| g10878.t1 | NAC | Vitvi16g00716.t01 | NAC | chr4.gff3_PROT_VIT_04s0044g00150.t01 | WOX |
| g11758.t1 | NAC | Vitvi16g01327.t01 | NAC | chr4.gff3_PROT_VIT_04s0044g00160.t01 | WOX |
| g12584.t1 | NAC | Vitvi17g00066.t01 | NAC | chr1.gff3_PROT_VIT_01s0010g03930.t01 | WRKY |
| g1285.t1 | NAC | Vitvi17g00316.t01 | NAC | chr1.gff3_PROT_VIT_01s0011g00720.t01 | WRKY |
| g13434.t1 | NAC | Vitvi17g00622.t01 | NAC | chr1.gff3_PROT_VIT_01s0026g01730.t01 | WRKY |
| g14440.t1 | NAC | Vitvi17g00685.t01 | NAC | chr10.gff3_PROT_VIT_10s0003g01600.t01 | WRKY |
| g14443.t1 | NAC | Vitvi17g01227.t01 | NAC | chr10.gff3_PROT_VIT_10s0003g02810.t01 | WRKY |
| g14444.t1 | NAC | Vitvi18g00052.t01 | NAC | chr10.gff3_PROT_VIT_10s0003g05740.t01 | WRKY |
| g14445.t1 | NAC | Vitvi18g00250.t01 | NAC | chr10.gff3_PROT_VIT_10s0116g01200.t01 | WRKY |
| g14446.t1 | NAC | Vitvi18g00761.t01 | NAC | chr11.gff3_PROT_VIT_11s0037g00150.t01 | WRKY |
| g14449.t1 | NAC | Vitvi18g01520.t01 | NAC | chr11.gff3_PROT_VIT_11s0052g00450.t01 | WRKY |
| g14620.t1 | NAC | Vitvi18g01684.t01 | NAC | chr12.gff3_PROT_VIT_12s0028g00270.t01 | WRKY |
| g14708.t1 | NAC | Vitvi18g02011.t01 | NAC | chr12.gff3_PROT_VIT_12s0055g00340.t01 | WRKY |
| g1500.t1 | NAC | Vitvi18g02404.t01 | NAC | chr12.gff3_PROT_VIT_12s0057g00550.t01 | WRKY |
| g15249.t1 | NAC | Vitvi18g02732.t01 | NAC | chr12.gff3_PROT_VIT_12s0059g00880.t01 | WRKY |
| g15250.t1 | NAC | Vitvi19g00188.t01 | NAC | chr13.gff3_PROT_VIT_13s0067g03130.t01 | WRKY |
| g15362.t1 | NAC | Vitvi19g00270.t01 | NAC | chr13.gff3_PROT_VIT_13s0067g03140.t01 | WRKY |
| g15650.t1 | NAC | Vitvi19g00271.t01 | NAC | chr14.gff3_PROT_VIT_14s0068g01770.t01 | WRKY |
| g15690.t1 | NAC | Vitvi19g01484.t01 | NAC | chr14.gff3_PROT_VIT_14s0081g00560.t01 | WRKY |
| g15945.t1 | NAC | Vitvi19g01561.t01 | NAC | chr14.gff3_PROT_VIT_14s0108g00120.t01 | WRKY |
| g16061.t1 | NAC | Vitvi19g01564.t01 | NAC | chr14.gff3_PROT_VIT_14s0108g01280.t01 | WRKY |
| g16255.t1 | NAC | Vitvi19g01566.t01 | NAC | chr15.gff3_PROT_VIT_15s0021g01310.t01 | WRKY |
| g16276.t1 | NAC | Vitvi19g01647.t01 | NAC | chr15.gff3_PROT_VIT_15s0046g01140.t01 | WRKY |
| g16322.t1 | NAC | Vitvi19g01751.t01 | NAC | chr15.gff3_PROT_VIT_15s0046g02150.t01 | WRKY |
| g16322.t2 | NAC | Vitvi19g01778.t01 | NAC | chr15.gff3_PROT_VIT_15s0046g02190.t01 | WRKY |
| g16349.t1 | NAC | Vitvi19g02272.t01 | NAC | chr16.gff3_PROT_VIT_16s0050g01480.t01 | WRKY |
| g16397.t1 | NAC | Vitvi19g02273.t01 | NAC | chr16.gff3_PROT_VIT_16s0050g02510.t01 | WRKY |
| g17320.t1 | NAC | Vitvi13g00049.t01 | NF-X1 | chr17.gff3_PROT_VIT_17s0000g01280.t01 | WRKY |
| g17767.t1 | NAC | Vitvi17g00161.t01 | NF-X1 | chr17.gff3_PROT_VIT_17s0000g05810.t01 | WRKY |
| g17769.t1 | NAC | Vitvi19g00036.t01 | NF-X1 | chr18.gff3_PROT_VIT_18s0001g10030.t01 | WRKY |
| g17845.t1 | NAC | Vitvi06g00224.t01 | NF-YA | chr19.gff3_PROT_VIT_19s0015g01870.t01 | WRKY |
| g18085.t1 | NAC | Vitvi08g00292.t01 | NF-YA | chr19.gff3_PROT_VIT_19s0090g00840.t01 | WRKY |
| g18618.t1 | NAC | Vitvi08g01883.t01 | NF-YA | chr19.gff3_PROT_VIT_19s0090g01720.t01 | WRKY |
| g18646.t1 | NAC | Vitvi09g00133.t01 | NF-YA | chr1_random.gff3_PROT_VIT_01s0011g00220.t01 | WRKY |
| g19064.t1 | NAC | Vitvi10g00911.t01 | NF-YA | chr2.gff3_PROT_VIT_02s0025g00420.t01 | WRKY |
| g19065.t1 | NAC | Vitvi11g00109.t01 | NF-YA | chr2.gff3_PROT_VIT_02s0025g01280.t01 | WRKY |
| g19281.t1 | NAC | Vitvi13g01729.t01 | NF-YA | chr2.gff3_PROT_VIT_02s0154g00210.t01 | WRKY |
| g19913.t1 | NAC | Vitvi01g01464.t01 | NF-YB | chr4.gff3_PROT_VIT_04s0008g01470.t01 | WRKY |
| g19992.t1 | NAC | Vitvi01g01468.t01 | NF-YB | chr4.gff3_PROT_VIT_04s0008g05750.t01 | WRKY |
| g20023.t1 | NAC | Vitvi05g00299.t01 | NF-YB | chr4.gff3_PROT_VIT_04s0008g05760.t01 | WRKY |
| g20094.t1 | NAC | Vitvi06g00351.t01 | NF-YB | chr4.gff3_PROT_VIT_04s0008g06600.t01 | WRKY |
| g20095.t1 | NAC | Vitvi06g01492.t01 | NF-YB | chr4.gff3_PROT_VIT_04s0023g00470.t01 | WRKY |
| g20192.t1 | NAC | Vitvi07g00273.t01 | NF-YB | chr4.gff3_PROT_VIT_04s0069g00920.t01 | WRKY |
| g21200.t1 | NAC | Vitvi07g01823.t01 | NF-YB | chr4.gff3_PROT_VIT_04s0069g00970.t01 | WRKY |
| g21236.t1 | NAC | Vitvi08g01154.t01 | NF-YB | chr4.gff3_PROT_VIT_04s0069g00980.t01 | WRKY |
| g21613.t1 | NAC | Vitvi10g00251.t01 | NF-YB | chr5.gff3_PROT_VIT_05s0077g00730.t01 | WRKY |
| g22605.t1 | NAC | Vitvi10g02273.t01 | NF-YB | chr6.gff3_PROT_VIT_06s0004g00230.t01 | WRKY |
| g23242.t1 | NAC | Vitvi10g02311.t01 | NF-YB | chr6.gff3_PROT_VIT_06s0004g07500.t01 | WRKY |
| g24131.t1 | NAC | Vitvi12g00719.t01 | NF-YB | chr7.gff3_PROT_VIT_07s0005g01520.t01 | WRKY |
| g24727.t1 | NAC | Vitvi13g00535.t01 | NF-YB | chr7.gff3_PROT_VIT_07s0005g01710.t01 | WRKY |
| g25115.t1 | NAC | Vitvi14g00217.t01 | NF-YB | chr7.gff3_PROT_VIT_07s0005g02570.t01 | WRKY |
| g25401.t1 | NAC | Vitvi17g01421.t01 | NF-YB | chr7.gff3_PROT_VIT_07s0031g00080.t01 | WRKY |
| g25548.t1 | NAC | Vitvi19g00673.t01 | NF-YB | chr7.gff3_PROT_VIT_07s0031g01710.t01 | WRKY |
| g26653.t1 | NAC | Vitvi19g00686.t01 | NF-YB | chr7.gff3_PROT_VIT_07s0031g01840.t01 | WRKY |
| g26654.t1 | NAC | Vitvi19g02067.t01 | NF-YB | chr7.gff3_PROT_VIT_07s0141g00680.t01 | WRKY |
| g26655.t1 | NAC | Vitvi01g01642.t01 | NF-YC | chr8.gff3_PROT_VIT_08s0007g00570.t01 | WRKY |
| g26656.t1 | NAC | Vitvi02g00347.t01 | NF-YC | chr8.gff3_PROT_VIT_08s0040g03070.t01 | WRKY |
| g26656.t2 | NAC | Vitvi05g00435.t01 | NF-YC | chr8.gff3_PROT_VIT_08s0058g00690.t01 | WRKY |
| g26657.t1 | NAC | Vitvi06g01109.t01 | NF-YC | chr8.gff3_PROT_VIT_08s0058g01390.t01 | WRKY |
| g26659.t1 | NAC | Vitvi06g01659.t01 | NF-YC | chr9.gff3_PROT_VIT_09s0018g00240.t01 | WRKY |
| g26660.t1 | NAC | Vitvi13g01402.t01 | NF-YC | chrUn.gff3_PROT_VIT_00s0463g00010.t01 | WRKY |
| g26695.t1 | NAC | Vitvi14g00264.t01 | NF-YC | chr1.gff3_PROT_VIT_01s0011g00140.t01 | YABBY |
| g26843.t1 | NAC | Vitvi14g02861.t01 | NF-YC | chr1.gff3_PROT_VIT_01s0127g00330.t01 | YABBY |
| g27076.t1 | NAC | Vitvi17g00088.t01 | NF-YC | chr11.gff3_PROT_VIT_11s0016g05590.t01 | YABBY |
| g27099.t1 | NAC | Vitvi19g01705.t01 | NF-YC | chr15.gff3_PROT_VIT_15s0048g00550.t01 | YABBY |
| g2741.t1 | NAC | Vitvi02g00180.t01 | Nin-like | chr2.gff3_PROT_VIT_02s0154g00070.t01 | YABBY |
| g28110.t1 | NAC | Vitvi03g00231.t01 | Nin-like | chr6.gff3_PROT_VIT_06s0009g00880.t01 | YABBY |
| g29052.t1 | NAC | Vitvi04g00143.t01 | Nin-like | chr8.gff3_PROT_VIT_08s0032g01110.t01 | YABBY |
| g29218.t1 | NAC | Vitvi04g01569.t01 | Nin-like | chr1.gff3_PROT_VIT_01s0011g06360.t01 | ZF-HD |
| g29331.t1 | NAC | Vitvi05g01560.t01 | Nin-like | chr1.gff3_PROT_VIT_01s0026g02450.t01 | ZF-HD |
| g29461.t1 | NAC | Vitvi12g00055.t01 | Nin-like | chr1.gff3_PROT_VIT_01s0026g02460.t01 | ZF-HD |
| g29998.t1 | NAC | Vitvi16g01402.t01 | Nin-like | chr12.gff3_PROT_VIT_12s0035g01880.t01 | ZF-HD |
| g30383.t1 | NAC | Vitvi17g00605.t01 | Nin-like | chr14.gff3_PROT_VIT_14s0108g00750.t01 | ZF-HD |
| g31134.t1 | NAC | Vitvi18g02488.t01 | Nin-like | chr14.gff3_PROT_VIT_14s0108g00760.t01 | ZF-HD |
| g31842.t1 | NAC | Vitvi19g00320.t01 | NZZ/SPL | chr14.gff3_PROT_VIT_14s0108g00810.t01 | ZF-HD |
| g3256.t1 | NAC | Vitvi19g01844.t01 | NZZ/SPL | chr17.gff3_PROT_VIT_17s0000g06200.t01 | ZF-HD |
| g32932.t1 | NAC | Vitvi01g00244.t01 | RAV | chr17_random.gff3_PROT_VIT_17s0000g00810.t01 | ZF-HD |
| g32936.t1 | NAC | Vitvi14g01248.t01 | RAV | chr18.gff3_PROT_VIT_18s0001g07140.t01 | ZF-HD |
| g34404.t1 | NAC | Vitvi02g01775.t01 | RAV | chr18.gff3_PROT_VIT_18s0001g12580.t01 | ZF-HD |
| g3513.t1 | NAC | Vitvi11g00678.t01 | RAV | chr18.gff3_PROT_VIT_18s0117g00270.t01 | ZF-HD |
| g35180.t1 | NAC | Vitvi06g01662.t01 | S1Fa-like | chr4.gff3_PROT_VIT_04s0023g01880.t01 | ZF-HD |
| g35509.t1 | NAC | Vitvi08g02161.t01 | S1Fa-like | chr8.gff3_PROT_VIT_08s0056g01130.t01 | ZF-HD |
| g35514.t1 | NAC | Vitvi12g00707.t01 | SAP | chrUn.gff3_PROT_VIT_00s0199g00250.t01 | ZF-HD |
| g35515.t1 | NAC | Vitvi01g01660.t01 | SBP |  |  |
| g35516.t1 | NAC | Vitvi01g01678.t01 | SBP |  |  |
| g36187.t1 | NAC | Vitvi01g01837.t01 | SBP |  |  |
| g36314.t1 | NAC | Vitvi01g02117.t01 | SBP |  |  |
| g37143.t1 | NAC | Vitvi04g01556.t01 | SBP |  |  |
| g38214.t1 | NAC | Vitvi05g00245.t01 | SBP |  |  |
| g4719.t1 | NAC | Vitvi05g00376.t01 | SBP |  |  |
| g6554.t1 | NAC | Vitvi07g00491.t01 | SBP |  |  |
| g7268.t1 | NAC | Vitvi08g01720.t01 | SBP |  |  |
| g763.t1 | NAC | Vitvi10g00481.t01 | SBP |  |  |
| g8089.t1 | NAC | Vitvi11g00909.t01 | SBP |  |  |
| g9136.t1 | NAC | Vitvi12g00280.t01 | SBP |  |  |
| g9210.t1 | NAC | Vitvi14g01524.t01 | SBP |  |  |
| g9597.t1 | NAC | Vitvi15g00619.t01 | SBP |  |  |
| g26805.t1 | NF-X1 | Vitvi15g00620.t01 | SBP |  |  |
| g29633.t1 | NF-X1 | Vitvi17g00100.t01 | SBP |  |  |
| g14458.t1 | NF-YA | Vitvi17g00473.t01 | SBP |  |  |
| g17823.t1 | NF-YA | Vitvi18g00027.t01 | SBP |  |  |
| g18396.t1 | NF-YA | Vitvi19g00200.t01 | SBP |  |  |
| g22319.t1 | NF-YA | Vitvi02g00197.t01 | SRS |  |  |
| g22319.t2 | NF-YA | Vitvi03g00024.t01 | SRS |  |  |
| g22867.t1 | NF-YA | Vitvi04g01399.t01 | SRS |  |  |
| g31060.t1 | NF-YA | Vitvi06g01239.t01 | SRS |  |  |
| g32010.t1 | NF-YA | Vitvi13g01413.t01 | SRS |  |  |
| g1472.t1 | NF-YB | Vitvi18g01041.t01 | SRS |  |  |
| g16301.t1 | NF-YB | Vitvi19g00013.t01 | STAT |  |  |
| g17072.t1 | NF-YB | Vitvi03g00004.t01 | TALE |  |  |
| g2029.t1 | NF-YB | Vitvi08g00633.t01 | TALE |  |  |
| g2032.t1 | NF-YB | Vitvi06g00918.t01 | TALE |  |  |
| g20386.t1 | NF-YB | Vitvi02g00016.t01 | TALE |  |  |
| g22524.t1 | NF-YB | Vitvi08g01274.t01 | TALE |  |  |
| g24796.t1 | NF-YB | Vitvi06g00248.t01 | TALE |  |  |
| g24796.t2 | NF-YB | Vitvi18g00902.t01 | TALE |  |  |
| g24796.t3 | NF-YB | Vitvi18g01016.t01 | TALE |  |  |
| g26037.t1 | NF-YB | Vitvi04g01210.t01 | TALE |  |  |
| g2622.t1 | NF-YB | Vitvi18g02768.t01 | TALE |  |  |
| g3078.t1 | NF-YB | Vitvi13g00366.t01 | TALE |  |  |
| g31668.t1 | NF-YB | Vitvi04g00072.t01 | TALE |  |  |
| g31681.t1 | NF-YB | Vitvi10g00124.t01 | TALE |  |  |
| g31682.t1 | NF-YB | Vitvi18g00602.t01 | TALE |  |  |
| g31889.t1 | NF-YB | Vitvi12g00424.t01 | TALE |  |  |
| g33192.t1 | NF-YB | Vitvi14g00093.t01 | TALE |  |  |
| g34518.t1 | NF-YB | Vitvi17g00850.t01 | TALE |  |  |
| g34520.t1 | NF-YB | Vitvi04g00546.t01 | TALE |  |  |
| g37268.t1 | NF-YB | Vitvi11g00601.t01 | TALE |  |  |
| g11852.t1 | NF-YC | Vitvi01g00694.t01 | TALE |  |  |
| g16382.t1 | NF-YC | Vitvi02g00425.t01 | TALE |  |  |
| g16643.t1 | NF-YC | Vitvi01g00228.t01 | TCP |  |  |
| g21310.t1 | NF-YC | Vitvi01g00980.t01 | TCP |  |  |
| g21937.t1 | NF-YC | Vitvi02g00421.t01 | TCP |  |  |
| g2888.t1 | NF-YC | Vitvi06g01646.t01 | TCP |  |  |
| g30575.t1 | NF-YC | Vitvi08g01011.t01 | TCP |  |  |
| g31770.t1 | NF-YC | Vitvi10g00540.t01 | TCP |  |  |
| g31838.t1 | NF-YC | Vitvi10g00838.t01 | TCP |  |  |
| g35640.t1 | NF-YC | Vitvi10g01466.t01 | TCP |  |  |
| g13459.t1 | Nin-like | Vitvi12g00219.t01 | TCP |  |  |
| g18495.t1 | Nin-like | Vitvi12g02006.t01 | TCP |  |  |
| g18495.t2 | Nin-like | Vitvi13g02208.t01 | TCP |  |  |
| g23837.t1 | Nin-like | Vitvi14g01253.t01 | TCP |  |  |
| g28060.t1 | Nin-like | Vitvi14g01398.t01 | TCP |  |  |
| g2917.t1 | Nin-like | Vitvi14g01519.t01 | TCP |  |  |
| g29370.t1 | Nin-like | Vitvi15g00765.t01 | TCP |  |  |
| g3409.t1 | Nin-like | Vitvi16g01833.t01 | TCP |  |  |
| g34366.t1 | Nin-like | Vitvi16g01846.t01 | TCP |  |  |
| g576.t1 | Nin-like | Vitvi17g00374.t01 | TCP |  |  |
| g28365.t1 | NZZ/SPL | Vitvi17g00495.t01 | TCP |  |  |
| g16056.t1 | RAV | Vitvi17g00581.t01 | TCP |  |  |
| g18212.t1 | RAV | Vitvi18g01875.t01 | TCP |  |  |
| g7473.t1 | RAV | Vitvi19g00140.t01 | TCP |  |  |
| g11825.t1 | RAV | Vitvi01g01477.t01 | Trihelix |  |  |
| g2730.t1 | S1Fa-like | Vitvi01g01533.t01 | Trihelix |  |  |
| g31856.t1 | S1Fa-like | Vitvi01g02298.t01 | Trihelix |  |  |
| g22513.t1 | SAP | Vitvi02g00291.t01 | Trihelix |  |  |
| g10762.t1 | SBP | Vitvi02g01203.t01 | Trihelix |  |  |
| g10837.t1 | SBP | Vitvi02g01753.t01 | Trihelix |  |  |
| g13001.t1 | SBP | Vitvi04g00253.t01 | Trihelix |  |  |
| g15125.t1 | SBP | Vitvi04g01604.t01 | Trihelix |  |  |
| g15348.t1 | SBP | Vitvi04g01808.t01 | Trihelix |  |  |
| g17639.t1 | SBP | Vitvi06g00200.t01 | Trihelix |  |  |
| g18478.t1 | SBP | Vitvi07g00941.t01 | Trihelix |  |  |
| g21409.t1 | SBP | Vitvi07g01957.t01 | Trihelix |  |  |
| g23138.t1 | SBP | Vitvi08g00658.t01 | Trihelix |  |  |
| g25089.t1 | SBP | Vitvi08g00730.t01 | Trihelix |  |  |
| g2562.t1 | SBP | Vitvi08g01505.t01 | Trihelix |  |  |
| g25766.t1 | SBP | Vitvi08g01538.t01 | Trihelix |  |  |
| g28778.t1 | SBP | Vitvi08g01540.t01 | Trihelix |  |  |
| g29249.t1 | SBP | Vitvi09g00393.t01 | Trihelix |  |  |
| g29974.t1 | SBP | Vitvi09g01306.t01 | Trihelix |  |  |
| g33761.t1 | SBP | Vitvi09g01496.t01 | Trihelix |  |  |
| g33762.t1 | SBP | Vitvi10g00254.t01 | Trihelix |  |  |
| g33762.t2 | SBP | Vitvi10g00584.t01 | Trihelix |  |  |
| g34437.t1 | SBP | Vitvi10g01388.t01 | Trihelix |  |  |
| g36888.t1 | SBP | Vitvi10g02271.t01 | Trihelix |  |  |
| g5654.t1 | SBP | Vitvi11g00354.t01 | Trihelix |  |  |
| g13506.t1 | SRS | Vitvi12g00182.t01 | Trihelix |  |  |
| g15160.t1 | SRS | Vitvi13g00084.t01 | Trihelix |  |  |
| g25578.t1 | SRS | Vitvi13g00728.t01 | Trihelix |  |  |
| g28946.t1 | SRS | Vitvi13g00740.t01 | Trihelix |  |  |
| g899.t1 | SRS | Vitvi13g01340.t01 | Trihelix |  |  |
| g33924.t1 | STAT | Vitvi14g00433.t01 | Trihelix |  |  |
| g15144.t1 | TALE | Vitvi14g01602.t01 | Trihelix |  |  |
| g20251.t1 | TALE | Vitvi15g00532.t01 | Trihelix |  |  |
| g33945.t1 | TALE | Vitvi16g00887.t01 | Trihelix |  |  |
| g27216.t1 | TALE | Vitvi17g00029.t01 | Trihelix |  |  |
| g13710.t1 | TALE | Vitvi17g00132.t01 | Trihelix |  |  |
| g31785.t1 | TALE | Vitvi17g01050.t01 | Trihelix |  |  |
| g750.t1 | TALE | Vitvi18g01649.t01 | Trihelix |  |  |
| g876.t1 | TALE | Vitvi18g01652.t01 | Trihelix |  |  |
| g875.t1 | TALE | Vitvi10g00515.t01 | VOZ |  |  |
| g21114.t1 | TALE | Vitvi12g00235.t01 | VOZ |  |  |
| g3314.t1 | TALE | Vitvi01g00938.t01 | Whirly |  |  |
| g31510.t1 | TALE | Vitvi18g02389.t01 | Whirly |  |  |
| g17914.t1 | TALE | Vitvi06g00380.t01 | WOX |  |  |
| g23471.t1 | TALE | Vitvi18g00757.t01 | WOX |  |  |
| g11685.t1 | TALE | Vitvi17g00216.t01 | WOX |  |  |
| g28385.t1 | TALE | Vitvi06g00445.t01 | WOX |  |  |
| g22760.t2 | TALE | Vitvi04g01449.t01 | WOX |  |  |
| g22760.t1 | TALE | Vitvi13g02039.t01 | WOX |  |  |
| g12524.t1 | TALE | Vitvi01g00427.t01 | WOX |  |  |
| g26129.t1 | TALE | Vitvi10g00519.t01 | WOX |  |  |
| g266.t1 | TALE | Vitvi08g00108.t01 | WOX |  |  |
| g10503.t1 | TCP | Vitvi18g00084.t01 | WOX |  |  |
| g14431.t1 | TCP | Vitvi04g01578.t01 | WOX |  |  |
| g16067.t1 | TCP | Vitvi04g01579.t01 | WOX |  |  |
| g16449.t1 | TCP | Vitvi01g00940.t01 | WRKY |  |  |
| g18213.t1 | TCP | Vitvi01g01680.t01 | WRKY |  |  |
| g22074.t1 | TCP | Vitvi01g01844.t01 | WRKY |  |  |
| g23724.t1 | TCP | Vitvi01g02157.t01 | WRKY |  |  |
| g26018.t1 | TCP | Vitvi02g00039.t01 | WRKY |  |  |
| g262.t1 | TCP | Vitvi02g00114.t01 | WRKY |  |  |
| g2795.t2 | TCP | Vitvi02g01435.t01 | WRKY |  |  |
| g29980.t1 | TCP | Vitvi02g01847.t01 | WRKY |  |  |
| g30379.t1 | TCP | Vitvi04g00133.t01 | WRKY |  |  |
| g3218.t1 | TCP | Vitvi04g00510.t01 | WRKY |  |  |
| g32221.t1 | TCP | Vitvi04g00511.t01 | WRKY |  |  |
| g32985.t1 | TCP | Vitvi04g00596.t01 | WRKY |  |  |
| g34644.t1 | TCP | Vitvi04g00756.t01 | WRKY |  |  |
| g34724.t1 | TCP | Vitvi04g00760.t01 | WRKY |  |  |
| g34783.t1 | TCP | Vitvi04g01163.t01 | WRKY |  |  |
| g553.t1 | TCP | Vitvi04g01985.t01 | WRKY |  |  |
| g14497.t1 | Trihelix | Vitvi05g00145.t01 | WRKY |  |  |
| g14689.t1 | Trihelix | Vitvi06g00741.t01 | WRKY |  |  |
| g15135.t1 | Trihelix | Vitvi06g01574.t01 | WRKY |  |  |
| g15135.t2 | Trihelix | Vitvi07g00026.t01 | WRKY |  |  |
| g15189.t1 | Trihelix | Vitvi07g00421.t01 | WRKY |  |  |
| g15501.t1 | Trihelix | Vitvi07g00434.t01 | WRKY |  |  |
| g16489.t1 | Trihelix | Vitvi07g00523.t01 | WRKY |  |  |
| g17159.t1 | Trihelix | Vitvi07g01694.t01 | WRKY |  |  |
| g17220.t1 | Trihelix | Vitvi07g01847.t01 | WRKY |  |  |
| g17222.t1 | Trihelix | Vitvi07g01860.t01 | WRKY |  |  |
| g17413.t1 | Trihelix | Vitvi08g00793.t01 | WRKY |  |  |
| g17450.t1 | Trihelix | Vitvi08g00868.t01 | WRKY |  |  |
| g17453.t1 | Trihelix | Vitvi08g01134.t01 | WRKY |  |  |
| g18549.t1 | Trihelix | Vitvi08g01221.t01 | WRKY |  |  |
| g20271.t1 | Trihelix | Vitvi09g01122.t01 | WRKY |  |  |
| g2030.t1 | Trihelix | Vitvi10g00063.t01 | WRKY |  |  |
| g21249.t1 | Trihelix | Vitvi10g00270.t01 | WRKY |  |  |
| g21249.t2 | Trihelix | Vitvi10g00618.t01 | WRKY |  |  |
| g21447.t1 | Trihelix | Vitvi10g00732.t01 | WRKY |  |  |
| g21474.t1 | Trihelix | Vitvi10g01078.t01 | WRKY |  |  |
| g21806.t1 | Trihelix | Vitvi11g00694.t01 | WRKY |  |  |
| g2222.t1 | Trihelix | Vitvi11g01188.t01 | WRKY |  |  |
| g22383.t1 | Trihelix | Vitvi12g00048.t01 | WRKY |  |  |
| g23283.t1 | Trihelix | Vitvi12g00148.t01 | WRKY |  |  |
| g26498.t1 | Trihelix | Vitvi12g00388.t01 | WRKY |  |  |
| g29198.t1 | Trihelix | Vitvi12g00664.t01 | WRKY |  |  |
| g29564.t1 | Trihelix | Vitvi12g01676.t01 | WRKY |  |  |
| g29895.t1 | Trihelix | Vitvi13g00189.t01 | WRKY |  |  |
| g30851.t1 | Trihelix | Vitvi13g01916.t01 | WRKY |  |  |
| g31708.t1 | Trihelix | Vitvi14g00540.t01 | WRKY |  |  |
| g3173.t1 | Trihelix | Vitvi14g01523.t01 | WRKY |  |  |
| g33437.t1 | Trihelix | Vitvi14g01907.t01 | WRKY |  |  |
| g3425.t1 | Trihelix | Vitvi14g02007.t01 | WRKY |  |  |
| g34468.t1 | Trihelix | Vitvi15g00539.t01 | WRKY |  |  |
| g34468.t2 | Trihelix | Vitvi15g01003.t01 | WRKY |  |  |
| g34506.t1 | Trihelix | Vitvi15g01087.t01 | WRKY |  |  |
| g3529.t1 | Trihelix | Vitvi15g01090.t01 | WRKY |  |  |
| g353.t1 | Trihelix | Vitvi16g01132.t01 | WRKY |  |  |
| g36476.t1 | Trihelix | Vitvi16g01133.t01 | WRKY |  |  |
| g29208.t1 | VOZ | Vitvi16g01213.t01 | WRKY |  |  |
| g3237.t1 | VOZ | Vitvi17g00102.t01 | WRKY |  |  |
| g34054.t1 | Whirly | Vitvi17g00556.t01 | WRKY |  |  |
| g3640.t1 | Whirly | Vitvi18g00742.t01 | WRKY |  |  |
| g18078.t1 | WOX | Vitvi19g00530.t01 | WRKY |  |  |
| g25528.t1 | WOX | Vitvi19g00617.t01 | WRKY |  |  |
| g25254.t1 | WOX | Vitvi19g00927.t01 | WRKY |  |  |
| g24508.t1 | WOX | Vitvi01g00013.t01 | YABBY |  |  |
| g16421.t1 | WOX | Vitvi01g00703.t01 | YABBY |  |  |
| g29730.t1 | WOX | Vitvi02g00510.t01 | YABBY |  |  |
| g34377.t1 | WOX | Vitvi06g00972.t01 | YABBY |  |  |
| g18508.t1 | WOX | Vitvi08g00274.t01 | YABBY |  |  |
| g18509.t1 | WOX | Vitvi11g00492.t01 | YABBY |  |  |
| g19639.t1 | WOX | Vitvi15g00708.t01 | YABBY |  |  |
| g17085.t1 | WOX | Vitvi01g00545.t01 | ZF-HD |  |  |
| g10779.t1 | WRKY | Vitvi01g01012.t01 | ZF-HD |  |  |
| g1089.t1 | WRKY | Vitvi01g01013.t01 | ZF-HD |  |  |
| g11899.t1 | WRKY | Vitvi04g01304.t01 | ZF-HD |  |  |
| g12148.t1 | WRKY | Vitvi07g01465.t01 | ZF-HD |  |  |
| g12956.t1 | WRKY | Vitvi07g02032.t01 | ZF-HD |  |  |
| g13465.t1 | WRKY | Vitvi08g00100.t01 | ZF-HD |  |  |
| g14157.t1 | WRKY | Vitvi12g01999.t01 | ZF-HD |  |  |
| g14244.t1 | WRKY | Vitvi12g02000.t01 | ZF-HD |  |  |
| g14996.t1 | WRKY | Vitvi12g02135.t01 | ZF-HD |  |  |
| g15094.t1 | WRKY | Vitvi14g01955.t01 | ZF-HD |  |  |
| g15127.t1 | WRKY | Vitvi14g01956.t01 | ZF-HD |  |  |
| g15574.t1 | WRKY | Vitvi14g01959.t01 | ZF-HD |  |  |
| g15654.t1 | WRKY | Vitvi17g00600.t01 | ZF-HD |  |  |
| g15923.t1 | WRKY | Vitvi17g01082.t01 | ZF-HD |  |  |
| g16152.t1 | WRKY | Vitvi18g00493.t01 | ZF-HD |  |  |
| g16521.t1 | WRKY | Vitvi18g00972.t01 | ZF-HD |  |  |
| g18067.t1 | WRKY | Vitvi18g01872.t01 | ZF-HD |  |  |
| g18067.t2 | WRKY |  |  |  |  |
| g18340.t1 | WRKY |  |  |  |  |
| g1854.t1 | WRKY |  |  |  |  |
| g1855.t1 | WRKY |  |  |  |  |
| g19556.t1 | WRKY |  |  |  |  |
| g1999.t1 | WRKY |  |  |  |  |
| g20342.t1 | WRKY |  |  |  |  |
| g20354.t1 | WRKY |  |  |  |  |
| g20540.t1 | WRKY |  |  |  |  |
| g20893.t1 | WRKY |  |  |  |  |
| g20896.t1 | WRKY |  |  |  |  |
| g21000.t1 | WRKY |  |  |  |  |
| g21412.t1 | WRKY |  |  |  |  |
| g22488.t1 | WRKY |  |  |  |  |
| g22546.t1 | WRKY |  |  |  |  |
| g22793.t1 | WRKY |  |  |  |  |
| g22794.t1 | WRKY |  |  |  |  |
| g23440.t1 | WRKY |  |  |  |  |
| g24778.t1 | WRKY |  |  |  |  |
| g2482.t1 | WRKY |  |  |  |  |
| g24865.t1 | WRKY |  |  |  |  |
| g25179.t1 | WRKY |  |  |  |  |
| g25179.t2 | WRKY |  |  |  |  |
| g25382.t1 | WRKY |  |  |  |  |
| g25382.t2 | WRKY |  |  |  |  |
| g25814.t1 | WRKY |  |  |  |  |
| g27013.t1 | WRKY |  |  |  |  |
| g27122.t1 | WRKY |  |  |  |  |
| g27666.t1 | WRKY |  |  |  |  |
| g2994.t1 | WRKY |  |  |  |  |
| g29975.t1 | WRKY |  |  |  |  |
| g30173.t1 | WRKY |  |  |  |  |
| g30178.t1 | WRKY |  |  |  |  |
| g30178.t2 | WRKY |  |  |  |  |
| g30179.t1 | WRKY |  |  |  |  |
| g30293.t1 | WRKY |  |  |  |  |
| g30427.t1 | WRKY |  |  |  |  |
| g3138.t1 | WRKY |  |  |  |  |
| g31443.t1 | WRKY |  |  |  |  |
| g32642.t1 | WRKY |  |  |  |  |
| g33662.t1 | WRKY |  |  |  |  |
| g3380.t1 | WRKY |  |  |  |  |
| g34971.t1 | WRKY |  |  |  |  |
| g36183.t1 | WRKY |  |  |  |  |
| g3639.t1 | WRKY |  |  |  |  |
| g36947.t1 | WRKY |  |  |  |  |
| g36963.t1 | WRKY |  |  |  |  |
| g585.t1 | WRKY |  |  |  |  |
| g6128.t1 | WRKY |  |  |  |  |
| g6533.t1 | WRKY |  |  |  |  |
| g15940.t1 | YABBY |  |  |  |  |
| g21063.t1 | YABBY |  |  |  |  |
| g22017.t1 | YABBY |  |  |  |  |
| g2383.t1 | YABBY |  |  |  |  |
| g25767.t1 | YABBY |  |  |  |  |
| g30714.t1 | YABBY |  |  |  |  |
| g30715.t1 | YABBY |  |  |  |  |
| g32018.t1 | YABBY |  |  |  |  |
| g32018.t2 | YABBY |  |  |  |  |
| g32018.t3 | YABBY |  |  |  |  |
| g16949.t1 | ZF-HD |  |  |  |  |
| g22046.t1 | ZF-HD |  |  |  |  |
| g22077.t1 | ZF-HD |  |  |  |  |
| g22120.t1 | ZF-HD |  |  |  |  |
| g22631.t1 | ZF-HD |  |  |  |  |
| g22632.t1 | ZF-HD |  |  |  |  |
| g23092.t1 | ZF-HD |  |  |  |  |
| g25678.t1 | ZF-HD |  |  |  |  |
| g26831.t1 | ZF-HD |  |  |  |  |
| g27070.t1 | ZF-HD |  |  |  |  |
| g27071.t1 | ZF-HD |  |  |  |  |
| g27072.t1 | ZF-HD |  |  |  |  |
| g29723.t1 | ZF-HD |  |  |  |  |
| g34788.t1 | ZF-HD |  |  |  |  |
| g34789.t1 | ZF-HD |  |  |  |  |
| g34813.t1 | ZF-HD |  |  |  |  |
| g570.t1 | ZF-HD |  |  |  |  |
| g819.t2 | ZF-HD |  |  |  |  |
