# Supplementary material for: Draft genome of the Native American cold hardy grapevine Vitis riparia Michx. ‘Manitoba 37’
Source: Hortic Res. 2020 Jun 1;7:92. doi: 10.1038/s41438-020-0316-2 (PMC7261805; doi:10.1038/s41438-020-0316-2)
Supplement: Supplementary file 12 — Supplementary Table 7 [file 41438_2020_316_MOESM12_ESM.docx]

**Supplementary Table 7a. *V. riparia* ‘Manitoba 37’ *WRKY* transcription factor classification.**

| ***V. riparia* WRKY protein id** | **Group** | **Conserved motif** | **Domain pattern** | **Zinc finger** |
| --- | --- | --- | --- | --- |
| WRKY Vitri g12956.t1 | I | C-X_4_-C-X_22_-H-X-H(N), C-X_4_-C-X_23_-H-X-H(C) | 2X[WRKYGQK] | C_2_H_2_ |
| WRKY Vitri g14244.t1 | I | C-X_4_-C-X_22_-H-X-H(N), C-X_4_-C-X_23_-H-X-H(C) | 2X[WRKYGQK] | C_2_H_2_ |
| WRKY Vitri g15654.t1 | I | C-X_4_-C-X_23_-H-X-H(N), C-X_4_-C-X_23_-H-X-H(C) | 2X[WRKYGQK] | C_2_H_2_ |
| WRKY Vitri g16152.t1 | I | C-X_4_-C-X_22_-H-X-H(N), C-X_4_-C-X_23_-H-X-H(C) | 2X[WRKYGQK] | C_2_H_2_ |
| WRKY Vitri g19556.t1 | I | C-X_4_-C-X_22_-H-X-H(N) | 2X[WRKYGQK] | C_2_H_2_ |
| WRKY Vitri g1999.t1 | I | C-X_4_-C-X_22_-H-X-H(N), C-X_4_-C-X_23_-H-X-H(C) | 2X[WRKYGQK] | C_2_H_2_ |
| WRKY Vitri g22488.t1 | I | C-X_4_-C-X_22_-H-X-H(N), C-X_4_-C-X_23_-H-X-H(C) | 2X[WRKYGQK] | C_2_H_2_ |
| WRKY Vitri g24778.t1 | I | C-X_4_-C-X_22_-H-X-H(N), C-X_4_-C-X_23_-H-X-H(C) | 2X[WRKYGQK] | C_2_H_2_ |
| WRKY Vitri g25179.t1 | I | C-X_4_-C-X_22_-H-X-H(N), C-X_4_-C-X_23_-H-X-H(C) | 2X[WRKYGQK] | C_2_H_2_ |
| WRKY Vitri g25179.t2 | I | C-X_4_-C-X_22_-H-X-H(N), C-X_4_-C-X_23_-H-X-H(C) | 2X[WRKYGQK] | C_2_H_2_ |
| WRKY Vitri g27122.t1 | I | C-X_4_-C-X_22_-H-X-H(N), C-X_4_-C-X_23_-H-X-H(C) | 2X[WRKYGQK] | C_2_H_2_ |
| WRKY Vitri g30293.t1 | I | C-X_4_-C-X_22_-H-X-H(N), C-X_4_-C-X_23_-H-X-H(C) | 2X[WRKYGQK] | C_2_H_2_ |
| WRKY Vitri g30427.t1 | I | C-X_4_-C-X_22_-H-X-H(N), C-X_4_-C-X_23_-H-X-H(C) | 2X[WRKYGQK] | C_2_H_2_ |
| WRKY Vitri g22793.t1 | IIa | C-X_5_-C-X_23_-H-X-H | WRKYGQK | C_2_H_2_ |
| WRKY Vitri g22794.t1 | IIa | C-X_5_-C-X_23_-H-X-H | WRKYGQK | C_2_H_2_ |
| WRKY Vitri g585.t1 | IIa | C-X_5_-C-X_23_-H-X-H | WRKYGQK | C_2_H_2_ |
| WRKY Vitri g11899.t1 | IIb | C-X_5_-C-X_23_-H-X-H | WRKYGQK | C_2_H_2_ |
| WRKY Vitri g12148.t1 | IIb | C-X_5_-C-X_23_-H-X-H | WRKYGQK | C_2_H_2_ |
| WRKY Vitri g23440.t1 | IIb | C-X_5_-C-X_23_-H-X-H | WRKYGQK | C_2_H_2_ |
| WRKY Vitri g27013.t1 | IIb | C-X_5_-C-X_23_-H-X-H | WRKYGQK | C_2_H_2_ |
| WRKY Vitri g31443.t1 | IIb | C-X_5_-C-X_23_-H-X-H | WRKYGQK | C_2_H_2_ |
| WRKY Vitri g32642.t1 | IIb | C-X_5_-C-X_23_-H-X-H | WRKYGQK | C_2_H_2_ |
| WRKY Vitri g3639.t1 | IIb | C-X_5_-C-X_23_-H-X-H | WRKYGQK | C_2_H_2_ |
| WRKY Vitri g6128.t1 | IIb | C-X_5_-C-X_23_-H-X-H | WRKYGQK | C_2_H_2_ |
| WRKY Vitri g13465.t1 | IIc | C-X_4_-C-X_23_-H-X-H | WRKYGQK | C_2_H_2_ |
| WRKY Vitri g15127.t1 | IIc | C-X_4_-C-X_23_-H-X-H | WRKYGQK | C_2_H_2_ |
| WRKY Vitri g20342.t1 | IIc | C-X_4_-C-X_23_-H-X-H | WRKYGQK | C_2_H_2_ |
| WRKY Vitri g20354.t1 | IIc | C-X_4_-C-X_23_-H-X-H | WRKYGKK | C_2_H_2_ |
| WRKY Vitri g20896.t1 | IIc | C-X_4_-C-X_23_-H-X-H | WRKYGQK | C_2_H_2_ |
| WRKY Vitri g21412.t1 | IIc | C-X_4_-C-X_23_-H-X-H | WRKYGQK | C_2_H_2_ |
| WRKY Vitri g2482.t1 | IIc | C-X_4_-C-X_23_-H-X-H | WRKYGQK | C_2_H_2_ |
| WRKY Vitri g24865.t1 | IIc | C-X_4_-C-X_23_-H-X-H | WRKYGQK | C_2_H_2_ |
| WRKY Vitri g25814.t1 | IIc | C-X_4_-C-X_23_-H-X-H | WRKYGQK | C_2_H_2_ |
| WRKY Vitri g29975.t1 | IIc | C-X_4_-C-X_23_-H-X-H | WRKYGQK | C_2_H_2_ |
| WRKY Vitri g30178.t1 | IIc | C-X_4_-C-X_23_-H-X-H | **WRKYGKK** | C_2_H_2_ |
| WRKY Vitri g30178.t2 | IIc | C-X_4_-C-X_23_-H-X-H | **WRKYGKK** | C_2_H_2_ |
| WRKY Vitri g30179.t1 | IIc | C-X_4_-C-X_23_-H-X-H | **WRKYGKK** | C_2_H_2_ |
| WRKY Vitri g33662.t1 | IIc | C-X_4_-C-X_23_-H-X-H | WRKYGQK | C_2_H_2_ |
| WRKY Vitri g3380.t1 | IIc | C-X_4_-C-X_23_-H-X-H | **WRKYGKK** | C_2_H_2_ |
| WRKY Vitri g34971.t1 | IIc | C-X_4_-C-X_23_-H-X-H | WRKYGQK | C_2_H_2_ |
| WRKY Vitri g36183.t1 | IIc | C-X_4_-C-X_23_-H-X-H | **WVDTDKR** | C_2_H_2_ |
| WRKY Vitri g36947.t1 | IIc | C-X_4_-C-X_23_-H-X-H | WRKYGQK | C_2_H_2_ |
| WRKY Vitri g6533.t1 | IIc | C-X_4_-C-X_23_-H-X-H | **WKKYGQK** | C_2_H_2_ |
| WRKY Vitri g1089.t1 | IId | C-X_5_-C-X_23_-H-X-H | WRKYGQK | C_2_H_2_ |
| WRKY Vitri g15574.t1 | IId | C-X_5_-C-X_23_-H-X-H | WRKYGQK | C_2_H_2_ |
| WRKY Vitri g15923.t1 | IId | C-X_5_-C-X_23_-H-X-H | WRKYGQK | C_2_H_2_ |
| WRKY Vitri g18067.t1 | IId | C-X_5_-C-X_23_-H-X-H | WRKYGQK | C_2_H_2_ |
| WRKY Vitri g18067.t2 | IId | C-X_5_-C-X_23_-H-X-H | WRKYGQK | C_2_H_2_ |
| WRKY Vitri g20540.t1 | IId | C-X_5_-C-X_23_-H-X-H | WRKYGQK | C_2_H_2_ |
| WRKY Vitri g27666.t1 | IId | C-X_5_-C-X_23_-H-X-H | WRKYGQK | C_2_H_2_ |
| WRKY Vitri g30173.t1 | IId | C-X_5_-C-X_23_-H-X-H | WRKYGQK | C_2_H_2_ |
| WRKY Vitri g10779.t1 | IIe | C-X_5_-C-X_23_-H-X-H | WRKYGQK | C_2_H_2_ |
| WRKY Vitri g15094.t1 | IIe | C-X_5_-C-X_23_-H-X-H | WRKYGQK | C_2_H_2_ |
| WRKY Vitri g16521.t1 | IIe | C-X_5_-C-X_23_-H-X-H | WRKYGQK | C_2_H_2_ |
| WRKY Vitri g20893.t1 | IIe | C-X_5_-C-X_23_-H-X-H | WRKYGQK | C_2_H_2_ |
| WRKY Vitri g22546.t1 | IIe | C-X_5_-C-X_23_-H-X-H | WRKYGQK | C_2_H_2_ |
| WRKY Vitri g25382.t1 | IIe | C-X_5_-C-X_23_-H-X-H | WRKYGQK | C_2_H_2_ |
| WRKY Vitri g25382.t2 | IIe | C-X_5_-C-X_23_-H-X-H | WRKYGQK | C_2_H_2_ |
| WRKY Vitri g3138.t1 | IIe | C-X_5_-C-X_23_-H-X-H | WRKYGQK | C_2_H_2_ |
| WRKY Vitri g36963.t1 | IIe | C-X_5_-C-X_23_-H-X-H | WRKYGQK | C_2_H_2_ |
| WRKY Vitri g14157.t1 | III | C-X_7_-C-X_23_-H-X-C | WRKYGQK | C_2_HC |
| WRKY Vitri g14996.t1 | III | C-X_7_-C-X_23_-H-X-C | WRKYGQK | C_2_HC |
| WRKY Vitri g18340.t1 | III | C-X_7_-C-X_23_-H-X-C | **WKKYGQK** | C_2_HC |
| WRKY Vitri g1854.t1 | III | C-X_7_-C-X_23_-H-X-C | WRKYGQK | C_2_HC |
| WRKY Vitri g1855.t1 | III | C-X_7_-C-X_23_-H-X-C | WRKYGQK | C_2_HC |
| WRKY Vitri g21000.t1 | III | C-X_7_-C-X_23_-H-X-C | WRKYGQK | C_2_HC |
| WRKY Vitri g2994.t1 | III | C-X_7_-C-X_23_-H-X-C | WRKYGQK | C_2_HC |

The variants of the conserved WRKYGQK peptide are shown in bold.

**Supplementary Table 7b. The WRKY protein sequences of *V. riparia* ‘Manitoba 37’ and *V. vinifera* ‘PN40024’ 12X.2, V3 based on different WRKY group classification as shown in Figure 3.**

| ***V. riparia* WRKY protein id** | ***V. vinifera* V3 WRKY protein id** | **Group** |
| --- | --- | --- |
| Vitri g12956.t1 | Vitvi01g02157.t01 | I |
| Vitri g14244.t1 | Vitvi04g01163.t01 | I |
| Vitri g16152.t1 | Vitvi08g00793.t01 | I |
| Vitri g19556.t1 | Vitvi08g01134.t01 | I |
| Vitri g1999.t1 | Vitvi10g00270.t01 | I |
| Vitri g22488.t1 | Vitvi11g00694.t01 | I |
| Vitri g24778.t1 | Vitvi12g00664.t01 | I |
| Vitri g25179.t1 | Vitvi14g02007.t01 | I |
| Vitri g25179.t2 | Vitvi19g00617.t01 | I |
| Vitri g27122.t1 | Vitvi19g00927.t01 | I |
| Vitri g30293.t1 | Vitvi06g00741.t01 | I |
| Vitri g30427.t1 |  | I |
| Vitri g22793.t1 | Vitvi04g00510.t01 | IIa |
| Vitri g22794.t1 | Vitvi04g00511.t01 | IIa |
| Vitri g585.t1 | Vitvi09g01122.t01 | IIa |
| Vitri g11899.t1 | Vitvi12g00388.t01 | IIb |
| Vitri g12148.t1 | Vitvi10g00063.t01 | IIb |
| Vitri g23440.t1 | Vitvi19g00530.t01 | IIb |
| Vitri g27013.t1 | Vitvi07g00523.t01 | IIb |
| Vitri g31443.t1 | Vitvi14g01907.t01 | IIb |
| Vitri g32642.t1 | Vitvi12g01676.t01 | IIb |
| Vitri g3639.t1 | Vitvi17g00556.t01 | IIb |
| Vitri g6128.t1 | Vitvi01g00940.t01 | IIb |
| Vitri g13465.t1 | Vitvi08g01221.t01 | IIc |
| Vitri g15127.t1 | Vitvi04g00133.t01 | IIc |
| Vitri g20342.t1 | Vitvi07g01847.t01 | IIc |
| Vitri g20354.t1 | Vitvi04g00760.t01 | IIc |
| Vitri g20896.t1 | Vitvi04g01985.t01 | IIc |
| Vitri g21412.t1 | Vitvi07g01860.t01 | IIc |
| Vitri g2482.t1 | Vitvi15g00539.t01 | IIc |
| Vitri g24865.t1 | Vitvi12g00048.t01 | IIc |
| Vitri g25814.t1 | Vitvi15g01087.t01 | IIc |
| Vitri g29975.t1 | Vitvi14g01523.t01 | IIc |
| Vitri g30178.t1 | Vitvi17g00102.t01 | IIc |
| Vitri g30178.t2 | Vitvi01g01680.t01 | IIc |
| Vitri g30179.t1 | Vitvi05g00145.t01 | IIc |
| Vitri g33662.t1 | Vitvi01g01844.t01 | IIc |
| Vitri g3380.t1 | Vitvi07g00434.t01 | IIc |
| Vitri g34971.t1 | Vitvi10g00732.t01 | IIc |
| Vitri g36183.t1 | Vitvi06g01574.t01 | IIc |
| Vitri g36947.t1 |  | IIc |
| Vitri g6533.t1 |  | IIc |
| Vitri g1089.t1 | Vitvi07g01694.t01 | IId |
| Vitri g15574.t1 | Vitvi02g01847.t01 | IId |
| Vitri g15923.t1 | Vitvi18g00742.t01 | IId |
| Vitri g18067.t1 | Vitvi14g00540.t01 | IId |
| Vitri g18067.t2 | Vitvi07g00026.t01 | IId |
| Vitri g20540.t1 | Vitvi11g01188.t01 | IId |
| Vitri g27666.t1 | Vitvi04g00756.t01 | IId |
| Vitri g30173.t1 |  | IId |
| Vitri g10779.t1 | Vitvi16g01133.t01 | IIe |
| Vitri g15094.t1 | Vitvi16g01132.t01 | IIe |
| Vitri g16521.t1 | Vitvi15g01090.t01 | IIe |
| Vitri g20893.t1 | Vitvi02g00039.t01 | IIe |
| Vitri g22546.t1 | Vitvi07g00421.t01 | IIe |
| Vitri g25382.t1 | Vitvi10g00618.t01 | IIe |
| Vitri g25382.t2 | Vitvi12g00148.t01 | IIe |
| Vitri g3138.t1 | Vitvi10g01078.t01 | IIe |
| Vitri g36963.t1 |  | IIe |
| Vitri g14157.t1 | Vitvi13g00189.t01 | III |
| Vitri g14996.t1 | Vitvi13g01916.t01 | III |
| Vitri g18340.t1 | Vitvi08g00868.t01 | III |
| Vitri g1854.t1 | Vitvi15g01003.t01 | III |
| Vitri g1855.t1 | Vitvi16g01213.t01 | III |
| Vitri g21000.t1 | Vitvi02g00114.t01 | III |
| Vitri g2994.t1 |  | III |

**Supplementary Table 7c. *WRKY* transcription factor classification comparison with different species.**

|  |  | **Group II** | | | | | | | | |  | |  | |  | |  |
| --- | --- | --- | --- | --- | --- | --- | --- | --- | --- | --- | --- | --- | --- | --- | --- | --- | --- |
| **Species** | **Group I** | **IIa** | | **IIb** | | **IIc** | | **IId** | | **IIe** | | **Group III** | | **NG** | | **Total** | |
| *V. riparia* | 13 | 3 | 8 | | 19 | | 8 | | 9 | | 7 | |  | | 67 | |  |
| *V. vinifera* V3 | 11 | 3 | 8 | | 17 | | 7 | | 8 | | 6 | |  | | 60 | |  |
| *V. vinifera^a^* ^V1^ | 12 | 3 | 8 | | 15 | | 6 | | 7 | | 6 | | 2 | | 59 | |  |
| Arabidopsis^a^ | 16 | 3 | 8 | | 17 | | 7 | | 8 | | 14 | | 1 | | 74 | |  |
| Rice^a^ | 15 | 4 | 8 | | 15 | | 7 | | 11 | | 36 | |  | | 96 | |  |
| Castor bean^a^ | 9 | 3 | 10 | | 12 | | 3 | | 5 | | 5 | |  | | 47 | |  |
| Poplar^a^ | 50 | 5 | 9 | | 13 | | 13 | | 4 | | 10 | |  | | 104 | |  |
| Tomato^a^ | 15 | 5 | 8 | | 16 | | 6 | | 17 | | 11 | | 3 | | 81 | |  |

^a^ According to Wang *et al.,* 2014

**Supplementary File 7d. *V. riparia* ‘Manitoba 37’ *MYB* transcription factor classification.**

| Gene ID | Subgroup | MYB |
| --- | --- | --- |
| Vitvi08g00069.t01 | 1 | AtMYB060 |
| g29703.t1 | 1 | AtMYB060 |
| Vitvi14g01960.t01 | 1 | AtMYB094 |
| Vitvi17g00598.t01 | 1 | AtMYB094 |
| g27073.t1 | 1 | AtMYB094 |
| g569.t1 | 1 | AtMYB094 |
| Vitvi05g01732.t01 | 2 | AtMYB015 |
| Vitvi05g01733.t01 | 2 | AtMYB015 |
| Vitvi12g00625.t01 | 2 | AtMYB015 |
| Vitvi12g00626.t01 | 2 | AtMYB015 |
| Vitvi14g00612.t01 | 2 | AtMYB015 |
| Vitvi16g01515.t01 | 2 | AtMYB015 |
| Vitvi16g01521.t01 | 2 | AtMYB015 |
| Vitvi16g01524.t01 | 2 | AtMYB015 |
| g11585.t1 | 2 | AtMYB015 |
| g25720.t1 | 2 | AtMYB015 |
| g25721.t1 | 2 | AtMYB015 |
| g2903.t1 | 2 | AtMYB015 |
| g29074.t1 | 2 | AtMYB015 |
| g30634.t1 | 2 | AtMYB015 |
| g30635.t1 | 2 | AtMYB015 |
| g30830.t1 | 2 | AtMYB015 |
| g30834.t1 | 2 | AtMYB015 |
| g30838.t1 | 2 | AtMYB015 |
| Vitvi19g01669.t01 | 3 | AtMYB058 |
| g29760.t1 | 3 | AtMYB058 |
| Vitvi15g00938.t01 | 4 | AtMYB003 |
| Vitvi16g00103.t01 | 4 | AtMYB003 |
| Vitvi16g01513.t01 | 4 | AtMYB003 |
| g12826.t1 | 4 | AtMYB003 |
| g24587.t1 | 4 | AtMYB003 |
| g26747.t1 | 4 | AtMYB003 |
| g30828.t1 | 4 | AtMYB003 |
| g30839.t1 | 4 | AtMYB003 |
| g31080.t1 | 4 | AtMYB003 |
| g37769.t1 | 4 | AtMYB003 |
| g37769.t2 | 4 | AtMYB003 |
| Vitvi01g00401.t01 | 4 | AtMYB004 |
| Vitvi03g00136.t01 | 4 | AtMYB004 |
| Vitvi04g00153.t01 | 4 | AtMYB004 |
| Vitvi04g00155.t01 | 4 | AtMYB004 |
| Vitvi04g01486.t01 | 4 | AtMYB004 |
| Vitvi07g02009.t01 | 4 | AtMYB004 |
| Vitvi14g00974.t01 | 4 | AtMYB004 |
| Vitvi17g00231.t01 | 4 | AtMYB004 |
| Vitvi18g01867.t01 | 4 | AtMYB004 |
| Vitvi18g01868.t01 | 4 | AtMYB004 |
| g22067.t1 | 4 | AtMYB004 |
| g22085.t1 | 4 | AtMYB004 |
| g22086.t1 | 4 | AtMYB004 |
| g22086.t2 | 4 | AtMYB004 |
| g24535.t1 | 4 | AtMYB004 |
| g25236.t1 | 4 | AtMYB004 |
| g25488.t1 | 4 | AtMYB004 |
| g28207.t1 | 4 | AtMYB004 |
| g31078.t1 | 4 | AtMYB004 |
| g3421.t1 | 4 | AtMYB004 |
| g3810.t1 | 4 | AtMYB004 |
| Vitvi09g00112.t01 | 4 | AtMYB007 |
| g25237.t1 | 4 | AtMYB007 |
| Vitvi04g00157.t01 | 4 | AtMYB032 |
| g3423.t1 | 4 | AtMYB032 |
| Vitvi04g00156.t01 | 5 | AtMYB123 |
| Vitvi09g00113.t01 | 5 | AtMYB123 |
| Vitvi11g00097.t01 | 5 | AtMYB123 |
| Vitvi11g00098.t01 | 5 | AtMYB123 |
| Vitvi11g00099.t01 | 5 | AtMYB123 |
| Vitvi12g00527.t01 | 5 | AtMYB123 |
| g22302.t1 | 5 | AtMYB123 |
| g22303.t1 | 5 | AtMYB123 |
| g22304.t1 | 5 | AtMYB123 |
| g23579.t1 | 5 | AtMYB123 |
| g3420.t1 | 5 | AtMYB123 |
| g3422.t1 | 5 | AtMYB123 |
| g3424.t1 | 5 | AtMYB123 |
| g29018.t1 | 6 | AtMYB090 |
| Vitvi01g00094.t01 | 6 | AtMYB113 |
| Vitvi01g00095.t01 | 6 | AtMYB113 |
| Vitvi02g01013.t01 | 6 | AtMYB113 |
| Vitvi02g01015.t01 | 6 | AtMYB113 |
| Vitvi02g01017.t01 | 6 | AtMYB113 |
| Vitvi02g01019.t01 | 6 | AtMYB113 |
| Vitvi02g01022.t01 | 6 | AtMYB113 |
| Vitvi02g01024.t01 | 6 | AtMYB113 |
| Vitvi02g01307.t01 | 6 | AtMYB113 |
| Vitvi02g01308.t01 | 6 | AtMYB113 |
| Vitvi02g01309.t01 | 6 | AtMYB113 |
| Vitvi14g00925.t01 | 6 | AtMYB113 |
| Vitvi14g00930.t01 | 6 | AtMYB113 |
| Vitvi14g00940.t01 | 6 | AtMYB113 |
| g10813.t1 | 6 | AtMYB113 |
| g11202.t1 | 6 | AtMYB113 |
| g25869.t1 | 6 | AtMYB113 |
| g25870.t1 | 6 | AtMYB113 |
| g27750.t1 | 6 | AtMYB113 |
| g27755.t1 | 6 | AtMYB113 |
| g27758.t1 | 6 | AtMYB113 |
| g29015.t1 | 6 | AtMYB113 |
| g32947.t1 | 6 | AtMYB113 |
| Vitvi07g00393.t01 | 7 | AtMYB012 |
| Vitvi13g01359.t01 | 7 | AtMYB012 |
| g32182.t1 | 7 | AtMYB012 |
| g37000.t1 | 7 | AtMYB012 |
| g5037.t1 | 7 | AtMYB012 |
| Vitvi05g00084.t01 | 7 | AtMYB111 |
| g5161.t1 | 7 | AtMYB111 |
| Vitvi14g01987.t01 | 9 | AtMYB016 |
| Vitvi17g00623.t01 | 9 | AtMYB016 |
| g27100.t1 | 9 | AtMYB016 |
| g29051.t1 | 9 | AtMYB016 |
| Vitvi15g01161.t01 | 9 | AtMYB017 |
| Vitvi16g01017.t01 | 9 | AtMYB017 |
| g13320.t1 | 9 | AtMYB017 |
| g27273.t1 | 9 | AtMYB017 |
| Vitvi01g01052.t01 | 9 | AtMYB106 |
| g13376.t1 | 9 | AtMYB106 |
| g13376.t2 | 9 | AtMYB106 |
| Vitvi02g00725.t01 | 10 | AtMYB009 |
| Vitvi16g00098.t01 | 10 | AtMYB009 |
| Vitvi16g00106.t01 | 10 | AtMYB009 |
| g30835.t1 | 10 | AtMYB009 |
| g37779.t1 | 10 | AtMYB009 |
| g8673.t1 | 10 | AtMYB009 |
| g11583.t1 | 10 | AtMYB107 |
| g30879.t1 | 10 | AtMYB107 |
| g5233.t1 | 10 | AtMYB107 |
| g8695.t1 | 10 | AtMYB107 |
| g25729.t1 | 11 | AtMYB041 |
| Vitvi10g00345.t01 | 11 | AtMYB074 |
| g13157.t1 | 11 | AtMYB074 |
| Vitvi10g00329.t01 | 11 | AtMYB102 |
| Vitvi12g00632.t01 | 11 | AtMYB102 |
| Vitvi16g00442.t01 | 11 | AtMYB102 |
| Vitvi19g00306.t01 | 11 | AtMYB102 |
| g13148.t1 | 11 | AtMYB102 |
| g15207.t1 | 11 | AtMYB102 |
| g36079.t1 | 11 | AtMYB102 |
| g12605.t1 | 12 | AtMYB028 |
| Vitvi16g00125.t01 | 12 | AtMYB029 |
| g30837.t1 | 12 | AtMYB034 |
| g31335.t1 | 12 | AtMYB034 |
| g7954.t1 | 12 | AtMYB034 |
| g8701.t1 | 12 | AtMYB034 |
| Vitvi16g00100.t01 | 12 | AtMYB051 |
| g30842.t1 | 12 | AtMYB051 |
| g37767.t1 | 12 | AtMYB051 |
| Vitvi16g00441.t01 | 12 | AtMYB076 |
| g24681.t1 | 12 | AtMYB122 |
| g30827.t1 | 13 | AtMYB050 |
| g30829.t1 | 13 | AtMYB050 |
| g37555.t1 | 13 | AtMYB050 |
| g2603.t1 | 13 | AtMYB055 |
| Vitvi05g00275.t01 | 13 | AtMYB061 |
| Vitvi07g00515.t01 | 13 | AtMYB061 |
| Vitvi14g00018.t01 | 13 | AtMYB061 |
| g19855.t1 | 13 | AtMYB061 |
| g6138.t1 | 13 | AtMYB061 |
| Vitvi17g00895.t01 | 13 | AtMYB086 |
| g28423.t1 | 13 | AtMYB086 |
| Vitvi06g01321.t01 | 14 | AtMYB036 |
| Vitvi08g01623.t01 | 14 | AtMYB036 |
| Vitvi11g00228.t01 | 14 | AtMYB036 |
| Vitvi11g01283.t01 | 14 | AtMYB036 |
| g17534.t1 | 14 | AtMYB036 |
| g19001.t1 | 14 | AtMYB036 |
| g35935.t1 | 14 | AtMYB036 |
| g38222.t1 | 14 | AtMYB036 |
| Vitvi04g00049.t01 | 14 | AtMYB068 |
| g3287.t1 | 14 | AtMYB068 |
| Vitvi07g03055.t01 | 14 | AtMYB084 |
| Vitvi18g00605.t01 | 14 | AtMYB084 |
| g17920.t1 | 14 | AtMYB084 |
| g20700.t1 | 14 | AtMYB084 |
| Vitvi10g01608.t01 | 15 | AtMYB000 |
| Vitvi15g01002.t01 | 15 | AtMYB000 |
| Vitvi16g01522.t01 | 15 | AtMYB000 |
| Vitvi16g01523.t01 | 15 | AtMYB000 |
| g11584.t1 | 15 | AtMYB000 |
| g31385.t1 | 15 | AtMYB000 |
| g5966.t1 | 15 | AtMYB000 |
| Vitvi14g03020.t01 | 15 | AtMYB023 |
| g24050.t1 | 15 | AtMYB023 |
| g37762.t1 | 15 | AtMYB023 |
| g37765.t1 | 15 | AtMYB023 |
| Vitvi01g00737.t01 | 15 | AtMYB066 |
| Vitvi02g01732.t01 | 15 | AtMYB066 |
| Vitvi04g00158.t01 | 15 | AtMYB066 |
| Vitvi04g00160.t01 | 15 | AtMYB066 |
| Vitvi09g00110.t01 | 15 | AtMYB066 |
| Vitvi17g00822.t01 | 15 | AtMYB066 |
| g12485.t1 | 15 | AtMYB066 |
| g15851.t1 | 15 | AtMYB066 |
| g21037.t1 | 15 | AtMYB066 |
| g31082.t1 | 15 | AtMYB066 |
| g3427.t1 | 15 | AtMYB066 |
| Vitvi16g01015.t01 | 16 | AtMYB018 |
| g11203.t1 | 16 | AtMYB018 |
| Vitvi06g01139.t01 | 18 | AtMYB033 |
| Vitvi13g01266.t01 | 18 | AtMYB033 |
| g13970.t1 | 18 | AtMYB033 |
| g3621.t1 | 18 | AtMYB033 |
| g5062.t1 | 18 | AtMYB065 |
| Vitvi12g00376.t01 | 18 | AtMYB101 |
| Vitvi19g00508.t01 | 18 | AtMYB101 |
| g13581.t1 | 18 | AtMYB101 |
| g23422.t1 | 18 | AtMYB101 |
| Vitvi14g01750.t01 | 19 | AtMYB024 |
| g24064.t1 | 19 | AtMYB024 |
| Vitvi01g00302.t01 | 20 | AtMYB062 |
| g15987.t1 | 20 | AtMYB062 |
| Vitvi07g00455.t01 | 20 | AtMYB078 |
| g2507.t1 | 20 | AtMYB078 |
| g36924.t1 | 20 | AtMYB078 |
| Vitvi05g00166.t01 | 20 | AtMYB108 |
| Vitvi17g00309.t01 | 20 | AtMYB116 |
| g11768.t1 | 20 | AtMYB116 |
| Vitvi04g01697.t01 | 21 | AtMYB052 |
| Vitvi17g00159.t01 | 21 | AtMYB052 |
| Vitvi18g01209.t01 | 21 | AtMYB052 |
| g1047.t1 | 21 | AtMYB052 |
| g18636.t1 | 21 | AtMYB052 |
| g26803.t1 | 21 | AtMYB052 |
| Vitvi01g01028.t01 | 21 | AtMYB105 |
| Vitvi08g00107.t01 | 21 | AtMYB105 |
| Vitvi14g01976.t01 | 21 | AtMYB105 |
| g22617.t1 | 21 | AtMYB105 |
| g27091.t1 | 21 | AtMYB105 |
| g29729.t1 | 21 | AtMYB105 |
| Vitvi03g00559.t01 | 22 | AtMYB044 |
| g4257.t1 | 22 | AtMYB044 |
| Vitvi07g01676.t01 | 22 | AtMYB073 |
| Vitvi18g00725.t01 | 22 | AtMYB073 |
| Vitvi18g00843.t01 | 22 | AtMYB073 |
| g18051.t1 | 22 | AtMYB073 |
| g20557.t1 | 22 | AtMYB073 |
| g682.t1 | 22 | AtMYB073 |
| g682.t2 | 22 | AtMYB073 |
| Vitvi13g00076.t01 | 22 | AtMYB077 |
| g26493.t1 | 22 | AtMYB077 |
| Vitvi04g01842.t01 | 23 | AtMYB001 |
| Vitvi08g01298.t01 | 23 | AtMYB001 |
| g13736.t1 | 23 | AtMYB001 |
| Vitvi13g00494.t01 | 23 | AtMYB109 |
| g12680.t1 | 23 | AtMYB109 |
| g17113.t1 | 23 | AtMYB109 |
| g34640.t1 | 23 | AtMYB109 |
| g30831.t1 | 24 | AtMYB053 |
| g24592.t1 | 24 | AtMYB092 |
| g30826.t1 | 24 | AtMYB092 |
| g36017.t1 | 24 | AtMYB092 |
| g7947.t1 | 24 | AtMYB092 |
| Vitvi07g02075.t01 | 24 | AtMYB093 |
| Vitvi11g01323.t01 | 24 | AtMYB093 |
| Vitvi16g00129.t01 | 24 | AtMYB093 |
| Vitvi16g01448.t01 | 24 | AtMYB093 |
| Vitvi16g01449.t01 | 24 | AtMYB093 |
| Vitvi16g01520.t01 | 24 | AtMYB093 |
| Vitvi16g01689.t01 | 24 | AtMYB093 |
| Vitvi18g00406.t01 | 24 | AtMYB093 |
| g10287.t1 | 24 | AtMYB093 |
| g10390.t1 | 24 | AtMYB093 |
| g12303.t1 | 24 | AtMYB093 |
| g15752.t1 | 24 | AtMYB093 |
| g19466.t1 | 24 | AtMYB093 |
| g24677.t1 | 24 | AtMYB093 |
| g30843.t1 | 24 | AtMYB093 |
| g32787.t1 | 24 | AtMYB093 |
| g33234.t1 | 24 | AtMYB093 |
| g36077.t1 | 24 | AtMYB093 |
| g37763.t1 | 24 | AtMYB093 |
| g8536.t1 | 24 | AtMYB093 |
| Vitvi06g00611.t01 | 25 | AtMYB064 |
| g1263.t1 | 25 | AtMYB119 |
| Vitvi02g00196.t01 | Atypical | AtMYB005 |
| Vitvi06g00059.t01 | Atypical | AtMYB005 |
| Vitvi08g01797.t01 | Atypical | AtMYB005 |
| Vitvi10g01641.t01 | Atypical | AtMYB005 |
| g14651.t1 | Atypical | AtMYB005 |
| g17729.t1 | Atypical | AtMYB005 |
| g31482.t1 | Atypical | AtMYB005 |
| g6196.t1 | Atypical | AtMYB005 |
| Vitvi02g00195.t01 | Atypical | AtMYB006 |
| Vitvi17g00232.t01 | Atypical | AtMYB006 |
| Vitvi14g02430.t01 | Atypical | AtMYB020 |
| Vitvi09g00142.t01 | Atypical | AtMYB026 |
| g16097.t1 | Atypical | AtMYB026 |
| g21692.t1 | Atypical | AtMYB026 |
| g21693.t1 | Atypical | AtMYB026 |
| g31052.t1 | Atypical | AtMYB026 |
| Vitvi08g01607.t01 | Atypical | AtMYB027 |
| g17515.t1 | Atypical | AtMYB027 |
| Vitvi14g01845.t01 | Atypical | AtMYB035 |
| Vitvi17g00510.t01 | Atypical | AtMYB035 |
| g23963.t1 | Atypical | AtMYB035 |
| g32204.t1 | Atypical | AtMYB035 |
| Vitvi14g01615.t01 | Atypical | AtMYB040 |
| g29881.t1 | Atypical | AtMYB040 |
| Vitvi02g01823.t01 | Atypical | AtMYB042 |
| Vitvi16g00775.t01 | Atypical | AtMYB042 |
| g15957.t1 | Atypical | AtMYB042 |
| g25123.t1 | Atypical | AtMYB042 |
| g439.t1 | Atypical | AtMYB043 |
| g5077.t1 | Atypical | AtMYB046 |
| Vitvi03g01247.t01 | Atypical | AtMYB047 |
| Vitvi06g00414.t01 | Atypical | AtMYB059 |
| g19683.t1 | Atypical | AtMYB059 |
| Vitvi01g01271.t01 | Atypical | AtMYB067 |
| Vitvi16g00102.t01 | Atypical | AtMYB067 |
| Vitvi16g01525.t01 | Atypical | AtMYB067 |
| Vitvi19g01749.t01 | Atypical | AtMYB067 |
| g16350.t1 | Atypical | AtMYB067 |
| g20290.t1 | Atypical | AtMYB067 |
| g35128.t1 | Atypical | AtMYB067 |
| g36145.t1 | Atypical | AtMYB067 |
| g36610.t1 | Atypical | AtMYB067 |
| g36686.t1 | Atypical | AtMYB067 |
| g9018.t1 | Atypical | AtMYB067 |
| Vitvi05g00861.t01 | Atypical | AtMYB071 |
| g2113.t1 | Atypical | AtMYB071 |
| g5764.t1 | Atypical | AtMYB072 |
| Vitvi19g00758.t01 | Atypical | AtMYB080 |
| g24682.t1 | Atypical | AtMYB080 |
| g35922.t1 | Atypical | AtMYB080 |
| Vitvi01g00956.t01 | Atypical | AtMYB088 |
| g23078.t1 | Atypical | AtMYB088 |
| Vitvi08g01203.t01 | Atypical | AtMYB091 |
| Vitvi14g01247.t01 | Atypical | AtMYB091 |
| g18211.t1 | Atypical | AtMYB091 |
| g24853.t1 | Atypical | AtMYB091 |
| Vitvi17g00832.t01 | Atypical | AtMYB098 |
| g24072.t1 | Atypical | AtMYB098 |
| g33806.t1 | Atypical | AtMYB098 |
| Vitvi02g00028.t01 | Atypical | AtMYB103 |
| g25373.t1 | Atypical | AtMYB103 |
| Vitvi15g00594.t01 | Atypical | AtMYB125 |
| g12350.t1 | Atypical | AtMYB125 |

**Supplementary File 7e. *V. riparia* ‘Manitoba 37’ *ERF* transcription factor classification.**

| **V. vinifera V3** | **V. Riparia** | [**V. vinifera V1**](http://genoscope.id/) | **Gene.name** | **Gene name**  **Zhuang etal. 2010.** | **Best hit**  **Arabidopsis** | **Family.Group** |
| --- | --- | --- | --- | --- | --- | --- |
| Vitvi19g00260 | g19438 | GSVIVP00027632001 | VvERF001 | VvDREB-A6-5 | AT2G20880 | I |
| Vitvi02g00121 | | GSVIVP00002438001 | VvERF002 | VvDREB-A6-3 | AT1G64380 | I |
| NA |  | GSVIVP00010923001 | VvERF003 | VvDREB-A6-2 | AT4G39780 | I |
| NA |  | GSVIVP00015253001 | VvERF004 | VvDREB-A6-1 | AT1G78080 | I |
| Vitvi05g01073 | g13650 | GSVIVP00031685001 | VvERF005 | VvDREB-A6-4 | AT4G13620 | I |
| Vitvi18g00755 | | GSVIVP00014863001 | VvERF006 | VvDREB-A5-6 | AT4G36900 | II |
| Vitvi18g02437 | g18077 | GSVIVP00014863001 | VvERF006 | VvDREB-A5-6 | AT4G36900 | II |
| Vitvi07g01702 | g20533 | GSVIVP00019541001 | VvERF007 | VvDREB-A5-5 | AT2G23340 | II |
| Vitvi18g00295 | | GSVIVP00015361001 | VvERF008 | VvDREB-A5-1 | AT1G21910 | II |
| Vitvi11g00045 | g22239 | GSVIVP00016398001 | VvERF009 | VvDREB-A5-3 | AT1G19210 | II |
| Vitvi11g00046 | | GSVIVP00016399001 | VvERF010 | VvDREB-A5-2 | AT1G19210 | II |
| Vitvi04g00190 | g3458 | GSVIVP00032308001 | VvERF011 | VvDREB-A5-4 | AT1G74930 | II |
| Vitvi18g02191 | g36825, g32973 | GSVIVP00021853001 | VvERF012 | VvDREB-A5-7 | AT1G22810 | II |
| Vitvi18g00755 | | GSVIVP00014863001 | VvERF013 |  | AT3G50260 | II |
| Vitvi18g02437 | g18077 | GSVIVP00014863001 | VvERF013 |  | AT3G50260 | II |
| NA |  | GIDVvP00036972001 | VvERF014 | VvAP2/ERF-4 | AT1G33760 | III |
| Vitvi15g00947 | g26762 | GSVIVP00008628001 | VvERF015 | VvDREB-A4-10 | AT1G01250 | III |
| NA |  | GIDVvP00036973001 | VvERF016 | VvAP2/ERF-5 | AT1G71450 | III |
| Vitvi18g02398 | g4444 | GSVIVP00031975001 | VvERF017 | VvDREB-A4-11 | AT1G71450 | III |
| Vitvi02g00406 | g249 | GSVIVP00001093001 | VvERF018 | VvDREB-A4-2 | AT1G12630 | III |
| Vitvi16g00942 | g11887 | GSVIVP00007409001 | VvERF019 | VvDREB-A4-1 | AT5G52020 | III |
| Vitvi08g01503 | | GSVIVP00021663001 | VvERF020 | VvDREB-A4-3 | AT2G36450 | III |
| Vitvi02g00407 | g250 | GSVIVP00001092001 | VvERF021 | VvDREB-A1-2 | AT1G63030 | III |
| Vitvi16g00941 | g11885 | GSVIVP00007410001 | VvERF022 | VvDREB-A1-1 | AT4G25490 | III |
| Vitvi19g01278 | | GSVIVP00009060001 | VvERF023 | VvDREB-A1-3 | AT1G12610 | III |
| Vitvi17g00769 | | GSVIVP00016137001 | VvERF024 | VvDREB-A1-4 | AT1G12610 | III |
| Vitvi11g01322 | | GSVIVP00016556001 | VvERF025 | VvDREB-A1-5 | AT4G25480 | III |
| Vitvi06g01414 | g19105 | GSVIVP00022074001 | VvERF026 | VvDREB-A1-6 | AT4G25490 | III |
| Vitvi06g01411 | g19103, g19104 | GSVIVP00022076001 | VvERF027 | VvDREB-A1-7 | AT4G25490 | III |
| NA |  | GIDVvP00011331001 | VvERF028 |  | AT2G44940 | III |
| NA |  | GIDVvP00033798001 | VvERF029 |  | AT2G44940 | III |
| Vitvi02g01729 | g15854 | GSVIVP00009518001 | VvERF030 | VvDREB-A4-7 | AT2G44940 | III |
| Vitvi02g01730 | | GSVIVP00009519001 | VvERF031 | VvDREB-A4-9 | AT2G44940 | III |
| NA |  | GIDVvP00023095001 | VvERF032 | VvDREB-A4-6 | AT2G44940 | III |
| NA |  | GIDVvP00023097001 | VvERF033 | VvDREB-A4-8 | AT2G44940 | III |
| Vitvi09g00323 | g22462, g3575 | GSVIVP00034342001 | VvERF034 | VvDREB-A4-5 | AT2G44940 | III |
| Vitvi04g00300 | | GSVIVP00032424001 | VvERF035 | VvDREB-A4-4 | AT5G11590 | III |
| NA |  | GIDVvP00044224001 | VvERF036 | VvERF-B6-5 | AT2G40350 | IV |
| Vitvi13g00116 | | GSVIVP00029367001 | VvERF037 | VvDREB-A2-3 | AT2G40340 | IV |
| Vitvi08g00778 | g14258 | GSVIVP00037399001 | VvERF038 | vvAP2/ERF-6 | AT2G38340 | IV |
| Vitvi18g01029 | | GSVIVP00014524001 | VvERF039 | VvDREB-A2-4 | AT1G75490 | IV |
| Vitvi08g01966 | | GSVIVP00025555001 | VvERF040 | VvDREB-A2-1 | AT3G57600 | IV |
| NA |  | GIDVvP00043670001 | VvERF041 |  | AT5G19790 | V |
| Vitvi09g00593 | g35575 | GSVIVP00000349001 | VvERF042 | VvERF-B6-1 | AT1G15360 | V |
| Vitvi11g00467 | g25281 | GSVIVP00016902001 | VvERF043 | VvERF-B6-3 | AT1G15360 | V |
| Vitvi04g00479 | | GSVIVP00032652001 | VvERF044 | VvERF-B6-2 | AT1G15360 | V |
| Vitvi04g00533 | g22771 | GSVIVP00032717001 | VvERF045 | VvERF-B6-6 | AT5G25190 | V |
| Vitvi09g00834 | g32108 | GSVIVP00033298001 | VvERF046 | VvERF-B6-7 | AT1G15360 | V |
| Vitvi09g00837 | g22771, g32108, g32111, g20501 | GSVIVP00033300001 | VvERF047 | VvERF-B6-8 | AT5G25190 | V |
| Vitvi17g00406 | | GSVIVP00017593001 | VvERF048 | VvERF-B6-13 | AT5G19790 | V |
| Vitvi14g02001 | | GSVIVP00021016001 | VvERF049 | VvERF-B6-9 | AT5G19790 | V |
| Vitvi06g00052 | | GSVIVP00024804001 | VvERF050 | VvERF-B6-11 | AT5G19790 | V |
| Vitvi08g01965 | g17731 | GSVIVP00025461001 | VvERF051 | VvERF-B6-10 | AT5G19790 | V |
| Vitvi02g00286 | | GSVIVP00000455001 | VvERF052 | VvERF-B6-12 | AT4G23750 | VI |
| Vitvi02g00093 | | GSVIVP00002468001 | VvERF053 | VvERF-B5-3 | AT4G23750 | VI |
| Vitvi16g01201 | | GSVIVP00006483001 | VvERF054 | VvERF-B5-2 | AT4G27950 | VI |
| Vitvi06g00810 | | GSVIVP00023927001 | VvERF055 | VvERF-B5-1 | AT4G23750 | VI |
| Vitvi15g01021 | | GSVIVP00026980001 | VvERF056 | VvERF-B5-4 | AT4G27950 | VI |
| Vitvi07g00357 | | GSVIVP00028041001 | VvERF057 | VvERF-B2-2 | AT2G47520 | VII |
| Vitvi05g01724 | | GSVIVP00033049001 | VvERF058 | VvERF-B2-3 | AT3G16770 | VII |
| Vitvi09g00031 | | GSVIVP00034010001 | VvERF059 | VvERF-B2-1 | AT1G53910 | VII |
| NA |  | GIDVvP00005323001 | VvERF060 |  | AT1G28360 | VIII |
| NA |  | GIDVvP00007464001 | VvERF061 |  | AT1G28360 | VIII |
| NA |  | GIDVvP00069539001 | VvERF062 |  | AT1G50640 | VIII |
| NA |  | GIDVvP00070292001 | VvERF063 |  | AT3G15210 | VIII |
| Vitvi10g01594 | g29239, g35196 | GSVIVP00003062001 | VvERF064 | VvERF-B1-3 | AT3G15210 | VIII |
| Vitvi07g02066 | g15575 | GSVIVP00023866001 | VvERF065 | VvERF-B1-1 | AT1G50640 | VIII |
| Vitvi12g00451 | g23495 | GSVIVP00025959001 | VvERF066 | VvERF-B1-2 | AT1G50640 | VIII |
| Vitvi01g00645 | g26173 | GSVIVP00009456001 | VvERF067 | VvERF-B1-4 | AT1G24590 | VIII |
| NA |  | GSVIVP00019797001 | VvERF068 | VvERF-B1-7 | AT5G18560 | VIII |
| NA |  | GSVIVP00029726001 | VvERF069 | VvERF-B1-6 | AT1G28160 | VIII |
| Vitvi14g01441 | | GSVIVP00037974001 | VvERF070 | VvERF-B1-5 | AT5G13910 | VIII |
| NA |  | GIDVvP00014061001 | VvERF071 |  | AT5G07580 | IX |
| Vitvi15g01202 | | GSVIVP00019092001 | VvERF072 | VvERF-B3-34 | AT4G25490 | IX |
| Vitvi15g00566 | | GSVIVP00019092001 | VvERF072 | VvERF-B3-34 | AT4G25490 | IX |
| Vitvi15g01203 | | GSVIVP00019092001 | VvERF072 | VvERF-B3-34 | AT4G25490 | IX |
| NA |  | GIDVvP00033671001 | VvERF073 |  | AT5G47220 | IX |
| NA |  | GIDVvP00033672001 | VvERF074 |  | AT5G47220 | IX |
| Vitvi10g00521 | | GSVIVP00003018001 | VvERF075 | VvERF-B3-37 | AT5G47220 | IX |
| NA |  | GSVIVP00009541001 | VvERF076 | VvERF-B3-36 | AT5G47220 | IX |
| Vitvi16g00349 | g32255 | GSVIVP00014229001 | VvERF077 | VvERF-B3-35 | AT2G44840 | IX |
| NA |  | GSVIVP00009539001 | VvERF078 | VvERF-B3-33 | AT5G47230 | IX |
| Vitvi16g00350 | | GSVIVP00014231001 | VvERF079 | VvERF-B3-29 | AT4G17490 | IX |
| Vitvi16g01429 | | GSVIVP00014237001 | VvERF080 | VvERF-B3-27 | AT5G61600 | IX |
| Vitvi16g01438 | | GSVIVP00014238001 | VvERF081 | VvERF-B3-23 | AT5G51190 | IX |
| Vitvi16g01430 | | GSVIVP00014240001 | VvERF082 | VvERF-B3-28 | AT5G61600 | IX |
| Vitvi16g01438? | | GSVIVP00014244001 | VvERF083 | VvERF-B3-22 | AT5G51190 | IX |
| Vitvi16g01432 | | GSVIVP00014245001 | VvERF084 | VvERF-B3-20 | AT5G61600 | IX |
| Vitvi16g00370 | | GSVIVP00014256001 | VvERF085 | VvERF-B3-21 | AT4G17490 | IX |
| Vitvi16g01444 | | GSVIVP00014260001 | VvERF086 | VvERF-B3-19 | AT4G17490 | IX |
| Vitvi16g01437 | | GSVIVP00014261001 | VvERF087 | VvERF-B3-15 | AT4G17490 | IX |
| NA |  | GSVIVP00014263001 | VvERF088 | quindi controllo colonna G e colonna H? | AT5G51190 | IX |
| Vitvi16g01434?/Vitvi16g01437? | | GSVIVP00014264001 | VvERF089 | VvERF-B3-16 | AT4G17490 | IX |
| NA |  | GSVIVP00014265001 | VvERF090 | VvERF-B3-18 | AT5G47230 | IX |
| Vitvi16g01434?/Vitvi16g01437? | | GSVIVP00014266001 | VvERF091 | VvERF-B3-14 | AT5G61600 | IX |
| Vitvi16g00380 | | GSVIVP00014267001 | VvERF092 | VvERF-B3-32 | AT5G51190 | IX |
| NA |  | GIDVvP00005378001 | VvERF093 | VvERF-B3-2 | AT3G23240 | IX |
| NA |  | GIDVvP00014064001 | VvERF094 |  | AT3G23230 | IX |
| Vitvi10g00522 | g16423 | GSVIVP00003017001 | VvERF095 | VvERF-B3-8 | AT4G18450 | IX |
| Vitvi07g02062 | | GSVIVP00007517001 | VvERF096 | VvERF-B3-9 | AT3G23220 | IX |
| Vitvi07g02064 | | GSVIVP00007518001 | VvERF097 | VvERF-B3-10 | AT3G23230 | IX |
| Vitvi07g00590 | | GSVIVP00007518001 | VvERF097 | VvERF-B3-10 | AT3G23230 | IX |
| Vitvi07g02070 | | GSVIVP00007519001 | VvERF098 | VvERF-B3-13 | AT3G23230 | IX |
| Vitvi07g02067 | | GSVIVP00007520001 | VvERF099 | VvERF-B3-5 | AT3G23240 | IX |
| Vitvi07g02065 | | GSVIVP00007523001 | VvERF100 | VvERF-B3-6 | AT3G23240 | IX |
| NA |  | GIDVvP00014072001 | VvERF101 | VvERF-B3-4 | AT3G23240 | IX |
| NA |  | GIDVvP00014075001 | VvERF102 | VvERF-B3-7 | AT3G23240 | IX |
| Vitvi07g02068 | g27334, g27335 | GSVIVP00007525001 | VvERF103 | VvERF-B3-3 | AT3G23240 | IX |
| NA |  | GSVIVP00014242001 | VvERF104 | VvERF-B3-26 | AT3G23230 | IX |
| Vitvi16g01424 | | GSVIVP00014247001 | VvERF105 | VvERF-B3-25 | AT3G23230 | IX |
| Vitvi16g00360 | | GSVIVP00014248001 | VvERF106 | VvERF-B3-24 | AT3G23230 | IX |
| Vitvi16g00362 | | GSVIVP00014250001 | VvERF107 | VvERF-B3-31 | AT3G23230 | IX |
| Vitvi16g00363 | | GSVIVP00014253001 | VvERF108 | VvERF-B3-30 | AT3G23230 | IX |
| Vitvi05g01722 | | GSVIVP00020218001 | VvERF109 | VvERF-B3-11 | AT3G23220 | IX |
| Vitvi05g01723 | | GSVIVP00020219001 | VvERF110 | VvERF-B3-12 | AT3G23230 | IX |
| Vitvi05g00715 | | GSVIVP00020221001 | VvERF111 | VvERF-B3-1 | AT3G23240 | IX |
| Vitvi01g01826 | | GSVIVP00006201001 | VvERF112 | VvERF-B4-4 | AT5G13330 | X |
| Vitvi07g01874 | | GSVIVP00019350001 | VvERF113 | VvERF-B4-1 | AT5G64750 | X |
| Vitvi18g01617 | | GSVIVP00031737001 | VvERF114 | VvERF-B4-2 | AT1G43160 | X |
| NA |  | GIDVvP00034511001 | VvERF115 | VvERF-B6-14 | AT4G34410 | X |
| NA |  | GIDVvP00034505001 | VvERF116 | VvERF-B6-16 | AT4G34410 | X |
| Vitvi03g00500 | | GSVIVP00036589001 | VvERF117 | VvERF-B6-17 | AT4G34410 | X |
| NA |  | GIDVvP00031111001 | VvERF118 |  | AT2G33710 | X |
| NA |  | GIDVvP00034507001 | VvERF119 | VvERF-B6-15 | AT2G33710 | X |
| Vitvi14g01458 | g30040 | GSVIVP00037958001 | VvERF120 | VvERF-B4-3 | AT2G33710 | X |
| NA |  | GIDVvP00030565001 | VvERF121 | #N/A | AT1G68550 | VI-L |
| Vitvi01g01827 | | GSVIVP00030332001 | VvERF122 | VvERF-B6-18 | AT1G68550 | VI-L |
| Vitvi11g00798 | g12372 | GSVIVP00017130001 | VvAP2-01 | VvAP2-2 | AT1G16060 | AP2 |
| Vitvi14g01902 | g27006, g3645 | GSVIVP00020889001 | VvAP2-02 | VvAP2-12 | AT3G54320 | AP2 |
| Vitvi06g00187 | | GSVIVP00024643001 | VvAP2-03 | VvAP2-11 | AT1G51190 | AP2 |
| Vitvi04g02088 | | GSVIVP00025028001 | VvAP2-04 | VvAP2-16 | AT3G20840 | AP2 |
| Vitvi04g01207 | | GSVIVP00025029001 | VvAP2-05 | VvAP2-10 | AT5G17430 | AP2 |
| Vitvi09g01269 | | GSVIVP00025239001 | VvAP2-06 | VvAP2-6 | AT1G16060 | AP2 |
| Vitvi08g01861 | g17793 | GSVIVP00025534001 | VvAP2-07 | VvAP2-15 | AT1G51190 | AP2 |
| Vitvi01g00934 | g33989 | GSVIVP00028999001 | VvAP2-08 | VvAP2-5 | AT3G54320 | AP2 |
| Vitvi11g01231 | g1130, g31195 | GSVIVP00031356001 | VvAP2-09 | VvAP2-9 | AT5G57390 | AP2 |
| Vitvi09g00108 | g31083 | GSVIVP00034094001 | VvAP2-10 | VvAP2-4 | AT1G72570 | AP2 |
| Vitvi13g00529 | g17077, g19616, g20530, g24789 | GSVIVP00018277001 | VvAP2-11 | VvAP2-1 | AT4G36920 | AP2 |
| Vitvi06g00360 | | GSVIVP00024452001 | VvAP2-12 |  | AT2G28550 | AP2 |
| Vitvi07g01706 | | GSVIVP00019538001 | VvAP2-13 | VvAP2-13 | AT4G36920 | AP2 |
| Vitvi08g01146 | | GSVIVP00023069001 | VvAP2-14 | VvAP2-8 | AT4G36920 | AP2 |
| Vitvi06g00360 | | GSVIVP00024453001 | VvAP2-15 | VvAP2-7 | AT4G36920 | AP2 |
| Vitvi07g01421 | g16916 | GSVIVP00011815001 | VvAP2-16 | VvAP2-3 | AT5G10510 | AP2 |
| Vitvi07g01419 | | GSVIVP00011815001 | VvAP2-16 | VvAP2-3 | AT5G10510 | AP2 |
| NA |  | GSVIVP00012204001 | VvAP2-17 |  | AT4G37750 | AP2 |
| NA |  | GSVIVP00012449001 | VvAP2-18 | VvAP2-18 | AT4G37750 | AP2 |
| Vitvi18g00618 | g14510, g17932, g20687 | GSVIVP00014997001 | VvAP2-19 | VvAP2-14 | AT4G37750 | AP2 |
| Vitvi13g01862 | | GSVIVP00033924001 | VvAP2-20 | VvAP2-17 | AT3G54320 | AP2 |
| Vitvi01g00244 | | GSVIVP00030292001 | VvRAV1 | VvRAV-4 | AT1G68840 | RAV |
| Vitvi14g01248 | | GSVIVP00038173001 | VvRAV2 | VvRAV-3 | AT1G68840 | RAV |
| Vitvi02g00275 | | GSVIVP00000473001 | VvRAV3 |  | AT2G46870 | RAV |
| Vitvi15g00863 | | GSVIVP00026190001 | VvRAV4 | VvRAV-1 | AT2G46871 | RAV |
| Vitvi08g01412 | | GSVIVP00021555001 | VvRAV5 |  | AT2G36080 | RAV |
| Vitvi11g00678 | | GSVIVP00017239001 | VvRAV6 | VvRAV-2 | AT1G51120 | RAV |
| NA |  | GIDVvP00018355001 | VvSoloist | Soloist | AT4G13040 | Soloist |

**Supplementary File 7f. *V. riparia* ‘Manitoba 37’ and *V. vinifera* ‘PN40024’ members of the “retrotrans_gag 2” family (LTR retrotransposons).**

| **V. riparia** | |  | **V. vinifera** |
| --- | --- | --- | --- |
| g510.t1cs |  | | Vitvi07g02836.t01cs |
| g513.t1cs |  | | Vitvi07g02824.t01cs |
| g519.t1cs |  | | Vitvi07g03015.t01cs |
| g1306.t1cs | | | Vitvi07g03005.t01cs |
| g2252.t1cs | | | Vitvi02g01772.t01cs |
| g2854.t1cs | | | Vitvi18g03291.t01cs |
| g3191.t1cs | | | Vitvi10g02091.t01cs |
| g3453.t1cs | | | Vitvi07g02740.t01cs |
| g3454.t1cs | | | Vitvi07g03191.t01cs |
| g4132.t1cs | | | Vitvi02g01787.t01cs |
| g4184.t1cs | | | Vitvi14g03109.t01cs |
| g4488.t1cs | | | Vitvi07g03078.t01cs |
| g4508.t1cs | | | Vitvi00g01823.t01cs |
| g5099.t1cs | | | Vitvi14g03119.t01cs |
| g5644.t1cs | | | Vitvi07g03038.t01cs |
| g5649.t1cs | | | Vitvi07g03037.t01cs |
| g6004.t1cs | | | Vitvi19g02362.t01cs |
| g6027.t1cs | | | Vitvi10g02156.t01cs |
| g6049.t1cs | | | Vitvi07g02939.t01cs |
| g6195.t1cs | | | Vitvi18g03330.t01cs |
| g6304.t1cs | | | Vitvi15g01717.t01cs |
| g6313.t1cs | | | Vitvi15g01744.t01cs |
| g6316.t1cs | | | Vitvi14g03135.t01cs |
| g6332.t1cs | | | Vitvi02g01781.t01cs |
| g6432.t1cs | | | Vitvi02g01679.t01cs |
| g6463.t1cs | | | Vitvi00g02189.t01cs |
| g6466.t1cs | | | Vitvi00g02194.t01cs |
| g6691.t1cs | | | Vitvi00g02204.t01cs |
| g6952.t1cs | | | Vitvi00g01182.t01cs |
| g7036.t1cs | | | Vitvi00g01213.t01cs |
| g7100.t1cs | | | Vitvi00g02262.t01cs |
| g7120.t1cs | | | Vitvi00g02316.t01cs |
| g7212.t1cs | | | Vitvi10g02421.t01cs |
| g7215.t1cs | | | Vitvi00g02351.t01cs |
| g7216.t1cs | | | Vitvi00g02354.t01cs |
| g7236.t1cs | | | Vitvi01g01909.t01cs |
| g7237.t1cs | | | Vitvi01g01940.t01cs |
| g7673.t1cs | | | Vitvi01g00622.t01cs |
| g7675.t1cs | | | Vitvi01g00804.t01cs |
| g7677.t1cs | | | Vitvi01g02151.t01cs |
| g7754.t1cs | | | Vitvi01g01224.t01cs |
| g7886.t1cs | | | Vitvi01g01358.t01cs |
| g7888.t1cs | | | Vitvi01g02181.t01cs |
| g8103.t1cs | | | Vitvi01g02190.t01cs |
| g8189.t1cs | | | Vitvi01g02201.t01cs |
| g8370.t1cs | | | Vitvi01g02211.t01cs |
| g8371.t1cs | | | Vitvi01g02244.t01cs |
| g8484.t1cs | | | Vitvi01g01702.t01cs |
| g8608.t1cs | | | Vitvi02g01402.t01cs |
| g8614.t1cs | | | Vitvi02g00492.t01cs |
| g8627.t1cs | | | Vitvi02g01497.t01cs |
| g8647.t1cs | | | Vitvi02g01511.t01cs |
| g8724.t1cs | | | Vitvi02g01535.t01cs |
| g8754.t1cs | | | Vitvi02g01629.t01cs |
| g8786.t1cs | | | Vitvi02g01668.t01cs |
| g9047.t1cs | | | Vitvi03g01321.t01cs |
| g9115.t1cs | | | Vitvi03g01720.t01cs |
| g9198.t1cs | | | Vitvi03g01125.t01cs |
| g9299.t1cs | | | Vitvi03g01826.t01cs |
| g9425.t1cs | | | Vitvi03g01837.t01cs |
| g9554.t1cs | | | Vitvi03g01867.t01cs |
| g9844.t1cs | | | Vitvi04g01815.t01cs |
| g9994.t1cs | | | Vitvi04g01816.t01cs |
| g10085.t1cs | | | Vitvi04g00426.t01cs |
| g10138.t1cs | | | Vitvi04g01921.t01cs |
| g10185.t1cs | | | Vitvi04g01931.t01cs |
| g10269.t1cs | | | Vitvi04g00638.t01cs |
| g10456.t1cs | | | Vitvi04g01947.t01cs |
| g10506.t1cs | | | Vitvi04g01971.t01cs |
| g10631.t1cs | | | Vitvi04g01987.t01cs |
| g10632.t1cs | | | Vitvi04g01996.t01cs |
| g10911.t1cs | | | Vitvi04g00822.t01cs |
| g11189.t1cs | | | Vitvi04g00823.t01cs |
| g11285.t1cs | | | Vitvi04g02045.t01cs |
| g11302.t1cs | | | Vitvi04g01070.t01cs |
| g11360.t1cs | | | Vitvi04g02047.t01cs |
| g11388.t1cs | | | Vitvi04g02048.t01cs |
| g11454.t1cs | | | Vitvi04g02079.t01cs |
| g11455.t1cs | | | Vitvi04g02115.t01cs |
| g11795.t1cs | | | Vitvi04g01344.t01cs |
| g12112.t1cs | | | Vitvi04g01648.t01cs |
| g12294.t1cs | | | Vitvi04g01650.t01cs |
| g12333.t1cs | | | Vitvi05g00525.t01cs |
| g12442.t1cs | | | Vitvi05g00773.t01cs |
| g12812.t1cs | | | Vitvi05g00856.t01cs |
| g13392.t1cs | | | Vitvi05g02023.t01cs |
| g13913.t1cs | | | Vitvi05g02024.t01cs |
| g13921.t1cs | | | Vitvi05g02084.t01cs |
| g13978.t1cs | | | Vitvi05g02085.t01cs |
| g14236.t1cs | | | Vitvi05g02088.t01cs |
| g14704.t1cs | | | Vitvi05g02096.t01cs |
| g15417.t1cs | | | Vitvi05g02101.t01cs |
| g15417.t2cs | | | Vitvi05g02220.t01cs |
| g15477.t1cs | | | Vitvi05g02261.t01cs |
| g15565.t1cs | | | Vitvi05g02265.t01cs |
| g15585.t2cs | | | Vitvi05g01713.t01cs |
| g15639.t1cs | | | Vitvi06g00003.t01cs |
| g16186.t1cs | | | Vitvi06g00711.t01cs |
| g16834.t1cs | | | Vitvi06g01831.t01cs |
| g16908.t1cs | | | Vitvi06g01849.t01cs |
| g17226.t1cs | | | Vitvi06g01857.t01cs |
| g17526.t1cs | | | Vitvi06g01967.t01cs |
| g18083.t1cs | | | Vitvi06g01981.t01cs |
| g18121.t1cs | | | Vitvi07g02175.t01cs |
| g18301.t1cs | | | Vitvi07g02293.t01cs |
| g18302.t1cs | | | Vitvi07g00712.t01cs |
| g18544.t1cs | | | Vitvi07g00859.t01cs |
| g18590.t1cs | | | Vitvi07g00864.t01cs |
| g18591.t1cs | | | Vitvi07g02359.t01cs |
| g18910.t1cs | | | Vitvi07g02376.t01cs |
| g19180.t1cs | | | Vitvi07g02406.t01cs |
| g19498.t1cs | | | Vitvi07g02423.t01cs |
| g19499.t1cs | | | Vitvi07g02454.t01cs |
| g19608.t1cs | | | Vitvi07g02468.t01cs |
| g20022.t1cs | | | Vitvi07g02472.t01cs |
| g20039.t1cs | | | Vitvi07g02503.t01cs |
| g20831.t1cs | | | Vitvi07g02709.t01cs |
| g21149.t1cs | | | Vitvi07g02715.t01cs |
| g21360.t1cs | | | Vitvi08g01976.t01cs |
| g21377.t1cs | | | Vitvi08g00040.t01cs |
| g21490.t1cs | | | Vitvi08g02012.t01cs |
| g21782.t1cs | | | Vitvi08g00238.t01cs |
| g22416.t1cs | | | Vitvi08g02054.t01cs |
| g22717.t1cs | | | Vitvi08g00706.t01cs |
| g22870.t1cs | | | Vitvi08g02073.t01cs |
| g22924.t1cs | | | Vitvi08g00920.t01cs |
| g23738.t1cs | | | Vitvi08g02127.t01cs |
| g23739.t1cs | | | Vitvi08g02178.t01cs |
| g23911.t1cs | | | Vitvi08g01647.t01cs |
| g24047.t1cs | | | Vitvi08g02400.t01cs |
| g24047.t2cs | | | Vitvi09g01498.t01cs |
| g24551.t1cs | | | Vitvi09g00101.t01cs |
| g24577.t1cs | | | Vitvi09g01531.t01cs |
| g24599.t1cs | | | Vitvi09g00472.t01cs |
| g24613.t1cs | | | Vitvi09g01726.t01cs |
| g25075.t1cs | | | Vitvi09g00798.t01cs |
| g25076.t1cs | | | Vitvi09g01745.t01cs |
| g25757.t2cs | | | Vitvi09g01747.t01cs |
| g26332.t1cs | | | Vitvi09g01785.t01cs |
| g26339.t1cs | | | Vitvi09g00989.t01cs |
| g27133.t1cs | | | Vitvi09g01015.t01cs |
| g27233.t1cs | | | Vitvi09g01839.t01cs |
| g27341.t1cs | | | Vitvi09g01846.t01cs |
| g27358.t1cs | | | Vitvi09g01878.t01cs |
| g27676.t1cs | | | Vitvi09g01906.t01cs |
| g27716.t1cs | | | Vitvi09g01907.t01cs |
| g28249.t1cs | | | Vitvi09g01952.t01cs |
| g28595.t1cs | | | Vitvi09g01347.t01cs |
| g28717.t1cs | | | Vitvi09g01964.t01cs |
| g28987.t1cs | | | Vitvi09g01976.t01cs |
| g28997.t1cs | | | Vitvi09g01997.t01cs |
| g29265.t1cs | | | Vitvi10g01604.t01cs |
| g30382.t1cs | | | Vitvi10g01802.t01cs |
| g30757.t1cs | | | Vitvi10g01805.t01cs |
| g30782.t1cs | | | Vitvi10g01815.t01cs |
| g31255.t1cs | | | Vitvi10g01843.t01cs |
| g31437.t1cs | | | Vitvi10g01855.t01cs |
| g31627.t1cs | | | Vitvi10g00974.t01cs |
| g31638.t1cs | | | Vitvi10g01954.t01cs |
| g32059.t1cs | | | Vitvi10g01382.t01cs |
| g32830.t1cs | | | Vitvi11g01352.t01cs |
| g32962.t1cs | | | Vitvi11g01395.t01cs |
| g33340.t1cs | | | Vitvi11g01434.t01cs |
| g33388.t1cs | | | Vitvi11g01515.t01cs |
| g33722.t1cs | | | Vitvi11g01535.t01cs |
| g33855.t1cs | | | Vitvi11g00876.t01cs |
| g34188.t1cs | | | Vitvi11g01598.t01cs |
| g34333.t1cs | | | Vitvi11g01600.t01cs |
| g34507.t1cs | | | Vitvi11g01603.t01cs |
| g34691.t1cs | | | Vitvi11g01612.t01cs |
| g34764.t1cs | | | Vitvi11g01616.t01cs |
| g34829.t1cs | | | Vitvi12g02287.t01cs |
| g34880.t1cs | | | Vitvi12g02290.t01cs |
| g34895.t1cs | | | Vitvi12g02422.t01cs |
| g35116.t1cs | | | Vitvi12g02513.t01cs |
| g35120.t1cs | | | Vitvi12g02533.t01cs |
| g35125.t1cs | | | Vitvi12g00998.t01cs |
| g35150.t1cs | | | Vitvi12g01198.t01cs |
| g35500.t1cs | | | Vitvi12g01536.t01cs |
| g35593.t1cs | | | Vitvi12g01727.t01cs |
| g35665.t1cs | | | Vitvi12g02673.t01cs |
| g35984.t1cs | | | Vitvi12g02696.t01cs |
| g36019.t1cs | | | Vitvi12g02717.t01cs |
| g36305.t1cs | | | Vitvi12g02749.t01cs |
| g36349.t1cs | | | Vitvi12g02764.t01cs |
| g36427.t1cs | | | Vitvi13g00039.t01cs |
| g36524.t1cs | | | Vitvi13g01993.t01cs |
| g36673.t1cs | | | Vitvi13g02011.t01cs |
| g37175.t1cs | | | Vitvi13g00460.t01cs |
| g37198.t1cs | | | Vitvi13g00760.t01cs |
| g37377.t1cs | | | Vitvi13g00807.t01cs |
| g37381.t1cs | | | Vitvi13g00819.t01cs |
| g37483.t1cs | | | Vitvi13g00824.t01cs |
| g37730.t1cs | | | Vitvi13g00833.t01cs |
| g37844.t1cs | | | Vitvi13g01005.t01cs |
| g37897.t1cs | | | Vitvi13g01019.t01cs |
| g37905.t1cs | | | Vitvi13g02202.t01cs |
| g37930.t1cs | | | Vitvi13g01192.t01cs |
| g14.t1cs |  | | Vitvi13g02242.t01cs |
| g16.t1cs |  | | Vitvi13g02249.t01cs |
| g520.t1cs |  | | Vitvi13g02265.t01cs |
| g939.t1cs |  | | Vitvi13g01452.t01cs |
| g1770.t1cs | | | Vitvi13g01503.t01cs |
| g2371.t1cs | | | Vitvi13g01506.t01cs |
| g2373.t1cs | | | Vitvi13g01520.t01cs |
| g3577.t1cs | | | Vitvi13g02432.t01cs |
| g3581.t1cs | | | Vitvi13g02436.t01cs |
| g3605.t1cs | | | Vitvi13g02455.t01cs |
| g3684.t1cs | | | Vitvi13g02533.t01cs |
| g4413.t1cs | | | Vitvi14g02441.t01cs |
| g4504.t1cs | | | Vitvi14g02450.t01cs |
| g4694.t1cs | | | Vitvi14g02694.t01cs |
| g4695.t1cs | | | Vitvi14g02698.t01cs |
| g5011.t1cs | | | Vitvi14g02730.t01cs |
| g8402.t1cs | | | Vitvi14g00711.t01cs |
| g10399.t1cs | | | Vitvi14g02766.t01cs |
| g11168.t1cs | | | Vitvi14g01143.t01cs |
| g13958.t1cs | | | Vitvi14g02883.t01cs |
| g16120.t1cs | | | Vitvi14g02921.t01cs |
| g23181.t1cs | | | Vitvi14g02975.t01cs |
| g23400.t1cs | | | Vitvi14g02976.t01cs |
| g24208.t1cs | | | Vitvi14g02978.t01cs |
| g24396.t1cs | | | Vitvi14g03014.t01cs |
| g25779.t1cs | | | Vitvi14g03064.t01cs |
| g25918.t1cs | | | Vitvi14g03067.t01cs |
| g27229.t1cs | | | Vitvi15g00043.t01cs |
| g27230.t1cs | | | Vitvi15g01246.t01cs |
| g27700.t1cs | | | Vitvi15g00093.t01cs |
| g28321.t1cs | | | Vitvi15g01250.t01cs |
| g29294.t1cs | | | Vitvi15g01260.t01cs |
|  |  | | Vitvi15g00130.t01cs |
|  |  | | Vitvi15g00140.t01cs |
|  |  | | Vitvi15g00164.t01cs |
|  |  | | Vitvi15g01292.t01cs |
|  |  | | Vitvi15g01367.t01cs |
|  |  | | Vitvi15g01373.t01cs |
|  |  | | Vitvi15g01398.t01cs |
|  |  | | Vitvi15g00442.t01cs |
|  |  | | Vitvi15g00446.t01cs |
|  |  | | Vitvi15g01412.t01cs |
|  |  | | Vitvi15g01479.t01cs |
|  |  | | Vitvi15g01484.t01cs |
|  |  | | Vitvi15g01492.t01cs |
|  |  | | Vitvi15g01533.t01cs |
|  |  | | Vitvi15g01599.t01cs |
|  |  | | Vitvi15g01652.t01cs |
|  |  | | Vitvi16g01533.t01cs |
|  |  | | Vitvi16g01551.t01cs |
|  |  | | Vitvi16g00200.t01cs |
|  |  | | Vitvi16g01565.t01cs |
|  |  | | Vitvi16g01574.t01cs |
|  |  | | Vitvi16g01617.t01cs |
|  |  | | Vitvi16g00425.t01cs |
|  |  | | Vitvi16g01686.t01cs |
|  |  | | Vitvi16g00464.t01cs |
|  |  | | Vitvi16g00550.t01cs |
|  |  | | Vitvi16g00585.t01cs |
|  |  | | Vitvi16g01747.t01cs |
|  |  | | Vitvi16g01760.t01cs |
|  |  | | Vitvi16g00678.t01cs |
|  |  | | Vitvi16g00728.t01cs |
|  |  | | Vitvi16g01792.t01cs |
|  |  | | Vitvi16g01807.t01cs |
|  |  | | Vitvi16g01814.t01cs |
|  |  | | Vitvi16g01829.t01cs |
|  |  | | Vitvi16g01862.t01cs |
|  |  | | Vitvi16g01869.t01cs |
|  |  | | Vitvi16g01047.t01cs |
|  |  | | Vitvi16g01255.t01cs |
|  |  | | Vitvi16g01300.t01cs |
|  |  | | Vitvi16g02049.t01cs |
|  |  | | Vitvi17g01450.t01cs |
|  |  | | Vitvi17g00717.t01cs |
|  |  | | Vitvi17g01558.t01cs |
|  |  | | Vitvi17g00992.t01cs |
|  |  | | Vitvi17g01603.t01cs |
|  |  | | Vitvi17g01659.t01cs |
|  |  | | Vitvi17g01258.t01cs |
|  |  | | Vitvi18g02682.t01cs |
|  |  | | Vitvi18g01274.t01cs |
|  |  | | Vitvi18g02893.t01cs |
|  |  | | Vitvi18g02897.t01cs |
|  |  | | Vitvi18g02946.t01cs |
|  |  | | Vitvi18g02983.t01cs |
|  |  | | Vitvi18g01759.t01cs |
|  |  | | Vitvi18g01766.t01cs |
|  |  | | Vitvi18g01862.t01cs |
|  |  | | Vitvi18g01938.t01cs |
|  |  | | Vitvi18g02136.t01cs |
|  |  | | Vitvi18g03159.t01cs |
|  |  | | Vitvi19g01887.t01cs |
|  |  | | Vitvi19g01919.t01cs |
|  |  | | Vitvi19g00424.t01cs |
|  |  | | Vitvi19g02034.t01cs |
|  |  | | Vitvi19g02043.t01cs |
|  |  | | Vitvi19g00779.t01cs |
|  |  | | Vitvi19g02092.t01cs |
|  |  | | Vitvi19g02098.t01cs |
|  |  | | Vitvi19g00883.t01cs |
|  |  | | Vitvi19g00894.t01cs |
|  |  | | Vitvi19g02115.t01cs |
|  |  | | Vitvi19g01021.t01cs |
|  |  | | Vitvi19g01031.t01cs |
|  |  | | Vitvi19g01185.t01cs |
|  |  | | Vitvi19g01309.t01cs |
|  |  | | Vitvi19g02209.t01cs |
|  |  | | Vitvi19g02217.t01cs |
|  |  | | Vitvi19g02225.t01cs |
|  |  | | Vitvi19g02305.t01cs |
|  |  | | Vitvi19g02318.t01cs |
|  |  | | Vitvi19g02352.t01cs |
|  |  | | Vitvi19g02354.t01cs |
|  |  | | Vitvi19g01766.t01cs |

**g. *V. riparia* ‘Manitoba 37’ and *V. vinifera* ‘PN40024’ *LBD* proteins.**

| **Class** | **Gene** | ***Species*** |
| --- | --- | --- |
| 1a | Vitvi07g00572.t01 | *V. vinifera* |
| 1a | Vitvi13g00333.t01 | *V. vinifera* |
| 1a | Vitvi08g00144.t01 | *V. vinifera* |
| 1a | Vitvi07g00573.t01 | *V. vinifera* |
| 1a | Vitvi15g00736.t01 | *V. vinifera* |
| 1a | Vitvi15g00735.t01 | *V. vinifera* |
| 1a | g11488.t1 | *V. riparia* |
| 1a | g1654.t1 | *V. riparia* |
| 1a | g19201.t1 | *V. riparia* |
| 1a | g11487.t1 | *V. riparia* |
| 1a | g21903.t1 | *V. riparia* |
| 1a | g21904.t1 | *V. riparia* |
| 1a | g29038.t1 | *Seedless* |
| 1a | g16409.t1 | *Seedless* |
| 1a | g15437.t1 | *Seedless* |
| 1a | Q9SLB6 | *Arabidopsis* |
| 1a | Q9M2J7 | *Arabidopsis* |
| 1a | Q9SJW5 | *Arabidopsis* |
| 1a | Q9LHS8 | *Arabidopsis* |
| 1a | Q9SRV3 | *Arabidopsis* |
| 1a | Q9SLB7 | *Arabidopsis* |
| 1a | OB1322 | *Arabidopsis* |
| 1a | O22132 | *Arabidopsis* |
| 1a | O22131 | *Arabidopsis* |
| 1a | O81323 | *Arabidopsis* |
| 2b | Vitvi07g01610.t01 | *V. vinifera* |
| 2b | Vitvi18g00677.t01 | *V. vinifera* |
| 2b | g20627.t1 | *V. riparia* |
| 2b | g17992.t1 | *V. riparia* |
| 2b | g23569.t1 | *Seedless* |
| 2b | g121.t1 | *Seedless* |
| 2b | Q9SZE8 | *Arabidopsis* |
| 2b | Q9SN23 | *Arabidopsis* |
| 2b | Q9FN11 | *Arabidopsis* |
| 1c | Vitvi07g01326.t01 | *V. vinifera* |
| 1c | Vitvi16g00859.t01 | *V. vinifera* |
| 1c | Vitvi07g01327.t01 | *V. vinifera* |
| 1c | Vitvi0701328.t01 | *V. vinifera* |
| 1c | Vitvi00g1060.t01 | *V. vinifera* |
| 1c | Vitvi17g00890.t01 | *V. vinifera* |
| 1c | Vitvi14g01707.t01 | *V. vinifera* |
| 1c | g14734.t1 | *V. riparia* |
| 1c | g14875.t1 | *V. riparia* |
| 1c | g14732.t1 | *V. riparia* |
| 1c | g14730.t1 | *V. riparia* |
| 1c | g28417.t1 | *V. riparia* |
| 1c | g28417.t2 | *V. riparia* |
| 1c | g24103.t1 | *V. riparia* |
| 1c | Q9FML4 | *Arabidopsis* |
| 1c | Q8L8Q3 | *Arabidopsis* |
| 1d | g26521.t1 | *V. riparia* |
| 1d | g35636.t1 | *V. riparia* |
| 1d | g36020.t1 | *V. riparia* |
| 1d | g32479.t1 | *V. riparia* |
| 1d | g9041.t1 | *V. riparia* |
| 1d | g19527.t1 | *V. riparia* |
| 1d | g26499.t1 | *V. riparia* |
| 1d | g1154.t1 | *V. riparia* |
| 1d | Vitvi10g01237.t01 | *V. riparia* |
| 1d | Vitvi19g01589.t01 | *V. riparia* |
| 1d | Vitvi16g01446.t01 | *V. riparia* |
| 1d | Vitvi13g00144.t01 | *V. riparia* |
| 1d | Vitvi06g00772.t01 | *V. riparia* |
| 1d | Vitvi13g00085.t01 | *V. riparia* |
| 1d | Vitvi06g00706.t01 | *V. riparia* |
| 1d | Vitvi13g00109.t01 | *V. riparia* |
| 1d | g16183.t1 | *Seedless* |
| 1d | g3720.t1 | *Seedless* |
| 1d | g11475.t1 | *Seedless* |
| 1d | g3637.t1 | *Seedless* |
| 1d | g21593.t1 | *Seedless* |
| 1d | g1006.t1 | *Seedless* |
| 1d | g14432.t1 | *Seedless* |
| 1d | g2068.t1 | *Seedless* |
| 1d | Q9SHE9 | *Arabidopsis* |
| 1d | P59468 | *Arabidopsis* |
| 1d | P59467 | *Arabidopsis* |
| 1d | Q9SA51 | *Arabidopsis* |
| 1d | Q8L5T5 | *Arabidopsis* |
| 1d | Q9AT61 | *Arabidopsis* |
| 1d | Q9SRL8 | *Arabidopsis* |
| 1d | Q8LBW3 | *Arabidopsis* |
| 1f | Vitvi13g00552.t01 | *V. vinifera* |
| 1f | Vitvi13g00559.t01 | *V. vinifera* |
| 1f | Vitvi14g00555.t01 | *V. vinifera* |
| 1f | Vitvi13g00551.t01 | *V. vinifera* |
| 1f | Vitvi13g00549.t01 | *V. vinifera* |
| 1f | Vitvi13g00545.t01 | *V. vinifera* |
| 1f | Vitvi13g00546.t01 | *V. vinifera* |
| 1f | Vitvi13g00556 | *V. vinifera* |
| 1f | Vitvi06g00336.t01 | *V. vinifera* |
| 1f | Vitvi06g00338.t01 | *V. vinifera* |
| 1f | Vitvi13g00543.t01 | *V. vinifera* |
| 1f | Vitvi13g01867.t01 | *V. vinifera* |
| 1f | Vitvi13g01866.t01 | *V. vinifera* |
| 1f | g26753.t1 | *V. riparia* |
| 1f | g36203.t1 | *V. riparia* |
| 1f | g36202.t1 | *V. riparia* |
| 1f | g36201.t1 | *V. riparia* |
| 1f | g17061.t1 | *V. riparia* |
| 1f | g17060.t1 | *V. riparia* |
| 1f | g31875.t1 | *V. riparia* |
| 1f | g31875.t1 | *V. riparia* |
| 1f | g31876.t1 | *V. riparia* |
| 1f | g17062.t1 | *V. riparia* |
| 1f | g36206.t1 | *V. riparia* |
| 1f | g36205.t1 | *V. riparia* |
| 1f | g6161.t1 | *Seedless* |
| 1f | g6160.t1 | *Seedless* |
| 1f | g6159.t1 | *Seedless* |
| 1f | g11037.t1 | *Seedless* |
| 1f | g11036.t1 | *Seedless* |
| 1f | g28521.t1 | *Seedless* |
| 1f | g4490.t1 | *Seedless* |
| 1f | g17074.t1 | *Seedless* |
| 1f | g17072.t1 | *Seedless* |
| 1f | g28169.t1 | *Seedless* |
| 1f | g11038.t1 | *Seedless* |
| 1f | g7214.t1 | *Seedless* |
| 1f | g7215.t1 | *Seedless* |
| 1f | Q9SK08 | *Arabidopsis* |
| 1f | Q9LQR0 | *Arabidopsis* |
| 1h | Q9FFL3 | *Arabidopsis* |
| 1h | Q49651 | *Arabidopsis* |
| 1h | Q9LIJ0 | *Arabidopsis* |
| 1h | Q64836 | *Arabidopsis* |
| 1h | Q9SCS4 | *Arabidopsis* |
| 1i | Vitvi17g00520.t01 | *V. vinifera* |
| 1i | Vitvi14g01878.t01 | *V. vinifera* |
| 1i | Vitvi04g01768.t01 | *V. vinifera* |
| 1i | Vitvi0900188.t01 | *V. vinifera* |
| 1i | Vitvi11g00169.t01 | *V. vinifera* |
| 1i | Vitvi12g00230.t01 | *V. vinifera* |
| 1i | Vitvi15g01216.t01 | *V. vinifera* |
| 1i | Vitvi15g01216.t01 | *V. vinifera* |
| 1i | Vitvi15g01217.t01 | *V. vinifera* |
| 1i | g32196.t1 | *V. riparia* |
| 1i | g26979.t1 | *V. riparia* |
| 1i | g3352.t1 | *V. riparia* |
| 1i | g38289.t1 | *V. riparia* |
| 1i | g3231.t1 | *V. riparia* |
| 1i | g5394.t1 | *V. riparia* |
| 1i | g26754.t1 | *V. riparia* |
| 1i | g13010.t1 | *Seedless* |
| 1i | g3004.t1 | *Seedless* |
| 1i | g19404.t1 | *Seedless* |
| 1i | g2507.t1 | *Seedless* |
| 1i | g17685.t1 | *Seedless* |
| 1i | g6157.t1 | *Seedless* |
| 1i | g6156.t1 | *Seedless* |
| 1i | Q9STS6 | *Arabidopsis* |
| 1i | P59469 | *Arabidopsis* |
| 1i | Q9LRW1 | *Arabidopsis* |
| 1i | Q9SSM9 | *Arabidopsis* |
| 1i | Q9ZUP0 | *Arabidopsis* |
| 1i | Q9C8V8 | *Arabidopsis* |
| 1i | O82198 | *Arabidopsis* |
| 1i | Q9LNB9 | *Arabidopsis* |
| 2a | Vitvi14g01193.t01 | *V. vinifera* |
| 2a | Vitvi01g00291.t01 | *V. vinifera* |
| 2a | Vitvi17g00325.t01 | *V. vinifera* |
| 2a | Vitvi01g00290.t01 | *V. vinifera* |
| 2a | g18175.t1 | *V. riparia* |
| 2a | g16006.t1 | *V. riparia* |
| 2a | g11752.t1 | *V. riparia* |
| 2a | g16007.t1 | *V. riparia* |
| 2a | g7453.t1 | *Seedless* |
| 2a | g1732.t1 | *Seedless* |
| 2a | g11265.t1 | *Seedless* |
| 2a | g1731.t1 | *Seedless* |
| 2a | Q9ZW96 | *Arabidopsis* |
| 2a | Q9M886 | *Arabidopsis* |
| 2a | Q9CA30 | *Arabidopsis* |
| 2a | Vitvi03g00628.t01 | *V. vinifera* |
| 2a | g18278.t1 | *V. riparia* |
| 2a | g925.t1 | *Seedless* |
| Unclassified | Vitvi07g02993.t01 | *V. vinifera* |
| Unclassified | g14839.t1 | *V. riparia* |
| Unclassified | O04479 | *Arabidopsis* |
| Unclassified | Q9FKZ3 | *Arabidopsis* |
