# Supplementary material for: Draft genome of the Native American cold hardy grapevine Vitis riparia Michx. ‘Manitoba 37’
Source: Hortic Res. 2020 Jun 1;7:92. doi: 10.1038/s41438-020-0316-2 (PMC7261805; doi:10.1038/s41438-020-0316-2)
Supplement: Supplementary file 13 — Supplementary Table 8 [file 41438_2020_316_MOESM13_ESM.docx]

**Supplementary Table 8. Mapping of GBS markers for F2 marker population on *V. vinifera* PN40024, 12X.2 and *V. riparia* ‘Manitoba 37’.**

| *V. vinifera* | Total Aligned Markers | *V. riparia* | | | Total Aligned Markers | Common Markers | *V. vinifera* Markers | *V. riparia* Markers |
| --- | --- | --- | --- | --- | --- | --- | --- | --- |
| **chr1** | 784 | | **chr1** | 676 | | 613 | 171 | 63 |
| **chr2** | 707 | | **chr2** | 860 | | 671 | 36 | 189 |
| **chr3** | 721 | | **chr3** | 701 | | 653 | 68 | 48 |
| **chr4** | 1295 | | **chr4** | 1205 | | 1081 | 214 | 124 |
| **chr5** | 1224 | | **chr5** | 1106 | | 881 | 343 | 225 |
| **chr6** | 944 | | **chr6** | 911 | | 680 | 264 | 231 |
| **chr7** | 1093 | | **chr7** | 1076 | | 957 | 136 | 119 |
| **chr8** | 1108 | | **chr8** | 1186 | | 959 | 149 | 227 |
| **chr9** | 944 | | **chr9** | 993 | | 758 | 186 | 235 |
| **chr10** | 501 | | **chr10** | 646 | | 423 | 78 | 223 |
| **chr11** | 715 | | **chr11** | 637 | | 563 | 152 | 74 |
| **chr12** | 886 | | **chr12** | 1026 | | 851 | 35 | 175 |
| **chr13** | 1160 | | **chr13** | 873 | | 763 | 397 | 110 |
| **chr14** | 1024 | | **chr14** | 933 | | 702 | 322 | 231 |
| **chr15** | 242 | | **chr15** | 504 | | 172 | 70 | 332 |
| **chr16** | 828 | | **chr16** | 821 | | 770 | 58 | 51 |
| **chr17** | 842 | | **chr17** | 949 | | 783 | 59 | 166 |
| **chr18** | 1308 | | **chr18** | 1505 | | 1265 | 43 | 240 |
| **chr19** | 739 | | **chr19** | 809 | | 611 | 128 | 198 |
| **chrUn** | 0 | | **chr20** | 6 | | 0 | 0 | 6 |
| **NA** | NA | | **chr21** | 2 | | NA | NA | 2 |
