# Supplementary material for: Draft genome of the Native American cold hardy grapevine Vitis riparia Michx. ‘Manitoba 37’
Source: Hortic Res. 2020 Jun 1;7:92. doi: 10.1038/s41438-020-0316-2 (PMC7261805; doi:10.1038/s41438-020-0316-2)
Supplement: Supplementary file 14 — Supplementary Table 9 [file 41438_2020_316_MOESM14_ESM.docx]

**Supplementary Table 9a. Flower type QTL gene id and PFAM name for V. riparia and V. vinifera.**

| ***V. riparia*** | |  |  |  |  |
| --- | --- | --- | --- | --- | --- |
|  | **PFAM** | **SeqID** | **PFAM_NAME** | **DOM_NAME** | **BRIEF NAME** |
| 1 | PF13649 | g15893.t1 | PrmA | Methyltransf_25 | PrmA |
| 2 | PF01230 | g15894.t1 | DcpS_C | HIT | DcpS_C |
| 3 | PF00571 | g15895.t1 | NA | CBS | CBS |
| 4 | PF14541 | g15896.t1 | Asp | TAXi_C | Asp |
| 5 | PF00650 | g15897.t1 | HSP20 | CRAL_TRIO | HSP20 |
| 6 | PF14559 | g15898.t1 | HSP20 | TPR_19 | HSP20 |
| 7 | PF00403 | g15899.t1 | Ribosomal_L23eN | HMA | Ribosomal_L23eN |
| 8 | PF10197 | g15900.t1 | NA | Cir_N | Cir_N |
| 9 | PF04438 | g15901.t1 | NA | zf-HIT | zf-HIT |
| 10 | PF00248 | g15902.t1 | NA | Aldo_ket_red | Aldo_ket_red |
| 11 | PF00010 | g15903.t1 | NA | HLH | HLH |
| 12 | NA | g15904.t1 | NA | NA | g15904.t1 |
| 13 | PF02866 | g15905.t1 | Ldh_1_N | Ldh_1_C | Ldh_1_N |
| 14 | PF00561 | g15906.t1 | GRAS | Abhydrolase_1 | GRAS |
| 15 | PF02991 | g15907.t1 | APG12 | Atg8 | APG12 |
| 16 | PF01266 | g15908.t1 | SE | DAO | SE |
| 17 | NA | g15909.t1 | tRNA_edit | NA | tRNA_edit |
| 18 | PF01266 | g15910.t1 | SE | DAO | SE |
| 19 | PF02866 | g15911.t1 | Ldh_1_N | Ldh_1_C | Ldh_1_N |
| 20 | PF01556 | g15912.t1 | NA | DnaJ_C | DnaJ_C |
| 21 | PF14547 | g15913.t1 | Tryp_alpha_amyl | Hydrophob_seed | Tryp_alpha_amyl |
| 22 | PF14547 | g15914.t1 | Tryp_alpha_amyl | Hydrophob_seed | Tryp_alpha_amyl |
| 23 | PF14547 | g15915.t1 | Tryp_alpha_amyl | Hydrophob_seed | Tryp_alpha_amyl |
| 24 | PF14547 | g15916.t1 | Tryp_alpha_amyl | Hydrophob_seed | Tryp_alpha_amyl |
| 25 | PF14547 | g15917.t1 | Tryp_alpha_amyl | Hydrophob_seed | Tryp_alpha_amyl |
| 26 | NA | g15918.t1 | PTR2 | NA | PTR2 |
| 27 | PF15413 | g15919.t1 | Oxysterol_BP | PH_11 | Oxysterol_BP |
| 28 | NA | g15920.t1 | PAP2 | NA | PAP2 |
| 29 | NA | g15921.t1 | MatE | NA | MatE |
| 30 | PF00156 | g15922.t1 | Gcd10p | Pribosyltran | Gcd10p |
| 31 | PF03106 | g15923.t1 | NA | WRKY | WRKY |
| 32 | NA | g15924.t1 | DUF863 | NA | DUF863 |
| 33 | PF07992 | g15925.t1 | FMO-like | Pyr_redox_2 | FMO-like |
| 34 | PF07992 | g15926.t1 | FMO-like | Pyr_redox_2 | FMO-like |
| 35 | PF07992 | g15927.t1 | FMO-like | Pyr_redox_2 | FMO-like |
| 36 | PF07992 | g15928.t1 | FMO-like | Pyr_redox_2 | FMO-like |
| 37 | PF00643 | g15929.t1 | PLATZ | zf-B_box | PLATZ |
| 38 | PF08541 | g15930.t1 | NA | ACP_syn_III_C | ACP_syn_III_C |
| 39 | NA | g15931.t1 | Exostosin | NA | Exostosin |
| 40 | NA | g15932.t1 | DOG1 | NA | DOG1 |
| 41 | NA | g15933.t1 | NA | NA | g15933.t1 |
| 42 | NA | g15934.t1 | NA | NA | g15934.t1 |
| 43 | NA | g15935.t1 | DUF3506 | NA | DUF3506 |
| 44 | PF08282 | g15936.t1 | Trehalose_PPase | Hydrolase_3 | Trehalose_PPase |
| 45 | PF00923 | g15937.t1 | NA | TAL_FSA | TAL_FSA |
| 46 | PF00251 | g15938.t1 | DUF3357 | Glyco_hydro_32N | DUF3357 |
| 47 | PF00394 | g15939.t1 | NA | Cu-oxidase | Cu-oxidase |
| 48 | PF00505 | g15940.t1 | YABBY | HMG_box | YABBY |
| 49 | PF00652 | g15941.t1 | DUF3474 | Ricin_B_lectin | DUF3474 |
| 50 | NA | g15942.t1 | Mem_trans | NA | Mem_trans |
| 51 | NA | g15943.t1 | MCRS_N | NA | MCRS_N |
| 52 | NA | g15944.t1 | NA | NA | g15944.t1 |
| 53 | NA | g15945.t1 | NAM | NA | NAM |
| 54 | PF00656 | g15946.t1 | Peptidase_C13 | Peptidase_C14 | Peptidase_C13 |
| 55 | PF14559 | g15947.t1 | TPR_16 | TPR_19 | TPR_16 |
| 56 | PF00009 | g15948.t1 | MMR_HSR1 | GTP_EFTU | MMR_HSR1 |
| 57 | PF04043 | g15949.t1 | NA | PMEI | PMEI |
| 58 | PF04043 | g15950.t1 | LTP_2 | PMEI | LTP_2 |
| 59 | PF04043 | g15951.t1 | LTP_2 | PMEI | LTP_2 |
| 60 | NA | g15952.t1 | DUF3537 | NA | DUF3537 |
| 61 | PF04043 | g15953.t1 | NA | PMEI | PMEI |
| 62 | NA | g15954.t1 | AOX | NA | AOX |
| 63 | PF13923 | g15955.t1 | LTP_2 | zf-C3HC4_2 | LTP_2 |
| 64 | PF14559 | g15956.t1 | TPR_16 | TPR_19 | TPR_16 |
| 65 | PF00249 | g15957.t1 | TPR_16 | Myb_DNA-binding | TPR_16 |
| 66 | PF03171 | g15958.t1 | COX17 | 2OG-FeII_Oxy | COX17 |
| 67 | PF13920 | g15959.t1 | Prok-RING_4 | zf-C3HC4_3 | Prok-RING_4 |
| 68 | PF05699 | g15960.t1 | DUF659 | Dimer_Tnp_hAT | DUF659 |
| 69 | PF00067 | g15961.t1 | TIP49 | p450 | TIP49 |
| 70 | PF00125 | g15962.t1 | Histone_H2A_C | Histone | Histone_H2A_C |
| 71 | PF00067 | g15963.t1 | FAE1_CUT1_RppA | p450 | FAE1_CUT1_RppA |
| 72 | PF03372 | g15964.t1 | NA | Exo_endo_phos | Exo_endo_phos |
| 73 | PF00447 | g16403.t1 | APG6 | HSF_DNA-bind | APG6 |
| 74 | PF01202 | g16404.t1 | Intron_maturas2 | SKI | Intron_maturas2 |
| 75 | PF01369 | g16405.t1 | DUF1981 | Sec7 | DUF1981 |
| 76 | PF13360 | g16406.t1 | NA | PQQ_2 | PQQ_2 |
| 77 | NA | g16407.t1 | Rad1 | NA | Rad1 |
| 78 | PF17177 | g16408.t1 | PPR | PPR_long | PPR |
| 79 | PF05773 | g16409.t1 | LOB | RWD | LOB |
| 80 | PF08282 | g16410.t1 | Glyco_transf_20 | Hydrolase_3 | Glyco_transf_20 |
| 81 | PF00732 | g16411.t1 | FAD_binding_2 | GMC_oxred_N | FAD_binding_2 |
| 82 | PF00005 | g16412.t1 | ABC_membrane | ABC_tran | ABC_membrane |
| 83 | PF13621 | g18698.t1 | Ribosomal_L21p | Cupin_8 | Ribosomal_L21p |
| 84 | PF17177 | g18699.t1 | PPR | PPR_long | PPR |
| 85 | NA | g18700.t1 | RPE65 | NA | RPE65 |
| 86 | PF00098 | g18701.t1 | RPE65 | zf-CCHC | RPE65 |
| 87 | PF00628 | g18702.t1 | ELM2 | PHD | ELM2 |
| 88 | PF00628 | g18703.t1 | Homeobox_KN | PHD | Homeobox_KN |
| 89 | NA | g18704.t1 | NA | NA | g18704.t1 |
| 90 | PF07651 | g18705.t1 | EamA | ANTH | EamA |
| 91 | NA | g18706.t1 | Ribosomal_L4 | NA | Ribosomal_L4 |
| 92 | NA | g18707.t1 | NA | NA | g18707.t1 |
| 93 | PF02201 | g18708.t1 | ABC2_membrane | SWIB | ABC2_membrane |
| 94 | PF12776 | g18709.t1 | DUF573 | Myb_DNA-bind_3 | DUF573 |
| 95 | NA | g18710.t1 | Transglut_core2 | NA | Transglut_core2 |
| 96 | NA | g18711.t1 | DAGK_cat | NA | DAGK_cat |
| 97 | PF12937 | g18712.t1 | Herpes_UL92 | F-box-like | Herpes_UL92 |
| 98 | PF00005 | g18713.t1 | Fer4_9 | ABC_tran | Fer4_9 |
| 99 | PF14559 | g18714.t1 | PPR | TPR_19 | PPR |
| 100 | NA | g18715.t1 | DUF716 | NA | DUF716 |
| 101 | PF00076 | g18716.t1 | Nucleotid_trans | RRM_1 | Nucleotid_trans |
| 102 | PF13961 | g18717.t1 | Retrotran_gag_3 | DUF4219 | Retrotran_gag_3 |
| 103 | PF00069 | g18718.t1 | K_trans | Pkinase | K_trans |
| 104 | PF00067 | g18719.t1 | NA | p450 | p450 |
| 105 | NA | g18720.t1 | Nnf1 | NA | Nnf1 |
| 106 | PF08502 | g18721.t1 | Adaptin_N | LeuA_dimer | Adaptin_N |
| 107 | NA | g18722.t1 | NA | NA | g18722.t1 |
| 108 | PF12701 | g18723.t1 | zf-ANAPC11 | LSM14 | zf-ANAPC11 |
| 109 | NA | g18724.t1 | NA | NA | g18724.t1 |
| 110 | NA | g18725.t1 | DUF1218 | NA | DUF1218 |
| 111 | PF00732 | g18726.t1 | FAD_binding_2 | GMC_oxred_N | FAD_binding_2 |
| 112 | PF00314 | g18727.t1 | UPF0114 | Thaumatin | UPF0114 |
| 113 | PF00314 | g18728.t1 | NA | Thaumatin | Thaumatin |
| 114 | PF17177 | g18729.t1 | PPR | PPR_long | PPR |
| 115 | PF00175 | g18730.t1 | NA | NAD_binding_1 | NAD_binding_1 |
| 116 | PF00226 | g18731.t1 | NA | DnaJ | DnaJ |
| 117 | NA | g18732.t1 | NA | NA | g18732.t1 |
| 118 | PF00327 | g18733.t1 | NA | Ribosomal_L30 | Ribosomal_L30 |
| 119 | PF13191 | g18734.t1 | PTR2 | AAA_16 | PTR2 |
| 120 | PF01180 | g18735.t1 | bZIP_1 | DHO_dh | bZIP_1 |
| 121 | NA | g18736.t1 | K_trans | NA | K_trans |
| 122 | PF01424 | g18737.t1 | zf-NF-X1 | R3H | zf-NF-X1 |
| 123 | PF07779 | g18738.t1 | NA | Cas1_AcylT | Cas1_AcylT |
| 124 | PF00249 | g18739.t1 | Spatacsin_C | Myb_DNA-binding | Spatacsin_C |
| 125 | NA | g18740.t1 | NA | NA | g18740.t1 |
| 126 | PF05191 | g18741.t1 | K_trans | ADK_lid | K_trans |
| 127 | PF02910 | g18742.t1 | PI3Ka | Succ_DH_flav_C | PI3Ka |
| 128 | NA | g18743.t1 | Transferase | NA | Transferase |
| 129 | NA | g18744.t1 | NIF | NA | NIF |
| 130 | PF00106 | g20786.t1 | NA | adh_short | adh_short |
| 131 | PF06424 | g20787.t1 | TPR_16 | PRP1_N | TPR_16 |
| 132 | NA | g20788.t1 | Sugar_tr | NA | Sugar_tr |
| 133 | PF00561 | g20789.t1 | Hydrolase_4 | Abhydrolase_1 | Hydrolase_4 |
| 134 | PF00561 | g20790.t1 | Hydrolase_4 | Abhydrolase_1 | Hydrolase_4 |
| 135 | PF00503 | g20791.t1 | Hydrolase_4 | G-alpha | Hydrolase_4 |
| 136 | NA | g20792.t1 | LANC_like | NA | LANC_like |
| 137 | PF01357 | g20793.t1 | NA | Pollen_allerg_1 | Pollen_allerg_1 |
| 138 | PF01399 | g20794.t1 | Tetraspannin | PCI | Tetraspannin |
| 139 | PF13508 | g20795.t1 | Acetyltransf_1 | Acetyltransf_7 | Acetyltransf_1 |
| 140 | NA | g20796.t1 | APG6 | NA | APG6 |
| 141 | PF13962 | g20797.t1 | Vma12 | PGG | Vma12 |
| 142 | NA | g20798.t1 | NA | NA | g20798.t1 |
| 143 | PF00173 | g20799.t1 | NA | Cyt-b5 | Cyt-b5 |
| 144 | PF01556 | g20800.t1 | SCAMP | DnaJ_C | SCAMP |
| 145 | PF13947 | g20801.t1 | NA | GUB_WAK_bind | GUB_WAK_bind |
| 146 | PF00561 | g20802.t1 | Hydrolase_4 | Abhydrolase_1 | Hydrolase_4 |
| 147 | PF00249 | g21159.t1 | bHLH-MYC_N | Myb_DNA-binding | bHLH-MYC_N |
| 148 | PF13180 | g21160.t1 | ALMT | PDZ_2 | ALMT |
| 149 | PF04438 | g21161.t1 | SHQ1 | zf-HIT | SHQ1 |
| 150 | PF00789 | g21162.t1 | CDC45 | UBX | CDC45 |
| 151 | PF00319 | g21163.t1 | K-box | SRF-TF | K-box |
| 152 | PF00069 | g21164.t1 | APH | Pkinase | APH |
| 153 | PF03226 | g21165.t1 | RGP | Yippee-Mis18 | RGP |
| 154 | PF00067 | g21166.t1 | OPT | p450 | OPT |
| 155 | PF01661 | g21167.t1 | MT-A70 | Macro | MT-A70 |
| 156 | PF02298 | g21168.t1 | PI-PLC-X | Cu_bind_like | PI-PLC-X |
| 157 | NA | g21169.t1 | MFS_5 | NA | MFS_5 |
| 158 | PF04434 | g21170.t1 | HpcH_HpaI | SWIM | HpcH_HpaI |
| 159 | NA | g21171.t1 | UPF0160 | NA | UPF0160 |
| 160 | PF09090 | g21172.t1 | VIT1 | MIF4G_like_2 | VIT1 |
| 161 | PF16845 | g21173.t1 | DUF659 | SQAPI | DUF659 |
| 162 | NA | g21174.t1 | DUF4666 | NA | DUF4666 |
| 163 | PF00069 | g21175.t1 | Haspin_kinase | Pkinase | Haspin_kinase |
| 164 | PF00010 | g21176.t1 | NA | HLH | HLH |
| 165 | PF06220 | g21177.t1 | OPT | zf-U1 | OPT |
| 166 | NA | g21178.t1 | OPT | NA | OPT |
| 167 | PF00450 | g21179.t1 | OPT | Peptidase_S10 | OPT |
| 168 | PF00450 | g21180.t1 | TOM20_plant | Peptidase_S10 | TOM20_plant |
| 169 | PF00076 | g21181.t1 | RabGAP-TBC | RRM_1 | RabGAP-TBC |
| 170 | PF00685 | g21182.t1 | DUF538 | Sulfotransfer_1 | DUF538 |
| 171 | PF00561 | g21183.t1 | Hydrolase_4 | Abhydrolase_1 | Hydrolase_4 |
| 172 | PF01053 | g21184.t1 | MFS_MOT1 | Cys_Met_Meta_PP | MFS_MOT1 |
| 173 | PF00067 | g21185.t1 | NA | p450 | p450 |
| 174 | PF13638 | g21186.t1 | Med10 | PIN_4 | Med10 |
| 175 | PF00046 | g21187.t1 | HALZ | Homeobox | HALZ |
| 176 | NA | g21188.t1 | NA | NA | g21188.t1 |
| 177 | PF09118 | g21189.t1 | Glyoxal_oxid_N | DUF1929 | Glyoxal_oxid_N |
| 178 | PF00010 | g21190.t1 | bHLH-MYC_N | HLH | bHLH-MYC_N |
| 179 | PF00561 | g21191.t1 | Hydrolase_4 | Abhydrolase_1 | Hydrolase_4 |
| 180 | NA | g21192.t1 | Lipase_GDSL | NA | Lipase_GDSL |
| 181 | NA | g21193.t1 | DUF4033 | NA | DUF4033 |
| 182 | PF00759 | g21194.t1 | NA | Glyco_hydro_9 | Glyco_hydro_9 |
| 183 | PF14559 | g21195.t1 | Lung_7-TM_R | TPR_19 | Lung_7-TM_R |
| 184 | PF08144 | g21196.t1 | DUF641 | CPL | DUF641 |
| 185 | PF08879 | g21197.t1 | Cpn60_TCP1 | WRC | Cpn60_TCP1 |
| 186 | PF03106 | g21198.t1 | DUF4005 | WRKY | DUF4005 |
| 187 | PF00462 | g21199.t1 | NA | Glutaredoxin | Glutaredoxin |
| 188 | PF00082 | g21200.t1 | NAM | Peptidase_S8 | NAM |
| 189 | PF04146 | g21201.t1 | DUF4228 | YTH | DUF4228 |
| 190 | PF02779 | g21202.t1 | DUF4228 | Transket_pyr | DUF4228 |
| 191 | PF08240 | g21203.t1 | ADH_zinc_N | ADH_N | ADH_zinc_N |
| 192 | PF00076 | g21204.t1 | eIF3g | RRM_1 | eIF3g |
| 193 | NA | g21205.t1 | PTR2 | NA | PTR2 |
| 194 | PF00628 | g21206.t1 | PHD_2 | PHD | PHD_2 |
| 195 | PF06337 | g21207.t1 | UCH | DUSP | UCH |
| 196 | PF00332 | g21208.t1 | PTR2 | Glyco_hydro_17 | PTR2 |
| 197 | NA | g21209.t1 | Ribosomal_60s | NA | Ribosomal_60s |
| 198 | PF08323 | g21210.t1 | Glycos_transf_1 | Glyco_transf_5 | Glycos_transf_1 |
| 199 | NA | g21211.t1 | MIF4G | NA | MIF4G |
| 200 | NA | g21212.t1 | Ctr | NA | Ctr |
| 201 | NA | g21213.t1 | NA | NA | g21213.t1 |
| 202 | PF13460 | g21214.t1 | E1_dh | NAD_binding_10 | E1_dh |
| 203 | PF01852 | g21215.t1 | NA | START | START |
| 204 | PF00076 | g21216.t1 | Galactosyl_T | RRM_1 | Galactosyl_T |
| 205 | PF13857 | g21217.t1 | PH_2 | Ank_5 | PH_2 |
| 206 | PF00082 | g21218.t1 | PA | Peptidase_S8 | PA |
| 207 | PF05922 | g21219.t1 | Reticulon | Inhibitor_I9 | Reticulon |
| 208 | PF00082 | g21220.t1 | PA | Peptidase_S8 | PA |
| 209 | PF00082 | g21221.t1 | NA | Peptidase_S8 | Peptidase_S8 |
| 210 | PF13639 | g21222.t1 | zf-ANAPC11 | zf-RING_2 | zf-ANAPC11 |
| 211 | PF03946 | g21223.t1 | Cation_efflux | Ribosomal_L11_N | Cation_efflux |
| 212 | PF08100 | g21224.t1 | Prok-RING_4 | Dimerisation | Prok-RING_4 |
| 213 | PF08100 | g21225.t1 | Methyltransf_2 | Dimerisation | Methyltransf_2 |
| 214 | NA | g21226.t1 | Methyltransf_2 | NA | Methyltransf_2 |
| 215 | PF08100 | g21227.t1 | E1_dh | Dimerisation | E1_dh |
| 216 | PF03171 | g21228.t1 | DIOX_N | 2OG-FeII_Oxy | DIOX_N |
| 217 | PF03171 | g21229.t1 | DIOX_N | 2OG-FeII_Oxy | DIOX_N |
| 218 | PF00719 | g21230.t1 | JmjN | Pyrophosphatase | JmjN |
| 219 | PF00646 | g21231.t1 | Acetyltransf_1 | F-box | Acetyltransf_1 |
| 220 | PF02953 | g21232.t1 | NA | zf-Tim10_DDP | zf-Tim10_DDP |
| 221 | PF00076 | g21233.t1 | B3 | RRM_1 | B3 |
| 222 | PF00403 | g21234.t1 | NA | HMA | HMA |
| 223 | NA | g21235.t1 | NA | NA | g21235.t1 |
| 224 | NA | g21236.t1 | NAM | NA | NAM |
| 225 | PF12906 | g21237.t1 | NA | RINGv | RINGv |
| 226 | PF12755 | g21238.t1 | DUF4042 | Vac14_Fab1_bd | DUF4042 |
| 227 | PF17123 | g21239.t1 | zf-ANAPC11 | zf-RING_11 | zf-ANAPC11 |
| 228 | PF08240 | g21240.t1 | ADH_zinc_N | ADH_N | ADH_zinc_N |
| 229 | NA | g21241.t1 | EamA | NA | EamA |
| 230 | PF03101 | g21242.t1 | Nucleoporin2 | FAR1 | Nucleoporin2 |
| 231 | NA | g21243.t1 | TPX2 | NA | TPX2 |
| 232 | NA | g21244.t1 | NA | NA | g21244.t1 |
| 233 | NA | g21245.t1 | EamA | NA | EamA |
| 234 | NA | g21246.t1 | NA | NA | g21246.t1 |
| 235 | PF13649 | g21247.t1 | Methyltransf_29 | Methyltransf_25 | Methyltransf_29 |
| 236 | NA | g21248.t1 | TMPIT | NA | TMPIT |
| 237 | PF13837 | g21249.t1 | Haspin_kinase | Myb_DNA-bind_4 | Haspin_kinase |
| 238 | PF00327 | g21250.t1 | NA | Ribosomal_L30 | Ribosomal_L30 |
| 239 | NA | g21251.t1 | DUF604 | NA | DUF604 |
| 240 | PF00069 | g21252.t1 | APH | Pkinase | APH |
| 241 | PF14569 | g21253.t1 | Tmemb_14 | zf-UDP | Tmemb_14 |
| 242 | NA | g21254.t1 | NA | NA | g21254.t1 |
| 243 | PF11940 | g21255.t1 | DUF3458_C | DUF3458 | DUF3458_C |
| 244 | PF11940 | g21256.t1 | DUF3458_C | DUF3458 | DUF3458_C |
| 245 | NA | g21257.t1 | BCAS3 | NA | BCAS3 |
| 246 | PF02214 | g21258.t1 | Hpt | BTB_2 | Hpt |
| 247 | PF01156 | g21259.t1 | CitMHS | IU_nuc_hydro | CitMHS |
| 248 | PF00067 | g21260.t1 | NA | p450 | p450 |
| 249 | PF00690 | g21261.t1 | E1-E2_ATPase | Cation_ATPase_N | E1-E2_ATPase |
| 250 | PF07859 | g21262.t1 | Chlorophyllase2 | Abhydrolase_3 | Chlorophyllase2 |
| 251 | PF00069 | g21263.t1 | NA | Pkinase | Pkinase |
| 252 | PF00106 | g21264.t1 | Epimerase | adh_short | Epimerase |
| 253 | NA | g21265.t1 | MIP | NA | MIP |
| 254 | NA | g21266.t1 | Peptidase_A22B | NA | Peptidase_A22B |
| 255 | NA | g21267.t1 | NA | NA | g21267.t1 |
| 256 | PF08245 | g21268.t1 | NA | Mur_ligase_M | Mur_ligase_M |
| 257 | PF13238 | g21269.t1 | DUF3741 | AAA_18 | DUF3741 |
| 258 | PF00958 | g21270.t1 | tRNA_Me_trans | GMP_synt_C | tRNA_Me_trans |
| 259 | PF03171 | g21271.t1 | DIOX_N | 2OG-FeII_Oxy | DIOX_N |
| 260 | PF00010 | g21272.t1 | bHLH-MYC_N | HLH | bHLH-MYC_N |
| 261 | PF00005 | g21273.t1 | tRNA-synt_1b | ABC_tran | tRNA-synt_1b |
| 262 | NA | g21274.t1 | NA | NA | g21274.t1 |
| 263 | PF00225 | g21275.t1 | NA | Kinesin | Kinesin |
| 264 | NA | g21276.t1 | Mito_carr | NA | Mito_carr |
| 265 | PF01167 | g21277.t1 | DUF3527 | Tub | DUF3527 |
| 266 | PF07500 | g21278.t1 | LRRNT_2 | TFIIS_M | LRRNT_2 |
| 267 | NA | g21279.t1 | NA | NA | g21279.t1 |
| 268 | PF00702 | g21280.t1 | HAD_2 | Hydrolase | HAD_2 |
| 269 | PF13806 | g21281.t1 | GCS2 | Rieske_2 | GCS2 |
| 270 | PF00091 | g21282.t1 | NA | Tubulin | Tubulin |
| 271 | PF07714 | g21283.t1 | TPR_16 | Pkinase_Tyr | TPR_16 |
| 272 | PF00168 | g21284.t1 | NA | C2 | C2 |
| 273 | PF02373 | g21285.t1 | PLU-1 | JmjC | PLU-1 |
| 274 | NA | g21286.t1 | NA | NA | g21286.t1 |
| 275 | PF13476 | g21287.t1 | PEARLI-4 | AAA_23 | PEARLI-4 |
| 276 | PF03479 | g21288.t1 | DUF4228 | DUF296 | DUF4228 |
| 277 | PF00578 | g21289.t1 | GSHPx | AhpC-TSA | GSHPx |
| 278 | PF00578 | g21290.t1 | GSHPx | AhpC-TSA | GSHPx |
| 279 | PF00578 | g21291.t1 | GSHPx | AhpC-TSA | GSHPx |
| 280 | PF07731 | g21292.t1 | Exostosin | Cu-oxidase_2 | Exostosin |
| 281 | PF00168 | g21293.t1 | PRT_C | C2 | PRT_C |
| 282 | NA | g21294.t1 | GASA | NA | GASA |
| 283 | PF02798 | g21295.t1 | BAG | GST_N | BAG |
| 284 | PF00702 | g21296.t1 | E1-E2_ATPase | Hydrolase | E1-E2_ATPase |
| 285 | PF00702 | g21297.t1 | E1-E2_ATPase | Hydrolase | E1-E2_ATPase |
| 286 | PF00702 | g21298.t1 | E1-E2_ATPase | Hydrolase | E1-E2_ATPase |
| 287 | PF13193 | g21299.t1 | AMP-binding | AMP-binding_C | AMP-binding |
| 288 | NA | g21300.t1 | PALP | NA | PALP |
| 289 | PF00091 | g21301.t1 | NA | Tubulin | Tubulin |
| 290 | PF07714 | g21302.t1 | zf-ANAPC11 | Pkinase_Tyr | zf-ANAPC11 |
| 291 | PF08772 | g21303.t1 | PIN_6 | NOB1_Zn_bind | PIN_6 |
| 292 | PF07714 | g21304.t1 | LRRNT_2 | Pkinase_Tyr | LRRNT_2 |
| 293 | PF03168 | g21305.t1 | Glyco_transf_90 | LEA_2 | Glyco_transf_90 |
| 294 | PF04525 | g21306.t1 | CBFB_NFYA | LOR | CBFB_NFYA |
| 295 | PF04525 | g21307.t1 | NA | LOR | LOR |
| 296 | PF04525 | g21308.t1 | NA | LOR | LOR |
| 297 | PF03810 | g21309.t1 | NT-C2 | IBN_N | NT-C2 |
| 298 | PF00808 | g21310.t1 | NA | CBFD_NFYB_HMF | CBFD_NFYB_HMF |
| 299 | NA | g21311.t1 | NA | NA | g21311.t1 |
| 300 | NA | g21312.t1 | Remorin_N | NA | Remorin_N |
| 301 | PF00656 | g21313.t1 | NA | Peptidase_C14 | Peptidase_C14 |
| 302 | PF00270 | g21314.t1 | Helicase_C | DEAD | Helicase_C |
| 303 | PF07714 | g21315.t1 | APH | Pkinase_Tyr | APH |
| 304 | PF00168 | g21316.t1 | Neprosin | C2 | Neprosin |
| 305 | PF01426 | g21317.t1 | Herpes_UL52 | BAH | Herpes_UL52 |
| 306 | PF01214 | g21318.t1 | Helicase_C | CK_II_beta | Helicase_C |
| 307 | PF13883 | g21319.t1 | DUF2470 | Pyrid_oxidase_2 | DUF2470 |
| 308 | PF17177 | g21320.t1 | Methyltransf_29 | PPR_long | Methyltransf_29 |
| 309 | PF07876 | g21321.t1 | Sec63 | Dabb | Sec63 |
| 310 | PF00270 | g21322.t1 | Helicase_C | DEAD | Helicase_C |
| 311 | PF03106 | g21323.t1 | PPI_Ypi1 | WRKY | PPI_Ypi1 |
| 312 | PF03106 | g21324.t1 | Tetraspannin | WRKY | Tetraspannin |
| 313 | PF00575 | g21325.t1 | Ribosomal_L21p | S1 | Ribosomal_L21p |
| 314 | PF00412 | g21326.t1 | zf-ANAPC11 | LIM | zf-ANAPC11 |
| 315 | PF00010 | g21327.t1 | SAGA-Tad1 | HLH | SAGA-Tad1 |
| 316 | PF13639 | g21328.t1 | zf-ANAPC11 | zf-RING_2 | zf-ANAPC11 |
| 317 | PF13949 | g21329.t1 | GRAS | ALIX_LYPXL_bnd | GRAS |
| 318 | PF00067 | g21330.t1 | BRAP2 | p450 | BRAP2 |
| 319 | PF02298 | g21331.t1 | GRAS | Cu_bind_like | GRAS |
| 320 | PF01429 | g21332.t1 | NA | MBD | MBD |
| 321 | NA | g21333.t1 | NA | NA | g21333.t1 |
| 322 | PF08767 | g21334.t1 | NA | CRM1_C | CRM1_C |
| 323 | PF00954 | g21335.t1 | H_PPase | S_locus_glycop | H_PPase |
| 324 | NA | g21336.t1 | Neprosin_AP | NA | Neprosin_AP |
| 325 | PF00954 | g21337.t1 | NA | S_locus_glycop | S_locus_glycop |
| 326 | PF00954 | g21338.t1 | NA | S_locus_glycop | S_locus_glycop |
| 327 | PF00199 | g21339.t1 | Catalase-rel | Catalase | Catalase-rel |
| 328 | PF00789 | g21340.t1 | NA | UBX | UBX |
| 329 | PF14555 | g21341.t1 | Asp | UBA_4 | Asp |
| 330 | PF01476 | g21342.t1 | PIG-U | LysM | PIG-U |
| 331 | PF00004 | g21343.t1 | Rad17 | AAA | Rad17 |
| 332 | PF00582 | g21344.t1 | NA | Usp | Usp |
| 333 | NA | g21345.t1 | NOT2_3_5 | NA | NOT2_3_5 |
| 334 | NA | g21346.t1 | NA | NA | g21346.t1 |
| 335 | PF07714 | g21347.t1 | APH | Pkinase_Tyr | APH |
| 336 | NA | g21348.t1 | COG2 | NA | COG2 |
| 337 | NA | g21349.t1 | DUF4005 | NA | DUF4005 |
| 338 | NA | g21350.t1 | Rhomboid | NA | Rhomboid |
| 339 | PF08542 | g21351.t1 | Rad17 | Rep_fac_C | Rad17 |
| 340 | NA | g21352.t1 | NA | NA | g21352.t1 |
| 341 | PF00013 | g21353.t1 | Amidase | KH_1 | Amidase |
| 342 | PF00300 | g21354.t1 | NA | His_Phos_1 | His_Phos_1 |
| 343 | NA | g21355.t1 | DUF1997 | NA | DUF1997 |
| 344 | PF01112 | g21356.t1 | NA | Asparaginase_2 | Asparaginase_2 |
| 345 | NA | g21357.t1 | DUF3444 | NA | DUF3444 |
|  |  |  |  |  |  |
| ***V. vinifera*** | |  |  |  |  |
|  | **PFAM** | **SeqID** | **PFAM_NAME** | **DOM_NAME** | **BRIEF NAME** |
| 1 | NA | Vitvi02g00295.t01 | MIP | NA | MIP |
| 2 | PF01433 | Vitvi02g00296.t01 | DUF3458_C | Peptidase_M1 | DUF3458_C |
| 3 | NA | Vitvi02g00297.t01 | NA | NA | Vitvi02g00297 |
| 4 | PF02214 | Vitvi02g00298.t01 | NA | BTB_2 | BTB_2 |
| 5 | NA | Vitvi02g00301.t01 | CitMHS | NA | CitMHS |
| 6 | NA | Vitvi02g00302.t01 | NA | NA | Vitvi02g00302 |
| 7 | PF00067 | Vitvi02g00303.t01 | NA | p450 | p450 |
| 8 | PF00690 | Vitvi02g00304.t01 | E1-E2_ATPase | Cation_ATPase_N | E1-E2_ATPase |
| 9 | PF12906 | Vitvi02g00305.t01 | NA | RINGv | RINGv |
| 10 | NA | Vitvi02g00306.t01 | NA | NA | Vitvi02g00306 |
| 11 | NA | Vitvi02g00307.t01 | NA | NA | Vitvi02g00307 |
| 12 | PF00069 | Vitvi02g00308.t01 | NA | Pkinase | Pkinase |
| 13 | PF00106 | Vitvi02g00309.t01 | Epimerase | adh_short | Epimerase |
| 14 | NA | Vitvi02g00310.t01 | MIP | NA | MIP |
| 15 | NA | Vitvi02g00311.t01 | Peptidase_A22B | NA | Peptidase_A22B |
| 16 | NA | Vitvi02g00312.t01 | NA | NA | Vitvi02g00312 |
| 17 | PF08245 | Vitvi02g00313.t01 | NA | Mur_ligase_M | Mur_ligase_M |
| 18 | NA | Vitvi02g00314.t01 | DUF3741 | NA | DUF3741 |
| 19 | PF00958 | Vitvi02g00315.t01 | tRNA_Me_trans | GMP_synt_C | tRNA_Me_trans |
| 20 | PF03171 | Vitvi02g00316.t01 | DIOX_N | 2OG-FeII_Oxy | DIOX_N |
| 21 | PF00010 | Vitvi02g00317.t01 | bHLH-MYC_N | HLH | bHLH-MYC_N |
| 22 | NA | Vitvi02g00318.t01 | tRNA-synt_1b | NA | tRNA-synt_1b |
| 23 | PF00225 | Vitvi02g00319.t01 | NA | Kinesin | Kinesin |
| 24 | NA | Vitvi02g00320.t01 | Mito_carr | NA | Mito_carr |
| 25 | PF07500 | Vitvi02g00322.t01 | DUF3527 | TFIIS_M | DUF3527 |
| 26 | PF00702 | Vitvi02g00324.t01 | HAD_2 | Hydrolase | HAD_2 |
| 27 | PF00562 | Vitvi02g00325.t01 | NA | RNA_pol_Rpb2_6 | RNA_pol_Rpb2_6 |
| 28 | NA | Vitvi02g00326.t01 | GCS2 | NA | GCS2 |
| 29 | PF00091 | Vitvi02g00327.t01 | NA | Tubulin | Tubulin |
| 30 | PF07714 | Vitvi02g00328.t01 | TPR_16 | Pkinase_Tyr | TPR_16 |
| 31 | PF02373 | Vitvi02g00329.t01 | PLU-1 | JmjC | PLU-1 |
| 32 | NA | Vitvi02g00330.t01 | RuvX | NA | RuvX |
| 33 | PF03479 | Vitvi02g00331.t01 | NA | DUF296 | DUF296 |
| 34 | PF00578 | Vitvi02g00332.t01 | GSHPx | AhpC-TSA | GSHPx |
| 35 | PF00578 | Vitvi02g00333.t01 | GSHPx | AhpC-TSA | GSHPx |
| 36 | NA | Vitvi02g00334.t01 | Exostosin | NA | Exostosin |
| 37 | PF02798 | Vitvi02g00335.t01 | NA | GST_N | GST_N |
| 38 | PF00702 | Vitvi02g00337.t01 | E1-E2_ATPase | Hydrolase | E1-E2_ATPase |
| 39 | PF13193 | Vitvi02g00338.t01 | AMP-binding | AMP-binding_C | AMP-binding |
| 40 | NA | Vitvi02g00339.t01 | PALP | NA | PALP |
| 41 | PF00091 | Vitvi02g00340.t01 | NA | Tubulin | Tubulin |
| 42 | PF08772 | Vitvi02g00341.t01 | PIN_6 | NOB1_Zn_bind | PIN_6 |
| 43 | PF07714 | Vitvi02g00342.t01 | LRRNT_2 | Pkinase_Tyr | LRRNT_2 |
| 44 | NA | Vitvi02g00343.t01 | NA | NA | Vitvi02g00343 |
| 45 | PF04525 | Vitvi02g00344.t01 | NA | LOR | LOR |
| 46 | PF04525 | Vitvi02g00345.t01 | NA | LOR | LOR |
| 47 | PF02403 | Vitvi02g00346.t01 | NT-C2 | Seryl_tRNA_N | NT-C2 |
| 48 | PF00808 | Vitvi02g00347.t01 | NA | CBFD_NFYB_HMF | CBFD_NFYB_HMF |
| 49 | NA | Vitvi02g00348.t01 | Remorin_C | NA | Remorin_C |
| 50 | PF00270 | Vitvi02g00349.t01 | Helicase_C | DEAD | Helicase_C |
| 51 | NA | Vitvi02g00350.t01 | K_trans | NA | K_trans |
| 52 | NA | Vitvi02g00351.t01 | NA | NA | Vitvi02g00351 |
| 53 | NA | Vitvi02g00352.t01 | NA | NA | Vitvi02g00352 |
| 54 | NA | Vitvi02g00353.t01 | NA | NA | Vitvi02g00353 |
| 55 | PF00069 | Vitvi02g00354.t01 | APH | Pkinase | APH |
| 56 | NA | Vitvi02g00356.t01 | NA | NA | Vitvi02g00356 |
| 57 | NA | Vitvi02g00357.t01 | NA | NA | Vitvi02g00357 |
| 58 | NA | Vitvi02g00358.t01 | NA | NA | Vitvi02g00358 |
| 59 | PF17407 | Vitvi02g00359.t01 | NA | Nrap_D6 | Nrap_D6 |
| 60 | NA | Vitvi02g00360.t01 | NA | NA | Vitvi02g00360 |
| 61 | NA | Vitvi02g00361.t01 | NA | NA | Vitvi02g00361 |
| 62 | NA | Vitvi02g00362.t01 | NA | NA | Vitvi02g00362 |
| 63 | PF02186 | Vitvi02g00363.t01 | NA | TFIIE_beta | TFIIE_beta |
| 64 | PF07876 | Vitvi02g00364.t01 | NA | Dabb | Dabb |
| 65 | PF00270 | Vitvi02g00365.t01 | Helicase_C | DEAD | Helicase_C |
| 66 | NA | Vitvi02g00366.t01 | Tetraspannin | NA | Tetraspannin |
| 67 | PF00575 | Vitvi02g00367.t01 | Ribosomal_L21p | S1 | Ribosomal_L21p |
| 68 | PF00412 | Vitvi02g00368.t01 | NA | LIM | LIM |
| 69 | NA | Vitvi02g00371.t01 | NA | NA | Vitvi02g00371 |
| 70 | NA | Vitvi02g00372.t01 | NA | NA | Vitvi02g00372 |
| 71 | PF00789 | Vitvi02g00373.t01 | NA | UBX | UBX |
| 72 | PF14555 | Vitvi02g00374.t01 | NA | UBA_4 | UBA_4 |
| 73 | NA | Vitvi02g00375.t01 | PIG-U | NA | PIG-U |
| 74 | PF00067 | Vitvi02g00377.t01 | NA | p450 | p450 |
| 75 | NA | Vitvi02g00378.t01 | NA | NA | Vitvi02g00378 |
| 76 | NA | Vitvi02g00379.t01 | NOT2_3_5 | NA | NOT2_3_5 |
| 77 | PF07714 | Vitvi02g00380.t01 | APH | Pkinase_Tyr | APH |
| 78 | NA | Vitvi02g00381.t01 | DUF4005 | NA | DUF4005 |
| 79 | NA | Vitvi02g00382.t01 | Rhomboid | NA | Rhomboid |
| 80 | PF08542 | Vitvi02g00383.t01 | Rad17 | Rep_fac_C | Rad17 |
| 81 | PF13639 | Vitvi02g00384.t01 | zf-ANAPC11 | zf-RING_2 | zf-ANAPC11 |
| 82 | NA | Vitvi02g00385.t01 | DUF810 | NA | DUF810 |
| 83 | PF00168 | Vitvi02g00386.t01 | NA | C2 | C2 |
| 84 | PF00447 | Vitvi02g00387.t01 | NA | HSF_DNA-bind | HSF_DNA-bind |
| 85 | NA | Vitvi02g00388.t01 | IPP-2 | NA | IPP-2 |
| 86 | PF00270 | Vitvi02g00389.t01 | Helicase_C | DEAD | Helicase_C |
| 87 | PF16363 | Vitvi02g00390.t01 | Epimerase | GDP_Man_Dehyd | Epimerase |
| 88 | PF00314 | Vitvi02g00391.t01 | NA | Thaumatin | Thaumatin |
| 89 | PF13976 | Vitvi02g00392.t01 | NA | gag_pre-integrs | gag_pre-integrs |
| 90 | PF00314 | Vitvi02g00393.t01 | NA | Thaumatin | Thaumatin |
| 91 | PF00168 | Vitvi02g00394.t01 | NA | C2 | C2 |
| 92 | PF00067 | Vitvi02g00395.t01 | NA | p450 | p450 |
| 93 | PF00067 | Vitvi02g00396.t01 | NA | p450 | p450 |
| 94 | PF00067 | Vitvi02g00397.t01 | NA | p450 | p450 |
| 95 | PF00646 | Vitvi02g00398.t01 | PP2 | F-box | PP2 |
| 96 | NA | Vitvi02g00399.t01 | LIM_bind | NA | LIM_bind |
| 97 | NA | Vitvi02g00401.t01 | RRP36 | NA | RRP36 |
| 98 | NA | Vitvi02g00402.t01 | MBOAT | NA | MBOAT |
| 99 | PF01661 | Vitvi02g00403.t01 | MatE | Macro | MatE |
| 100 | NA | Vitvi02g00404.t01 | Sugar_tr | NA | Sugar_tr |
| 101 | PF00847 | Vitvi02g00406.t01 | NA | AP2 | AP2 |
| 102 | PF00847 | Vitvi02g00407.t01 | NA | AP2 | AP2 |
| 103 | NA | Vitvi02g00408.t01 | NA | NA | Vitvi02g00408 |
| 104 | NA | Vitvi02g00409.t01 | DUF4378 | NA | DUF4378 |
| 105 | NA | Vitvi02g00410.t01 | UAA | NA | UAA |
| 106 | NA | Vitvi02g00411.t01 | Ran_BP1 | NA | Ran_BP1 |
| 107 | PF00106 | Vitvi02g00412.t01 | Synaptobrevin | adh_short | Synaptobrevin |
| 108 | PF00069 | Vitvi02g00413.t01 | APH | Pkinase | APH |
| 109 | PF00623 | Vitvi02g00414.t01 | DUF3223 | RNA_pol_Rpb1_2 | DUF3223 |
| 110 | PF03105 | Vitvi02g00415.t01 | MFS_1 | SPX | MFS_1 |
| 111 | PF05199 | Vitvi02g00416.t01 | Lycopene_cycl | GMC_oxred_C | Lycopene_cycl |
| 112 | PF01179 | Vitvi02g00417.t01 | NA | Cu_amine_oxid | Cu_amine_oxid |
| 113 | NA | Vitvi02g00418.t01 | NA | NA | Vitvi02g00418 |
| 114 | NA | Vitvi02g00419.t01 | NA | NA | Vitvi02g00419 |
| 115 | PF08540 | Vitvi02g00420.t01 | NA | HMG_CoA_synt_C | HMG_CoA_synt_C |
| 116 | NA | Vitvi02g00421.t01 | TCP | NA | TCP |
| 117 | PF00082 | Vitvi02g00422.t01 | PA | Peptidase_S8 | PA |
| 118 | PF04321 | Vitvi02g00423.t01 | Epimerase | RmlD_sub_bind | Epimerase |
| 119 | PF00168 | Vitvi02g00424.t01 | PLD_C | C2 | PLD_C |
| 120 | PF00046 | Vitvi02g00425.t01 | KNOX2 | Homeobox | KNOX2 |
| 121 | NA | Vitvi02g00426.t01 | MINDY_DUB | NA | MINDY_DUB |
| 122 | NA | Vitvi02g00428.t01 | NA | NA | Vitvi02g00428 |
| 123 | PF12638 | Vitvi02g00429.t01 | NA | Staygreen | Staygreen |
| 124 | PF12638 | Vitvi02g00430.t01 | NA | Staygreen | Staygreen |
| 125 | NA | Vitvi02g00431.t01 | eIF2A | NA | eIF2A |
| 126 | NA | Vitvi02g00432.t01 | DUF1191 | NA | DUF1191 |
| 127 | PF03330 | Vitvi02g00433.t01 | NA | DPBB_1 | DPBB_1 |
| 128 | PF01096 | Vitvi02g00434.t01 | NA | TFIIS_C | TFIIS_C |
| 129 | PF03171 | Vitvi02g00435.t01 | DIOX_N | 2OG-FeII_Oxy | DIOX_N |
| 130 | PF02826 | Vitvi02g00436.t01 | F420_oxidored | 2-Hacid_dh_C | F420_oxidored |
| 131 | PF02826 | Vitvi02g00437.t01 | F420_oxidored | 2-Hacid_dh_C | F420_oxidored |
| 132 | PF00010 | Vitvi02g00439.t01 | NA | HLH | HLH |
| 133 | PF04146 | Vitvi02g00440.t01 | NA | YTH | YTH |
| 134 | NA | Vitvi02g00441.t01 | COMPASS-Shg1 | NA | COMPASS-Shg1 |
| 135 | PF00082 | Vitvi02g00442.t01 | PA | Peptidase_S8 | PA |
| 136 | PF00082 | Vitvi02g00443.t01 | PA | Peptidase_S8 | PA |
| 137 | PF00080 | Vitvi02g00444.t01 | NA | Sod_Cu | Sod_Cu |
| 138 | PF00293 | Vitvi02g00445.t01 | NA | NUDIX | NUDIX |
| 139 | PF00067 | Vitvi02g00446.t01 | NA | p450 | p450 |
| 140 | NA | Vitvi02g00447.t01 | NA | NA | Vitvi02g00447 |
| 141 | NA | Vitvi02g00448.t01 | NA | NA | Vitvi02g00448 |
| 142 | PF08879 | Vitvi02g00449.t01 | NA | WRC | WRC |
| 143 | NA | Vitvi02g00450.t01 | TPT | NA | TPT |
| 144 | NA | Vitvi02g00451.t01 | SLAC1 | NA | SLAC1 |
| 145 | NA | Vitvi02g00452.t01 | Neprosin | NA | Neprosin |
| 146 | NA | Vitvi02g00453.t01 | NA | NA | Vitvi02g00453 |
| 147 | NA | Vitvi02g00454.t01 | TPX2_importin | NA | TPX2_importin |
| 148 | NA | Vitvi02g00455.t01 | NA | NA | Vitvi02g00455 |
| 149 | PF00082 | Vitvi02g00456.t01 | PA | Peptidase_S8 | PA |
| 150 | PF00155 | Vitvi02g00457.t01 | NA | Aminotran_1_2 | Aminotran_1_2 |
| 151 | PF14604 | Vitvi02g00458.t01 | SNARE_assoc | SH3_9 | SNARE_assoc |
| 152 | NA | Vitvi02g00459.t01 | DUF2499 | NA | DUF2499 |
| 153 | NA | Vitvi02g00460.t01 | NA | NA | Vitvi02g00460 |
| 154 | PF03604 | Vitvi02g00461.t01 | zf-A20 | DNA_RNApol_7kD | zf-A20 |
| 155 | PF00225 | Vitvi02g00462.t01 | Arm_2 | Kinesin | Arm_2 |
| 156 | NA | Vitvi02g00463.t01 | zf-AN1 | NA | zf-AN1 |
| 157 | PF00225 | Vitvi02g00464.t01 | HEAT_2 | Kinesin | HEAT_2 |
| 158 | PF03479 | Vitvi02g00465.t01 | NA | DUF296 | DUF296 |
| 159 | NA | Vitvi02g00466.t01 | MatE | NA | MatE |
| 160 | PF01842 | Vitvi02g00467.t01 | NA | ACT | ACT |
| 161 | PF03479 | Vitvi02g00468.t01 | NA | DUF296 | DUF296 |
| 162 | PF00574 | Vitvi02g00469.t01 | NA | CLP_protease | CLP_protease |
| 163 | NA | Vitvi02g00470.t01 | Tfb5 | NA | Tfb5 |
| 164 | NA | Vitvi02g00471.t01 | Cornichon | NA | Cornichon |
| 165 | NA | Vitvi02g00472.t01 | NA | NA | Vitvi02g00472 |
| 166 | NA | Vitvi02g00473.t01 | NA | NA | Vitvi02g00473 |
| 167 | PF17177 | Vitvi02g00474.t01 | PPR | PPR_long | PPR |
| 168 | PF00875 | Vitvi02g00475.t01 | NA | DNA_photolyase | DNA_photolyase |
| 169 | NA | Vitvi02g00476.t01 | DUF659 | NA | DUF659 |
| 170 | NA | Vitvi02g00477.t01 | DUF724 | NA | DUF724 |
| 171 | NA | Vitvi02g00478.t01 | NA | NA | Vitvi02g00478 |
| 172 | PF04545 | Vitvi02g00479.t01 | Mlf1IP | Sigma70_r4 | Mlf1IP |
| 173 | NA | Vitvi02g00480.t01 | RF-1 | NA | RF-1 |
| 174 | NA | Vitvi02g00481.t01 | DUF1442 | NA | DUF1442 |
| 175 | PF08669 | Vitvi02g00482.t01 | NA | GCV_T_C | GCV_T_C |
| 176 | PF07714 | Vitvi02g00483.t01 | LRRNT_2 | Pkinase_Tyr | LRRNT_2 |
| 177 | NA | Vitvi02g00484.t01 | NA | NA | Vitvi02g00484 |
| 178 | PF13499 | Vitvi02g00485.t01 | Caleosin | EF-hand_7 | Caleosin |
| 179 | PF00561 | Vitvi02g00486.t01 | Hydrolase_4 | Abhydrolase_1 | Hydrolase_4 |
| 180 | NA | Vitvi02g00487.t01 | NA | NA | Vitvi02g00487 |
| 181 | PF01936 | Vitvi02g00488.t01 | DUF629 | NYN | DUF629 |
| 182 | PF16363 | Vitvi02g00489.t01 | Epimerase | GDP_Man_Dehyd | Epimerase |
| 183 | NA | Vitvi02g00490.t01 | NA | NA | Vitvi02g00490 |
| 184 | NA | Vitvi02g00491.t01 | NA | NA | Vitvi02g00491 |
| 185 | NA | Vitvi02g00492.t01 | Retrotran_gag_2 | NA | Retrotran_gag_2 |
| 186 | NA | Vitvi02g01376.t01 | NA | NA | Vitvi02g01376 |
| 187 | NA | Vitvi02g01377.t01 | NA | NA | Vitvi02g01377 |
| 188 | NA | Vitvi02g01378.t01 | NA | NA | Vitvi02g01378 |
| 189 | NA | Vitvi02g01379.t01 | BORCS6 | NA | BORCS6 |
| 190 | PF13476 | Vitvi02g01380.t01 | PEARLI-4 | AAA_23 | PEARLI-4 |
| 191 | NA | LOC100264973.t01 | NA | NA | LOC100264973 |
| 192 | PF00702 | Vitvi02g01382.t01 | E1-E2_ATPase | Hydrolase | E1-E2_ATPase |
| 193 | PF00702 | Vitvi02g01383.t01 | E1-E2_ATPase | Hydrolase | E1-E2_ATPase |
| 194 | NA | Vitvi02g01384.t01 | Glyco_transf_90 | NA | Glyco_transf_90 |
| 195 | PF04525 | Vitvi02g01385.t01 | NA | LOR | LOR |
| 196 | NA | Vitvi02g01386.t01 | NA | NA | Vitvi02g01386 |
| 197 | NA | Vitvi02g01387.t01 | NA | NA | Vitvi02g01387 |
| 198 | NA | Vitvi02g01388.t01 | NA | NA | Vitvi02g01388 |
| 199 | NA | Vitvi02g01389.t01 | NA | NA | Vitvi02g01389 |
| 200 | NA | Vitvi02g01390.t01 | Neprosin | NA | Neprosin |
| 201 | NA | Vitvi02g01391.t01 | NA | NA | Vitvi02g01391 |
| 202 | NA | Vitvi02g01392.t01 | Neprosin | NA | Neprosin |
| 203 | NA | Vitvi02g01393.t01 | PPI_Ypi1 | NA | PPI_Ypi1 |
| 204 | NA | Vitvi02g01394.t01 | NA | NA | Vitvi02g01394 |
| 205 | PF00067 | Vitvi02g01395.t01 | NA | p450 | p450 |
| 206 | NA | Vitvi02g01396.t01 | NA | NA | Vitvi02g01396 |
| 207 | PF00067 | Vitvi02g01397.t01 | NA | p450 | p450 |
| 208 | NA | LOC100263341.t01 | NA | NA | LOC100263341 |
| 209 | PF04535 | Vitvi02g01399.t01 | NA | DUF588 | DUF588 |
| 210 | NA | LOC100263258.t01 | NA | NA | LOC100263258 |
| 211 | NA | Vitvi02g01401.t01 | NA | NA | Vitvi02g01401 |
| 212 | NA | Vitvi02g01402.t01 | Retrotran_gag_2 | NA | Retrotran_gag_2 |
| 213 | PF00314 | Vitvi02g01403.t01 | NA | Thaumatin | Thaumatin |
| 214 | PF00314 | Vitvi02g01404.t01 | NA | Thaumatin | Thaumatin |
| 215 | PF00314 | Vitvi02g01405.t01 | NA | Thaumatin | Thaumatin |
| 216 | PF00314 | Vitvi02g01406.t01 | NA | Thaumatin | Thaumatin |
| 217 | PF00314 | Vitvi02g01407.t01 | NA | Thaumatin | Thaumatin |
| 218 | PF00314 | Vitvi02g01408.t01 | NA | Thaumatin | Thaumatin |
| 219 | PF00314 | Vitvi02g01409.t01 | NA | Thaumatin | Thaumatin |
| 220 | NA | Vitvi02g01410.t01 | NA | NA | Vitvi02g01410 |
| 221 | PF00082 | Vitvi02g01411.t01 | PA | Peptidase_S8 | PA |
| 222 | PF00082 | Vitvi02g01412.t01 | PA | Peptidase_S8 | PA |
| 223 | PF00082 | Vitvi02g01413.t01 | PA | Peptidase_S8 | PA |
| 224 | NA | Vitvi02g01414.t01 | NA | NA | Vitvi02g01414 |
| 225 | PF00067 | Vitvi02g01415.t01 | NA | p450 | p450 |
| 226 | PF00067 | Vitvi02g01416.t01 | NA | p450 | p450 |
| 227 | NA | Vitvi02g01417.t01 | NA | NA | Vitvi02g01417 |
| 228 | NA | Vitvi02g01418.t01 | NA | NA | Vitvi02g01418 |
| 229 | NA | Vitvi02g01419.t01 | NA | NA | Vitvi02g01419 |
| 230 | NA | Vitvi02g01420.t01 | NA | NA | Vitvi02g01420 |
| 231 | NA | Vitvi02g01421.t01 | NA | NA | Vitvi02g01421 |
| 232 | NA | Vitvi02g01422.t01 | NA | NA | Vitvi02g01422 |
| 233 | NA | Vitvi02g01423.t01 | NA | NA | Vitvi02g01423 |
| 234 | NA | Vitvi02g01424.t01 | NA | NA | Vitvi02g01424 |
| 235 | NA | Vitvi02g01425.t01 | NA | NA | Vitvi02g01425 |
| 236 | NA | Vitvi02g01426.t01 | NA | NA | Vitvi02g01426 |
| 237 | NA | VviGRAS8a.t01 | NA | NA | VviGRAS8a |
| 238 | PF00067 | Vitvi02g00493.t01 | NA | p450 | p450 |
| 239 | NA | Vitvi02g00494.t01 | NA | NA | Vitvi02g00494 |
| 240 | PF00067 | Vitvi02g01427.t01 | NA | p450 | p450 |

**Supplementary Table 9B. Summer lateral QTL gene id and PFAM name for *V. riparia* and *V. vinifera*.**

| ***V. riparia*** | |  |  |  |  |
| --- | --- | --- | --- | --- | --- |
|  | **PFAM** | **SeqID** | **PFAM_NAME** | **DOM_NAME** | **BRIEF NAME** |
|  | PF13639 | g220.t1 | zf-ANAPC11 | zf-RING_2 | zf-ANAPC11 |
|  | NA | g221.t1 | DUF810 | NA | DUF810 |
|  | PF00168 | g222.t1 | DUF641 | C2 | DUF641 |
|  | NA | g223.t1 | NA | NA | g223.t1 |
|  | PF00447 | g224.t1 | HALZ | HSF_DNA-bind | HALZ |
|  | NA | g225.t1 | IPP-2 | NA | IPP-2 |
|  | PF00270 | g226.t1 | Helicase_C | DEAD | Helicase_C |
|  | PF04535 | g227.t1 | Methyltr_RsmB-F | DUF588 | Methyltr_RsmB-F |
|  | PF16363 | g228.t1 | Epimerase | GDP_Man_Dehyd | Epimerase |
|  | PF00249 | g229.t1 | Big_2 | Myb_DNA-binding | Big_2 |
|  | PF00314 | g230.t1 | IKI3 | Thaumatin | IKI3 |
|  | PF00314 | g231.t1 | NA | Thaumatin | Thaumatin |
|  | PF00314 | g232.t1 | NA | Thaumatin | Thaumatin |
|  | PF00314 | g233.t1 | NA | Thaumatin | Thaumatin |
|  | PF00314 | g234.t1 | GPHR_N | Thaumatin | GPHR_N |
|  | PF00314 | g235.t1 | NA | Thaumatin | Thaumatin |
|  | PF00314 | g236.t1 | NA | Thaumatin | Thaumatin |
|  | PF00314 | g237.t1 | NA | Thaumatin | Thaumatin |
|  | PF00314 | g238.t1 | PPR | Thaumatin | PPR |
|  | PF00314 | g239.t1 | NA | Thaumatin | Thaumatin |
|  | PF00168 | g240.t1 | NA | C2 | C2 |
|  | PF00067 | g241.t1 | NA | p450 | p450 |
|  | PF00067 | g242.t1 | NA | p450 | p450 |
|  | PF00646 | g243.t1 | PP2 | F-box | PP2 |
|  | PF04618 | g244.t1 | LIM_bind | HD-ZIP_N | LIM_bind |
|  | NA | g245.t1 | MBOAT | NA | MBOAT |
|  | PF00702 | g246.t1 | E1-E2_ATPase | Hydrolase | E1-E2_ATPase |
|  | PF01661 | g247.t1 | MatE | Macro | MatE |
|  | NA | g248.t1 | NifU | NA | NifU |
|  | PF00847 | g249.t1 | NA | AP2 | AP2 |
|  | PF00847 | g250.t1 | Aldose_epim | AP2 | Aldose_epim |
|  | PF02209 | g251.t1 | DUF4378 | VHP | DUF4378 |
|  | PF00626 | g252.t1 | UAA | Gelsolin | UAA |
|  | NA | g253.t1 | Ran_BP1 | NA | Ran_BP1 |
|  | PF00106 | g254.t1 | Epimerase | adh_short | Epimerase |
|  | PF00069 | g255.t1 | RIO1 | Pkinase | RIO1 |
|  | PF00623 | g256.t1 | DUF3223 | RNA_pol_Rpb1_2 | DUF3223 |
|  | PF03105 | g257.t1 | MFS_1 | SPX | MFS_1 |
|  | PF00732 | g258.t1 | Lycopene_cycl | GMC_oxred_N | Lycopene_cycl |
|  | PF01179 | g259.t1 | CDP-OH_P_transf | Cu_amine_oxid | CDP-OH_P_transf |
|  | NA | g260.t1 | IBB | NA | IBB |
|  | PF08540 | g261.t1 | NA | HMG_CoA_synt_C | HMG_CoA_synt_C |
|  | PF00320 | g262.t1 | TCP | GATA | TCP |
|  | PF00082 | g263.t1 | RNase_T | Peptidase_S8 | RNase_T |
|  | PF04321 | g264.t1 | Epimerase | RmlD_sub_bind | Epimerase |
|  | PF00168 | g265.t1 | PLD_C | C2 | PLD_C |
|  | PF00046 | g266.t1 | KNOX2 | Homeobox | KNOX2 |
|  | PF04564 | g267.t1 | MINDY_DUB | U-box | MINDY_DUB |
|  | PF00319 | g268.t1 | K-box | SRF-TF | K-box |
|  | PF12638 | g269.t1 | Prok-RING_4 | Staygreen | Prok-RING_4 |
|  | PF12638 | g270.t1 | zf-ANAPC11 | Staygreen | zf-ANAPC11 |
|  | NA | g271.t1 | eIF2A | NA | eIF2A |
|  | NA | g272.t1 | DUF1191 | NA | DUF1191 |
|  | PF03330 | g273.t1 | TPT | DPBB_1 | TPT |
|  | PF01096 | g274.t1 | TPT | TFIIS_C | TPT |
|  | PF03171 | g275.t1 | DIOX_N | 2OG-FeII_Oxy | DIOX_N |
|  | PF02826 | g276.t1 | F420_oxidored | 2-Hacid_dh_C | F420_oxidored |
|  | PF02826 | g277.t1 | F420_oxidored | 2-Hacid_dh_C | F420_oxidored |
|  | PF00010 | g278.t1 | HSP20 | HLH | HSP20 |
|  | PF04146 | g279.t1 | NA | YTH | YTH |
|  | NA | g280.t1 | COMPASS-Shg1 | NA | COMPASS-Shg1 |
|  | PF00082 | g281.t1 | PA | Peptidase_S8 | PA |
|  | PF00082 | g282.t1 | PA | Peptidase_S8 | PA |
|  | PF00249 | g283.t1 | Myb_CC_LHEQLE | Myb_DNA-binding | Myb_CC_LHEQLE |
|  | PF00082 | g284.t1 | NA | Peptidase_S8 | Peptidase_S8 |
|  | PF00082 | g285.t1 | PA | Peptidase_S8 | PA |
|  | PF00082 | g286.t1 | PA | Peptidase_S8 | PA |
|  | PF00080 | g287.t1 | NA | Sod_Cu | Sod_Cu |
|  | PF00293 | g288.t1 | zf-TAZ | NUDIX | zf-TAZ |
|  | PF00067 | g289.t1 | PMSR | p450 | PMSR |
|  | NA | g290.t1 | NA | NA | g290.t1 |
|  | PF00005 | g291.t1 | K_trans | ABC_tran | K_trans |
|  | PF00067 | g292.t1 | Nup160 | p450 | Nup160 |
|  | PF00067 | g293.t1 | NA | p450 | p450 |
|  | PF00067 | g294.t1 | BORCS8 | p450 | BORCS8 |
|  | PF08801 | g295.t1 | Nup160 | Nucleoporin_N | Nup160 |
|  | PF08879 | g296.t1 | NA | WRC | WRC |
|  | PF00069 | g297.t1 | TPT | Pkinase | TPT |
|  | PF02298 | g298.t1 | STPPase_N | Cu_bind_like | STPPase_N |
|  | PF02298 | g299.t1 | LBP_BPI_CETP | Cu_bind_like | LBP_BPI_CETP |
|  | PF00628 | g300.t1 | SLAC1 | PHD | SLAC1 |
|  | NA | g301.t1 | Neprosin | NA | Neprosin |
|  | NA | g302.t1 | Neprosin_AP | NA | Neprosin_AP |
|  | NA | g303.t1 | AA_permease | NA | AA_permease |
|  | PF08711 | g304.t1 | TPX2_importin | Med26 | TPX2_importin |
|  | PF00082 | g305.t1 | PA | Peptidase_S8 | PA |
|  | PF05922 | g306.t1 | NA | Inhibitor_I9 | Inhibitor_I9 |
|  | PF00155 | g307.t1 | CTP_transf_1 | Aminotran_1_2 | CTP_transf_1 |
|  | PF00069 | g308.t1 | SNARE_assoc | Pkinase | SNARE_assoc |
|  | NA | g309.t1 | SBF | NA | SBF |
|  | NA | g310.t1 | DUF2499 | NA | DUF2499 |
|  | NA | g311.t1 | Lipase_3 | NA | Lipase_3 |
|  | NA | g312.t1 | NA | NA | g312.t1 |
|  | PF03604 | g313.t1 | zf-A20 | DNA_RNApol_7kD | zf-A20 |
|  | PF00225 | g314.t1 | Arm_2 | Kinesin | Arm_2 |
|  | PF03479 | g315.t1 | ASL_C | DUF296 | ASL_C |
|  | NA | g316.t1 | MatE | NA | MatE |
|  | PF05193 | g317.t1 | M16C_assoc | Peptidase_M16_C | M16C_assoc |
|  | PF07859 | g318.t1 | NA | Abhydrolase_3 | Abhydrolase_3 |
|  | PF01842 | g319.t1 | Chlorophyllase2 | ACT | Chlorophyllase2 |
|  | PF03479 | g320.t1 | Chlorophyllase2 | DUF296 | Chlorophyllase2 |
|  | PF07859 | g321.t1 | TRAP_alpha | Abhydrolase_3 | TRAP_alpha |
|  | PF00574 | g322.t1 | NA | CLP_protease | CLP_protease |
|  | PF07859 | g323.t1 | Cornichon | Abhydrolase_3 | Cornichon |
|  | PF07859 | g324.t1 | NA | Abhydrolase_3 | Abhydrolase_3 |
|  | NA | g325.t1 | NA | NA | g325.t1 |
|  | PF17177 | g326.t1 | PPR | PPR_long | PPR |
|  | PF03114 | g327.t1 | FA_hydroxylase | BAR | FA_hydroxylase |
|  | NA | g328.t1 | DUF659 | NA | DUF659 |
|  | PF00875 | g329.t1 | NA | DNA_photolyase | DNA_photolyase |
|  | NA | g330.t1 | NAM | NA | NAM |
|  | NA | g331.t1 | DUF724 | NA | DUF724 |
|  | PF02953 | g332.t1 | DHHC | zf-Tim10_DDP | DHHC |
|  | PF00035 | g333.t1 | RF-1 | dsrm | RF-1 |
|  | PF00069 | g334.t1 | DUF1442 | Pkinase | DUF1442 |
|  | PF08669 | g335.t1 | NA | GCV_T_C | GCV_T_C |
|  | PF07714 | g336.t1 | LRRNT_2 | Pkinase_Tyr | LRRNT_2 |
|  | PF00462 | g337.t1 | NA | Glutaredoxin | Glutaredoxin |
|  | PF13499 | g338.t1 | Caleosin | EF-hand_7 | Caleosin |
|  | PF00561 | g339.t1 | Hydrolase_4 | Abhydrolase_1 | Hydrolase_4 |
|  | PF01936 | g340.t1 | NA | NYN | NYN |
|  | PF16363 | g341.t1 | Epimerase | GDP_Man_Dehyd | Epimerase |
|  | PF01179 | g342.t1 | Pollen_Ole_e_I | Cu_amine_oxid | Pollen_Ole_e_I |
|  | PF00067 | g343.t1 | Actin | p450 | Actin |
|  | PF00067 | g344.t1 | CLPTM1 | p450 | CLPTM1 |
|  | PF01179 | g345.t1 | NA | Cu_amine_oxid | Cu_amine_oxid |
|  | PF00067 | g346.t1 | NA | p450 | p450 |
|  | PF00067 | g347.t1 | NA | p450 | p450 |
|  | PF01842 | g348.t1 | Med23 | ACT | Med23 |
|  | PF13962 | g349.t1 | TCP | PGG | TCP |
|  | NA | g350.t1 | OPT | NA | OPT |
|  | PF13962 | g351.t1 | NA | PGG | PGG |
|  | PF00749 | g352.t1 | tRNA_synt_1c_R1 | tRNA-synt_1c | tRNA_synt_1c_R1 |
|  | PF13837 | g353.t1 | DUF573 | Myb_DNA-bind_4 | DUF573 |
|  | PF00160 | g354.t1 | Acetyltransf_1 | Pro_isomerase | Acetyltransf_1 |
|  | PF07992 | g355.t1 | Glyco_transf_9 | Pyr_redox_2 | Glyco_transf_9 |
|  | NA | g356.t1 | PEMT | NA | PEMT |
|  | PF00076 | g357.t1 | Adenine_glyco | RRM_1 | Adenine_glyco |
|  | PF03106 | g358.t1 | Nucleotid_trans | WRKY | Nucleotid_trans |
|  | NA | g359.t1 | NA | NA | g359.t1 |
|  | PF02115 | g360.t1 | Nucleotid_trans | Rho_GDI | Nucleotid_trans |
|  | NA | g361.t1 | NA | NA | g361.t1 |
|  | PF00307 | g362.t1 | NA | CH | CH |
|  | PF08059 | g363.t1 | NA | SEP | SEP |
|  | PF12047 | g364.t1 | Cript | DNMT1-RFD | Cript |
|  | PF00071 | g365.t1 | RabGAP-TBC | Ras | RabGAP-TBC |
|  | PF06747 | g366.t1 | GRP | CHCH | GRP |
|  | PF00067 | g367.t1 | NA | p450 | p450 |
|  | PF00067 | g368.t1 | GRP | p450 | GRP |
|  | PF00067 | g369.t1 | Topoisom_I_N | p450 | Topoisom_I_N |
|  | PF00651 | g2898.t1 | Sec1 | BTB | Sec1 |
|  | PF13279 | g2899.t1 | ORMDL | NA | ORMDL |
|  | PF01363 | g2900.t1 | zf-Dof | FYVE | zf-Dof |
|  | PF16529 | g2901.t1 | Nup160 | Ge1_WD40 | Nup160 |
|  | PF00562 | g2902.t1 | RNA_pol_Rpb2_1 | RNA_pol_Rpb2_6 | RNA_pol_Rpb2_1 |
|  | PF00249 | g2903.t1 | NA | Myb_DNA-binding | Myb_DNA-binding |
|  | PF13489 | g2904.t1 | Rsm22 | Methyltransf_23 | Rsm22 |
|  | PF00106 | g2905.t1 | Epimerase | adh_short | Epimerase |
|  | PF01204 | g2906.t1 | Chlorophyllase2 | Trehalase | Chlorophyllase2 |
|  | NA | g2907.t1 | Neprosin_AP | NA | Neprosin_AP |
|  | PF16770 | g2908.t1 | Neprosin | RTT107_BRCT_5 | Neprosin |
|  | NA | g2909.t1 | Neprosin | NA | Neprosin |
|  | PF00005 | g2910.t1 | CN_hydrolase | ABC_tran | CN_hydrolase |
|  | PF02782 | g2911.t1 | HSP70 | FGGY_C | HSP70 |
|  | PF03126 | g2912.t1 | ETC_C1_NDUFA5 | Plus-3 | ETC_C1_NDUFA5 |
|  | PF01373 | g2913.t1 | NA | Glyco_hydro_14 | Glyco_hydro_14 |
|  | NA | g2914.t1 | DUF313 | NA | DUF313 |
|  | PF14559 | g2915.t1 | TPR_16 | TPR_19 | TPR_16 |
|  | PF16860 | g2916.t1 | MtN3_slv | CX9C | MtN3_slv |
|  | PF00564 | g2917.t1 | RWP-RK | PB1 | RWP-RK |
|  | NA | g2918.t1 | TRAPP | NA | TRAPP |
|  | NA | g2919.t1 | DUF1242 | NA | DUF1242 |
|  | PF01487 | g2920.t1 | NA | DHquinase_I | DHquinase_I |
|  | PF01487 | g2921.t1 | DUF2439 | DHquinase_I | DUF2439 |
|  | PF13901 | g2922.t1 | Shikimate_DH | zf-RING_9 | Shikimate_DH |
|  | NA | g2923.t1 | CSN8_PSD8_EIF3K | NA | CSN8_PSD8_EIF3K |
|  | PF01250 | g2924.t1 | Ribosomal_L18p | Ribosomal_S6 | Ribosomal_L18p |
|  | PF13639 | g2925.t1 | zf-ANAPC11 | zf-RING_2 | zf-ANAPC11 |
|  | PF13632 | g2926.t1 | Cellulose_synt | Glyco_trans_2_3 | Cellulose_synt |
|  | PF12678 | g2927.t1 | zf-ANAPC11 | zf-rbx1 | zf-ANAPC11 |
|  | PF13632 | g2928.t1 | Cellulose_synt | Glyco_trans_2_3 | Cellulose_synt |
|  | PF13632 | g2929.t1 | Cellulose_synt | Glyco_trans_2_3 | Cellulose_synt |
|  | PF13632 | g2930.t1 | Cellulose_synt | Glyco_trans_2_3 | Cellulose_synt |
|  | NA | g2931.t1 | NA | NA | g2931.t1 |
|  | PF13632 | g2932.t1 | Cellulose_synt | Glyco_trans_2_3 | Cellulose_synt |
|  | PF00168 | g2933.t1 | Cellulose_synt | C2 | Cellulose_synt |
|  | NA | g2934.t1 | Raffinose_syn | NA | Raffinose_syn |
|  | NA | g2935.t1 | Cellulose_synt | NA | Cellulose_synt |
|  | PF01423 | g2936.t1 | Cellulose_synt | LSM | Cellulose_synt |
|  | PF13947 | g2937.t1 | Cellulose_synt | GUB_WAK_bind | Cellulose_synt |
|  | NA | g2938.t1 | NA | NA | g2938.t1 |
|  | PF00295 | g2939.t1 | Cellulose_synt | Glyco_hydro_28 | Cellulose_synt |
|  | PF13632 | g2940.t1 | Cellulose_synt | Glyco_trans_2_3 | Cellulose_synt |
|  | PF08144 | g2941.t1 | Cellulose_synt | CPL | Cellulose_synt |
|  | PF00226 | g2942.t1 | NPR1_interact | DnaJ | NPR1_interact |
|  | PF13632 | g2943.t1 | Cellulose_synt | Glyco_trans_2_3 | Cellulose_synt |
|  | PF13632 | g2944.t1 | Cellulose_synt | Glyco_trans_2_3 | Cellulose_synt |
|  | NA | g2945.t1 | Cellulose_synt | NA | Cellulose_synt |
|  | PF13632 | g2946.t1 | Cellulose_synt | Glyco_trans_2_3 | Cellulose_synt |
|  | NA | g2947.t1 | Auxin_resp | NA | Auxin_resp |
|  | PF13091 | g2948.t1 | PLDc | PLDc_2 | PLDc |
|  | PF00109 | g2949.t1 | Ribosomal_L19e | ketoacyl-synt | Ribosomal_L19e |
|  | PF10539 | g2950.t1 | NA | Dev_Cell_Death | Dev_Cell_Death |
|  | PF00462 | g2951.t1 | NA | Glutaredoxin | Glutaredoxin |
|  | NA | g2952.t1 | NA | NA | g2952.t1 |
|  | PF02229 | g2953.t1 | NA | PC4 | PC4 |
|  | PF13474 | g2954.t1 | NA | SnoaL_3 | SnoaL_3 |
|  | NA | g2955.t1 | Sad1_UNC | NA | Sad1_UNC |
|  | PF13920 | g2956.t1 | Sina | zf-C3HC4_3 | Sina |
|  | PF00628 | g2957.t1 | EloA-BP1 | PHD | EloA-BP1 |
|  | PF00036 | g2958.t1 | EF-hand_4 | EF-hand_1 | EF-hand_4 |
|  | PF00036 | g2959.t1 | EF-hand_4 | EF-hand_1 | EF-hand_4 |
|  | NA | g2960.t1 | Lipase_GDSL | NA | Lipase_GDSL |
|  | PF00240 | g2961.t1 | PI3_PI4_kinase | ubiquitin | PI3_PI4_kinase |
|  | PF03168 | g2962.t1 | Piezo_RRas_bdg | LEA_2 | Piezo_RRas_bdg |
|  | PF12697 | g2963.t1 | Lipase_3 | Abhydrolase_6 | Lipase_3 |
|  | PF16363 | g2964.t1 | Epimerase | GDP_Man_Dehyd | Epimerase |
|  | PF00350 | g2965.t1 | Dynamin_M | Dynamin_N | Dynamin_M |
|  | PF16363 | g2966.t1 | Epimerase | GDP_Man_Dehyd | Epimerase |
|  | NA | g2967.t1 | DUF1639 | NA | DUF1639 |
|  | NA | g2968.t1 | PP2C | NA | PP2C |
|  | NA | g2969.t1 | PP2C | NA | PP2C |
|  | NA | g2970.t1 | NA | NA | g2970.t1 |
|  | PF03351 | g2971.t1 | Cytochrom_B561 | DOMON | Cytochrom_B561 |
|  | NA | g2972.t1 | PPR | NA | PPR |
|  | PF00334 | g2973.t1 | Aa_trans | NDK | Aa_trans |
|  | NA | g2974.t1 | MitMem_reg | NA | MitMem_reg |
|  | PF00169 | g2975.t1 | Peptidase_M41 | PH | Peptidase_M41 |
|  | PF13365 | g2976.t1 | NA | Trypsin_2 | Trypsin_2 |
|  | PF00270 | g2977.t1 | NdhS | DEAD | NdhS |
|  | PF13637 | g2978.t1 | zf-CCCH | Ank_4 | zf-CCCH |
|  | PF00365 | g2979.t1 | DUF4228 | PFK | DUF4228 |
|  | PF00628 | g2980.t1 | zf-UBR | PHD | zf-UBR |
|  | PF13639 | g2981.t1 | 60KD_IMP | zf-RING_2 | 60KD_IMP |
|  | NA | g2982.t1 | AMP-binding | NA | AMP-binding |
|  | NA | g2983.t1 | DUF2428 | NA | DUF2428 |
|  | PF10557 | g2984.t1 | PP2C | Cullin_Nedd8 | PP2C |
|  | PF00759 | g2985.t1 | ATP-synt_Eps | Glyco_hydro_9 | ATP-synt_Eps |
|  | PF00266 | g2986.t1 | NA | Aminotran_5 | Aminotran_5 |
|  | PF08167 | g2987.t1 | Ribosomal_S4 | RIX1 | Ribosomal_S4 |
|  | NA | g2988.t1 | DNA_primase_S | NA | DNA_primase_S |
|  | PF00295 | g2989.t1 | Polyketide_cyc2 | Glyco_hydro_28 | Polyketide_cyc2 |
|  | PF00091 | g2990.t1 | NA | Tubulin | Tubulin |
|  | PF00005 | g2991.t1 | ABC_membrane | ABC_tran | ABC_membrane |
|  | PF00069 | g2992.t1 | NA | Pkinase | Pkinase |
|  | PF05922 | g2993.t1 | NA | Inhibitor_I9 | Inhibitor_I9 |
|  | PF03106 | g2994.t1 | Hydrolase_4 | WRKY | Hydrolase_4 |
|  | PF13460 | g2995.t1 | 3Beta_HSD | NAD_binding_10 | 3Beta_HSD |
|  | PF16529 | g2996.t1 | DUF629 | Ge1_WD40 | DUF629 |
|  | NA | g2997.t1 | UDPGT | NA | UDPGT |
|  | NA | g2998.t1 | PPR | NA | PPR |
|  | PF12895 | g2999.t1 | Aa_trans | ANAPC3 | Aa_trans |
|  | PF00505 | g3000.t1 | DUF629 | HMG_box | DUF629 |
|  | PF14416 | g3001.t1 | PC-Esterase | PMR5N | PC-Esterase |
|  | PF13874 | g3002.t1 | zinc_ribbon_12 | Nup54 | zinc_ribbon_12 |
|  | PF04601 | g3003.t1 | NA | DUF569 | DUF569 |
|  | NA | g3004.t1 | Mito_carr | NA | Mito_carr |
|  | NA | g3005.t1 | KIP1 | NA | KIP1 |
|  | PF14559 | g3006.t1 | HAT | TPR_19 | HAT |
|  | NA | g3007.t1 | Branch | NA | Branch |
|  | NA | g3008.t1 | Med22 | NA | Med22 |
|  | NA | g3009.t1 | Aldedh | NA | Aldedh |
|  | PF00069 | g3010.t1 | NTP_transferase | Pkinase | NTP_transferase |
|  | PF07714 | g3011.t1 | DUF1221 | Pkinase_Tyr | DUF1221 |
|  | PF01074 | g3012.t1 | NA | Glyco_hydro_38 | Glyco_hydro_38 |
|  | NA | g3013.t1 | IKI3 | NA | IKI3 |
|  | PF02319 | g3014.t1 | AGP | E2F_TDP | AGP |
|  | PF06747 | g3015.t1 | DUF760 | CHCH | DUF760 |
|  | PF02298 | g3016.t1 | Glyco_transf_8 | Cu_bind_like | Glyco_transf_8 |
|  | PF14570 | g3017.t1 | NA | zf-RING_4 | zf-RING_4 |
|  | PF00179 | g3018.t1 | Sin3a_C | UQ_con | Sin3a_C |
|  | PF00069 | g3019.t1 | LRRNT_2 | Pkinase | LRRNT_2 |
|  | PF05190 | g3020.t1 | Aldose_epim | MutS_IV | Aldose_epim |
|  | PF08801 | g3021.t1 | MFMR | Nucleoporin_N | MFMR |
|  | PF00106 | g3022.t1 | Galactosyl_T | adh_short | Galactosyl_T |
|  | PF00036 | g3023.t1 | vATP-synt_E | EF-hand_1 | vATP-synt_E |
|  | PF00005 | g3024.t1 | ABC_membrane | ABC_tran | ABC_membrane |
|  | PF00005 | g3025.t1 | ABC_membrane | ABC_tran | ABC_membrane |
|  | PF00005 | g3026.t1 | ABC_membrane | ABC_tran | ABC_membrane |
|  | PF00005 | g3027.t1 | ABC_membrane | ABC_tran | ABC_membrane |
|  | PF00005 | g3028.t1 | ABC_membrane | ABC_tran | ABC_membrane |
|  | PF00005 | g3029.t1 | ABC_membrane | ABC_tran | ABC_membrane |
|  | PF00393 | g3030.t1 | NA | 6PGD | 6PGD |
|  | NA | g3031.t1 | DUF1191 | NA | DUF1191 |
|  | PF00651 | g3032.t1 | WSD | BTB | WSD |
|  | PF03417 | g3033.t1 | ATP-synt_E | AAT | ATP-synt_E |
|  | PF00903 | g3034.t1 | NA | Glyoxalase | Glyoxalase |
|  | PF00249 | g3035.t1 | LMBR1 | Myb_DNA-binding | LMBR1 |
|  | PF00646 | g3036.t1 | NA | F-box | F-box |
|  | PF00582 | g3037.t1 | Na_H_Exchanger | Usp | Na_H_Exchanger |
|  | PF00582 | g3038.t1 | Na_H_Exchanger | Usp | Na_H_Exchanger |
|  | PF00582 | g3039.t1 | Na_H_Exchanger | Usp | Na_H_Exchanger |
|  | PF04253 | g3040.t1 | Dirigent | TFR_dimer | Dirigent |
|  | PF04253 | g3041.t1 | Dirigent | TFR_dimer | Dirigent |
|  | NA | g3042.t1 | Dirigent | NA | Dirigent |
|  | PF12895 | g3043.t1 | UPF0016 | ANAPC3 | UPF0016 |
|  | NA | g3044.t1 | PPR | NA | PPR |
|  | NA | g3045.t1 | ALMT | NA | ALMT |
|  | PF00069 | g3046.t1 | EF-hand_4 | Pkinase | EF-hand_4 |
|  | NA | g3047.t1 | K_trans | NA | K_trans |
|  | PF00394 | g3048.t1 | Abi | Cu-oxidase | Abi |
|  | PF00004 | g3049.t1 | TIP49 | AAA | TIP49 |
|  | PF07732 | g3050.t1 | Reticulon | Cu-oxidase_3 | Reticulon |
|  | PF14693 | g3051.t1 | zf-Dof | Ribosomal_TL5_C | zf-Dof |
|  | PF00150 | g3052.t1 | ATG101 | Cellulase | ATG101 |
|  | PF00150 | g3053.t1 | NA | Cellulase | Cellulase |
|  | PF00150 | g3054.t1 | NA | Cellulase | Cellulase |
|  | PF00150 | g3055.t1 | NA | Cellulase | Cellulase |
|  | PF14200 | g3056.t1 | Ferric_reduct | RicinB_lectin_2 | Ferric_reduct |
|  | PF00150 | g3057.t1 | NA | Cellulase | Cellulase |
|  | PF10241 | g3058.t1 | Vps51 | KxDL | Vps51 |
|  | PF03168 | g3059.t1 | NA | LEA_2 | LEA_2 |
|  | PF03168 | g3060.t1 | Fn3-like | LEA_2 | Fn3-like |
|  | PF14416 | g3061.t1 | PC-Esterase | PMR5N | PC-Esterase |
|  | PF02254 | g3062.t1 | Na_H_Exchanger | TrkA_N | Na_H_Exchanger |
|  | PF04828 | g3063.t1 | SelR | GFA | SelR |
|  | PF00069 | g3064.t1 | NA | Pkinase | Pkinase |
|  | NA | g3065.t1 | DUF761 | NA | DUF761 |
|  | NA | g3066.t1 | NA | NA | g3066.t1 |
|  | PF14204 | g3067.t1 | Ribosomal_L5e | Ribosomal_L18_c | Ribosomal_L5e |
|  | NA | g3068.t1 | Polyketide_cyc2 | NA | Polyketide_cyc2 |
|  | PF02373 | g3069.t1 | NA | JmjC | JmjC |
|  | NA | g3070.t1 | DREPP | NA | DREPP |
|  | PF08069 | g3071.t1 | AA_kinase | Ribosomal_S13_N | AA_kinase |
|  | PF08541 | g3072.t1 | FAE1_CUT1_RppA | ACP_syn_III_C | FAE1_CUT1_RppA |
|  | PF01357 | g15808.t1 | NA | Pollen_allerg_1 | Pollen_allerg_1 |
|  | PF02182 | g15809.t1 | SET | SAD_SRA | SET |
|  | PF00067 | g15810.t1 | NA | p450 | p450 |
|  | PF00320 | g15811.t1 | NA | GATA | GATA |
|  | NA | g15812.t1 | DUF4539 | NA | DUF4539 |
|  | PF13476 | g15813.t1 | Bax1-I | AAA_23 | Bax1-I |
|  | PF00635 | g15814.t1 | DUF3774 | Motile_Sperm | DUF3774 |
|  | PF00069 | g15815.t1 | APH | Pkinase | APH |
|  | NA | g15816.t1 | Ribosomal_L19 | NA | Ribosomal_L19 |
|  | PF01156 | g15817.t1 | MFS_1 | IU_nuc_hydro | MFS_1 |
|  | NA | g15818.t1 | Ins134_P3_kin | NA | Ins134_P3_kin |
|  | PF08784 | g15819.t1 | Transferase | RPA_C | Transferase |
|  | PF13450 | g15820.t1 | FAD_binding_3 | NAD_binding_8 | FAD_binding_3 |
|  | NA | g15821.t1 | NA | NA | g15821.t1 |
|  | PF00071 | g15822.t1 | MMR_HSR1 | Ras | MMR_HSR1 |
|  | PF17177 | g15823.t1 | PPR | PPR_long | PPR |
|  | NA | g15824.t1 | Tau95 | NA | Tau95 |
|  | PF13847 | g15825.t1 | HABP4_PAI-RBP1 | Methyltransf_31 | HABP4_PAI-RBP1 |
|  | PF12697 | g15826.t1 | Lipase_3 | Abhydrolase_6 | Lipase_3 |
|  | PF03171 | g15827.t1 | DIOX_N | 2OG-FeII_Oxy | DIOX_N |
|  | PF04146 | g15828.t1 | VEFS-Box | YTH | VEFS-Box |
|  | PF00628 | g15829.t1 | Jas | PHD | Jas |
|  | PF00295 | g15830.t1 | Pectate_lyase_3 | Glyco_hydro_28 | Pectate_lyase_3 |
|  | PF00378 | g15831.t1 | NA | ECH_1 | ECH_1 |
|  | PF00069 | g15832.t1 | B3 | Pkinase | B3 |
|  | PF00155 | g15833.t1 | UPF0113 | Aminotran_1_2 | UPF0113 |
|  | PF01088 | g15834.t1 | LRRNT_2 | Peptidase_C12 | LRRNT_2 |
|  | PF00032 | g2044.t1 | Pex24p | Cytochrom_B_C | Pex24p |
|  | PF01474 | g2045.t1 | Pyr_redox_3 | DAHP_synth_2 | Pyr_redox_3 |
|  | PF02732 | g2046.t1 | BPS1 | ERCC4 | BPS1 |
|  | PF00160 | g2047.t1 | NA | Pro_isomerase | Pro_isomerase |
|  | PF14547 | g2048.t1 | LTP_2 | Hydrophob_seed | LTP_2 |
|  | PF03501 | g2049.t1 | LTP_2 | S10_plectin | LTP_2 |
|  | PF14547 | g2050.t1 | LTP_2 | Hydrophob_seed | LTP_2 |
|  | PF14547 | g2051.t1 | LTP_2 | Hydrophob_seed | LTP_2 |
|  | NA | g2052.t1 | LTP_2 | NA | LTP_2 |
|  | PF14547 | g2053.t1 | LTP_2 | Hydrophob_seed | LTP_2 |
|  | PF00364 | g2054.t1 | LTP_2 | Biotin_lipoyl | LTP_2 |
|  | PF14547 | g2055.t1 | LTP_2 | Hydrophob_seed | LTP_2 |
|  | PF14547 | g2056.t1 | LTP_2 | Hydrophob_seed | LTP_2 |
|  | PF09325 | g2057.t1 | FH2 | Vps5 | FH2 |
|  | PF14547 | g2058.t1 | LTP_2 | Hydrophob_seed | LTP_2 |
|  | PF00069 | g2059.t1 | Choline_kinase | Pkinase | Choline_kinase |
|  | PF00036 | g2060.t1 | EF-hand_4 | EF-hand_1 | EF-hand_4 |
|  | NA | g2061.t1 | NA | NA | g2061.t1 |
|  | PF17177 | g2062.t1 | PPR | PPR_long | PPR |
|  | PF00067 | g2063.t1 | Polyketide_cyc2 | p450 | Polyketide_cyc2 |
|  | PF14416 | g2064.t1 | PPR | PMR5N | PPR |
|  | NA | g2065.t1 | ATG27 | NA | ATG27 |
|  | PF00295 | g2066.t1 | Pectate_lyase_3 | Glyco_hydro_28 | Pectate_lyase_3 |
|  | NA | g2067.t1 | Glyco_hydro_85 | NA | Glyco_hydro_85 |
|  | NA | g2068.t1 | LOB | NA | LOB |
|  | PF00141 | g2069.t1 | NA | peroxidase | peroxidase |
|  | PF00190 | g2070.t1 | NA | Cupin_1 | Cupin_1 |
|  | PF00190 | g2071.t1 | PCO_ADO | Cupin_1 | PCO_ADO |
|  | PF00190 | g2072.t1 | NA | Cupin_1 | Cupin_1 |
|  | PF00190 | g2073.t1 | NA | Cupin_1 | Cupin_1 |
|  | NA | g2074.t1 | Senescence_reg | NA | Senescence_reg |
|  | NA | g2075.t1 | Pollen_Ole_e_I | NA | Pollen_Ole_e_I |
|  | NA | g2076.t1 | DUF4005 | NA | DUF4005 |
|  | PF00226 | g2077.t1 | Pam16 | DnaJ | Pam16 |
|  | PF00036 | g2078.t1 | Mito_carr | EF-hand_1 | Mito_carr |
|  | NA | g2079.t1 | Mt_ATP-synt_D | NA | Mt_ATP-synt_D |
|  | NA | g2080.t1 | NA | NA | g2080.t1 |
|  | PF01191 | g2081.t1 | NA | RNA_pol_Rpb5_C | RNA_pol_Rpb5_C |
|  | NA | g2082.t1 | NA | NA | g2082.t1 |
|  | NA | g2083.t1 | NA | NA | g2083.t1 |
|  | NA | g2084.t1 | Ribosomal_60s | NA | Ribosomal_60s |
|  | PF01423 | g2085.t1 | NA | LSM | LSM |
|  | PF09759 | g2086.t1 | HEAT_2 | Atx10homo_assoc | HEAT_2 |
|  | NA | g2087.t1 | Mito_carr | NA | Mito_carr |
|  | NA | g2088.t1 | NA | NA | g2088.t1 |
|  | NA | g2089.t1 | NA | NA | g2089.t1 |
|  | NA | g2090.t1 | NA | NA | g2090.t1 |
|  | NA | g2091.t1 | RAI1 | NA | RAI1 |
|  | PF00010 | g2092.t1 | bHLH-MYC_N | HLH | bHLH-MYC_N |
|  | PF01214 | g2093.t1 | FBA_1 | CK_II_beta | FBA_1 |
|  | NA | g2094.t1 | NA | NA | g2094.t1 |
|  | NA | g2095.t1 | NA | NA | g2095.t1 |
|  | PF06220 | g2096.t1 | Ribosomal_L37ae | zf-U1 | Ribosomal_L37ae |
|  | PF13905 | g2097.t1 | SNF2_N | Thioredoxin_8 | SNF2_N |
|  | PF12352 | g2098.t1 | Sec20 | V-SNARE_C | Sec20 |
|  | PF00069 | g2099.t1 | APH | Pkinase | APH |
|  | PF01373 | g2100.t1 | NA | Glyco_hydro_14 | Glyco_hydro_14 |
|  | PF04043 | g2101.t1 | XendoU | PMEI | XendoU |
|  | PF00169 | g2102.t1 | VPS13 | PH | VPS13 |
|  | PF00412 | g2103.t1 | zf-FLZ | LIM | zf-FLZ |
|  | PF14559 | g2104.t1 | IATP | TPR_19 | IATP |
|  | PF13894 | g2105.t1 | Peptidase_C13 | zf-C2H2_4 | Peptidase_C13 |
|  | PF13920 | g2106.t1 | Prok-RING_4 | zf-C3HC4_3 | Prok-RING_4 |
|  | NA | g2107.t1 | NA | NA | g2107.t1 |
|  | PF05362 | g2108.t1 | LON_substr_bdg | Lon_C | LON_substr_bdg |
|  | PF03171 | g2109.t1 | DIOX_N | 2OG-FeII_Oxy | DIOX_N |
|  | PF03171 | g2110.t1 | DIOX_N | 2OG-FeII_Oxy | DIOX_N |
|  | NA | g15630.t1 | NA | NA | g15630.t1 |
|  | PF00069 | g15631.t1 | APH | Pkinase | APH |
|  | PF08996 | g15632.t1 | Memo | zf-DNA_Pol | Memo |
|  | PF08513 | g15633.t1 | Nup160 | LisH | Nup160 |
|  | PF10607 | g15634.t1 | PI-PLC-X | CLTH | PI-PLC-X |
|  | PF08513 | g15635.t1 | NA | LisH | LisH |
|  | NA | g15636.t1 | NA | NA | g15636.t1 |
|  | PF03168 | g15637.t1 | NA | LEA_2 | LEA_2 |
|  | PF00069 | g15638.t1 | NA | Pkinase | Pkinase |
|  | NA | g15639.t1 | Retrotran_gag_3 | NA | Retrotran_gag_3 |
|  | PF03662 | g15640.t1 | RALF | Glyco_hydro_79n | RALF |
|  | PF09190 | g15641.t1 | tRNA-synt_1e | NA | tRNA-synt_1e |
|  | PF00010 | g15642.t1 | Galactosyl_T | HLH | Galactosyl_T |
|  | PF13620 | g15643.t1 | DUF2012 | CarboxypepD_reg | DUF2012 |
|  | PF12697 | g15644.t1 | EamA | Abhydrolase_6 | EamA |
|  | PF16656 | g15645.t1 | Lipase_3 | Pur_ac_phosph_N | Lipase_3 |
|  | PF00076 | g15646.t1 | FH2 | RRM_1 | FH2 |
|  | PF02826 | g15647.t1 | ADH_zinc_N | 2-Hacid_dh_C | ADH_zinc_N |
|  | PF07992 | g15648.t1 | FAD_binding_3 | Pyr_redox_2 | FAD_binding_3 |
|  | PF01287 | g15649.t1 | NA | eIF-5a | eIF-5a |
|  | PF09346 | g15650.t1 | NAM | NA | NAM |
|  | NA | g15651.t1 | NA | NA | g15651.t1 |
|  | PF00562 | g15652.t1 | RNA_pol_Rpb2_1 | RNA_pol_Rpb2_6 | RNA_pol_Rpb2_1 |
|  | NA | g15653.t1 | Nuf2 | NA | Nuf2 |
|  | PF03106 | g15654.t1 | DUF1685 | WRKY | DUF1685 |
|  | PF16113 | g15655.t1 | Nucleotid_trans | ECH_2 | Nucleotid_trans |
|  | PF13091 | g15656.t1 | PLDc | PLDc_2 | PLDc |
|  | PF00651 | g15657.t1 | NPH3 | BTB | NPH3 |
|  | NA | g15658.t1 | PSK | NA | PSK |
|  | NA | g15659.t1 | EMP70 | NA | EMP70 |
|  | NA | g15660.t1 | RRM_DME | NA | RRM_DME |
|  | PF00173 | g15661.t1 | NA | Cyt-b5 | Cyt-b5 |
|  | PF02298 | g15662.t1 | DUF4228 | Cu_bind_like | DUF4228 |
|  | PF00651 | g15663.t1 | Sas10_Utp3 | BTB | Sas10_Utp3 |
|  | PF17177 | g15664.t1 | PPR | PPR_long | PPR |
|  | NA | g15665.t1 | Suf | NA | Suf |
|  | PF00350 | g15666.t1 | Dynamin_M | Dynamin_N | Dynamin_M |
|  | PF00702 | g15667.t1 | HAD_2 | Hydrolase | HAD_2 |
|  | PF00702 | g15668.t1 | HAD_2 | Hydrolase | HAD_2 |
|  | PF00071 | g15669.t1 | NA | Ras | Ras |
|  | PF17177 | g15670.t1 | PPR | PPR_long | PPR |
|  | PF08513 | g15671.t1 | Bud13 | LisH | Bud13 |
|  | PF00076 | g15672.t1 | G-patch | RRM_1 | G-patch |
|  | PF03479 | g15673.t1 | K_trans | DUF296 | K_trans |
|  | NA | g15674.t1 | NA | NA | g15674.t1 |
|  | PF03479 | g15675.t1 | NA | DUF296 | DUF296 |
|  | PF01217 | g15676.t1 | Adap_comp_sub | Clat_adaptor_s | Adap_comp_sub |
|  | PF06419 | g15677.t1 | NA | COG6 | COG6 |
|  | PF07714 | g15678.t1 | NA | Pkinase_Tyr | Pkinase_Tyr |
|  | PF02298 | g15679.t1 | LEA_1 | Cu_bind_like | LEA_1 |
|  | PF00111 | g15680.t1 | NA | Fer2 | Fer2 |
|  | PF06943 | g15681.t1 | NA | zf-LSD1 | zf-LSD1 |
|  | NA | g15682.t1 | KHA | NA | KHA |
|  | PF13920 | g15683.t1 | Prok-RING_4 | zf-C3HC4_3 | Prok-RING_4 |
|  | NA | g15684.t1 | NA | NA | g15684.t1 |
|  | PF01740 | g15685.t1 | Sulfate_transp | STAS | Sulfate_transp |
|  | NA | g15686.t1 | NA | NA | g15686.t1 |
|  | PF00646 | g15687.t1 | UCH | F-box | UCH |
|  | NA | g15688.t1 | ALMT | NA | ALMT |
|  | PF07534 | g15689.t1 | NA | TLD | TLD |
|  | NA | g15690.t1 | NAM | NA | NAM |
|  | PF05617 | g15691.t1 | UPF0051 | Prolamin_like | UPF0051 |
|  | PF17177 | g15692.t1 | PPR | PPR_long | PPR |
|  | NA | g15693.t1 | PP2C | NA | PP2C |
|  | PF00240 | g15694.t1 | Tryp_alpha_amyl | ubiquitin | Tryp_alpha_amyl |
|  | PF01597 | g15695.t1 | NA | GCV_H | GCV_H |
|  | PF07714 | g15696.t1 | DIOX_N | Pkinase_Tyr | DIOX_N |
|  | PF03168 | g15697.t1 | Glyco_transf_41 | LEA_2 | Glyco_transf_41 |
|  | PF00005 | g15698.t1 | PTR2 | ABC_tran | PTR2 |
|  | PF02887 | g15699.t1 | PK | PK_C | PK |
|  | NA | g15700.t1 | NA | NA | g15700.t1 |
|  | NA | g15701.t1 | NA | NA | g15701.t1 |
|  | NA | g15702.t1 | TMEM51 | NA | TMEM51 |
|  | PF03110 | g15703.t1 | TMEM51 | SBP | TMEM51 |
|  | NA | g15704.t1 | TMEM51 | NA | TMEM51 |
|  | NA | g15705.t1 | NA | NA | g15705.t1 |
|  | PF13912 | g15706.t1 | zf-C2H2_jaz | zf-C2H2_6 | zf-C2H2_jaz |
|  | PF07687 | g15707.t1 | Peptidase_M20 | M20_dimer | Peptidase_M20 |
|  | NA | g15708.t1 | Abi | NA | Abi |
|  | PF03725 | g15709.t1 | Bax1-I | RNase_PH_C | Bax1-I |
|  | PF00628 | g15710.t1 | Chlorophyllase | PHD | Chlorophyllase |
|  | NA | g15711.t1 | EPF | NA | EPF |
|  | PF06200 | g15712.t1 | Mito_carr | tify | Mito_carr |
|  | PF03079 | g15713.t1 | DUF3456 | ARD | DUF3456 |
|  | PF00407 | g15714.t1 | Polyketide_cyc2 | Bet_v_1 | Polyketide_cyc2 |
|  | PF09337 | g15715.t1 | NA | NA | g15715.t1 |
|  | NA | g15716.t1 | Dymeclin | NA | Dymeclin |
|  | NA | g15717.t1 | Chloroa_b-bind | NA | Chloroa_b-bind |
|  | PF00226 | g15718.t1 | NA | DnaJ | DnaJ |
|  | PF00010 | g15719.t1 | bHLH-MYC_N | HLH | bHLH-MYC_N |
|  | NA | g15720.t1 | GHMP_kinases_C | NA | GHMP_kinases_C |
|  | PF01412 | g15721.t1 | PPR | ArfGap | PPR |
|  | PF01105 | g15722.t1 | UCH | EMP24_GP25L | UCH |
|  | PF00582 | g15723.t1 | NA | Usp | Usp |
|  | PF08423 | g15724.t1 | RecA | Rad51 | RecA |
|  | NA | g15725.t1 | NA | NA | g15725.t1 |
|  | PF01423 | g15726.t1 | NDUFA12 | LSM | NDUFA12 |
|  | NA | g15727.t1 | F-actin_cap_A | NA | F-actin_cap_A |
|  | PF06839 | g15728.t1 | Metallothio_PEC | zf-GRF | Metallothio_PEC |
|  | PF00643 | g15729.t1 | DUF740 | zf-B_box | DUF740 |
|  | NA | g15730.t1 | PLATZ | NA | PLATZ |
|  | PF00487 | g15731.t1 | Tetraspannin | FA_desaturase | Tetraspannin |
|  | PF00076 | g15732.t1 | Cpn60_TCP1 | RRM_1 | Cpn60_TCP1 |
|  | PF13639 | g15733.t1 | zf-ANAPC11 | zf-RING_2 | zf-ANAPC11 |
|  | PF00010 | g15734.t1 | Lung_7-TM_R | HLH | Lung_7-TM_R |
|  | PF06747 | g15735.t1 | GWT1 | CHCH | GWT1 |
|  | NA | g15736.t1 | LETM1 | NA | LETM1 |
|  | PF01417 | g15737.t1 | NA | ENTH | ENTH |
|  | PF10431 | g15738.t1 | NA | ClpB_D2-small | ClpB_D2-small |
|  | PF13238 | g15739.t1 | EnY2 | AAA_18 | EnY2 |
|  | PF00004 | g15740.t1 | MIP | AAA | MIP |
|  | PF00004 | g15741.t1 | Rad17 | AAA | Rad17 |
|  | PF13639 | g15742.t1 | Prok-RING_4 | zf-RING_2 | Prok-RING_4 |
|  | PF16131 | g15743.t1 | zf-CCCH | Torus | zf-CCCH |
|  | PF13460 | g15744.t1 | 3Beta_HSD | NAD_binding_10 | 3Beta_HSD |
|  | PF08801 | g15745.t1 | Vma12 | Nucleoporin_N | Vma12 |
|  | NA | g15746.t1 | NA | NA | g15746.t1 |
|  | PF14559 | g15747.t1 | AUX_IAA | TPR_19 | AUX_IAA |
|  | PF00332 | g15748.t1 | NA | Glyco_hydro_17 | Glyco_hydro_17 |
|  | PF00076 | g15749.t1 | PPR | RRM_1 | PPR |
|  | PF00097 | g15750.t1 | SNARE_assoc | zf-C3HC4 | SNARE_assoc |
|  | NA | g15751.t1 | Ins134_P3_kin | NA | Ins134_P3_kin |
|  | PF00249 | g15752.t1 | NA | Myb_DNA-binding | Myb_DNA-binding |
|  | NA | g15753.t1 | NA | NA | g15753.t1 |
|  | NA | g15754.t1 | PTR2 | NA | PTR2 |
|  | NA | g15755.t1 | Isochorismatase | NA | Isochorismatase |
|  | PF03061 | g15756.t1 | NA | 4HBT | 4HBT |
|  | PF03061 | g15757.t1 | PHO4 | 4HBT | PHO4 |
|  | PF13193 | g15758.t1 | PHO4 | AMP-binding_C | PHO4 |
|  | NA | g15759.t1 | CitMHS | NA | CitMHS |
|  | PF03604 | g15760.t1 | CcmH | DNA_RNApol_7kD | CcmH |
|  | NA | g15761.t1 | PHO4 | NA | PHO4 |
|  | PF07714 | g15762.t1 | APH | Pkinase_Tyr | APH |
|  | PF00071 | g15763.t1 | PHO4 | Ras | PHO4 |
|  | PF00071 | g15764.t1 | PhoLip_ATPase_C | Ras | PhoLip_ATPase_C |
|  | NA | g15765.t1 | NA | NA | g15765.t1 |
|  | PF00134 | g3076.t1 | PP2C | Cyclin_N | PP2C |
|  | NA | g3077.t1 | zf-CCCH | NA | zf-CCCH |
|  | PF00808 | g3078.t1 | tRNA_synt_2f | CBFD_NFYB_HMF | tRNA_synt_2f |
|  | PF08241 | g3079.t1 | Methyltransf_29 | Methyltransf_11 | Methyltransf_29 |
|  | PF16190 | g3080.t1 | NA | E1_FCCH | E1_FCCH |
|  | NA | g3081.t1 | NA | NA | g3081.t1 |
|  | PF08320 | g3082.t1 | NA | PIG-X | PIG-X |
|  | PF14523 | g3083.t1 | SNARE | Syntaxin_2 | SNARE |
|  | PF05617 | g3084.t1 | ATP-synt_Z | Prolamin_like | ATP-synt_Z |
|  | PF00076 | g3085.t1 | Branch | RRM_1 | Branch |
|  | NA | g3086.t1 | NA | NA | g3086.t1 |
|  | NA | g3087.t1 | NA | NA | g3087.t1 |
|  | PF07992 | g3088.t1 | Pyr_redox_3 | Pyr_redox_2 | Pyr_redox_3 |
|  | PF02749 | g3089.t1 | MFS_5 | QRPTase_N | MFS_5 |
|  | NA | g3090.t1 | DUF632 | NA | DUF632 |
|  | PF17171 | g3091.t1 | Sulfate_transp | GST_C_6 | Sulfate_transp |
|  | PF03143 | g3092.t1 | NA | GTP_EFTU_D3 | GTP_EFTU_D3 |
|  | PF13460 | g3093.t1 | Epimerase | NAD_binding_10 | Epimerase |
|  | NA | g3094.t1 | Nop53 | NA | Nop53 |
|  | PF12174 | g3095.t1 | PARP | RST | PARP |
|  | PF12174 | g3096.t1 | PARP | RST | PARP |
|  | PF00249 | g3097.t1 | NA | Myb_DNA-binding | Myb_DNA-binding |
|  | PF02373 | g3098.t1 | JmjN | JmjC | JmjN |
|  | NA | g3099.t1 | NA | NA | g3099.t1 |
|  | PF00072 | g3100.t1 | UDPGT | Response_reg | UDPGT |
|  | PF00903 | g3101.t1 | Glyoxalase_4 | Glyoxalase | Glyoxalase_4 |
|  | NA | g3102.t1 | NA | NA | g3102.t1 |
|  | NA | g3103.t1 | NA | NA | g3103.t1 |
|  | PF08240 | g3104.t1 | ADH_zinc_N | ADH_N | ADH_zinc_N |
|  | NA | g15835.t1 | Oleosin | NA | Oleosin |
|  | PF01399 | g15836.t1 | DUF106 | PCI | DUF106 |
|  | NA | g15837.t1 | Tmemb_14 | NA | Tmemb_14 |
|  | PF01363 | g15838.t1 | NA | FYVE | FYVE |
|  | PF03126 | g15839.t1 | NA | Plus-3 | Plus-3 |
|  | PF00690 | g15840.t1 | E1-E2_ATPase | Cation_ATPase_N | E1-E2_ATPase |
|  | PF00293 | g15841.t1 | DUF4339 | NUDIX | DUF4339 |
|  | PF05903 | g15842.t1 | NA | Peptidase_C97 | Peptidase_C97 |
|  | PF00690 | g15843.t1 | E1-E2_ATPase | Cation_ATPase_N | E1-E2_ATPase |
|  | PF02089 | g15844.t1 | DUF676 | Palm_thioest | DUF676 |
|  | NA | g15845.t1 | DUF599 | NA | DUF599 |
|  | PF17177 | g15846.t1 | PPR | PPR_long | PPR |
|  | PF00782 | g15847.t1 | NA | DSPc | DSPc |
|  | PF08783 | g15848.t1 | NA | DWNN | DWNN |
|  | PF00651 | g15849.t1 | AIM24 | BTB | AIM24 |
|  | PF04998 | g15850.t1 | Exostosin | RNA_pol_Rpb1_5 | Exostosin |
|  | PF00249 | g15851.t1 | NA | Myb_DNA-binding | Myb_DNA-binding |
|  | NA | g15852.t1 | mit_SMPDase | NA | mit_SMPDase |
|  | NA | g15853.t1 | Cation_efflux | NA | Cation_efflux |
|  | PF00847 | g15854.t1 | NA | AP2 | AP2 |
|  | PF00067 | g15855.t1 | Cation_efflux | p450 | Cation_efflux |
|  | PF00067 | g15856.t1 | NA | p450 | p450 |
|  | PF14749 | g15857.t1 | ACOX | Acyl-CoA_ox_N | ACOX |
|  | PF13920 | g15858.t1 | ELYS | zf-C3HC4_3 | ELYS |
|  | PF03171 | g15859.t1 | DIOX_N | 2OG-FeII_Oxy | DIOX_N |
|  | PF00249 | g15860.t1 | NA | Myb_DNA-binding | Myb_DNA-binding |
|  | NA | g15861.t1 | O-FucT | NA | O-FucT |
|  | NA | g15862.t1 | DUF1639 | NA | DUF1639 |
|  | PF04618 | g15863.t1 | HALZ | HD-ZIP_N | HALZ |
|  | PF13847 | g15864.t1 | DUF761 | Methyltransf_31 | DUF761 |
|  | PF00072 | g15865.t1 | NA | Response_reg | Response_reg |
|  | PF00722 | g15866.t1 | NA | Glyco_hydro_16 | Glyco_hydro_16 |
|  | PF00694 | g15867.t1 | DXP_synthase_N | Aconitase_C | DXP_synthase_N |
|  | NA | g15868.t1 | Ribosomal_L15e | NA | Ribosomal_L15e |
|  | PF00488 | g15869.t1 | SecY | MutS_V | SecY |
|  | PF02894 | g15870.t1 | GFO_IDH_MocA | GFO_IDH_MocA_C | GFO_IDH_MocA |
|  | PF00561 | g15871.t1 | PS_Dcarbxylase | Abhydrolase_1 | PS_Dcarbxylase |
|  | PF00551 | g15872.t1 | NA | Formyl_trans_N | Formyl_trans_N |
|  | PF00169 | g15873.t1 | Auxin_canalis | PH | Auxin_canalis |
|  | NA | g15874.t1 | DUF707 | NA | DUF707 |
|  | PF16113 | g15875.t1 | NA | ECH_2 | ECH_2 |
|  | NA | g15876.t1 | MIP | NA | MIP |
|  | PF05641 | g15877.t1 | DUF547 | Agenet | DUF547 |
|  | NA | g15878.t1 | O-FucT | NA | O-FucT |
|  | PF00413 | g15879.t1 | NA | Peptidase_M10 | Peptidase_M10 |
|  | PF17177 | g15880.t1 | PPR | PPR_long | PPR |
|  | PF13912 | g15881.t1 | EamA | zf-C2H2_6 | EamA |
|  | PF12931 | g15882.t1 | Coatomer_WDAD | Sec16_C | Coatomer_WDAD |
|  | PF00152 | g15883.t1 | GRAS | tRNA-synt_2 | GRAS |
|  | NA | g15884.t1 | NA | NA | g15884.t1 |
|  | PF12874 | g15885.t1 | zf-C2H2_jaz | zf-met | zf-C2H2_jaz |
|  | PF00745 | g15886.t1 | Pex19 | GlutR_dimer | Pex19 |
|  | NA | g15887.t1 | Pectinesterase | NA | Pectinesterase |
|  | NA | g15888.t1 | Mannosyl_trans3 | NA | Mannosyl_trans3 |
|  | PF00069 | g15889.t1 | Haspin_kinase | Pkinase | Haspin_kinase |
|  | PF09454 | g29542.t1 | Nsp1_C | Vps23_core | Nsp1_C |
|  | NA | g29543.t1 | PP2C | NA | PP2C |
|  | NA | g29544.t1 | Cellulose_synt | NA | Cellulose_synt |
|  | PF07974 | g29545.t1 | Exostosin | EGF_2 | Exostosin |
|  | NA | g29546.t1 | FH2 | NA | FH2 |
|  | NA | g29547.t1 | SSF | NA | SSF |
|  | PF17177 | g29548.t1 | PPR | PPR_long | PPR |
|  | NA | g29549.t1 | NA | NA | g29549.t1 |
|  | NA | g29550.t1 | ABC_membrane | NA | ABC_membrane |
|  | PF16113 | g29551.t1 | SSF | ECH_2 | SSF |
|  | PF17177 | g29552.t1 | PPR | PPR_long | PPR |
|  | NA | g29553.t1 | AOX | NA | AOX |
|  | NA | g29554.t1 | Glyco_hydro_31 | NA | Glyco_hydro_31 |
|  | PF13802 | g29555.t1 | Glyco_hydro_31 | Gal_mutarotas_2 | Glyco_hydro_31 |
|  | PF13802 | g29556.t1 | Glyco_hydro_31 | Gal_mutarotas_2 | Glyco_hydro_31 |
|  | NA | g29557.t1 | NA | NA | g29557.t1 |
|  | PF00270 | g29558.t1 | Helicase_C | DEAD | Helicase_C |
|  | PF17177 | g29559.t1 | PPR | PPR_long | PPR |
|  | NA | g29560.t1 | DUF1668 | NA | DUF1668 |
|  | PF13637 | g29561.t1 | NA | Ank_4 | Ank_4 |
|  | NA | g29562.t1 | VPS13 | NA | VPS13 |
|  | NA | g29563.t1 | NA | NA | g29563.t1 |
|  | PF13837 | g29564.t1 | NA | Myb_DNA-bind_4 | Myb_DNA-bind_4 |
|  | PF12874 | g29565.t1 | zf-C2H2_jaz | zf-met | zf-C2H2_jaz |
|  | PF00069 | g29566.t1 | LRRNT_2 | Pkinase | LRRNT_2 |
|  | NA | g29567.t1 | SBP_bac_6 | NA | SBP_bac_6 |
|  | PF14604 | g29568.t1 | Nsp1_C | SH3_9 | Nsp1_C |
|  | PF14543 | g29569.t1 | Asp | TAXi_N | Asp |
|  | PF14543 | g29570.t1 | Asp | TAXi_N | Asp |
|  | PF14543 | g29571.t1 | Asp | TAXi_N | Asp |
|  | PF14543 | g29572.t1 | Asp | TAXi_N | Asp |
|  | PF14543 | g29573.t1 | Asp | TAXi_N | Asp |
|  | PF00063 | g29574.t1 | EPSP_synthase | Myosin_head | EPSP_synthase |
|  | PF00134 | g29575.t1 | Sulfate_transp | Cyclin_N | Sulfate_transp |
|  | NA | g29576.t1 | TraB | NA | TraB |
|  | NA | g29577.t1 | Auxin_canalis | NA | Auxin_canalis |
|  | NA | g29578.t1 | PORR | NA | PORR |
|  | PF07714 | g29579.t1 | NA | Pkinase_Tyr | Pkinase_Tyr |
|  | PF13439 | g29580.t1 | Glycos_transf_1 | Glyco_transf_4 | Glycos_transf_1 |
|  | NA | g29581.t1 | NA | NA | g29581.t1 |
|  | PF13439 | g29582.t1 | Nodulin-like | Glyco_transf_4 | Nodulin-like |
|  | NA | g29583.t1 | DUF3511 | NA | DUF3511 |
|  | NA | g29584.t1 | HAUS-augmin3 | NA | HAUS-augmin3 |
|  | NA | g29585.t1 | NA | NA | g29585.t1 |
|  | NA | g29586.t1 | NA | NA | g29586.t1 |
|  | PF17177 | g29587.t1 | PPR | PPR_long | PPR |
|  | NA | g29588.t1 | Transferase | NA | Transferase |
|  | NA | g29589.t1 | NA | NA | g29589.t1 |
|  | NA | g29590.t1 | NA | NA | g29590.t1 |
|  | NA | g29591.t1 | NA | NA | g29591.t1 |
|  | PF03372 | g29592.t1 | NA | Exo_endo_phos | Exo_endo_phos |
|  | NA | g29593.t1 | NA | NA | g29593.t1 |
|  | NA | g29594.t1 | NA | NA | g29594.t1 |
|  | NA | g29595.t1 | PAE | NA | PAE |
|  | PF00235 | g29596.t1 | NA | Profilin | Profilin |
|  | PF13639 | g29597.t1 | Prok-RING_4 | zf-RING_2 | Prok-RING_4 |
|  | PF01373 | g29598.t1 | BES1_N | Glyco_hydro_14 | BES1_N |
|  | PF13532 | g29599.t1 | PRONE | 2OG-FeII_Oxy_2 | PRONE |
|  | NA | g29600.t1 | NA | NA | g29600.t1 |
|  | NA | g29601.t1 | Transferase | NA | Transferase |
|  | NA | g29602.t1 | Transferase | NA | Transferase |
|  | NA | g29603.t1 | NA | NA | g29603.t1 |
|  | PF00106 | g20786.t1 | NA | adh_short | adh_short |
|  | PF06424 | g20787.t1 | TPR_16 | PRP1_N | TPR_16 |
|  | NA | g20788.t1 | Sugar_tr | NA | Sugar_tr |
|  | PF00561 | g20789.t1 | Hydrolase_4 | Abhydrolase_1 | Hydrolase_4 |
|  | PF00561 | g20790.t1 | Hydrolase_4 | Abhydrolase_1 | Hydrolase_4 |
|  | PF00503 | g20791.t1 | Hydrolase_4 | G-alpha | Hydrolase_4 |
|  | NA | g20792.t1 | LANC_like | NA | LANC_like |
|  | PF01357 | g20793.t1 | NA | Pollen_allerg_1 | Pollen_allerg_1 |
|  | PF01399 | g20794.t1 | Tetraspannin | PCI | Tetraspannin |
|  | PF13508 | g20795.t1 | Acetyltransf_1 | Acetyltransf_7 | Acetyltransf_1 |
|  | NA | g20796.t1 | APG6 | NA | APG6 |
|  | PF13962 | g20797.t1 | Vma12 | PGG | Vma12 |
|  | NA | g20798.t1 | NA | NA | g20798.t1 |
|  | PF00173 | g20799.t1 | NA | Cyt-b5 | Cyt-b5 |
|  | PF01556 | g20800.t1 | SCAMP | DnaJ_C | SCAMP |
|  | PF13947 | g20801.t1 | NA | GUB_WAK_bind | GUB_WAK_bind |
|  | PF00561 | g20802.t1 | Hydrolase_4 | Abhydrolase_1 | Hydrolase_4 |
|  | PF13460 | g34704.t1 | NmrA | NAD_binding_10 | NmrA |
|  | PF12678 | g27202.t1 | zf-ANAPC11 | zf-rbx1 | zf-ANAPC11 |
|  | PF01388 | g27203.t1 | ELM2 | ARID | ELM2 |
|  | PF00179 | g27204.t1 | NA | UQ_con | UQ_con |
|  | NA | g27205.t1 | SAP30_Sin3_bdg | NA | SAP30_Sin3_bdg |
|  | PF00571 | g27206.t1 | DUF21 | CBS | DUF21 |
|  | PF09066 | g27207.t1 | Adaptin_N | B2-adapt-app_C | Adaptin_N |
|  | PF16203 | g27208.t1 | ResIII | ERCC3_RAD25_C | ResIII |
|  | PF13639 | g27209.t1 | zf-ANAPC11 | zf-RING_2 | zf-ANAPC11 |
|  | PF00439 | g27210.t1 | NA | Bromodomain | Bromodomain |
|  | PF13639 | g27211.t1 | Prok-RING_4 | zf-RING_2 | Prok-RING_4 |
|  | NA | g27212.t1 | Bap31 | NA | Bap31 |
|  | PF00651 | g27213.t1 | PseudoU_synth_2 | BTB | PseudoU_synth_2 |
|  | PF00249 | g27214.t1 | NA | Myb_DNA-binding | Myb_DNA-binding |
|  | PF00300 | g27215.t1 | His_Phos_2 | His_Phos_1 | His_Phos_2 |
|  | PF07526 | g27216.t1 | Homeobox_KN | POX | Homeobox_KN |
|  | PF13912 | g27217.t1 | DUF604 | zf-C2H2_6 | DUF604 |
|  | PF17177 | g27218.t1 | PPR | PPR_long | PPR |
|  | PF12680 | g27219.t1 | zf-C2H2_jaz | SnoaL_2 | zf-C2H2_jaz |
|  | PF13912 | g27220.t1 | zf-C2H2_jaz | zf-C2H2_6 | zf-C2H2_jaz |
|  | NA | g27221.t1 | NTP_transf_2 | NA | NTP_transf_2 |
|  | NA | g27222.t1 | FA_hydroxylase | NA | FA_hydroxylase |
|  | NA | g27223.t1 | NA | NA | g27223.t1 |
|  | PF00295 | g27224.t1 | Pectate_lyase_3 | Glyco_hydro_28 | Pectate_lyase_3 |
|  | PF00069 | g27225.t1 | Haspin_kinase | Pkinase | Haspin_kinase |
|  | PF10551 | g27226.t1 | DBD_Tnp_Mut | MULE | DBD_Tnp_Mut |
|  | NA | g27227.t1 | NA | NA | g27227.t1 |
|  | PF13857 | g27228.t1 | Sacchrp_dh_NADP | Ank_5 | Sacchrp_dh_NADP |
|  | PF13961 | g27229.t1 | UDPGT | DUF4219 | UDPGT |
|  | PF03405 | g27230.t1 | Retrotran_gag_2 | FA_desaturase_2 | Retrotran_gag_2 |
|  | PF00069 | g27231.t1 | Haspin_kinase | Pkinase | Haspin_kinase |
|  | PF13637 | g27232.t1 | NA | Ank_4 | Ank_4 |
|  | PF13857 | g27233.t1 | Retrotran_gag_2 | Ank_5 | Retrotran_gag_2 |
|  | NA | g27234.t1 | NA | NA | g27234.t1 |
|  | PF00378 | g27235.t1 | G-patch | ECH_1 | G-patch |
|  | NA | g27236.t1 | NA | NA | g27236.t1 |
|  | PF03107 | g27237.t1 | Cation_efflux | C1_2 | Cation_efflux |
|  | PF03107 | g27238.t1 | LRRNT_2 | C1_2 | LRRNT_2 |
|  | PF17177 | g27239.t1 | PPR | PPR_long | PPR |
|  | PF08541 | g27240.t1 | Ubiq_cyt_C_chap | ACP_syn_III_C | Ubiq_cyt_C_chap |
|  | NA | g27241.t1 | NA | NA | g27241.t1 |
|  | PF13621 | g18698.t1 | Ribosomal_L21p | Cupin_8 | Ribosomal_L21p |
|  | PF17177 | g18699.t1 | PPR | PPR_long | PPR |
|  | NA | g18700.t1 | RPE65 | NA | RPE65 |
|  | PF00098 | g18701.t1 | RPE65 | zf-CCHC | RPE65 |
|  | PF00628 | g18702.t1 | ELM2 | PHD | ELM2 |
|  | PF00628 | g18703.t1 | Homeobox_KN | PHD | Homeobox_KN |
|  | NA | g18704.t1 | NA | NA | g18704.t1 |
|  | PF07651 | g18705.t1 | EamA | ANTH | EamA |
|  | NA | g18706.t1 | Ribosomal_L4 | NA | Ribosomal_L4 |
|  | NA | g18707.t1 | NA | NA | g18707.t1 |
|  | PF02201 | g18708.t1 | ABC2_membrane | SWIB | ABC2_membrane |
|  | PF12776 | g18709.t1 | DUF573 | Myb_DNA-bind_3 | DUF573 |
|  | NA | g18710.t1 | Transglut_core2 | NA | Transglut_core2 |
|  | NA | g18711.t1 | DAGK_cat | NA | DAGK_cat |
|  | PF12937 | g18712.t1 | Herpes_UL92 | F-box-like | Herpes_UL92 |
|  | PF00005 | g18713.t1 | Fer4_9 | ABC_tran | Fer4_9 |
|  | PF14559 | g18714.t1 | PPR | TPR_19 | PPR |
|  | NA | g18715.t1 | DUF716 | NA | DUF716 |
|  | PF00076 | g18716.t1 | Nucleotid_trans | RRM_1 | Nucleotid_trans |
|  | PF13961 | g18717.t1 | Retrotran_gag_3 | DUF4219 | Retrotran_gag_3 |
|  | PF00069 | g18718.t1 | K_trans | Pkinase | K_trans |
|  | PF00067 | g18719.t1 | NA | p450 | p450 |
|  | NA | g18720.t1 | Nnf1 | NA | Nnf1 |
|  | PF08502 | g18721.t1 | Adaptin_N | LeuA_dimer | Adaptin_N |
|  | NA | g18722.t1 | NA | NA | g18722.t1 |
|  | PF12701 | g18723.t1 | zf-ANAPC11 | LSM14 | zf-ANAPC11 |
|  | NA | g18724.t1 | NA | NA | g18724.t1 |
|  | NA | g18725.t1 | DUF1218 | NA | DUF1218 |
|  | PF00732 | g18726.t1 | FAD_binding_2 | GMC_oxred_N | FAD_binding_2 |
|  | PF00314 | g18727.t1 | UPF0114 | Thaumatin | UPF0114 |
|  | PF00314 | g18728.t1 | NA | Thaumatin | Thaumatin |
|  | PF17177 | g18729.t1 | PPR | PPR_long | PPR |
|  | PF00175 | g18730.t1 | NA | NAD_binding_1 | NAD_binding_1 |
|  | PF00226 | g18731.t1 | NA | DnaJ | DnaJ |
|  | NA | g18732.t1 | NA | NA | g18732.t1 |
|  | PF00327 | g18733.t1 | NA | Ribosomal_L30 | Ribosomal_L30 |
|  | PF13191 | g18734.t1 | PTR2 | AAA_16 | PTR2 |
|  | PF01180 | g18735.t1 | bZIP_1 | DHO_dh | bZIP_1 |
|  | NA | g18736.t1 | K_trans | NA | K_trans |
|  | PF01424 | g18737.t1 | zf-NF-X1 | R3H | zf-NF-X1 |
|  | PF07779 | g18738.t1 | NA | Cas1_AcylT | Cas1_AcylT |
|  | PF00249 | g18739.t1 | Spatacsin_C | Myb_DNA-binding | Spatacsin_C |
|  | NA | g18740.t1 | NA | NA | g18740.t1 |
|  | PF05191 | g18741.t1 | K_trans | ADK_lid | K_trans |
|  | PF02910 | g18742.t1 | PI3Ka | Succ_DH_flav_C | PI3Ka |
|  | NA | g18743.t1 | Transferase | NA | Transferase |
|  | NA | g18744.t1 | NIF | NA | NIF |
|  | PF00249 | g21159.t1 | bHLH-MYC_N | Myb_DNA-binding | bHLH-MYC_N |
|  | PF13180 | g21160.t1 | ALMT | PDZ_2 | ALMT |
|  | PF04438 | g21161.t1 | SHQ1 | zf-HIT | SHQ1 |
|  | PF00789 | g21162.t1 | CDC45 | UBX | CDC45 |
|  | PF00319 | g21163.t1 | K-box | SRF-TF | K-box |
|  | PF00069 | g21164.t1 | APH | Pkinase | APH |
|  | PF03226 | g21165.t1 | RGP | Yippee-Mis18 | RGP |
|  | PF00067 | g21166.t1 | OPT | p450 | OPT |
|  | PF01661 | g21167.t1 | MT-A70 | Macro | MT-A70 |
|  | PF02298 | g21168.t1 | PI-PLC-X | Cu_bind_like | PI-PLC-X |
|  | NA | g21169.t1 | MFS_5 | NA | MFS_5 |
|  | PF04434 | g21170.t1 | HpcH_HpaI | SWIM | HpcH_HpaI |
|  | NA | g21171.t1 | UPF0160 | NA | UPF0160 |
|  | PF09090 | g21172.t1 | VIT1 | MIF4G_like_2 | VIT1 |
|  | PF16845 | g21173.t1 | DUF659 | SQAPI | DUF659 |
|  | NA | g21174.t1 | DUF4666 | NA | DUF4666 |
|  | PF00069 | g21175.t1 | Haspin_kinase | Pkinase | Haspin_kinase |
|  | PF00010 | g21176.t1 | NA | HLH | HLH |
|  | PF06220 | g21177.t1 | OPT | zf-U1 | OPT |
|  | NA | g21178.t1 | OPT | NA | OPT |
|  | PF00450 | g21179.t1 | OPT | Peptidase_S10 | OPT |
|  | PF00450 | g21180.t1 | TOM20_plant | Peptidase_S10 | TOM20_plant |
|  | PF00076 | g21181.t1 | RabGAP-TBC | RRM_1 | RabGAP-TBC |
|  | PF00685 | g21182.t1 | DUF538 | Sulfotransfer_1 | DUF538 |
|  | PF00561 | g21183.t1 | Hydrolase_4 | Abhydrolase_1 | Hydrolase_4 |
|  | PF01053 | g21184.t1 | MFS_MOT1 | Cys_Met_Meta_PP | MFS_MOT1 |
|  | PF00067 | g21185.t1 | NA | p450 | p450 |
|  | PF13638 | g21186.t1 | Med10 | PIN_4 | Med10 |
|  | PF00046 | g21187.t1 | HALZ | Homeobox | HALZ |
|  | NA | g21188.t1 | NA | NA | g21188.t1 |
|  | PF09118 | g21189.t1 | Glyoxal_oxid_N | DUF1929 | Glyoxal_oxid_N |
|  | PF00010 | g21190.t1 | bHLH-MYC_N | HLH | bHLH-MYC_N |
|  | PF00561 | g21191.t1 | Hydrolase_4 | Abhydrolase_1 | Hydrolase_4 |
|  | NA | g21192.t1 | Lipase_GDSL | NA | Lipase_GDSL |
|  | NA | g21193.t1 | DUF4033 | NA | DUF4033 |
|  | PF00759 | g21194.t1 | NA | Glyco_hydro_9 | Glyco_hydro_9 |
|  | PF14559 | g21195.t1 | Lung_7-TM_R | TPR_19 | Lung_7-TM_R |
|  | PF08144 | g21196.t1 | DUF641 | CPL | DUF641 |
|  | PF08879 | g21197.t1 | Cpn60_TCP1 | WRC | Cpn60_TCP1 |
|  | PF03106 | g21198.t1 | DUF4005 | WRKY | DUF4005 |
|  | PF00462 | g21199.t1 | NA | Glutaredoxin | Glutaredoxin |
|  | PF00082 | g21200.t1 | NAM | Peptidase_S8 | NAM |
|  | PF04146 | g21201.t1 | DUF4228 | YTH | DUF4228 |
|  | PF02779 | g21202.t1 | DUF4228 | Transket_pyr | DUF4228 |
|  | PF08240 | g21203.t1 | ADH_zinc_N | ADH_N | ADH_zinc_N |
|  | PF00076 | g21204.t1 | eIF3g | RRM_1 | eIF3g |
|  | NA | g21205.t1 | PTR2 | NA | PTR2 |
|  | PF00628 | g21206.t1 | PHD_2 | PHD | PHD_2 |
|  | PF06337 | g21207.t1 | UCH | DUSP | UCH |
|  | PF00332 | g21208.t1 | PTR2 | Glyco_hydro_17 | PTR2 |
|  | NA | g21209.t1 | Ribosomal_60s | NA | Ribosomal_60s |
|  | PF08323 | g21210.t1 | Glycos_transf_1 | Glyco_transf_5 | Glycos_transf_1 |
|  | NA | g21211.t1 | MIF4G | NA | MIF4G |
|  | NA | g21212.t1 | Ctr | NA | Ctr |
|  | NA | g21213.t1 | NA | NA | g21213.t1 |
|  | PF13460 | g21214.t1 | E1_dh | NAD_binding_10 | E1_dh |
|  | PF01852 | g21215.t1 | NA | START | START |
|  | PF00076 | g21216.t1 | Galactosyl_T | RRM_1 | Galactosyl_T |
|  | PF13857 | g21217.t1 | PH_2 | Ank_5 | PH_2 |
|  | PF00082 | g21218.t1 | PA | Peptidase_S8 | PA |
|  | PF05922 | g21219.t1 | Reticulon | Inhibitor_I9 | Reticulon |
|  | PF00082 | g21220.t1 | PA | Peptidase_S8 | PA |
|  | PF00082 | g21221.t1 | NA | Peptidase_S8 | Peptidase_S8 |
|  | PF13639 | g21222.t1 | zf-ANAPC11 | zf-RING_2 | zf-ANAPC11 |
|  | PF03946 | g21223.t1 | Cation_efflux | Ribosomal_L11_N | Cation_efflux |
|  | PF08100 | g21224.t1 | Prok-RING_4 | Dimerisation | Prok-RING_4 |
|  | PF08100 | g21225.t1 | Methyltransf_2 | Dimerisation | Methyltransf_2 |
|  | NA | g21226.t1 | Methyltransf_2 | NA | Methyltransf_2 |
|  | PF08100 | g21227.t1 | E1_dh | Dimerisation | E1_dh |
|  | PF03171 | g21228.t1 | DIOX_N | 2OG-FeII_Oxy | DIOX_N |
|  | PF03171 | g21229.t1 | DIOX_N | 2OG-FeII_Oxy | DIOX_N |
|  | PF00719 | g21230.t1 | JmjN | Pyrophosphatase | JmjN |
|  | PF00646 | g21231.t1 | Acetyltransf_1 | F-box | Acetyltransf_1 |
|  | PF02953 | g21232.t1 | NA | zf-Tim10_DDP | zf-Tim10_DDP |
|  | PF00076 | g21233.t1 | B3 | RRM_1 | B3 |
|  | PF00403 | g21234.t1 | NA | HMA | HMA |
|  | NA | g21235.t1 | NA | NA | g21235.t1 |
|  | NA | g21236.t1 | NAM | NA | NAM |
|  | PF12906 | g21237.t1 | NA | RINGv | RINGv |
|  | PF12755 | g21238.t1 | DUF4042 | Vac14_Fab1_bd | DUF4042 |
|  | PF17123 | g21239.t1 | zf-ANAPC11 | zf-RING_11 | zf-ANAPC11 |
|  | PF08240 | g21240.t1 | ADH_zinc_N | ADH_N | ADH_zinc_N |
|  | NA | g21241.t1 | EamA | NA | EamA |
|  | PF03101 | g21242.t1 | Nucleoporin2 | FAR1 | Nucleoporin2 |
|  | NA | g21243.t1 | TPX2 | NA | TPX2 |
|  | NA | g21244.t1 | NA | NA | g21244.t1 |
|  | NA | g21245.t1 | EamA | NA | EamA |
|  | NA | g21246.t1 | NA | NA | g21246.t1 |
|  | PF13649 | g21247.t1 | Methyltransf_29 | Methyltransf_25 | Methyltransf_29 |
|  | NA | g21248.t1 | TMPIT | NA | TMPIT |
|  | PF13837 | g21249.t1 | Haspin_kinase | Myb_DNA-bind_4 | Haspin_kinase |
|  | PF00327 | g21250.t1 | NA | Ribosomal_L30 | Ribosomal_L30 |
|  | NA | g21251.t1 | DUF604 | NA | DUF604 |
|  | PF00069 | g21252.t1 | APH | Pkinase | APH |
|  | PF14569 | g21253.t1 | Tmemb_14 | zf-UDP | Tmemb_14 |
|  | NA | g21254.t1 | NA | NA | g21254.t1 |
|  | PF11940 | g21255.t1 | DUF3458_C | DUF3458 | DUF3458_C |
|  | PF11940 | g21256.t1 | DUF3458_C | DUF3458 | DUF3458_C |
|  | NA | g21257.t1 | BCAS3 | NA | BCAS3 |
|  | PF02214 | g21258.t1 | Hpt | BTB_2 | Hpt |
|  | PF01156 | g21259.t1 | CitMHS | IU_nuc_hydro | CitMHS |
|  | PF00067 | g21260.t1 | NA | p450 | p450 |
|  | PF00690 | g21261.t1 | E1-E2_ATPase | Cation_ATPase_N | E1-E2_ATPase |
|  | PF07859 | g21262.t1 | Chlorophyllase2 | Abhydrolase_3 | Chlorophyllase2 |
|  | PF00069 | g21263.t1 | NA | Pkinase | Pkinase |
|  | PF00106 | g21264.t1 | Epimerase | adh_short | Epimerase |
|  | NA | g21265.t1 | MIP | NA | MIP |
|  | NA | g21266.t1 | Peptidase_A22B | NA | Peptidase_A22B |
|  | NA | g21267.t1 | NA | NA | g21267.t1 |
|  | PF08245 | g21268.t1 | NA | Mur_ligase_M | Mur_ligase_M |
|  | PF13238 | g21269.t1 | DUF3741 | AAA_18 | DUF3741 |
|  | PF00958 | g21270.t1 | tRNA_Me_trans | GMP_synt_C | tRNA_Me_trans |
|  | PF03171 | g21271.t1 | DIOX_N | 2OG-FeII_Oxy | DIOX_N |
|  | PF00010 | g21272.t1 | bHLH-MYC_N | HLH | bHLH-MYC_N |
|  | PF00005 | g21273.t1 | tRNA-synt_1b | ABC_tran | tRNA-synt_1b |
|  | NA | g21274.t1 | NA | NA | g21274.t1 |
|  | PF00225 | g21275.t1 | NA | Kinesin | Kinesin |
|  | NA | g21276.t1 | Mito_carr | NA | Mito_carr |
|  | PF01167 | g21277.t1 | DUF3527 | Tub | DUF3527 |
|  | PF07500 | g21278.t1 | LRRNT_2 | TFIIS_M | LRRNT_2 |
|  | NA | g21279.t1 | NA | NA | g21279.t1 |
|  | PF00702 | g21280.t1 | HAD_2 | Hydrolase | HAD_2 |
|  | PF13806 | g21281.t1 | GCS2 | Rieske_2 | GCS2 |
|  | PF00091 | g21282.t1 | NA | Tubulin | Tubulin |
|  | PF07714 | g21283.t1 | TPR_16 | Pkinase_Tyr | TPR_16 |
|  | PF00168 | g21284.t1 | NA | C2 | C2 |
|  | PF02373 | g21285.t1 | PLU-1 | JmjC | PLU-1 |
|  | NA | g21286.t1 | NA | NA | g21286.t1 |
|  | PF13476 | g21287.t1 | PEARLI-4 | AAA_23 | PEARLI-4 |
|  | PF03479 | g21288.t1 | DUF4228 | DUF296 | DUF4228 |
|  | PF00578 | g21289.t1 | GSHPx | AhpC-TSA | GSHPx |
|  | PF00578 | g21290.t1 | GSHPx | AhpC-TSA | GSHPx |
|  | PF00578 | g21291.t1 | GSHPx | AhpC-TSA | GSHPx |
|  | PF07731 | g21292.t1 | Exostosin | Cu-oxidase_2 | Exostosin |
|  | PF00168 | g21293.t1 | PRT_C | C2 | PRT_C |
|  | NA | g21294.t1 | GASA | NA | GASA |
|  | PF02798 | g21295.t1 | BAG | GST_N | BAG |
|  | PF00702 | g21296.t1 | E1-E2_ATPase | Hydrolase | E1-E2_ATPase |
|  | PF00702 | g21297.t1 | E1-E2_ATPase | Hydrolase | E1-E2_ATPase |
|  | PF00702 | g21298.t1 | E1-E2_ATPase | Hydrolase | E1-E2_ATPase |
|  | PF13193 | g21299.t1 | AMP-binding | AMP-binding_C | AMP-binding |
|  | NA | g21300.t1 | PALP | NA | PALP |
|  | PF00091 | g21301.t1 | NA | Tubulin | Tubulin |
|  | PF07714 | g21302.t1 | zf-ANAPC11 | Pkinase_Tyr | zf-ANAPC11 |
|  | PF08772 | g21303.t1 | PIN_6 | NOB1_Zn_bind | PIN_6 |
|  | PF07714 | g21304.t1 | LRRNT_2 | Pkinase_Tyr | LRRNT_2 |
|  | PF03168 | g21305.t1 | Glyco_transf_90 | LEA_2 | Glyco_transf_90 |
|  | PF04525 | g21306.t1 | CBFB_NFYA | LOR | CBFB_NFYA |
|  | PF04525 | g21307.t1 | NA | LOR | LOR |
|  | PF04525 | g21308.t1 | NA | LOR | LOR |
|  | PF03810 | g21309.t1 | NT-C2 | IBN_N | NT-C2 |
|  | PF00808 | g21310.t1 | NA | CBFD_NFYB_HMF | CBFD_NFYB_HMF |
|  | NA | g21311.t1 | NA | NA | g21311.t1 |
|  | NA | g21312.t1 | Remorin_N | NA | Remorin_N |
|  | PF00656 | g21313.t1 | NA | Peptidase_C14 | Peptidase_C14 |
|  | PF00270 | g21314.t1 | Helicase_C | DEAD | Helicase_C |
|  | PF07714 | g21315.t1 | APH | Pkinase_Tyr | APH |
|  | PF00168 | g21316.t1 | Neprosin | C2 | Neprosin |
|  | PF01426 | g21317.t1 | Herpes_UL52 | BAH | Herpes_UL52 |
|  | PF01214 | g21318.t1 | Helicase_C | CK_II_beta | Helicase_C |
|  | PF13883 | g21319.t1 | DUF2470 | Pyrid_oxidase_2 | DUF2470 |
|  | PF17177 | g21320.t1 | Methyltransf_29 | PPR_long | Methyltransf_29 |
|  | PF07876 | g21321.t1 | Sec63 | Dabb | Sec63 |
|  | PF00270 | g21322.t1 | Helicase_C | DEAD | Helicase_C |
|  | PF03106 | g21323.t1 | PPI_Ypi1 | WRKY | PPI_Ypi1 |
|  | PF03106 | g21324.t1 | Tetraspannin | WRKY | Tetraspannin |
|  | PF00575 | g21325.t1 | Ribosomal_L21p | S1 | Ribosomal_L21p |
|  | PF00412 | g21326.t1 | zf-ANAPC11 | LIM | zf-ANAPC11 |
|  | PF00010 | g21327.t1 | SAGA-Tad1 | HLH | SAGA-Tad1 |
|  | PF13639 | g21328.t1 | zf-ANAPC11 | zf-RING_2 | zf-ANAPC11 |
|  | PF13949 | g21329.t1 | GRAS | ALIX_LYPXL_bnd | GRAS |
|  | PF00067 | g21330.t1 | BRAP2 | p450 | BRAP2 |
|  | PF02298 | g21331.t1 | GRAS | Cu_bind_like | GRAS |
|  | PF01429 | g21332.t1 | NA | MBD | MBD |
|  | NA | g21333.t1 | NA | NA | g21333.t1 |
|  | PF08767 | g21334.t1 | NA | CRM1_C | CRM1_C |
|  | PF00954 | g21335.t1 | H_PPase | S_locus_glycop | H_PPase |
|  | NA | g21336.t1 | Neprosin_AP | NA | Neprosin_AP |
|  | PF00954 | g21337.t1 | NA | S_locus_glycop | S_locus_glycop |
|  | PF00954 | g21338.t1 | NA | S_locus_glycop | S_locus_glycop |
|  | PF00199 | g21339.t1 | Catalase-rel | Catalase | Catalase-rel |
|  | PF00789 | g21340.t1 | NA | UBX | UBX |
|  | PF14555 | g21341.t1 | Asp | UBA_4 | Asp |
|  | PF01476 | g21342.t1 | PIG-U | LysM | PIG-U |
|  | PF00004 | g21343.t1 | Rad17 | AAA | Rad17 |
|  | PF00582 | g21344.t1 | NA | Usp | Usp |
|  | NA | g21345.t1 | NOT2_3_5 | NA | NOT2_3_5 |
|  | NA | g21346.t1 | NA | NA | g21346.t1 |
|  | PF07714 | g21347.t1 | APH | Pkinase_Tyr | APH |
|  | NA | g21348.t1 | COG2 | NA | COG2 |
|  | NA | g21349.t1 | DUF4005 | NA | DUF4005 |
|  | NA | g21350.t1 | Rhomboid | NA | Rhomboid |
|  | PF08542 | g21351.t1 | Rad17 | Rep_fac_C | Rad17 |
|  | NA | g21352.t1 | NA | NA | g21352.t1 |
|  | PF00013 | g21353.t1 | Amidase | KH_1 | Amidase |
|  | PF00300 | g21354.t1 | NA | His_Phos_1 | His_Phos_1 |
|  | NA | g21355.t1 | DUF1997 | NA | DUF1997 |
|  | PF01112 | g21356.t1 | NA | Asparaginase_2 | Asparaginase_2 |
|  | NA | g21357.t1 | DUF3444 | NA | DUF3444 |
|  | NA | g21358.t1 | NT-C2 | NA | NT-C2 |
|  | PF07992 | g15890.t1 | NA | Pyr_redox_2 | Pyr_redox_2 |
|  | NA | g15891.t1 | NA | NA | g15891.t1 |
|  | PF00226 | g15892.t1 | DUF3395 | DnaJ | DUF3395 |
|  | PF13649 | g15893.t1 | PrmA | Methyltransf_25 | PrmA |
|  | PF01230 | g15894.t1 | DcpS_C | HIT | DcpS_C |
|  | PF00571 | g15895.t1 | NA | CBS | CBS |
|  | PF14541 | g15896.t1 | Asp | TAXi_C | Asp |
|  | PF00650 | g15897.t1 | HSP20 | CRAL_TRIO | HSP20 |
|  | PF14559 | g15898.t1 | HSP20 | TPR_19 | HSP20 |
|  | PF00403 | g15899.t1 | Ribosomal_L23eN | HMA | Ribosomal_L23eN |
|  | PF10197 | g15900.t1 | NA | Cir_N | Cir_N |
|  | PF04438 | g15901.t1 | NA | zf-HIT | zf-HIT |
|  | PF00248 | g15902.t1 | NA | Aldo_ket_red | Aldo_ket_red |
|  | PF00010 | g15903.t1 | NA | HLH | HLH |
|  | NA | g15904.t1 | NA | NA | g15904.t1 |
|  | PF02866 | g15905.t1 | Ldh_1_N | Ldh_1_C | Ldh_1_N |
|  | PF00561 | g15906.t1 | GRAS | Abhydrolase_1 | GRAS |
|  | PF02991 | g15907.t1 | APG12 | Atg8 | APG12 |
|  | PF01266 | g15908.t1 | SE | DAO | SE |
|  | NA | g15909.t1 | tRNA_edit | NA | tRNA_edit |
|  | PF01266 | g15910.t1 | SE | DAO | SE |
|  | PF02866 | g15911.t1 | Ldh_1_N | Ldh_1_C | Ldh_1_N |
|  | PF01556 | g15912.t1 | NA | DnaJ_C | DnaJ_C |
|  | PF14547 | g15913.t1 | Tryp_alpha_amyl | Hydrophob_seed | Tryp_alpha_amyl |
|  | PF14547 | g15914.t1 | Tryp_alpha_amyl | Hydrophob_seed | Tryp_alpha_amyl |
|  | PF14547 | g15915.t1 | Tryp_alpha_amyl | Hydrophob_seed | Tryp_alpha_amyl |
|  | PF14547 | g15916.t1 | Tryp_alpha_amyl | Hydrophob_seed | Tryp_alpha_amyl |
|  | PF14547 | g15917.t1 | Tryp_alpha_amyl | Hydrophob_seed | Tryp_alpha_amyl |
|  | NA | g15918.t1 | PTR2 | NA | PTR2 |
|  | PF15413 | g15919.t1 | Oxysterol_BP | PH_11 | Oxysterol_BP |
|  | NA | g15920.t1 | PAP2 | NA | PAP2 |
|  | NA | g15921.t1 | MatE | NA | MatE |
|  | PF00156 | g15922.t1 | Gcd10p | Pribosyltran | Gcd10p |
|  | PF03106 | g15923.t1 | NA | WRKY | WRKY |
|  | NA | g15924.t1 | DUF863 | NA | DUF863 |
|  | PF07992 | g15925.t1 | FMO-like | Pyr_redox_2 | FMO-like |
|  | PF07992 | g15926.t1 | FMO-like | Pyr_redox_2 | FMO-like |
|  | PF07992 | g15927.t1 | FMO-like | Pyr_redox_2 | FMO-like |
|  | PF07992 | g15928.t1 | FMO-like | Pyr_redox_2 | FMO-like |
|  | PF00643 | g15929.t1 | PLATZ | zf-B_box | PLATZ |
|  | PF08541 | g15930.t1 | NA | ACP_syn_III_C | ACP_syn_III_C |
|  | NA | g15931.t1 | Exostosin | NA | Exostosin |
|  | NA | g15932.t1 | DOG1 | NA | DOG1 |
|  | NA | g15933.t1 | NA | NA | g15933.t1 |
|  | NA | g15934.t1 | NA | NA | g15934.t1 |
|  | NA | g15935.t1 | DUF3506 | NA | DUF3506 |
|  | PF08282 | g15936.t1 | Trehalose_PPase | Hydrolase_3 | Trehalose_PPase |
|  | PF00923 | g15937.t1 | NA | TAL_FSA | TAL_FSA |
|  | PF00251 | g15938.t1 | DUF3357 | Glyco_hydro_32N | DUF3357 |
|  | PF00394 | g15939.t1 | NA | Cu-oxidase | Cu-oxidase |
|  | PF00505 | g15940.t1 | YABBY | HMG_box | YABBY |
|  | PF00652 | g15941.t1 | DUF3474 | Ricin_B_lectin | DUF3474 |
|  | NA | g15942.t1 | Mem_trans | NA | Mem_trans |
|  | NA | g15943.t1 | MCRS_N | NA | MCRS_N |
|  | NA | g15944.t1 | NA | NA | g15944.t1 |
|  | NA | g15945.t1 | NAM | NA | NAM |
|  | PF00656 | g15946.t1 | Peptidase_C13 | Peptidase_C14 | Peptidase_C13 |
|  | PF14559 | g15947.t1 | TPR_16 | TPR_19 | TPR_16 |
|  | PF00009 | g15948.t1 | MMR_HSR1 | GTP_EFTU | MMR_HSR1 |
|  | PF04043 | g15949.t1 | NA | PMEI | PMEI |
|  | PF04043 | g15950.t1 | LTP_2 | PMEI | LTP_2 |
|  | PF04043 | g15951.t1 | LTP_2 | PMEI | LTP_2 |
|  | NA | g15952.t1 | DUF3537 | NA | DUF3537 |
|  | PF04043 | g15953.t1 | NA | PMEI | PMEI |
|  | NA | g15954.t1 | AOX | NA | AOX |
|  | PF13923 | g15955.t1 | LTP_2 | zf-C3HC4_2 | LTP_2 |
|  | PF14559 | g15956.t1 | TPR_16 | TPR_19 | TPR_16 |
|  | PF00249 | g15957.t1 | TPR_16 | Myb_DNA-binding | TPR_16 |
|  | PF03171 | g15958.t1 | COX17 | 2OG-FeII_Oxy | COX17 |
|  | PF13920 | g15959.t1 | Prok-RING_4 | zf-C3HC4_3 | Prok-RING_4 |
|  | PF05699 | g15960.t1 | DUF659 | Dimer_Tnp_hAT | DUF659 |
|  | PF00067 | g15961.t1 | TIP49 | p450 | TIP49 |
|  | PF00125 | g15962.t1 | Histone_H2A_C | Histone | Histone_H2A_C |
|  | PF00067 | g15963.t1 | FAE1_CUT1_RppA | p450 | FAE1_CUT1_RppA |
|  | PF03372 | g15964.t1 | NA | Exo_endo_phos | Exo_endo_phos |
|  | NA | g36435.t1 | PUNUT | NA | PUNUT |
|  | PF00071 | g36436.t1 | NA | Ras | Ras |
|  | NA | g36437.t1 | NA | NA | g36437.t1 |
|  | NA | g36438.t1 | NA | NA | g36438.t1 |
|  | NA | g36439.t1 | COPI_C | NA | COPI_C |
|  | NA | g36440.t1 | NA | NA | g36440.t1 |
|  | NA | g36441.t1 | NA | NA | g36441.t1 |
|  | NA | g36442.t1 | NA | NA | g36442.t1 |
|  | PF13833 | g36443.t1 | EF-hand_4 | EF-hand_8 | EF-hand_4 |
|  | PF13833 | g36444.t1 | EF-hand_4 | EF-hand_8 | EF-hand_4 |
|  | PF13833 | g36445.t1 | EF-hand_4 | EF-hand_8 | EF-hand_4 |
|  | PF00249 | g36446.t1 | NA | Myb_DNA-binding | Myb_DNA-binding |
|  | PF02878 | g36447.t1 | PGM_PMM_IV | PGM_PMM_I | PGM_PMM_IV |
|  | NA | g36448.t1 | NA | NA | g36448.t1 |
|  | NA | g36449.t1 | Vps55 | NA | Vps55 |
|  | PF09402 | g36450.t1 | NA | MSC | MSC |
|  | NA | g36451.t1 | Methyltransf_29 | NA | Methyltransf_29 |
|  | PF13847 | g36452.t1 | Methyltransf_29 | Methyltransf_31 | Methyltransf_29 |
|  | PF00249 | g36453.t1 | NA | Myb_DNA-binding | Myb_DNA-binding |
|  | NA | g36454.t1 | Tetraspannin | NA | Tetraspannin |
|  | NA | g36455.t1 | NA | NA | g36455.t1 |
|  | NA | g36456.t1 | NA | NA | g36456.t1 |
|  | PF03372 | g36457.t1 | NA | Exo_endo_phos | Exo_endo_phos |
|  | PF00141 | g36458.t1 | NA | peroxidase | peroxidase |
|  | PF13793 | g36459.t1 | NA | Pribosyltran_N | Pribosyltran_N |
|  | PF10241 | g36460.t1 | NA | KxDL | KxDL |
|  | NA | g36461.t1 | Got1 | NA | Got1 |
|  | PF04043 | g36462.t1 | NA | PMEI | PMEI |
|  | NA | g36463.t1 | NA | NA | g36463.t1 |
|  | PF00670 | g36464.t1 | NA | AdoHcyase_NAD | AdoHcyase_NAD |
|  | PF09416 | g36465.t1 | ResIII | UPF1_Zn_bind | ResIII |
|  | PF13649 | g36466.t1 | Methyltransf_16 | Methyltransf_25 | Methyltransf_16 |
|  | NA | g36467.t1 | NA | NA | g36467.t1 |
|  | PF03171 | g36468.t1 | DIOX_N | 2OG-FeII_Oxy | DIOX_N |
|  | PF00447 | g16403.t1 | APG6 | HSF_DNA-bind | APG6 |
|  | PF01202 | g16404.t1 | Intron_maturas2 | SKI | Intron_maturas2 |
|  | PF01369 | g16405.t1 | DUF1981 | Sec7 | DUF1981 |
|  | PF13360 | g16406.t1 | NA | PQQ_2 | PQQ_2 |
|  | NA | g16407.t1 | Rad1 | NA | Rad1 |
|  | PF17177 | g16408.t1 | PPR | PPR_long | PPR |
|  | PF05773 | g16409.t1 | LOB | RWD | LOB |
|  | PF08282 | g16410.t1 | Glyco_transf_20 | Hydrolase_3 | Glyco_transf_20 |
|  | PF00732 | g16411.t1 | FAD_binding_2 | GMC_oxred_N | FAD_binding_2 |
|  | PF00005 | g16412.t1 | ABC_membrane | ABC_tran | ABC_membrane |
|  | NA | g35214.t1 | Pectinesterase | NA | Pectinesterase |
|  | NA | g35215.t1 | Pectinesterase | NA | Pectinesterase |
|  | NA | g35216.t1 | NA | NA | g35216.t1 |
|  | NA | g35217.t1 | NA | NA | g35217.t1 |
|  | NA | g35218.t1 | NA | NA | g35218.t1 |
|  | PF01416 | g35219.t1 | NA | PseudoU_synth_1 | PseudoU_synth_1 |
|  | PF01416 | g35220.t1 | NA | PseudoU_synth_1 | PseudoU_synth_1 |
|  | PF00067 | g35221.t1 | Methyltransf_PK | p450 | Methyltransf_PK |
|  | PF00067 | g35222.t1 | Methyltransf_PK | p450 | Methyltransf_PK |
|  | NA | g35223.t1 | NA | NA | g35223.t1 |
|  | NA | g35224.t1 | NA | NA | g35224.t1 |
|  | PF00226 | g35225.t1 | DUF3444 | DnaJ | DUF3444 |
|  | NA | g35226.t1 | APG5 | NA | APG5 |
|  | PF00013 | g35227.t1 | 40S_S4_C | KH_1 | 40S_S4_C |
|  | NA | g35228.t1 | MFMR | NA | MFMR |
|  | PF00722 | g35229.t1 | XET_C | Glyco_hydro_16 | XET_C |
|  | PF14570 | g35230.t1 | Cellulose_synt | zf-RING_4 | Cellulose_synt |
|  | NA | g35231.t1 | NA | NA | g35231.t1 |
|  | PF00561 | g35232.t1 | Hydrolase_4 | Abhydrolase_1 | Hydrolase_4 |
|  | PF14829 | g35233.t1 | Acyltransferase | GPAT_N | Acyltransferase |
|  | PF00690 | g35234.t1 | NEMP | Cation_ATPase_N | NEMP |
|  | NA | g35235.t1 | DAD | NA | DAD |
|  | NA | g35236.t1 | NA | NA | g35236.t1 |
|  | PF07724 | g35237.t1 | NA | AAA_2 | AAA_2 |
|  | NA | g35238.t1 | Glyco_transf_8 | NA | Glyco_transf_8 |
|  | NA | g35239.t1 | NA | NA | g35239.t1 |
|  | NA | g35240.t1 | DPM2 | NA | DPM2 |
|  | PF13499 | g35241.t1 | EF-hand_4 | EF-hand_7 | EF-hand_4 |
|  | NA | g35242.t1 | PAP_fibrillin | NA | PAP_fibrillin |
|  | NA | g35243.t1 | NA | NA | g35243.t1 |
|  | PF01852 | g35244.t1 | Homeobox_KN | START | Homeobox_KN |
|  | NA | g35245.t1 | NA | NA | g35245.t1 |
|  | NA | g35246.t1 | NA | NA | g35246.t1 |
|  | PF12862 | g35247.t1 | NA | ANAPC5 | ANAPC5 |
|  | PF08284 | g35248.t1 | Retrotrans_gag | RVP_2 | Retrotrans_gag |
|  | PF00198 | g25267.t1 | Transferase | 2-oxoacid_dh | Transferase |
|  | PF01565 | g25268.t1 | Transferase | FAD_binding_4 | Transferase |
|  | PF13191 | g25269.t1 | NA | AAA_16 | AAA_16 |
|  | PF07651 | g25270.t1 | CAP_N | ANTH | CAP_N |
|  | NA | g25271.t1 | Branch | NA | Branch |
|  | NA | g25272.t1 | NA | NA | g25272.t1 |
|  | PF13959 | g25273.t1 | Helicase_C | DUF4217 | Helicase_C |
|  | PF00076 | g25274.t1 | NT-C2 | RRM_1 | NT-C2 |
|  | PF01453 | g25275.t1 | Npa1 | B_lectin | Npa1 |
|  | PF00141 | g25276.t1 | NopRA1 | peroxidase | NopRA1 |
|  | NA | g25277.t1 | TPX2 | NA | TPX2 |
|  | PF00646 | g25278.t1 | NA | F-box | F-box |
|  | PF00254 | g25279.t1 | DUF4050 | FKBP_C | DUF4050 |
|  | PF13091 | g25280.t1 | PLD_C | PLDc_2 | PLD_C |
|  | PF00847 | g25281.t1 | DUF1313 | AP2 | DUF1313 |
|  | NA | g25282.t1 | RdRP | NA | RdRP |
|  | PF02148 | g25283.t1 | BRAP2 | zf-UBP | BRAP2 |
|  | PF03105 | g25284.t1 | CNH | SPX | CNH |
|  | PF07859 | g25285.t1 | Chlorophyllase2 | Abhydrolase_3 | Chlorophyllase2 |
|  | PF07859 | g25286.t1 | Chlorophyllase2 | Abhydrolase_3 | Chlorophyllase2 |
|  | PF07859 | g25287.t1 | Chlorophyllase2 | Abhydrolase_3 | Chlorophyllase2 |
|  | PF07859 | g25288.t1 | Chlorophyllase2 | Abhydrolase_3 | Chlorophyllase2 |
|  | PF07859 | g25289.t1 | Chlorophyllase2 | Abhydrolase_3 | Chlorophyllase2 |
|  | PF00684 | g25290.t1 | NOC3p | DnaJ_CXXCXGXG | NOC3p |
|  | PF00270 | g25291.t1 | Helicase_C | DEAD | Helicase_C |
|  | PF07651 | g25292.t1 | Auxin_inducible | ANTH | Auxin_inducible |
|  | PF00069 | g25293.t1 | Haspin_kinase | Pkinase | Haspin_kinase |
|  | PF00069 | g25294.t1 | NA | Pkinase | Pkinase |
|  | NA | g25295.t1 | TPT | NA | TPT |
|  | PF17177 | g25296.t1 | DYW_deaminase | PPR_long | DYW_deaminase |
|  | PF13191 | g25297.t1 | AFG1_ATPase | AAA_16 | AFG1_ATPase |
|  | PF03330 | g25298.t1 | NA | DPBB_1 | DPBB_1 |
|  | PF03330 | g25299.t1 | NA | DPBB_1 | DPBB_1 |
|  | NA | g25300.t1 | Mito_carr | NA | Mito_carr |
|  | PF14569 | g25301.t1 | Cellulose_synt | zf-UDP | Cellulose_synt |
|  | PF13874 | g25302.t1 | bZIP_1 | Nup54 | bZIP_1 |
|  | PF00215 | g25303.t1 | DUF4666 | OMPdecase | DUF4666 |
|  | PF00046 | g25304.t1 | Cwf_Cwc_15 | Homeobox | Cwf_Cwc_15 |
|  | PF16421 | g25305.t1 | GCN5L1 | E2F_CC-MB | GCN5L1 |
|  | NA | g25306.t1 | DUF247 | NA | DUF247 |
|  | NA | g25307.t1 | DUF247 | NA | DUF247 |
|  | NA | g25308.t1 | DUF247 | NA | DUF247 |
|  | PF00179 | g25309.t1 | DUF247 | UQ_con | DUF247 |
|  | PF00641 | g25310.t1 | ALMT | zf-RanBP | ALMT |
|  | PF01453 | g25311.t1 | Glycos_transf_1 | B_lectin | Glycos_transf_1 |
|  | PF03638 | g25312.t1 | Pectinesterase | TCR | Pectinesterase |
|  | PF02230 | g25313.t1 | Hydrolase_4 | Abhydrolase_2 | Hydrolase_4 |
|  | NA | g25314.t1 | Rcd1 | NA | Rcd1 |
|  | PF01215 | g25315.t1 | NA | COX5B | COX5B |
|  | PF00160 | g25316.t1 | DUF21 | Pro_isomerase | DUF21 |
|  | NA | g25317.t1 | Ammonium_transp | NA | Ammonium_transp |
|  | PF17177 | g25318.t1 | DYW_deaminase | PPR_long | DYW_deaminase |
|  | PF00145 | g25319.t1 | NA | DNA_methylase | DNA_methylase |
|  | PF17177 | g25320.t1 | PPR | PPR_long | PPR |
|  | NA | g25321.t1 | NA | NA | g25321.t1 |
|  | PF05641 | g25322.t1 | ENT | Agenet | ENT |
|  | NA | g25323.t1 | DUF1645 | NA | DUF1645 |
|  | PF00071 | g25324.t1 | MMR_HSR1 | Ras | MMR_HSR1 |
|  | PF00071 | g25325.t1 | MMR_HSR1 | Ras | MMR_HSR1 |
|  | PF04862 | g25326.t1 | NA | DUF642 | DUF642 |
|  | PF00076 | g25327.t1 | UCR_14kD | RRM_1 | UCR_14kD |
|  | PF13637 | g25328.t1 | Ion_trans | Ank_4 | Ion_trans |
|  | PF02779 | g25329.t1 | DXP_synthase_N | Transket_pyr | DXP_synthase_N |
|  | PF17177 | g25330.t1 | PPR | PPR_long | PPR |
|  | PF06200 | g25331.t1 | NA | tify | tify |
|  | PF03171 | g25332.t1 | DIOX_N | 2OG-FeII_Oxy | DIOX_N |
|  | NA | g25333.t1 | V_ATPase_I | NA | V_ATPase_I |
|  | PF03171 | g25334.t1 | DIOX_N | 2OG-FeII_Oxy | DIOX_N |
|  | NA | g25335.t1 | Coatomer_WDAD | NA | Coatomer_WDAD |
|  | PF08513 | g25336.t1 | Coatomer_WDAD | LisH | Coatomer_WDAD |
|  | NA | g25337.t1 | NA | NA | g25337.t1 |
|  | PF07992 | g25338.t1 | Pyr_redox_3 | Pyr_redox_2 | Pyr_redox_3 |
|  | NA | g25339.t1 | NA | NA | g25339.t1 |
|  | NA | g25340.t1 | NA | NA | g25340.t1 |
|  | PF14510 | g25341.t1 | NA | ABC_trans_N | ABC_trans_N |
|  | PF16679 | g25342.t1 | NA | CDT1_C | CDT1_C |
|  | PF00954 | g25343.t1 | NA | S_locus_glycop | S_locus_glycop |
|  | PF14510 | g25344.t1 | NA | ABC_trans_N | ABC_trans_N |
|  | NA | g25345.t1 | E1-E2_ATPase | NA | E1-E2_ATPase |
|  | NA | g25346.t1 | Ctr | NA | Ctr |
|  | PF00270 | g25347.t1 | Transferase | DEAD | Transferase |
|  | PF14510 | g25348.t1 | PDR_assoc | ABC_trans_N | PDR_assoc |
|  | NA | g25349.t1 | PP2C | NA | PP2C |
|  | PF13415 | g25350.t1 | NA | Kelch_3 | Kelch_3 |
|  | NA | g25351.t1 | NA | NA | g25351.t1 |
|  | NA | g25352.t1 | NA | NA | g25352.t1 |
|  | NA | g25353.t1 | ATP-synt_C | NA | ATP-synt_C |
|  | PF13450 | g25354.t1 | FAD_binding_3 | NAD_binding_8 | FAD_binding_3 |
|  | PF13508 | g25355.t1 | Acetyltransf_1 | Acetyltransf_7 | Acetyltransf_1 |
|  | NA | g25356.t1 | NA | NA | g25356.t1 |
|  | PF03372 | g25357.t1 | NA | Exo_endo_phos | Exo_endo_phos |
|  | PF08541 | g25358.t1 | FAE1_CUT1_RppA | ACP_syn_III_C | FAE1_CUT1_RppA |
|  | PF00004 | g25359.t1 | TIP49 | AAA | TIP49 |
|  | PF03330 | g25360.t1 | Exostosin | DPBB_1 | Exostosin |
|  | PF03330 | g25361.t1 | NA | DPBB_1 | DPBB_1 |
|  | PF17177 | g25362.t1 | PPR | PPR_long | PPR |
|  | PF03330 | g25363.t1 | NA | DPBB_1 | DPBB_1 |
|  | PF17177 | g25364.t1 | PPR | PPR_long | PPR |
|  | PF03330 | g25365.t1 | NA | DPBB_1 | DPBB_1 |
|  | PF00931 | g25366.t1 | PPR | NB-ARC | PPR |
|  | PF17177 | g25367.t1 | PPR | PPR_long | PPR |
|  | NA | g25368.t1 | DYW_deaminase | NA | DYW_deaminase |
|  | PF03330 | g25369.t1 | MBOAT_2 | DPBB_1 | MBOAT_2 |
|  | PF02518 | g25370.t1 | HSP90 | HATPase_c | HSP90 |
|  | PF17177 | g25371.t1 | PPR | PPR_long | PPR |
|  | PF03501 | g25372.t1 | NA | S10_plectin | S10_plectin |
|  | PF00249 | g25373.t1 | NA | Myb_DNA-binding | Myb_DNA-binding |
|  | NA | g25374.t1 | BPS1 | NA | BPS1 |
|  | PF07992 | g25375.t1 | Pyr_redox_3 | Pyr_redox_2 | Pyr_redox_3 |
|  | PF00403 | g25376.t1 | Asp_Glu_race | HMA | Asp_Glu_race |
|  | PF00155 | g25377.t1 | NA | Aminotran_1_2 | Aminotran_1_2 |
|  | PF00326 | g25378.t1 | YgbB | Peptidase_S9 | YgbB |
|  | NA | g25379.t1 | DDA1 | NA | DDA1 |
|  | PF12656 | g25380.t1 | G-patch | G-patch_2 | G-patch |
|  | PF04406 | g25381.t1 | SVIP | TP6A_N | SVIP |
|  | PF03106 | g25382.t1 | Ku_C | WRKY | Ku_C |
|  | PF00759 | g25383.t1 | TRAP_alpha | Glyco_hydro_9 | TRAP_alpha |
|  | PF05726 | g25384.t1 | Pirin | Pirin_C | Pirin |
|  | PF14559 | g25385.t1 | TPR_16 | TPR_19 | TPR_16 |
|  | PF04969 | g25386.t1 | NA | CS | CS |
|  | PF02891 | g25387.t1 | BET | zf-MIZ | BET |
|  | PF00149 | g25388.t1 | STPPase_N | Metallophos | STPPase_N |
|  | NA | g25389.t1 | NA | NA | g25389.t1 |
|  | PF04525 | g25390.t1 | Lig_chan | LOR | Lig_chan |
|  | PF08030 | g25391.t1 | NADPH_Ox | NAD_binding_6 | NADPH_Ox |
|  | PF03168 | g25392.t1 | NA | LEA_2 | LEA_2 |
|  | PF03168 | g25393.t1 | NA | LEA_2 | LEA_2 |

| ***V. vinifera*** | |  |  |  |
| --- | --- | --- | --- | --- |
| **PFAM** | **SeqID** | **PFAM_NAME** | **DOM_NAME** | **BRIEF NAME** |
| NA | Vitvi02g00484.t01 | NA | NA | Vitvi02g00484 |
| PF13499 | Vitvi02g00485.t01 | Caleosin | EF-hand_7 | Caleosin |
| PF00561 | Vitvi02g00486.t01 | Hydrolase_4 | Abhydrolase_1 | Hydrolase_4 |
| NA | Vitvi02g00487.t01 | NA | NA | Vitvi02g00487 |
| PF01936 | Vitvi02g00488.t01 | DUF629 | NYN | DUF629 |
| PF16363 | Vitvi02g00489.t01 | Epimerase | GDP_Man_Dehyd | Epimerase |
| NA | Vitvi02g00490.t01 | NA | NA | Vitvi02g00490 |
| NA | Vitvi02g00491.t01 | NA | NA | Vitvi02g00491 |
| NA | Vitvi02g00492.t01 | Retrotran_gag_2 | NA | Retrotran_gag_2 |
| PF00067 | Vitvi02g00493.t01 | NA | p450 | p450 |
| NA | Vitvi02g00494.t01 | NA | NA | Vitvi02g00494 |
| NA | Vitvi02g01426.t01 | NA | NA | Vitvi02g01426 |
| PF00067 | Vitvi02g01427.t01 | NA | p450 | p450 |
| PF05699 | Vitvi02g00495.t01 | DUF659 | Dimer_Tnp_hAT | DUF659 |
| PF06747 | Vitvi02g00496.t01 | COX17 | CHCH | COX17 |
| NA | Vitvi02g00497.t01 | NA | NA | Vitvi02g00497 |
| NA | Vitvi02g00498.t01 | NA | NA | Vitvi02g00498 |
| PF04043 | Vitvi02g00499.t01 | NA | PMEI | PMEI |
| PF04043 | Vitvi02g00500.t01 | NA | PMEI | PMEI |
| PF04043 | Vitvi02g00501.t01 | NA | PMEI | PMEI |
| PF00009 | Vitvi02g00502.t01 | MMR_HSR1 | GTP_EFTU | MMR_HSR1 |
| NA | Vitvi02g00503.t01 | NA | NA | Vitvi02g00503 |
| NA | Vitvi02g00504.t01 | NA | NA | Vitvi02g00504 |
| PF00656 | Vitvi02g00505.t01 | Peptidase_C13 | Peptidase_C14 | Peptidase_C13 |
| NA | Vitvi02g00507.t01 | Auxin_inducible | NA | Auxin_inducible |
| NA | Vitvi02g00508.t01 | NAM | NA | NAM |
| NA | Vitvi02g00509.t01 | NA | NA | Vitvi02g00509 |
| PF00505 | Vitvi02g00510.t01 | YABBY | HMG_box | YABBY |
| PF00394 | Vitvi02g00511.t01 | NA | Cu-oxidase | Cu-oxidase |
| PF00251 | Vitvi02g00512.t01 | NA | Glyco_hydro_32N | Glyco_hydro_32N |
| PF00923 | Vitvi02g00513.t01 | NA | TAL_FSA | TAL_FSA |
| PF08282 | Vitvi02g00514.t01 | Trehalose_PPase | Hydrolase_3 | Trehalose_PPase |
| NA | Vitvi02g00515.t01 | DOG1 | NA | DOG1 |
| NA | Vitvi02g00516.t01 | Exostosin | NA | Exostosin |
| PF08541 | Vitvi02g00517.t01 | NA | ACP_syn_III_C | ACP_syn_III_C |
| NA | Vitvi02g00518.t01 | NA | NA | Vitvi02g00518 |
| PF07992 | Vitvi02g00519.t01 | FMO-like | Pyr_redox_2 | FMO-like |
| PF07992 | Vitvi02g00521.t01 | FMO-like | Pyr_redox_2 | FMO-like |
| PF07992 | Vitvi02g00522.t01 | FMO-like | Pyr_redox_2 | FMO-like |
| NA | Vitvi02g00523.t01 | NA | NA | Vitvi02g00523 |
| PF07992 | Vitvi02g00524.t01 | FMO-like | Pyr_redox_2 | FMO-like |
| NA | Vitvi02g00525.t01 | DUF863 | NA | DUF863 |
| NA | Vitvi02g00526.t01 | NA | NA | Vitvi02g00526 |
| NA | Vitvi02g00527.t01 | PAP2 | NA | PAP2 |
| PF15413 | Vitvi02g00528.t01 | Oxysterol_BP | PH_11 | Oxysterol_BP |
| NA | Vitvi02g00529.t01 | PTR2 | NA | PTR2 |
| NA | Vitvi02g00530.t01 | NA | NA | Vitvi02g00530 |
| PF14547 | Vitvi02g00531.t01 | Tryp_alpha_amyl | Hydrophob_seed | Tryp_alpha_amyl |
| PF14547 | Vitvi02g00532.t01 | Tryp_alpha_amyl | Hydrophob_seed | Tryp_alpha_amyl |
| PF02866 | Vitvi02g00533.t01 | Ldh_1_N | Ldh_1_C | Ldh_1_N |
| NA | Vitvi02g00534.t01 | tRNA_edit | NA | tRNA_edit |
| PF02991 | Vitvi02g00535.t01 | APG12 | Atg8 | APG12 |
| NA | Vitvi02g00538.t01 | NA | NA | Vitvi02g00538 |
| NA | Vitvi02g00540.t01 | NA | NA | Vitvi02g00540 |
| PF04438 | Vitvi02g00541.t01 | NA | zf-HIT | zf-HIT |
| PF10197 | Vitvi02g00542.t01 | NA | Cir_N | Cir_N |
| PF00403 | Vitvi02g00543.t01 | PTEN_C2 | HMA | PTEN_C2 |
| NA | Vitvi02g00544.t01 | NA | NA | Vitvi02g00544 |
| PF00635 | Vitvi02g00545.t01 | NA | Motile_Sperm | Motile_Sperm |
| PF00650 | Vitvi02g00546.t01 | NA | CRAL_TRIO | CRAL_TRIO |
| PF14543 | Vitvi02g00547.t01 | Asp | TAXi_N | Asp |
| PF01230 | Vitvi02g00548.t01 | DcpS_C | HIT | DcpS_C |
| PF13649 | Vitvi02g00549.t01 | PrmA | Methyltransf_25 | PrmA |
| PF00226 | Vitvi02g00551.t01 | DUF3395 | DnaJ | DUF3395 |
| NA | Vitvi02g00552.t01 | NA | NA | Vitvi02g00552 |
| NA | Vitvi02g00553.t01 | Pectinesterase | NA | Pectinesterase |
| PF00745 | Vitvi02g00554.t01 | Pex19 | GlutR_dimer | Pex19 |
| PF12874 | Vitvi02g00555.t01 | zf-C2H2_jaz | zf-met | zf-C2H2_jaz |
| NA | Vitvi02g01428.t01 | NA | NA | Vitvi02g01428 |
| PF14559 | Vitvi02g01429.t01 | TPR_16 | TPR_19 | TPR_16 |
| NA | Vitvi02g01430.t01 | NA | NA | Vitvi02g01430 |
| PF04043 | Vitvi02g01431.t01 | NA | PMEI | PMEI |
| NA | Vitvi02g01432.t01 | NA | NA | Vitvi02g01432 |
| PF00652 | Vitvi02g01433.t01 | NA | Ricin_B_lectin | Ricin_B_lectin |
| PF00643 | Vitvi02g01434.t01 | PLATZ | zf-B_box | PLATZ |
| PF03106 | Vitvi02g01435.t01 | NA | WRKY | WRKY |
| NA | Vitvi02g01436.t01 | Oxysterol_BP | NA | Oxysterol_BP |
| NA | Vitvi02g01437.t01 | NA | NA | Vitvi02g01437 |
| PF14547 | Vitvi02g01438.t01 | Tryp_alpha_amyl | Hydrophob_seed | Tryp_alpha_amyl |
| PF14547 | Vitvi02g01439.t01 | Tryp_alpha_amyl | Hydrophob_seed | Tryp_alpha_amyl |
| PF14547 | Vitvi02g01440.t01 | Tryp_alpha_amyl | Hydrophob_seed | Tryp_alpha_amyl |
| NA | Vitvi02g01441.t01 | NA | NA | Vitvi02g01441 |
| NA | Vitvi02g01442.t01 | Intron_maturas2 | NA | Intron_maturas2 |
| NA | Vitvi02g01443.t01 | NA | NA | Vitvi02g01443 |
| NA | Vitvi02g01444.t01 | NA | NA | Vitvi02g01444 |
| NA | Vitvi02g01445.t01 | NA | NA | Vitvi02g01445 |
| NA | Vitvi02g01446.t01 | HSP20 | NA | HSP20 |
| NA | Vitvi02g01447.t01 | HSP20 | NA | HSP20 |
| NA | Vitvi02g01448.t01 | NA | NA | Vitvi02g01448 |
| NA | Vitvi02g01449.t01 | NA | NA | Vitvi02g01449 |
| NA | Vitvi02g01450.t01 | NA | NA | Vitvi02g01450 |
| NA | Vitvi02g01451.t01 | RNA_pol_Rpc34 | NA | RNA_pol_Rpc34 |
| NA | VviHAM1.t01 | NA | NA | VviHAM1 |
| PF00036 | Vitvi02g00586.t01 | EF-hand_4 | EF-hand_1 | EF-hand_4 |
| PF10559 | Vitvi02g00556.t01 | SecY | Plug_translocon | SecY |
| NA | Vitvi02g00557.t01 | NA | NA | Vitvi02g00557 |
| PF00152 | Vitvi02g00558.t01 | NA | tRNA-synt_2 | tRNA-synt_2 |
| PF12931 | Vitvi02g00559.t01 | NA | Sec16_C | Sec16_C |
| PF13912 | Vitvi02g00560.t01 | zf-C2H2_jaz | zf-C2H2_6 | zf-C2H2_jaz |
| NA | Vitvi02g00561.t01 | EamA | NA | EamA |
| PF17177 | Vitvi02g00563.t01 | PPR | PPR_long | PPR |
| PF00413 | Vitvi02g00564.t01 | NA | Peptidase_M10 | Peptidase_M10 |
| NA | Vitvi02g00565.t01 | O-FucT | NA | O-FucT |
| PF05641 | Vitvi02g00566.t01 | NA | Agenet | Agenet |
| NA | Vitvi02g00567.t01 | Lzipper-MIP1 | NA | Lzipper-MIP1 |
| NA | Vitvi02g00568.t01 | MIP | NA | MIP |
| PF16113 | Vitvi02g00569.t01 | NA | ECH_2 | ECH_2 |
| NA | Vitvi02g00570.t01 | DUF707 | NA | DUF707 |
| NA | Vitvi02g00571.t01 | NA | NA | Vitvi02g00571 |
| PF00551 | Vitvi02g00572.t01 | NA | Formyl_trans_N | Formyl_trans_N |
| NA | Vitvi02g00573.t01 | PS_Dcarbxylase | NA | PS_Dcarbxylase |
| NA | Vitvi02g00574.t01 | NA | NA | Vitvi02g00574 |
| PF02894 | Vitvi02g00575.t01 | NA | GFO_IDH_MocA_C | GFO_IDH_MocA_C |
| PF05192 | Vitvi02g00576.t01 | NA | MutS_III | MutS_III |
| PF14547 | Vitvi02g00578.t01 | LTP_2 | Hydrophob_seed | LTP_2 |
| NA | Vitvi02g00579.t01 | NA | NA | Vitvi02g00579 |
| PF00069 | Vitvi02g00580.t01 | Choline_kinase | Pkinase | Choline_kinase |
| PF00036 | Vitvi02g00581.t01 | EF-hand_4 | EF-hand_1 | EF-hand_4 |
| NA | Vitvi02g00582.t01 | NA | NA | Vitvi02g00582 |
| PF00149 | Vitvi02g00583.t01 | NA | Metallophos | Metallophos |
| NA | Vitvi02g00584.t01 | NA | NA | Vitvi02g00584 |
| PF00067 | Vitvi02g00585.t01 | NA | p450 | p450 |
| PF00149 | Vitvi02g00587.t01 | NA | Metallophos | Metallophos |
| NA | Vitvi02g00588.t01 | PPR | NA | PPR |
| NA | Vitvi02g00589.t01 | NA | NA | Vitvi02g00589 |
| NA | Vitvi02g00590.t01 | RAI1 | NA | RAI1 |
| PF06217 | Vitvi02g00593.t01 | Nop14 | GAGA_bind | Nop14 |
| NA | Vitvi02g00594.t01 | RAI1 | NA | RAI1 |
| NA | Vitvi02g00595.t01 | NA | NA | Vitvi02g00595 |
| PF01214 | Vitvi02g00597.t01 | NA | CK_II_beta | CK_II_beta |
| NA | Vitvi02g00598.t01 | NA | NA | Vitvi02g00598 |
| PF06220 | Vitvi02g00599.t01 | Ribosomal_L37ae | zf-U1 | Ribosomal_L37ae |
| NA | Vitvi02g00600.t01 | SNF2_N | NA | SNF2_N |
| NA | Vitvi02g00601.t01 | NA | NA | Vitvi02g00601 |
| NA | Vitvi02g00602.t01 | NA | NA | Vitvi02g00602 |
| PF07714 | Vitvi02g00604.t01 | APH | Pkinase_Tyr | APH |
| PF01373 | Vitvi02g00605.t01 | NA | Glyco_hydro_14 | Glyco_hydro_14 |
| NA | Vitvi02g00606.t01 | XendoU | NA | XendoU |
| PF00169 | Vitvi02g00607.t01 | VPS13 | PH | VPS13 |
| PF00412 | Vitvi02g00614.t01 | zf-FLZ | LIM | zf-FLZ |
| NA | Vitvi02g00615.t01 | DUF702 | NA | DUF702 |
| NA | Vitvi02g01452.t01 | NA | NA | Vitvi02g01452 |
| NA | LOC100247746.t01 | NA | NA | LOC100247746 |
| PF14547 | Vitvi02g01455.t01 | LTP_2 | Hydrophob_seed | LTP_2 |
| PF14547 | Vitvi02g01456.t01 | LTP_2 | Hydrophob_seed | LTP_2 |
| PF14547 | Vitvi02g01457.t01 | LTP_2 | Hydrophob_seed | LTP_2 |
| PF00160 | Vitvi02g01458.t01 | NA | Pro_isomerase | Pro_isomerase |
| PF14547 | Vitvi02g01459.t01 | LTP_2 | Hydrophob_seed | LTP_2 |
| PF14547 | Vitvi02g01460.t01 | LTP_2 | Hydrophob_seed | LTP_2 |
| PF14547 | Vitvi02g01461.t01 | LTP_2 | Hydrophob_seed | LTP_2 |
| NA | Vitvi02g01462.t01 | NA | NA | Vitvi02g01462 |
| PF14547 | Vitvi02g01463.t01 | LTP_2 | Hydrophob_seed | LTP_2 |
| PF14547 | Vitvi02g01464.t01 | LTP_2 | Hydrophob_seed | LTP_2 |
| NA | Vitvi02g01465.t01 | NA | NA | Vitvi02g01465 |
| PF14547 | Vitvi02g01466.t01 | LTP_2 | Hydrophob_seed | LTP_2 |
| NA | Vitvi02g01467.t01 | NA | NA | Vitvi02g01467 |
| NA | Vitvi02g01468.t01 | NA | NA | Vitvi02g01468 |
| NA | Vitvi02g01469.t01 | NA | NA | Vitvi02g01469 |
| NA | Vitvi02g01470.t01 | NA | NA | Vitvi02g01470 |
| PF13920 | Vitvi02g01471.t01 | Prok-RING_4 | zf-C3HC4_3 | Prok-RING_4 |
| PF05362 | Vitvi02g00617.t01 | LON_substr_bdg | Lon_C | LON_substr_bdg |
| PF03171 | Vitvi02g00618.t01 | DIOX_N | 2OG-FeII_Oxy | DIOX_N |
| PF03171 | Vitvi02g00619.t01 | DIOX_N | 2OG-FeII_Oxy | DIOX_N |
| PF03171 | Vitvi02g00620.t01 | DIOX_N | 2OG-FeII_Oxy | DIOX_N |
| NA | Vitvi02g00622.t01 | NA | NA | Vitvi02g00622 |
| PF03171 | Vitvi02g00623.t01 | DIOX_N | 2OG-FeII_Oxy | DIOX_N |
| NA | Vitvi02g00626.t01 | NA | NA | Vitvi02g00626 |
| PF03171 | Vitvi02g01472.t01 | DIOX_N | 2OG-FeII_Oxy | DIOX_N |
| PF03171 | Vitvi02g01473.t01 | DIOX_N | 2OG-FeII_Oxy | DIOX_N |
| PF03171 | Vitvi02g01474.t01 | DIOX_N | 2OG-FeII_Oxy | DIOX_N |
| PF13561 | Vitvi02g01475.t01 | NA | adh_short_C2 | adh_short_C2 |
| NA | Vitvi02g01476.t01 | NA | NA | Vitvi02g01476 |
| NA | Vitvi02g01477.t01 | DIOX_N | NA | DIOX_N |
| PF03171 | Vitvi02g01478.t01 | NA | 2OG-FeII_Oxy | 2OG-FeII_Oxy |
| PF03171 | Vitvi02g01479.t01 | DIOX_N | 2OG-FeII_Oxy | DIOX_N |
