# Supplementary material for: Draft genome of the Native American cold hardy grapevine Vitis riparia Michx. ‘Manitoba 37’
Source: Hortic Res. 2020 Jun 1;7:92. doi: 10.1038/s41438-020-0316-2 (PMC7261805; doi:10.1038/s41438-020-0316-2)
Supplement: Supplementary file 15 — Supplementary Table 10 [file 41438_2020_316_MOESM15_ESM.docx]

**Supplementary Table 10a. DNA library and filtering of raw reads information for *Vitis riparia* ‘Manitoba 37’.**

| **Library Type** | **Sequencer** | **Library insert size (bp)** | **Read length (nt)** | **Raw reads (M) (forward & reverse)** | **Raw Coverage (X) (forward & reverse)** | **Filtered reads* (M) (forward & reverse)** | **Final Coverage (X) (forward & reverse)** |
| --- | --- | --- | --- | --- | --- | --- | --- |
| Paired-end | Illumina I | 346 | 100 | 341 | 68.2 | 213.4 | 39.2 |
| Paired-end |  | 473 | 100 | 383.4 | 76.7 | 281 | 51.7 |
| Paired-end |  | 478 | 100 | 396.8 | 79.3 | 274.2 | 50.4 |
| Paired-end | Illumina Hiseq 2500 | 600 | 160 | 134.6 | 43.1 | 88.2 | 22.9 |
| Paired-end -overlapping |  | 450 | 260 | 292.6 | 152.1 | 150.4 | 107.4 |
| Paired-end -overlapping |  | 250 | 160 | 128.4 | 41.1 | 58.1 | 26.6 |
| Mate-pair |  | 3-5 kb | 160 | 195.4 | 62.5 | 83.2 | 24 |
| Mate-pair |  | 8-10 kb | 160 | 220.6 | 70.6 | 90.4 | 26 |
| Mate-pair |  | 15-20 kb | 160 | 202.6 | 64.8 | 74.8 | 21.5 |
| **Total** |  |  |  | **2295.4** | **658.4** | **1313.7** | **369.7** |

* Filtered reads were obtained in several steps. First adaptors were removed from both ends using cutadapt for paired-end libraries and perl script was used for mate-pairs libraries to remove adaptors. Using Trimmomatic with parameters (HEADCROP:8 LEADING:30 TRAILING:30 MINLEN:70) performed for all paired-end reads and for mate-pairs all parameters were same except MINLEN:40. We checked FastQC reports of all reads obtained from previous step then we performed SLIDINGWINDOW:3:20 using Trimmomatic for 346, 473, 478, 450 and 600 libraries.

PEAR was used to merge 250 and 450 libraries separately which resulted three .fastq files of each library as merged.fastq, unmerged-forward.fastq and unmerged-reverse.fastq. For 250 unmerged files we performed SLIDINGWINDOW:3:20 MINLEN:70 and for 450 unmerged files we performed SLIDINGWINDOW:3:20 MINLEN:70 HEADCROP:2 using Trimmomatic.

Duplicates from all reads then removed using FastUniq. Reads then corrected using Quake with k-mer 19. In the end few bases were removed from some libraries using NGSTOOLKIT. For all mate-pairs libraries, 8 bases removed from right end. In 250 library, 50 bases removed from right end in 250 merged file and 9 bases from left side, 8 bases from right end removed in 250 unmerged files. In 450 library, 25 bases removed from right end in 450 merged file and 2 bases removed from left end in 450 unmerged files. In 600 library, 14 bases from left end and 8 bases removed from right end. After this step total 1313.7 M reads were considered for *de novo* genome assembly. **Supplementary Table 10. Methods references for genome assembly and evaluation tools.**

**A. DNA sequencing and pre-processing of reads**

1. Martin, M. Cutadapt removes adapter sequences from high-throughput sequencing reads. *EMBnet.journal* **17**, 10–12 (2011).
2. Bolger, A. M., Lohse, M. & Usadel, B. Trimmomatic: A flexible trimmer for Illumina Sequence Data. *Bioinformatics* **30**, 2114-2120 (2014).
3. Zhang, J., Kobert, K., Flouri, T. & Stamatakis, A. PEAR: a fast and accurate Illumina Paired-End reAd mergeR. *Bioinformatics* **30**, 614–620 (2014).
4. Xu, H. et al. FastUniq: a fast de novo duplicates removal tool for paired short reads. *Plos One* **7**, e52249 (2012).
5. Kelley, D. R., Schatz, M. C. & Salzberg, S. L. Quake: quality-aware detection and correction of sequencing errors. Genome Biol. **11,** R116 (2010).
6. Patel, R. K. & Jain, M. NGS QC Toolkit: A Toolkit for Quality Control of Next Generation Sequencing Data. *PLOS ONE* **7**, e30619 (2012).
7. Marçais, G. & Kingsford, C. A fast, lock-free approach for efficient parallel counting of occurrences of k-mers. *Bioinformatics* **27**, 764–770 (2011).
8. Vurture, G. W. et al. GenomeScope: fast reference-free genome profiling from short reads. *Bioinformatics* **33**, 2202–2204 (2017).
9. Langmead, B. & Salzberg, S. L. Fast gapped-read alignment with Bowtie 2. *Nat. Methods* **9**, 357–359 (2012).
10. Li, H. et al. The Sequence Alignment/Map format and SAMtools. *Bioinformatics* **25**, 2078–2079 (2009).

**B. De novo heterozygous genome assembly of *V. riparia* Manitoba 37.**

1. Luo, R. et al. SOAPdenovo2: an empirically improved memory-efficient short-read de novo assembler. *GigaScience* **1**, 18 (2012).
2. Schmieder, R. & Edwards, R. Fast identification and removal of sequence contamination from genomic and metagenomic datasets. *Plos One* **6**, e17288 (2011).
3. Hunt, M. et al. REAPR: a universal tool for genome assembly evaluation. *Genome Biol.* **14**, R47 (2013).
4. Cabanettes, F. & Klopp, C. D-GENIES: dot plot large genomes in an interactive efficient and simple way. *Peer J.* **6**, e4958 (2018).
5. Wu, T. D. & Watanabe, C. K. GMAP: a genomic mapping and alignment program for mRNA and EST sequences. *Bioinformatics* **21**, 1859–1875 (2005).
6. Kent, W. J. BLAT--the BLAST-like alignment tool. *Genome Res*. **12**, 656–664 (2002).
7. Kurtz, S. et al. Versatile and open software for comparing large genomes. *Genome Biol*. **5**, R12 (2004).

**C. *V. riparia* SNP calling**

1. Langmead, B. & Salzberg, S. L. Fast gapped-read alignment with Bowtie 2. *Nat. Methods* **9**, 357–359 (2012).
2. Li, H. et al. The Sequence Alignment/Map format and SAMtools. *Bioinformatics* **25**, 2078–2079 (2009).
3. Cingolani, P. et al. A program for annotating and predicting the effects of single nucleotide polymorphisms, SnpEff: SNPs in the genome of Drosophila melanogaster strain w1118; iso-2; iso-3. *Fly (Austin)* **6**, 80–92 (2012).

**D. De novo gene prediction and functional annotation.**

1. Smit, A. F. A. & Hubley, R. RepeatModeler Open-1.0. 2008-2015 <http://www.repeatmasker.org> (Accessed: 25th July 2019).
2. Hoff, K. J., Lange, S., Lomsadze, A., Borodovsky, M. & Stanke, M. BRAKER1: Unsupervised RNA-Seq-Based Genome Annotation with GeneMark-ET and AUGUSTUS. *Bioinformatics* **32**, 767–769 (2016).
3. Simão, F. A., Waterhouse, R. M., Ioannidis, P., Kriventseva, E. V. & Zdobnov, E. M. BUSCO: assessing genome assembly and annotation completeness with single-copy orthologs. *Bioinformatics* **31**, 3210–3212 (2015).
4. Conesa, A. & Götz, S. Blast2GO: A comprehensive suite for functional analysis in plant genomics. *Int. J. Plant Genomics* 619832 (2008).

**E. Whole genome synteny and orthologous analysis of *V. riparia* Manitoba 37 and *V. vinifera*.**

1. Soderlund, C., Bomhoff, M. & Nelson, W. M. SyMAP v3.4: a turnkey synteny system with application to plant genomes. *Nucleic Acids Res.* **39**, e68–e68 (2011).
2. Ensembl Plants. Available at: http://plants.ensembl.org/index.html. (Accessed: 1st June 2019).
3. Sook Jung et al., 15 years of GDR: New data and functionality in the Genome Database for Rosaceae. *Nucleic* *Acids Res.* **47,** D1137-D1145 (2018).
4. Li, L., Stoeckert, C. J. & Roos, D. S. OrthoMCL: Identification of Ortholog Groups for Eukaryotic Genomes. *Genome Res.* **13**, 2178–2189 (2003).
5. Wang, Y., Coleman-Derr, D., Chen, G. & Gu, Y. Q. OrthoVenn: a web server for genome wide comparison and annotation of orthologous clusters across multiple species. *Nucleic Acids Res*. **43**, W78–W84 (2015).
6. Kumar, S., Stecher, G. & Tamura, K. MEGA7: Molecular Evolutionary Genetics Analysis Version 7.0 for Bigger Datasets. *Mol. Biol. Evol.* **33**, 1870–1874 (2016).

**F. Plant transcription factors prediction and phylogenetic tree of gene families**

1. Kumar, S., Stecher, G. & Tamura, K. MEGA7: Molecular Evolutionary Genetics Analysis Version 7.0 for Bigger Datasets. *Mol. Biol. Evol.* **33**, 1870–1874 (2016).
2. Yu, G., Smith, D. K., Zhu, H., Guan, Y., Lam, T. T-Y. ggtree: an R package for visualization and annotation of phylogenetic trees with their covariates and other associated data." *Meth. Ecol.Evol.* 8, 28-36 (2017).

Bodenhofer U., Bonatesta, E., Horejs-Kainrath, C., & Hochreiter, S. msa: an R package for multiple sequence alignment.” *Bioinformatics* **31,** 3997–3999. (2015).

**G. Alignment of F2 GBS markers to *V. riparia* Manitoba 37 and *V. vinifera* PN40024 12X.2**

1. Eddy S. R. Profile hidden Markov models. *Bioinformatics* **14**, 755-763 (1998).
2. Finn, R. D. et al. "Pfam: the protein families database." *Nucleic acids research* **42**, D222-D230 (2013).
3. Zhang, H., Meltzer, P. & Davis, S. RCircos: an R package for Circos 2D track plots. BMC Bioinformatics **14**, 244 (2013).
